# Analysis of *Streptococcus dysgalactiae* subspecies *equisimilis* gene transcripts during experimental primate necrotizing myositis

**DOI:** 10.1128/mbio.01349-25

**Published:** 2025-07-22

**Authors:** Jesus M. Eraso, Randall J. Olsen, S. Wesley Long, Ahmad Faili, Samer Kayal, James M. Musser

**Affiliations:** 1 Laboratory for Molecular and Translational Human Infectious Diseases Research, Center for Infectious Diseases, Houston Methodist Research Institute 167626 , Houston, Texas, USA; 2 Department of Pathology and Genomic Medicine, Houston Methodist Hospital 23534 , Houston, Texas, USA; 3 Department of Pathology and Laboratory Medicine, Weill Medical College of Cornell University 207092, New York, New York, USA; 4 CHU de Rennes, Service de Bactériologie et d’Hygiène Hospitalière INSERM, CIC 1414 538067 , Rennes, Brittany, France; 5 Université Rennes 1, Faculté de Pharmacie 27079, Rennes, Brittany, France; 6 OSS-Oncogenesis, Stress, and Signaling INSERM 1242 679935, Rennes, Brittany, France; 7 Université Rennes 1, Faculté de Médecine 27079, Rennes, Brittany, France; GSK Vaccines, Siena, Italy

**Keywords:** *Streptococcus dysgalactiae*, RNAseq, primate, pathogenesis, emerging clone

## Abstract

*Streptococcus dysgalactiae* subspecies *equisimilis* (SDSE) is a gram-positive bacterial pathogen capable of causing various infections in humans. Recently, isolates of SDSE *emm* type *stG62647* have emerged as a cause of severe invasive infections, including necrotizing myositis. However, the molecular processes underlying these infections remain poorly understood. To address this gap, we performed RNAseq analysis to examine SDSE gene transcript levels during experimental necrotizing myositis infection in non-human primates, animals phylogenetically closely related to humans. We analyzed the transcriptomes of two related SDSE *stG62647* human isolates (MGCS36044 and MGCS36089) during necrotizing myositis infection in six non-human primates. The transcriptome from *in vitro* growth in nutrient-rich media differed considerably from that of SDSE bacteria grown *in vivo* in experimental necrotizing myositis, with 254 genes differentially expressed, indicating extensive genetic adaptation in infected skeletal muscle. Notably, we observed a marked upregulation of *ihk-irr* genes encoding a two-component regulatory system that promotes evasion of phagocytosis and resistance to killing by human polymorphonuclear leukocytes in *Streptococcus pyogenes*. Similarly, genes comprising the *sag* operon encoding the streptolysin S cytolytic toxin virulence factor demonstrated very high transcript abundance *in vivo*. Additionally, we present evidence that a 40-nt deletion in *fasB* alters expression of *ska*, encoding streptokinase. Collectively, our data provide new insights into the SDSE genes transcribed *in vivo*, thereby enhancing our understanding of the molecular basis of pathogen and primate host interactions. The SDSE genes identified in this study offer promising targets for future studies on molecular pathogenesis and therapeutic interventions.

**IMPORTANCE:**

*Streptococcus dysgalactiae* subspecies *equisimilis* (SDSE) has emerged as an increasingly important bacterial pathogen causing serious invasive infections in humans worldwide. Despite its clinical importance, the mechanisms through which SDSE causes infections remain poorly understood, and no licensed vaccine currently exists. SDSE can cause necrotizing myositis, an infection with high morbidity and mortality. We used a primate infection model and bacterial transcriptome analysis to gain new understanding of the molecular events contributing to SDSE pathogenesis in necrotizing myositis. Our results provide extensive new information about the transcriptome of SDSE *in vivo* and reveal numerous potential targets for future therapeutic and vaccine research.

## INTRODUCTION

One fundamental goal of contemporary infectious disease research is to understand the molecular interactions between microbial pathogens and their hosts. Analyses of this type are especially important to perform when successful new pathogens emerge and cause widespread human disease. Severe invasive infections caused by the gram-positive bacterial pathogen *Streptococcus dysgalactiae* subspecies *equisimilis* (SDSE) have been reported relatively recently, with increasing frequency in many countries in Europe, Asia, and elsewhere (1–11). SDSE is genetically closely related to *Streptococcus pyogenes* (group A streptococcus, GAS), a well-characterized pathogen. Compared to SDSE, GAS virulence mechanisms are better understood (12–26) due to extensive research on the well-documented dramatic changes in disease frequency and severity (27–33), advanced genetic tools for generating isogenic and transposon-containing mutant strains (34–42), and the availability of established animal infection models (43–52), including several non-human primate (NHP) models that are excellent mimics of human diseases (53–55).

SDSE causes a variety of severe human infections, including necrotizing fasciitis/myositis, bacteremia, osteoarticular infections, and toxic shock syndrome (6, 56, 57), often associated with high morbidity and mortality (6, 58). Thus, understanding the molecular basis of SDSE virulence may lead to improved treatments and better clinical outcomes. To investigate SDSE disease pathogenesis at the molecular level, we used an NHP model of necrotizing myositis infection. NHPs are phylogenetically closely related to humans, and our laboratory has demonstrated that the cynomolgus macaque is the gold-standard animal model for investigating streptococcal molecular pathogenesis (31, 37, 48, 54). Furthermore, GAS *in vivo* transcriptome analysis using NHP models (54) has successfully generated extensive insights into molecular pathogenesis and identified many potential therapeutic targets.

Our primary hypothesis is that SDSE strains undergo extensive transcriptome changes during necrotizing myositis in primates compared to growth *in vitro* in rich media, reflecting adaptation to host-specific conditions during necrotizing myositis. We studied two clonally related SDSE clinical isolates (MGCS36044 and MGCS36089) belonging to a recently emerged SDSE *emm* type (*stG62647*) that is responsible for severe clinical manifestations in humans, including necrotizing soft-tissue infections in multiple countries (2–4, 6–8, 10). Notably, strain MGCS36089 was previously used as a reference strain in mouse virulence assays (59). We determined *in vivo* transcript values representing 81–83% genomic content in these two SDSE isolates. Our analysis revealed considerable transcriptome differences *in vivo*, with 254 genes differentially expressed, indicating extensive genetic adaptation in infected NHP skeletal muscle. We discovered that *ihk-irr* genes encoding a two-component regulatory system (TCS) in GAS that controls a coordinated response promoting evasion of phagocytosis and resistance to killing by human polymorphonuclear leukocytes were strikingly upregulated. Similarly, genes comprising the *sag* operon encoding the streptolysin S cytolytic toxin virulence factor had very high transcript abundance *in vivo*. Collectively, our findings address a critical knowledge gap concerning an emerging pathogen causing severe human infections and contribute new understanding of the pathobiology of SDSE.

## MATERIALS AND METHODS

### Bacterial isolates and growth media

The two SDSE strains studied (MGCS36044 and MGCS36089) are genetically closely related, differing from one another by only 624 single nucleotide polymorphisms in the core genome (see below). In general, with a few important exceptions noted below, they have identical alleles at genes known to encode major regulators, such as TCS, and stand-alone regulatory genes, such as *mga*. Similarly, the two strains each had 14 nucleotides in a homopolymeric tract known to influence the level of pilus gene transcripts and alter virulence (59). The strains were grown *in vitro* in Todd-Hewitt broth (Becton, Dickinson & Co.) supplemented with 0.2% yeast extract (THY medium), as previously described (54). Trypticase soy agar supplemented with 5% sheep red blood cells (Becton, Dickinson & Co.) was used as solid media for *in vitro* growth of SDSE strains.

### RNAseq library preparation for samples grown *in vitro*

We used a previously described protocol for RNAseq analysis of bacteria grown *in vitro* (59). SDSE bacteria were grown and prepared in quadruplicate. Strains MGCS36044 and MGCS36089 were grown in THY broth at 37°C and collected at optical density OD = 1 (mid-exponential phase [ME]) and OD = 2 (early stationary phase; [ES]). Growth curves for both strains were previously performed in quintuplicate to determine the collection ODs. The cDNA libraries were sequenced with an Illumina NextSeq 500/550 instrument.

### NHP skeletal muscle samples

We used a previously described NHP necrotizing myositis infection model originally formulated for GAS molecular pathogenesis studies (48). Skeletal muscle biopsies from SDSE-infected NHPs were obtained as described for the RNAseq analysis (31, 37, 48, 54). Initially, eight cynomolgus macaques (2.1 to 5.2 years of age, 1.6 to 3.1 kg of body weight, males and females) were infected. Briefly, animals were sedated, the injection site was marked on the quadriceps muscle, and 1 × 10^8^ CFU/kg body weight of the indicated SDSE strain was inoculated intramuscularly at a uniform depth. Isolates MCGS36044 and MCGS36089 were used because they are genetically representative of contemporary epidemic serotype *emm*-type *stG62647* SDSE strains, their genomes have been sequenced to closure, they have been used previously in several mouse necrotizing myositis experiments (59, 60), and, with one important exception described below, they have wild-type alleles for all major transcriptional regulator (MTR) genes. These two isolates were recovered from the bone of patients diagnosed with osteitis. The NHPs were observed for 24 h after inoculation and necropsied. The infected thigh muscle was removed *en bloc*, and a 0.5 cm full-thickness transverse section was cut through the injection site with a TissueTek long trimming blade (Bevel). Multiple infected tissue samples (layers) weighing approximately 150 mg were excised radially in concentric sections from the infection site with an 8 mm biopsy punch (Fray Products). Samples used for RNAseq analysis were submerged immediately in 2 mL RNase-free screw cap tubes (Corning) containing 750 µL 1× RNA/DNA Shield (Zymo Research) and flash-frozen. The layers were numbered 1 through 5, with layer 1 corresponding to the inoculation site and layer 5 representing the layer farthest from the inoculation site (Fig. S1). Muscle samples were also collected in sterile phosphate-buffered saline (MP Biochemicals) for quantitative bacterial culture.

### RNAseq library preparation and sequencing of *in viv*o samples

RNAseq library preparation was conducted as described previously for GAS necrotizing myositis experiments (54). Five biopsy sections corresponding to layers 1–5 from two NHPs were processed daily for RNA extraction. A total of 80 samples were processed (eight NHPs/two SDSE isolates/five layers). Tissue samples were thawed on ice and processed as described (54) with the following modifications. Each sample was divided into three parts after homogenization, and after nucleic acid purification, the three samples were concentrated with RNA Clean & Concentrator (Zymo Research). RNA quality was assessed with an Agilent 6000 Nano Kit (Agilent) and an Agilent 2100 Bioanalyzer. RNA integrity number (RIN) values for the 80 samples ranged from 2.6 to 7.9, and the corresponding DV200 (percentage of RNA fragments longer than 200 nucleotides) values ranged from 54 to 80. The concentration of total RNA was determined with a Qubit RNA BR Assay Kit (Thermo Fisher Scientific). Microbenrich (Invitrogen) was used to enrich SDSE RNA from the mixture of SDSE and NHP RNA. Depletion of rRNA was performed with the NEBNext rRNA Depletion Kit (bacteria), and each reaction was spiked with 1 µL of NEBNext V2 ribosomal RNA depletion solution (human/mouse/rat) (NEB). The efficacy of the ribosomal depletion procedure was assessed with an RNA 6000 Pico Kit (Agilent) and anAgilent 2100 Bioanalyzer. The incubation times for RNA fragmentation varied depending on the DV200 and RIN values: 60 (DV200 71–80/7 min), 18 (DV200 66–70/4 min), and two samples (DV200 54–65/3 min). cDNA libraries were made with the NEBNext Ultra II Directional RNA Library Prep Kit and barcoded with multiplex oligos for Illumina (1–48) (both from NEB). Samples were analyzed with a NovaSeq 6000 instrument and S1 flow cells (Illumina). The initial sequencing runs contained 27, 27, and 26 pooled cDNA libraries for a total of 80 libraries (8 NHPs × 5 biopsies [layers] × 2 strains). The RNAseq reads were mapped to the host genome (*Macaca fascicularis*_6.0) assembly using STAR (https://github.com/alexdobin/STAR) and to the SDSE genomes using EDGE-Pro (61). After identification of the NHPs and biopsy layers that yielded the highest number of reads mapping to the SDSE genomes, repeat sequencing was performed for nine pooled cDNA libraries corresponding to MGCS36044 (four runs) and nine pooled libraries corresponding to MGCS36089 (three runs).

### RNAseq data analysis

fastQ files corresponding to *in vivo* and *in vitro* samples were demultiplexed, and adapter contamination and read-quality filtering were performed with Trimmomatic (62). Sequence data quality was determined with FastQC (63), and the mean per-base sequence quality was ≥Q30 for all libraries. The RNAseq reads were mapped to the cognate closed and circularized reference strain genome (60) with EDGE-Pro (61). In the initial round of sequencing, the 80 cDNA libraries produced between 50M and 115M reads for the *in vivo* samples (two strains/eight NHPs/five layers), whereas the second round of sequencing yielded, on average, between 160 and 260M reads for four sequencing runs in the case of MGCS36044 and three runs for MGCS36089 (Table S1; Fig. S2A and B). For each MGCS strain, samples from two of the initial eight NHPs were not processed further due to insufficient sequencing reads mapping to SDSE. Principal component analysis (PCA) was performed with PCAexplorer (64). For the PCA analysis, the *in vivo* samples had one biological replicate, and the *in vitro* samples were analyzed in quadruplicate. Venn diagrams were generated with RStudio (RStudio: Integrated Development Environment for R, Posit Software, PBC, Boston, MA; http://www.posit.co/).

### Downsampling an MGCS36044 replicate grown *in vitr*o

A FASTQ file corresponding to one replicate from SDSE strain MGCS36044 grown *in vitro* and containing approximately 28 M reads, which generated full genome coverage, was randomly downsampled using fastq-sample release 1.0.1 (https://github.com/f plazaonate/fastq-sample). This generated 22 new FASTQ files which contained the following in decreasing order: 2 million and (in thousands) 150, 125, 100, 90, 80, 70, 60, 50, 40, 30, 20, 10, 9, 8, 7, 6, 5, 4, 3, 2, and 1 reads (Fig. S3). RNAseq reads corresponding to both the original and the resulting 22 downsampled files were mapped to the strain MGCS36044 reference genome (60) using EDGE-Pro (61) to generate 23 sets of normalized counts. Subsequently, transcript abundance ranks were determined, and genes were organized into transcript abundance quartiles (Q1—highest abundance/Q4—lowest abundance). Twenty-two pairwise comparisons of transcriptome values by rank were performed by comparing each downsized file to an isogenic file derived from the original. For each comparison, the number of genes identified and Q1 correspondence were obtained (Fig. S3A), and *R*^2^ values were calculated (Fig. S3B). The sizes of downsampled files are presented in Fig. S3C.

### SDSE chromosome polymorphism analysis

Polymorphisms in the two reference SDSE chromosomes were identified by mapping Illumina paired-end reads to the genome of reference strain MGCS36044 with SMALT (https://www.sanger.ac.uk/tool/smalt/). Polymorphisms identified between the aligned reads and the reference genome (MGCS36044) were identified with FreeBayes (https://doi.org/10.48550/arXiv.1207.3907), as previously described (59). Reverse mapping of Illumina reads was also performed using MGCS36089 as the reference strain, and the results were essentially identical.

## RESULTS

### Transcriptome analysis of two *stG62647* SDSE strains grown *in vitro* in rich media

To test our hypothesis that SDSE strains undergo extensive transcriptome changes during necrotizing myositis in primates compared to growth *in vitro* in rich media, we studied the transcriptomes of *stG62647* strains MGCS36044 and MGCS36089 grown *in vitro* in THY broth and harvested cells at mid-exponential (ME) and early stationary (ES) growth phases. We used an RNAseq protocol (Fig. S1B) that has been used extensively for SDSE and GAS (17, 54, 59). Bacteria from each strain were separately analyzed in quadruplicate, and transcript levels were determined at both growth phases. The transcript values were ranked from highest to lowest abundance and presented in Table S2A and B (strain MGCS36044) and Table S2C and D (strain MGCS36089).

**Fig 1:**
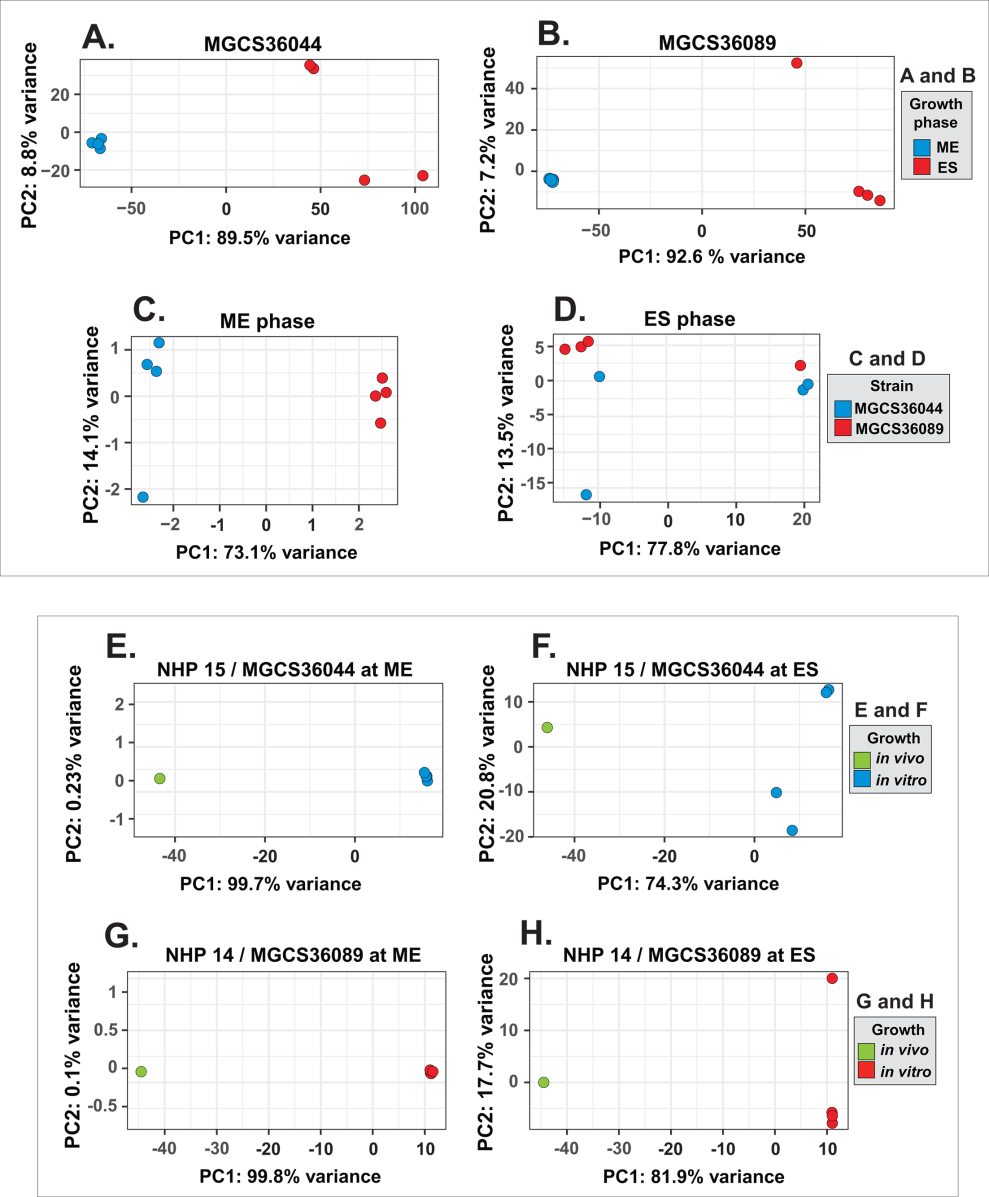
Principal component analysis of SDSE gene expression during *in vitro* and *in vivo* growth. Strains were grown *in vitro* in quadruplicate to mid-exponential (ME) and early stationary (ES) phases and *in vivo* as single replicates in skeletal muscle tissue from two non-human primates (NHPs), NHPs 15 (MGCS36044) and 14 (MGCS36089). These two NHPs were selected for analysis based on the highest number of SDSE genes with mapped reads. Top panel: growth *in vitro*. Thirteen genes were excluded due to low expression. (A) MGCS36044 (1,973 genes) grown at ME (blue) and ES (red). (B) MGCS36089 (1,984 genes) grown at ME (blue) and ES (red). For both isolates, ME replicates clustered together and separated from the ES replicates along principal component 1 (PC1) (C and D). MGCS36044 (blue) and MGCS36089 (red) grown at ME (**C**) and ES (**D**). A total of 1,954 genes were shared between both isolates. Bottom panel: *in vivo* vs. *in vitro* growth. A total of 1,662 genes (MGCS36044) and 1,630 (MGCS36089) had mapped reads *in vivo.* (E and F) MGCS36044 grown *in vivo* (green) and *in vitro* (blue) at ME and ES. (G and H) MGCS36089 grown *in vivo* (green) and *in vitro* (red) at ME and ES. There was a clear separation between replicates grown *in vivo* and *in vitro* along PC1.

Comparing ME to ES phases, we found 697 (35%) and 976 (49%) gene transcripts upregulated (using a twofold cutoff) and 279 (14%) and 251 (13%) genes downregulated in MGCS36044 and MGCS36089, respectively. The transcript values for the 58 putative *vir* genes in strain MGCS36044 are shown in Table 1, with analogous data for MGCS36089 in Table S3. Genes with the highest fold change between ME and ES phases included the *fas* operon genes, *nga* and *slo*, *mga*, adhesin genes, such as *lmb* and *fbp*, and *scpA*. In contrast, genes with the lowest fold change included the *sag* operon genes, *hylB*, and *pulA* (Table 1). Thus, substantial transcriptome changes were observed in the ES phase for these genetically related SDSE strains, as expected. These results are consistent with the PCA analysis (Fig. 1A and B) and previous data from a larger number of *stG62647* isolates (59).

**TABLE 1:** Expression of MGCS36044 virulence genes during two *in vitro* growth phases

No.	Locus tag	Gene	Function	MGCS36044 ME RPKMs^*a*^	MGCS36044 ES RPKMs^*b*^	Fold ME/ES^*c*^
1	MGCS36044_03866	*fasX*	FasBCA regulatory RNA	3,374.5	332.3	10.2
2	MGCS36044_03868	*fasA*	TCS**^*d*^** response regulator	309.5	58.3	5.3
3	MGCS36044_03994	*slo*	Streptolysin O	78.8	15.5	5.1
4	MGCS36044_03998	*nga*	Secreted nicotinamide adenine dinucleotide	51.3	10.5	4.9
5	MGCS36044_00514	*mga*	M protein trans-acting positive regulator Mga	304.8	67.5	4.5
6	MGCS36044_02062	*lmb*	Bifunctional metal ABC transporter/laminin binding protein	18.3	4.3	4.2
7	MGCS36044_03870	*fasC*	TCS histidine kinase	193.8	48.0	4.0
8	MGCS36044_00378	*fbp*	Fibronectin-binding protein	861.3	216.3	4.0
9	MGCS36044_02058	*scpA*	C5a peptidase SCP A	53.8	13.5	4.0
10	MGCS36044_03614	*shr*	Heme-binding secreted protein	94.8	28.3	3.3
11	MGCS36044_01134	*silCR*	Streptococcal invasion locus auto-inducing pheromone peptide	1.0	0.3	3.3
12	MGCS36044_00516	*emm*	Cell surface M protein	2,061.3	683.8	3.0
13	MGCS36044_03616	*isp2*	CHAP domain-containing immunogenic protein	2,174.8	1,015.8	2.1
14	MGCS36044_00946	*hlyX*	Hemolysin family protein	381.3	181.3	2.1
15	MGCS36044_02866	*slr*	InlA-like streptococcal leucine rich lipoprotein	6.8	3.3	2.0
16	MGCS36044_03700	*cppA*	CppA family putative C3-glycoprotein degrading protein	464.8	239.5	1.9
17	MGCS36044_00362	*rofA*	Pilus transcriptional regulator	230.3	138.8	1.7
18	MGCS36044_04274	*htrA*	Trypsin-like serine protease	1,195.5	734.8	1.6
19	MGCS36044_00880	*covS*	TCS histidine kinase	584.5	391.0	1.5
20	MGCS36044_03298	*liaF*	LiaFSR membrane component protein	155.3	104.3	1.5
21	MGCS36044_01140	*silE*	Streptococcal invasion locus pheromone	5.0	3.5	1.4
22	MGCS36044_03296	*liaS*	LiaFSR histidine kinase	237.5	169.0	1.4
23	MGCS36044_01138	*silD*	Streptococcal invasion locus pheromone secretion accessory protein	3.3	2.5	1.3
24	MGCS36044_03294	*liaR*	LiaFSR response regulator protein	252.8	203.3	1.2
25	MGCS36044_03908	*fbpB*	Cell surface fibronectin binding protein	34.5	28.0	1.2
26	MGCS36044_00878	*covR*	TCS response regulator	1,287.8	1,050.0	1.2
27	MGCS36044_02290	*ciaH*	TCS histidine kinase	467.0	388.3	1.2
28	MGCS36044_03926	*speG*	Streptococcal pyrogenic exotoxin (G)	48.3	40.8	1.2
29	MGCS36044_03140	*yesM*	TCS histidine kinase	141.5	121.0	1.2
30	MGCS36044_03142	*yesN*	TCS response regulator	104.3	89.5	1.2
31	MGCS36044_00526	*ska*	Secreted streptokinase	617.3	542.3	1.1
32	MGCS36044_02292	*ciaR*	TCS response regulator	351.0	333.8	1.1
33	MGCS36044_01240	*vicK*	TCS histidine kinase	572.5	558.0	1.0
34	MGCS36044_01508	*srrG*	Streptolysin S small regulatory RNA	8,556.8	8,467.3	1.0
35	MGCS36044_01238	*vicR*	TCS response regulator	470.3	489.5	−1.0
36	MGCS36044_01062	*mtsR*	Metal-dependent transcriptional regulator MtsR	407.0	425.3	−1.0
37	MGCS36044_02840	*spg*	Extracellular cell surface IgG-binding protein	4,110.3	4,459.0	−1.1
38	MGCS36044_02048	*irr*	TCS response regulator	111.3	120.8	−1.1
39	MGCS36044_03944	*perR*	Peroxide-responsive transcriptional repressor	780.0	877.8	−1.1
40	MGCS36044_02050	*ihk*	TCS histidine kinase sensor	110.8	132.3	−1.2
41	MGCS36044_03208	*trxS*	TCS histidine kinase	93.8	116.5	−1.2
42	MGCS36044_03808	*gapA*	Glyceraldehyde-3-phosphate dehydrogenase	17,642.5	22,529.0	−1.3
43	MGCS36044_03206	*trxR*	TCS response regulator	109.3	162.8	−1.5
44	MGCS36044_02052	*isp*	Secreted CHAP domain-containing immunogenic protein	129.0	203.0	−1.6
45	MGCS36044_01126	*silA*	TCS response regulator	37.0	67.0	−1.8
46	MGCS36044_01194	*ccpA*	Catabolite control protein	484.3	1,041.8	−2.2
47	MGCS36044_01524	*sagG*	Streptolysin S export protein	1,426.0	4,089.8	−2.9
48	MGCS36044_01528	*sagI*	Streptolysin S export permease protein	1,719.5	4,993.5	−2.9
49	MGCS36044_01526	*sagH*	Streptolysin S export permease	1,559.3	4,623.3	−3.0
50	MGCS36044_01518	*sagD*	Streptolysin S biosynthesis protein	1,320.8	3,970.0	−3.0
51	MGCS36044_01520	*sagE*	Streptolysin S self-immunity protein	1,486.5	4,472.5	−3.0
52	MGCS36044_01516	*sagC*	Streptolysin S biosynthesis protein	1,431.3	4,353.3	−3.0
53	MGCS36044_01522	*sagF*	Streptolysin S biosynthesis protein	1,480.5	4,922.5	−3.3
54	MGCS36044_00534	*pulA*	Cell surface pullulanase	29.8	109.5	−3.7
55	MGCS36044_01514	*sagB*	Streptolysin S biosynthesis protein	1,649.3	7,113.3	−4.3
56	MGCS36044_01410	*hylB*	Secreted hyaluronate lyase	122.8	997.0	−8.1
57	MGCS36044_01510	*sagA*	Streptolysin S precursor protein	4,498.8	69,008.0	−15.3
58	MGCS36044_01512	*sagA_RNA*	sagA RNA	6,051.0	99,748.5	−16.5

^*a*^ RPKMs correspond to the mean of four replicates. ME, mid-exponential phase.

^*b*^ ES, early stationary phase.

^*c*^ Fold was calculated by dividing the mean RPKM value at the mid-exponential phase by the mean RPKM value at the early stationary phase.

^*d*^ TCS, two-component signal transduction system.

When comparing the two strains directly, we found only a small number of transcript-level differences at either growth phase. For example, only 21 (1%) and six (0.3%) gene transcripts were up- and downregulated at ME, respectively, and 45 (2.3%) and 20 (1%) at ES (ratio MGCS36044/MGCS36089). The relatively small number of differences in gene transcript levels observed between these clonally related strains is consistent with their close overall genomic relationship (59). However, several genes showed notable transcript-level differences between strains. For example, *ska* and *fasX* were highly upregulated in MGCS36044 compared to MGCS36089 (6- to 10-fold) across both growth phases, whereas *speG* encoding a streptococcal pyrogenic exotoxin was downregulated (2.5- to 3 fold) (Table 2). Additionally, *fbpB* encoding a fibronectin-binding protein was downregulated at both ME (~3-fold) and ES (−1.6-fold) in both strains. These results are consistent with the PCA analysis (Fig. 1C and D).

**TABLE 2:** Differentially expressed genes comparing *in vitro*-grown MGCS36044 and MGCS36089 at two growth phases^*g*^^,^^*i*^

No.	MGCS36044 locus tag	MGCS36089 locus tag	Gene	Virulence^*a*^	Signal^*b*^	Function	Growth phase	MGCS36044 rpkm^*c*^	MGCS36089 rpkm	Fold^*d*^ 36044/36089
1	MGCS36044_00526	MGCS36089_00524	*ska*	Virulence	Secreted	Secreted streptokinase	ME^*e*^	617.3	60.8	10.2
2	MGCS36044_03866	MGCS36089_03878	*fasX*	Virulence		FasBCA signal transduction system small RNA	ME	3,374.5	551.3	6.1
3	MGCS36044_00588	MGCS36089_00586	–^*h*^		Secreted	Putative protein	ME	501.5	146.5	3.4
4	MGCS36044_03908	MGCS36089_03920	*fbpB*	Virulence	Secreted	Fibronectin binding protein (B)	ME	34.5	98.5	−2.9
5	MGCS36044_03926	MGCS36089_03938	*speG*	Virulence	Secreted	Streptococcal pyrogenic exotoxin	ME	48.3	149.3	−3.1
6	MGCS36044_01776	MGCS36089_01764	–		Secreted	Mucin-binding protein	ME	5.5	23.5	−4.3
1	MGCS36044_03866	MGCS36089_03878	*fasX*	Virulence		FasBCA signal transduction system small RNA	ES^*f*^	332.3	33.3	10.0
2	MGCS36044_00526	MGCS36089_00524	*ska*	Virulence	Secreted	Secreted streptokinase	ES	542.3	81.8	6.6
3	MGCS36044_03212	MGCS36089_03224	*ugpB_1*		Lipo	Glycerol-3-phosphate ABC transporter	ES	400.0	121.8	3.3
4	MGCS36044_00588	MGCS36089_00586	–		Secreted	Putative protein	ES	1,562.3	670.8	2.3
5	MGCS36044_00518	MGCS36089_00518	–		Secreted	UshA family bifunctional 2',3'-cyclic-nucleotide 2'-phosphodiesterase/ 3'-nucleotidase	ES	305.8	135.5	2.3
6	MGCS36044_04014	MGCS36089_04028	*pepD_2*		Secreted	Dipeptidase PepD	ES	1,489.5	706.8	2.1
7	MGCS36044_03926	MGCS36089_03938	*speG*	Virulence	Secreted	Streptococcal pyrogenic exotoxin	ES	40.8	103.3	−2.5

^*a*^ Putative vir genes.

^*b*^ SignalP6-predicted. Lipo refers to secreted lipoprotein.

^*c*^ RPKMs refers to the mean of EDGE-pro normalized counts for four replicates.

^*d*^ Fold cutoff was ≥2.

^*e*^ ME, mid-exponential phase.

^*f*^ ES, early stationary phase.

^*g*^ Only virulence genes and genes with a predicted export signal sequence are shown. Originally, 1,986 and 1,997 genes were analyzed corresponding to MGCS36044 and MGCS36089, respectively. Thirteen genes with low or no expression values and only detected in one of the strains, were excluded, and 1,954 genes were shared by both strains.

^*h*^ "–" indicates genes with a locus tag but not a gene name.

^*i*^ Empty cells indicate genes that are either not virulent or do not have an export signal sequence.

### Primate infection and RNAseq analysis

We next examined the transcriptome profiles of two *emm* type *stG62647* SDSE strains (59, 60) in a primate model of necrotizing myositis. We used a validated infection protocol previously employed for MGAS2221, an *emm1* GAS isolate (54). Each NHP was concurrently infected with strain MGCS36044 in the right leg and strain MGCS36089 in the left leg (Fig. S1A). A crucial advantage of this infection protocol is that it significantly reduces the number of NHPs required for transcriptome analysis. The two SDSE strains were isolated from skeletal muscle biopsies from eight NHPs at 24 h post-infection (37, 42, 54). As previously described for NHPs infected with GAS, muscle biopsies were obtained in five concentric layers (L1 to L5), with L1 corresponding to the inoculation site and L5 to the outermost layer. These samples, designated as *in vivo*, were compared to those from the same strains grown *in vitro* in rich media.

SDSE transcript data were obtained by RNAseq analysis. Initially, 80 cDNA libraries (representing 8 NHPs × 5 layers × 2 strains) were sequenced, yielding 48 to 113 M total reads per biopsy layer (Fig. S2A). Depending on the biopsy layer, 39 to 71,590 reads mapped to the SDSE reference genome (data not shown). After pooling reads from all five biopsy layers per NHP (54), the total number of reads mapping to SDSE ranged from approximately 1,700 to 85,000 reads per NHP (Table S1). Despite enrichment for pathogen transcripts during RNA extraction and preparation, the vast majority of reads not mapping to SDSE mapped to the primate host (*M. fascicularis*), as expected based on our prior GAS study (54).

**Fig 2:**
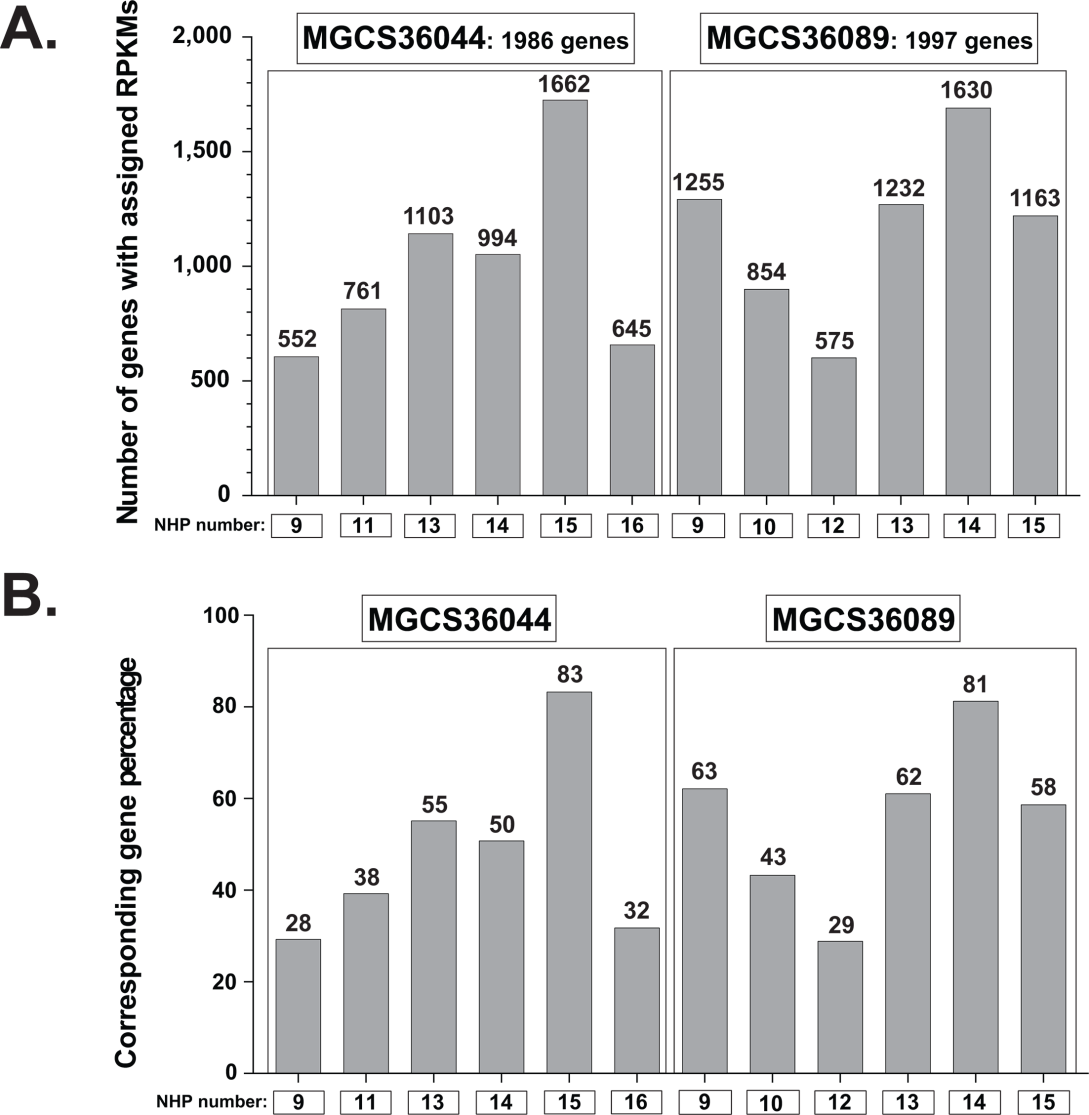
Abundance and genome coverage of the MGCS36044 and MGCS36089 genes during *in vivo* infections in six NHPs. Individual NHP identifiers are shown below each graph. Two NHPs were excluded from analysis due to an insufficient number of reads mapping to each strain. (A) The number of MGCS36044 genes (left panel) and MGCS36089 (right panel) with mapped reads are shown for the six NHPs. (B) Percent genome coverage was calculated for 1,986 (MGCS36044) and 1,997 (MGCS36089) genes, excluding those encoding rRNAs, tRNAs, and disrupted genes.

We performed additional sequencing to increase the number of reads mapping to the SDSE isolates and thereby increase the coverage of the pathogen genome. To maximize efficiency and decrease cost, we excluded biopsies from two NHPs yielding fewer than 7,000 total reads. This exclusion was based on our prior GAS study (54), with the number of DNA sequencing runs formulated according to Haas et al. (65). Thus, cDNA libraries corresponding to nine biopsy layers obtained from six NHPs were re-sequenced. After re-sequencing, a total of 90,000 to 1 M reads mapped to the genome of SDSE strain MGCS36044 and 170,000 to 664,000 to the genome of SDSE strain MGCS36089 (Table S1). The distribution of SDSE genes to which the reads mapped and the corresponding percent genome coverage are shown in Fig. 2; Table S1 and Fig. S2B. Despite multiple sequencing runs to increase the number of SDSE transcripts obtained for each animal, transcripts from 324 genes in strain MGCS36044 and 367 genes in strain MGCS36089 remained undetected likely due to low expression levels (Fig. S3) and host RNA contamination, even after microbial RNA enrichment (Fig. S2B, B and D).

**Fig 3:**
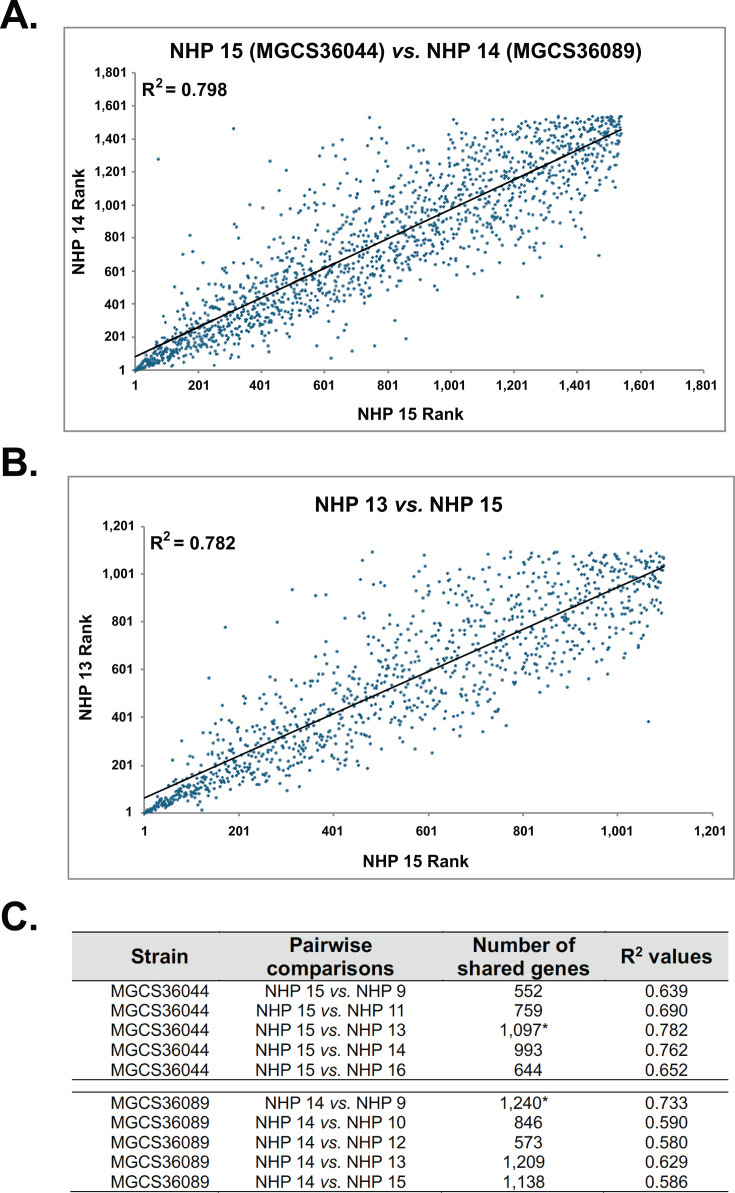
Pairwise comparisons of MGCS36044 and MGCS36089 transcriptomes from biopsies. Transcript abundance ranks were calculated based on normalized counts. The obtained *R*^2^ values are shown. Black lines indicate trend lines for each comparison. (A) SDSE strain comparison. Biopsies from NHP 15 (infected with MGCS36044) were compared to biopsies from NHP 14 (infected with MGCS36089). These two NHPs had yielded the highest number of SDSE genes with mapped reads, and the results correspond to 1,535 genes shared by both strains. (B) Comparison between biopsies from two NHPs infected with the same strain. Biopsies from NHPs 15 and 13 yielded the highest and second highest numbers of MGCS36044 genes with mapped reads, respectively. The results correspond to 1,097 SDSE shared genes. (C) Strain-specific *R*^2^ values corresponding to all samples. For each strain, the NHP sample with the largest number of genes with mapped reads [NHP 15 for MGCS36044 and NHP 14 for MGCS36089] was compared to all other samples. The number of shared genes and the corresponding *R*^2^ values are indicated. The comparisons involving the highest number of shared genes (*) yielded the highest *R*^2^ values.

Biopsies from NHPs 15 (MGCS36044) and 14 (MGCS36089) yielded the highest numbers of mapped SDSE genes, corresponding to 1,662 (83% genome coverage) (Table S4A) and 1,630 genes (81% genome coverage), respectively (Table S4B). These two SDSE strains shared transcripts from 1,535 mapped genes. When ranked by transcript abundance and compared to each other by pairwise comparison, the correlation of mapped reads was high (*R*^2^ = 0.798) (Fig. 3A). This result is consistent with these two *stG62647* strains having very similar *in vivo* gene transcript levels in infected primate skeletal muscle. Conversely, biopsies from NHPs 9 (MGCS36044) and 12 (MGCS36089) yielded the lowest numbers of genes with mapped reads, corresponding to only 28 and 29% genome coverages, respectively.

We performed pairwise analyses to test the hypothesis that gene transcript abundance was similar between samples from the same strain in the six NHPs analyzed. Samples collected from NHPs 15 and 13 (strain MGCS36044; 1,097 shared mapped genes) (Fig. 3B) and NHPs 14 and 9 (strain MGCS36089; 1,240 shared mapped genes) showed high correlation values (*R*^2^) 0.782 and 0.733, respectively (Fig. 3C). Overall, the *R*^2^ values for the 10 total comparisons ranged from 0.58 to 0.78 (Fig. 3C). These data indicate that these two related SDSE strains had similar *in vivo* transcriptomes in this primate model of necrotizing myositis and that there was a good correlation between samples obtained from NHPs infected with the same strain.

Because of their potential relevance to understanding molecular pathogenesis and developing future therapeutics, we examined transcript levels from putative *vir* genes and genes containing an export signal sequence. Compared to GAS, relatively little pathogenesis research has been done with SDSE. Thus, we categorized putative *vir* genes primarily based on existing molecular pathogenesis studies of GAS. Importantly, 14 and 13 putative *vir* genes were among the genes with the highest transcript levels for strains MGCS36044 and MGCS36089, respectively (Table S4A and B). Because biopsies from each NHP yielded a different number of SDSE gene transcripts, the number of detected *vir* genes ranged from 54 (of 1,662 total detected genes) to 29 (of 552 total detected genes) for strain MGCS36044 (Table S5A) and from 52 (of 1,631 total detected genes) to 29 (of 575 total detected genes) in the six NHPs infected with strain MGCS36089 (Table S5B).

**Fig 4:**
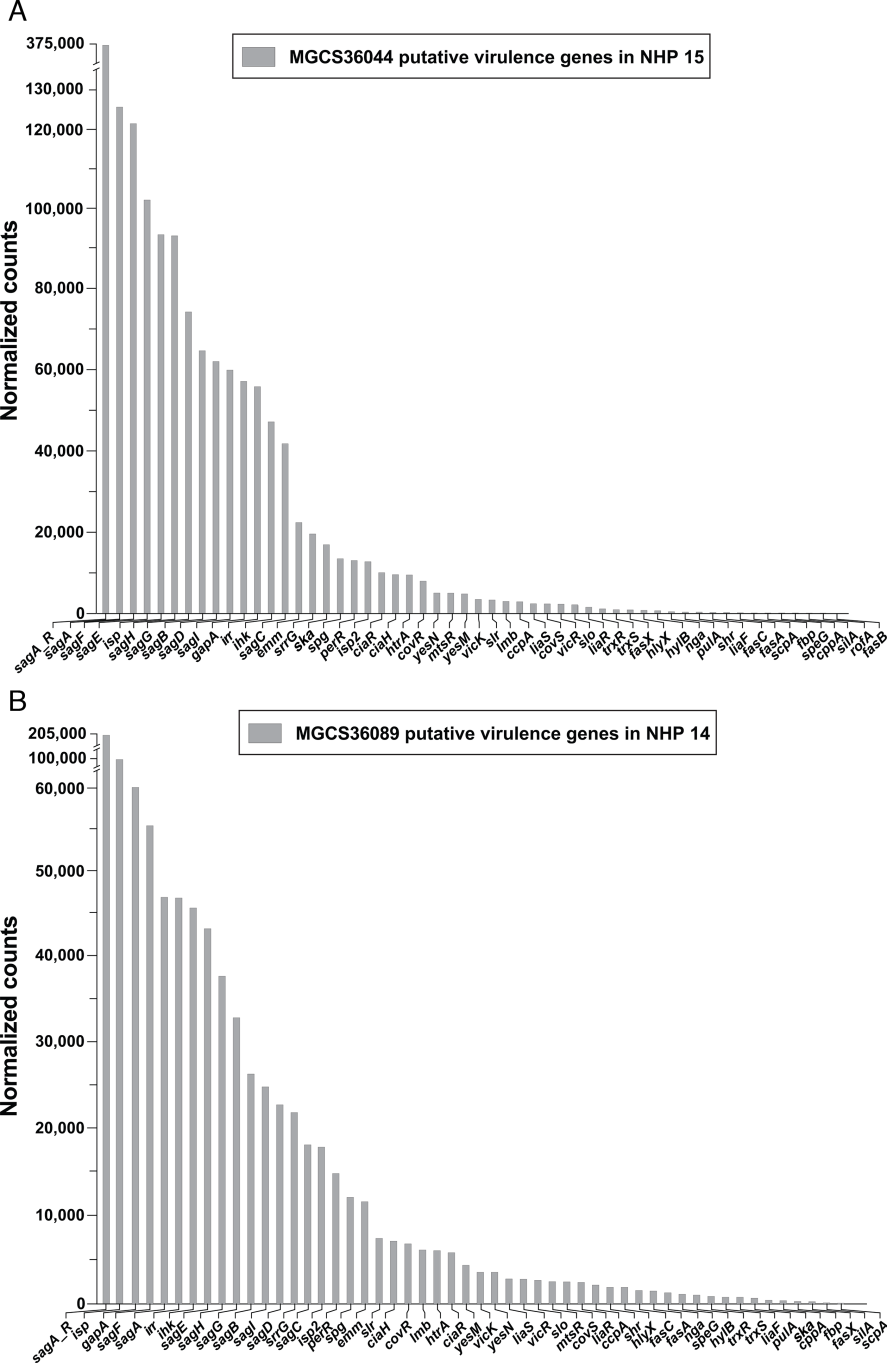
Expression of SDSE putative virulence genes during *in vivo* infection. Only data from NHPs with the highest number of genes with mapped reads are shown. (A) MGCS36044 in NHP 15. Fifty-four putative virulence genes out of a total of 1,662 genes had mapped reads. (B) MGCS36089 in NHP 14. Fifty-two putative virulence genes out of a total of 1,631 had mapped reads.

The *vir* gene transcripts corresponding to the NHPs with the highest number of detected SDSE genes are shown in Fig. 4A and B for strains MGCS36044 and MGCS36089, respectively. The *sag* operon genes, *gapA*, *ihk*, and *irr* exhibited very high transcript abundance in all biopsies from NHPs infected with both SDSE strains. Importantly, regardless of the infecting strain, transcript rank value correspondence was high for the majority of *vir* genes across biopsies (Table S5A and B). However, the *ska* gene encoding streptokinase was a notable exception. The *ska* transcript level in animals infected with strain MGCS36044 was much higher than in those infected with strain MGCS36089: for NHP 15 (infected with strain MGCS36044), *ska* ranked 75th in abundance, whereas for NHP 14 (infected with strain MGCS36089), *ska* ranked 1,299th (Fig. 4A and B and Table S5A and B). Consistent with its higher transcript level in MGCS36044-infected animals, *ska* transcripts were detected in all biopsies from this group but in biopsies from only three of six NHPs infected with strain MGCS36089.

Because genes encoding secreted proteins may be important candidates for therapeutic development, we also examined transcript levels of genes predicted to have export signal sequences. Transcripts from 27 to 86 genes were detected in biopsies from the six NHPs infected with MGCS36044 (Table S6A) and from 35 to 84 genes for the six infected with MGCS36089 (Table S6B). Regardless of the infecting strain, the correspondence in the transcript rank values was very high among the six NHPs. Genes encoding TlpA-family disulfide reductases had the highest transcript levels, along with *isp* and *isp2* (two virulence genes encoding immunogenic secreted proteins) and *mtsA* and *adcA* (genes involved in metal acquisition). In addition to *isp* and *isp2*, *emm* and *spg* were the most highly expressed *vir* genes containing export signal sequences. Notably, *ska* also had high transcript levels in MGCS36044 but not in MGCS36089. Collectively, vir genes and genes encoding inferred exported gene products showed similar levels of transcript abundance during *in vivo* infection with both strains in the six NHPs.

### SDSE transcript changes during adaptation to primate muscle

To test the hypothesis that gene transcript levels recovered from infected NHP skeletal muscle tissue differ significantly from those grown *in vitro*, we analyzed the relationship between transcript profiles of the *in vivo* and *in vitro* samples. This analysis aimed to identify individual genes and gene functional categories used by SDSE during NHP necrotizing myositis. Due to the close similarity in expression values between MGCS36044 and MGCS36089, we primarily present results for MGCS36044 and address key differences between the strains as needed.

**Fig 5:**
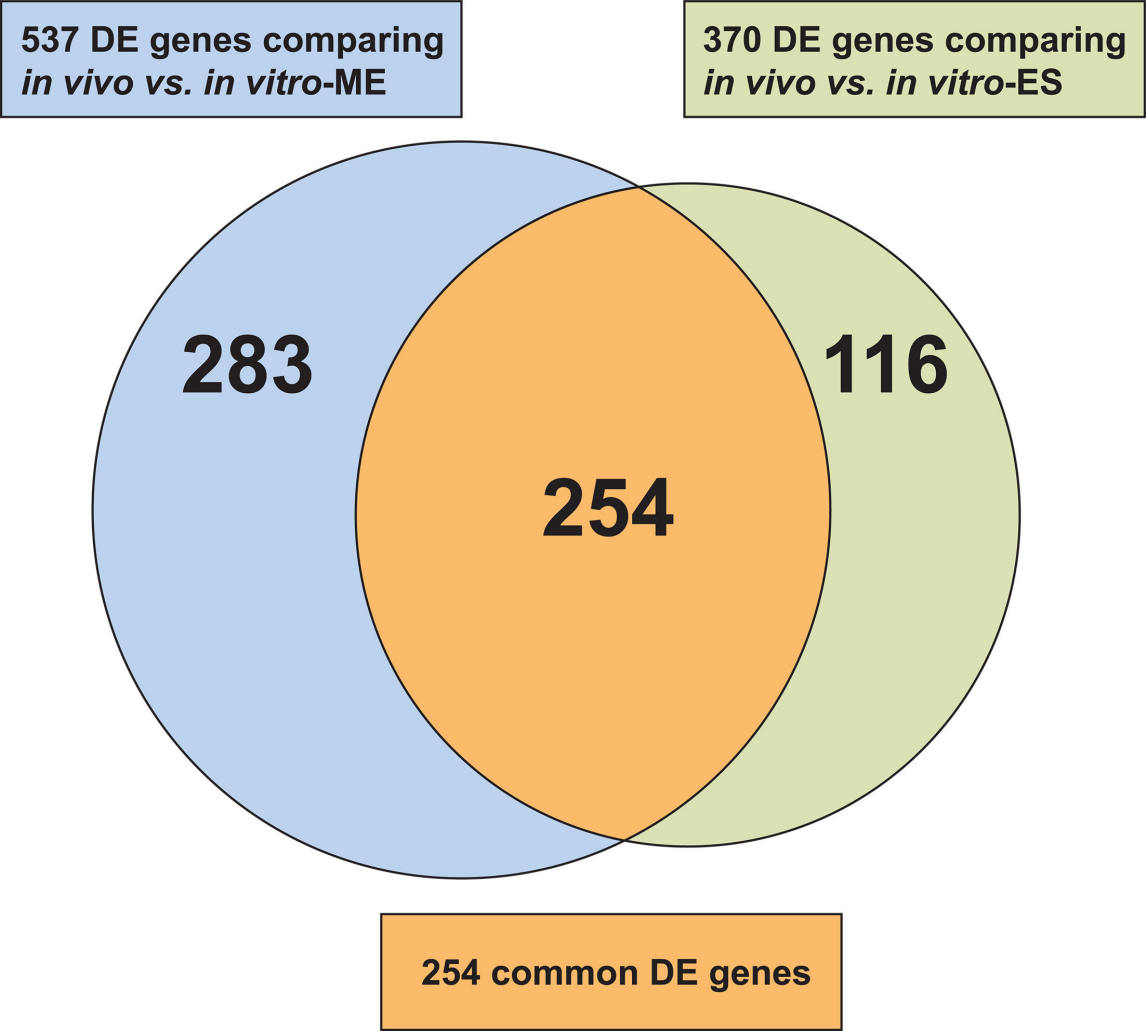
Differentially expressed MGCS36044 genes during *in vivo* growth in NHPs compared to *in vitro* growth. A total of 537 and 370 MGCS36044 genes from NHP 15 were differentially expressed *in vivo* relative to *in vitro* at ME (blue) and ES (green), respectively. A total of 254 genes (orange) were common to both categories. The fold change value cutoff and adjusted *P*-value cutoff were 2 and ≤0.05, respectively.

The PCA analysis revealed a large variance along PC1 when comparing *in vivo* samples to corresponding *in vitro* samples at ME (Fig. 1E and G) and ES (Fig. 1F and H), consistent across both strains. This finding indicated extensive transcriptome differences during NHP necrotizing myositis. Of the 1,655 MGCS36044 genes shared by the *in vivo* and *in vitro* data sets, ranking by transcript abundance and identifying differentially expressed (DE) genes using a twofold cutoff showed 537 DE genes (264 upregulated and 273 downregulated) *in vivo* versus *in vitro*-ME and 370 DE genes (185 and 185, respectively) *in vivo* versus *in vitro*-ES (Fig. 5). Similar values were obtained with MGCS36089. MGCS36044 had 254 DE genes common to both comparisons (Fig. 5 ; Table S7). Notably, 21 *vir* genes, 16 MTR genes, and 32 stress genes were upregulated *in vivo*. The relationship between transcript abundance ranks for the 54 *vir* MGCS36044 genes detected *in vivo* versus *in vitro* at both growth phases is shown in Table 3. Among DE *vir* genes, *isp*, *irr*, and *ihk* had much higher transcript abundance *in vivo* compared to *in vitro* at both growth phases. Notably, *ska* was more highly expressed *in vivo* (~4.5 fold) compared to *in vitro* in MGCS36044 but not in MGCS36089. In addition, two DE MTR genes with higher transcript abundance *in vivo* (MGCS36044_01350 and MGCS36044_01352) encoded a putative extracytoplasmic function (ECF) sigma factor (*rpoE*- σ^E^-σ^24^) and its anti-sigma factor, respectively. This gene pair is present in several streptococcal species but not in GAS. Thus, as expected, SDSE gene transcript abundance in infected NHP skeletal muscle tissue differed significantly from *in vitro* conditions.

**TABLE 3:** Comparison of MGCS36044 putative virulence genes during *in vivo* and *in vitro* growth at two phases^*e*^

No.	Gene	Function	*In vivo* rank^*a*^	*In vitro* ME rank^*b*^	ME fold^*c*^	No.	Gene	*In vitro* ES rank	ES fold^*d*^
1	***isp***	CHAP domain-containing immunogenic protein	14	1,160	**82.9**	1	***isp***	762	**54.4**
2	***irr***	TCS response regulator	27	1,226	**45.4**	2	***irr***	1,096	**40.6**
3	***ihk***	TCS histidine kinase sensor	31	1,228	**39.6**	3	***ihk***	1,044	**33.7**
4	***sagF***	Streptolysin S biosynthesis protein	10	144	**14.4**	4	***yesN***	1,243	**4.5**
5	***sagE***	Streptolysin S self-immunity protein	12	143	**11.9**	5	***ska***	316	**4.2**
6	***sagA_RNA***	sagA RNA	4	46	**11.5**	6	***emm***	257	**4.1**
7	***sagH***	Streptolysin S export permease protein	15	138	**9.2**	7	***slr***	1,652	**4.1**
8	***sagG***	Streptolysin S export protein	17	151	**8.9**	8	***ciaR***	468	**3.7**
9	***sagA***	Streptolysin S precursor	8	61	**7.6**	9	***lmb***	1,651	**3.7**
10	***sagD***	Streptolysin S biosynthesis protein	21	160	**7.6**	10	***yesM***	1,094	**3.7**
11	***sagB***	Streptolysin S biosynthesis protein	20	133	**6.7**	11	***ciaH***	412	**3.2**
12	***sagI***	Streptolysin S export permease protein	22	127	**5.8**	12	***sagF***	30	**3.0**
13	***ciaR***	TCS regulator protein	125	646	**5.2**	13	***sagE***	35	**2.9**
14	***ska***	Streptokinase	75	340	**4.5**	14	***slo***	1,610	**2.9**
15	***yesN***	TCS response regulator	278	1,245	**4.5**	15	***sagG***	39	**2.3**
16	***sagC***	Streptolysin S biosynthesis protein	36	149	**4.1**	16	***sagH***	33	**2.2**
17	***slr***	InlA-like streptococcal leucine rich protein	407	1,636	**4.0**	17	***sagD***	41	**2.0**
18	***yesM***	TCS histidine kinase	295	1,122	**3.8**	18	***perR***	196	**1.9**
19	***ciaH***	TCS histidine kinase	129	476	**3.7**	19	***htrA***	240	**1.8**
20	***lmb***	Bifunctional metal ABC transporter protein/laminin-binding protein	445	1,579	**3.5**	20	***liaS***	889	**1.7**
21	***perR***	Peroxide-responsive transcriptional repressor	105	271	**2.6**	21	***nga***	1,635	**1.7**
22	***slo***	Streptolysin O	562	1,352	**2.4**	22	***isp2***	172	**1.6**
23	***mtsR***	Metal-dependent transcriptional regulator	281	548	**2.0**	23	***shr***	1,549	**1.4**
24	***trxR***	TCS response regulator	704	1,233	**1.8**	24	***mtsR***	382	**1.4**
25	***emm***	Cell surface M protein	63	107	**1.7**	25	***trxS***	1,112	**1.4**
26	***liaS***	TCS histidine kinase	528	878	**1.7**	26	***sagI***	29	**1.3**
27	***trxS***	TCS histidine kinase	823	1,295	**1.6**	27	***trxR***	913	**1.3**
28	***nga***	NAD glycohydrolase	989	1,447	**1.5**	28	***liaR***	760	**1.3**
29	***liaR***	TCS response regulator	599	848	**1.4**	29	***scpA***	1,618	**1.3**
30	***pulA***	Pullulanase	1103	1,527	**1.4**	30	***fasC***	1,454	**1.2**
31	***htrA***	Trypsin-like serine protease	131	180	**1.4**	31	***fasA***	1,396	**1.1**
32	***hylB***	Secreted hyaluronate lyase	962	1,189	**1.2**	32	***covR***	166	**1.1**
33	***shr***	Heme-binding secreted protein	1,133	1,289	**1.1**	33	***speG***	1,486	**1.0**
34	***scpA***	C5a peptidase	1,280	1,443	**1.1**	34	***pulA***	1,146	**1.0**
35	***covR***	TCS response regulator	158	165	**1.0**	35	***liaF***	1,173	**1.0**
36	***speG***	Streptococcal pyrogenic exotoxin (G)	1,417	1,452	**1.0**	36	***sagC***	37	**1.0**
37	***ccpA***	Catabolite control protein	460	462	**1.0**	37	***sagB***	20	**1.0**
38	***silA***	TCS response regulator SilA	1,551	1,499	**1.0**	38	***hlyX***	847	**−1.1**
39	***isp2***	CHAP domain-containing immunogenic secreted protein	107	103	**1.0**	39	***fasB***	1,491	**−1.1**
40	***liaF***	TCS membrane component protein	1,137	1,093	**1.0**	40	***silA***	1,342	**−1.2**
41	***vicK***	TCS histidine kinase	392	376	**1.0**	41	***vicK***	305	**−1.3**
42	***vicR***	TCS response regulator	544	474	**−1.1**	42	***covS***	408	**−1.3**
43	***fasC***	TCS histidine kinase	1,169	985	**−1.2**	43	***rofA***	1,019	**−1.5**
44	***covS***	TCS histidine kinase	534	367	**−1.5**	44	***vicR***	339	**−1.6**
45	***spg***	Cell surface IgG-binding streptococcal protein (G)	100	67	**−1.5**	45	***fbp***	728	**−1.9**
46	***hlyX***	Hemolysin family protein	899	591	**−1.5**	46	***fasX***	471	**−1.9**
47	***fasB***	TCS histidine kinase	1,631	1,059	**−1.5**	47	***cppA***	650	**−2.3**
48	***fasA***	TCS response regulator	1,256	732	**−1.7**	48	***ccpA***	169	**−2.7**
49	***rofA***	Pilus transcriptional regulator	1,554	899	**−1.7**	49	***spg***	36	**−2.8**
50	***cppA***	Putative C3-glycoprotein degrading proteinase	1,500	478	**−3.1**	50	***sagA***	2	**−4.0**
51	***srrG***	Streptolysin S small regulatory RNA	69	14	**−4.9**	51	***sagA_RNA***	1	**−4.0**
52	***fbp***	Fibronectin-binding protein	1,375	237	**−5.8**	52	***gapA***	6	**−4.2**
53	***fasX***	FasBCA TCS small regulatory RNA FasX	891	78	**−11.4**	53	***srrG***	15	**−4.6**
54	***gapA***	Glyceraldehyde-3-phosphate dehydrogenase	25	1	**−25.0**	54	***hylB***	174	**−5.5**

^*a*^ Rank refers to the transcript abundance rank, and is based on RPKMs, where the highest transcript abundance corresponds to rank = 1.

^*b*^ For each gene, the RPKM value for the *in vitro* samples was the mean of the four replicates.

^*c*^ ME fold was calculated as the ratio between the *in vitro* rank at ME/*in vivo* rank. Positive values correspond to genes with higher transcript abundance *in vivo* (upregulated *in vivo*), whereas negative values indicate higher transcript abundance *in vitro* (downregulated *in vivo*).

^*d*^ ES fold was calculated as the ratio between the *in vitro* rank at ES/*in vivo* rank.

^*e*^ A total of 1,655 MGCS36044 genes were shared between the *in vivo* (one replicate collected from NHP 15) and *in vitro* samples (four replicates), which had been grown in rich media and collected at two time points, mid-exponential (ME) and early stationary (ES) phases of growth. A total of 23 and five genes were up- and downregulated *in vivo* compared to *in vitro* ME and 17 and eight, respectively, compared to *in vitro* ES.

### MGCS36044 and MGCS36089 transcripts and mouse virulence

MGCS36044 is more virulent than MGCS36089 in a mouse model of necrotizing myositis (59, 60). To investigate the molecular basis of this biological difference, we examined *vir* gene transcript levels in both strains. The transcript levels of most genes were very similar for both isolates, but *ska* transcript levels were much higher in MGCS36044 compared to MGCS36089 both *in vitro* (Table 2) and *in vivo* (Fig. 4A and B).

**Fig 6:**
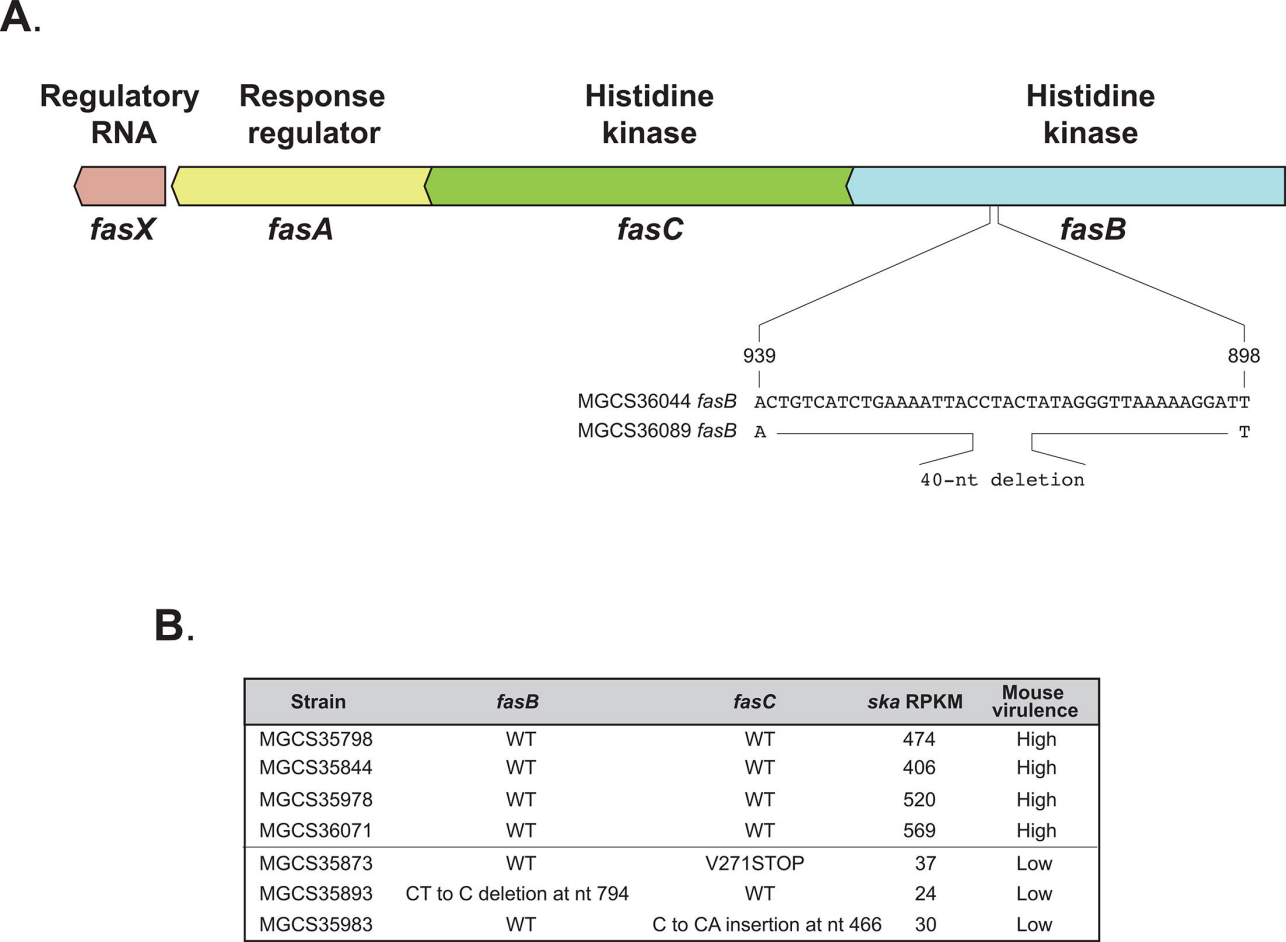
Organization of the *fasBCAX* operon and associated mutations. (A) Genes and corresponding gene products in the atypical *fasBCA* two-component system, which encodes two histidine kinases, in strains MGCS36044 and MGCS36089. In both strains, the operon is oriented toward the chromosomal origin of replication (*ori*). From left to right: (i) *fasX*, *fas* operon regulatory RNA; (ii) *fasA*, response regulator; (iii) *fasC*, histidine kinase; and (iv) *fasB*, histidine kinase. *fasB* from MGCS36089 contains a 40 nt internal deletion (from nt. 899 to 938, inclusive), which results in a frameshift at codon 300 of 447 total codons. (B) Summary of mutations in *fasB* or *fasC* corresponding to *ska* transcript abundance (in RPKMs) and virulence in a mouse model of necrotizing myositis (59). Data are shown for four strains lacking mutations in *vir* and *MTR* genes and three strains with mutations in either *fasB* or *fasC*. WT, wild-type allele.

Two transcriptional regulatory systems (FasBCAX and CovRS) are known positive regulators of *ska* expression in GAS and SDSE (21, 66, 67). Thus, we inspected the coding sequences and upstream presumed regulatory regions of these two gene systems for polymorphisms potentially influencing transcript levels. The *covRS* region sequences were identical in the two SDSE isolates. In contrast, MGCS36089 had a 40 nt internal deletion in *fasB*, encoding a histidine kinase, resulting in FasB truncation (Fig. 6A). This truncation likely contributes to the decreased *ska* transcript levels and decreased mouse virulence observed for MGCS36089 compared to MGCS36044 (Fig. 6A). To further investigate this possibility, we examined *ska* transcript levels in three additional *stG62647* clinical isolates from French Brittany with mutations in either *fasB* or *fasC* (*fasC* encodes a second FasBCAX histidine kinase) (66) and compared them to four *stG62647* clinical isolates from the same region without mutations in these genes. Isolates in both groups lacked additional mutations in other *vir* or MTR genes (59) (Fig. 6B). Compared to isolates lacking *fas* gene mutations, isolates containing *fas* gene mutations all had decreased levels of *fasX* and *ska* transcripts and reduced mouse virulence (59) (Table S8). Taken together, the data support the interpretation that decreased *ska* gene transcript levels in these clonally related *stG62647* SDSE isolates contribute to reduced virulence in a mouse model of necrotizing myositis. However, additional experiments are required to formally test this hypothesis.

## DISCUSSION

Integrative analysis of genome polymorphisms, *in vitro* and *in vivo* transcript data, and primate virulence data has yielded new insights into GAS molecular pathogenesis (54). The primary goal of our study was to determine if an analogous experimental approach using two clonally related SDSE *emm stG62647* human isolates would provide useful leads for subsequent molecular pathogenesis and therapeutic studies. We used a validated model of streptococcal necrotizing myositis (31, 42, 48, 54) to analyze the *in vivo* SDSE transcriptome. Two isolates of *emm stG62647* were selected, as clonally related isolates of this *emm* type are prominent causes of invasive and other human infections and have been increasingly reported globally (2–8, 10, 60). Thus, obtaining new molecular pathogenesis data on these emerging isolates is particularly important.

Primate infection models are valuable due to their close phylogenetic relationship with humans. Although recent progress has been made with experimental GAS infections in the human upper respiratory tract (68, 69), analogous *in vivo* studies for serious SDSE infections are not feasible. Our study also allowed for comparison of SDSE data with previous findings from the *emm1* GAS strain MGAS2221 (54). SDSE is genetically closely related to GAS and causes many of the same types of human infections, including necrotizing myositis (70, 71). Thus, we anticipated some overlap in transcript data between the two species, as confirmed in our results.

We initially focused on *in vivo* transcript data from the NHP infected with MGCS36044, the isolate providing the highest number of SDSE genes with assigned normalized counts. Importantly, SDSE transcript data from five additional NHPs infected with MGCS36044 and six infected with MGCS36089 were similar, especially for genes with high transcript abundance (Fig. 4; Tables S4A and B, S5A and B, and S6A and B). The concordance of these data likely reflects our standardized necrotizing myositis infection protocol (17, 42, 54, 59) and the close clonal relationship between MGCS36044 and MGCS36089. The differences we observed in gene transcript data between primate biopsies were primarily due to variation in RNAseq read numbers obtained from each biopsy, likely resulting from technical variability due to limited tissue sample sizes. Nonetheless, the rank order of genes with high transcript magnitude was closely similar across NHPs.

### Comparison of findings for NHPs infected with SDSE and GAS *emm1* MGAS2221

We previously analyzed gene transcripts from three NHPs infected with *emm1* MGAS2221, a strain whose genome represents organisms responsible for a relatively recent pandemic (23, 26, 31, 54). Although additional *emm1* genomic variants have since emerged and spread intercontinentally (27, 30, 72–76), MGAS2221 remains a so-called “global” *emm1* genotype. The *in vivo* transcriptome data obtained from NHPs infected with MGAS2221 were instrumental in identifying five genes (*ihk-irr*, *ciaH*, *isp*, *slr*, and *dahA*) as contributors to the molecular pathogenesis of necrotizing myositis (54). These findings not only identified new virulence mechanisms but also provided a strong foundation for subsequent translational research and motivation for the present SDSE study. We did not generate isogenic SDSE mutant strains to directly confirm their role in molecular pathogenesis for two reasons: (i) subsequent NHP experiments would be prohibitively expensive; and (ii) the very close concordance in gene transcript profiles between GAS and SDSE infections makes it difficult to justify the cost and ethical consideration of additional NHP analyses using isogenic SDSE mutant strains. Thus, we tentatively conclude that the SDSE genes with high transcript abundance (listed in Fig. 4, Table 3, and Table S4 through 7) likely contribute to the molecular pathogenesis of necrotizing myositis. We believe that one or more of the identified SDSE genes may hold promise as targets in future translational research studies.

Comparisons between NHP infections with SDSE and *emm1* GAS strain MGAS2221 (54) revealed similarities in the *in vivo* expression patterns of *sag*, *ihk-irr*, *ciaHR*, *covR*, and *emm* in both SDSE strains compared to GAS (Tables S4A and B and S5A and B). Overall, the expression profiles of *vir* and MTR genes were similar in SDSE and GAS, consistent with their genetic relatedness (70, 71). However, some differences were observed. For example, despite their close genomic similarity, we identified genetic polymorphisms between strains MGCS36044 and MGCS36089 that may contribute to the virulence difference observed in a mouse model of necrotizing myositis (59, 60). Two lines of evidence support the hypothesis that a 40 nt deletion in *fasB* present in MGCS36089, which results in a truncated FasB histidine kinase, is responsible for the decreased *ska* transcript abundance observed in MGCS36089 compared to MGCS36044 (Table 2; Tables S2A and D, and S4A and B). First, the *fasBCAX* operon has been reported to upregulate *ska* expression in both GAS and SDSE (66, 67), suggesting that mutations that deleteriously affect *fasB* are likely to decrease *ska* transcript abundance. Second, *stG62647* clinical isolates with mutations in either *fasB* or *fasC* exhibited lower *ska* and *fasX* transcript abundance (Fig. 6 and Table S8) compared to strains lacking mutations in the *fasBCAX* operon or other MTR genes.

### Importance of advancing understanding of SDSE molecular pathogenesis

Given that SDSE and GAS are phylogenetically closely related, it is reasonable to hypothesize that some of the molecular processes driving SDSE pathogenesis resemble those involved in GAS-host interactions. As we have shown here, there are similarities in gene transcript patterns during necrotizing myositis caused by SDSE and GAS. However, despite sharing many proven or putative virulence factors, important differences remain. For example, SDSE lacks genes encoding many well-characterized GAS virulence factors, including streptococcal pyrogenic exotoxins (e.g., SpeA and SpeC), the potent extracellular cysteine protease, SpeB, and the streptococcal inhibitor of complement, SIC. Conversely, we identified MTR genes in SDSE not present in GAS, such as the putative ECF sigma factor/anti-sigma factor gene pair. In addition, the genome size of SDSE isolates is approximately 2.1 to 2.2 Mbp (6, 60, 77), which is somewhat larger than the GAS genome (~1.8 Mbp), suggesting that the increased genetic information present in SDSE isolates may contribute to processes important for pathogen-host interactions.

Epidemiologic and clinical studies indicate that, compared to patients with invasive GAS infections, those with invasive infections caused by SDSE are older and have more underlying medical problems, such as type 2 diabetes, cardiovascular disease, and cancer (2, 58, 78–81). Populations in nearly all developed countries are aging rapidly. In the United States alone, approximately 10,000 people reach the age of 65 each day. These demographic trends suggest that SDSE infections will likely continue to increase, further underscoring the need to better understand the molecular mechanisms underlying SDSE pathogenesis, especially those that may lead to the development of novel therapeutics or vaccines.

### Vaccine and therapeutics consideration

There are currently no licensed vaccines against SDSE and GAS, two human pathogens responsible for considerable morbidity and mortality globally. In the case of GAS, many vaccine candidates have been identified over more than a century of investigation (82). However, both pathogens share many of the same challenges in formulating an efficacious vaccine. First, there are no well-defined human correlates of protective immunity for either organism (83–85), a limitation that has long hindered vaccine research. While this topic has been studied extensively for GAS, relatively less work has been focused on SDSE. Second, natural populations of both SDSE and GAS exhibit extensive genetic diversity, including variability in gene content and allelic variation among shared genes (3, 12, 17, 31, 59, 60, 86–88). Third, both SDSE and GAS can cause a range of infections affecting diverse anatomic sites, including (but not limited to) the upper respiratory tract, blood, skin, and soft tissues. For GAS, studies have shown extensive variation in transcriptomes and virulence factors depending on the site of infection (53, 55, 89–91). In contrast, these dynamics are far less understood for SDSE largely due to limited investigations into its molecular pathogenesis. Infection-specific transcriptomes can impede the development of a successful vaccine, as virulence factors contributing to throat infection, for example, may be irrelevant or less important in infections of the lower respiratory tract, bloodstream, soft tissues, or vaginal tract (54, 55, 89, 91–94). Fourth, for GAS, even modest changes in the genome, such as single nucleotide polymorphisms in transcriptional regulators (24, 95) or variation in the length of homopolymeric tracts (15, 17), can substantially alter the transcriptome and virulence factor production. Though similar processes may operate in SDSE (59), they remain poorly characterized. Fifth, although our data strongly suggest that SDSE and GAS rely on some analogous virulence genes and circuits in necrotizing myositis, less is known about other genes contributing to molecular pathogenesis. Taken together, these and other challenges (e.g., the very rapid global spread of successful new variant lineages, intrahost variation, and variation in antigen expression) underscore the complexity of developing an efficacious vaccine for either pathogen. It is our opinion that the path forward is likely to remain long, arduous, expensive, and fraught with difficulties.

## Data Availability

Transcriptome data have been submitted to the National Center for Biotechnology Information (NCBI) under Bioproject accession number GSE296749.

## SUPPLEMENTAL MATERIAL

Supplemental Tables mbio.01349-25-s0001.pdf Tables S1 to S8.

Supplemental Figures mbio.01349-25-s0002.pdf Figures S1 to S3.

ASM does not own the copyrights to Supplemental Material that may be linked to, or accessed through, an article. The authors have granted ASM a non-exclusive, world-wide license to publish the Supplemental Material files. Please contact the corresponding author directly for reuse.
