# Supplementary material for: Analysis of Streptococcus dysgalactiae subspecies equisimilis gene transcripts during experimental primate necrotizing myositis
Source: mBio. 2025 Jul 22;16(8):e01349-25. doi: 10.1128/mbio.01349-25 (PMC12345147; doi:10.1128/mbio.01349-25)
Supplement: Supplemental Tables — Tables S1 to S8. [file mbio.01349-25-s0001.pdf]

Table S1. Number of sequencing reads and corresponding number of MGCS36044 and MGCS36089 genes identified during *in vivo* infections of eight NHPs

	Number of reads mapping to SDSE				SDSE genome coverage			
	Original sequencing runs(1)		Final number of reads(2)		Number of genes(3)	Genome percentage(4)	Number of genes	Genome percentage
**NHP ID**	**MGCS36044**	**MGCS36089**	**MGCS36044**	**MGCS36089**	**MGCS36044**	**MGCS36044**	**MGCS36089**	**MGCS36089**
**9**	10,244	26,779	131,039	271,844	**552**	28	**1,255**	63
**10**	1,801	16,798	N/A	229,961	N/A	N/A	**854**	43
**11**	11,447	1,689	199,700	N/A	**761**	38	N/A	N/A
**12**	5,791	13,292	N/A	166,123	N/A	N/A	**575**	29
**13**	10,848	29,135	189,346	222,140	**1,103**	55	**1,232**	62
**14**	9,143	72,094	180,108	664,029	**994**	50	**1,630**	81
**15**	84,738	19,460	1,026,473	215,966	**1,662**	83	**1,163**	58
**16**	7,600	5,868	90,087	N/A	**645**	32	N/A	N/A

(1) 80 cDNA corresponding to 8 NHPs/5 biopsy layers/2 SDSE strains
(2) Combination of all sequencing reads for 6 NHPs, after 2 NHPs were triaged per SDSE strain because an original number of reads ≤ 7,000
(3) Number of genes with mapped reads
(4) Corresponding genomic percentage based on the number of genes with mapped reads

Table S2A. Ranked MGCS36044 genes during growth *in vitro* at mid-exponential phase

No.	Locus tag	SignalP6 predicted[1]	Virulence[2]	Gene	Function	RPKMs[3]	RANK[4]
1	MGCS36044_03808		Virulence	*gapA*	glyceraldehyde-3-phosphate dehydrogenase GapA	17642.5	1
2	MGCS36044_01354			*tufA*	translation elongation factor Tu protein TufA	16186.8	2
3	MGCS36044_00222			*rplQ*	50S ribosomal L17 protein RplQ	11148.0	3
4	MGCS36044_01804			*rplL*	50S ribosomal L7/L12 protein RplL	10999.3	4
5	MGCS36044_00224			-	L17DE RNA	10864.0	5
6	MGCS36044_01806			-	rli38 RNA	10469.3	6
7	MGCS36044_01802			*rplJ*	50S ribosomal L10 protein RplJ	10392.0	7
8	MGCS36044_00220			*rpoA*	DNA-directed RNA polymerase subunit alpha RpoA	10376.5	8
9	MGCS36044_00194			*rpsZ*	type Z 30S ribosomal S14 protein RpsZ	10225.5	9
10	MGCS36044_00218			*rpsK*	30S ribosomal S11 protein RpsK	10110.3	10
11	MGCS36044_03334			-	RNaseP_bact_b RNA	9608.5	11
12	MGCS36044_00214			*rpmJ*	50S ribosomal L36 protein RpmJ	8885.8	12
13	MGCS36044_00192			*rplE*	50S ribosomal L5 protein RplE	8828.8	13
14	MGCS36044_01508		Virulence	*srrG*	streptolysin S small regulatory RNA SrrG	8556.8	14
15	MGCS36044_03656			*rpsF*	30S ribosomal S6 protein RpsF	8453.0	15
16	MGCS36044_00186			*rpsQ*	30S ribosomal S17 protein RpsQ	8438.5	16
17	MGCS36044_01506			*eno*	phosphopyruvate hydratase -- enolase protein	8293.5	17
18	MGCS36044_03654			*ssb_2*	single-stranded DNA-binding protein	8280.5	18
19	MGCS36044_00178			*rplV*	50S ribosomal L22 protein RplV	8223.5	19
20	MGCS36044_02888			*ptsH*	PTS transporter phosphocarrier protein PtsH	8049.8	20
21	MGCS36044_00216			*rpsM*	30S ribosomal S13 protein RpsM	8013.8	21
22	MGCS36044_00174			*rplB*	50S ribosomal L2 protein RplB	7932.3	22
23	MGCS36044_00188			*rplN*	50S ribosomal L14 protein RplN	7875.0	23
24	MGCS36044_00180			*rpsC*	30S ribosomal S3 protein RpsC	7854.0	24
25	MGCS36044_00182			*rplP*	50S ribosomal L29 protein RplP	7853.3	25
26	MGCS36044_00758			*fba_2*	fructose-bisphosphate aldolase	7839.0	26
27	MGCS36044_00190			*rplX*	50S ribosomal L24 protein RplX	7809.0	27
28	MGCS36044_03814			*rpsL*	30S ribosomal S12 protein RpsL	7757.3	28
29	MGCS36044_00172			*rplW*	50S ribosomal L23 protein RplW	7691.8	29
30	MGCS36044_00198			*rplF*	50S ribosomal L6 protein RplF	7518.8	30
31	MGCS36044_00170			*rplD*	50S ribosomal L4 protein RplD	7478.5	31
32	MGCS36044_00176			*rpsS*	30S ribosomal S19 protein RpsS	7273.3	32
33	MGCS36044_03812			*rpsG*	30S ribosomal S7 protein RpsG	7193.8	33
34	MGCS36044_01080			*rplA*	50S ribosomal L1 protein RplA	7138.8	34
35	MGCS36044_00204			*rpmD*	50S ribosomal L30 protein RpmD	7115.0	35
36	MGCS36044_01680			*prp*	ribosomal-processing cysteine protease Prp	7023.3	36
37	MGCS36044_01490			*rplS*	50S ribosomal L19 protein RpsL	7022.0	37
38	MGCS36044_00168			*rplC*	50S ribosomal L3 protein RplC	6824.5	38
39	MGCS36044_00606			*rpsI*	30S ribosomal S9 protein RpsI	6797.3	39
40	MGCS36044_02392			*rpsA*	30S ribosomal S1 protein RpsA	6526.8	40
41	MGCS36044_00208			*secY*	preprotein translocase subunit SecY	6399.8	41

No.	Locus tag	SignalP6 predicted[(1)]	Virulence[(2)]	Gene	Function	RPKMs[(3)]	RANK[(4)]
42	MGCS36044_00196			*rpsH*	30S ribosomal S8 protein RpsH	6317.8	42
43	MGCS36044_01716			-	KH domain-containing protein	6247.3	43
44	MGCS36044_00604			*rplM*	50S ribosomal L13 protein RplM	6105.8	44
45	MGCS36044_01470			*rpmE*	50S ribosomal L31 type B protein RpmE	6074.3	45
46	MGCS36044_01512		Virulence	-	sagA RNA	6051.0	46
47	MGCS36044_00200			*rplR*	50S ribosomal L18 protein RplR	5990.0	47
48	MGCS36044_03810			*fusA*	FusA family elongation factor EF-G	5910.0	48
49	MGCS36044_02994			-	DNA-binding protein HU	5848.3	49
50	MGCS36044_04060			*rpsB*	30S ribosomal S2 protein RpsB	5838.3	50
51	MGCS36044_00184			*rpmC*	50S ribosomal L16 protein RpmC	5684.5	51
52	MGCS36044_02384			*ssrA*	transfer-messenger RNA	5628.0	52
53	MGCS36044_01078			*rplK*	50S ribosomal L11P protein RplK	5627.3	53
54	MGCS36044_04202			*rpsD*	30S ribosomal S4 protein RpsD	5564.8	54
55	MGCS36044_01650			*rplT*	50S ribosomal L20 protein RplT	5473.3	55
56	MGCS36044_04144			*rpmGA*	50S ribosomal L33 protein RpmGA	5262.5	56
57	MGCS36044_00202			*rpsE*	30S ribosomal S5 protein RpsE	5070.3	57
58	MGCS36044_03652			*rpsR*	30S ribosomal S18 protein RpsR	5058.8	58
59	MGCS36044_00104	Secreted		*sibA*	CHAP domain-containing protein/secreted	4946.0	59
60	MGCS36044_00564			*rpsO*	30S ribosomal S15 protein RpsO	4583.5	60
61	MGCS36044_01510		Virulence	*sagA*	streptolysin S precursor SagA	4498.8	61
62	MGCS36044_03448			*srtB*	pilus polymerization class B sortase SrtB	4453.0	62
63	MGCS36044_02370			*pyk*	pyruvate kinase Pyk	4384.3	63
64	MGCS36044_02024			*ldh*	L-lactate dehydrogenase Ldh	4311.3	64
65	MGCS36044_00166			*rpsJ*	30S ribosomal S10 protein RpsJ	4270.5	65
66	MGCS36044_01648			*rpmI*	50S ribosomal L35 protein RpmL	4139.3	66
67	MGCS36044_02840	Secreted	Virulence	*spg*	extracellular cell surface IgG-binding	4110.3	67
68	MGCS36044_03446	Secreted		-	pilus ancillary/minor protein 2	4010.8	68
69	MGCS36044_04062			*tsf*	translation elongation factor Tsf	4008.5	69
70	MGCS36044_03536			*fabT*	transcriptional regulatory protein FabT	3989.0	70
71	MGCS36044_02886			*ptsI*	phosphoenolpyruvate--protein phosphotransferase	3957.8	71
72	MGCS36044_00212			*infA*	translation initiation factor IF-1 protein InfA	3947.8	72
73	MGCS36044_01564			*atpC*	ATP synthase epsilon subunit AtpC	3771.5	73
74	MGCS36044_01682			*rpmA*	50S ribosomal L27 protein RpmA	3733.0	74
75	MGCS36044_02980			*gpmA*	phosphoglycerate mutase GpmA	3660.5	75
76	MGCS36044_03452			*sipA*	signal peptidase I SipA	3550.3	76
77	MGCS36044_00206			*rplO*	50S ribosomal L15 protein RplO	3468.3	77
78	MGCS36044_01562			*atpD*	ATP synthase beta subunit AtpD	3412.3	78
79	MGCS36044_03866		Virulence	*fasX*	FasBCAX signal transduction system small RNA	3374.5	79
80	MGCS36044_02288			*rpsT*	30S ribosomal S20 protein RpsT	3374.3	80
81	MGCS36044_03450	Secreted		-	pilus backbone/major protein	3372.5	81
82	MGCS36044_01646			*infC*	translation initiation factor InfC	3332.0	82
83	MGCS36044_01558			*atpA*	ATP synthase alpha chain, AtpA	3232.8	83
84	MGCS36044_03506			*manN*	PTS transporter mannose-specific IID component	3215.5	84

No.	Locus tag	SignalP6 predicted[1]	Virulence[2]	Gene	Function	RPKMs[3]	RANK[4]
85	MGCS36044_03538			*phaB*	enoyl-CoA hydratase protein PhaB	3191.3	85
86	MGCS36044_01554			*atpF*	ATP synthase B subunit AtpF	3148.0	86
87	MGCS36044_02372			*pfkA*	6-phosphofructokinase PfkA	3087.8	87
88	MGCS36044_01556			*atpH*	ATP synthase delta subunit AtpH	3083.3	88
89	MGCS36044_01560			*atpG*	ATP synthase gamma subunit AtpG	3059.5	89
90	MGCS36044_03534			*fabH*	3-oxoacyl-[acyl-carrier-protein] synthase protein FabH	3058.8	90
91	MGCS36044_03454	Secreted		-	pilus ancillary/minor protein 1	3001.5	91
92	MGCS36044_00740			*tig*	trigger factor molecular chaperone Tig	2894.5	92
93	MGCS36044_01714			*rpsP*	30S ribosomal S16 protein RpsP	2878.5	93
94	MGCS36044_01678			*rplU*	50S ribosomal L21 protein RplU	2806.0	94
95	MGCS36044_01552			*atpB*	ATP synthase A subunit AtpB	2782.5	95
96	MGCS36044_03804			*pgk*	phosphoglycerate kinase Pgk	2755.8	96
97	MGCS36044_03858			*rpmH*	50S ribosomal L34 protein RpmH	2642.5	97
98	MGCS36044_03502			*manL*	PTS transporter mannose-specific IIB & IIA	2578.5	98
99	MGCS36044_00364	Secreted		-	secreted pilin backbone/major protein	2535.0	99
100	MGCS36044_03504			*manM*	PTS transporter mannose-specific IIC component	2529.5	100
101	MGCS36044_04104			*spxA_2*	transcriptional regulator SpxA	2513.8	101
102	MGCS36044_00482			*ssrS*	6S RNA	2422.0	102
103	MGCS36044_01364			*tpiA*	triose-phosphate isomerase TpiA	2370.0	103
104	MGCS36044_01066	Lipo[5]		*mtsA*	metal ABC transporter substrate-binding	2217.8	104
105	MGCS36044_02908	Lipo		*prsA*	peptidylprolyl isomerase lipoprotein PrsA	2200.0	105
106	MGCS36044_03616	Secreted	Virulence	*isp2*	Isp-related CHAP domain-containing immunogenic	2174.8	106
107	MGCS36044_02670			*dltC*	D-alanine--poly(phosphoribitol) ligase subunit	2141.5	107
108	MGCS36044_03532			*acpP_2*	acyl carrier protein AcpP	2131.5	108
109	MGCS36044_04038			*ahpF*	alkyl hydroperoxide reductase F subunit AhpF	2124.8	109
110	MGCS36044_00516	Secreted	Virulence	*emm*	cell surface M protein Emm	2061.3	110
111	MGCS36044_01728			*apbA*	2-dehydropantoate 2-reductase	2054.5	111
112	MGCS36044_03048			-	RNA-binding protein	2047.8	112
113	MGCS36044_03518			*accC*	acetyl-CoA carboxylase biotin carboxylase	2046.5	113
114	MGCS36044_02672			*dltB*	D-alanyl-lipoteichoic acid biosynthesis protein DltB	2039.5	114
115	MGCS36044_00316			*rpoC*	DNA-directed RNA polymerase subunit beta' RpoC	2003.0	115
116	MGCS36044_03522			*accB*	acetyl-CoA carboxylase biotin carboxyl carrier	1981.3	116
117	MGCS36044_01598			*rpsU*	30S ribosomal S21 protein RpsU	1969.3	117
118	MGCS36044_03520			*fabZ*	3-hydroxyacyl-ACP dehydratase FabZ	1870.8	118
119	MGCS36044_02020			*fadH2*	FadH2 superfamily FAD-dependent oxidoreductase	1868.3	119
120	MGCS36044_02344			*yeaQ*	GlsB/YeaQ/YmgE family stress response membrane	1850.0	120
121	MGCS36044_03704			*glpF_2*	glycerol uptake facilitator GlpF	1832.3	121
122	MGCS36044_03050			-	YggT family protein	1810.5	122
123	MGCS36044_03516			*accA*	acetyl-CoA carboxylase, carboxyltransferase beta	1798.8	123
124	MGCS36044_02668			*dltD*	D-alanyl-lipoteichoic acid biosynthesis protein	1788.5	124
125	MGCS36044_03046			*divIVA*	cell division protein DivIVA	1779.8	125
126	MGCS36044_03054			*yggS*	YggS family pyridoxal phosphate-dependent	1740.8	126
127	MGCS36044_03514			*accD*	acetyl-CoA carboxylase carboxyl transferase	1735.8	127

No.	Locus tag	SignalP6 predicted[(1)]	Virulence[(2)]	Gene	Function	RPKMs[(3)]	RANK[(4)]
128	MGCS36044_02674			*dltA*	D-alanine--poly(phosphoribitol) ligase subunit DltA	1731.0	128
129	MGCS36044_03638			-	Asp23/Gls24 family envelope stress response protein	1724.0	129
130	MGCS36044_01528		Virulence	*sagI*	streptolysin S export permease protein SagI	1719.5	130
131	MGCS36044_03682			*pflB*	formate C-acetyltransferase	1716.8	131
132	MGCS36044_03920			*pgi*	Pgi family glucose-6-phosphate isomerase	1707.0	132
133	MGCS36044_02340			-	DUF2273 domain-containing protein	1700.3	133
134	MGCS36044_03078			*dps*	DNA protection during starvation protein	1667.8	134
135	MGCS36044_03640			*efp*	translation elongation factor (P) Efp	1659.0	135
136	MGCS36044_01514		Virulence	*sagB*	streptolysin S biosynthesis protein SagB	1649.3	136
137	MGCS36044_02334			-	Asp23/Gls24 family envelope stress response protein	1623.8	137
138	MGCS36044_02338			-	Asp23/Gls24 family envelope stress response protein	1617.8	138
139	MGCS36044_02342			*amaP*	alkaline shock response membrane anchor protein	1605.8	139
140	MGCS36044_02884			*gapN*	NADP-dependent glyceraldehyde-3-phosphate	1563.3	140
141	MGCS36044_01526		Virulence	*sagH*	streptolysin S export permease protein SagH	1559.3	141
142	MGCS36044_03056			*ftsZ*	cell division protein FtsZ	1557.3	142
143	MGCS36044_03524			*fabF*	3-oxoacyl-[acyl-carrier-protein] synthase	1544.5	143
144	MGCS36044_03528			*fabD*	malonyl CoA-acyl carrier protein transacylase	1534.5	144
145	MGCS36044_03052			*sepF*	cell division protein SepF	1504.8	145
146	MGCS36044_01520		Virulence	*sagE*	streptolysin S self-immunity protein SagE	1486.5	146
147	MGCS36044_01522		Virulence	*sagF*	streptolysin S biosynthesis protein SagF	1480.5	147
148	MGCS36044_02336			-	CsbD family protein	1464.5	148
149	MGCS36044_03526			*fabG_2*	3-ketoacyl-(acyl-carrier-protein) reductase	1454.5	149
150	MGCS36044_04036			*ahpC*	alkyl hydroperoxide reductase C subunit AhpC	1434.5	150
151	MGCS36044_03636			*nusB*	transcription termination protein NusB	1433.5	151
152	MGCS36044_01516		Virulence	*sagC*	streptolysin S biosynthesis protein SagC	1431.3	152
153	MGCS36044_03544			*dnaK*	molecular chaperone DnaK	1427.0	153
154	MGCS36044_01524		Virulence	*sagG*	streptolysin S export protein SagG	1426.0	154
155	MGCS36044_00766			-	DAK2 domain-containing protein	1423.8	155
156	MGCS36044_03344			*pepC*	aminopeptidase (A) PepC	1422.5	156
157	MGCS36044_03336			*gpsB*	cell division regulator GpsB	1415.0	157
158	MGCS36044_00314			*rpoB*	DNA-directed RNA polymerase subunit beta RpoB	1392.3	158
159	MGCS36044_03816	Secreted		*prgA*	surface exclusion domain-containing secreted	1374.5	159
160	MGCS36044_03314			*rny*	ribonuclease (Y) Rny	1361.3	160
161	MGCS36044_03464			*infB*	translation initiation factor IF-2	1343.3	161
162	MGCS36044_04142			*rpmF*	50S ribosomal L32 protein RpmF	1337.3	162
163	MGCS36044_01518		Virulence	*sagD*	streptolysin S biosynthesis protein SagD	1320.8	163
164	MGCS36044_04224			*mnmA*	tRNA 2-thiouridine(34) synthase MnmA	1317.8	164
165	MGCS36044_01726			-	PTS transporter subunit IIC	1317.3	165
166	MGCS36044_00874			*htpX*	zinc metalloprotease HtpX	1314.3	166
167	MGCS36044_03070			*typA*	translational GTPase TypA	1288.0	167
168	MGCS36044_00878		Virulence	*covR*	TCS[(6)] DNA-binding response regulator CovR	1287.8	168
169	MGCS36044_01094	Secreted		*lysM*	LysM peptidoglycan-binding domain-containing	1274.0	169
170	MGCS36044_04232			-	transglycosylase SLT domain-containing protein	1271.8	170

No.	Locus tag	SignalP6 predicted[(1)]	Virulence[(2)]	Gene	Function	RPKMs[(3)]	RANK[(4)]
171	MGCS36044_03530			*fabK*	Enoyl-[acyl-carrier-protein] reductase protein	1258.0	171
172	MGCS36044_04222			*marC*	MarC family small neutral amino acid	1253.5	172
173	MGCS36044_03622			*secA*	preprotein translocase subunit SecA	1252.3	173
174	MGCS36044_01808			-	hypothetical protein	1242.5	174
175	MGCS36044_00872			*lemA*	LemA family protein	1232.8	175
176	MGCS36044_00030			*ftsH*	ATP-dependent zinc metalloprotease FtsH	1223.3	176
177	MGCS36044_03798			*glnA*	glutamine synthetase GlnA	1222.0	177
178	MGCS36044_04254			*guaB*	IMP dehydrogenase GuaB	1216.8	178
179	MGCS36044_03806	Lipo		*lppC*	e(P4) family 5'-nucleotidase lipoprotein	1213.0	179
180	MGCS36044_04098			*ruvX*	Holliday junction resolvase RuvX	1206.5	180
181	MGCS36044_00966			*upp*	uracil phosphoribosyltransferase Upp	1203.5	181
182	MGCS36044_02952			*deaD*	DEAD/DEAH box helicase	1203.0	182
183	MGCS36044_04274		Virulence	*htrA*	trypsin-like serine protease HtrA	1195.5	183
184	MGCS36044_00106			*prs*	ribose-phosphate pyrophosphokinase PrsA	1178.5	184
185	MGCS36044_02598			*rfbC*	dTDP-4-dehydrorhamnose 3,5-epimerase RfbC	1175.8	185
186	MGCS36044_03800			*glnR*	glutamine synthetase transcriptional repressor	1173.5	186
187	MGCS36044_03300			*pknB*	Stk1 family PASTA domain-containing Ser/Thr	1159.3	187
188	MGCS36044_02596			*rfbB*	dTDP-glucose 4,6-dehydratase RfbB	1127.5	188
189	MGCS36044_03310			*rpoZ*	DNA-directed RNA polymerase omega subunit RpoZ	1126.0	189
190	MGCS36044_01600			*mscL*	large-conductance mechanosensitive channel	1121.0	190
191	MGCS36044_02600			*rfbA*	glucose-1-phosphate thymidylyltransferase RfbA	1083.8	191
192	MGCS36044_02438			*xapA*	XapA family purine-nucleoside phosphorylase	1072.8	192
193	MGCS36044_03414			*degV_2*	DegV family fatty acid-binding protein	1067.8	193
194	MGCS36044_02528			*glmM*	phosphoglucosamine mutase GlmM	1060.8	194
195	MGCS36044_03288			*cysK*	cysteine synthase A CysK	1060.5	195
196	MGCS36044_00910			*yccA*	YccA family protein	1050.8	196
197	MGCS36044_01430			*ftsX*	cell division permease-like protein FtsX	1049.0	197
198	MGCS36044_01320			*lysS*	lysyl-tRNA synthetase LysS	1047.0	198
199	MGCS36044_04230			-	HAD hydrolase-like protein	1045.3	199
200	MGCS36044_02440			*arsC_2*	arsenate reductase ArsC	1045.0	200
201	MGCS36044_02676			*dltX*	teichoic acid D-Ala incorporation-associated	1043.8	201
202	MGCS36044_01086			*frr*	ribosome recycling factor Frr	1039.0	202
203	MGCS36044_04068			*treB*	PTS transporter trehalose-specific EIIBC	1038.8	203
204	MGCS36044_03312			*gmk*	guanylate kinase Gmk	1028.8	204
205	MGCS36044_01734			*fruA*	fructose-specific PTS transporter EIIC component	1026.5	205
206	MGCS36044_00210			*adk*	adenylate kinase protein Adk	1024.5	206
207	MGCS36044_02614			*apt*	adenine phosphoribosyltransferase Apt	1023.8	207
208	MGCS36044_03560			*gatB_2*	aspartyl-tRNA(Asn) or glutamyl-tRNA(Gln)	1023.8	207
209	MGCS36044_02278			*cdd*	cytidine deaminase Cdd	1015.5	209
210	MGCS36044_02036	Secreted		-	DUF1002 domain-containing putative secreted	1013.3	210
211	MGCS36044_02294			*pepN*	lysyl aminopeptidase/alanine aminopeptidase	1009.5	211
212	MGCS36044_02682			*glnQ_2*	glutamine transport ATP-binding protein GlnQ	991.8	212
213	MGCS36044_02364			*glmS*	glutamine--fructose-6-phosphate transaminase	988.0	213

No.	Locus tag	SignalP6 predicted[(1)]	Virulence[(2)]	Gene	Function	RPKMs[(3)]	RANK[(4)]
214	MGCS36044_02436			*deoD*	DeoD-type purine-nucleoside phosphorylase	975.8	214
215	MGCS36044_01550			*atpE*	ATP synthase C subunit AtpE	975.0	215
216	MGCS36044_00308			*tyrS*	tyrosyl-tRNA synthetase TyrS	971.5	216
217	MGCS36044_03662			*trxA_2*	thioredoxin TrxA	971.0	217
218	MGCS36044_02276	Lipo		-	putative nucleoside ABC transporter	965.0	218
219	MGCS36044_03058			*ftsA*	cell division protein FtsA	952.0	219
220	MGCS36044_02442			*deoB*	phosphopentomutase DeoB	947.8	220
221	MGCS36044_02270			-	putative nucleoside ABC transporter permease	942.0	221
222	MGCS36044_03940			*tgt*	tRNA guanosine(34) transglycosylase Tgt	936.8	222
223	MGCS36044_00798	Lipo		*oppA_1*	oligopeptide ABC transporter substrate-binding	936.3	223
224	MGCS36044_03874			*gltX*	glutamate--tRNA ligase	936.3	223
225	MGCS36044_03356			-	GlnQ family polar amino acid ABC transporter	933.5	225
226	MGCS36044_01500			*ezrA*	cell division septation ring formation regulator	918.0	226
227	MGCS36044_01710			-	TVP38 superfamily protein	915.5	227
228	MGCS36044_00366	Secreted		-	secreted pilin minor/ancillary protein	913.5	228
229	MGCS36044_03470			*nusA*	transcription termination factor NusA	904.3	229
230	MGCS36044_02128			*guaA*	glutamine-hydrolyzing GMP synthase	901.3	230
231	MGCS36044_03330			*mapZ*	MapZ family cell division site-positioning	897.8	231
232	MGCS36044_02272			-	putative nucleoside ABC transporter permease	887.0	232
233	MGCS36044_04228			*sdhA*	L-serine dehydratase alpha subunit SdhA	882.0	233
234	MGCS36044_03860			*jag*	RNA-binding protein Jag	880.8	234
235	MGCS36044_03562			*gatA_2*	aspartyl-tRNA(Asn) or glutamyl-tRNA(Gln) amidotransferase A subunit GatA	876.5	235
236	MGCS36044_03786			*rnjA_2*	mRNA degradation ribonuclease RnjA	875.5	236
237	MGCS36044_00804			*oppD_1*	oligopeptide ABC transporter permease protein	872.3	237
238	MGCS36044_00592			-	NYN domain-containing protein	866.3	238
239	MGCS36044_01428			*ftsE*	cell division ATP-binding protein FtsE	861.5	239
240	MGCS36044_00378		Virulence	*fbp*	secreted fibronectin-binding protein	861.3	240
241	MGCS36044_02296			*phoU_2*	phosphate signaling complex protein PhoU	857.3	241
242	MGCS36044_00902	Lipo		*yidC_1*	membrane protein insertase lipoprotein YidC	857.0	242
243	MGCS36044_04100			-	IreB-related regulatory phosphoprotein	852.5	243
244	MGCS36044_01298			-	PspC domain-containing protein	845.0	244
245	MGCS36044_02718			-	DUF1846 domain-containing protein	842.5	245
246	MGCS36044_00578			*cysE*	serine O-acetyltransferase CysE	838.0	246
247	MGCS36044_01660			-	YlbF/YmcA family competence regulator	837.0	247
248	MGCS36044_04260			*uup*	Uup family ATPase components of ABC transporters with duplicated ATPase domains	836.8	248
249	MGCS36044_02708			*pepS*	aminopeptidase PepS	835.3	249
250	MGCS36044_03382			*tkt*	transketolase Tkt	835.0	250
251	MGCS36044_00764			-	Asp23/Gls24 family envelope stress response	832.8	251
252	MGCS36044_01708			-	ABC transporter permease	832.0	252
253	MGCS36044_00950			*ppaC*	manganese-dependent inorganic pyrophosphatase	830.0	253
254	MGCS36044_00390			*purA*	adenylosuccinate synthase PurA	829.8	254
255	MGCS36044_00968			*clpP*	ATP-dependent Clp protease proteolytic subunit	825.8	255
256	MGCS36044_03466			-	YlxQ-related RNA-binding protein	824.0	256

No.	Locus tag	SignalP6 predicted[(1)]	Virulence[(2)]	Gene	Function	RPKMs[(3)]	RANK[(4)]
257	MGCS36044_00806			*oppF_1*	oligopeptide ABC transporter ATP-binding protein	823.0	257
258	MGCS36044_03290			-	S1 RNA-binding domain-containing protein	821.0	258
259	MGCS36044_01978			*eutD*	phosphate acetyltransferase EutD	820.8	259
260	MGCS36044_00590			*rmlB*	23S rRNA (guanosine(2251)-2'-O)-methyltransferase RlmB	820.5	260
261	MGCS36044_01438			*asnC*	asparaginyl-tRNA synthetase protein AsnC	819.5	261
262	MGCS36044_03900			*galU*	UTP--glucose-1-phosphate uridylyltransferase	818.8	262
263	MGCS36044_02298			*ptsB1*	phosphate ABC transporter ATP-binding protein PstB1	817.8	263
264	MGCS36044_00900			*greA*	transcription elongation factor GreA	816.3	264
265	MGCS36044_03302			*pppL*	Stp1/IreP family PP2C-type Ser/Thr phosphatase	809.8	265
266	MGCS36044_03478			*cotS*	CotS family thiamine kinase	804.8	266
267	MGCS36044_00368			*srtC_1*	class C sortase SrtC	804.5	267
268	MGCS36044_00376			*srtC_3*	class C sortase SrtC	802.0	268
269	MGCS36044_04096			-	DUF1292 domain-containing protein	796.0	269
270	MGCS36044_02216			-	apolipoprotein A1/A4/E family protein	795.5	270
271	MGCS36044_03462			*rbfA*	30S ribosome-binding factor RbfA	790.5	271
272	MGCS36044_00312			-	Lacto-rpoB	790.0	272
273	MGCS36044_03584			*alaT*	AlaT family aminotransferase	787.0	273
274	MGCS36044_00760			*rpmB*	50S ribosomal L28 protein RpmB	781.5	274
275	MGCS36044_03944		Virulence	*perR*	peroxide-responsive transcriptional repressor	780.0	275
276	MGCS36044_02544			*acoL*	dihydrolipoyl dehydrogenase AcoL	777.8	276
277	MGCS36044_02280			*deoC*	deoxyribose-phosphate aldolase DeoC	777.0	277
278	MGCS36044_03764			*ybaB*	YbaB family DNA-binding protein	777.0	277
279	MGCS36044_02314			*spxA_1*	transcriptional regulator SpxA	775.0	279
280	MGCS36044_02904			*alaS*	alanine--tRNA synthetase AlaS	774.0	280
281	MGCS36044_03974			*leuS*	leucine--tRNA synthase LeuS	772.3	281
282	MGCS36044_02936			*sodA*	superoxide dismutase SodA	770.3	282
283	MGCS36044_03476			*trmB*	tRNA (guanosine(46)-N7)-methyltransferase TrmB	756.3	283
284	MGCS36044_03468			-	YlxR family putative RNA-binding protein	739.5	284
285	MGCS36044_01794			*engB*	ribosome biogenesis GTP-binding protein EngB	737.5	285
286	MGCS36044_02988			*zntA*	ZntA family P-type heavy metal transporter	736.0	286
287	MGCS36044_01108			-	Nudix superfamily phosphohydrolase	734.8	287
288	MGCS36044_03546			*grpE*	heat shock protein/nucleotide exchange factor	725.5	288
289	MGCS36044_01308			-	DUF3270 domain-containing protein	720.3	289
290	MGCS36044_02274			-	putative nucleoside ABC transporter ATP-binding	717.8	290
291	MGCS36044_03540			*dnaJ*	chaperone protein DnaJ	717.3	291
292	MGCS36044_04090			*nrdD_2*	anaerobic ribonucleoside-triphosphate reductase NrdD	717.0	292
293	MGCS36044_01732			*fruK*	1-phosphofructokinase FruK	714.8	293
294	MGCS36044_02300			*ptsB2*	phosphate ABC transporter ATP-binding protein PstB2	713.3	294
295	MGCS36044_00576			-	polynucleotide phosphorylase/polyadenylase	712.5	295
296	MGCS36044_01084			*pyrH*	UMP kinase PyrH	706.5	296
297	MGCS36044_04128			*argS*	arginine--tRNA synthase ArgS	702.3	297
298	MGCS36044_03582			*codY*	CodY family GTP-sensing pleiotropic	694.5	298
299	MGCS36044_01706			-	LolD superfamily ABC transporter ATPase	692.0	299

No.	Locus tag	SignalP6 predicted[(1)]	Virulence[(2)]	Gene	Function	RPKMs[(3)]	RANK[(4)]
300	MGCS36044_00742			*rpoE*	DNA-directed RNA polymerase subunit delta RpoE	689.3	300
301	MGCS36044_01202			*thrS*	threonyl-tRNA synthetase ThrS	688.3	301
302	MGCS36044_00336			*ackA*	acetate kinase AckA	686.8	302
303	MGCS36044_00898			*mltG*	endolytic transglycosylase MltG	686.8	302
304	MGCS36044_04210			*rplI*	50S ribosomal L9 protein RplI	686.3	304
305	MGCS36044_02548			*acoC*	dihydrolipoamide acetyltransferase AcoC	683.5	305
306	MGCS36044_01068			*mtsB*	metal ABC transporter ATP-binding protein MtsB	680.8	306
307	MGCS36044_04108			*recA*	recombinase RecA	680.0	307
308	MGCS36044_03034			-	DUF1797 family protein	678.3	308
309	MGCS36044_02218			-	hypothetical protein	675.3	309
310	MGCS36044_04276			*parB*	chromosome partitioning protein ParB	674.8	310
311	MGCS36044_00370			*srtC_2*	class C sortase SrtC	671.0	311
312	MGCS36044_03358			*hisM*	HisM family amino acid ABC transporter permease	670.0	312
313	MGCS36044_00372	Secreted		-	truncated secreted pilin minor/ancillary protein	668.0	313
314	MGCS36044_02214			-	LCB5 family diacylglycerol lipid kinase	662.5	314
315	MGCS36044_02892			*nrdE_2*	class 1b ribonucleoside-diphosphate reductase alpha subunit NrdE	661.5	315
316	MGCS36044_03650			-	DUF1129 domain-containing protein	659.0	316
317	MGCS36044_00574			*pnp*	polyribonucleotide nucleotidyltransferase Pnp	657.5	317
318	MGCS36044_00848			-	YebC/PmpR family DNA-binding transcriptional	657.5	317
319	MGCS36044_02550			*acoB*	pyruvate dehydrogenase E1 component beta subunit	655.5	319
320	MGCS36044_03488			-	Cps2a family anionic cell wall polymer	650.5	320
321	MGCS36044_03342			*pbp1A*	bifunctional PG transglycosylase-transpeptidase	650.3	321
322	MGCS36044_01738	Lipo		-	putative peptidoglycan hydrolase lipoprotein	647.5	322
323	MGCS36044_01306			-	YtxH domain-containing protein	646.8	323
324	MGCS36044_03542			-	PfpI family predicted protease/amidase	644.3	324
325	MGCS36044_00028			-	hypoxanthine-guanine phosphoribosyltransferase	644.0	325
326	MGCS36044_01272			-	SPJ_0845 family protein	643.8	326
327	MGCS36044_03400			*glyS*	glycine--tRNA ligase beta subunit GlyS	643.5	327
328	MGCS36044_01200			-	glycosyltransferase	642.5	328
329	MGCS36044_00548			*proS*	prolyl-tRNA synthetase ProS	639.5	329
330	MGCS36044_01256			*ftsY*	signal recognition particle-docking protein	637.8	330
331	MGCS36044_03818			*purR*	pur operon repressor PurR	635.8	331
332	MGCS36044_04208			*dnaC*	replicative DNA helicase DnaC	634.3	332
333	MGCS36044_03044			*ileS*	isoleucine--tRNA synthetase IleS	633.3	333
334	MGCS36044_02448			*pepV*	dipeptidase PepV	631.8	334
335	MGCS36044_02882			-	PgdA-like putative PG GlcNAc deacetylase	631.0	335
336	MGCS36044_04238			*cbiO1*	cobalt ABC transporter ATPase CbiO2	629.8	336
337	MGCS36044_01372			*murN*	peptidoglycan lipid II-Ala--L-alanine ligase	625.5	337
338	MGCS36044_02444			*rpiA*	ribose-5-phosphate isomerase RpiA	624.5	338
339	MGCS36044_01374			*murM*	peptidoglycan lipid II--L-alanine ligase protein	623.8	339
340	MGCS36044_00540			*yajC*	preprotein translocase subunit YajC	622.5	340
341	MGCS36044_00830			*nadD*	nicotinate-nucleotide adenylyltransferase NadD	622.0	341
342	MGCS36044_00888			*der*	ribosome biogenesis GTPase Der	622.0	341

No.	Locus tag	SignalP6 predicted[(1)]	Virulence[(2)]	Gene	Function	RPKMs[(3)]	RANK[(4)]
343	MGCS36044_01376			-	sugar-phosphatase	621.5	343
344	MGCS36044_00782			*rgpG*	undecaprenyl/decaprenyl-phosphate	619.3	344
345	MGCS36044_01534			*queT*	Queuosine precursor transporter QueT	618.5	345
346	MGCS36044_00526	Secreted	Virulence	*ska*	secreted streptokinase Ska	617.3	346
347	MGCS36044_02894			*nrdF_2*	class 1b ribonucleoside-diphosphate reductase beta subunit NrdF	617.3	346
348	MGCS36044_02302			*ptsA*	phosphate ABC transporter permease PstA	616.8	348
349	MGCS36044_02986			-	Spy1186876 RNA	616.0	349
350	MGCS36044_02546			-	MGCS36044_02546	615.8	350
351	MGCS36044_04242			*rodZ*	cytoskeletal protein RodZ	615.3	351
352	MGCS36044_01744			*degV_1*	DegV family protein	613.5	352
353	MGCS36044_02612			*dnaD*	DNA replication protein DnaD	609.3	353
354	MGCS36044_01644			-	L20_leader RNA	608.8	354
355	MGCS36044_02906			-	LURP-one-related family protein	603.8	355
356	MGCS36044_01028			*metS*	methionine--tRNA synthase MetS	603.0	356
357	MGCS36044_01384			*mgtA*	MgtA superfamily cation-translocating P-type	601.8	357
358	MGCS36044_03548			*hrcA*	heat-inducible transcriptional repressor HrcA	601.8	357
359	MGCS36044_04064			*pepO*	endopeptidase PepO	598.5	359
360	MGCS36044_03512			*serS*	seryl-tRNA synthetase SerS	598.0	360
361	MGCS36044_01426			*prfB*	peptide chain release factor 2 PrfB	597.8	361
362	MGCS36044_02834			*map*	methionyl aminopeptidase Map	596.8	362
363	MGCS36044_02380			-	ABC transporter permease	596.3	363
364	MGCS36044_03472			*rimP*	ribosome maturation factor RimP	595.3	364
365	MGCS36044_02266			*panT*	pantothenic acid transporter PanT	595.0	365
366	MGCS36044_03292			-	putative bifunctional	593.5	366
367	MGCS36044_00802			*oppC_1*	oligopeptide ABC transporter permease protein	593.3	367
368	MGCS36044_02680	Lipo		*glnP_2*	glutamine-binding protein/glutamine transport	590.5	368
369	MGCS36044_03396	Lipo		-	PepSY domain-containing lipoprotein	589.8	369
370	MGCS36044_03362			*cshB*	DEAD/DEAH box helicase	588.0	370
371	MGCS36044_01604			*rpoD*	RNA polymerase sigma factor RpoD	587.3	371
372	MGCS36044_04240			*pgsA*	CDP-diacylglycerol--glycerol-3-phosphate	587.3	371
373	MGCS36044_00880		Virulence	*covS*	TCS sensor kinase CovS	584.5	373
374	MGCS36044_03862	Lipo		*yidC_2*	YidC/Oxa1 family membrane protein insertase	584.5	373
375	MGCS36044_03170			*valS*	valine--tRNA synthetase ValS	584.3	375
376	MGCS36044_03040			-	DUF1827 family protein	583.5	376
377	MGCS36044_03864			*rnpA*	ribonuclease P protein component RnpA	581.3	377
378	MGCS36044_01704			-	RND family transporter membrane fusion protein	576.5	378
379	MGCS36044_03370			*mraW*	S-adenosyl-methyltransferase MraW	575.5	379
380	MGCS36044_03252			-	DUF402 domain-containing protein	573.3	380
381	MGCS36044_02220			-	CsbD family protein	573.0	381
382	MGCS36044_01240		Virulence	*vicK*	TCS signal transduction sensor kinase VicK	572.5	382
383	MGCS36044_01070			*mtsC*	metal ABC transporter permease MtsC	571.5	383
384	MGCS36044_01568			*murA_1*	UDP-N-acetylglucosamine	570.5	384
385	MGCS36044_00834			-	cysteine hydrolase	569.8	385

No.	Locus tag	SignalP6 predicted[1]	Virulence[2]	Gene	Function	RPKMs[3]	RANK[4]
386	MGCS36044_01106			*era*	GTPase Era	569.5	386
387	MGCS36044_02962			*prfC*	peptide chain release factor 3 PrfC	569.3	387
388	MGCS36044_00004			*dnaN*	DNA polymerase III subunit beta protein DnaN	564.8	388
389	MGCS36044_01104			*dgkA*	diacylglycerol kinase DgkA	564.5	389
390	MGCS36044_04000			*nusG*	transcription antitermination protein NusG	564.3	390
391	MGCS36044_03352			*trxB_2*	thioredoxin-disulfide reductase TrxB	563.5	391
392	MGCS36044_03770			-	DUF536 domain-containing protein	561.8	392
393	MGCS36044_02536			-	CobQ-like type 1 glutamine amidotransferase	561.5	393
394	MGCS36044_02610			*nth*	endonuclease III Nth	555.8	394
395	MGCS36044_04022			*clpC*	ATP-dependent Clp protease ATP-binding subunit ClpC	552.8	395
396	MGCS36044_00908			*rnaY*	RnaY family HD domain-containing protein	552.0	396
397	MGCS36044_03346			*nadE*	ammonia-dependent NAD(+) synthetase NadE	550.5	397
398	MGCS36044_02304			*ptsC*	phosphate ABC transporter permease PstC	548.5	398
399	MGCS36044_04018			*groEL*	chaperonin GroEL	548.0	399
400	MGCS36044_02996			-	DUF2140 domain-containing protein	546.5	400
401	MGCS36044_00894			*murC*	UDP-N-acetylmuramate--L-alanine ligase MurC	545.8	401
402	MGCS36044_01296			-	SprT family protein	545.8	401
403	MGCS36044_00832			*yqeK*	bis(5'-nucleosyl)-tetraphosphatase (symmetrical)	545.5	403
404	MGCS36044_04106			-	hypothetical protein	545.3	404
405	MGCS36044_01966			*nifS_2*	NifS superfamily cysteine desulfurase	545.0	405
406	MGCS36044_03088			*rlmN*	23S rRNA (adenine(2503)-C(2))-methyltransferase	544.0	406
407	MGCS36044_01934			*potB*	spermidine putrescine ABC transport system permease protein PotB	542.5	407
408	MGCS36044_00556			-	NAD(P)H-dependent oxidoreductase	541.5	408
409	MGCS36044_01968			*ribP*	ribose-phosphate pyrophosphokinase RibP	541.3	409
410	MGCS36044_01312			-	PrtC family collagenase-like protease	540.8	410
411	MGCS36044_01436			*aspC*	aspartate aminotransferase protein AspC	540.5	411
412	MGCS36044_01932			*potA*	spermidine putrescine ABC transport system	539.5	412
413	MGCS36044_03898			*gpsA*	NAD(P)H-dependent glycerol-3-phosphate	539.5	412
414	MGCS36044_01102			*ybeY*	rRNA maturation RNase YbeY	538.8	414
415	MGCS36044_03364			*mraY*	phospho-N-acetylmuramoyl-pentapeptide-	538.5	415
416	MGCS36044_02306	Lipo		*ptsS*	phosphate ABC transporter substrate-binding lipoprotein PstS	535.5	416
417	MGCS36044_01302			*lgt*	prolipoprotein diacylglyceryl transferase Lgt	534.5	417
418	MGCS36044_02950			*kup*	potassium uptake protein Kup	534.5	417
419	MGCS36044_03348			*pncB*	nicotinate phosphoribosyltransferase PncB	533.3	419
420	MGCS36044_01792			*clpX*	ATP-dependent Clp protease, ATP-binding subunit	531.0	420
421	MGCS36044_00912			*yneF*	YneF family protein	529.5	421
422	MGCS36044_03490			-	GNAT family N-acetyltransferase	527.8	422
423	MGCS36044_00800			*oppB_1*	oligopeptide ABC transporter permease protein	527.5	423
424	MGCS36044_03552			-	LD/DD carboxypeptidase family protein	527.3	424
425	MGCS36044_01484			*fldA*	flavodoxin FldA	527.0	425
426	MGCS36044_00558			*def*	peptide deformylase Def	524.5	426
427	MGCS36044_02832			*brkB*	BrkB family protein	522.5	427
428	MGCS36044_03952			*proW*	proline/glycine betaine ABC transporter ATPase	521.0	428

No.	Locus tag	SignalP6 predicted(1)	Virulence(2)	Gene	Function	RPKMs(3)	RANK(4)
429	MGCS36044_01730			*fruR*	fructose operon transcriptional repressor	520.8	429
430	MGCS36044_01606			-	metal-sulfur cluster assembly factor	520.3	430
431	MGCS36044_03000			-	DegV family EDD domain-containing protein	519.3	431
432	MGCS36044_00024	Secreted		-	class A beta-lactamase-related serine hydrolase	517.5	432
433	MGCS36044_02878			*udk*	uridine kinase Udk	517.5	432
434	MGCS36044_03366			*pbp2X*	PG transpeptidase class B penicillin-binding	517.3	434
435	MGCS36044_02264			*coaC*	phosphopantothenoylcysteine decarboxylase CoaC	516.0	435
436	MGCS36044_01230			*glnP_1*	glutamine ABC transporter permease GlnP	515.8	436
437	MGCS36044_02028			*srtA*	class A sortase SrtA	514.8	437
438	MGCS36044_02964			-	TIGR02206 family membrane protein	513.5	438
439	MGCS36044_01652			*ltaS*	LTA synthase LtaS	513.3	439
440	MGCS36044_02232			*nrdD_1*	ATP cone domain-containing protein,	513.0	440
441	MGCS36044_03060			*ftsQ*	cell division protein FtsQ/DivIB	511.8	441
442	MGCS36044_02402			*parC*	DNA topoisomerase IV subunit A ParC	511.5	442
443	MGCS36044_02378			-	ABC transporter ATP-binding protein	511.3	443
444	MGCS36044_00356			-	deoxyadenosine kinase	511.0	444
445	MGCS36044_03110			*sdrC*	SdrC family PDZ domain-containing protein	510.3	445
446	MGCS36044_01638			*ebsA*	EbsA family pore-forming protein	508.8	446
447	MGCS36044_02864			*metK*	methionine adenosyltransferase MetK	504.5	447
448	MGCS36044_01570			*epuA*	DNA-directed RNA polymerase beta subunit EpuA	502.5	448
449	MGCS36044_01790			-	hypothetical protein	502.5	448
450	MGCS36044_02998			-	SGNH-hydrolase superfamily of lipases and	502.0	450
451	MGCS36044_00588	Secreted		-	putative secreted protein	501.5	451
452	MGCS36044_03280			*raiA*	ribosome-associated translation inhibitor RaiA	501.0	452
453	MGCS36044_02398			-	DUF2969 domain-containing protein	500.5	453
454	MGCS36044_02762			-	helix-turn-helix domain-containing putative	498.3	454
455	MGCS36044_00358			*dus*	tRNA-dihydrouridine synthase Dus	497.5	455
456	MGCS36044_00584			*mrnC*	mini-ribonuclease 3 MrnC	497.3	456
457	MGCS36044_04252			*glcU*	glucose uptake permease GlcU	495.3	457
458	MGCS36044_02140			-	FAD-binding oxidoreductase	494.8	458
459	MGCS36044_01938			*potD*	spermidine putrescine ABC transport system substrate-binding protein PotD	492.8	459
460	MGCS36044_01234	Secreted		-	glutamine ABC transporter substrate-binding	491.8	460
461	MGCS36044_01640			-	LysM peptidoglycan-binding domain-containing protein	491.5	461
462	MGCS36044_00744			*pyrG*	CTP synthase Pyg	491.3	462
463	MGCS36044_01300			*hprK*	HPr(Ser) kinase/phosphatase HprK	490.0	463
464	MGCS36044_02212		Virulence	*yqfA*	membrane channel forming/hemolysin III protein	487.5	464
465	MGCS36044_02136			*ffh*	signal recognition particle protein	486.8	465
466	MGCS36044_01232			-	glutamine ABC transporter permease	486.5	466
467	MGCS36044_01056			*nudF*	NUDIX hydrolase NudF	485.3	467
468	MGCS36044_01088			*cvfB*	S1 RNA-binding domain-containing protein CvfB	485.0	468
469	MGCS36044_01194		Virulence	*ccpA*	catabolite control protein CcpA	484.3	469
470	MGCS36044_02538			*lplA_2*	lipoate--protein ligase LplA	482.3	470
471	MGCS36044_02200			*topA*	type I DNA topoisomerase TopA	480.3	471

No.	Locus tag	SignalP6 predicted[(1)]	Virulence[(2)]	Gene	Function	RPKMs[(3)]	RANK[(4)]
472	MGCS36044_03550			-	FlgJ-related putative peptidoglycan hydrolase	480.3	471
473	MGCS36044_00780			*mecA*	negative regulator of genetic competence,	478.3	473
474	MGCS36044_02560			*rnjA_1*	mRNA degradation ribonuclease RnjA	477.8	474
475	MGCS36044_01058			*macP*	cell wall synthase accessory phosphoprotein	476.8	475
476	MGCS36044_04138			*aspS*	aspartyl-tRNA synthetase	475.8	476
477	MGCS36044_00310			*pbp1B*	bifunctional PG transglycosylase-transpeptidase,	475.3	477
478	MGCS36044_01936			*potC*	spermidine putrescine ABC transport system permease protein PotC	474.8	478
479	MGCS36044_04226			*sdhB*	L-serinedehydratase beta subunit SdhB	470.8	479
480	MGCS36044_03376			-	hypothetical protein	470.5	480
481	MGCS36044_01238		Virulence	*vicR*	TCS DNA-binding response regulator VicR	470.3	481
482	MGCS36044_02968			*ddl*	D-alanine--D-alanine ligase Ddl	467.5	482
483	MGCS36044_02290		Virulence	*ciaH*	TCS sensor histidine kinase protein CiaH	467.0	483
484	MGCS36044_00754			-	alpha/beta hydrolase	465.5	484
485	MGCS36044_03700		Virulence	*cppA*	CppA family putative C3-glycoprotein degrading	464.8	485
486	MGCS36044_00032			*plaP*	amino acid permease PlaP	464.0	486
487	MGCS36044_02006			-	Sua5/YciO/YrdC/YwlC family protein ribosome	464.0	486
488	MGCS36044_01620			*rgpF*	alpha-L-Rha alpha-1,3-L-rhamnosyltransferase	462.8	488
489	MGCS36044_03946			*yccU*	YccU family CoA-binding protein	459.0	489
490	MGCS36044_00920			-	CBS domain-containing protein	456.8	490
491	MGCS36044_01930			*murB*	UDP-N-acetylmuramate dehydrogenase MurB	456.0	491
492	MGCS36044_03176			-	VOC family protein	455.8	492
493	MGCS36044_00892			-	hypothetical protein	455.3	493
494	MGCS36044_02496			*lepA*	translation elongation factor 4 LepA	452.8	494
495	MGCS36044_03564			*gatC_2*	aspartyl-tRNA(Asn) or glutamyl-tRNA(Gln) amidotransferase C subunit GatC	450.8	495
496	MGCS36044_00926			*scp2*	segregation/condensation complex subunit (B)	450.5	496
497	MGCS36044_00886			*dnaI*	primosomal protein DnaI	450.0	497
498	MGCS36044_00546			*rseP*	RIP metalloprotease RseP	447.3	498
499	MGCS36044_03948			-	hypothetical protein	447.3	498
500	MGCS36044_01636			*pepT*	peptidase (T) PepT	446.8	500
501	MGCS36044_01348			*ftsW*	cell division protein FtsW	446.3	501
502	MGCS36044_00996			*rsmI*	16S rRNA (cytidine(1402)-2'-O)-methyltransferase	445.8	502
503	MGCS36044_01100			-	uracil DNA glycosylase superfamily protein	445.5	503
504	MGCS36044_00772			-	ABC amino acid transporter ATP-binding protein	445.3	504
505	MGCS36044_00836			*rsfS*	ribosome silencing factor RsfS	444.5	505
506	MGCS36044_01060			*mtnN*	5'-methylthioadenosine/adenosylhomocysteine	441.8	506
507	MGCS36044_03112			*coaD*	pantetheine-phosphate adenylyltransferase CoaD	441.3	507
508	MGCS36044_00552			*polC*	DNA polymerase III PolC	441.0	508
509	MGCS36044_02552			*acoA*	Pyruvate dehydrogenase E1 component alpha	441.0	508
510	MGCS36044_00774	Secreted		-	ABC amino acid transporter substrate-binding	440.5	510
511	MGCS36044_01576			*pheT*	phenylalanyl-tRNA synthetase beta subunit PheT	440.5	510
512	MGCS36044_01656			*aroD*	type I 3-dehydroquinate dehydratase AroD	440.3	512
513	MGCS36044_03072			*pspE*	PspE family rhodanese-like domain-containing	439.8	513
514	MGCS36044_02008			-	GNAT family N-acetyltransferase	437.3	514

No.	Locus tag	SignalP6 predicted(1)	Virulence(2)	Gene	Function	RPKMs(3)	RANK(4)
515	MGCS36044_01964			-	DUF1831 domain-containing protein	437.0	515
516	MGCS36044_01188			*gloA*	lactoylglutathione lyase protein GloA	434.8	516
517	MGCS36044_00930			*yidD*	membrane protein insertion efficiency factor	434.0	517
518	MGCS36044_01198			-	glycosyltransferase	432.8	518
519	MGCS36044_00112			*acpP_1*	acyl carrier protein AcpP	431.8	519
520	MGCS36044_00582			*cysS*	cysteine--tRNA synthetase CysS	431.8	519
521	MGCS36044_02404			-	hypothetical protein	431.5	521
522	MGCS36044_00580			-	hypothetical protein	430.8	522
523	MGCS36044_01498			*gyrB*	DNA topoisomerase ATP-hydrolyzing B subunit	430.3	523
524	MGCS36044_01632			*galE*	UDP-glucose 4-epimerase GalE	429.8	524
525	MGCS36044_03368			*ftsL*	cell division protein FtsL	429.8	524
526	MGCS36044_01622			-	glycosyltransferase family 2 protein	428.5	526
527	MGCS36044_02450			*nfnB*	NfnB family nitroreductase	428.5	526
528	MGCS36044_03648			-	CorA family divalent cation transport protein	426.0	528
529	MGCS36044_01608			*rmlD*	dTDP-4-dehydrorhamnose reductase protein RmlD	425.8	529
530	MGCS36044_01304			-	COG4768 superfamily YoxC-like protein	425.3	530
531	MGCS36044_03178			-	helix-hairpin-helix domain-containing protein	424.3	531
532	MGCS36044_03062			*murG*	UDP-N-acetylglucosamine--N-acetylmuramyl-	423.0	532
533	MGCS36044_03554			-	PhoE family broad specificity phosphatase	422.8	533
534	MGCS36044_00876			*yceD*	large ribosomal RNA subunit accumulation protein	422.3	534
535	MGCS36044_00922			*xerD_1*	site-specific tyrosine recombinase XerD	418.5	535
536	MGCS36044_04102			-	SSRC41 RNA	418.3	536
537	MGCS36044_02026			*gyrA*	DNA gyrase subunit A GyrA	418.0	537
538	MGCS36044_04092			-	DUF2079 domain-containing protein	418.0	537
539	MGCS36044_03508			-	DUF956 family protein	417.0	539
540	MGCS36044_01642			*cmk*	CMP kinase Cmk	415.0	540
541	MGCS36044_00302			*adcR*	zinc-dependent MarR family transcriptional	413.5	541
542	MGCS36044_04140			*hisS*	histidine--tRNA synthase HisS	413.0	542
543	MGCS36044_01616			*rgpD*	ABC transporter polysaccharide/polyol phosphate ATPase component RgpD	412.3	543
544	MGCS36044_00360			*hslO*	Hsp33 family molecular chaperone HslO	412.0	544
545	MGCS36044_03136			-	B3/4 domain-containing protein	411.8	545
546	MGCS36044_03678			-	hypothetical protein	411.3	546
547	MGCS36044_01670			*thiI*	thiamine biosynthesis/tRNA modification protein	410.8	547
548	MGCS36044_00828			*yhbY*	ribosome assembly RNA-binding protein YhbY	410.5	548
549	MGCS36044_04020			*groES*	co-chaperone GroES	409.8	549
550	MGCS36044_04212			-	DHH family phosphoesterase	409.3	550
551	MGCS36044_00010			*engD*	redox-regulated ATPase EngD	408.5	551
552	MGCS36044_02002			*prfA*	peptide chain release factor 1 PrfA	408.3	552
553	MGCS36044_04216			*mnmG*	tRNA uridine-5-carboxymethylaminomethyl(34)	408.3	552
554	MGCS36044_03086			-	VanZ like family glycopeptide antibiotic	407.8	554
555	MGCS36044_02586			-	tetra tricopeptide repeat family protein	407.5	555
556	MGCS36044_03668			-	colicin V production family protein	407.3	556
557	MGCS36044_00768			-	SPFH domain-containing protein	407.0	557

No.	Locus tag	SignalP6 predicted(1)	Virulence(2)	Gene	Function	RPKMs(3)	RANK(4)
558	MGCS36044_01048			-	ASCH domain-containing RNA-binding protein	407.0	557
559	MGCS36044_01062		Virulence	*mtsR*	metal-dependent transcriptional regulator MtsR	407.0	557
560	MGCS36044_02400			*bcaT*	branched-chain amino acid aminotransferase	407.0	557
561	MGCS36044_00952			-	cytoplasmic protein	406.8	561
562	MGCS36044_02704			*obgE*	GTPase ObgE	406.5	562
563	MGCS36044_00306			*adcB*	metal ABC transporter permease AdcB	406.3	563
564	MGCS36044_01192			*pepQ*	Xaa-Pro dipeptidase protein PepQ	405.8	564
565	MGCS36044_01618			*rgpE*	glycosyltransferase family GT2 protein RgpE	404.5	565
566	MGCS36044_00594			-	DegV family protein	403.0	566
567	MGCS36044_00924			*scp1*	segregation/condensation complex subunit (A)	402.8	567
568	MGCS36044_03402			*glyQ*	glycine--tRNA ligase alpha subunit GlyQ	401.5	568
569	MGCS36044_00918			-	YfcE family metallophosphoesterase	400.3	569
570	MGCS36044_03486			-	chemotaxis protein	400.3	569
571	MGCS36044_03956			-	diacylglycerol kinase family lipid kinase	399.5	571
572	MGCS36044_00884			*dnaB*	replication initiation and membrane attachment	398.3	572
573	MGCS36044_01324			-	PhoE superfamily phosphatase	398.3	572
574	MGCS36044_03332			*rlmL*	23S rRNA G2445 N2-methylase RlmL	395.8	574
575	MGCS36044_01072	Lipo		-	peptidylprolyl isomerase lipoprotein	395.0	575
576	MGCS36044_03116			*asnA*	asparagine synthetase AsnA	393.8	576
577	MGCS36044_02022			-	FolA superfamily dihydrofolate reductase	393.5	577
578	MGCS36044_02960			-	Pleckstrin homology-like domain-containing	393.0	578
579	MGCS36044_00562			-	major facilitator transporter family protein	391.0	579
580	MGCS36044_03090			*yutD*	YutD family protein of unknown function	391.0	579
581	MGCS36044_02434			-	LytR family transcriptional regulator	390.5	581
582	MGCS36044_04206			-	Veg family protein	389.8	582
583	MGCS36044_00866			*ktrA*	potassium uptake transporter gating subunit	389.5	583
584	MGCS36044_03826			*rpe*	ribulose-phosphate 3-epimerase Rpe	388.0	584
585	MGCS36044_02348			-	glycine RNA	387.8	585
586	MGCS36044_02966			*murF*	UDP-N-acetylmuramoyl-tripeptide--D-alanyl-D-	387.5	586
587	MGCS36044_01180			-	cell division protein FtsW-like	386.8	587
588	MGCS36044_04004			*pbp2A*	multimodular transpeptidase-transglycosylase	386.3	588
589	MGCS36044_00850	Lipo		-	amino acid ABC transporter substrate-binding	385.8	589
590	MGCS36044_01628			-	glycosyltransferase family 1 protein	384.5	590
591	MGCS36044_04136			-	YitT family protein putative ABC transporter ATPase	384.5	590
592	MGCS36044_04086			*mviM*	MviM family predicted dehydrogenase	384.3	592
593	MGCS36044_03480			*ecsB*	ABC exoprotein transporter permease EcsB	383.3	593
594	MGCS36044_01326			*yccF*	YccF domain-containing protein	382.8	594
595	MGCS36044_00110			*plsX*	phosphate acyltransferase PlsX	382.5	595
596	MGCS36044_00304			*adcC*	metal ABC transporter ATP-binding protein AdcC	382.5	595
597	MGCS36044_00522			*dtd*	D-tyrosyl-tRNA deacylase Dtd	382.5	595
598	MGCS36044_00792			*sufB*	Fe-S cluster assembly protein SufB	382.5	595
599	MGCS36044_03172			-	MGCS36044_0AAA family ATPase3172	382.3	599
600	MGCS36044_02012			-	nucleoid-associated bacterial family protein	381.8	600

No.	Locus tag	SignalP6 predicted[1]	Virulence[2]	Gene	Function	RPKMs[3]	RANK[4]
601	MGCS36044_00946		Virulence	*hlyX*	hemolysin family protein HylX	381.3	601
602	MGCS36044_02984			*pyrD*	dihydroorotate dehydrogenase PyrD	380.0	602
603	MGCS36044_00896			-	GNAT family N-acetyltransferase	378.0	603
604	MGCS36044_02356	Lipo		-	amino acid ABC transporter substrate-binding lipoprotein	377.8	604
605	MGCS36044_02872			-	GAF domain containing protein	377.8	604
606	MGCS36044_03620			*acpS*	AcpS family provisional 4'-phosphopantetheinyl	376.8	606
607	MGCS36044_00914			*murI*	glutamate racemase MurI	376.0	607
608	MGCS36044_01190			-	AD(P)H-dependent oxidoreductase	376.0	607
609	MGCS36044_02870			*dnaX*	DNA polymerase III gamma/tau subunit DnaX	376.0	607
610	MGCS36044_04066			*treC*	trehalose-6-phosphate hydrolase TreC	375.8	610
611	MGCS36044_00826			*yqeH*	ribosome biogenesis GTPase YqeH	375.3	611
612	MGCS36044_03746			-	type I restriction-modification system (M)	375.3	611
613	MGCS36044_04184			-	DUF368 domain-containing protein	374.8	613
614	MGCS36044_03192			*aroB*	3-dehydroquinate synthase protein AroB	373.8	614
615	MGCS36044_00352			-	DUF4479 and tRNA-binding domain-containing	372.3	615
616	MGCS36044_02618			*dltE*	short-chain dehydrogenase DltE	371.5	616
617	MGCS36044_01602			*dnaG*	DNA primase protein DnaG	371.0	617
618	MGCS36044_00020			*divIC*	septum formation initiator family protein	370.8	618
619	MGCS36044_01538			*dagK*	diacylglycerol kinase family lipid kinase	370.8	618
620	MGCS36044_01626			-	DUF2142 domain-containing protein	369.8	620
621	MGCS36044_00504			-	M42 family metallopeptidase	368.8	621
622	MGCS36044_03796	Lipo		*oppA_2*	oligopeptide ABC transporter substrate-binding lipoprotein OppA	368.5	622
623	MGCS36044_03378			*ccmA*	CcmA family multidrug ABC transporter ATPase	368.0	623
624	MGCS36044_03418			-	Cof-type HAD-IIB family phosphohydrolase	367.3	624
625	MGCS36044_00152			*oatA*	acetyltransferase OatA	367.0	625
626	MGCS36044_00998			-	DUF5684 domain-containing protein	367.0	625
627	MGCS36044_00994			*yabA*	DNA replication initiation control protein YabA	366.8	627
628	MGCS36044_03950			*polA*	DNA polymerase I PolA	366.8	627
629	MGCS36044_01772			-	isopentenyl-diphosphate delta-isomerase	365.5	629
630	MGCS36044_01962			-	DUF4649 domain-containing protein	365.5	629
631	MGCS36044_01328			-	aminoacyl-tRNA deacylase	365.3	631
632	MGCS36044_02004			*prmC*	peptide chain release factor N(5)-glutamine	365.3	631
633	MGCS36044_01624			-	DUF2304 domain-containing protein	364.8	633
634	MGCS36044_01764			*sptS*	SptS-like TCS sensor histidine kinase	364.5	634
635	MGCS36044_01654			*rlmK*	23S rRNA methyltransferase RmlK	364.0	635
636	MGCS36044_03492			*tsaE*	tRNA (adenosine(37)-N6)-threonylcarbamoyltransferase TsaE	363.0	636
637	MGCS36044_00544			*cdsA*	phosphatidate cytidylyltransferase CdsA	362.0	637
638	MGCS36044_02534			*murE_2*	UDP-N-acetylmuramoylalanyl-D-glutamate-2,	361.0	638
639	MGCS36044_04114			*tag*	DNA-3-methyladenine glycosylase Tag	359.5	639
640	MGCS36044_03618			*alr*	alanine racemase Alr	359.3	640
641	MGCS36044_01590			*rexA*	ATP-dependent nuclease A subunit RexA	358.8	641
642	MGCS36044_01236			*glnQ_1*	glutamine ABC transporter ATPase GlnQ	358.5	642
643	MGCS36044_00384			-	PTS sugar transporter subunit IIC	358.3	643

No.	Locus tag	SignalP6 predicted(1)	Virulence(2)	Gene	Function	RPKMs(3)	RANK(4)
644	MGCS36044_01294			*tex*	RNA-binding transcriptional accessory protein	357.8	644
645	MGCS36044_03020			-	phospho-sugar mutase	357.8	644
646	MGCS36044_03482			*ecsA*	ABC exoprotein transporter ATPase EcsA	357.5	646
647	MGCS36044_03340			*recU*	Holliday junction resolvase RecU	356.5	647
648	MGCS36044_00022			-	hypothetical protein	355.5	648
649	MGCS36044_02932			-	DUF3114 domain-containing protein	355.5	648
650	MGCS36044_00392	Lipo		-	BMP family ABC transporter substrate-binding	355.3	650
651	MGCS36044_01540			*pulA_2*	type I pullulanase PulA	354.8	651
652	MGCS36044_02920			*pepF_2*	oligoendopeptidase (F) PepF	354.3	652
653	MGCS36044_00790			*sufE*	SUF system NifU family Fe-S cluster assembly	354.0	653
654	MGCS36044_02862			*murA_2*	UDP-N-acetylglucosamine 1-carboxyvinyltransferase MurA	353.5	654
655	MGCS36044_01788			*dyr*	dihydrofolate reductase Dyr	353.0	655
656	MGCS36044_03664			-	FAD-containing oxidoreductase	352.3	656
657	MGCS36044_01424			*queG*	epoxyqueuosine reductase QueG	352.0	657
658	MGCS36044_02292		Virulence	*ciaR*	TCS DNA-binding response regulator protein CiaR	351.0	658
659	MGCS36044_01050			-	Gfo/Idh/MocA family oxidoreductase	350.3	659
660	MGCS36044_02406			*parE*	DNA topoisomerase IV subunit B ParE	350.3	659
661	MGCS36044_03954			*proV*	proline/glycine betaine ABC transporter permease	350.3	659
662	MGCS36044_00554			-	MarR family transcriptional regulator	350.0	662
663	MGCS36044_02010			*glyA*	serine hydroxymethyl transferase GlyA	350.0	662
664	MGCS36044_01688			*rluD*	ribosomal large subunit pseudouridine synthase RluD	349.8	664
665	MGCS36044_02332			-	GntR family transcriptional regulator	349.0	665
666	MGCS36044_01960			*rex*	redox-sensing transcriptional repressor Rex	348.5	666
667	MGCS36044_03934			-	metallo-beta-lactamase superfamily protein	347.8	667
668	MGCS36044_01928			*folK*	2-amino-4-hydroxy-6- hydroxymethyldihydropteridine pyrophosphokinase protein FolK	347.5	668
669	MGCS36044_01782			*mvaS1*	hydroxymethylglutaryl-CoA reductase protein (1)	347.3	669
670	MGCS36044_01630			-	LTA synthase family protein	344.5	670
671	MGCS36044_01044			*fabG_1*	3-oxoacyl-ACP reductase FabG	344.0	671
672	MGCS36044_03882			-	AIM24 family protein	343.0	672
673	MGCS36044_03180			-	DUF1912 family protein	342.8	673
674	MGCS36044_02620			*rnz*	ribonuclease Rnz	342.5	674
675	MGCS36044_01322			-	HAD family hydrolase	342.3	675
676	MGCS36044_04010			*rluA_2*	RluA family pseudouridine synthase	342.3	675
677	MGCS36044_01174			*secG*	preprotein translocase subunit SecG	342.0	677
678	MGCS36044_01446			*whiA*	cell division involved DNA-binding protein WhiA	342.0	677
679	MGCS36044_00140			*purB*	adenylosuccinate lyase PurB	341.0	679
680	MGCS36044_03744			-	type I restriction-modification system	340.8	680
681	MGCS36044_03626			*manA*	mannose-6-phosphate isomerase ManA	340.0	681
682	MGCS36044_03828			*rsgA*	ribosome small subunit-dependent GTPase (A)	340.0	681
683	MGCS36044_03670			-	hypothetical protein	339.8	683
684	MGCS36044_02558			*frmB*	FrmB family esterase	339.5	684
685	MGCS36044_03672			*rnhC*	HIII ribonuclease RnhC	339.3	685
686	MGCS36044_01474			*nrnA*	bifunctional oligoribonuclease/PAP phosphatase	338.0	686

No.	Locus tag	SignalP6 predicted[(1)]	Virulence[(2)]	Gene	Function	RPKMs[(3)]	RANK[(4)]
687	MGCS36044_00300			*ipk*	4-diphosphocytidyl-2-C-methyl-D-erythritol kinase Ipk	337.8	687
688	MGCS36044_01046			-	DUF3977 family protein	337.3	688
689	MGCS36044_03646			*uvrA*	excinuclease ABC subunit (A) UvrA	337.3	688
690	MGCS36044_01634			-	RfbX superfamily lipopolysaccharide biosynthesis	337.0	690
691	MGCS36044_00002			*dnaA*	chromosomal replication initiator protein DnaA	335.8	691
692	MGCS36044_04134			-	YitT family protein putative ABC transporter ATPase	335.8	691
693	MGCS36044_01054			*glmU*	bifunctional UDP-N-acetylglucosamine	335.0	693
694	MGCS36044_00788			*sufS*	cysteine desulfurase SufS	334.8	694
695	MGCS36044_04132			-	YitT family protein putative ABC transporter	334.3	695
696	MGCS36044_03174			-	GNAT family N-acetyltransferase	334.0	696
697	MGCS36044_01270			-	permease	333.8	697
698	MGCS36044_00350			*trxA_1*	thioredoxin family protein TrxA-like	333.3	698
699	MGCS36044_01254			-	Cof-type HAD-IIB family hydrolase	331.5	699
700	MGCS36044_04112			-	VOC family protein	331.5	699
701	MGCS36044_01926			*folQ*	dihydroneopterin aldolase protein FolB	330.5	701
702	MGCS36044_02186			*gid*	tRNA (uracil-5-)-methyltransferase/glucose	330.0	702
703	MGCS36044_01614			*rgpC*	ABC transporter polysaccharide/polyol phosphate	329.5	703
704	MGCS36044_02014			-	lysozyme family protein	328.3	704
705	MGCS36044_03820			*cbf*	YhaM family 3'-5' exoribonuclease	328.3	704
706	MGCS36044_02268			*pgmA*	phospho-sugar mutase PgmA	327.5	706
707	MGCS36044_03074			*glcK*	glucokinase GlcK	327.5	706
708	MGCS36044_01712			-	glycerophosphodiester phosphodiesterase	327.3	708
709	MGCS36044_02588			*perM*	PerM family predicted purR regulated permease	326.3	709
710	MGCS36044_01184			*wcaA*	WcaA superfamily glycosyltransferase involved in	325.0	710
711	MGCS36044_02206			*ylqF*	ribosome biogenesis GTPase YlqF	325.0	710
712	MGCS36044_01242			*vicX*	zinc-dependent hydrolase protein VicX	324.5	712
713	MGCS36044_03674			*lepB_2*	signal peptidase I LepB	324.5	712
714	MGCS36044_02358			-	amino acid ABC transporter ATP-binding protein	324.0	714
715	MGCS36044_00864			*sstT*	serine/threonine transporter SstT	323.8	715
716	MGCS36044_01742			-	DUF1149 domain-containing protein	323.8	715
717	MGCS36044_00972			-	YlbF family regulatory protein	323.5	717
718	MGCS36044_01344			*pepF_1*	oligoendopeptidase PepF	323.0	718
719	MGCS36044_02446			*mnmE*	MnmE family tRNA	322.0	719
720	MGCS36044_02650	Lipo		*malE*	maltose/maltodextrin ABC transport system substrate-binding lipoprotein MalE	321.3	720
721	MGCS36044_03398			*ynzC*	DUF896 family protein	321.0	721
722	MGCS36044_01770			*mvaK2*	mevalonate kinase MvaK2	320.8	722
723	MGCS36044_03254			-	DUF960 domain-containing protein	320.3	723
724	MGCS36044_04118			*mdtH*	MdtH-related MFS mutidrug resistance	319.8	724
725	MGCS36044_01924			*folP*	dihydropteroate synthase protein FolP	318.8	725
726	MGCS36044_02346			*pcrA*	DNA helicase PcrA	317.5	726
727	MGCS36044_01008			*arsC_1*	arsenate reductase ArsC	317.0	727
728	MGCS36044_01170			-	DUF853 domain-containing protein	316.8	728
729	MGCS36044_01976			*rluA_1*	RluA family pseudouridine synthase	315.5	729

No.	Locus tag	SignalP6 predicted(1)	Virulence(2)	Gene	Function	RPKMs(3)	RANK(4)
730	MGCS36044_01922			*folE*	GTP cyclohydrolase I protein FolE	314.3	730
731	MGCS36044_02678			*uvrB*	excinuclease ABC subunit UvrB	314.3	730
732	MGCS36044_04204			-	helix-turn-helix domain-containing transcriptional regulator	314.3	730
733	MGCS36044_00928			*rluB*	ribosomal large subunit pseudouridine synthase	314.0	733
734	MGCS36044_01268			-	DUF1980 domain-containing protein	314.0	733
735	MGCS36044_01758	Secreted		-	extracellular cell wall anchored bifunctional	313.5	735
736	MGCS36044_00346			*pepA*	glutamyl aminopeptidase PepA	312.5	736
737	MGCS36044_01074			*ftsK*	cell division protein FtsK	312.3	737
738	MGCS36044_02530	Lipo		*ybbR*	YbbR family lipoprotein	311.5	738
739	MGCS36044_03068			-	DUF3165 family protein	311.3	739
740	MGCS36044_00916			*rdgB*	RdgB family non-canonical purine NTP	310.8	740
741	MGCS36044_02146			-	TCS DNA-binding response regulator (heme	310.8	740
742	MGCS36044_02204			*rnhB*	HII ribonuclease RnhB	310.8	740
743	MGCS36044_03114			*rsmD*	16S rRNA (guanine(966)-N(2))-methyltransferase	310.3	743
744	MGCS36044_03588			-	HAD-related haloacid dehalogenase hydrolase	309.5	744
745	MGCS36044_03868		Virulence	*fasA*	TCS response regulator	309.5	744
746	MGCS36044_03822			*rmuC*	DNA recombination protein RmuC	308.8	746
747	MGCS36044_01920			*folC*	dihydrofolate synthase FolC	308.3	747
748	MGCS36044_03354			-	DUF4059 family protein	307.8	748
749	MGCS36044_03038			-	NUDIX hydrolase	306.8	749
750	MGCS36044_00502			-	Udk family kinase	306.0	750
751	MGCS36044_03886			*radA*	DNA repair protein RadA	305.3	751
752	MGCS36044_00514		Virulence	*mga*	M protein trans-acting positive regulator Mga	304.8	752
753	MGCS36044_02316			*ribF*	bifunctional riboflavin kinase/FAD synthetase	304.8	752
754	MGCS36044_00150			-	hypothetical protein	303.8	754
755	MGCS36044_00530			*msmK*	sn-glycerol-3-phosphate ABC transporter	303.8	754
756	MGCS36044_02126			-	SpF66_sRNA	303.3	756
757	MGCS36044_02430			-	hypothetical protein	303.0	757
758	MGCS36044_01536			*ligA*	NAD-dependent DNA ligase LigA	302.5	758
759	MGCS36044_02624			*hflX*	GTP-binding protein HflX	302.3	759
760	MGCS36044_01246			*smc*	chromosome segregation protein Smc	301.8	760
761	MGCS36044_01768			*mvaD*	diphosphomevalonate decarboxylase MvaD	301.5	761
762	MGCS36044_00520			*relA*	bifunctional (p)ppGpp synthase/hydrolase RelA	299.3	762
763	MGCS36044_00974			-	YlbG family protein	299.3	762
764	MGCS36044_00824			*yqeG*	HAD IIIA-type phosphatase YqeG	298.5	764
765	MGCS36044_01748			-	Uup family ATPase components of ABC transporters	298.5	764
766	MGCS36044_03328			*luxS*	S-ribosylhomocysteine lyase	298.5	764
767	MGCS36044_02248			*fhs_1*	formate--tetrahydrofolate ligase Fhs	296.5	767
768	MGCS36044_00860			*metP_1*	methionine ABC transporter permease MetP	296.3	768
769	MGCS36044_00786			*sufD*	Fe-S cluster assembly protein SufD	295.5	769
770	MGCS36044_03304			*rsmB*	16S rRNA (cytosine(967)-C(5))-methyltransferase	295.5	769
771	MGCS36044_03784			-	DUF1447 family protein	295.5	769
772	MGCS36044_03360	Secreted		*hisJ*	HisJ family amino acid ABC transporter	295.0	772

No.	Locus tag	SignalP6 predicted(1)	Virulence(2)	Gene	Function	RPKMs(3)	RANK(4)
773	MGCS36044_03190			*aroGA*	bifunctional 3-deoxy-7-phosphoheptulonate	294.3	773
774	MGCS36044_02604			*zupT*	ZIP family metal transporter ZupT	293.3	774
775	MGCS36044_01958			-	gamma-glutamyl-gamma-aminobutyrate hydrolase	293.0	775
776	MGCS36044_03824			*thiN*	thiamine diphosphokinase ThiN	292.0	776
777	MGCS36044_00990			*holB*	DNA polymerase III subunit delta' HolB	291.5	777
778	MGCS36044_03684			-	ElaA-related predicted N-acetyltransferase	291.5	777
779	MGCS36044_02262			*coaB*	phosphopantothenate--cysteine ligase CoaB	291.3	779
780	MGCS36044_00344			*proC*	pyrroline-5-carboxylate reductase ProC	289.8	780
781	MGCS36044_01342			*btuE*	BtuE superfamily glutathione peroxidase	289.0	781
782	MGCS36044_02616			*recJ*	single-stranded-DNA-specific exonuclease RecJ	289.0	781
783	MGCS36044_01476			-	GNAT family N-acetyltransferase	288.8	783
784	MGCS36044_00962			*murE_1*	UDP-N-acetylmuramoyl-L-alanyl-D-glutamate--L-	288.0	784
785	MGCS36044_01786			*thyA*	thymidylate synthase ThyA	288.0	784
786	MGCS36044_03420			-	YadS family trimeric intracellular cation	287.3	786
787	MGCS36044_02826			-	LCP family anionic cell polymer synthesis	287.0	787
788	MGCS36044_02518			-	MGCS360TIGR01906 family membrane protein44_02518	285.5	788
789	MGCS36044_00292			*cydB*	cytochrome d ubiquinol oxidase subunit (II)	285.3	789
790	MGCS36044_01548			*glgA*	glycogen synthase GlgA	284.8	790
791	MGCS36044_01612			*rgpB*	glycosyltransferase family GT2 protein RgpB	284.5	791
792	MGCS36044_03404			*ypbQ*	YpbQ family isoprenylcysteine carboxyl	283.8	792
793	MGCS36044_03778			*tsaD*	tRNA (adenosine(37)-N6)-threonylcarbamoyltransferase complex transferase subunit TsaD	283.0	793
794	MGCS36044_00940			-	PgpB family membrane-associated phospholipid	282.3	794
795	MGCS36044_02360			-	amino acid ABC transporter permease	282.0	795
796	MGCS36044_04088			-	hypothetical protein	282.0	795
797	MGCS36044_02622			-	hypothetical protein	280.8	797
798	MGCS36044_04028			*cspA*	cold-shock protein CspA	280.8	797
799	MGCS36044_01666			-	hypothetical protein	280.3	799
800	MGCS36044_01784			*mvaS2*	hydroxymethylglutaryl-CoA synthase protein (2)	280.0	800
801	MGCS36044_03372			*proA*	glutamate-5-semialdehyde dehydrogenase ProA	280.0	800
802	MGCS36044_02222			-	Uup family ATPase components of ABC transporters with duplicated ATPase domains	279.8	802
803	MGCS36044_01986			*osmF*	OsmF superfamily glycine/betaine transport	278.3	803
804	MGCS36044_00890			*snf*	SWF/SNF family helicase	277.0	804
805	MGCS36044_02162	Lipo		-	FMN-binding protein, major membrane immunogen,	276.8	805
806	MGCS36044_04082			*nrdG*	anaerobic ribonucleoside-triphosphate reductase	276.3	806
807	MGCS36044_01228			-	DUF3114 domain-containing protein	276.0	807
808	MGCS36044_03666			*mutS2*	DNA mismatch repair endonuclease MutS2	275.3	808
809	MGCS36044_00026			*tilS*	tRNA lysidine(34) synthetase TilS	275.0	809
810	MGCS36044_02880			*srmB*	SrmB superfamily II DNA and RNA helicase	274.5	810
811	MGCS36044_03592			-	aldo/keto reductase	274.3	811
812	MGCS36044_00868			*ktrB*	potassium uptake transporter channel subunit	273.8	812
813	MGCS36044_01098			*phoH*	phosphate starvation-inducible protein PhoH	273.8	812
814	MGCS36044_02930			*queA*	S-adenosylmethionine ribosyltransferase-isomerase QueA	273.5	814

No.	Locus tag	SignalP6 predicted(1)	Virulence(2)	Gene	Function	RPKMs(3)	RANK(4)
815	MGCS36044_02556			-	hypothetical protein	272.5	815
816	MGCS36044_01176			*rnr*	exoribonuclease (R) Rnr	272.0	816
817	MGCS36044_02824			-	23S rRNA (uracil(1939)-C(5))-methyltransferase	272.0	816
818	MGCS36044_04080			*yaaA*	peroxide stress protein YaaA	272.0	816
819	MGCS36044_01574			*pheS*	phenylalanyl-tRNA synthetase alpha subunit PheS	270.8	819
820	MGCS36044_03596			*cbiO*	cobalt ECF transporter (A) ATPase component	270.0	820
821	MGCS36044_03832			*rsmA*	16S rRNA (adenine(1518)-N(6)/adenine(1519)-N(6))-dimethyltransferase RsmA	270.0	820
822	MGCS36044_01974			*nadK*	NAD kinase NadK	269.8	822
823	MGCS36044_04116			*ruvA*	Holliday junction ATP-dependent DNA helicase	269.3	823
824	MGCS36044_00146			*ruvB*	Holliday junction branch migration DNA helicase	269.0	824
825	MGCS36044_01746			-	tRNA CCA-pyrophosphorylase	268.0	825
826	MGCS36044_01796			-	hypothetical protein	268.0	825
827	MGCS36044_01496			-	HAD-IA family hydrolase	267.8	827
828	MGCS36044_01610			*rgpA*	glycosyltransferase family 1 protein RgpA	267.8	827
829	MGCS36044_02318			*truB*	tRNA pseudouridine(55) synthase TruB	267.8	827
830	MGCS36044_03238			-	LLM class flavin-dependent oxidoreductase	266.8	830
831	MGCS36044_01480			*add*	adenosine deaminase Add	266.3	831
832	MGCS36044_04218			-	NUDIX domain-containing protein	266.3	831
833	MGCS36044_00148			-	low molecular weight phosphotyrosine protein	265.8	833
834	MGCS36044_02636			-	rhodanese-related sulfurtransferase	265.0	834
835	MGCS36044_03018			*folD*	bifunctional methylenetetrahydrofolate	265.0	834
836	MGCS36044_00494			*rsmE*	16S rRNA (uracil(1498)-N(3))-methyltransferase	264.8	836
837	MGCS36044_02410			*pyrC*	dihydroorotase PyrC	263.8	837
838	MGCS36044_02970			*recR*	recombination mediator RecR	263.5	838
839	MGCS36044_00858			*metN_1*	methionine ABC transporter ATP-binding protein	263.3	839
840	MGCS36044_02520			*nagD*	NagD family hydrolase	262.5	840
841	MGCS36044_01274			-	NUDIX hydrolase superfamily protein	262.3	841
842	MGCS36044_01722			*trmD*	tRNA (guanosine(37)-N1)-methyltransferase TrmD	261.8	842
843	MGCS36044_04084			-	putative acetyltransferase	261.8	842
844	MGCS36044_03932			*tadA*	tRNA adenosine(34) deaminase TadA	261.3	844
845	MGCS36044_00154			*adhE*	bifunctional acetaldehyde-CoA/alcohol	260.5	845
846	MGCS36044_01918			*thrB*	ThrB family homoserine kinase	260.3	846
847	MGCS36044_03064			*murD*	UDP-N-acetylmuramoyl-L-alanine--D-glutamate	259.8	847
848	MGCS36044_00484			*rarA*	replication-associated recombination protein A	259.5	848
849	MGCS36044_03788			*oppF_2*	oligopeptide ABC transporter ATPase OppF	259.3	849
850	MGCS36044_03082			-	MdlB family multidrug ABC transporter	258.3	850
851	MGCS36044_00992			*yaaT*	cell fate regulator YaaT	257.5	851
852	MGCS36044_02820			-	DinB family protein	257.5	851
853	MGCS36044_00964			*murJ*	peptidoglycan lipid-II intermediate flippase	256.8	853
854	MGCS36044_02910			*yrrM*	YrrM family O-methyltransferase	256.8	853
855	MGCS36044_04006			-	translation initiation inhibitor	256.5	855
856	MGCS36044_02150			-	cupin domain-containing protein	255.5	856
857	MGCS36044_02608			*trmK*	tRNA (adenine(22)-N(1))-methyltransferase TrmK	254.8	857

No.	Locus tag	SignalP6 predicted[1]	Virulence[2]	Gene	Function	RPKMs[3]	RANK[4]
858	MGCS36044_03888			*dut*	deoxyuridine 5'-triphosphate nucleotidohydrolase	254.8	857
859	MGCS36044_03706			*pepX*	PepX family Xaa-Pro dipeptidyl-peptidase	254.5	859
860	MGCS36044_03350			*ansP*	AnsP family L-asparagine transporter and related	254.3	860
861	MGCS36044_00542			*uppS*	UDP pyrophosphate synthase UppS	253.5	861
862	MGCS36044_03594			*recG*	ATP-dependent DNA helicase RecG	253.5	861
863	MGCS36044_00560			-	Crp family cyclic nucleotide-binding	253.0	863
864	MGCS36044_00746			*lytS*	TCS sensor histidine kinase LytS	253.0	863
865	MGCS36044_03294		Virulence	*liaR*	three component system signal transduction response regulator protein	252.8	865
866	MGCS36044_01006			*ogt*	O6-methylguanine-DNA--protein-cysteine	252.5	866
867	MGCS36044_00006			-	DUF951 doamin-containing protein	252.3	867
868	MGCS36044_03960			*bglG_3*	BglG family transcription antiterminator	252.3	867
869	MGCS36044_03708			-	pyridoxamine 5'-phosphate oxidase family	252.0	869
870	MGCS36044_00386			-	toxic anion resistance protein, tellurite	251.5	870
871	MGCS36044_03484			*hit*	HIT family protein	251.0	871
872	MGCS36044_02646			*malR*	maltose operon transcriptional repressor MalR	249.5	872
873	MGCS36044_04120			*mutL*	DNA mismatch repair endonuclease MutL	249.3	873
874	MGCS36044_02606			-	Nif3-like dinuclear metal center hexameric	248.3	874
875	MGCS36044_01178			*smpB*	SsrA(tmRNA)-binding protein SmpB	247.8	875
876	MGCS36044_03016			-	PfkB superfamily kinase	247.8	875
877	MGCS36044_01686			*lspA*	lipoprotein signal peptidase II LspA	247.5	877
878	MGCS36044_02504			-	CRISPR-DR22 RNA	247.5	877
879	MGCS36044_00160			*thrC*	threonine synthase ThrC	246.5	879
880	MGCS36044_03004			*recN*	DNA repair protein RecN	246.5	879
881	MGCS36044_01798			*clpL*	ATP-dependent Clp protease ATP-binding subunit	245.8	881
882	MGCS36044_04236			*cbiO2*	cobalt ABC transporter ATPase CbiO1	245.8	881
883	MGCS36044_01258			-	MFS transporter	245.5	883
884	MGCS36044_01566			-	DUF1146 domain-containing protein	244.8	884
885	MGCS36044_00778			*uppP*	undecaprenyl pyrophosphate phosphatase UppP	243.0	885
886	MGCS36044_01676			-	L21_leader RNA	242.0	886
887	MGCS36044_02532			-	DisA N domain-containing diadenylate cyclase	241.5	887
888	MGCS36044_01762			*sptR*	SptR-like TCS DNA-binding response regulator	241.3	888
889	MGCS36044_01252			-	Cof-type HAD-IIB family hydrolase	240.3	889
890	MGCS36044_02148			-	TCS histidine kinase sensor	239.0	890
891	MGCS36044_03598			*cbiQ_1*	cobalt ECF transporter (T) transmembrane	238.8	891
892	MGCS36044_01346			*ppc*	phosphoenolpyruvate carboxylase Ppc	238.5	892
893	MGCS36044_03374			*proB*	glutamate 5-kinase ProB	238.5	892
894	MGCS36044_00862			*brnQ_1*	branched-chain amino acid transport system II	238.3	894
895	MGCS36044_01664			-	dihydrofolate synthase	238.3	894
896	MGCS36044_02156			-	NusG domain II-containing protein	238.0	896
897	MGCS36044_03296		Virulence	*liaS*	three component system signal transduction sensor histidine kinase protein	237.5	897
898	MGCS36044_01310			-	PrtC family collagenase-like protease	237.0	898
899	MGCS36044_02822			-	CD1845 family protein	236.5	899
900	MGCS36044_04002			*secE*	preprotein translocase subunit protein SecE	236.5	899

No.	Locus tag	SignalP6 predicted(1)	Virulence(2)	Gene	Function	RPKMs(3)	RANK(4)
901	MGCS36044_00380			-	PAS domain-containing protein	236.3	901
902	MGCS36044_00334			-	class I SAM-dependent methyltransferase	236.0	902
903	MGCS36044_00748			*lytR*	TCS DNA-binding response regulator LytR	235.5	903
904	MGCS36044_01444			*cofD*	CofD/YvcK superfamily 2-phospho-L-lactate	235.0	904
905	MGCS36044_03084			-	SmdA family multidrug ABC transporter	235.0	904
906	MGCS36044_01998			*pptA*	phenylpyruvate tautomerase PptA	233.8	906
907	MGCS36044_01432			-	MBL fold metallo-hydrolase	233.0	907
908	MGCS36044_02484			*yeiH*	YeiH family membrane protein	233.0	907
909	MGCS36044_00960			*fhuA*	ferrichrome ABC transporter ATP-binding protein	232.8	909
910	MGCS36044_01442			*rapZ*	RNase adapter RapZ	232.8	909
911	MGCS36044_02366			*lepB_1*	signal peptidase I	232.5	911
912	MGCS36044_01004			-	GNAT family N-acetyltransferase	232.0	912
913	MGCS36044_02256			*gcvH*	glycine cleavage system protein H GcvH	232.0	912
914	MGCS36044_02944	Secreted		*plsC*	secreted 1-acyl-sn-glycerol-3-phosphate	231.8	914
915	MGCS36044_04250			*recF*	DNA replication/repair protein RecF	231.8	914
916	MGCS36044_03698			-	beta-lactamase family protein	231.3	916
917	MGCS36044_02000			*tdk*	thymidine kinase Tdk	230.5	917
918	MGCS36044_03924			-	hypothetical protein	230.5	917
919	MGCS36044_00362		Virulence	*rofA*	pilus transcriptional regulator RofA	230.3	919
920	MGCS36044_01172			-	multidrug efflux MFS transporter	229.8	920
921	MGCS36044_03624			-	IS30 family transposase	229.8	920
922	MGCS36044_00882			*nrdR*	transcriptional regulator NrdR	228.8	922
923	MGCS36044_04124			-	YmcA-related cell fate/competence/biofilm	228.8	922
924	MGCS36044_04234			*cbiQ_2*	cobalt ABC transporter permease CbiQ	228.8	922
925	MGCS36044_00512			*nrdI_1*	class Ib ribonucleoside-diphosphate reductase	228.5	925
926	MGCS36044_03792			*oppC_2*	oligopeptide ABC transporter permease OppC	227.8	926
927	MGCS36044_00794	Secreted		*dacA_1*	secreted D-alanyl-D-alanine carboxypeptidase	227.5	927
928	MGCS36044_02710			-	CorA family divalent cation transport protein	227.0	928
929	MGCS36044_03740			-	major facilitator superfamily protein	226.5	929
930	MGCS36044_03790			*oppD_2*	oligopeptide ABC transporter ATPase OppD	226.0	930
931	MGCS36044_02590			*mutX*	8-oxo-dGTP diphosphatase MutX	225.0	931
932	MGCS36044_03834			*rrmV*	5S rRNA maturation endonuclease RnmV	224.8	932
933	MGCS36044_01280			*pnpS*	phosphate TCS signal transductionhistidine	223.0	933
934	MGCS36044_04126			*argR_3*	arginine repressor ArgR	223.0	933
935	MGCS36044_03134			*argR_2*	arginine responsive transcriptional repressor	222.5	935
936	MGCS36044_01076			-	DUF3397 domain-containing protein	222.3	936
937	MGCS36044_03440			*copA_2*	copper-exporting ATPase CopA	222.3	936
938	MGCS36044_00290			*cydA*	cytochrome ubiquinol oxidase subunit (I) CydA	221.5	938
939	MGCS36044_01572			*endA*	DNA-entry competence-associated nuclease EndA	221.5	938
940	MGCS36044_02376			*yhcF*	YhcF family transcriptional regulator	221.5	938
941	MGCS36044_00414			*pgpA*	phosphatidylglycerophosphatase protein PgpA	221.3	941
942	MGCS36044_01912	Secreted		*dacA_3*	secreted D,D-carboxypeptidase penicillin-binding	220.8	942
943	MGCS36044_00394			*pflC*	pyruvate formate-lyase activating enzyme PflC	219.3	943

No.	Locus tag	SignalP6 predicted(1)	Virulence(2)	Gene	Function	RPKMs(3)	RANK(4)
944	MGCS36044_01740			-	hypothetical protein	219.3	943
945	MGCS36044_01024			-	hypothetical protein	219.0	945
946	MGCS36044_02138			-	hypothetical protein	216.8	946
947	MGCS36044_01662			*gorA*	glutathione reductase GorA	216.5	947
948	MGCS36044_02412			*ung*	uracil-DNA glycosylase Ung	216.0	948
949	MGCS36044_02502			-	CRISPR-DR22 RNA	214.5	949
950	MGCS36044_02210			-	DUF1836 domain-containing protein	213.5	950
951	MGCS36044_02408			*plsY*	glycerol-3-phosphate 1-O-acyltransferase PlsY	213.5	950
952	MGCS36044_03498			-	SSRC34_2 RNA	213.5	950
953	MGCS36044_02142			-	helix-turn-helix transcriptional regulator	213.3	953
954	MGCS36044_02282			*deoA*	pyrimidine-nucleoside phosphorylase DeoA	213.3	953
955	MGCS36044_03026			*feoB*	ferrous iron transport protein (B) FeoB	212.8	955
956	MGCS36044_03416			-	TetR/AcrR family transcriptional regulator	212.5	956
957	MGCS36044_04256			*trpS*	tryptophanyl-tRNA synthetase	211.5	957
958	MGCS36044_00492			*prmA*	50S ribosomal protein L11 methyltransferase	211.3	958
959	MGCS36044_02630			-	C4-dicarboxylate ABC transporter	210.8	959
960	MGCS36044_03642			*comEB*	competence protein ComEB	210.8	959
961	MGCS36044_00870			*rsmG*	16S rRNA (guanine(527)-N(7))-methyltransferase	210.3	961
962	MGCS36044_02522			*fatA*	FatA family acyl-[acyl-carrier-protein]	210.3	961
963	MGCS36044_00486			*pabA*	aminodeoxychorismate/anthranilate synthase	209.8	963
964	MGCS36044_04258			*yitT*	membrane anchor protein YitT	209.8	963
965	MGCS36044_02322			-	aromatic acid exporter family protein	209.3	965
966	MGCS36044_02948			-	GIY-YIG catalytic domain-containing putative	209.0	966
967	MGCS36044_01386			-	hypothetical protein	208.8	967
968	MGCS36044_00288			*nox*	NAD(P)/FAD-dependent oxidoreductase Nox	208.0	968
969	MGCS36044_01684			*lysR*	LysR family transcriptional regulator	207.3	969
970	MGCS36044_01982			-	Na+ driven multidrug efflux pump	207.0	970
971	MGCS36044_03892			-	ABC transporter ATPase/permease	206.8	971
972	MGCS36044_00958	Lipo		*fhuD*	iron-hydroxamate ABC transporter	206.3	972
973	MGCS36044_01984			*opuBA*	OpuBA superfamily glycine/betaine ABC	205.3	973
974	MGCS36044_02076	Lipo		*dppA*	dipeptide-binding ABC transport system	204.8	974
975	MGCS36044_02066			-	hypothetical protein	204.5	975
976	MGCS36044_02890			*nrdH*	glutaredoxin-like protein NrdH	203.8	976
977	MGCS36044_03600			-	ECF transporter (S) specificity component	203.8	976
978	MGCS36044_00496			-	LacI family DNA-binding transcriptional	203.0	978
979	MGCS36044_01658			*aroC*	chorismate synthase AroC	202.8	979
980	MGCS36044_01096			*mycA*	oleate hydratase/myosin-crossreactive antigen	202.5	980
981	MGCS36044_02506			-	CRISPR-DR22 RNA	202.5	980
982	MGCS36044_04220			-	Spd-sr37 RNA	202.3	982
983	MGCS36044_02132			*mngR*	MngR family DNA-binding transcriptional	202.0	983
984	MGCS36044_01378			-	HD domain-containing phosphohydrolase	201.8	984
985	MGCS36044_01668			*nifS_1*	cysteine desulfurase NifS	201.5	985
986	MGCS36044_01448			*pepD_1*	C69 family dipeptidase PepD	201.3	986

No.	Locus tag	SignalP6 predicted[(1)]	Virulence[(2)]	Gene	Function	RPKMs[(3)]	RANK[(4)]
987	MGCS36044_01546			*glgD*	glucose-1-phosphate adenylyltransferase subunit GlgD	201.0	987
988	MGCS36044_02260			*lipL*	lipoate--protein ligase	201.0	987
989	MGCS36044_01162			*mutM*	DNA-formamidopyrimidine glycosylase MutM	199.8	989
990	MGCS36044_01760			-	GTP pyrophosphokinase family protein	199.8	989
991	MGCS36044_02684			-	hypothetical protein	199.8	989
992	MGCS36044_01910			*xerD_2*	site-specific integrase	199.3	992
993	MGCS36044_02972			*pbp2B*	penicillin-binding protein PBP2B/FtsI	199.0	993
994	MGCS36044_02144			*yhhX*	PRK10206 superfamily putative oxidoreductase. Region of difference 36044_ROD.6, putative MGE	198.8	994
995	MGCS36044_02854			*fadR*	FadR family DNA-binding transcriptional	198.5	995
996	MGCS36044_03942			-	DUF975 family protein	198.3	996
997	MGCS36044_02310			-	inositol monophosphatase family protein	198.0	997
998	MGCS36044_00488			*pabB*	para-aminobenzoate synthetase	197.8	998
999	MGCS36044_02458			-	NAD-dependent succinate-semialdehyde	197.3	999
1000	MGCS36044_03036			*clpA*	ATP-dependent Clp protease ATP-binding subunit ClpA	197.3	999
1001	MGCS36044_00510			-	PreQ1	196.3	1001
1002	MGCS36044_01164			*coaE*	dephospho-CoA kinase CoaE	195.8	1002
1003	MGCS36044_00396			-	GlpR-like transcriptional regulator protein	195.5	1003
1004	MGCS36044_02868			*birA*	bifunctional biotin--[acetyl-CoA-carboxylase]	195.0	1004
1005	MGCS36044_02896			*clcB*	voltage-gated ClC-type chloride channel ClcB	194.8	1005
1006	MGCS36044_02030			-	vicinal oxygen chelate (VOC) family protein	194.0	1006
1007	MGCS36044_00734			-	mechanosensitive ion channel family protein	193.8	1007
1008	MGCS36044_01756			*fms*	peptide deformylase	193.8	1007
1009	MGCS36044_02562			*phnK*	PhnK family ABC transporter ATPase component	193.8	1007
1010	MGCS36044_03002	Secreted		-	extracellular cell surface putative nucleotidase	193.8	1007
1011	MGCS36044_03870		Virulence	*fasC*	TCS histidine kinase	193.8	1007
1012	MGCS36044_04024			*ctsR*	CtsR family transcriptional regulator	193.5	1012
1013	MGCS36044_03802			-	YgaE family protein	193.3	1013
1014	MGCS36044_00784			*sufC*	Fe-S cluster assembly ATPase SufC	192.5	1014
1015	MGCS36044_01504			-	DUF1694 domain-containing protein	192.5	1014
1016	MGCS36044_02602			*dadA*	FAD-binding oxidoreductase DadA	192.3	1016
1017	MGCS36044_03782			*tsaB*	tRNA (adenosine(37)-N6)-threonylcarbamoyltransferase complex dimerization subunit type 1 TsaB	192.3	1016
1018	MGCS36044_01488			*clcA*	voltage-gated chloride channel family protein	192.0	1018
1019	MGCS36044_01700			*carA*	carbamoyl-phosphate synthase small subunit CarA	192.0	1018
1020	MGCS36044_04244			*pqqF*	pitrilysin family predicted Zn-dependent	191.8	1020
1021	MGCS36044_03772			*yhiN*	YhiN family predicted flavoprotein	191.5	1021
1022	MGCS36044_02500			-	CRISPR-DR22 RNA	191.3	1022
1023	MGCS36044_01090			*msrA*	peptide-methionine (S)-S-oxide reductase MsrA	190.8	1023
1024	MGCS36044_03906			-	5-formyltetrahydrofolate cyclo-ligase	190.5	1024
1025	MGCS36044_01954			-	AI-2E family transporter	190.3	1025
1026	MGCS36044_01948			*eriC*	EriC family voltage gated chloride channel	190.0	1026
1027	MGCS36044_02312			-	UPF0223 family protein	190.0	1026
1028	MGCS36044_03008			*tlyA*	TlyA family RNA methyltransferase	188.8	1028

No.	Locus tag	SignalP6 predicted[(1)]	Virulence[(2)]	Gene	Function	RPKMs[(3)]	RANK[(4)]
1029	MGCS36044_02836			*spxR*	SpxR family CBS-HotDog domain-containing	188.3	1029
1030	MGCS36044_03496			*cof*	Cof family hydrolase	188.0	1030
1031	MGCS36044_03930	Lipo		-	CYK3 family lipoprotein putatively involved in cell division and chromosome partitioning	187.8	1031
1032	MGCS36044_00988			*tmk*	thymidylate kinase Tmk	187.0	1032
1033	MGCS36044_01002			*serC*	3-phosphoserine/phosphohydroxythreonine	185.8	1033
1034	MGCS36044_02134			*ylxM*	YlxM superfamily signal recognition particle	185.8	1033
1035	MGCS36044_01914			-	polysaccharide deacetylase family protein	184.0	1035
1036	MGCS36044_03436			*glpR*	GlpR family DNA-binding transcriptional	184.0	1035
1037	MGCS36044_00840			-	nucleotidyltransferase	183.8	1037
1038	MGCS36044_01278			*pnpR*	phosphate TCS signal transduction response	183.8	1037
1039	MGCS36044_02324			*hsdS*	type I restriction endonuclease subunit S	183.3	1039
1040	MGCS36044_01588			*rexB*	ATP-dependent nuclease B subunit RexB	183.0	1040
1041	MGCS36044_01698			*pyrB*	aspartate transcarbomoylase PyrB	183.0	1040
1042	MGCS36044_01916			*hom*	homoserine dehydrogenase Hom	182.8	1042
1043	MGCS36044_03558			-	drug/metabolite transporter superfamily protein	182.3	1043
1044	MGCS36044_01766			*mvaK1*	mevalonate kinase MvaK1	182.0	1044
1045	MGCS36044_02524			*hemN*	HemN family coproporphyrinogen III oxidase or	181.3	1045
1046	MGCS36044_02720			*fetB*	iron export ABC transporter permease subunit	181.0	1046
1047	MGCS36044_03138			-	MmcQ/YjbR family DNA-binding protein	179.8	1047
1048	MGCS36044_01000			*cutC*	copper homeostasis protein CutC	179.5	1048
1049	MGCS36044_02460			*yjjG*	YjjG family noncanonical pyrimidine	179.5	1048
1050	MGCS36044_02362			*phnA*	PnhA family zinc ribbon domain-containing	178.0	1050
1051	MGCS36044_01696			*pyrP*	uracil permease protein PyrP	177.0	1051
1052	MGCS36044_02238			*cls*	cardiolipin synthase	177.0	1051
1053	MGCS36044_00018			-	RNA-binding S4 domain-containing protein	176.8	1053
1054	MGCS36044_01314			-	YdbC family protein	176.5	1054
1055	MGCS36044_03006			*argR_1*	ArgR family transcriptional regulator	176.0	1055
1056	MGCS36044_01092			*yozE*	YozE family protein	175.8	1056
1057	MGCS36044_01544			*glgC*	glucose-1-phosphate adenylyltransferase subunit	175.5	1057
1058	MGCS36044_01724			*trxB_1*	NAD(P)/FAD-dependent oxidoreductase	175.5	1057
1059	MGCS36044_00388			-	MGCS36044_00388	175.3	1059
1060	MGCS36044_04166			*pezT*	zeta toxin family protein PezT	175.0	1060
1061	MGCS36044_01752			-	MdlB family multidrug ABC transporter ATPase and permease component	174.0	1061
1062	MGCS36044_00948			*pflA*	pyruvate formate-lyase activating enzyme PflA	173.8	1062
1063	MGCS36044_02250			*lplA_1*	lipoate--protein ligase	173.0	1063
1064	MGCS36044_03024			-	FeoB associated cysteine-rich protein	172.5	1064
1065	MGCS36044_03076			-	DUF910 domain-containing protein	172.5	1064
1066	MGCS36044_00426			-	hypothetical protein	171.5	1066
1067	MGCS36044_00904			*acyP*	acylphosphatase AcyP	171.3	1067
1068	MGCS36044_04122			*mutS*	DNA mismatch repair protein MutS	171.0	1068
1069	MGCS36044_02454			*uvrC*	excinuclease ABC subunit UvrC	170.5	1069
1070	MGCS36044_03904			*glpG*	GlpG family membrane associated serine protease	170.5	1069
1071	MGCS36044_03010			*ispA*	IspA family geranylgeranyl pyrophosphate	170.3	1071

No.	Locus tag	SignalP6 predicted[(1)]	Virulence[(2)]	Gene	Function	RPKMs[(3)]	RANK[(4)]
1072	MGCS36044_00506			-	ECF transporter S component	170.0	1072
1073	MGCS36044_00622			-	DNA cytosine methyltransferase	169.5	1073
1074	MGCS36044_01052			-	UhpC family MFS transporter	169.3	1074
1075	MGCS36044_01996			-	thiamine biosynthesis protein ApbE-like	168.8	1075
1076	MGCS36044_00428			-	FAD/NAD(P)-binding protein	168.5	1076
1077	MGCS36044_00808			-	IS30 family transposase	168.5	1076
1078	MGCS36044_01196			*amyS*	alpha-amylase AmyS	168.0	1078
1079	MGCS36044_02252			-	NAD-dependent deacetylase	168.0	1078
1080	MGCS36044_03794			*oppB_2*	oligopeptide ABC transporter permease OppB	167.8	1080
1081	MGCS36044_03728			-	ABC transporter permease	167.5	1081
1082	MGCS36044_03438			*copZ_2*	copper chaperone CopZ	166.8	1082
1083	MGCS36044_00424			-	putative transcriptional regulator	166.5	1083
1084	MGCS36044_02554			-	ABC transporter ATP-binding protein	166.3	1084
1085	MGCS36044_03872		Virulence	*fasB*	TCS histidine kinase	166.3	1084
1086	MGCS36044_00776	Secreted		-	DUF2207 domain-containing secreted protein	166.0	1086
1087	MGCS36044_02056	Lipo		-	TlpA_family protein	166.0	1086
1088	MGCS36044_02320			-	DUF2130 domain-containing protein	166.0	1086
1089	MGCS36044_02874			*prsW*	PrsW family glutamic-type intramembrane	165.8	1089
1090	MGCS36044_03894			-	ABC transporter ATPase/permease	165.3	1090
1091	MGCS36044_02540			-	DUF4097 family beta strand repeat-containing	165.0	1091
1092	MGCS36044_00398			-	DeoR-like transcriptional regulator protein	164.8	1092
1093	MGCS36044_00732			-	DUF436 family protein	163.8	1093
1094	MGCS36044_00658			-	type II toxin-antitoxin system Phd/YefM family	163.5	1094
1095	MGCS36044_04040			*hutI*	imidazolonepropionase HutI	163.0	1095
1096	MGCS36044_03836			*tatD*	Tat protein secretion system quality control	162.8	1096
1097	MGCS36044_02088			-	site-specific integrase. Region of difference 36044_ROD.5, putative MGE	162.3	1097
1098	MGCS36044_04164			-	site-specific integrase	162.3	1097
1099	MGCS36044_04168			-	helix-turn-helix domain-containing	162.0	1099
1100	MGCS36044_02254			-	protein-ADP-ribose hydrolase	160.0	1100
1101	MGCS36044_01702			*carB*	carbamoyl-phosphate synthase large subunit CarB	159.8	1101
1102	MGCS36044_02258			-	NADH-dependent flavin oxidoreductase	159.5	1102
1103	MGCS36044_03306			*fmt*	methionyl-tRNA formyl transferase Fmt	159.5	1102
1104	MGCS36044_01972			-	GTP pyrophosphokinase family protein	159.3	1104
1105	MGCS36044_03012			*xseB*	exodeoxyribonuclease VII small subunit XseB	159.0	1105
1106	MGCS36044_02374			*dnaE*	DNA polymerase III subunit alpha DnaE	158.0	1106
1107	MGCS36044_00532			*dexB*	glucan 1,6-alpha-glucosidase DexB	157.5	1107
1108	MGCS36044_01014			*exoA*	exodeoxyribonuclease III protein ExoA	157.5	1107
1109	MGCS36044_01800			-	Possible RNA. L10_leader	157.5	1107
1110	MGCS36044_04246			*pqqL*	pitrilysin family predicted Zn-dependent peptidase	157.5	1107
1111	MGCS36044_03150			-	CorA family divalent cation transport protein	157.3	1111
1112	MGCS36044_02180			-	PASTA domain-containing protein	157.0	1112
1113	MGCS36044_02876			-	DUF1294 domain-containing protein	156.5	1113
1114	MGCS36044_02308			*rsmF*	RsmF family rRNA cytosine-C5-methyltransferase	156.3	1114

No.	Locus tag	SignalP6 predicted[(1)]	Virulence[(2)]	Gene	Function	RPKMs[(3)]	RANK[(4)]
1115	MGCS36044_03644			*pepP*	PepP family Xaa-Pro peptidase	156.3	1114
1116	MGCS36044_04272			*rlmH*	23S rRNA (pseudouridine(1915)-N(3))-methyltransferase RlmH	156.3	1114
1117	MGCS36044_00508			*rihB*	pyrimidine-specific ribonucleoside hydrolase	156.0	1117
1118	MGCS36044_03730			-	ABC transporter ATP-binding protein	156.0	1117
1119	MGCS36044_01036			*brnQ_2*	branched-chain amino acid transport system II carrier protein BrnQ	155.5	1119
1120	MGCS36044_02492			-	RnaY family phopsphodiesterase	155.3	1120
1121	MGCS36044_03298		Virulence	*liaF*	three component system signal transduction membrane component protein	155.3	1120
1122	MGCS36044_00838			-	class I SAM-dependent methyltransferase	155.0	1122
1123	MGCS36044_01380			-	DUF1934 domain-containing protein	154.8	1123
1124	MGCS36044_03766			-	MerR/SoxR family transcriptional regulator	154.3	1124
1125	MGCS36044_03676			*recD*	ATP-dependent DNA helicase RecD	153.8	1125
1126	MGCS36044_02830			*aroA*	3-phosphoshikimate 1-carboxyvinyltransferase	152.8	1126
1127	MGCS36044_02016			-	MdlB superfamily multidrug ABC transporter	152.3	1127
1128	MGCS36044_02828			*aroK*	shikimate kinase AroK	152.0	1128
1129	MGCS36044_03200			-	permease	151.5	1129
1130	MGCS36044_02416	Lipo		-	amino-acid ABC transporter substrate-binding	151.0	1130
1131	MGCS36044_01370			-	FMN-binding domain containing L-lactate oxidase	150.8	1131
1132	MGCS36044_03702			-	putative sulfite exporter	150.5	1132
1133	MGCS36044_01042			-	DUF2829 domain-containing protein	150.0	1133
1134	MGCS36044_02386			-	DUF3862 domain-containing lipoprotein	149.5	1134
1135	MGCS36044_01224			*paaJ*	acetyl-CoA acetyl transferase PaaJ	149.0	1135
1136	MGCS36044_02712			*rsuA_1*	ribosomal small subunit pseudouridine synthase	148.5	1136
1137	MGCS36044_01420			-	HAD family phosphatase	148.0	1137
1138	MGCS36044_00490			-	DUF3013 family protein	147.8	1138
1139	MGCS36044_01434			*dinG*	bifunctional DnaQ family exonuclease --	147.5	1139
1140	MGCS36044_02918			-	NAD(P)H-dependent oxidoreductase	147.3	1140
1141	MGCS36044_00162			-	MATE family multidrug efflux transporter	147.0	1141
1142	MGCS36044_00944			-	RsmB/NOP family class I SAM-dependent RNA methyltransferase	145.8	1142
1143	MGCS36044_01720			*rimM*	ribosome maturation factor RimM	145.5	1143
1144	MGCS36044_03724	Secreted		-	putative secreted protein	145.5	1143
1145	MGCS36044_00728			*thiD*	bifunctional hydroxymethylpyrimidine	144.3	1145
1146	MGCS36044_00670			-	hypothetical protein	143.8	1146
1147	MGCS36044_00730			-	ECF transporter S component	143.8	1146
1148	MGCS36044_03884			-	carbonic anhydrase	142.5	1148
1149	MGCS36044_00450			-	hypothetical protein	142.3	1149
1150	MGCS36044_00602			-	L13_leader	142.3	1149
1151	MGCS36044_01750			-	MdlB family multidrug ABC transporter ATPase and	141.8	1151
1152	MGCS36044_03140		Virulence	*yesM*	TCS sensor kinase YesM	141.5	1152
1153	MGCS36044_00382			*ridA*	RidA family protein	141.0	1153
1154	MGCS36044_02564			-	ABC transporter permease component	141.0	1153
1155	MGCS36044_00008			-	helix-turn-helix domain-containing protein	140.0	1155
1156	MGCS36044_01694			*pyrR*	bifunctional pyrimidine regulatory	140.0	1155
1157	MGCS36044_01894			-	integrase catalytic subunit	140.0	1155

No.	Locus tag	SignalP6 predicted[(1)]	Virulence[(2)]	Gene	Function	RPKMs[(3)]	RANK[(4)]
1158	MGCS36044_02018			-	MdlB superfamily multidrug ABC transporter ATP-binding protein	139.8	1158
1159	MGCS36044_02068			*dppF*	dipeptide ABC transport system ATP-binding DppF	139.3	1159
1160	MGCS36044_03132			*fnr*	Crp/Fnr family transcriptional regulator	139.0	1160
1161	MGCS36044_03938			-	CHY zinc finger domain-containing protein	138.0	1161
1162	MGCS36044_02808			-	TetR/AcrR family transcriptional regulator	137.5	1162
1163	MGCS36044_00034			-	IS30 family transposase	137.3	1163
1164	MGCS36044_01882			*copB*	copper-exporting ATPase cCopB	137.3	1163
1165	MGCS36044_02656			*malH*	LacI family transcriptional regulatory protein	137.0	1165
1166	MGCS36044_03732			-	PLDc N-terminal domain-containing protein	137.0	1165
1167	MGCS36044_01226			*ppsB*	long-chain fatty acid--CoA ligase PpsB	136.8	1167
1168	MGCS36044_01330			*thiT*	energy-coupled thiamine transporter ThiT	136.5	1168
1169	MGCS36044_01542			*glgB*	1,4-alpha-glucan branching protein GlgB	136.0	1169
1170	MGCS36044_03736			-	MFS transporter	135.5	1170
1171	MGCS36044_02130			-	hypothetical protein	135.3	1171
1172	MGCS36044_01672			*capA*	CapA family protein	134.5	1172
1173	MGCS36044_00296			*cdyC*	thiol reductant ABC exporter subunit CydC	134.3	1173
1174	MGCS36044_02382			-	TVP38/TMEM64 family protein	134.3	1173
1175	MGCS36044_00108			*recO*	DNA repair protein RecO	134.0	1175
1176	MGCS36044_03780			*rimI*	ribosomal-protein-alanine N-acetyltransferase	134.0	1175
1177	MGCS36044_00286			-	1,4-dihydroxy-2-naphthoate octaprenyltransferase	133.8	1177
1178	MGCS36044_04262			*yfhO*	YfhO family protein	133.5	1178
1179	MGCS36044_01892			*cadA*	cadmium-translocating P-type ATPase CadA	133.0	1179
1180	MGCS36044_02350			*alsT*	sodium:alanine symporter family protein	133.0	1179
1181	MGCS36044_02498			*ndk*	nucleoside-diphosphate kinase Ndk. Followed by a CRISPR with 5 repeat units	132.0	1181
1182	MGCS36044_03586			*uspA*	UspA family nucleotide-binding universal stress	131.8	1182
1183	MGCS36044_00342			-	hypothetical protein	131.5	1183
1184	MGCS36044_02838			-	GNAT family N-acetyltransferase	131.3	1184
1185	MGCS36044_00550			-	nucleotidyltransferase family protein	131.0	1185
1186	MGCS36044_03014			*xseA*	exodeoxyribonuclease VII large subunit XseA	131.0	1185
1187	MGCS36044_00158			-	hypothetical protein	130.8	1187
1188	MGCS36044_01970			*yjbK*	YbjK superfamily CYTH domain-containing	130.8	1187
1189	MGCS36044_03890			*queH*	epoxyqueuosine reductase QueH	130.8	1187
1190	MGCS36044_00954			*fhuG*	iron ABC transporter permease FhuG	130.0	1190
1191	MGCS36044_01016			*sdpI*	SdpI family immunity protein	130.0	1190
1192	MGCS36044_03734			-	Cro/CI family transcriptional regulator	129.8	1192
1193	MGCS36044_03412			-	hypothetical protein	129.5	1193
1194	MGCS36044_02722			*fetA*	iron export ABC transporter ATP-binding subunit	129.3	1194
1195	MGCS36044_02052	Secreted	Virulence	*isp*	secreted CHAP domain-containing immunogenic	129.0	1195
1196	MGCS36044_02462			-	DUF1524 domain-containing protein	128.8	1196
1197	MGCS36044_02494			*msrB*	peptide-methionine (R)-S-oxide reductase MsrB	128.8	1196
1198	MGCS36044_01494			*rodA*	rod shape-determining protein RodA	128.5	1198
1199	MGCS36044_03248			*trmA*	TrmA RNA methyltransferase	128.3	1199
1200	MGCS36044_03726			-	GNAT family acetyltransferase	128.3	1199

No.	Locus tag	SignalP6 predicted[(1)]	Virulence[(2)]	Gene	Function	RPKMs[(3)]	RANK[(4)]
1201	MGCS36044_04148			-	cadmium efflux system accessory protein	128.3	1199
1202	MGCS36044_03680			*dinB*	DNA polymerase IV DinB	128.0	1202
1203	MGCS36044_04012			-	hypothetical protein	128.0	1202
1204	MGCS36044_00298			*preA*	polyprenyl synthetase family protein PreA	127.5	1204
1205	MGCS36044_03840			*rpiR*	RpiR family transcriptional regulator	127.5	1204
1206	MGCS36044_03028			*feoA*	ferrous iron transport protein (A) FeoA	126.8	1206
1207	MGCS36044_00294			*cydD*	thiol reductant ABC exporter subunit CydD	126.3	1207
1208	MGCS36044_00528			*lrp*	PucR family transcriptional regulator/leucine	126.3	1207
1209	MGCS36044_03202			*aroE*	shikimate dehydrogenase AroE	126.3	1207
1210	MGCS36044_02468			*ykgJ*	YkgJ family cysteine cluster protein	126.0	1210
1211	MGCS36044_03152			-	CRISPR with 9 repeat units	125.8	1211
1212	MGCS36044_02924			-	MFS transporter	125.5	1212
1213	MGCS36044_03410			*nptA*	NtpA family Na/Pi cotransporter	125.5	1212
1214	MGCS36044_00016			-	oligosaccharide flippase family protein	125.3	1214
1215	MGCS36044_01582			-	ABC transporter permease	125.3	1214
1216	MGCS36044_02054			-	hypothetical protein	125.0	1216
1217	MGCS36044_01736			-	putative peptidoglycan hydrolase	124.8	1217
1218	MGCS36044_01584			-	ABC transporter ATP-binding component LolD-like	124.5	1218
1219	MGCS36044_02654			*malG*	maltose/maltodextrin ABC transport system permease protein MalG	124.5	1218
1220	MGCS36044_02422			-	GNAT family N-acetyltransferase	124.0	1220
1221	MGCS36044_00156			*adhP*	alcohol dehydrogenase AdhP	123.8	1221
1222	MGCS36044_02628			-	DUF3042 family protein	123.8	1221
1223	MGCS36044_01472			-	IS30 family transposase	123.5	1223
1224	MGCS36044_02584			*yloA*	YloA family predicted ribosome quality control	123.0	1224
1225	MGCS36044_04056			*hutG*	formiminoglutamase HutG	123.0	1224
1226	MGCS36044_01410	Secreted	Virulence	*hylB*	secreted hyaluronate lyase HylB	122.8	1226
1227	MGCS36044_03250			*recX*	recombination regulator RecX	122.8	1226
1228	MGCS36044_02934	Lipo		*blaA*	beta-lactamase-related serine hydrolase	122.5	1228
1229	MGCS36044_02420			-	IS3 family transposase	122.3	1229
1230	MGCS36044_03204			*lacZ*	beta-galactosidase LacZ	122.3	1229
1231	MGCS36044_03408			*nagA*	N-acetylglucosamine-6-phosphate deacetylase	122.3	1229
1232	MGCS36044_02466			*ycjU*	YcjU family beta-phosphoglucomutase or related	121.3	1232
1233	MGCS36044_02566			-	ABC transporter substrate binding component	121.3	1232
1234	MGCS36044_02188			-	DUF3307 domain-containing protein	120.8	1234
1235	MGCS36044_02594			-	Abi family CAAX protease self-immunity protein	120.5	1235
1236	MGCS36044_03442			*copY_2*	DNA-binding copper transport transcriptional repressor CopY	120.5	1235
1237	MGCS36044_00956			*fhuB*	iron ABC transporter permease FhuB	120.3	1237
1238	MGCS36044_01586			-	neutral zinc metallopeptidase	120.0	1238
1239	MGCS36044_00942			*yhcC*	YhcC family Fe-S oxidoreductase	119.8	1239
1240	MGCS36044_01458			-	GntR family transcriptional regulator	119.8	1239
1241	MGCS36044_01780			-	HTH domain-containing putative transcriptional regulator	119.5	1241
1242	MGCS36044_02542			-	PadR family transcriptional regulator	119.5	1241
1243	MGCS36044_03278			-	IS30 family transposase	119.5	1241

No.	Locus tag	SignalP6 predicted[(1)]	Virulence[(2)]	Gene	Function	RPKMs[(3)]	RANK[(4)]
1244	MGCS36044_02164			-	polyprenyl synthetase family protein	119.0	1244
1245	MGCS36044_01754			*gdhA*	NADP-specific glutamate dehydrogenase GdhA	118.0	1245
1246	MGCS36044_02414			-	amino acid ABC transporter permease	117.8	1246
1247	MGCS36044_03308			*priA*	primosomal protein PriA	117.8	1246
1248	MGCS36044_00938			-	ECF transporter S component	117.3	1248
1249	MGCS36044_04150			-	DNA-binding HTH domain-containing	117.3	1248
1250	MGCS36044_02464			-	maltose/galactose O-acetyltransferase	117.0	1250
1251	MGCS36044_00906			-	SpoU family RNA methyltransferase	116.8	1251
1252	MGCS36044_00934			*trml*	TrmL family tRNA (cytosine34-2'-O-)-methyl	116.8	1251
1253	MGCS36044_01868			-	hypothetical protein	116.8	1251
1254	MGCS36044_04186			-	thioredoxin family protein	116.8	1251
1255	MGCS36044_00672	Pilin		-	putative lipoprotein	114.8	1255
1256	MGCS36044_02898			-	CPBP family intramembrane metalloprotease	114.8	1255
1257	MGCS36044_03184			-	MOP/MATE family multidrug-resistance efflux	114.5	1257
1258	MGCS36044_03634			*scrR*	sucrose operon repressor ScrR	114.3	1258
1259	MGCS36044_03590			*asnB*	L-asparaginase AsnB	113.0	1259
1260	MGCS36044_01896			-	DUF4365 family protein	112.8	1260
1261	MGCS36044_02284			*rsmC*	class I SAM-dependent methyltransferase	112.8	1260
1262	MGCS36044_02352			*fieF*	FieF family cation diffusion facilitator family	112.3	1262
1263	MGCS36044_02488			*spiA*	sakacin P immunity protein SpiA	111.8	1263
1264	MGCS36044_00538			-	thioredoxin family protein	111.5	1264
1265	MGCS36044_02048		Virulence	*irr*	TCS signal transduction DNA-binding response	111.3	1265
1266	MGCS36044_04146			*cadD*	CadD family cadmium resistance transporter	111.3	1265
1267	MGCS36044_03210			*trxT*	Trx TCS operon protein TrxT	111.0	1267
1268	MGCS36044_02050		Virulence	*ihk*	TCS signal transduction histidine kinase sensor	110.8	1268
1269	MGCS36044_03244			*thlA*	thiolase ThlA	110.8	1268
1270	MGCS36044_03720			-	BaeS family TCS sensor histidine kinase	110.0	1270
1271	MGCS36044_00738			-	alpha-amylase family glycosyl hydrolase	109.8	1271
1272	MGCS36044_02432			-	LysR family transcriptional regulator	109.8	1271
1273	MGCS36044_02798			-	MdlB family multidrug ABC transporter ATPase and permease	109.3	1273
1274	MGCS36044_02974			*focA*	formate transporter FocA	109.3	1273
1275	MGCS36044_03206		Virulence	*trxR*	TCS DNA-binding response regulator protein TrxR	109.3	1273
1276	MGCS36044_04008			-	hypothetical protein	109.3	1273
1277	MGCS36044_02236			-	unknown	109.0	1277
1278	MGCS36044_01334			-	GH25 muramidase superfamily lysozyme	108.8	1278
1279	MGCS36044_02938			*holA*	DNA polymerase III delta subunit HolA	108.8	1278
1280	MGCS36044_02946			*trmN6*	tRNA1(Val) A37 N6-methylase TrmN6	108.8	1278
1281	MGCS36044_02070			*dppD*	dipeptide ABC transport system ATP-binding protein DppD	108.5	1281
1282	MGCS36044_01482			-	IS1548 family transposase	107.8	1282
1283	MGCS36044_02786			-	ORF6N domain-containing protein	107.5	1283
1284	MGCS36044_00014			*trcF*	transcription-repair coupling factor TcrF	107.3	1284
1285	MGCS36044_00598			-	helix-turn-helix transcriptional regulator	107.3	1284
1286	MGCS36044_03936			*bioY*	biotin transporter BioY	106.5	1286

No.	Locus tag	SignalP6 predicted(1)	Virulence(2)	Gene	Function	RPKMs(3)	RANK(4)
1287	MGCS36044_02652			*malF*	maltose/maltodextrin ABC transport system permease protein MalF	104.8	1287
1288	MGCS36044_03142		Virulence	*yesN*	TCS DNA-binding response regulator YesN	104.3	1288
1289	MGCS36044_03660			*mutY*	A/G-specific adenine glycosylase MutY	104.3	1288
1290	MGCS36044_04248			*ybcJ*	ribosome associated protein YbcJ	104.3	1288
1291	MGCS36044_00136			-	DUF4041 domain-containing protein	104.0	1291
1292	MGCS36044_03580			-	cysteine hydrolase	104.0	1291
1293	MGCS36044_03602			-	CydD-related ABC transporter ATPase/permease	104.0	1291
1294	MGCS36044_00524			-	ISAs1 family transposase	103.5	1294
1295	MGCS36044_02976			*niaX*	niacin transporter NiaX	103.5	1294
1296	MGCS36044_04180			-	hypothetical protein	103.5	1294
1297	MGCS36044_01266			*bglA*	6-phospho-beta-glucosidase BglA	103.3	1297
1298	MGCS36044_02978			*niaR*	niacin-responsive transcriptional repressor	103.3	1297
1299	MGCS36044_00610			-	helix-turn-helix transcriptional regulator	102.8	1299
1300	MGCS36044_02190			-	SatD family protein	102.8	1299
1301	MGCS36044_02234			*asd*	aspartate-semialdehyde dehydrogenase	102.3	1301
1302	MGCS36044_03494			*azgA*	AzgA family permease	102.3	1301
1303	MGCS36044_00608			-	site-specific integrase	102.0	1303
1304	MGCS36044_01594	Lipo		-	ABC transporter substrate-binding lipoprotein	102.0	1303
1305	MGCS36044_02072			*dppC*	dipeptide ABC transport system permease protein	102.0	1303
1306	MGCS36044_03042			-	hypothetical protein	101.8	1306
1307	MGCS36044_03510			-	acyltransferase family protein	101.8	1306
1308	MGCS36044_00138			-	hypothetical protein	101.5	1308
1309	MGCS36044_03768			*dnaQ*	DNA polymerase III epsilon subunit DnaQ	101.5	1308
1310	MGCS36044_01674			-	IS30 family transposase	101.3	1310
1311	MGCS36044_02724			*paaI*	PaaI family thioesterase	101.0	1311
1312	MGCS36044_01388			*regR*	transcriptional regulator RegR	100.8	1312
1313	MGCS36044_02714			-	putative thioesterase	100.8	1312
1314	MGCS36044_01206			-	ABC transporter permease component	100.5	1314
1315	MGCS36044_01222			-	acyl dehydratase	100.5	1314
1316	MGCS36044_00280			-	XRE family ImmR-like transcriptional regulator	100.3	1316
1317	MGCS36044_03320			*atoD*	butyrate-acetoacetate CoA-transferase alpha	100.0	1317
1318	MGCS36044_02912			-	OFA family MFS transporter	99.5	1318
1319	MGCS36044_03022			*phrB*	PhrB family deoxyribodipyrimidine photolyase	99.3	1319
1320	MGCS36044_02526			-	hypothetical protein	99.0	1320
1321	MGCS36044_03222			-	hypothetical protein	99.0	1320
1322	MGCS36044_02100			-	XRE family HTH-type transcriptional regulator	98.8	1322
1323	MGCS36044_04110			*cinA*	competence/damage-inducible protein CinA	98.8	1322
1324	MGCS36044_03902			-	DUF6198 family protein	98.5	1324
1325	MGCS36044_00762			-	IS1548 family transposase	98.3	1325
1326	MGCS36044_02166			-	NAD(P)/FAD-dependent oxidoreductase	98.0	1326
1327	MGCS36044_02926			*rsuA_2*	rsuA_2ribosomal small subunit pseudouridine synthase RsuA	97.8	1327
1328	MGCS36044_00842			-	MerR family transcriptional regulator	97.5	1328
1329	MGCS36044_02900			-	DUF3169 family protein	97.3	1329

No.	Locus tag	SignalP6 predicted[(1)]	Virulence[(2)]	Gene	Function	RPKMs[(3)]	RANK[(4)]
1330	MGCS36044_03610	Lipo		*htsA*	heme ABC transporter substrate-binding	97.3	1329
1331	MGCS36044_00440			-	hypothetical protein	96.8	1331
1332	MGCS36044_01350			-	RpoE superfamily DNA-directed RNA polymerase	96.8	1331
1333	MGCS36044_03322			*atoA*	acyl CoA:acetate/3-ketoacid CoA transferase beta	95.5	1333
1334	MGCS36044_02486			-	DUF2974 domain-containing protein	95.3	1334
1335	MGCS36044_03722			-	OmpR family TCS DNA-binding response regulator	95.0	1335
1336	MGCS36044_00656			-	Txe/YoeB family addiction module toxin	94.8	1336
1337	MGCS36044_01208			-	ABC transporter permease component	94.8	1336
1338	MGCS36044_01406			*kdgK*	2-dehydro-3-deoxygluconokinase KdgK	94.8	1336
1339	MGCS36044_03614	Secreted	Virulence	*shr*	heme-binding secreted protein Shr	94.8	1336
1340	MGCS36044_01332			-	Unknown	94.5	1340
1341	MGCS36044_02418			-	GatA family amidase	94.3	1341
1342	MGCS36044_00708			-	IS30 family transposase	94.0	1342
1343	MGCS36044_03208		Virulence	*trxS*	TCS sensor histidine kinase TrxS	93.8	1343
1344	MGCS36044_01340	Lipo		-	DUF4430 domain-containing lipoprotein	93.5	1344
1345	MGCS36044_03912			-	Mga-related helix-turn-helix domain-containing	93.5	1344
1346	MGCS36044_01244			*rnc*	ribonuclease III Rnc	93.3	1346
1347	MGCS36044_02286			*coaA*	type I pantothenate kinase	93.3	1346
1348	MGCS36044_03240			*caiC*	CaiC family Acyl-CoA synthetase	92.8	1348
1349	MGCS36044_02424			*pyrE*	orotate phosphoribosyltransferase PyrE	92.5	1349
1350	MGCS36044_03286			-	YIH1 family putative translation regulator	92.5	1349
1351	MGCS36044_00600			-	IS30 family transposase	92.3	1351
1352	MGCS36044_03696			-	PurR family transcriptional regulator	92.3	1351
1353	MGCS36044_04194			-	DUF4097 family protein	92.3	1351
1354	MGCS36044_00418			-	MccC family LD-carboxypeptidase	91.8	1354
1355	MGCS36044_01988			*guaC*	guanosine 5'-monophosphate oxidoreductase GuaC	91.8	1354
1356	MGCS36044_03878			-	hypothetical protein	91.8	1354
1357	MGCS36044_00706			-	Cof-type HAD-IIB family hydrolase	91.5	1357
1358	MGCS36044_00752			*lrgB*	antiholin-like protein LrgB	91.5	1357
1359	MGCS36044_01486			*pheA*	chorismate mutase PheA	91.5	1357
1360	MGCS36044_00596			-	hypothetical protein	91.0	1360
1361	MGCS36044_02702			-	hypothetical protein	90.8	1361
1362	MGCS36044_00348			-	DUF4651 domain-containing protein	90.5	1362
1363	MGCS36044_01478			*gstA*	GstA superfamily glutathione-dependent	90.5	1362
1364	MGCS36044_01412			-	hypothetical protein	90.3	1364
1365	MGCS36044_01408			*kgdA*	bifunctional 4-hydroxy-2-oxoglutarate (KHG)	89.5	1365
1366	MGCS36044_01530	Secreted		-	cell surface extracellular nuclease	89.3	1366
1367	MGCS36044_01870			-	XRE family HTH-type transcriptional regulator	89.3	1366
1368	MGCS36044_00726			*truA*	tRNA pseudouridine(38-40) synthase TruA	88.0	1368
1369	MGCS36044_02914			-	HAD family hydrolase	88.0	1368
1370	MGCS36044_03880			-	LytTR family transcriptional regulator	88.0	1368
1371	MGCS36044_01596			-	IS1548 family transposase	87.5	1371
1372	MGCS36044_03194	Lipo		-	putative lipoprotein	86.8	1372

No.	Locus tag	SignalP6 predicted(1)	Virulence(2)	Gene	Function	RPKMs(3)	RANK(4)
1373	MGCS36044_02638			-	hypothetical protein	86.3	1373
1374	MGCS36044_01580			*ybgA*	YbgA family DUF1722 domain-containing protein	85.8	1374
1375	MGCS36044_02074			*dppB*	dipeptide ABC transport system permease protein DppB	85.5	1375
1376	MGCS36044_02788			-	DUF1413 domain-cotaing protein	85.3	1376
1377	MGCS36044_01864			-	hypothetical protein	84.8	1377
1378	MGCS36044_03092			-	IS1548 family transposase	84.8	1377
1379	MGCS36044_04078			*catE*	catechol-2,3-dioxygenase CatE	84.8	1377
1380	MGCS36044_01404			*rpiB*	RpiB/LacA/LacB family sugar-phosphate isomerase	84.5	1380
1381	MGCS36044_04214			-	IS1548 family transposase	84.5	1380
1382	MGCS36044_02368			-	IS1548 family transposase	84.3	1382
1383	MGCS36044_03752			-	hypothetical protein	84.0	1383
1384	MGCS36044_01838			-	type IV toxin-antitoxin system AbiEi family	83.3	1384
1385	MGCS36044_02326			-	ABC transporter permease	83.3	1384
1386	MGCS36044_03326			*gntT*	GntT family H+/gluconate symporter or related	82.8	1386
1387	MGCS36044_03556			-	GlsB/YeaQ/YmgE family stress response membrane protein	82.8	1386
1388	MGCS36044_00750			*lrgA*	antiholin-like murein hydrolase modulator LrgA	82.3	1388
1389	MGCS36044_02626			*miaA*	tRNA (adenosine(37)-N6)-dimethylallyltransferase	82.3	1388
1390	MGCS36044_03566			*ppdK*	pyruvate phosphate dikinase PpdK	82.3	1388
1391	MGCS36044_00408			*mipB*	fructose-6-phosphate aldolase MipB	82.0	1391
1392	MGCS36044_01836			-	nucleotidyl transferase AbiEii/AbiGii toxin	82.0	1391
1393	MGCS36044_01250			-	IS1548 family transposase	81.5	1393
1394	MGCS36044_03318			*atoB*	3-ketoacyl-CoA thiolase/acetyl-CoA	81.3	1394
1395	MGCS36044_01130			-	IS1548 family transposase	81.0	1395
1396	MGCS36044_01204			-	ABC transporter ATP-binding component	81.0	1395
1397	MGCS36044_02740			-	resolvase/recombinase family protein	81.0	1395
1398	MGCS36044_00340			-	CAAX amino terminal protease family membrane	80.8	1398
1399	MGCS36044_02154			*xerS*	site-specific tyrosine recombinase XerS	80.8	1398
1400	MGCS36044_03712			-	Xre family helix-turn-helix transcriptional regulator	80.0	1400
1401	MGCS36044_01578			*osmC*	YhfA family OsmC-related salt-stress induced	79.8	1401
1402	MGCS36044_02426			*pyrF*	orotidine-5'-phosphate decarboxylase PyrF	79.5	1402
1403	MGCS36044_00412			-	hypothetical protein	79.3	1403
1404	MGCS36044_02470	Lipo		-	extracellular solute-binding lipoprotein	79.3	1403
1405	MGCS36044_03994	Secreted	Virulence	*slo*	secreted cholesterol-dependent cytolysin	78.8	1405
1406	MGCS36044_00460			-	helix-turn-helix transcriptional regulator	78.5	1406
1407	MGCS36044_03612	Secreted		*shp*	heme-binding secreted protein Shp	78.5	1406
1408	MGCS36044_03774			-	hypothetical protein	78.5	1406
1409	MGCS36044_00164			-	IS30 family transposase	78.3	1409
1410	MGCS36044_01878			*copA_1*	copper-exporting ATPase CopA	78.3	1409
1411	MGCS36044_02328			-	ABC transporter ATP-binding protein LolD-like	78.3	1409
1412	MGCS36044_01010			-	PhnB family glyoxalase/bleomycin	78.0	1412
1413	MGCS36044_01210			*dhaQ*	DhaKLM operon coactivator DhaQ	78.0	1412
1414	MGCS36044_03910			-	IS30 family transposase	78.0	1412
1415	MGCS36044_00856			*dapE*	M20/M25/M40 family metallo-hydrolase	77.8	1415

No.	Locus tag	SignalP6 predicted(1)	Virulence(2)	Gene	Function	RPKMs(3)	RANK(4)
1416	MGCS36044_00126			*purH*	bifunctional formyltransferase/IMP cyclohydrolase PurH	77.5	1416
1417	MGCS36044_00518	Secreted		-	cell surface UshA family bifunctional	77.3	1417
1418	MGCS36044_03608			*htsB*	heme ABC transporter permease HtsB	76.5	1418
1419	MGCS36044_00278			-	HNH endonuclease	76.3	1419
1420	MGCS36044_01900			-	MobC family plasmid mobilization relaxosome	76.0	1420
1421	MGCS36044_01880			*copZ_1*	copper chaperone CopZ	75.5	1421
1422	MGCS36044_00274			-	helix-turn-helix transcriptional regulator	74.5	1422
1423	MGCS36044_03578			-	DUF156 family protein	74.5	1422
1424	MGCS36044_02490			-	sakacin P family class II bacteriocin	73.8	1424
1425	MGCS36044_00228			-	MGCS36044_00228	72.8	1425
1426	MGCS36044_01940			*maeR*	TCS signal transduction response regulator MaeR	72.8	1425
1427	MGCS36044_02098			-	hypothetical protein	72.8	1425
1428	MGCS36044_02848			*uxuA*	mannonate dehydratase	72.3	1428
1429	MGCS36044_04192			-	DUF1700 domain-containing protein	72.0	1429
1430	MGCS36044_02158			-	1,4-dihydroxy-2-naphthoate polyprenyltransferase	71.8	1430
1431	MGCS36044_03126			*arcB*	ornithine carbamoyltransferase ArcB	71.8	1430
1432	MGCS36044_03604			-	CydC-related ABC transporter ATPase/permease	71.8	1430
1433	MGCS36044_03750			-	HTH cro/C1-type domain-containing protein	71.8	1430
1434	MGCS36044_01264			*bglF*	PTS beta-glucoside transporter IIBCA component	71.5	1434
1435	MGCS36044_04182			-	Rgg/GadR/MutR family transcriptional regulator	71.3	1435
1436	MGCS36044_00770			-	putative metal homeostasis protein	71.0	1436
1437	MGCS36044_01850			-	DUF5966 family protein	71.0	1436
1438	MGCS36044_03630			*scrA*	sucrose-specific PTS fused IIB/IIC/IIA component	71.0	1436
1439	MGCS36044_01220			*gla*	glycerol uptake facilitator protein Gla	70.8	1439
1440	MGCS36044_03896			-	MarR family transcriptional regulator	70.8	1439
1441	MGCS36044_02170			-	BaeS family TCS histidine kinase sensor	69.5	1441
1442	MGCS36044_02846			-	SDR family D-mannonate oxidoreductase	69.5	1441
1443	MGCS36044_03324			-	3-hydroxybutyrate dehydrogenase	69.5	1441
1444	MGCS36044_02632			-	glutathione S-transferase N-terminal	68.8	1444
1445	MGCS36044_01450	Secreted		*adcA*	zinc ABC transporter secreted substrate-binding	68.3	1445
1446	MGCS36044_02112			-	helix-turn-helix domain-containing protein	68.0	1446
1447	MGCS36044_03570			*ccpN*	CcpN family CBS pair domain transcriptional	68.0	1446
1448	MGCS36044_03978			-	IS30 family transposase	68.0	1446
1449	MGCS36044_00970			-	DUF4298 domain-containing protein	67.8	1449
1450	MGCS36044_03118			*arcC*	carbamate kinase ArcC	67.8	1449
1451	MGCS36044_02046			-	SalY superfamily ABC transporter permease	67.0	1451
1452	MGCS36044_02856			-	beta-D-glucuronidase	67.0	1451
1453	MGCS36044_02916			-	GNAT family N-acetyltransferase	66.8	1453
1454	MGCS36044_02110			-	hypothetical protein	66.5	1454
1455	MGCS36044_03128			-	GNAT family N-acetyltransferase	66.5	1454
1456	MGCS36044_04014	Secreted		*pepD_2*	secreted dipeptidase PepD	66.5	1454
1457	MGCS36044_00406			*pflD*	pyruvate formate-lyase protein PflD	66.0	1457
1458	MGCS36044_01218			*dhaM*	PTS-dependent dihydroxyacetone kinase	66.0	1457

No.	Locus tag	SignalP6 predicted(1)	Virulence(2)	Gene	Function	RPKMs(3)	RANK(4)
1459	MGCS36044_01440			-	DUF1003 domain-containing protein	65.8	1459
1460	MGCS36044_01824			*traG_1*	type IV secretory system conjugative DNA	65.8	1459
1461	MGCS36044_01452			-	GntR family transcriptional regulator	65.3	1461
1462	MGCS36044_02388			-	IS1182 family transposase	64.8	1462
1463	MGCS36044_04094			-	hypothetical protein	64.8	1462
1464	MGCS36044_01690			-	membrane-associated alkaline phosphatase	64.5	1464
1465	MGCS36044_01898			-	SAG1252 family conjugative relaxosome accessory	64.5	1464
1466	MGCS36044_03098	Lipo		*rbsB*	D-ribose ABC transporter substrate-binding	64.5	1464
1467	MGCS36044_00458			-	PhoE family phosphoglycerate mutase. Region of difference 36044_ROD.2, putative MGE	64.3	1467
1468	MGCS36044_01994			*pbuX*	xanthine permease PbuX	64.0	1468
1469	MGCS36044_02706			-	DUF4044 domain-containing protein	64.0	1468
1470	MGCS36044_02858			-	sugar kinase	63.8	1470
1471	MGCS36044_02640			-	NADPH-dependent FMN reductase	63.3	1471
1472	MGCS36044_03096			-	IS110 family transposase	63.3	1471
1473	MGCS36044_03182			-	MarR family winged helix-turn-helix	63.3	1471
1474	MGCS36044_02648			-	IS30 family transposase	62.8	1474
1475	MGCS36044_02806			-	ECF transporter S component	62.8	1474
1476	MGCS36044_03606			*htsC*	heme ABC transporter ATP-binding protein HtsC	62.5	1476
1477	MGCS36044_03120			-	IS110 family transposase	62.3	1477
1478	MGCS36044_01382			-	SSRC10 RNA	62.0	1478
1479	MGCS36044_02642			*glgP*	maltodextrin phosphorylase protein GlgP	62.0	1478
1480	MGCS36044_00012			*pth*	aminoacyl-tRNA hydrolase Pth	61.5	1480
1481	MGCS36044_02090			-	DUF3173 family protein	61.5	1480
1482	MGCS36044_02902			-	transcriptional regulator	61.5	1480
1483	MGCS36044_00668			-	Cro/CI family transcriptional regulator	61.0	1483
1484	MGCS36044_03568			-	kinase/pyrophosphorylase	61.0	1483
1485	MGCS36044_02108			-	hypothetical protein	60.8	1485
1486	MGCS36044_03574			-	CoA-disulfide reductase	60.5	1486
1487	MGCS36044_01336			-	transposase IS116/IS110/IS902 family protein	60.3	1487
1488	MGCS36044_02428			-	PyrR RNA	60.0	1488
1489	MGCS36044_03130			*arcA*	arginine deiminase ArcA	60.0	1488
1490	MGCS36044_03996			*ifs*	nicotine adenine dinucleotide glycohydrolase	59.8	1490
1491	MGCS36044_00630			-	ATP-binding protein	59.5	1491
1492	MGCS36044_02172			-	OmpR family TCS DNA-binding response regulator	59.5	1491
1493	MGCS36044_02330			*tetR*	TetR family transcriptional regulator	59.3	1493
1494	MGCS36044_01122			-	IS1182 family transposase	59.0	1494
1495	MGCS36044_03384			*fsa*	FSA family fructose-6-phosphate aldolase	59.0	1494
1496	MGCS36044_01890			*arsR*	cadmium efflux system metalloregulator ArsR/SmtB	58.5	1496
1497	MGCS36044_02726			-	nucleoside phosphorylase family protein	58.5	1496
1498	MGCS36044_03032			-	amino acid ABC transporter permease	58.3	1498
1499	MGCS36044_02472			-	YesN family TCS DNA-binding response regulator	58.0	1499
1500	MGCS36044_02982			-	IS982 family transposase	57.8	1500
1501	MGCS36044_02060			-	unknown	57.5	1501

No.	Locus tag	SignalP6 predicted(1)	Virulence(2)	Gene	Function	RPKMs(3)	RANK(4)
1502	MGCS36044_03030			-	amino acid ABC transporter ATP-binding protein	57.3	1502
1503	MGCS36044_01338			-	ECF transporter S component	57.0	1503
1504	MGCS36044_00448			-	radical SAM protein	56.8	1504
1505	MGCS36044_01848			-	DUF5962 family protein	56.5	1505
1506	MGCS36044_02182			*citC*	citrate lyase ligase CitC	56.5	1505
1507	MGCS36044_00422			*proX*	prolyl-tRNA synthetase associated	56.0	1507
1508	MGCS36044_00452			-	MdlB family ABC transporter ATP-binding/permease	56.0	1507
1509	MGCS36044_01990			-	unknown	56.0	1507
1510	MGCS36044_02354			-	IS1182 family transposase	56.0	1507
1511	MGCS36044_03188			-	hypothetical protein	56.0	1507
1512	MGCS36044_04178			-	IS982 family transposase	56.0	1507
1513	MGCS36044_01216			*dhaL*	dihydroxyacetone kinase subunit DhaL	55.8	1513
1514	MGCS36044_02850			*uxaC*	uronate isomerase	55.8	1513
1515	MGCS36044_01828			*trbL*	conjugal transfer protein TrbL	55.5	1515
1516	MGCS36044_04172			-	MFS transporter	55.5	1515
1517	MGCS36044_00438			-	bacteriocin immunity protein	55.0	1517
1518	MGCS36044_02928			*nagB*	glucosamine-6-phosphate deaminase NagB	55.0	1517
1519	MGCS36044_01902			-	SAG1250 family conjugative relaxase	54.3	1519
1520	MGCS36044_02058	Secreted	Virulence	*scpA*	cell surface extracellular C5a peptidase ScpA	53.8	1520
1521	MGCS36044_02804			*ecfT*	ECF transporter transmembrane protein EcfT	53.3	1521
1522	MGCS36044_03456		Virulence	-	YSIRK-targeted surface antigen transcriptional	53.3	1521
1523	MGCS36044_03748			-	hypothetical protein	53.0	1523
1524	MGCS36044_00724			-	aspartate kinase	52.8	1524
1525	MGCS36044_01502			*serB*	phosphoserine phosphatase SerB	52.8	1524
1526	MGCS36044_02160	Lipo		*apbE*	ApbE family thiamine biosynthesis lipoprotein	52.8	1524
1527	MGCS36044_02802			*ecfA2*	EcfA2 family ECF transporter ATPase	52.8	1524
1528	MGCS36044_00262			-	FRG domain-containing protein	52.5	1528
1529	MGCS36044_00628			-	SIR2 family protein	52.3	1529
1530	MGCS36044_04130			-	Uup family of ABC transporter with duplicated	52.3	1529
1531	MGCS36044_01862			-	DUF5960 family protein	52.0	1531
1532	MGCS36044_04072			-	Spy491738 RNA	52.0	1531
1533	MGCS36044_02508			*csn2*	CRISPR-associated protein Csn2	51.5	1533
1534	MGCS36044_03998	Secreted	Virulence	*nga*	secreted nicotine adenine dinucleotide	51.3	1534
1535	MGCS36044_01822			-	hypothetical protein	51.0	1535
1536	MGCS36044_01844			-	hypothetical protein	51.0	1535
1537	MGCS36044_03460	Secreted		*aes*	Aes family secreted acetyl esterase/lipase	50.8	1537
1538	MGCS36044_01842			-	calcium-binding protein	50.5	1538
1539	MGCS36044_02512			*cas1_1*	type II CRISPR-associated endonuclease Cas1	50.5	1538
1540	MGCS36044_00456			-	aminoglycoside 6-adenylyltransferase	50.3	1540
1541	MGCS36044_02080			-	IS30 family transposase	50.0	1541
1542	MGCS36044_00354			*ssb_1*	single-stranded DNA-binding protein Ssb	49.5	1542
1543	MGCS36044_03830			-	hypothetical protein	49.5	1542
1544	MGCS36044_01826			-	hypothetical protein	49.0	1544

No.	Locus tag	SignalP6 predicted[(1)]	Virulence[(2)]	Gene	Function	RPKMs[(3)]	RANK[(4)]
1545	MGCS36044_03094			-	SDR family oxidoreductase	49.0	1544
1546	MGCS36044_03926	Secreted	Virulence	*speG*	streptococcal pyrogenic exotoxin (G) SpeG	48.3	1546
1547	MGCS36044_01592			*aspB*	aspartate aminotransferase AspB	48.0	1547
1548	MGCS36044_01118			-	hypothetical protein	47.8	1548
1549	MGCS36044_01352			-	sigma factor regulator	47.8	1548
1550	MGCS36044_00318			-	DUF1033 domain-containing protein	47.5	1550
1551	MGCS36044_01262			*bglG_1*	transcriptional antiterminator BglG	47.5	1550
1552	MGCS36044_03080			-	hypothetical protein	47.5	1550
1553	MGCS36044_03990			-	IS982 family transposase	47.5	1550
1554	MGCS36044_04030			-	metallo-dependent amidohydrolase	47.3	1554
1555	MGCS36044_00626			-	MvaI/BcnI family restriction endonuclease	47.0	1555
1556	MGCS36044_01422			*fbp2*	fructose-bisphosphatase Fbp2	47.0	1555
1557	MGCS36044_03406			-	ARA1 family aldo/keto reductase	47.0	1555
1558	MGCS36044_01876			*copY_1*	copper transport repressor CopY	46.8	1558
1559	MGCS36044_03572			-	rhodanese-like domain-containing protein	46.8	1558
1560	MGCS36044_00478			-	site-specific integrase	46.5	1560
1561	MGCS36044_02474			-	YesM family TCS sensor histidine kinase	46.5	1560
1562	MGCS36044_03100			*rbsC*	D-ribose ABC transporter permease RbsC	46.5	1560
1563	MGCS36044_01026	Secreted		*yoaK*	putative secreted YoaK family protein	46.3	1563
1564	MGCS36044_00854	Lipo		-	MetQ/NlpA family ABC transporter substrate-binding lipoprotein	46.0	1564
1565	MGCS36044_03982	Secreted		-	putative secreted protein	46.0	1564
1566	MGCS36044_04174			-	arginase family protein	45.3	1566
1567	MGCS36044_02574	Secreted		*aphA*	secreted acid phosphatase AphA	45.0	1567
1568	MGCS36044_02658			*malA*	maltodextrose utilization protein MalA	45.0	1567
1569	MGCS36044_04070			*treR*	trehalose operon repressor TreR	45.0	1567
1570	MGCS36044_01906			-	unknown	44.8	1570
1571	MGCS36044_02510			*cas2_1*	CRISPR-associated endoribonuclease Cas2	44.8	1570
1572	MGCS36044_03236			*yncA*	YncA family L-amino acid N-acyltransferase	44.5	1572
1573	MGCS36044_02084			*kefB*	Kef-type K+ transporter membrane component KefB	44.0	1573
1574	MGCS36044_01212			*dhaS*	dihydroxyacetone kinase transcriptional	43.8	1574
1575	MGCS36044_02800			-	MdlB family multidrug ABC transporter ATPase and permease	43.8	1574
1576	MGCS36044_02852			*eda*	Eda family bifunctional	43.8	1574
1577	MGCS36044_03234			-	ParB-like nuclease domain-containing protein	43.8	1574
1578	MGCS36044_00454			-	type II toxin-antitoxin system PemK/MazF family	43.5	1578
1579	MGCS36044_02456			-	HAD hydrolase family protein	43.5	1578
1580	MGCS36044_02700			*pnuC*	nicotinamide riboside transporter PnuC	43.5	1578
1581	MGCS36044_04200			-	IS1182 family transposase	43.5	1578
1582	MGCS36044_00134			*purK*	5-(carboxyamino)imidazole ribonucleotide	43.3	1582
1583	MGCS36044_02644			*malQ*	4-alpha-glucanotransferase (amylomaltase)	43.3	1582
1584	MGCS36044_00468			-	hypothetical protein	43.0	1584
1585	MGCS36044_01992			*xpt*	xanthine phosphoribosyltransferase Xpt	42.8	1585
1586	MGCS36044_02716			-	MFS transporter	42.8	1585
1587	MGCS36044_01214			*dhaK*	dihydroxyacetone kinase subunit DhaK	42.5	1587

No.	Locus tag	SignalP6 predicted(1)	Virulence(2)	Gene	Function	RPKMs(3)	RANK(4)
1588	MGCS36044_02230			*norG*	PLP-dependent aminotransferase family protein	42.5	1587
1589	MGCS36044_00444			*flaR*	DNA topology modulation protein	42.3	1589
1590	MGCS36044_03102			*rbsA*	D-ribose transporter ATPase RbsA	41.8	1590
1591	MGCS36044_00446			-	GNAT family N-acetyltransferase	41.5	1591
1592	MGCS36044_01276			-	cupin domain-containing protein	41.3	1592
1593	MGCS36044_01718	Secreted		*sdrD*	SdrD superfamily cell surface extracellular	41.3	1592
1594	MGCS36044_03224			*bglB_2*	6-phospho-beta-glucosidase BglB	41.3	1592
1595	MGCS36044_03922			*resA*	TlpA family protein disulfide reductase	41.3	1592
1596	MGCS36044_00430			-	ATP-binding cassette domain-containing protein	40.5	1596
1597	MGCS36044_00936			-	FMN RNA	40.5	1596
1598	MGCS36044_02044			-	LolD superfamily ABC transporter ATP-binding	40.5	1596
1599	MGCS36044_03198			-	hypothetical protein	40.3	1599
1600	MGCS36044_03576			-	rhodanese-like domain-containing protein	40.3	1599
1601	MGCS36044_04026			-	cspA RNA	40.3	1599
1602	MGCS36044_04176			-	radical SAM protein	40.0	1602
1603	MGCS36044_03316			-	LysR family transcriptional regulator	39.8	1603
1604	MGCS36044_01840	Secreted		-	cell surface extracellular antigen I/II family	39.5	1604
1605	MGCS36044_02592			-	hypothetical protein	38.8	1605
1606	MGCS36044_01908			-	DUF3173 domain-containing protein	38.3	1606
1607	MGCS36044_02228			*pdxK*	pyridoxamine kinase PdxK	38.3	1606
1608	MGCS36044_01904			-	Cro/CI family transcriptional regulator	38.0	1608
1609	MGCS36044_02168			-	Gx transporter family protein	37.8	1609
1610	MGCS36044_04188			-	NUDIX hydrolase	37.8	1609
1611	MGCS36044_00464			-	hypothetical protein	37.5	1611
1612	MGCS36044_04074			-	hypothetical protein	37.5	1611
1613	MGCS36044_01956			*radC*	DNA repair protein RadC	37.3	1613
1614	MGCS36044_01126		Virulence	*silA*	TCS DNA-binding response regulator SilA	37.0	1614
1615	MGCS36044_01952			-	NanC-like SGNH/GDSL hydrolase family protein	37.0	1614
1616	MGCS36044_02034	Lipo		-	streptococcal histidine triad (HIT) lipoprotein	37.0	1614
1617	MGCS36044_02990			-	Rgg/GadR/MutR family transcriptional regulator	37.0	1614
1618	MGCS36044_01038			-	hypothetical protein	36.8	1618
1619	MGCS36044_01158	Secreted		-	secreted transglutaminase	36.8	1618
1620	MGCS36044_03338			*drpA*	DNA processing protein (A) DprA	36.8	1618
1621	MGCS36044_02842			-	BglX family glycosyl hydrolase	36.3	1621
1622	MGCS36044_03628			*scrK*	fructokinase protein ScrK	35.5	1622
1623	MGCS36044_03754			-	type IV secretion system DNA-binding	35.5	1622
1624	MGCS36044_00536			-	hypothetical protein	35.0	1624
1625	MGCS36044_00498			*ptsG*	PTS glucose-specific II ABC component	34.8	1625
1626	MGCS36044_01874			*cadD_1*	CadD family cadmium resistance transporter	34.8	1625
1627	MGCS36044_03718			-	OmpR family TCS DNA-binding response regulator	34.8	1625
1628	MGCS36044_03908	Secreted	Virulence	*fbpB*	cell surface fibronectin binding protein (B)	34.5	1628
1629	MGCS36044_00466			-	hypothetical protein	34.3	1629
1630	MGCS36044_01950			*araC*	AraC family transcriptional regulator	34.3	1629

No.	Locus tag	SignalP6 predicted[(1)]	Virulence[(2)]	Gene	Function	RPKMs[(3)]	RANK[(4)]
1631	MGCS36044_03714	Lipo		-	chromosome assembly-related lipoprotein	34.3	1629
1632	MGCS36044_00436			-	Maf family protein	34.0	1632
1633	MGCS36044_02064			*htpA*	streptococcal histidine triad (HIT)	34.0	1632
1634	MGCS36044_03716			-	BaeS family TCS sensor histidine kinase	34.0	1632
1635	MGCS36044_01852			-	hypothetical protein	33.5	1635
1636	MGCS36044_02042			*acrA*	AcrA superfamily multidrug efflux pump	33.5	1635
1637	MGCS36044_02514			*cas9*	type II CRISPR RNA-guided endonuclease Cas9	33.5	1635
1638	MGCS36044_03122			*arcT*	M20 family metallodipeptidase ArcT	33.5	1635
1639	MGCS36044_02226			-	ECF transporter S component	33.3	1639
1640	MGCS36044_04198			-	hypothetical protein	33.3	1639
1641	MGCS36044_01858			-	DUF5945 family protein	33.0	1641
1642	MGCS36044_00686			-	toxin-antitoxin system, toxin component, Fic	32.8	1642
1643	MGCS36044_01012			-	GloA family glyoxalase/bleomycin	32.8	1642
1644	MGCS36044_01030			*nrdF_1*	ribonucleotide-diphosphate reductase subunit	32.8	1642
1645	MGCS36044_01402			*idnO*	gluconate 5-dehydrogenase IdnO	32.8	1642
1646	MGCS36044_03106			*rbsK*	ribokinase RbsK	32.8	1642
1647	MGCS36044_03776			*rpsN2*	30S ribosomal S14-2 protein RpsN2	32.8	1642
1648	MGCS36044_00420			-	hypothetical protein	32.5	1648
1649	MGCS36044_01168			-	hypothetical protein	32.5	1648
1650	MGCS36044_04196			-	YhgE/Pip domain-containing protein	32.5	1648
1651	MGCS36044_01034			*nrdE_1*	class 1b ribonucleoside-diphosphate reductase	32.0	1651
1652	MGCS36044_01942			*maeK*	TCS signal transduction sensor histidine kinase	31.8	1652
1653	MGCS36044_03386			-	putative transcriptional regulator	31.8	1652
1654	MGCS36044_00132			*purE*	5-(carboxyamino)imidazole ribonucleotide mutase	31.3	1654
1655	MGCS36044_01856			-	DUF5965 family protein	31.3	1654
1656	MGCS36044_03104			*rbsD*	D-ribose pyranase RbsD	31.3	1654
1657	MGCS36044_03108			*rbsR*	ribose transport operon repressor RbsR	31.3	1654
1658	MGCS36044_03444			-	IS982 family transposase	31.3	1654
1659	MGCS36044_03958			*ulaG*	L-ascorbate utilization protein (G) UlaG	31.3	1654
1660	MGCS36044_01032			*nrdI_2*	ribonucleotide reductase assembly protein NrdI	30.5	1660
1661	MGCS36044_03226			-	beta-N-acetylglucosaminidase	30.0	1661
1662	MGCS36044_03632			*scrB*	sucrose-6-phosphate hydrolase ScrB	30.0	1661
1663	MGCS36044_00124			*vanZ*	VanZ family putative glycopeptide antibiotic	29.8	1663
1664	MGCS36044_00534	Secreted	Virulence	*pulA_1*	cell surface pullulanase PulA	29.8	1663
1665	MGCS36044_00130			*purD*	phosphoribosylamine--glycine ligase PurD	29.5	1665
1666	MGCS36044_02040	Lipo		-	ABC transporter protein	29.5	1665
1667	MGCS36044_02106			-	hypothetical protein	29.5	1665
1668	MGCS36044_04076			*yybR*	putative HTH-type transcriptional regulator	29.5	1665
1669	MGCS36044_00462	Secreted		-	cell surface PgrA surface exclusion	29.3	1669
1670	MGCS36044_02660			*malD*	maltodextrin transport system permease protein	29.3	1669
1671	MGCS36044_03686			-	alpha-glycosidase	29.0	1671
1672	MGCS36044_01418			-	transposase	28.5	1672
1673	MGCS36044_03842			*nanK*	N-acetylmannosamine kinase NanK	28.5	1672

No.	Locus tag	SignalP6 predicted(1)	Virulence(2)	Gene	Function	RPKMs(3)	RANK(4)
1674	MGCS36044_02728	Lipo		*nlpA*	MetQ/NlpA family ABC transporter substrate-binding lipoprotein	28.3	1674
1675	MGCS36044_03388			-	FadH2 family NAD(FAD)-dependent dehydrogenase	28.0	1675
1676	MGCS36044_00120			*purM*	phosphoribosylformylglycinamidine cyclo-ligase	27.8	1676
1677	MGCS36044_02184			*oadA*	oxaloacetate decarboxylase subunit alpha OadA	27.5	1677
1678	MGCS36044_02748			-	MATE family efflux transporter	27.5	1677
1679	MGCS36044_00282			-	hypothetical protein	27.3	1679
1680	MGCS36044_00688			-	hypothetical protein	27.3	1679
1681	MGCS36044_02954	Lipo		*metQ*	methionine uptake ABC transporter	27.0	1681
1682	MGCS36044_03390			*glpF_1*	GlpF family glycerol uptake facilitator and	27.0	1681
1683	MGCS36044_04016			*mutT*	8-oxo-dGTP diphosphatase, DNA mismatch repair	27.0	1681
1684	MGCS36044_00122			*purN*	phosphoribosylglycinamide formyltransferase	26.5	1684
1685	MGCS36044_02480			-	AgaB family protein	26.5	1684
1686	MGCS36044_01166			-	ABC transporter ATP-binding protein	26.3	1686
1687	MGCS36044_01260			-	Unknown	26.3	1686
1688	MGCS36044_03228			-	PurR/LacI family transcriptional regulator	26.3	1686
1689	MGCS36044_01454			*agaS*	AgaS superfamily sugar isomerase SIS	26.0	1689
1690	MGCS36044_01830			-	PrgI family protein	26.0	1689
1691	MGCS36044_01834			-	phage tail tip lysozyme	26.0	1689
1692	MGCS36044_03742			-	hypothetical protein	26.0	1689
1693	MGCS36044_00128	Secreted		-	CHAP domain-containing protein	25.8	1693
1694	MGCS36044_00654			-	replication initiation factor domain-containing	25.8	1693
1695	MGCS36044_01832			-	AAA family ATPase	25.5	1695
1696	MGCS36044_01854			-	toprim domain-containing protein	25.5	1695
1697	MGCS36044_00500			*rgfB*	endonuclease/exonuclease/phosphatase family	25.3	1697
1698	MGCS36044_01316			-	NAD(P)-binding domain-containing protein	25.3	1697
1699	MGCS36044_02666	Lipo		*malX*	maltose/maltodextrin-binding lipoprotein MalX	25.3	1697
1700	MGCS36044_02750			-	PEP-utilizing enzyme	25.3	1697
1701	MGCS36044_02478			*manY*	ManY family PTS mannose/fructose IIC component	25.0	1701
1702	MGCS36044_03392			*glpO*	type 1 glycerol-3-phosphate oxidase GlpO	25.0	1701
1703	MGCS36044_02732			*dcm*	DNA (cytosine-5-)-methyltransferase Dcm	24.8	1703
1704	MGCS36044_03158			*cas4*	CRISPR-associated protein Cas4	24.8	1703
1705	MGCS36044_00572			*talA*	transaldolase TalA	24.5	1705
1706	MGCS36044_03914			-	hypothetical protein	24.5	1705
1707	MGCS36044_02476			*manZ*	ManZ family PTS mannose/fructose IID component	24.0	1707
1708	MGCS36044_00268			-	Rep family plasmid replication protein	23.8	1708
1709	MGCS36044_00662			-	conjugal transfer protein	23.8	1708
1710	MGCS36044_00664			-	cytoplasmic protein	23.8	1708
1711	MGCS36044_00796			*dacA_2*	D-alanyl-D-alanine carboxypeptidase DacA	23.8	1708
1712	MGCS36044_03124			*arcD*	arginine/ornithine antiporter protein ArcD	23.8	1708
1713	MGCS36044_00118			*purF*	amidophosphoribosyltransferase PurF	23.5	1713
1714	MGCS36044_03282			*comFC*	ComFC family predicted	23.5	1713
1715	MGCS36044_02482			*manX*	ManX family PTS mannose/fructose IIA component	23.0	1715
1716	MGCS36044_00472			-	FtsK/SpoIIIE domain-containing protein	22.8	1716

No.	Locus tag	SignalP6 predicted[(1)]	Virulence[(2)]	Gene	Function	RPKMs[(3)]	RANK[(4)]
1717	MGCS36044_03066			-	putative secreted protein	22.8	1716
1718	MGCS36044_01818			-	hypothetical protein	22.5	1718
1719	MGCS36044_01846			-	SNF2-related protein	22.5	1718
1720	MGCS36044_02686			*celB_2*	PTS cellobiose transporter IIC subunit CelB	22.3	1720
1721	MGCS36044_03854	Lipo		*ugpB_2*	carbohydrate ABC transporter substrate-binding	22.3	1720
1722	MGCS36044_00624			-	helix-turn-helix transcriptional regulator	22.0	1722
1723	MGCS36044_03916			-	hypothetical protein	22.0	1722
1724	MGCS36044_03992	Secreted		-	TrbC/VirB2-related secreted effector protein	22.0	1722
1725	MGCS36044_01152			-	CAAX amino terminal protease family protein	21.8	1725
1726	MGCS36044_02174			-	ABC transporter permease	21.5	1726
1727	MGCS36044_02572	Secreted		-	putative secreted protein	21.5	1726
1728	MGCS36044_04190			*padR*	PadR family transcriptional regulator	21.5	1726
1729	MGCS36044_00434			-	hypothetical protein	21.3	1729
1730	MGCS36044_01116			-	Blp family class II bacteriocin with	21.3	1729
1731	MGCS36044_02570	Lipo		-	putative lipoprotein	21.3	1729
1732	MGCS36044_03164			*csd1*	Csd1 family CRISPR-associated protein	21.3	1729
1733	MGCS36044_02176			-	ABC transporter ATPase	21.0	1733
1734	MGCS36044_02844			-	phosphoglycolate phosphatase	21.0	1733
1735	MGCS36044_00116			*purL*	phosphoribosylformylglycinamidine synthase PurL	20.8	1735
1736	MGCS36044_00676			-	XRE family transcriptional regulator	20.8	1735
1737	MGCS36044_00684			-	lantibiotic transporter	20.8	1735
1738	MGCS36044_02118			-	Rep family protein	20.8	1735
1739	MGCS36044_02956			*metP_2*	methionine uptake ABC transporter permease MetP	20.8	1735
1740	MGCS36044_00678			-	bacteriocin	20.5	1740
1741	MGCS36044_02202			*dprA*	DNA-processing protein DprA	20.5	1740
1742	MGCS36044_03156			*cas1_2*	type I-C CRISPR-associated endonuclease Cas1	20.5	1740
1743	MGCS36044_03160			*cas7*	type I-C CRISPR-associated protein Cas7/Csd2	20.5	1740
1744	MGCS36044_03394			*glpK*	glycerol kinase GlpK	20.5	1740
1745	MGCS36044_03928			-	Bacteria_small_SRP	20.5	1740
1746	MGCS36044_02664			*amyB*	cyclomaltodextrinase protein AmyB	20.3	1746
1747	MGCS36044_02992			-	MFS transporter	20.3	1746
1748	MGCS36044_03848			-	DUF386 family protein	20.3	1746
1749	MGCS36044_01416			-	IS3 family transposase	20.0	1749
1750	MGCS36044_03876			-	MefA-related MFS transporter	20.0	1749
1751	MGCS36044_04162			-	MerR family transcriptional regulator	20.0	1749
1752	MGCS36044_02662			*malC*	maltodextrin transport system permease protein MalC	19.8	1752
1753	MGCS36044_00470			-	hypothetical protein	19.5	1753
1754	MGCS36044_00680			-	lantibiotic dehydratase	19.5	1753
1755	MGCS36044_02734			-	hypothetical protein	19.5	1753
1756	MGCS36044_01820			-	CPBP family intramembrane metalloprotease	19.3	1756
1757	MGCS36044_03458			-	IS982 family transposase	19.3	1756
1758	MGCS36044_00666			-	hypothetical protein	19.0	1758
1759	MGCS36044_00682			-	lanthionine synthetase C family protein	19.0	1758

No.	Locus tag	SignalP6 predicted[(1)]	Virulence[(2)]	Gene	Function	RPKMs[(3)]	RANK[(4)]
1760	MGCS36044_01816			-	arsenate reductase	18.8	1760
1761	MGCS36044_04160			-	MGCS36044_04160replication protein	18.8	1760
1762	MGCS36044_03846			-	DUF624 domain-containing protein	18.5	1762
1763	MGCS36044_02062	Lipo	Virulence	*lmb*	bifunctional metal ABC transporter	18.3	1763
1764	MGCS36044_00270			-	hypothetical protein	18.0	1764
1765	MGCS36044_00570			-	PTS transporter IIC component UlaA-like	18.0	1764
1766	MGCS36044_01860			-	MGCS36044_018hypothetical protein60	18.0	1764
1767	MGCS36044_02576			-	hypothetical protein	18.0	1764
1768	MGCS36044_02692			*celA_2*	PTS cellobiose transporter IIB subunit CelA	17.8	1768
1769	MGCS36044_02958			*metN_2*	methionine uptake ABC transporter ATP-binding	17.8	1768
1770	MGCS36044_03658			-	hypothetical protein	17.8	1768
1771	MGCS36044_03500			-	SSRC34_1 RNA	17.5	1771
1772	MGCS36044_00142			-	helix-turn-helix domain-containing protein	17.3	1772
1773	MGCS36044_03850			*ugpE_2*	carbohydrate ABC transporter permease UgpE-like	17.3	1772
1774	MGCS36044_00284			-	NUDIX domain-containing protein	17.0	1774
1775	MGCS36044_01812			-	DNA (cytosine-5-)-methyltransferase	17.0	1774
1776	MGCS36044_02694			*bglG_2*	transcription antiterminator BglG	17.0	1774
1777	MGCS36044_03844			*nanA*	N-acetylneuraminate lyase	17.0	1774
1778	MGCS36044_03852			*ugpA_2*	carbohydrate ABC transporter permease UgpA-like	17.0	1774
1779	MGCS36044_02688			-	DUF3284 domain-containing protein	16.8	1779
1780	MGCS36044_04058			-	AAA family ATPase domain-containing DNA-binding	16.8	1779
1781	MGCS36044_02104			-	hypothetical protein	16.5	1781
1782	MGCS36044_02690			*celC_2*	PTS cellobiose transporter IIA subunit CelC	16.5	1781
1783	MGCS36044_03856			*nanE*	N-acetylmannosamine-6-phosphate 2-epimerase	16.5	1781
1784	MGCS36044_00852	Lipo		-	MetQ/NlpA family ABC transporter	16.3	1784
1785	MGCS36044_01692			-	PyrR RNA	16.3	1784
1786	MGCS36044_02922			*coiA*	competence protein CoiA	16.3	1784
1787	MGCS36044_03154			*cas2_2*	CRISPR-associated endonuclease Cas2	16.3	1784
1788	MGCS36044_03166			*cas5*	type I-C CRISPR-associated protein Cas5	16.3	1784
1789	MGCS36044_04156			-	FtsK/SpoIIIE domain-containing protein	16.3	1784
1790	MGCS36044_00474			-	Rep family protein	16.0	1790
1791	MGCS36044_01456			*lacD_1*	tagatose-bisphosphate aldolase LacD-like	16.0	1790
1792	MGCS36044_02736			-	RepA N-terminal domain-containing protein	16.0	1790
1793	MGCS36044_02116			-	hypothetical protein	15.8	1793
1794	MGCS36044_00144			-	quorum-sensing system DWW-type pheromone	15.5	1794
1795	MGCS36044_01144			-	lactobin A/cerein 7B family class IIb	15.3	1795
1796	MGCS36044_02082			*femX*	FemABX-like family peptidoglycan interpeptide	15.3	1795
1797	MGCS36044_02208			-	MFS transporter	15.3	1795
1798	MGCS36044_03162			*cas8*	type I-C CRISPR-associated protein Cas8c/Csd1	15.3	1795
1799	MGCS36044_04158			-	hypothetical protein	15.3	1795
1800	MGCS36044_01532			-	preQ1-II RNA	15.0	1800
1801	MGCS36044_02196			-	IS982 family transposase	15.0	1800
1802	MGCS36044_00568			-	PTS transporter IIB component UlaB-like	14.8	1802

No.	Locus tag	SignalP6 predicted[(1)]	Virulence[(2)]	Gene	Function	RPKMs[(3)]	RANK[(4)]
1803	MGCS36044_02698			*bglB_1*	6-phospho-beta-glucosidase BglB	14.5	1803
1804	MGCS36044_01064			-	Spy490380c RNA	14.3	1804
1805	MGCS36044_03422			*lacD_2*	tagatose-bisphosphate aldolase LacD	14.3	1804
1806	MGCS36044_00320			*comYA*	competence system type II/IV secretion system	14.0	1806
1807	MGCS36044_00338			-	elix-turn-helix transcriptional regulator	14.0	1806
1808	MGCS36044_00416			-	hypothetical protein	14.0	1806
1809	MGCS36044_01394			*agaW*	WPTS transporter hyaluronate-oligosaccharide-specific IIC component AgaW	14.0	1806
1810	MGCS36044_02114			-	hypothetical protein	14.0	1806
1811	MGCS36044_00272			-	hypothetical protein	13.8	1811
1812	MGCS36044_01392			*agaD*	PTS transporter	13.8	1811
1813	MGCS36044_03232			*mngB*	alpha-mannosidase MngB	13.8	1811
1814	MGCS36044_04154			-	hypothetical protein	13.8	1811
1815	MGCS36044_00476			-	DUF771 domain-containing protein	13.5	1815
1816	MGCS36044_00674			-	ImmA/IrrE family metallo-endopeptidase	13.5	1815
1817	MGCS36044_01150			-	hypothetical protein	13.5	1815
1818	MGCS36044_02124			-	site-specific integrase	13.5	1815
1819	MGCS36044_01282	Lipo		*pstS*	phosphate ABC transporter substrate-binding	13.3	1819
1820	MGCS36044_01396			*agaV*	VPTS transporter hyaluronate-oligosaccharide-specific IIB component AgaV	13.3	1819
1821	MGCS36044_01146			-	putative bacteriocin with double-glycine leader	13.0	1821
1822	MGCS36044_03984			-	MdlB family ABC transporter ATP-binding/permease protein	13.0	1821
1823	MGCS36044_02752			-	TetR/AcrR family transcriptional regulator	12.3	1823
1824	MGCS36044_00566			-	PTS transporter IIA component UlaC-like	12.0	1824
1825	MGCS36044_01288			*pstB*	phosphate ABC transporter ATP-binding protein	12.0	1824
1826	MGCS36044_03426			*lacB*	galactose-6-phosphate isomerase subunit LacB	12.0	1824
1827	MGCS36044_00722			*comX_1*	competence protein ComX	11.8	1827
1828	MGCS36044_00822			*comX_2*	competence protein ComX	11.8	1827
1829	MGCS36044_01148			-	putative bacteriocin with double-glycine leader peptide	11.8	1827
1830	MGCS36044_02738			-	hypothetical protein. Region of difference 36044_ROD.7, putative MGE	11.8	1827
1831	MGCS36044_01142			-	BlpM-like bacteriocin with double-glycine leader	11.5	1831
1832	MGCS36044_01398			*ugl*	unsaturated chondroitin disaccharide hydrolase	11.5	1831
1833	MGCS36044_03710			-	Xre family helix-turn-helix transcriptional. Region of difference 36044_ROD.8, putative MGE	11.5	1831
1834	MGCS36044_01286			*pstA*	phosphate ABC transporter, permease protein PstA	11.3	1834
1835	MGCS36044_01468			-	PTS sugar transport IIA subunit	11.3	1834
1836	MGCS36044_01814			-	hypothetical protein	11.3	1834
1837	MGCS36044_03424			*lacC*	tagatose-6-phosphate kinase LacC	11.3	1834
1838	MGCS36044_00660			-	MGCS36044_006FtsK/SpoIIIE domain-containing protein60	11.0	1838
1839	MGCS36044_01290			*phoU_1*	phosphate signaling complex protein PhoU	11.0	1838
1840	MGCS36044_03284			*comFA*	ComFA family DNA/RNA helicase	11.0	1838
1841	MGCS36044_01284			*pstC*	phosphate ABC transporter, permease protein	10.8	1841
1842	MGCS36044_00114			*purC*	phosphoribosylaminoimidazolesuccinocarboxamide	10.5	1842
1843	MGCS36044_00652			-	conjugal transfer protein	10.5	1842
1844	MGCS36044_02742			-	hypothetical protein	10.5	1842

No.	Locus tag	SignalP6 predicted[(1)]	Virulence[(2)]	Gene	Function	RPKMs[(3)]	RANK[(4)]
1845	MGCS36044_03168			*cas3*	CRISPR-associated helicase Cas3	10.5	1842
1846	MGCS36044_00618			-	bacterial mobilization protein	10.3	1846
1847	MGCS36044_01888			-	DUF5960 family protein	10.3	1846
1848	MGCS36044_00646			-	conjugal transfer protein	10.0	1848
1849	MGCS36044_00404			*celB_1*	PTS transporter cellobiose-specific IIC	9.8	1849
1850	MGCS36044_00620			-	transposase	9.8	1849
1851	MGCS36044_00650			-	conjugal transfer protein	9.8	1849
1852	MGCS36044_01400			*agaF*	PTS transporter hyaluronate-oligosaccharide-specific IIA component AgaF	9.8	1849
1853	MGCS36044_02092			-	hypothetical protein	9.8	1849
1854	MGCS36044_03430			*sgcC*	galactitol-specific PTS transporter IIB subunit	9.8	1849
1855	MGCS36044_04152			-	sigma-70 family RNA polymerase sigma factor	9.8	1849
1856	MGCS36044_00648			-	conjugal transfer protein	9.5	1856
1857	MGCS36044_01020			*lctO*	L-lactate oxidase LctO	9.5	1856
1858	MGCS36044_03692			-	carbohydrate ABC transporter permease UgpE-like	9.5	1856
1859	MGCS36044_00698			*gatB_1*	PTS galactitol transporter IIB component GatB	9.3	1859
1860	MGCS36044_01318			-	ammonium transporter	9.3	1859
1861	MGCS36044_01460			*bga*	beta-galactosidase Bga	9.3	1859
1862	MGCS36044_02102			-	hypothetical protein	9.3	1859
1863	MGCS36044_02178			-	S41 family peptidase	9.3	1859
1864	MGCS36044_02860			*melB*	MelB family MFS transporter	9.3	1859
1865	MGCS36044_03144			*msrA/B*	Peptide methionine sulfoxide reductase	9.3	1859
1866	MGCS36044_03220			-	COG3537 superfamily putative	9.3	1859
1867	MGCS36044_00702			-	triose-phosphate isomerase	9.0	1867
1868	MGCS36044_00616			-	DUF5962 domain-containing protein	8.8	1868
1869	MGCS36044_01390			-	oligohyaluronate lyase	8.8	1868
1870	MGCS36044_01464			-	PTS sugar transport IIC subunit	8.8	1868
1871	MGCS36044_01774			-	HTH domain-containing putative transcriptional	8.8	1868
1872	MGCS36044_03428			*lacA*	galactose-6-phosphate isomerase subunit LacA	8.8	1868
1873	MGCS36044_00402			*celA_1*	PTS transporter cellobiose-specific IIB	8.5	1873
1874	MGCS36044_01110			-	hypothetical protein	8.5	1873
1875	MGCS36044_00704			-	YjbQ family protein	8.3	1875
1876	MGCS36044_01466			-	PTS sugar transport IID subunit	8.0	1876
1877	MGCS36044_02122			-	DUF3173 family protein	8.0	1876
1878	MGCS36044_03432			*sgaB*	galactitol-specific PTS transporter IIC subunit	8.0	1876
1879	MGCS36044_03838			-	cysteine hydrolase	8.0	1876
1880	MGCS36044_00614			-	phage replisome organizer N-terminal	7.8	1880
1881	MGCS36044_00690			-	tyrosine-type site-specific	7.8	1880
1882	MGCS36044_01462			-	PTS sugar transport IIB subunit	7.8	1880
1883	MGCS36044_03146	Lipo		*tlpA*	TlpA family protein disulfide reductase	7.8	1880
1884	MGCS36044_01018			*lctP*	L-lactate permease LctP	7.5	1884
1885	MGCS36044_01156			-	hypothetical protein	7.5	1884
1886	MGCS36044_01884			-	TIGR01906 family membrane protein	7.5	1884
1887	MGCS36044_02516			-	tracrRNA RNA	7.5	1884

No.	Locus tag	SignalP6 predicted[(1)]	Virulence[(2)]	Gene	Function	RPKMs[(3)]	RANK[(4)]
1888	MGCS36044_03230			-	metal-independent alpha-mannosidase	7.5	1884
1889	MGCS36044_00266			-	DUF3173 domain-containing protein	7.3	1889
1890	MGCS36044_04170			-	parvulin-like peptidyl-prolyl isomerase	7.3	1889
1891	MGCS36044_00696			*gatC_1*	PTS galactitol transporter IIC component GatC	7.0	1891
1892	MGCS36044_02094			-	FtsK/SpoIIIE domain-containing protein	6.8	1892
1893	MGCS36044_02866	Lipo	Virulence	*slr*	InlA-like streptococcal leucine rich lipoprotein	6.8	1892
1894	MGCS36044_03694			-	carbohydrate ABC transporter permease UgpA-like	6.8	1892
1895	MGCS36044_00264			*xerC*	site-specific integrase	6.5	1895
1896	MGCS36044_01886			-	hypothetical protein	6.5	1895
1897	MGCS36044_03218			*nagC*	NagC family sugar kinase	6.5	1895
1898	MGCS36044_01040	Secreted		-	putative secreted protein	6.3	1898
1899	MGCS36044_03148			*ccdA*	thiol-disulfide oxidoreductase-associated membrane protein	6.3	1898
1900	MGCS36044_03760			-	DUF3173 family protein	6.3	1898
1901	MGCS36044_00642			-	DNA segregation ATPase, conjugal transfer	6.0	1901
1902	MGCS36044_01946			*maeE*	NAD-dependent malic enzyme MaeE	6.0	1901
1903	MGCS36044_02096			-	hypothetical protein	6.0	1901
1904	MGCS36044_02754			*mobC*	plasmid mobilization relaxosome protein MobC	6.0	1901
1905	MGCS36044_03212	Lipo		*ugpB_1*	glycerol-3-phosphate ABC transporter	6.0	1901
1906	MGCS36044_03434			-	PTS sugar transporter subunit IIA	6.0	1901
1907	MGCS36044_03758			-	replication protein	6.0	1901
1908	MGCS36044_00644			-	conjugal transfer protein	5.8	1908
1909	MGCS36044_03214			*ugpE_1*	glycerol-3-phosphate ABC transporter permease	5.8	1908
1910	MGCS36044_00640			-	conjugal transfer protein	5.5	1910
1911	MGCS36044_01776	Secreted		-	extracellular cell wall anchored mucin-binding	5.5	1910
1912	MGCS36044_02582			-	hypothetical protein	5.5	1910
1913	MGCS36044_01944			*maeP*	citrate/malate symporter protein MaeP	5.3	1913
1914	MGCS36044_02120			-	hypothetical protein	5.3	1913
1915	MGCS36044_03216			*ugpA_1*	sugar ABC transporter permease UgpA	5.3	1913
1916	MGCS36044_03986			-	hypothetical protein	5.3	1913
1917	MGCS36044_00612			-	DNA-binding protein	5.0	1917
1918	MGCS36044_00692			-	PRD domain/PTS transporter IIA domain protein	5.0	1917
1919	MGCS36044_01140		Virulence	*silE*	streptococcal invasion locus pheromone	5.0	1917
1920	MGCS36044_02756			-	relaxase	5.0	1917
1921	MGCS36044_00322			*comYB*	competence system type II secretion system	4.5	1921
1922	MGCS36044_00982			*livG*	branched-chain amino acid ABC transporter ATP-binding protein LivG	4.5	1921
1923	MGCS36044_01362			-	glycosyltransferase family 2 protein	4.5	1921
1924	MGCS36044_02940			*comEC*	DNA internalization-related competence protein	4.5	1921
1925	MGCS36044_00400			*celC_1*	PTS transporter cellobiose-specific IIA	4.3	1925
1926	MGCS36044_00634			-	helix-turn-helix domain-containing protein	4.3	1925
1927	MGCS36044_00986			-	cystathionine beta-synthase (CBS)	4.3	1925
1928	MGCS36044_00636	Secreted		-	CHAP domain-containing protein	4.0	1928
1929	MGCS36044_00984			*livF*	branched-chain amino acid ABC transporter ATP-binding protein LivF	4.0	1928
1930	MGCS36044_02942			*comEA*	DNA uptake competence protein ComEA	4.0	1928

No.	Locus tag	SignalP6 predicted(1)	Virulence(2)	Gene	Function	RPKMs(3)	RANK(4)
1931	MGCS36044_00638			-	putative conjugal transfer protein	3.5	1931
1932	MGCS36044_00980			*livM*	branched-chain amino acid ABC transporter permease LivM	3.5	1931
1933	MGCS36044_02816			-	NTPase domain-containing protein	3.5	1931
1934	MGCS36044_00330	Pilin		*comYF*	competence system protein ComYF	3.3	1934
1935	MGCS36044_01138		Virulence	*silD*	streptococcal invasion locus pheromone secretion	3.3	1934
1936	MGCS36044_01356			-	hypothetical protein	3.3	1934
1937	MGCS36044_02744			-	unknown	3.3	1934
1938	MGCS36044_00326	Pilin		*comYD*	competence system type II secretion system GspH	3.0	1938
1939	MGCS36044_00976	Lipo		*livK*	branched-chain amino acid ABC transporter	3.0	1938
1940	MGCS36044_00978			*livH*	branched-chain amino acid ABC transporter permease LivH	3.0	1938
1941	MGCS36044_02764			*topB*	DNA topoisomerase III TopB	3.0	1938
1942	MGCS36044_01292	Secreted		-	putative secreted protein	2.8	1942
1943	MGCS36044_01360			-	glycosyltransferase	2.8	1942
1944	MGCS36044_03962			*ulaF*	L-ribulose-5-phosphate 4-epimerase UlaF	2.8	1942
1945	MGCS36044_00328	Pilin		*comYE*	competence system protein ComYE	2.5	1945
1946	MGCS36044_00332			*comYG*	competence system protein ComYG	2.5	1945
1947	MGCS36044_02810			-	site-specific serine recombinase family protein	2.5	1945
1948	MGCS36044_01358			-	hypothetical protein	2.3	1948
1949	MGCS36044_02814			-	DUF3801 domain-containing protein	2.3	1948
1950	MGCS36044_04042			*hutU*	urocanate hydratase HutU	2.3	1948
1951	MGCS36044_00324	Pilin		*comYC*	competence system prepilin-type N-terminal	2.0	1951
1952	MGCS36044_01154			-	hypothetical protein	2.0	1951
1953	MGCS36044_02818			-	RepA N-terminal domain-containing protein	2.0	1951
1954	MGCS36044_03756			-	hypothetical protein	2.0	1951
1955	MGCS36044_03964			*ulaE*	L-xylulose 5-phosphate 3-epimerase UlaE	2.0	1951
1956	MGCS36044_04050			*hutD*	histidine uptake and utilization HutD	2.0	1951
1957	MGCS36044_02790			-	phage replisome organizer N-terminal domain-containing protein	1.8	1957
1958	MGCS36044_03968			*ulaC*	ascorbate-specific PTS transporter EIIA protein	1.8	1957
1959	MGCS36044_04044			*fctD*	glutamate formiminotransferase FctD	1.8	1957
1960	MGCS36044_04048			*fhs_2*	formate--tetrahydrofolate ligase Fhs	1.8	1957
1961	MGCS36044_04052			*potE*	PotE family amino acid transporter	1.8	1957
1962	MGCS36044_04054			*hutH*	histidine ammonia-lyase HutH	1.8	1957
1963	MGCS36044_02758			-	conjugative element protein	1.5	1963
1964	MGCS36044_02812			*virD4*	type IV secretory system conjugative DNA transfer protein VirD4	1.5	1963
1965	MGCS36044_03966			*ulaD*	3-keto-L-gulonate 6-phosphate decarboxylase	1.5	1963
1966	MGCS36044_03970			*ulaB*	ascorbate-specific PTS transporter EIIB protein	1.5	1963
1967	MGCS36044_03972			*ulaA*	ascorbate-specific PTS transporter EIIC protein	1.5	1963
1968	MGCS36044_02784			*tnpW*	TnpW family transposon-encoded protein	1.3	1968
1969	MGCS36044_03988			-	hypothetical protein	1.3	1968
1970	MGCS36044_04046			-	formiminotetrahydrofolate cyclodeaminase	1.3	1968
1971	MGCS36044_01134		Virulence	*silCR*	streptococcal invasion locus auto-inducing	1.0	1971
1972	MGCS36044_02746			-	sigma-70 family RNA polymerase sigma factor like	1.0	1971
1973	MGCS36044_02760			-	DNA methyltransferase	1.0	1971

No.	Locus tag	SignalP6 predicted[(1)]	Virulence[(2)]	Gene	Function	RPKMs[(3)]	RANK[(4)]
1974	MGCS36044_02792			*mobA*	MobA/MobL family protein	1.0	1971
1975	MGCS36044_02772			*virB4*	type IV secretory pathway component VirB4	0.8	1975
1976	MGCS36044_02782			*traG_2*	conjugal transfer protein TraG		
1977	MGCS36044_01136			*silC*	streptococcal invasion locus signaling peptide	0.5	1977
1978	MGCS36044_02774			-	conjugal transfer protein	0.5	1977
1979	MGCS36044_02776			*virB6*	VirB6-like conjugal transfer protein	0.5	1977
1980	MGCS36044_02778			-	Maff2 family protein	0.5	1977
1981	MGCS36044_02796			-	sigma-70 family RNA polymerase sigma factor like protein	0.5	1977
1982	MGCS36044_02766	Secreted		-	DUF4366 domain-containing predicted secreted	0.3	1982
1983	MGCS36044_02768			-	conjugal transfer protein	0.3	1982
1984	MGCS36044_02780			-	single-stranded DNA-binding protein	0.3	1982
1985	MGCS36044_02770			-	CHAP domain-containing protein	0.0	1985
1986	MGCS36044_02794			-	DUF3847 domain-containing protein	0.0	1985

MGCS36044 was grown *in vitro*, in rich media, and in quadruplicate. Genes were ranked by the mean transcript abundance calculated from four replicates, at mid-exponential (ME) growth phase (OD=1.0). Genes are ordered by transcript abundance rank, from highest (rank=1) to lowest.

(1) Genes predicted by SignalP 6.0 to have an export signal sequence.
(2) Virulence refers to putative virulence genes.
(3) RPKMs were assigned by EDGEPro.
(4) Rank refers to the corresponding transcript abundance rank based on RPKMs.
(5) Lipo, exported lipoprotein attached to the cell envelope.
(6) TCS, two-component system.

Table S2B. Ranked MGCS36044 genes during growth *in vitro* at early stationary phase

No.	Locus tag	Signal6P predicted[(1)]	Virulence[(2)]	Gene	Function	RPKMs[(3)]	RANK[(4)]
1	MGCS36044_01512		Virulence	-	sagA RNA	99748.5	1
2	MGCS36044_01510		Virulence	*sagA*	streptolysin S precursor SagA	69008.0	2
3	MGCS36044_03334			-	RNaseP_bact_b RNA	42223.3	3
4	MGCS36044_02384			*ssrA*	transfer-messenger RNA	33701.0	4
5	MGCS36044_01354			*tufA*	translation elongation factor Tu protein TufA	27632.3	5
6	MGCS36044_03808		Virulence	*gapA*	glyceraldehyde-3-phosphate dehydrogenase GapA	22529.0	6
7	MGCS36044_03126			*arcB*	ornithine carbamoyltransferase ArcB	16401.0	7
8	MGCS36044_03128			-	GNAT family N-acetyltransferase	16204.5	8
9	MGCS36044_02344			*yeaQ*	GlsB/YeaQ/YmgE family stress response membrane	14461.5	9
10	MGCS36044_03130			*arcA*	arginine deiminase ArcA	14113.3	10
11	MGCS36044_00482			*ssrS*	6S RNA	13134.5	11
12	MGCS36044_02334			-	Asp23/Gls24 family envelope stress response protein	9810.0	12
13	MGCS36044_02336			-	CsbD family protein	9322.5	13
14	MGCS36044_02340			-	DUF2273 domain-containing protein	8642.5	14
15	MGCS36044_01508		Virulence	*srrG*	streptolysin S small regulatory RNA SrrG	8467.3	15
16	MGCS36044_01506			*eno*	phosphopyruvate hydratase -- enolase protein	8203.0	16
17	MGCS36044_02338			-	Asp23/Gls24 family envelope stress response protein	8023.0	17
18	MGCS36044_03122			*arcT*	M20 family metallodipeptidase ArcT	7361.3	18
19	MGCS36044_04038			*ahpF*	alkyl hydroperoxide reductase F subunit AhpF	7294.5	19
20	MGCS36044_01514		Virulence	*sagB*	streptolysin S biosynthesis protein SagB	7113.3	20
21	MGCS36044_02650	Lipo[(5)]		*malE*	maltose/maltodextrin ABC transport system substrate-binding lipoprotein MalE	6933.0	21
22	MGCS36044_02342			*amaP*	alkaline shock response membrane anchor protein	6912.5	22
23	MGCS36044_03118			*arcC*	carbamate kinase ArcC	6051.8	23
24	MGCS36044_03124			*arcD*	arginine/ornithine antiporter protein ArcD	6048.8	24
25	MGCS36044_00572			*talA*	transaldolase TalA	5789.8	25
26	MGCS36044_02216			-	apolipoprotein A1/A4/E family protein	5789.8	25
27	MGCS36044_02888			*ptsH*	PTS transporter phosphocarrier protein PtsH	5755.3	27
28	MGCS36044_03078			*dps*	DNA protection during starvation protein	5674.8	28
29	MGCS36044_01528		Virulence	*sagI*	streptolysin S export permease protein SagI	4993.5	29
30	MGCS36044_01522		Virulence	*sagF*	streptolysin S biosynthesis protein SagF	4922.5	30
31	MGCS36044_02218			-	hypothetical protein	4883.0	31
32	MGCS36044_00758			*fba_2*	fructose-bisphosphate aldolase	4635.0	32
33	MGCS36044_01526		Virulence	*sagH*	streptolysin S export permease protein SagH	4623.3	33
34	MGCS36044_04036			*ahpC*	alkyl hydroperoxide reductase C subunit AhpC	4584.5	34
35	MGCS36044_01520		Virulence	*sagE*	streptolysin S self-immunity protein SagE	4472.5	35
36	MGCS36044_02840	Secreted	Virulence	*spg*	extracellular cell surface IgG-binding	4459.0	36
37	MGCS36044_01516		Virulence	*sagC*	streptolysin S biosynthesis protein SagC	4353.3	37
38	MGCS36044_02994			-	DNA-binding protein HU	4110.5	38
39	MGCS36044_01524		Virulence	*sagG*	streptolysin S export protein SagG	4089.8	39

No.	Locus tag	Signal6P predicted[(1)]	Virulence[(2)]	Gene	Function	RPKMs[(3)]	RANK[(4)]
40	MGCS36044_00570			-	PTS transporter IIC component UlaA-like	3974.8	40
41	MGCS36044_01518		Virulence	*sagD*	streptolysin S biosynthesis protein SagD	3970.0	41
42	MGCS36044_03682			*pflB*	formate C-acetyltransferase	3796.5	42
43	MGCS36044_02220			-	CsbD family protein	3769.8	43
44	MGCS36044_03280			*raiA*	ribosome-associated translation inhibitor RaiA	3586.0	44
45	MGCS36044_00874			*htpX*	zinc metalloprotease HtpX	3000.5	45
46	MGCS36044_03344			*pepC*	aminopeptidase (A) PepC	2978.0	46
47	MGCS36044_03120			-	IS110 family transposase	2976.3	47
48	MGCS36044_00568			-	PTS transporter IIB component UlaB-like	2942.8	48
49	MGCS36044_03336			*gpsB*	cell division regulator GpsB	2846.8	49
50	MGCS36044_00156			*adhP*	alcohol dehydrogenase AdhP	2777.0	50
51	MGCS36044_00872			*lemA*	LemA family protein	2725.0	51
52	MGCS36044_01336			-	transposase IS116/IS110/IS902 family protein	2718.5	52
53	MGCS36044_00158			-	hypothetical protein	2679.0	53
54	MGCS36044_02020			*fadH2*	FadH2 superfamily FAD-dependent oxidoreductase	2627.3	54
55	MGCS36044_03096			-	IS110 family transposase	2625.0	55
56	MGCS36044_00194			*rpsZ*	type Z 30S ribosomal S14 protein RpsZ	2553.3	56
57	MGCS36044_03544			*dnaK*	molecular chaperone DnaK	2511.0	57
58	MGCS36044_01066	Lipo		*mtsA*	metal ABC transporter substrate-binding	2423.8	58
59	MGCS36044_02936			*sodA*	superoxide dismutase SodA	2423.8	58
60	MGCS36044_00192			*rplE*	50S ribosomal L5 protein RplE	2398.5	60
61	MGCS36044_04104			*spxA_2*	transcriptional regulator SpxA	2336.5	61
62	MGCS36044_00566			-	PTS transporter IIA component UlaC-like	2205.8	62
63	MGCS36044_00190			*rplX*	50S ribosomal L24 protein RplX	2163.8	63
64	MGCS36044_00590			*rmlB*	23S rRNA (guanosine(2251)-2'-O)-methyltransferase RlmB	2160.8	64
65	MGCS36044_01708			-	ABC transporter permease	2152.3	65
66	MGCS36044_03810			*fusA*	FusA family elongation factor EF-G	2140.5	66
67	MGCS36044_02024			*ldh*	L-lactate dehydrogenase Ldh	2105.5	67
68	MGCS36044_01554			*atpF*	ATP synthase B subunit AtpF	2104.5	68
69	MGCS36044_01564			*atpC*	ATP synthase epsilon subunit AtpC	2051.8	69
70	MGCS36044_01710			-	TVP38 superfamily protein	2051.3	70
71	MGCS36044_01558			*atpA*	ATP synthase alpha chain, AtpA	2010.5	71
72	MGCS36044_00178			*rplV*	50S ribosomal L22 protein RplV	1990.8	72
73	MGCS36044_00752			*lrgB*	antiholin-like protein LrgB	1988.0	73
74	MGCS36044_00180			*rpsC*	30S ribosomal S3 protein RpsC	1956.0	74
75	MGCS36044_00186			*rpsQ*	30S ribosomal S17 protein RpsQ	1937.8	75
76	MGCS36044_01706			-	LolD superfamily ABC transporter ATPase	1932.0	76
77	MGCS36044_02886			*ptsI*	phosphoenolpyruvate--protein phosphotransferase	1931.3	77
78	MGCS36044_00182			*rplP*	50S ribosomal L29 protein RplP	1931.0	78
79	MGCS36044_01562			*atpD*	ATP synthase beta subunit AtpD	1910.5	79
80	MGCS36044_00188			*rplN*	50S ribosomal L14 protein RplN	1885.3	80

No.	Locus tag	Signal6P predicted[(1)]	Virulence[(2)]	Gene	Function	RPKMs[(3)]	RANK[(4)]
81	MGCS36044_00592			-	NYN domain-containing protein	1885.3	80
82	MGCS36044_01556			*atpH*	ATP synthase delta subunit AtpH	1869.5	82
83	MGCS36044_02670			*dltC*	D-alanine--poly(phosphoribitol) ligase subunit	1848.8	83
84	MGCS36044_00218			*rpsK*	30S ribosomal S11 protein RpsK	1838.5	84
85	MGCS36044_02458			-	NAD-dependent succinate-semialdehyde	1780.5	85
86	MGCS36044_01560			*atpG*	ATP synthase gamma subunit AtpG	1771.3	86
87	MGCS36044_02672			*dltB*	D-alanyl-lipoteichoic acid biosynthesis protein DltB	1753.3	87
88	MGCS36044_00222			*rplQ*	50S ribosomal L17 protein RplQ	1736.5	88
89	MGCS36044_00198			*rplF*	50S ribosomal L6 protein RplF	1724.5	89
90	MGCS36044_00176			*rpsS*	30S ribosomal S19 protein RpsS	1697.3	90
91	MGCS36044_01552			*atpB*	ATP synthase A subunit AtpB	1694.8	91
92	MGCS36044_00224			-	L17DE RNA	1689.5	92
93	MGCS36044_01704			-	RND family transporter membrane fusion protein	1682.5	93
94	MGCS36044_00174			*rplB*	50S ribosomal L2 protein RplB	1661.5	94
95	MGCS36044_02440			*arsC_2*	arsenate reductase ArsC	1652.3	95
96	MGCS36044_03506			*manN*	PTS transporter mannose-specific IID component	1621.3	96
97	MGCS36044_02668			*dltD*	D-alanyl-lipoteichoic acid biosynthesis protein	1618.0	97
98	MGCS36044_02438			*xapA*	XapA family purine-nucleoside phosphorylase	1616.3	98
99	MGCS36044_00220			*rpoA*	DNA-directed RNA polymerase subunit alpha RpoA	1603.8	99
100	MGCS36044_02674			*dltA*	D-alanine--poly(phosphoribitol) ligase subunit DltA	1603.5	100
101	MGCS36044_02442			*deoB*	phosphopentomutase DeoB	1576.3	101
102	MGCS36044_00588	Secreted		-	putative secreted protein	1562.3	102
103	MGCS36044_03662			*trxA_2*	thioredoxin TrxA	1541.0	103
104	MGCS36044_02370			*pyk*	pyruvate kinase Pyk	1531.5	104
105	MGCS36044_00204			*rpmD*	50S ribosomal L30 protein RpmD	1507.5	105
106	MGCS36044_03546			*grpE*	heat shock protein/nucleotide exchange factor	1501.0	106
107	MGCS36044_04014	Secreted		*pepD_2*	secreted dipeptidase PepD	1489.5	107
108	MGCS36044_00172			*rplW*	50S ribosomal L23 protein RplW	1485.3	108
109	MGCS36044_00216			*rpsM*	30S ribosomal S13 protein RpsM	1474.8	109
110	MGCS36044_00170			*rplD*	50S ribosomal L4 protein RplD	1472.3	110
111	MGCS36044_03814			*rpsL*	30S ribosomal S12 protein RpsL	1462.0	111
112	MGCS36044_00208			*secY*	preprotein translocase subunit SecY	1450.8	112
113	MGCS36044_00196			*rpsH*	30S ribosomal S8 protein RpsH	1449.5	113
114	MGCS36044_02276	Lipo		-	putative nucleoside ABC transporter	1447.8	114
115	MGCS36044_01804			*rplL*	50S ribosomal L7/L12 protein RplL	1447.0	115
116	MGCS36044_00214			*rpmJ*	50S ribosomal L36 protein RpmJ	1435.8	116
117	MGCS36044_03448			*srtB*	pilus polymerization class B sortase SrtB	1430.3	117
118	MGCS36044_03586			*uspA*	UspA family nucleotide-binding universal stress	1422.0	118
119	MGCS36044_02980			*gpmA*	phosphoglycerate mutase GpmA	1421.5	119
120	MGCS36044_00154			*adhE*	bifunctional acetaldehyde-CoA/alcohol	1413.5	120
121	MGCS36044_01680			*prp*	ribosomal-processing cysteine protease Prp	1402.0	121

No.	Locus tag	Signal6P predicted[(1)]	Virulence[(2)]	Gene	Function	RPKMs[(3)]	RANK[(4)]
122	MGCS36044_01806			-	rli38 RNA	1389.8	122
123	MGCS36044_02436			*deoD*	DeoD-type purine-nucleoside phosphorylase	1381.8	123
124	MGCS36044_00200			*rplR*	50S ribosomal L18 protein RplR	1381.0	124
125	MGCS36044_02884			*gapN*	NADP-dependent glyceraldehyde-3-phosphate	1374.0	125
126	MGCS36044_03656			*rpsF*	30S ribosomal S6 protein RpsF	1367.0	126
127	MGCS36044_04068			*treB*	PTS transporter trehalose-specific EIIBC	1357.5	127
128	MGCS36044_03396	Lipo		-	PepSY domain-containing lipoprotein	1354.5	128
129	MGCS36044_01490			*rplS*	50S ribosomal L19 protein RpsL	1351.5	129
130	MGCS36044_02278			*cdd*	cytidine deaminase Cdd	1348.0	130
131	MGCS36044_01272			-	SPJ_0845 family protein	1343.5	131
132	MGCS36044_03804			*pgk*	phosphoglycerate kinase Pgk	1337.8	132
133	MGCS36044_03812			*rpsG*	30S ribosomal S7 protein RpsG	1332.8	133
134	MGCS36044_01364			*tpiA*	triose-phosphate isomerase TpiA	1318.3	134
135	MGCS36044_03446	Secreted		-	pilus ancillary/minor protein 2	1309.3	135
136	MGCS36044_01716			-	KH domain-containing protein	1307.0	136
137	MGCS36044_01470			*rpmE*	50S ribosomal L31 type B protein RpmE	1302.5	137
138	MGCS36044_00184			*rpmC*	50S ribosomal L16 protein RpmC	1285.3	138
139	MGCS36044_03654			*ssb_2*	single-stranded DNA-binding protein	1278.5	139
140	MGCS36044_03956			-	diacylglycerol kinase family lipid kinase	1268.0	140
141	MGCS36044_04060			*rpsB*	30S ribosomal S2 protein RpsB	1260.0	141
142	MGCS36044_03504			*manM*	PTS transporter mannose-specific IIC component	1238.0	142
143	MGCS36044_03502			*manL*	PTS transporter mannose-specific IIB & IIA	1231.5	143
144	MGCS36044_03806	Lipo		*lppC*	e(P4) family 5'-nucleotidase lipoprotein	1197.8	144
145	MGCS36044_00168			*rplC*	50S ribosomal L3 protein RplC	1196.8	145
146	MGCS36044_00910			*yccA*	YccA family protein	1192.3	146
147	MGCS36044_02372			*pfkA*	6-phosphofructokinase PfkA	1171.3	147
148	MGCS36044_00202			*rpsE*	30S ribosomal S5 protein RpsE	1163.8	148
149	MGCS36044_01392			*agaD*	PTS transporter	1150.5	149
150	MGCS36044_03040			-	DUF1827 family protein	1149.3	150
151	MGCS36044_02392			*rpsA*	30S ribosomal S1 protein RpsA	1147.8	151
152	MGCS36044_01802			*rplJ*	50S ribosomal L10 protein RplJ	1142.8	152
153	MGCS36044_03452			*sipA*	signal peptidase I SipA	1142.8	152
154	MGCS36044_03450	Secreted		-	pilus backbone/major protein	1142.5	154
155	MGCS36044_03048			-	RNA-binding protein	1137.8	155
156	MGCS36044_02280			*deoC*	deoxyribose-phosphate aldolase DeoC	1131.5	156
157	MGCS36044_01080			*rplA*	50S ribosomal L1 protein RplA	1124.0	157
158	MGCS36044_01650			*rplT*	50S ribosomal L20 protein RplT	1123.8	158
159	MGCS36044_00564			*rpsO*	30S ribosomal S15 protein RpsO	1122.0	159
160	MGCS36044_01600			*mscL*	large-conductance mechanosensitive channel	1102.0	160
161	MGCS36044_00606			*rpsI*	30S ribosomal S9 protein RpsI. Region of difference 36044_ROD.3, putative MGE	1095.5	161
162	MGCS36044_03920			*pgi*	Pgi family glucose-6-phosphate isomerase	1093.5	162

No.	Locus tag	Signal6P predicted[(1)]	Virulence[(2)]	Gene	Function	RPKMs[(3)]	RANK[(4)]
163	MGCS36044_01394			*agaW*	WPTS transporter hyaluronate-oligosaccharide-specific IIC component AgaW	1092.3	163
164	MGCS36044_03536			*fabT*	transcriptional regulatory protein FabT	1092.3	163
165	MGCS36044_03548			*hrcA*	heat-inducible transcriptional repressor HrcA	1072.0	165
166	MGCS36044_03056			*ftsZ*	cell division protein FtsZ	1070.0	166
167	MGCS36044_00878		Virulence	*covR*	TCS[(6)] DNA-binding response regulator CovR	1050.0	167
168	MGCS36044_01798			*clpL*	ATP-dependent Clp protease ATP-binding subunit	1046.8	168
169	MGCS36044_00740			*tig*	trigger factor molecular chaperone Tig	1046.5	169
170	MGCS36044_01194		Virulence	*ccpA*	catabolite control protein CcpA	1041.8	170
171	MGCS36044_03054			*yggS*	YggS family pyridoxal phosphate-dependent	1038.0	171
172	MGCS36044_01396			*agaV*	VPTS transporter hyaluronate-oligosaccharide-specific IIB component AgaV	1031.5	172
173	MGCS36044_03616	Secreted	Virulence	*isp2*	Isp-related CHAP domain-containing immunogenic	1015.8	173
174	MGCS36044_01978			*eutD*	phosphate acetyltransferase EutD	1005.0	174
175	MGCS36044_01410	Secreted	Virulence	*hylB*	secreted hyaluronate lyase HylB	997.0	175
176	MGCS36044_03052			*sepF*	cell division protein SepF	989.8	176
177	MGCS36044_01306			-	YtxH domain-containing protein	986.8	177
178	MGCS36044_03522			*accB*	acetyl-CoA carboxylase biotin carboxyl carrier	982.8	178
179	MGCS36044_01298			-	PspC domain-containing protein	974.5	179
180	MGCS36044_03050			-	YggT family protein	968.5	180
181	MGCS36044_01398			*ugl*	unsaturated chondroitin disaccharide hydrolase	965.8	181
182	MGCS36044_03518			*accC*	acetyl-CoA carboxylase biotin carboxylase	965.0	182
183	MGCS36044_04098			*ruvX*	Holliday junction resolvase RuvX	949.3	183
184	MGCS36044_01220			*gla*	glycerol uptake facilitator protein Gla	939.0	184
185	MGCS36044_02380			-	ABC transporter permease	931.8	185
186	MGCS36044_02600			*rfbA*	glucose-1-phosphate thymidylyltransferase RfbA	926.5	186
187	MGCS36044_03046			*divIVA*	cell division protein DivIVA	922.3	187
188	MGCS36044_04062			*tsf*	translation elongation factor Tsf	911.5	188
189	MGCS36044_03534			*fabH*	3-oxoacyl-[acyl-carrier-protein] synthase protein FabH	911.0	189
190	MGCS36044_02598			*rfbC*	dTDP-4-dehydrorhamnose 3,5-epimerase RfbC	908.5	190
191	MGCS36044_01548			*glgA*	glycogen synthase GlgA	905.0	191
192	MGCS36044_01218			*dhaM*	PTS-dependent dihydroxyacetone kinase	904.3	192
193	MGCS36044_01638			*ebsA*	EbsA family pore-forming protein	902.8	193
194	MGCS36044_00604			*rplM*	50S ribosomal L13 protein RplM	901.5	194
195	MGCS36044_01078			*rplK*	50S ribosomal L11P protein RplK	893.0	195
196	MGCS36044_03454	Secreted		-	pilus ancillary/minor protein 1	892.3	196
197	MGCS36044_00206			*rplO*	50S ribosomal L15 protein RplO	888.3	197
198	MGCS36044_03944		Virulence	*perR*	peroxide-responsive transcriptional repressor	877.8	198
199	MGCS36044_03638			-	Asp23/Gls24 family envelope stress response protein	871.0	199
200	MGCS36044_01640			-	LysM peptidoglycan-binding domain-containing protein	864.3	200
201	MGCS36044_02654			*malG*	maltose/maltodextrin ABC transport system permease protein MalG	860.5	201
202	MGCS36044_01408			*kgdA*	bifunctional 4-hydroxy-2-oxoglutarate (KHG)	854.8	202

No.	Locus tag	Signal6P predicted[1]	Virulence[2]	Gene	Function	RPKMs[3]	RANK[4]
203	MGCS36044_02632			-	glutathione S-transferase N-terminal	850.5	203
204	MGCS36044_01406			*kdgK*	2-dehydro-3-deoxygluconokinase KdgK	849.8	204
205	MGCS36044_02676			*dltX*	teichoic acid D-Ala incorporation-associated	849.3	205
206	MGCS36044_03816	Secreted		*prgA*	surface exclusion domain-containing secreted	841.0	206
207	MGCS36044_01648			*rpmI*	50S ribosomal L35 protein RpmL	840.0	207
208	MGCS36044_04018			*groEL*	chaperonin GroEL	837.0	208
209	MGCS36044_04202			*rpsD*	30S ribosomal S4 protein RpsD	836.3	209
210	MGCS36044_02378			-	ABC transporter ATP-binding protein	836.0	210
211	MGCS36044_04144			*rpmGA*	50S ribosomal L33 protein RpmGA. Region of difference 36044_ROD.9, putative MGE	834.0	211
212	MGCS36044_00316			*rpoC*	DNA-directed RNA polymerase subunit beta' RpoC	832.8	212
213	MGCS36044_00766			-	DAK2 domain-containing protein	832.0	213
214	MGCS36044_02294			*pepN*	lysyl aminopeptidase/alanine aminopeptidase	825.3	214
215	MGCS36044_03538			*phaB*	enoyl-CoA hydratase protein PhaB	820.8	215
216	MGCS36044_03640			*efp*	translation elongation factor (P) Efp	820.8	215
217	MGCS36044_03520			*fabZ*	3-hydroxyacyl-ACP dehydratase FabZ	815.8	217
218	MGCS36044_03540			*dnaJ*	chaperone protein DnaJ	803.3	218
219	MGCS36044_03652			*rpsR*	30S ribosomal S18 protein RpsR	799.3	219
220	MGCS36044_02596			*rfbB*	dTDP-glucose 4,6-dehydratase RfbB	794.3	220
221	MGCS36044_02908	Lipo		*prsA*	peptidylprolyl isomerase lipoprotein PrsA	790.8	221
222	MGCS36044_02476			*manZ*	ManZ family PTS mannose/fructose IID component	787.0	222
223	MGCS36044_03036			*clpA*	ATP-dependent Clp protease ATP-binding subunit ClpA	782.8	223
224	MGCS36044_00450			-	hypothetical protein	777.5	224
225	MGCS36044_03382			*tkt*	transketolase Tkt	772.0	225
226	MGCS36044_03524			*fabF*	3-oxoacyl-[acyl-carrier-protein] synthase	765.0	226
227	MGCS36044_00166			*rpsJ*	30S ribosomal S10 protein RpsJ	764.3	227
228	MGCS36044_03300			*pknB*	Stk1 family PASTA domain-containing Ser/Thr	762.0	228
229	MGCS36044_01192			*pepQ*	Xaa-Pro dipeptidase protein PepQ	754.3	229
230	MGCS36044_02478			*manY*	ManY family PTS mannose/fructose IIC component	749.8	230
231	MGCS36044_02544			*acoL*	dihydrolipoyl dehydrogenase AcoL	748.3	231
232	MGCS36044_03136			-	B3/4 domain-containing protein	746.5	232
233	MGCS36044_03440			*copA_2*	copper-exporting ATPase CopA	745.5	233
234	MGCS36044_01714			*rpsP*	30S ribosomal S16 protein RpsP	745.0	234
235	MGCS36044_01190			-	AD(P)H-dependent oxidoreductase	744.3	235
236	MGCS36044_02448			*pepV*	dipeptidase PepV	744.0	236
237	MGCS36044_01188			*gloA*	lactoylglutathione lyase protein GloA	743.8	237
238	MGCS36044_03516			*accA*	acetyl-CoA carboxylase, carboxyltransferase beta	743.8	237
239	MGCS36044_00798	Lipo		*oppA_1*	oligopeptide ABC transporter substrate-binding	741.8	239
240	MGCS36044_00336			*ackA*	acetate kinase AckA	741.0	240
241	MGCS36044_01682			*rpmA*	50S ribosomal L27 protein RpmA	736.3	241
242	MGCS36044_04274		Virulence	*htrA*	trypsin-like serine protease HtrA	734.8	242
243	MGCS36044_00750			*lrgA*	antiholin-like murein hydrolase modulator LrgA	733.5	243

No.	Locus tag	Signal6P predicted[(1)]	Virulence[(2)]	Gene	Function	RPKMs[(3)]	RANK[(4)]
244	MGCS36044_00364	Secreted		-	secreted pilin backbone/major protein	730.5	244
245	MGCS36044_02882			-	PgdA-like putative PG GlcNAc deacetylase	730.5	244
246	MGCS36044_01430			*ftsX*	cell division permease-like protein FtsX	729.3	246
247	MGCS36044_01728			*apbA*	2-dehydropantoate 2-reductase	720.3	247
248	MGCS36044_01216			*dhaL*	dihydroxyacetone kinase subunit DhaL	720.0	248
249	MGCS36044_03584			*alaT*	AlaT family aminotransferase	719.5	249
250	MGCS36044_03514			*accD*	acetyl-CoA carboxylase carboxyl transferase	716.8	250
251	MGCS36044_03532			*acpP_2*	acyl carrier protein AcpP	711.0	251
252	MGCS36044_02288			*rpsT*	30S ribosomal S20 protein RpsT	707.5	252
253	MGCS36044_02332			-	GntR family transcriptional regulator	707.5	252
254	MGCS36044_00530			*msmK*	sn-glycerol-3-phosphate ABC transporter	702.5	254
255	MGCS36044_03636			*nusB*	transcription termination protein NusB	699.0	255
256	MGCS36044_00104	Secreted		*sibA*	CHAP domain-containing protein/secreted	698.3	256
257	MGCS36044_02150			-	cupin domain-containing protein	697.8	257
258	MGCS36044_02480			-	AgaB family protein	696.0	258
259	MGCS36044_01678			*rplU*	50S ribosomal L21 protein RplU	688.0	259
260	MGCS36044_00516	Secreted	Virulence	*emm*	cell surface M protein Emm	683.8	260
261	MGCS36044_00966			*upp*	uracil phosphoribosyltransferase Upp	683.3	261
262	MGCS36044_00212			*infA*	translation initiation factor IF-1 protein InfA	679.5	262
263	MGCS36044_01546			*glgD*	glucose-1-phosphate adenylyltransferase subunit GlgD	675.0	263
264	MGCS36044_04090			*nrdD_2*	anaerobic ribonucleoside-triphosphate reductase NrdD	671.0	264
265	MGCS36044_04100			-	IreB-related regulatory phosphoprotein	670.0	265
266	MGCS36044_03330			*mapZ*	MapZ family cell division site-positioning	662.5	266
267	MGCS36044_02652			*malF*	maltose/maltodextrin ABC transport system permease protein MalF	658.5	267
268	MGCS36044_00968			*clpP*	ATP-dependent Clp protease proteolytic subunit	657.8	268
269	MGCS36044_01544			*glgC*	glucose-1-phosphate adenylyltransferase subunit	656.3	269
270	MGCS36044_01338			-	ECF transporter S component	652.8	270
271	MGCS36044_03526			*fabG_2*	3-ketoacyl-(acyl-carrier-protein) reductase	652.5	271
272	MGCS36044_03426			*lacB*	galactose-6-phosphate isomerase subunit LacB	650.0	272
273	MGCS36044_03542			-	PfpI family predicted protease/amidase	639.3	273
274	MGCS36044_01660			-	YlbF/YmcA family competence regulator	637.5	274
275	MGCS36044_01400			*agaF*	PTS transporter hyaluronate-oligosaccharide-specific IIA component AgaF	637.0	275
276	MGCS36044_00314			*rpoB*	DNA-directed RNA polymerase subunit beta RpoB	634.5	276
277	MGCS36044_03858			*rpmH*	50S ribosomal L34 protein RpmH	634.3	277
278	MGCS36044_02270			-	putative nucleoside ABC transporter permease	633.8	278
279	MGCS36044_01646			*infC*	translation initiation factor InfC	630.3	279
280	MGCS36044_03678			-	hypothetical protein	629.8	280
281	MGCS36044_01550			*atpE*	ATP synthase C subunit AtpE	628.0	281
282	MGCS36044_03424			*lacC*	tagatose-6-phosphate kinase LacC	625.8	282
283	MGCS36044_01404			*rpiB*	RpiB/LacA/LacB family sugar-phosphate isomerase	621.5	283

No.	Locus tag	Signal6P predicted[(1)]	Virulence[(2)]	Gene	Function	RPKMs[(3)]	RANK[(4)]
284	MGCS36044_01170			-	DUF853 domain-containing protein	616.5	284
285	MGCS36044_03486			-	chemotaxis protein	615.5	285
286	MGCS36044_02126			-	SpF66_sRNA	615.0	286
287	MGCS36044_03438			*copZ_2*	copper chaperone CopZ	614.5	287
288	MGCS36044_03528			*fabD*	malonyl CoA-acyl carrier protein transacylase	613.5	288
289	MGCS36044_00764			-	Asp23/Gls24 family envelope stress response	611.5	289
290	MGCS36044_03946			*yccU*	YccU family CoA-binding protein	603.5	290
291	MGCS36044_02232			*nrdD_1*	ATP cone domain-containing protein,	602.8	291
292	MGCS36044_01636			*pepT*	peptidase (T) PepT	602.3	292
293	MGCS36044_01302			*lgt*	prolipoprotein diacylglyceryl transferase Lgt	601.3	293
294	MGCS36044_03950			*polA*	DNA polymerase I PolA	600.8	294
295	MGCS36044_04020			*groES*	co-chaperone GroES	599.3	295
296	MGCS36044_02548			*acoC*	dihydrolipoamide acetyltransferase AcoC	594.8	296
297	MGCS36044_01428			*ftsE*	cell division ATP-binding protein FtsE	594.3	297
298	MGCS36044_03430			*sgcC*	galactitol-specific PTS transporter IIB subunit	591.3	298
299	MGCS36044_00902	Lipo		*yidC_1*	membrane protein insertase lipoprotein YidC	591.0	299
300	MGCS36044_02550			*acoB*	pyruvate dehydrogenase E1 component beta subunit	589.8	300
301	MGCS36044_03948			-	hypothetical protein	577.0	301
302	MGCS36044_03900			*galU*	UTP--glucose-1-phosphate uridylyltransferase	575.5	302
303	MGCS36044_03384			*fsa*	FSA family fructose-6-phosphate aldolase	573.5	303
304	MGCS36044_00908			*rnaY*	RnaY family HD domain-containing protein	571.5	304
305	MGCS36044_02272			-	putative nucleoside ABC transporter permease	570.5	305
306	MGCS36044_03622			*secA*	preprotein translocase subunit SecA	566.0	306
307	MGCS36044_03406			-	ARA1 family aldo/keto reductase	564.3	307
308	MGCS36044_01240		Virulence	*vicK*	TCS signal transduction sensor kinase VicK	558.0	308
309	MGCS36044_02214			-	LCB5 family diacylglycerol lipid kinase	553.3	309
310	MGCS36044_02266			*panT*	pantothenic acid transporter PanT	553.3	309
311	MGCS36044_02264			*coaC*	phosphopantothenoylcysteine decarboxylase CoaC	551.0	311
312	MGCS36044_00312			-	Lacto-rpoB	549.8	312
313	MGCS36044_02444			*rpiA*	ribose-5-phosphate isomerase RpiA	549.5	313
314	MGCS36044_01304			-	COG4768 superfamily YoxC-like protein	547.3	314
315	MGCS36044_02022			-	FolA superfamily dihydrofolate reductase	546.8	315
316	MGCS36044_01068			*mtsB*	metal ABC transporter ATP-binding protein MtsB	546.5	316
317	MGCS36044_03530			*fabK*	Enoyl-[acyl-carrier-protein] reductase protein	544.8	317
318	MGCS36044_04096			-	DUF1292 domain-containing protein	544.5	318
319	MGCS36044_00526	Secreted	Virulence	*ska*	secreted streptokinase Ska	542.3	319
320	MGCS36044_01300			*hprK*	HPr(Ser) kinase/phosphatase HprK	540.5	320
321	MGCS36044_02268			*pgmA*	phospho-sugar mutase PgmA	539.3	321
322	MGCS36044_03070			*typA*	translational GTPase TypA	539.0	322
323	MGCS36044_01968			*ribP*	ribose-phosphate pyrophosphokinase RibP	536.0	323
324	MGCS36044_01096			*mycA*	oleate hydratase/myosin-crossreactive antigen	535.8	324

No.	Locus tag	Signal6P predicted[(1)]	Virulence[(2)]	Gene	Function	RPKMs[(3)]	RANK[(4)]
325	MGCS36044_02528			*glmM*	phosphoglucosamine mutase GlmM	535.0	325
326	MGCS36044_03058			*ftsA*	cell division protein FtsA	534.3	326
327	MGCS36044_02718			-	DUF1846 domain-containing protein	526.0	327
328	MGCS36044_04260			*uup*	Uup family ATPase components of ABC transporters with duplicated ATPase domains	521.8	328
329	MGCS36044_00030			*ftsH*	ATP-dependent zinc metalloprotease FtsH	517.0	329
330	MGCS36044_01644			-	L20_leader RNA	510.0	330
331	MGCS36044_00948			*pflA*	pyruvate formate-lyase activating enzyme PflA	504.8	331
332	MGCS36044_03512			*serS*	seryl-tRNA synthetase SerS	504.3	332
333	MGCS36044_02314			*spxA_1*	transcriptional regulator SpxA	502.3	333
334	MGCS36044_03302			*pppL*	Stp1/IreP family PP2C-type Ser/Thr phosphatase	501.5	334
335	MGCS36044_01586			-	neutral zinc metallopeptidase	500.8	335
336	MGCS36044_00904			*acyP*	acylphosphatase AcyP	496.8	336
337	MGCS36044_03290			-	S1 RNA-binding domain-containing protein	494.8	337
338	MGCS36044_04106			-	hypothetical protein	493.8	338
339	MGCS36044_01642			*cmk*	CMP kinase Cmk	492.5	339
340	MGCS36044_02434			-	LytR family transcriptional regulator	492.3	340
341	MGCS36044_02450			*nfnB*	NfnB family nitroreductase	490.0	341
342	MGCS36044_01238		Virulence	*vicR*	TCS DNA-binding response regulator VicR	489.5	342
343	MGCS36044_02274			-	putative nucleoside ABC transporter ATP-binding	488.0	343
344	MGCS36044_01966			*nifS_2*	NifS superfamily cysteine desulfurase	487.5	344
345	MGCS36044_04040			*hutI*	imidazolonepropionase HutI	484.8	345
346	MGCS36044_03292			-	putative bifunctional	484.5	346
347	MGCS36044_01542			*glgB*	1,4-alpha-glucan branching protein GlgB	483.5	347
348	MGCS36044_02546			-	MGCS36044_02546	480.8	348
349	MGCS36044_04254			*guaB*	IMP dehydrogenase GuaB	478.5	349
350	MGCS36044_01604			*rpoD*	RNA polymerase sigma factor RpoD	476.5	350
351	MGCS36044_00106			*prs*	ribose-phosphate pyrophosphokinase PrsA	476.0	351
352	MGCS36044_00768			-	SPFH domain-containing protein	470.0	352
353	MGCS36044_01164			*coaE*	dephospho-CoA kinase CoaE	468.3	353
354	MGCS36044_00390			*purA*	adenylosuccinate synthase PurA	467.8	354
355	MGCS36044_01402			*idnO*	gluconate 5-dehydrogenase IdnO	466.0	355
356	MGCS36044_02538			*lplA_2*	lipoate--protein ligase LplA	465.8	356
357	MGCS36044_03664			-	FAD-containing oxidoreductase	465.8	356
358	MGCS36044_01538			*dagK*	diacylglycerol kinase family lipid kinase	464.0	358
359	MGCS36044_03342			*pbp1A*	bifunctional PG transglycosylase-transpeptidase	461.8	359
360	MGCS36044_03464			*infB*	translation initiation factor IF-2	461.8	359
361	MGCS36044_00004			*dnaN*	DNA polymerase III subunit beta protein DnaN	460.8	361
362	MGCS36044_02430			-	hypothetical protein	460.0	362
363	MGCS36044_01104			*dgkA*	diacylglycerol kinase DgkA	459.0	363
364	MGCS36044_01102			*ybeY*	rRNA maturation RNase YbeY	458.3	364
365	MGCS36044_01108			-	Nudix superfamily phosphohydrolase	456.8	365

No.	Locus tag	Signal6P predicted[(1)]	Virulence[(2)]	Gene	Function	RPKMs[(3)]	RANK[(4)]
366	MGCS36044_01086			*frr*	ribosome recycling factor Frr	452.8	366
367	MGCS36044_03422			*lacD_2*	tagatose-bisphosphate aldolase LacD	452.8	366
368	MGCS36044_03428			*lacA*	galactose-6-phosphate isomerase subunit LacA	451.0	368
369	MGCS36044_01214			*dhaK*	dihydroxyacetone kinase subunit DhaK	450.0	369
370	MGCS36044_01070			*mtsC*	metal ABC transporter permease MtsC	446.5	370
371	MGCS36044_03684			-	ElaA-related predicted N-acetyltransferase	444.5	371
372	MGCS36044_03072			*pspE*	PspE family rhodanese-like domain-containing	444.0	372
373	MGCS36044_03786			*rnjA_2*	mRNA degradation ribonuclease RnjA	443.3	373
374	MGCS36044_04224			*mnmA*	tRNA 2-thiouridine(34) synthase MnmA	443.3	373
375	MGCS36044_02574	Secreted		*aphA*	secreted acid phosphatase AphA	440.5	375
376	MGCS36044_02920			*pepF_2*	oligoendopeptidase (F) PepF	439.5	376
377	MGCS36044_03038			-	NUDIX hydrolase	438.3	377
378	MGCS36044_02036	Secreted		-	DUF1002 domain-containing putative secreted	437.3	378
379	MGCS36044_01320			*lysS*	lysyl-tRNA synthetase LysS	435.5	379
380	MGCS36044_02892			*nrdE_2*	class 1b ribonucleoside-diphosphate reductase alpha subunit NrdE	433.5	380
381	MGCS36044_02128			*guaA*	glutamine-hydrolyzing GMP synthase	432.8	381
382	MGCS36044_00950			*ppaC*	manganese-dependent inorganic pyrophosphatase	431.8	382
383	MGCS36044_04064			*pepO*	endopeptidase PepO	431.8	382
384	MGCS36044_00888			*der*	ribosome biogenesis GTPase Der	430.3	384
385	MGCS36044_04128			*argS*	arginine--tRNA synthase ArgS	428.5	385
386	MGCS36044_01062		Virulence	*mtsR*	metal-dependent transcriptional regulator MtsR	425.3	386
387	MGCS36044_01982			-	Na+ driven multidrug efflux pump	424.0	387
388	MGCS36044_01100			-	uracil DNA glycosylase superfamily protein	422.8	388
389	MGCS36044_02678			*uvrB*	excinuclease ABC subunit UvrB	422.0	389
390	MGCS36044_01764			*sptS*	SptS-like TCS sensor histidine kinase	420.3	390
391	MGCS36044_01540			*pulA_2*	type I pullulanase PulA	417.8	391
392	MGCS36044_01200			-	glycosyltransferase	417.5	392
393	MGCS36044_03442			*copY_2*	DNA-binding copper transport transcriptional repressor CopY	416.3	393
394	MGCS36044_04108			*recA*	recombinase RecA	416.3	393
395	MGCS36044_01274			-	NUDIX hydrolase superfamily protein	413.0	395
396	MGCS36044_02834			*map*	methionyl aminopeptidase Map	408.5	396
397	MGCS36044_02872			-	GAF domain containing protein	408.5	396
398	MGCS36044_02714			-	putative thioesterase	405.0	398
399	MGCS36044_02364			*glmS*	glutamine--fructose-6-phosphate transaminase	404.8	399
400	MGCS36044_02552			*acoA*	Pyruvate dehydrogenase E1 component alpha	404.8	399
401	MGCS36044_00484			*rarA*	replication-associated recombination protein A	402.8	401
402	MGCS36044_03898			*gpsA*	NAD(P)H-dependent glycerol-3-phosphate	402.5	402
403	MGCS36044_03212	Lipo		*ugpB_1*	glycerol-3-phosphate ABC transporter	400.0	403
404	MGCS36044_01106			*era*	GTPase Era	399.0	404
405	MGCS36044_03432			*sgaB*	galactitol-specific PTS transporter IIC subunit	399.0	404
406	MGCS36044_03478			*cotS*	CotS family thiamine kinase	398.8	406

No.	Locus tag	Signal6P predicted[(1)]	Virulence[(2)]	Gene	Function	RPKMs[(3)]	RANK[(4)]
407	MGCS36044_03818			*purR*	pur operon repressor PurR	398.5	407
408	MGCS36044_03362			*cshB*	DEAD/DEAH box helicase	398.0	408
409	MGCS36044_01426			*prfB*	peptide chain release factor 2 PrfB	395.3	409
410	MGCS36044_00732			-	DUF436 family protein	393.3	410
411	MGCS36044_00540			*yajC*	preprotein translocase subunit YajC	392.8	411
412	MGCS36044_00880		Virulence	*covS*	TCS sensor kinase CovS	391.0	412
413	MGCS36044_03826			*rpe*	ribulose-phosphate 3-epimerase Rpe	390.3	413
414	MGCS36044_00804			*oppD_1*	oligopeptide ABC transporter permease protein	389.0	414
415	MGCS36044_03552			-	LD/DD carboxypeptidase family protein	389.0	414
416	MGCS36044_02290		Virulence	*ciaH*	TCS sensor histidine kinase protein CiaH	388.3	416
417	MGCS36044_02638			-	hypothetical protein	387.8	417
418	MGCS36044_03390			*glpF_1*	GlpF family glycerol uptake facilitator and	386.3	418
419	MGCS36044_04276			*parB*	chromosome partitioning protein ParB	386.3	418
420	MGCS36044_01726			-	PTS transporter subunit IIC	382.8	420
421	MGCS36044_02870			*dnaX*	DNA polymerase III gamma/tau subunit DnaX	382.0	421
422	MGCS36044_03000			-	DegV family EDD domain-containing protein	381.0	422
423	MGCS36044_02708			*pepS*	aminopeptidase PepS	380.8	423
424	MGCS36044_03134			*argR_2*	arginine responsive transcriptional repressor	379.3	424
425	MGCS36044_01256			*ftsY*	signal recognition particle-docking protein	378.8	425
426	MGCS36044_01474			*nrnA*	bifunctional oligoribonuclease/PAP phosphatase	378.5	426
427	MGCS36044_02536			-	CobQ-like type 1 glutamine amidotransferase	377.5	427
428	MGCS36044_01456			*lacD_1*	tagatose-bisphosphate aldolase LacD-like	377.0	428
429	MGCS36044_02146			-	TCS DNA-binding response regulator (heme	376.8	429
430	MGCS36044_00552			*polC*	DNA polymerase III PolC	376.3	430
431	MGCS36044_03346			*nadE*	ammonia-dependent NAD(+) synthetase NadE	376.0	431
432	MGCS36044_03348			*pncB*	nicotinate phosphoribosyltransferase PncB	376.0	431
433	MGCS36044_02988			*zntA*	ZntA family P-type heavy metal transporter	375.5	433
434	MGCS36044_02140			-	FAD-binding oxidoreductase	374.3	434
435	MGCS36044_03098	Lipo		*rbsB*	D-ribose ABC transporter substrate-binding	372.8	435
436	MGCS36044_01094	Secreted		*lysM*	LysM peptidoglycan-binding domain-containing	371.5	436
437	MGCS36044_00848			-	YebC/PmpR family DNA-binding transcriptional	370.8	437
438	MGCS36044_03484			*hit*	HIT family protein	369.3	438
439	MGCS36044_00806			*oppF_1*	oligopeptide ABC transporter ATP-binding protein	368.8	439
440	MGCS36044_03860			*jag*	RNA-binding protein Jag	366.5	440
441	MGCS36044_04222			*marC*	MarC family small neutral amino acid	366.5	440
442	MGCS36044_01308			-	DUF3270 domain-containing protein	365.0	442
443	MGCS36044_01606			-	metal-sulfur cluster assembly factor	364.5	443
444	MGCS36044_02212		Virulence	*yqfA*	membrane channel forming/hemolysin III protein	363.0	444
445	MGCS36044_02832			*brkB*	BrkB family protein	361.3	445
446	MGCS36044_03252			-	DUF402 domain-containing protein	360.8	446
447	MGCS36044_04066			*treC*	trehalose-6-phosphate hydrolase TreC	360.0	447

No.	Locus tag	Signal6P predicted[(1)]	Virulence[(2)]	Gene	Function	RPKMs[(3)]	RANK[(4)]
448	MGCS36044_03560			*gatB_2*	aspartyl-tRNA(Asn) or glutamyl-tRNA(Gln)	359.8	448
449	MGCS36044_03874			*gltX*	glutamate--tRNA ligase	359.3	449
450	MGCS36044_02586			-	tetra tricopeptide repeat family protein	358.8	450
451	MGCS36044_01986			*osmF*	OsmF superfamily glycine/betaine transport	358.3	451
452	MGCS36044_02376			*yhcF*	YhcF family transcriptional regulator	358.3	451
453	MGCS36044_01500			*ezrA*	cell division septation ring formation regulator	354.3	453
454	MGCS36044_02482			*manX*	ManX family PTS mannose/fructose IIA component	354.0	454
455	MGCS36044_00578			*cysE*	serine O-acetyltransferase CysE	351.8	455
456	MGCS36044_01296			-	SprT family protein	351.3	456
457	MGCS36044_02996			-	DUF2140 domain-containing protein	351.3	456
458	MGCS36044_03310			*rpoZ*	DNA-directed RNA polymerase omega subunit RpoZ	349.8	458
459	MGCS36044_00302			*adcR*	zinc-dependent MarR family transcriptional	348.0	459
460	MGCS36044_01794			*engB*	ribosome biogenesis GTP-binding protein EngB	347.5	460
461	MGCS36044_01024			-	hypothetical protein	344.5	461
462	MGCS36044_03934			-	metallo-beta-lactamase superfamily protein	344.0	462
463	MGCS36044_00494			*rsmE*	16S rRNA (uracil(1498)-N(3))-methyltransferase	342.5	463
464	MGCS36044_00532			*dexB*	glucan 1,6-alpha-glucosidase DexB	342.5	463
465	MGCS36044_02484			*yeiH*	YeiH family membrane protein	342.5	463
466	MGCS36044_01598			*rpsU*	30S ribosomal S21 protein RpsU	339.5	466
467	MGCS36044_00876			*yceD*	large ribosomal RNA subunit accumulation protein	338.8	467
468	MGCS36044_03488			-	Cps2a family anionic cell wall polymer	338.8	467
469	MGCS36044_03388			-	FadH2 family NAD(FAD)-dependent dehydrogenase	337.5	469
470	MGCS36044_01198			-	glycosyltransferase	336.3	470
471	MGCS36044_03828			*rsgA*	ribosome small subunit-dependent GTPase (A)	335.5	471
472	MGCS36044_01608			*rmlD*	dTDP-4-dehydrorhamnose reductase protein RmlD	335.0	472
473	MGCS36044_02292		Virulence	*ciaR*	TCS DNA-binding response regulator protein CiaR	333.8	473
474	MGCS36044_04112			-	VOC family protein	333.0	474
475	MGCS36044_00830			*nadD*	nicotinate-nucleotide adenylyltransferase NadD	332.8	475
476	MGCS36044_03866		Virulence	*fasX*	FasBCAX signal transduction system small RNA	332.3	476
477	MGCS36044_00308			*tyrS*	tyrosyl-tRNA synthetase TyrS	331.0	477
478	MGCS36044_01048			-	ASCH domain-containing RNA-binding protein	331.0	477
479	MGCS36044_01384			*mgtA*	MgtA superfamily cation-translocating P-type	330.8	479
480	MGCS36044_01568			*murA_1*	UDP-N-acetylglucosamine	327.3	480
481	MGCS36044_01438			*asnC*	asparaginyl-tRNA synthetase protein AsnC	327.0	481
482	MGCS36044_01602			*dnaG*	DNA primase protein DnaG	327.0	481
483	MGCS36044_00576			-	polynucleotide phosphorylase/polyadenylase	326.5	483
484	MGCS36044_02998			-	SGNH-hydrolase superfamily of lipases and	326.0	484
485	MGCS36044_00300			*ipk*	4-diphosphocytidyl-2-C-methyl-D-erythritol kinase Ipk	325.5	485
486	MGCS36044_00594			-	DegV family protein	325.3	486

No.	Locus tag	Signal6P predicted[(1)]	Virulence[(2)]	Gene	Function	RPKMs[(3)]	RANK[(4)]
487	MGCS36044_01268			-	DUF1980 dopmain-containing protein	325.3	486
488	MGCS36044_03626			*manA*	mannose-6-phosphate isomerase ManA	324.3	488
489	MGCS36044_01270			-	permease	321.8	489
490	MGCS36044_01960			*rex*	redox-sensing transcriptional repressor Rex	320.5	490
491	MGCS36044_03974			*leuS*	leucine--tRNA synthase LeuS	320.5	490
492	MGCS36044_02588			*perM*	PerM family predicted purR regulated permease	318.5	492
493	MGCS36044_02262			*coaB*	phosphopantothenate--cysteine ligase CoaB	318.3	493
494	MGCS36044_01590			*rexA*	ATP-dependent nuclease A subunit RexA	316.0	494
495	MGCS36044_03386			-	putative transcriptional regulator	316.0	494
496	MGCS36044_03476			*trmB*	tRNA (guanosine(46)-N7)-methyltransferase TrmB	315.8	496
497	MGCS36044_00522			*dtd*	D-tyrosyl-tRNA deacylase Dtd	315.3	497
498	MGCS36044_02614			*apt*	adenine phosphoribosyltransferase Apt	313.3	498
499	MGCS36044_04102			-	SSRC41 RNA	313.3	498
500	MGCS36044_03562			*gatA_2*	aspartyl-tRNA(Asn) or glutamyl-tRNA(Gln) amidotransferase A subunit GatA	313.0	500
501	MGCS36044_03666			*mutS2*	DNA mismatch repair endonuclease MutS2	312.8	501
502	MGCS36044_03392			*glpO*	type 1 glycerol-3-phosphate oxidase GlpO	312.3	502
503	MGCS36044_03408			*nagA*	N-acetylglucosamine-6-phosphate deacetylase	312.3	502
504	MGCS36044_01044			*fabG_1*	3-oxoacyl-ACP reductase FabG	311.5	504
505	MGCS36044_03588			-	HAD-related haloacid dehalogenase hydrolase	311.5	504
506	MGCS36044_01162			*mutM*	DNA-formamidopyrimidine glycosylase MutM	310.5	506
507	MGCS36044_04022			*clpC*	ATP-dependent Clp protease ATP-binding subunit ClpC	310.5	506
508	MGCS36044_03550			-	FlgJ-related putative peptidoglycan hydrolase	310.0	508
509	MGCS36044_03882			-	AIM24 family protein	308.8	509
510	MGCS36044_03376			-	hypothetical protein	308.5	510
511	MGCS36044_02894			*nrdF_2*	class 1b ribonucleoside-diphosphate reductase beta subunit NrdF	308.3	511
512	MGCS36044_03170			*valS*	valine--tRNA synthetase ValS	307.8	512
513	MGCS36044_00730			-	ECF transporter S component	307.5	513
514	MGCS36044_00574			*pnp*	polyribonucleotide nucleotidyltransferase Pnp	307.0	514
515	MGCS36044_01484			*fldA*	flavodoxin FldA	307.0	514
516	MGCS36044_01412			-	hypothetical protein	306.8	516
517	MGCS36044_00152			*oatA*	acetyltransferase OatA	306.5	517
518	MGCS36044_02540			-	DUF4097 family beta strand repeat-containing	306.5	517
519	MGCS36044_00518	Secreted		-	cell surface UshA family bifunctional	305.8	519
520	MGCS36044_01046			-	DUF3977 family protein	305.8	519
521	MGCS36044_03764			*ybaB*	YbaB family DNA-binding protein	305.8	519
522	MGCS36044_01744			*degV_1*	DegV family protein	305.5	522
523	MGCS36044_01964			-	DUF1831 domain-containing protein	305.0	523
524	MGCS36044_01476			-	GNAT family N-acetyltransferase	304.3	524
525	MGCS36044_01712			-	glycerophosphodiester phosphodiesterase	303.8	525
526	MGCS36044_03314			*rny*	ribonuclease (Y) Rny	303.5	526
527	MGCS36044_03312			*gmk*	guanylate kinase Gmk	303.3	527

No.	Locus tag	Signal6P predicted[(1)]	Virulence[(2)]	Gene	Function	RPKMs[(3)]	RANK[(4)]
528	MGCS36044_01050			-	Gfo/Idh/MocA family oxidoreductase	302.8	528
529	MGCS36044_03940			*tgt*	tRNA guanosine(34) transglycosylase Tgt	302.5	529
530	MGCS36044_03060			*ftsQ*	cell division protein FtsQ/DivIB	301.5	530
531	MGCS36044_04086			*mviM*	MviM family predicted dehydrogenase	301.5	530
532	MGCS36044_03110			*sdrC*	SdrC family PDZ domain-containing protein	300.5	532
533	MGCS36044_01652			*ltaS*	LTA synthase LtaS	300.3	533
534	MGCS36044_01962			-	DUF4649 domain-containing protein	299.5	534
535	MGCS36044_00548			*proS*	prolyl-tRNA synthetase ProS	299.3	535
536	MGCS36044_03020			-	phospho-sugar mutase	298.5	536
537	MGCS36044_02986			-	Spy1186876 RNA	298.0	537
538	MGCS36044_01536			*ligA*	NAD-dependent DNA ligase LigA	297.3	538
539	MGCS36044_00414			*pgpA*	phosphatidylglycerophosphatase protein PgpA	296.0	539
540	MGCS36044_00832			*yqeK*	bis(5'-nucleosyl)-tetraphosphatase (symmetrical)	295.5	540
541	MGCS36044_00996			*rsmI*	16S rRNA (cytidine(1402)-2'-O)-methyltransferase	294.8	541
542	MGCS36044_01084			*pyrH*	UMP kinase PyrH	293.8	542
543	MGCS36044_02148			-	TCS histidine kinase sensor	293.8	542
544	MGCS36044_03044			*ileS*	isoleucine--tRNA synthetase IleS	293.8	542
545	MGCS36044_00912			*yneF*	YneF family protein	292.3	545
546	MGCS36044_01344			*pepF_1*	oligoendopeptidase PepF	291.8	546
547	MGCS36044_00742			*rpoE*	DNA-directed RNA polymerase subunit delta RpoE	291.3	547
548	MGCS36044_00150			-	hypothetical protein	290.3	548
549	MGCS36044_00002			*dnaA*	chromosomal replication initiator protein DnaA	289.3	549
550	MGCS36044_01352			-	sigma factor regulator	289.0	550
551	MGCS36044_02656			*malH*	LacI family transcriptional regulatory protein	288.8	551
552	MGCS36044_02282			*deoA*	pyrimidine-nucleoside phosphorylase DeoA	287.8	552
553	MGCS36044_03034			-	DUF1797 family protein	287.8	552
554	MGCS36044_03062			*murG*	UDP-N-acetylglucosamine--N-acetylmuramyl-	287.8	552
555	MGCS36044_00310			*pbp1B*	bifunctional PG transglycosylase-transpeptidase,	287.5	555
556	MGCS36044_03554			-	PhoE family broad specificity phosphatase	287.3	556
557	MGCS36044_00028			-	hypoxanthine-guanine phosphoribosyltransferase	285.8	557
558	MGCS36044_01790			-	hypothetical protein	284.0	558
559	MGCS36044_00886			*dnaI*	primosomal protein DnaI	283.0	559
560	MGCS36044_00558			*def*	peptide deformylase Def	282.8	560
561	MGCS36044_00920			-	CBS domain-containing protein	281.8	561
562	MGCS36044_02560			*rnjA_1*	mRNA degradation ribonuclease RnjA	281.8	561
563	MGCS36044_03418			-	Cof-type HAD-IIB family phosphohydrolase	281.3	563
564	MGCS36044_01570			*epuA*	DNA-directed RNA polymerase beta subunit EpuA	281.0	564
565	MGCS36044_01984			*opuBA*	OpuBA superfamily glycine/betaine ABC	281.0	564
566	MGCS36044_03470			*nusA*	transcription termination factor NusA	280.3	566
567	MGCS36044_00728			*thiD*	bifunctional hydroxymethylpyrimidine	279.8	567
568	MGCS36044_02960			-	Pleckstrin homology-like domain-containing	278.8	568

No.	Locus tag	Signal6P predicted[(1)]	Virulence[(2)]	Gene	Function	RPKMs[(3)]	RANK[(4)]
569	MGCS36044_01762			*sptR*	SptR-like TCS DNA-binding response regulator	278.3	569
570	MGCS36044_00800			*oppB_1*	oligopeptide ABC transporter permease protein	277.8	570
571	MGCS36044_00898			*mltG*	endolytic transglycosylase MltG	277.8	570
572	MGCS36044_00834			-	cysteine hydrolase	277.5	572
573	MGCS36044_02880			*srmB*	SrmB superfamily II DNA and RNA helicase	276.8	573
574	MGCS36044_04210			*rplI*	50S ribosomal L9 protein RplI	276.8	573
575	MGCS36044_00304			*adcC*	metal ABC transporter ATP-binding protein AdcC	276.5	575
576	MGCS36044_00802			*oppC_1*	oligopeptide ABC transporter permease protein	276.3	576
577	MGCS36044_00110			*plsX*	phosphate acyltransferase PlsX	275.5	577
578	MGCS36044_03820			*cbf*	YhaM family 3'-5' exoribonuclease	274.8	578
579	MGCS36044_00344			*proC*	pyrroline-5-carboxylate reductase ProC	274.5	579
580	MGCS36044_04010			*rluA_2*	RluA family pseudouridine synthase	274.5	579
581	MGCS36044_03704			*glpF_2*	glycerol uptake facilitator GlpF	273.8	581
582	MGCS36044_02846			-	SDR family D-mannonate oxidoreductase	273.3	582
583	MGCS36044_03352			*trxB_2*	thioredoxin-disulfide reductase TrxB	270.8	583
584	MGCS36044_01342			*btuE*	BtuE superfamily glutathione peroxidase	270.0	584
585	MGCS36044_01424			*queG*	epoxyqueuosine reductase QueG	269.3	585
586	MGCS36044_02762			-	helix-turn-helix domain-containing putative	268.0	586
587	MGCS36044_03340			*recU*	Holliday junction resolvase RecU	268.0	586
588	MGCS36044_03798			*glnA*	glutamine synthetase GlnA	267.5	588
589	MGCS36044_00306			*adcB*	metal ABC transporter permease AdcB	266.3	589
590	MGCS36044_03582			*codY*	CodY family GTP-sensing pleiotropic	266.3	589
591	MGCS36044_00346			*pepA*	glutamyl aminopeptidase PepA	265.5	591
592	MGCS36044_03420			-	YadS family trimeric intracellular cation	265.5	591
593	MGCS36044_02720			*fetB*	iron export ABC transporter permease subunit	264.3	593
594	MGCS36044_01196			*amyS*	alpha-amylase AmyS	263.8	594
595	MGCS36044_01498			*gyrB*	DNA topoisomerase ATP-hydrolyzing B subunit	262.5	595
596	MGCS36044_02028			*srtA*	class A sortase SrtA	262.5	595
597	MGCS36044_01436			*aspC*	aspartate aminotransferase protein AspC	262.3	597
598	MGCS36044_01892			*cadA*	cadmium-translocating P-type ATPase CadA	262.0	598
599	MGCS36044_03466			-	YlxQ-related RNA-binding protein	261.3	599
600	MGCS36044_00556			-	NAD(P)H-dependent oxidoreductase	260.8	600
601	MGCS36044_04114			*tag*	DNA-3-methyladenine glycosylase Tag	260.5	601
602	MGCS36044_02848			*uxuA*	mannonate dehydratase	260.3	602
603	MGCS36044_03004			*recN*	DNA repair protein RecN	260.0	603
604	MGCS36044_02066			-	hypothetical protein	258.8	604
605	MGCS36044_02904			*alaS*	alanine--tRNA synthetase AlaS	258.0	605
606	MGCS36044_02296			*phoU_2*	phosphate signaling complex protein PhoU	257.5	606
607	MGCS36044_04242			*rodZ*	cytoskeletal protein RodZ	257.0	607
608	MGCS36044_02298			*ptsB1*	phosphate ABC transporter ATP-binding protein PstB1	256.5	608
609	MGCS36044_03462			*rbfA*	30S ribosome-binding factor RbfA	256.5	608

No.	Locus tag	Signal6P predicted(1)	Virulence(2)	Gene	Function	RPKMs(3)	RANK(4)
610	MGCS36044_03800			*glnR*	glutamine synthetase transcriptional repressor	256.5	608
611	MGCS36044_00498			*ptsG*	PTS glucose-specific II ABC component	255.8	611
612	MGCS36044_03726			-	GNAT family acetyltransferase	255.8	611
613	MGCS36044_02496			*lepA*	translation elongation factor 4 LepA	255.5	613
614	MGCS36044_01792			*clpX*	ATP-dependent Clp protease, ATP-binding subunit	255.3	614
615	MGCS36044_02590			*mutX*	8-oxo-dGTP diphosphatase MutX	255.3	614
616	MGCS36044_03932			*tadA*	tRNA adenosine(34) deaminase TadA	255.3	614
617	MGCS36044_00520			*relA*	bifunctional (p)ppGpp synthase/hydrolase RelA	255.0	617
618	MGCS36044_00998			-	DUF5684 domain-containing protein	254.0	618
619	MGCS36044_01098			*phoH*	phosphate starvation-inducible protein PhoH	252.8	619
620	MGCS36044_03356			-	GlnQ family polar amino acid ABC transporter	252.8	619
621	MGCS36044_01958			-	gamma-glutamyl-gamma-aminobutyrate hydrolase	252.5	621
622	MGCS36044_03824			*thiN*	thiamine diphosphokinase ThiN	252.5	621
623	MGCS36044_01374			*murM*	peptidoglycan lipid II--L-alanine ligase protein	252.3	623
624	MGCS36044_00972			-	YlbF family regulatory protein	251.8	624
625	MGCS36044_01072	Lipo		-	peptidylprolyl isomerase lipoprotein	251.5	625
626	MGCS36044_00112			*acpP_1*	acyl carrier protein AcpP	251.3	626
627	MGCS36044_03112			*coaD*	pantetheine-phosphate adenylyltransferase CoaD	251.3	626
628	MGCS36044_03618			*alr*	alanine racemase Alr	251.0	628
629	MGCS36044_02144			*yhhX*	PRK10206 superfamily putative oxidoreductase. Region of difference 36044_ROD.6, putative MGE	250.3	629
630	MGCS36044_02646			*malR*	maltose operon transcriptional repressor MalR	249.8	630
631	MGCS36044_00918			-	YfcE family metallophosphoesterase	249.0	631
632	MGCS36044_03332			*rlmL*	23S rRNA G2445 N2-methylase RlmL	248.8	632
633	MGCS36044_02710			-	CorA family divalent cation transport protein	247.5	633
634	MGCS36044_03730			-	ABC transporter ATP-binding protein	247.0	634
635	MGCS36044_00894			*murC*	UDP-N-acetylmuramate--L-alanine ligase MurC	246.5	635
636	MGCS36044_03670			-	hypothetical protein	246.5	635
637	MGCS36044_00492			*prmA*	50S ribosomal protein L11 methyltransferase	246.0	637
638	MGCS36044_03728			-	ABC transporter permease	246.0	637
639	MGCS36044_01480			*add*	adenosine deaminase Add	245.8	639
640	MGCS36044_03620			*acpS*	AcpS family provisional 4'-phosphopantetheinyl	245.5	640
641	MGCS36044_00922			*xerD_1*	site-specific tyrosine recombinase XerD	245.0	641
642	MGCS36044_02136			*ffh*	signal recognition particle protein	244.8	642
643	MGCS36044_01922			*folE*	GTP cyclohydrolase I protein FolE	244.5	643
644	MGCS36044_03150			-	CorA family divalent cation transport protein	244.5	643
645	MGCS36044_02534			*murE_2*	UDP-N-acetylmuramoylalanyl-D-glutamate-2,	244.3	645
646	MGCS36044_01920			*folC*	dihydrofolate synthase FolC	243.8	646
647	MGCS36044_03668			-	colicin V production family protein	243.3	647
648	MGCS36044_02056	Lipo		-	TlpA-family protein	242.8	648
649	MGCS36044_03434			-	PTS sugar transporter subunit IIA	242.8	648

No.	Locus tag	Signal6P predicted[(1)]	Virulence[(2)]	Gene	Function	RPKMs[(3)]	RANK[(4)]
650	MGCS36044_04004			*pbp2A*	multimodular transpeptidase-transglycosylase	242.8	648
651	MGCS36044_04140			*hisS*	histidine--tRNA synthase HisS	242.8	648
652	MGCS36044_03490			-	GNAT family N-acetyltransferase	242.3	652
653	MGCS36044_03100			*rbsC*	D-ribose ABC transporter permease RbsC	240.5	653
654	MGCS36044_03952			*proW*	proline/glycine betaine ABC transporter ATPase	240.0	654
655	MGCS36044_03822			*rmuC*	DNA recombination protein RmuC	239.8	655
656	MGCS36044_03700		Virulence	*cppA*	CppA family putative C3-glycoprotein degrading	239.5	656
657	MGCS36044_04084			-	putative acetyltransferase	239.3	657
658	MGCS36044_04228			*sdhA*	L-serine dehydratase alpha subunit SdhA	239.3	657
659	MGCS36044_03394			*glpK*	glycerol kinase GlpK	239.0	659
660	MGCS36044_00994			*yabA*	DNA replication initiation control protein YabA	238.5	660
661	MGCS36044_00884			*dnaB*	replication initiation and membrane attachment	238.3	661
662	MGCS36044_03724	Secreted		-	putative secreted protein	238.0	662
663	MGCS36044_01758	Secreted		-	extracellular cell wall anchored bifunctional	237.5	663
664	MGCS36044_01008			*arsC_1*	arsenate reductase ArsC	237.0	664
665	MGCS36044_04230			-	HAD hydrolase-like protein	237.0	664
666	MGCS36044_04118			*mdtH*	MdtH-related MFS mutidrug resistance	236.8	666
667	MGCS36044_00782			*rgpG*	undecaprenyl/decaprenyl-phosphate	235.5	667
668	MGCS36044_03734			-	Cro/CI family transcriptional regulator	235.5	667
669	MGCS36044_01672			*capA*	CapA family protein	235.0	669
670	MGCS36044_03650			-	DUF1129 domain-containing protein	235.0	669
671	MGCS36044_03132			*fnr*	Crp/Fnr family transcriptional regulator	234.5	671
672	MGCS36044_03378			*ccmA*	CcmA family multidrug ABC transporter ATPase	234.5	671
673	MGCS36044_00486			*pabA*	aminodeoxychorismate/anthranilate synthase	233.3	673
674	MGCS36044_02006			-	Sua5/YciO/YrdC/YwlC family protein ribosome	233.3	673
675	MGCS36044_02456			-	HAD hydrolase family protein	233.3	673
676	MGCS36044_02008			-	GNAT family N-acetyltransferase	233.0	676
677	MGCS36044_01924			*folP*	dihydropteroate synthase protein FolP	232.8	677
678	MGCS36044_01376			-	sugar-phosphatase	232.5	678
679	MGCS36044_01894			-	integrase catalytic subunit	232.5	678
680	MGCS36044_04142			*rpmF*	50S ribosomal L32 protein RpmF	232.5	678
681	MGCS36044_01180			-	cell division protein FtsW-like	232.3	681
682	MGCS36044_01934			*potB*	spermidine putrescine ABC transport system permease protein PotB	232.3	681
683	MGCS36044_00546			*rseP*	RIP metalloprotease RseP	232.0	683
684	MGCS36044_01688			*rluD*	ribosomal large subunit pseudouridine synthase RluD	232.0	683
685	MGCS36044_02868			*birA*	bifunctional biotin--[acetyl-CoA-carboxylase]	231.8	685
686	MGCS36044_02088			-	site-specific integrase. Region of difference 36044_ROD.5, putative MGE	231.3	686
687	MGCS36044_03172			-	MGCS36044_0AAA family ATPase3172	230.8	687
688	MGCS36044_03074			*glcK*	glucokinase GlcK	230.0	688
689	MGCS36044_00828			*yhbY*	ribosome assembly RNA-binding protein YhbY	229.8	689
690	MGCS36044_00788			*sufS*	cysteine desulfurase SufS	229.0	690

No.	Locus tag	Signal6P predicted(1)	Virulence(2)	Gene	Function	RPKMs(3)	RANK(4)
691	MGCS36044_01372			*murN*	peptidoglycan lipid II-Ala--L-alanine ligase	229.0	690
692	MGCS36044_02470	Lipo		-	extracellular solute-binding lipoprotein	229.0	690
693	MGCS36044_04208			*dnaC*	replicative DNA helicase DnaC	229.0	690
694	MGCS36044_02704			*obgE*	GTPase ObgE	228.3	694
695	MGCS36044_00148			-	low molecular weight phosphotyrosine protein	228.0	695
696	MGCS36044_03592			-	aldo/keto reductase	227.8	696
697	MGCS36044_01666			-	hypothetical protein	227.0	697
698	MGCS36044_03646			*uvrA*	excinuclease ABC subunit (A) UvrA	226.8	698
699	MGCS36044_03400			*glyS*	glycine--tRNA ligase beta subunit GlyS	226.0	699
700	MGCS36044_03862	Lipo		*yidC_2*	YidC/Oxa1 family membrane protein insertase	225.5	700
701	MGCS36044_04082			*nrdG*	anaerobic ribonucleoside-triphosphate reductase	225.5	700
702	MGCS36044_00962			*murE_1*	UDP-N-acetylmuramoyl-L-alanyl-D-glutamate--L-	225.3	702
703	MGCS36044_02316			*ribF*	bifunctional riboflavin kinase/FAD synthetase	225.3	702
704	MGCS36044_03864			*rnpA*	ribonuclease P protein component RnpA	225.3	702
705	MGCS36044_00210			*adk*	adenylate kinase protein Adk	225.0	705
706	MGCS36044_03364			*mraY*	phospho-N-acetylmuramoyl-pentapeptide-	225.0	705
707	MGCS36044_03594			*recG*	ATP-dependent DNA helicase RecG	225.0	705
708	MGCS36044_03480			*ecsB*	ABC exoprotein transporter permease EcsB	224.5	708
709	MGCS36044_00786			*sufD*	Fe-S cluster assembly protein SufD	224.3	709
710	MGCS36044_01932			*potA*	spermidine putrescine ABC transport system	224.3	709
711	MGCS36044_03468			-	YlxR family putative RNA-binding protein	224.0	711
712	MGCS36044_01432			-	MBL fold metallo-hydrolase	223.8	712
713	MGCS36044_00366	Secreted		-	secreted pilin minor/ancillary protein	223.5	713
714	MGCS36044_00926			*scp2*	segregation/condensation complex subunit (B)	223.5	713
715	MGCS36044_03366			*pbp2X*	PG transpeptidase class B penicillin-binding	223.5	713
716	MGCS36044_01246			*smc*	chromosome segregation protein Smc	222.8	716
717	MGCS36044_03008			*tlyA*	TlyA family RNA methyltransferase	222.8	716
718	MGCS36044_02850			*uxaC*	uronate isomerase	222.5	718
719	MGCS36044_01348			*ftsW*	cell division protein FtsW	222.3	719
720	MGCS36044_01350			-	RpoE superfamily DNA-directed RNA polymerase	222.0	720
721	MGCS36044_01254			-	Cof-type HAD-IIB family hydrolase	221.0	721
722	MGCS36044_02318			*truB*	tRNA pseudouridine(55) synthase TruB	221.0	721
723	MGCS36044_01330			*thiT*	energy-coupled thiamine transporter ThiT	220.5	723
724	MGCS36044_03176			-	VOC family protein	220.5	723
725	MGCS36044_03102			*rbsA*	D-ribose transporter ATPase RbsA	220.0	725
726	MGCS36044_03732			-	PLDc N-terminal domain-containing protein	220.0	725
727	MGCS36044_02180			-	PASTA domain-containing protein	218.8	727
728	MGCS36044_02300			*ptsB2*	phosphate ABC transporter ATP-binding protein PstB2	218.5	728
729	MGCS36044_00924			*scp1*	segregation/condensation complex subunit (A)	218.3	729
730	MGCS36044_01184			*wcaA*	WcaA superfamily glycosyltransferase involved in	217.5	730
731	MGCS36044_03370			*mraW*	S-adenosyl-methyltransferase MraW	217.5	730

No.	Locus tag	Signal6P predicted[(1)]	Virulence[(2)]	Gene	Function	RPKMs[(3)]	RANK[(4)]
732	MGCS36044_02026			*gyrA*	DNA gyrase subunit A GyrA	217.3	732
733	MGCS36044_01782			*mvaS1*	hydroxymethylglutaryl-CoA reductase protein (1)	216.5	733
734	MGCS36044_00378		Virulence	*fbp*	secreted fibronectin-binding protein	216.3	734
735	MGCS36044_02410			*pyrC*	dihydroorotase PyrC	216.3	734
736	MGCS36044_00850	Lipo		-	amino acid ABC transporter substrate-binding	215.5	736
737	MGCS36044_00836			*rsfS*	ribosome silencing factor RsfS	215.3	737
738	MGCS36044_00356			-	deoxyadenosine kinase	215.0	738
739	MGCS36044_04138			*aspS*	aspartyl-tRNA synthetase	214.0	739
740	MGCS36044_03902			-	DUF6198 family protein	213.8	740
741	MGCS36044_00778			*uppP*	undecaprenyl pyrophosphate phosphatase UppP	213.3	741
742	MGCS36044_00826			*yqeH*	ribosome biogenesis GTPase YqeH	213.0	742
743	MGCS36044_00554			-	MarR family transcriptional regulator	212.8	743
744	MGCS36044_01228			-	DUF3114 domain-containing protein	211.8	744
745	MGCS36044_04240			*pgsA*	CDP-diacylglycerol--glycerol-3-phosphate	211.5	745
746	MGCS36044_00974			-	YlbG family protein	211.3	746
747	MGCS36044_00358			*dus*	tRNA-dihydrouridine synthase Dus	210.8	747
748	MGCS36044_00930			*yidD*	membrane protein insertion efficiency factor	210.8	747
749	MGCS36044_03706			*pepX*	PepX family Xaa-Pro dipeptidyl-peptidase	210.5	749
750	MGCS36044_00376			*srtC_3*	class C sortase SrtC	210.0	750
751	MGCS36044_01926			*folQ*	dihydroneopterin aldolase protein FolB	210.0	750
752	MGCS36044_02542			-	PadR family transcriptional regulator	209.8	752
753	MGCS36044_00754			-	alpha/beta hydrolase	209.0	753
754	MGCS36044_02968			*ddl*	D-alanine--D-alanine ligase Ddl	208.5	754
755	MGCS36044_01446			*whiA*	cell division involved DNA-binding protein WhiA	207.8	755
756	MGCS36044_01662			*gorA*	glutathione reductase GorA	207.8	755
757	MGCS36044_00386			-	toxic anion resistance protein, tellurite	207.3	757
758	MGCS36044_00428			-	FAD/NAD(P)-binding protein	206.8	758
759	MGCS36044_02530	Lipo		*ybbR*	YbbR family lipoprotein	206.3	759
760	MGCS36044_04092			-	DUF2079 domain-containing protein	206.0	760
761	MGCS36044_02642			*glgP*	maltodextrin phosphorylase protein GlgP	205.8	761
762	MGCS36044_00146			*ruvB*	Holliday junction branch migration DNA helicase	205.5	762
763	MGCS36044_02002			*prfA*	peptide chain release factor 1 PrfA	205.3	763
764	MGCS36044_04238			*cbiO1*	cobalt ABC transporter ATPase CbiO2	205.3	763
765	MGCS36044_03224			*bglB_2*	6-phospho-beta-glucosidase BglB	204.5	765
766	MGCS36044_00896			-	GNAT family N-acetyltransferase	203.8	766
767	MGCS36044_02722			*fetA*	iron export ABC transporter ATP-binding subunit	203.5	767
768	MGCS36044_03294		Virulence	*liaR*	three component system signal transduction response regulator protein	203.3	768
769	MGCS36044_03482			*ecsA*	ABC exoprotein transporter ATPase EcsA	203.3	768
770	MGCS36044_01914			-	polysaccharide deacetylase family protein	203.0	770
771	MGCS36044_02052	Secreted	Virulence	*isp*	secreted CHAP domain-containing immunogenic	203.0	770
772	MGCS36044_04124			-	YmcA-related cell fate/competence/biofilm	202.3	772

No.	Locus tag	Signal6P predicted[(1)]	Virulence[(2)]	Gene	Function	RPKMs[(3)]	RANK[(4)]
773	MGCS36044_03770			-	DUF536 domain-containing protein	202.0	773
774	MGCS36044_03708			-	pyridoxamine 5'-phosphate oxidase family	201.8	774
775	MGCS36044_01918			*thrB*	ThrB family homoserine kinase	201.5	775
776	MGCS36044_03108			*rbsR*	ribose transport operon repressor RbsR	201.3	776
777	MGCS36044_01242			*vicX*	zinc-dependent hydrolase protein VicX	201.0	777
778	MGCS36044_04126			*argR_3*	arginine repressor ArgR	200.5	778
779	MGCS36044_02520			*nagD*	NagD family hydrolase	199.8	779
780	MGCS36044_00488			*pabB*	para-aminobenzoate synthetase	199.3	780
781	MGCS36044_01928			*folK*	2-amino-4-hydroxy-6-hydroxymethyldihydropteridine pyrophosphokinase protein FolK	199.0	781
782	MGCS36044_03010			*ispA*	IspA family geranylgeranyl pyrophosphate	198.8	782
783	MGCS36044_00940			-	PgpB family membrane-associated phospholipid	198.5	783
784	MGCS36044_01808			-	hypothetical protein	198.5	783
785	MGCS36044_03358			*hisM*	HisM family amino acid ABC transporter permease	198.3	785
786	MGCS36044_00790			*sufE*	SUF system NifU family Fe-S cluster assembly	198.0	786
787	MGCS36044_01006			*ogt*	O6-methylguanine-DNA--protein-cysteine	197.8	787
788	MGCS36044_03624			-	IS30 family transposase	197.3	788
789	MGCS36044_00544			*cdsA*	phosphatidate cytidylyltransferase CdsA	197.0	789
790	MGCS36044_03238			-	LLM class flavin-dependent oxidoreductase	196.3	790
791	MGCS36044_00780			*mecA*	negative regulator of genetic competence,	196.0	791
792	MGCS36044_01058			*macP*	cell wall synthase accessory phosphoprotein	195.8	792
793	MGCS36044_00368			*srtC_1*	class C sortase SrtC	195.5	793
794	MGCS36044_00914			*murI*	glutamate racemase MurI	195.5	793
795	MGCS36044_01386			-	hypothetical protein	195.3	795
796	MGCS36044_03672			*rnhC*	HIII ribonuclease RnhC	195.3	795
797	MGCS36044_01788			*dyr*	dihydrofolate reductase Dyr	195.0	797
798	MGCS36044_04120			*mutL*	DNA mismatch repair endonuclease MutL	195.0	797
799	MGCS36044_01056			*nudF*	NUDIX hydrolase NudF	194.8	799
800	MGCS36044_03174			-	GNAT family N-acetyltransferase	194.8	799
801	MGCS36044_01172			-	multidrug efflux MFS transporter	194.5	801
802	MGCS36044_00900			*greA*	transcription elongation factor GreA	193.8	802
803	MGCS36044_03088			*rlmN*	23S rRNA (adenine(2503)-C(2))-methyltransferase	193.8	802
804	MGCS36044_00952			-	cytoplasmic protein	193.5	804
805	MGCS36044_01656			*aroD*	type I 3-dehydroquinate dehydratase AroD	192.8	805
806	MGCS36044_01020			*lctO*	L-lactate oxidase LctO	192.5	806
807	MGCS36044_01448			*pepD_1*	C69 family dipeptidase PepD	192.3	807
808	MGCS36044_01896			-	DUF4365 family protein	192.0	808
809	MGCS36044_03746			-	type I restriction-modification system (M)	192.0	808
810	MGCS36044_04088			-	hypothetical protein	192.0	808
811	MGCS36044_01028			*metS*	methionine--tRNA synthase MetS	191.0	811
812	MGCS36044_01784			*mvaS2*	hydroxymethylglutaryl-CoA synthase protein (2)	190.8	812

No.	Locus tag	Signal6P predicted(1)	Virulence(2)	Gene	Function	RPKMs(3)	RANK(4)
813	MGCS36044_01936			*potC*	spermidine putrescine ABC transport system permease protein PotC	190.8	812
814	MGCS36044_03114			*rsmD*	16S rRNA (guanine(966)-N(2))-methyltransferase	190.5	814
815	MGCS36044_02950			*kup*	potassium uptake protein Kup	190.3	815
816	MGCS36044_01004			-	GNAT family N-acetyltransferase	189.8	816
817	MGCS36044_00916			*rdgB*	RdgB family non-canonical purine NTP	189.5	817
818	MGCS36044_01422			*fbp2*	fructose-bisphosphatase Fbp2	189.5	817
819	MGCS36044_01910			*xerD_2*	site-specific integrase	189.5	817
820	MGCS36044_02852			*eda*	Eda family bifunctional	189.5	817
821	MGCS36044_00892			-	hypothetical protein	189.3	821
822	MGCS36044_03012			*xseB*	exodeoxyribonuclease VII small subunit XseB	189.3	821
823	MGCS36044_03204			*lacZ*	beta-galactosidase LacZ	188.8	823
824	MGCS36044_02248			*fhs_1*	formate--tetrahydrofolate ligase Fhs	188.3	824
825	MGCS36044_04000			*nusG*	transcription antitermination protein NusG	188.0	825
826	MGCS36044_00776	Secreted		-	DUF2207 domain-containing secreted protein	187.8	826
827	MGCS36044_01060			*mtnN*	5'-methylthioadenosine/adenosylhomocysteine	187.8	826
828	MGCS36044_01280			*pnpS*	phosphate TCS signal transduction histidine	187.5	828
829	MGCS36044_02682			*glnQ_2*	glutamine transport ATP-binding protein GlnQ	187.5	828
830	MGCS36044_03254			-	DUF960 domain-containing protein	187.5	828
831	MGCS36044_01000			*cutC*	copper homeostasis protein CutC	187.3	831
832	MGCS36044_02302			*ptsA*	phosphate ABC transporter permease PstA	187.3	831
833	MGCS36044_00792			*sufB*	Fe-S cluster assembly protein SufB	186.8	833
834	MGCS36044_01760			-	GTP pyrophosphokinase family protein	186.8	833
835	MGCS36044_02906			-	LURP-one-related family protein	186.8	833
836	MGCS36044_02010			*glyA*	serine hydroxymethyl transferase GlyA	186.3	836
837	MGCS36044_01998			*pptA*	phenylpyruvate tautomerase PptA	185.8	837
838	MGCS36044_02348			-	glycine RNA	185.8	837
839	MGCS36044_01930			*murB*	UDP-N-acetylmuramate dehydrogenase MurB	185.5	839
840	MGCS36044_02518			-	MGCS360TIGR01906 family membrane protein44_02518	184.8	840
841	MGCS36044_04116			*ruvA*	Holliday junction ATP-dependent DNA helicase	184.8	840
842	MGCS36044_01458			-	GntR family transcriptional regulator	184.5	842
843	MGCS36044_02612			*dnaD*	DNA replication protein DnaD	184.3	843
844	MGCS36044_00824			*yqeG*	HAD IIIA-type phosphatase YqeG	184.0	844
845	MGCS36044_01664			-	dihydrofolate synthase	184.0	844
846	MGCS36044_00408			*mipB*	fructose-6-phosphate aldolase MipB	183.8	846
847	MGCS36044_02498			*ndk*	nucleoside-diphosphate kinase Ndk. Followed by a CRISPR with 5 repeat units	183.5	847
848	MGCS36044_00006			-	DUF951 domain-containing protein	183.3	848
849	MGCS36044_02404			-	hypothetical protein	183.0	849
850	MGCS36044_00160			*thrC*	threonine synthase ThrC	182.8	850
851	MGCS36044_02054			-	hypothetical protein	182.0	851
852	MGCS36044_00584			*mrnC*	mini-ribonuclease 3 MrnC	181.8	852
853	MGCS36044_03508			-	DUF956 family protein	181.8	852

No.	Locus tag	Signal6P predicted[1]	Virulence[2]	Gene	Function	RPKMs[3]	RANK[4]
854	MGCS36044_02618			*dltE*	short-chain dehydrogenase DltE	181.5	854
855	MGCS36044_00946		Virulence	*hlyX*	hemolysin family protein HylX	181.3	855
856	MGCS36044_00964			*murJ*	peptidoglycan lipid-II intermediate flippase	181.0	856
857	MGCS36044_01938			*potD*	spermidine putrescine ABC transport system substrate-binding protein PotD	180.8	857
858	MGCS36044_04164			-	site-specific integrase	180.8	857
859	MGCS36044_02012			-	nucleoid-associated bacterial family protein	180.5	859
860	MGCS36044_02558			*frmB*	FrmB family esterase	180.5	859
861	MGCS36044_03006			*argR_1*	ArgR family transcriptional regulator	180.5	859
862	MGCS36044_04184			-	DUF368 domain-containing protein	180.0	862
863	MGCS36044_02156			-	NusG domain II-containing protein	179.5	863
864	MGCS36044_00500			*rgfB*	endonuclease/exonuclease/phosphatase family	179.3	864
865	MGCS36044_03472			*rimP*	ribosome maturation factor RimP	179.3	864
866	MGCS36044_00560			-	Crp family cyclic nucleotide-binding	179.0	866
867	MGCS36044_00990			*holB*	DNA polymerase III subunit delta' HolB	178.8	867
868	MGCS36044_03304			*rsmB*	16S rRNA (cytosine(967)-C(5))-methyltransferase	178.3	868
869	MGCS36044_02200			*topA*	type I DNA topoisomerase TopA	178.0	869
870	MGCS36044_02820			-	DinB family protein	178.0	869
871	MGCS36044_02628			-	DUF3042 family protein	177.3	871
872	MGCS36044_01530	Secreted		-	cell surface extracellular nuclease	177.0	872
873	MGCS36044_02930			*queA*	S-adenosylmethionine ribosyltransferase-isomerase QueA	177.0	872
874	MGCS36044_02594			-	Abi family CAAX protease self-immunity protein	176.8	874
875	MGCS36044_03368			*ftsL*	cell division protein FtsL	176.5	875
876	MGCS36044_03566			*ppdK*	pyruvate phosphate dikinase PpdK	176.5	875
877	MGCS36044_03492			*tsaE*	tRNA (adenosine(37)-N6)-threonylcarbamoyltransferase TsaE	176.3	877
878	MGCS36044_00928			*rluB*	ribosomal large subunit pseudouridine synthase	175.5	878
879	MGCS36044_01654			*rlmK*	23S rRNA methyltransferase RmlK	175.3	879
880	MGCS36044_02620			*rnz*	ribonuclease Rnz	174.5	880
881	MGCS36044_02412			*ung*	uracil-DNA glycosylase Ung	174.3	881
882	MGCS36044_03104			*rbsD*	D-ribose pyranase RbsD	174.3	881
883	MGCS36044_03152			-	CRISPR with 9 repeat units	174.3	881
884	MGCS36044_03642			*comEB*	competence protein ComEB	174.3	881
885	MGCS36044_01076			-	DUF3397 domain-containing protein	173.0	885
886	MGCS36044_04136			-	YitT family protein putative ABC transporter ATPase	172.8	886
887	MGCS36044_01882			*copB*	copper-exporting ATPase cCopB	172.3	887
888	MGCS36044_02454			*uvrC*	excinuclease ABC subunit UvrC	171.0	888
889	MGCS36044_02142			-	helix-turn-helix transcriptional regulator	170.8	889
890	MGCS36044_01122			-	IS1182 family transposase	170.5	890
891	MGCS36044_02304			*ptsC*	phosphate ABC transporter permease PstC	170.5	890
892	MGCS36044_02610			*nth*	endonuclease III Nth	170.3	892
893	MGCS36044_01772			-	isopentenyl-diphosphate delta-isomerase	170.0	893
894	MGCS36044_02854			*fadR*	FadR family DNA-binding transcriptional	170.0	893

No.	Locus tag	Signal6P predicted[(1)]	Virulence[(2)]	Gene	Function	RPKMs[(3)]	RANK[(4)]
895	MGCS36044_01326			*yccF*	YccF domain-containing protein	169.8	895
896	MGCS36044_00372	Secreted		-	truncated secreted pilin minor/ancillary protein	169.5	896
897	MGCS36044_02952			*deaD*	DEAD/DEAH box helicase	169.5	896
898	MGCS36044_04006			-	translation initiation inhibitor	169.3	898
899	MGCS36044_02472			-	YesN family TCS DNA-binding response regulator	169.0	899
900	MGCS36044_03296		Virulence	*liaS*	three component system signal transduction sensor histidine kinase protein	169.0	899
901	MGCS36044_01576			*pheT*	phenylalanyl-tRNA synthetase beta subunit PheT	168.0	901
902	MGCS36044_03832			*rsmA*	16S rRNA (adenine(1518)-N(6)/adenine(1519)-N(6))- dimethyltransferase RsmA	168.0	901
903	MGCS36044_00342			-	hypothetical protein	167.8	903
904	MGCS36044_02624			*hflX*	GTP-binding protein HflX	167.3	904
905	MGCS36044_01252			-	Cof-type HAD-IIB family hydrolase	166.3	905
906	MGCS36044_02928			*nagB*	glucosamine-6-phosphate deaminase NagB	166.3	905
907	MGCS36044_04232			-	transglycosylase SLT domain-containing protein	166.0	907
908	MGCS36044_01676			-	L21_leader RNA	165.8	908
909	MGCS36044_03090			*yutD*	YutD family protein of unknown function	165.8	908
910	MGCS36044_03564			*gatC_2*	aspartyl-tRNA(Asn) or glutamyl-tRNA(Gln) amidotransferase C subunit GatC	165.8	908
911	MGCS36044_00992			*yaaT*	cell fate regulator YaaT	165.5	911
912	MGCS36044_01278			*pnpR*	phosphate TCS signal transduction response	165.5	911
913	MGCS36044_03954			*proV*	proline/glycine betaine ABC transporter permease	165.3	913
914	MGCS36044_02984			*pyrD*	dihydroorotate dehydrogenase PyrD	165.0	914
915	MGCS36044_01016			*sdpI*	SdpI family immunity protein	164.8	915
916	MGCS36044_03064			*murD*	UDP-N-acetylmuramoyl-L-alanine--D-glutamate	164.3	916
917	MGCS36044_02306	Lipo		*ptsS*	phosphate ABC transporter substrate-binding lipoprotein PstS	164.0	917
918	MGCS36044_02446			*mnmE*	MnmE family tRNA	164.0	917
919	MGCS36044_00388			-	MGCS36044_00388	163.8	919
920	MGCS36044_03744			-	type I restriction-modification system	163.8	919
921	MGCS36044_02492			-	RnaY family phosphodiesterase	163.5	921
922	MGCS36044_03014			*xseA*	exodeoxyribonuclease VII large subunit XseA	163.3	922
923	MGCS36044_03206		Virulence	*trxR*	TCS DNA-binding response regulator protein TrxR	162.8	923
924	MGCS36044_02878			*udk*	uridine kinase Udk	162.5	924
925	MGCS36044_03630			*scrA*	sucrose-specific PTS fused IIB/IIC/IIA component	162.5	924
926	MGCS36044_03796	Lipo		*oppA_2*	oligopeptide ABC transporter substrate-binding lipoprotein OppA	162.5	924
927	MGCS36044_02824			-	23S rRNA (uracil(1939)-C(5))-methyltransferase	162.0	927
928	MGCS36044_03106			*rbsK*	ribokinase RbsK	162.0	927
929	MGCS36044_01324			-	PhoE superfamily phosphatase	161.5	929
930	MGCS36044_02630			-	C4-dicarboxylate ABC transporter	161.3	930
931	MGCS36044_00032			*plaP*	amino acid permease PlaP	161.0	931
932	MGCS36044_00370			*srtC_2*	class C sortase SrtC	161.0	931
933	MGCS36044_01620			*rgpF*	alpha-L-Rha alpha-1,3-L-rhamnosyltransferase	161.0	931

No.	Locus tag	Signal6P predicted(1)	Virulence(2)	Gene	Function	RPKMs(3)	RANK(4)
934	MGCS36044_01842			-	calcium-binding protein	161.0	931
935	MGCS36044_02402			*parC*	DNA topoisomerase IV subunit A ParC	160.8	935
936	MGCS36044_01844			-	hypothetical protein	160.5	936
937	MGCS36044_01444			*cofD*	CofD/YvcK superfamily 2-phospho-L-lactate	160.3	937
938	MGCS36044_02864			*metK*	methionine adenosyltransferase MetK	160.0	938
939	MGCS36044_00024	Secreted		-	class A beta-lactamase-related serine hydrolase. SignalP6 predicted standard signal peptide secreted	159.5	939
940	MGCS36044_01496			-	HAD-IA family hydrolase	159.5	939
941	MGCS36044_01074			*ftsK*	cell division protein FtsK	159.3	941
942	MGCS36044_03178			-	helix-hairpin-helix domain-containing protein	159.3	941
943	MGCS36044_03674			*lepB_2*	signal peptidase I LepB	159.3	941
944	MGCS36044_00582			*cysS*	cysteine--tRNA synthetase CysS	159.0	944
945	MGCS36044_01174			*secG*	preprotein translocase subunit SecG	159.0	944
946	MGCS36044_02004			*prmC*	peptide chain release factor N(5)-glutamine	158.8	946
947	MGCS36044_00866			*ktrA*	potassium uptake transporter gating subunit	158.3	947
948	MGCS36044_01442			*rapZ*	RNase adapter RapZ	158.3	947
949	MGCS36044_01734			*fruA*	fructose-specific PTS transporter EIIC component	157.8	949
950	MGCS36044_00580			-	hypothetical protein	157.3	950
951	MGCS36044_02644			*malQ*	4-alpha-glucanotransferase (amylomaltase)	157.3	950
952	MGCS36044_00360			*hslO*	Hsp33 family molecular chaperone HslO	157.0	952
953	MGCS36044_01346			*ppc*	phosphoenolpyruvate carboxylase Ppc	157.0	952
954	MGCS36044_02382			-	TVP38/TMEM64 family protein	157.0	952
955	MGCS36044_00448			-	radical SAM protein	156.5	955
956	MGCS36044_01768			*mvaD*	diphosphomevalonate decarboxylase MvaD	156.3	956
957	MGCS36044_00726			*truA*	tRNA pseudouridine(38-40) synthase TruA	156.0	957
958	MGCS36044_01388			*regR*	transcriptional regulator RegR	156.0	957
959	MGCS36044_01742			-	DUF1149 domain-containing protein	156.0	957
960	MGCS36044_02138			-	hypothetical protein	156.0	957
961	MGCS36044_01504			-	DUF1694 domain-containing protein	155.8	961
962	MGCS36044_01468			-	PTS sugar transport IIA subunit	155.5	962
963	MGCS36044_02522			*fatA*	FatA family acyl-[acyl-carrier-protein]	155.3	963
964	MGCS36044_01756			*fms*	peptide deformylase	155.0	964
965	MGCS36044_02366			*lepB_1*	signal peptidase I	155.0	964
966	MGCS36044_02556			-	hypothetical protein	155.0	964
967	MGCS36044_01622			-	glycosyltransferase family 2 protein	154.8	967
968	MGCS36044_02256			*gcvH*	glycine cleavage system protein H GcvH	154.8	967
969	MGCS36044_03138			-	MmcQ/YjbR family DNA-binding protein	154.5	969
970	MGCS36044_01088			*cvfB*	S1 RNA-binding domain-containing protein CvfB	154.3	970
971	MGCS36044_02932			-	DUF3114 domain-containing protein	154.0	971
972	MGCS36044_03924			-	hypothetical protein	154.0	971
973	MGCS36044_02622			-	hypothetical protein	153.8	973
974	MGCS36044_01770			*mvaK2*	mevalonate kinase MvaK2	153.5	974

No.	Locus tag	Signal6P predicted[(1)]	Virulence[(2)]	Gene	Function	RPKMs[(3)]	RANK[(4)]
975	MGCS36044_02186			*gid*	tRNA (uracil-5-)-methyltransferase/glucose	153.3	975
976	MGCS36044_03648			-	CorA family divalent cation transport protein	152.5	976
977	MGCS36044_02970			*recR*	recombination mediator RecR	152.0	977
978	MGCS36044_03372			*proA*	glutamate-5-semialdehyde dehydrogenase ProA	151.5	978
979	MGCS36044_00424			-	putative transcriptional regulator	151.3	979
980	MGCS36044_01748			-	Uup family ATPase components of ABC transporters	151.3	979
981	MGCS36044_01916			*hom*	homoserine dehydrogenase Hom	151.0	981
982	MGCS36044_02504			-	CRISPR-DR22 RNA	151.0	981
983	MGCS36044_03018			*folD*	bifunctional methylenetetrahydrofolate	151.0	981
984	MGCS36044_04166			*pezT*	zeta toxin family protein PezT	151.0	981
985	MGCS36044_00292			*cydB*	cytochrome d ubiquinol oxidase subunit (II)	150.8	985
986	MGCS36044_00406			*pflD*	pyruvate formate-lyase protein PflD	150.8	985
987	MGCS36044_01042			-	DUF2829 domain-containing protein	150.5	987
988	MGCS36044_01616			*rgpD*	ABC transporter polysaccharide/polyol phosphate ATPase component RgpD	150.5	987
989	MGCS36044_02460			*yjjG*	YjjG family noncanonical pyrimidine	150.5	987
990	MGCS36044_01328			-	aminoacyl-tRNA deacylase	150.0	990
991	MGCS36044_02532			-	DisA N domain-containing diadenylate cyclase	150.0	990
992	MGCS36044_02842			-	BglX family glycosyl hydrolase	149.8	992
993	MGCS36044_00352			-	DUF4479 and tRNA-binding domain-containing	149.0	993
994	MGCS36044_03784			-	DUF1447 family protein	149.0	993
995	MGCS36044_02388			-	IS1182 family transposase	148.0	995
996	MGCS36044_02822			-	CD1845 family protein	148.0	995
997	MGCS36044_01176			*rnr*	exoribonuclease (R) Rnr	147.8	997
998	MGCS36044_01618			*rgpE*	glycosyltransferase family GT2 protein RgpE	147.8	997
999	MGCS36044_03086			-	VanZ like family glycopeptide antibiotic	147.5	999
1000	MGCS36044_03192			*aroB*	3-dehydroquinate synthase protein AroB	147.5	999
1001	MGCS36044_03328			*luxS*	S-ribosylhomocysteine lyase	147.5	999
1002	MGCS36044_04212			-	DHH family phosphoesterase	147.5	999
1003	MGCS36044_01312			-	PrtC family collagenase-like protease	147.3	1003
1004	MGCS36044_01690			-	membrane-associated alkaline phosphatase	146.5	1004
1005	MGCS36044_00496			-	LacI family DNA-binding transcriptional	145.5	1005
1006	MGCS36044_01294			*tex*	RNA-binding transcriptional accessory protein	145.5	1005
1007	MGCS36044_01686			*lspA*	lipoprotein signal peptidase II LspA	145.3	1007
1008	MGCS36044_01588			*rexB*	ATP-dependent nuclease B subunit RexB	144.8	1008
1009	MGCS36044_02014			-	lysozyme family protein	144.8	1008
1010	MGCS36044_03016			-	PfkB superfamily kinase	144.8	1008
1011	MGCS36044_01670			*thiI*	thiamine biosynthesis/tRNA modification protein	144.3	1011
1012	MGCS36044_02494			*msrB*	peptide-methionine (R)-S-oxide reductase MsrB	143.8	1012
1013	MGCS36044_00890			*snf*	SWF/SNF family helicase	143.5	1013
1014	MGCS36044_01634			-	RfbX superfamily lipopolysaccharide biosynthesis	142.8	1014
1015	MGCS36044_01466			-	PTS sugar transport IID subunit	142.3	1015

No.	Locus tag	Signal6P predicted[(1)]	Virulence[(2)]	Gene	Function	RPKMs[(3)]	RANK[(4)]
1016	MGCS36044_01996			-	thiamine biosynthesis protein ApbE-like	142.3	1015
1017	MGCS36044_02962			*prfC*	peptide chain release factor 3 PrfC	142.3	1015
1018	MGCS36044_03834			*rrmV*	5S rRNA maturation endonuclease RnmV	142.3	1015
1019	MGCS36044_02474			-	YesM family TCS sensor histidine kinase	141.5	1019
1020	MGCS36044_03886			*radA*	DNA repair protein RadA	141.3	1020
1021	MGCS36044_00882			*nrdR*	transcriptional regulator NrdR	141.0	1021
1022	MGCS36044_01178			*smpB*	SsrA(tmRNA)-binding protein SmpB	140.8	1022
1023	MGCS36044_02354			-	IS1182 family transposase	140.5	1023
1024	MGCS36044_02406			*parE*	DNA topoisomerase IV subunit B ParE	140.5	1023
1025	MGCS36044_00504			-	M42 family metallopeptidase	140.3	1025
1026	MGCS36044_04206			-	Veg family protein	140.0	1026
1027	MGCS36044_00426			-	hypothetical protein	139.8	1027
1028	MGCS36044_02616			*recJ*	single-stranded-DNA-specific exonuclease RecJ	139.8	1027
1029	MGCS36044_03226			-	beta-N-acetylglucosaminidase	139.8	1027
1030	MGCS36044_00542			*uppS*	UDP pyrophosphate synthase UppS	139.5	1030
1031	MGCS36044_01900			-	MobC family plasmid mobilization relaxosome	139.5	1030
1032	MGCS36044_04252			*glcU*	glucose uptake permease GlcU	139.5	1030
1033	MGCS36044_02374			*dnaE*	DNA polymerase III subunit alpha DnaE	139.3	1033
1034	MGCS36044_00362		Virulence	*rofA*	pilus transcriptional regulator RofA	138.8	1034
1035	MGCS36044_01850			-	DUF5966 family protein	138.8	1034
1036	MGCS36044_02836			*spxR*	SpxR family CBS-HotDog domain-containing	138.3	1036
1037	MGCS36044_01332			-	Unknown	137.8	1037
1038	MGCS36044_04080			*yaaA*	peroxide stress protein YaaA	137.8	1037
1039	MGCS36044_03216			*ugpA_1*	sugar ABC transporter permease UgpA	137.5	1039
1040	MGCS36044_02640			-	NADPH-dependent FMN reductase	137.0	1040
1041	MGCS36044_02826			-	LCP family anionic cell polymer synthesis	137.0	1040
1042	MGCS36044_00010			*engD*	redox-regulated ATPase EngD	136.8	1042
1043	MGCS36044_02228			*pdxK*	pyridoxamine kinase PdxK	136.8	1042
1044	MGCS36044_02918			-	NAD(P)H-dependent oxidoreductase	136.5	1044
1045	MGCS36044_02286			*coaA*	type I pantothenate kinase	136.3	1045
1046	MGCS36044_00350			*trxA_1*	thioredoxin family protein TrxA-like	136.0	1046
1047	MGCS36044_02604			*zupT*	ZIP family metal transporter ZupT	136.0	1046
1048	MGCS36044_00868			*ktrB*	potassium uptake transporter channel subunit	134.8	1048
1049	MGCS36044_01464			-	PTS sugar transport IIC subunit	134.8	1048
1050	MGCS36044_01974			*nadK*	NAD kinase NadK	134.5	1050
1051	MGCS36044_01054			*glmU*	bifunctional UDP-N-acetylglucosamine	134.0	1051
1052	MGCS36044_01746			-	tRNA CCA-pyrophosphorylase	134.0	1051
1053	MGCS36044_02966			*murF*	UDP-N-acetylmuramoyl-tripeptide--D-alanyl-D-	133.3	1053
1054	MGCS36044_03436			*glpR*	GlpR family DNA-binding transcriptional	133.3	1053
1055	MGCS36044_04134			-	YitT family protein putative ABC transporter ATPase	133.3	1053
1056	MGCS36044_01420			-	HAD family phosphatase	133.0	1056
1057	MGCS36044_03570			*ccpN*	CcpN family CBS pair domain transcriptional	133.0	1056

No.	Locus tag	Signal6P predicted[(1)]	Virulence[(2)]	Gene	Function	RPKMs[(3)]	RANK[(4)]
1058	MGCS36044_02890			*nrdH*	glutaredoxin-like protein NrdH	132.8	1058
1059	MGCS36044_03414			*degV_2*	DegV family fatty acid-binding protein	132.5	1059
1060	MGCS36044_00502			-	Udk family kinase	132.3	1060
1061	MGCS36044_00734			-	mechanosensitive ion channel family protein	132.3	1060
1062	MGCS36044_02050		Virulence	*ihk*	TCS signal transduction histidine kinase sensor	132.3	1060
1063	MGCS36044_02284			*rsmC*	class I SAM-dependent methyltransferase	132.3	1060
1064	MGCS36044_03740			-	major facilitator superfamily protein	132.3	1060
1065	MGCS36044_01628			-	glycosyltransferase family 1 protein	131.8	1065
1066	MGCS36044_03158			*cas4*	CRISPR-associated protein Cas4	131.0	1066
1067	MGCS36044_00020			*divIC*	septum formation initiator family protein	130.8	1067
1068	MGCS36044_03402			*glyQ*	glycine--tRNA ligase alpha subunit GlyQ	130.8	1067
1069	MGCS36044_02132			*mngR*	MngR family DNA-binding transcriptional	130.5	1069
1070	MGCS36044_02972			*pbp2B*	penicillin-binding protein PBP2B/FtsI	130.5	1069
1071	MGCS36044_03888			*dut*	deoxyuridine 5'-triphosphate nucleotidohydrolase	130.5	1069
1072	MGCS36044_04122			*mutS*	DNA mismatch repair protein MutS	129.8	1072
1073	MGCS36044_01658			*aroC*	chorismate synthase AroC	129.3	1073
1074	MGCS36044_02502			-	CRISPR-DR22 RNA	129.3	1073
1075	MGCS36044_03778			*tsaD*	tRNA (adenosine(37)-N6)-threonylcarbamoyltransferase complex transferase subunit TsaD	129.3	1073
1076	MGCS36044_00490			-	DUF3013 family protein	129.0	1076
1077	MGCS36044_01472			-	IS30 family transposase	129.0	1076
1078	MGCS36044_01202			*thrS*	threonyl-tRNA synthetase ThrS	128.8	1078
1079	MGCS36044_02506			-	CRISPR-DR22 RNA	128.8	1078
1080	MGCS36044_00784			*sufC*	Fe-S cluster assembly ATPase SufC	128.0	1080
1081	MGCS36044_01572			*endA*	DNA-entry competence-associated nuclease EndA	128.0	1080
1082	MGCS36044_03248			*trmA*	TrmA RNA methyltransferase	128.0	1080
1083	MGCS36044_02524			*hemN*	HemN family coproporphyrinogen III oxidase or	127.8	1083
1084	MGCS36044_04168			-	helix-turn-helix domain-containing	127.8	1083
1085	MGCS36044_01976			*rluA_1*	RluA family pseudouridine synthase	127.5	1085
1086	MGCS36044_01002			*serC*	3-phosphoserine/phosphohydroxythreonine	127.3	1086
1087	MGCS36044_01574			*pheS*	phenylalanyl-tRNA synthetase alpha subunit PheS	127.0	1087
1088	MGCS36044_02400			*bcaT*	branched-chain amino acid aminotransferase	127.0	1087
1089	MGCS36044_02162	Lipo		-	FMN-binding protein, major membrane immunogen,	126.8	1089
1090	MGCS36044_03164			*csd1*	Csd1 family CRISPR-associated protein	126.8	1089
1091	MGCS36044_04058			-	AAA family ATPase domain-containing DNA-binding	126.8	1089
1092	MGCS36044_00772			-	ABC amino acid transporter ATP-binding protein	126.5	1092
1093	MGCS36044_00706			-	Cof-type HAD-IIB family hydrolase	126.3	1093
1094	MGCS36044_01954			-	AI-2E family transporter	126.3	1093
1095	MGCS36044_02606			-	Nif3-like dinuclear metal center hexameric	126.3	1093
1096	MGCS36044_00022			-	hypothetical protein	126.0	1096
1097	MGCS36044_00774	Secreted		-	ABC amino acid transporter substrate-binding	126.0	1096

No.	Locus tag	Signal6P predicted[(1)]	Virulence[(2)]	Gene	Function	RPKMs[(3)]	RANK[(4)]
1098	MGCS36044_02896			*clcB*	voltage-gated ClC-type chloride channel ClcB	125.8	1098
1099	MGCS36044_02250			*lplA_1*	lipoate--protein ligase	125.0	1099
1100	MGCS36044_01322			-	HAD family hydrolase	124.3	1100
1101	MGCS36044_03644			*pepP*	PepP family Xaa-Pro peptidase	124.3	1100
1102	MGCS36044_02210			-	DUF1836 domain-containing protein	123.8	1102
1103	MGCS36044_02260			*lipL*	lipoate--protein ligase	123.8	1102
1104	MGCS36044_03680			*dinB*	DNA polymerase IV DinB	123.8	1102
1105	MGCS36044_01482			-	IS1548 family transposase	123.5	1105
1106	MGCS36044_01632			*galE*	UDP-glucose 4-epimerase GalE	123.3	1106
1107	MGCS36044_03244			*thlA*	thiolase ThlA	123.3	1106
1108	MGCS36044_03416			-	TetR/AcrR family transcriptional regulator	122.8	1108
1109	MGCS36044_02432			-	LysR family transcriptional regulator	122.5	1109
1110	MGCS36044_01052			-	UhpC family MFS transporter	122.0	1110
1111	MGCS36044_03628			*scrK*	fructokinase protein ScrK	121.5	1111
1112	MGCS36044_03842			*nanK*	N-acetylmannosamine kinase NanK	121.3	1112
1113	MGCS36044_03140		Virulence	*yesM*	TCS sensor kinase YesM	121.0	1113
1114	MGCS36044_04132			-	YitT family protein putative ABC transporter	121.0	1113
1115	MGCS36044_00140			*purB*	adenylosuccinate lyase PurB	120.8	1115
1116	MGCS36044_02048		Virulence	*irr*	TCS signal transduction DNA-binding response	120.8	1115
1117	MGCS36044_00290			*cydA*	cytochrome ubiquinol oxidase subunit (I) CydA	120.5	1117
1118	MGCS36044_00988			*tmk*	thymidylate kinase Tmk	120.5	1117
1119	MGCS36044_01534			*queT*	Queuosine precursor transporter QueT	120.0	1119
1120	MGCS36044_02830			*aroA*	3-phosphoshikimate 1-carboxyvinyltransferase	119.8	1120
1121	MGCS36044_04200			-	IS1182 family transposase	119.8	1120
1122	MGCS36044_03836			*tatD*	Tat protein secretion system quality control	119.5	1122
1123	MGCS36044_00440			-	hypothetical protein	118.8	1123
1124	MGCS36044_01378			-	HD domain-containing phosphohydrolase	118.5	1124
1125	MGCS36044_01624			-	DUF2304 domain-containing protein	118.5	1124
1126	MGCS36044_03214			*ugpE_1*	glycerol-3-phosphate ABC transporter permease	118.5	1124
1127	MGCS36044_03884			-	carbonic anhydrase	118.5	1124
1128	MGCS36044_00524			-	ISAs1 family transposase	118.3	1128
1129	MGCS36044_02500			-	CRISPR-DR22 RNA	118.0	1129
1130	MGCS36044_03180			-	DUF1912 family protein	118.0	1129
1131	MGCS36044_01014			*exoA*	exodeoxyribonuclease III protein ExoA	117.0	1131
1132	MGCS36044_01626			-	DUF2142 domain-containing protein	116.5	1132
1133	MGCS36044_03208		Virulence	*trxS*	TCS sensor histidine kinase TrxS	116.5	1132
1134	MGCS36044_02680	Lipo		*glnP_2*	glutamine-binding protein/glutamine transport	116.3	1134
1135	MGCS36044_03854	Lipo		*ugpB_2*	carbohydrate ABC transporter substrate-binding	115.8	1135
1136	MGCS36044_01898			-	SAG1252 family conjugative relaxosome accessory	115.3	1136
1137	MGCS36044_01488			*clcA*	voltage-gated chloride channel family protein	115.0	1137
1138	MGCS36044_04028			*cspA*	cold-shock protein CspA	115.0	1137

No.	Locus tag	Signal6P predicted(1)	Virulence(2)	Gene	Function	RPKMs(3)	RANK(4)
1139	MGCS36044_03210			*trxT*	Trx TCS operon protein TrxT	114.8	1139
1140	MGCS36044_00760			*rpmB*	50S ribosomal L28 protein RpmB	114.5	1140
1141	MGCS36044_01230			*glnP_1*	glutamine ABC transporter permease GlnP	114.5	1140
1142	MGCS36044_01738	Lipo		-	putative peptidoglycan hydrolase lipoprotein	114.5	1140
1143	MGCS36044_01462			-	PTS sugar transport IIB subunit	114.3	1143
1144	MGCS36044_02608			*trmK*	tRNA (adenine(22)-N(1))-methyltransferase TrmK	114.3	1143
1145	MGCS36044_00394			*pflC*	pyruvate formate-lyase activating enzyme PflC	114.0	1145
1146	MGCS36044_01796			-	hypothetical protein	114.0	1145
1147	MGCS36044_00744			*pyrG*	CTP synthase Pyg	113.8	1147
1148	MGCS36044_01566			-	DUF1146 domain-containing protein	113.8	1147
1149	MGCS36044_01684			*lysR*	LysR family transcriptional regulator	113.8	1147
1150	MGCS36044_04110			*cinA*	competence/damage-inducible protein CinA	113.8	1147
1151	MGCS36044_00840			-	nucleotidyltransferase	113.0	1151
1152	MGCS36044_01614			*rgpC*	ABC transporter polysaccharide/polyol phosphate	113.0	1151
1153	MGCS36044_01722			*trmD*	tRNA (guanosine(37)-N1)-methyltransferase TrmD	113.0	1151
1154	MGCS36044_00318			-	DUF1033 domain-containing protein	112.8	1154
1155	MGCS36044_01848			-	DUF5962 family protein	112.8	1154
1156	MGCS36044_02828			*aroK*	shikimate kinase AroK	112.5	1156
1157	MGCS36044_03398			*ynzC*	DUF896 family protein	112.3	1157
1158	MGCS36044_02226			-	ECF transporter S component	112.0	1158
1159	MGCS36044_02874			*prsW*	PrsW family glutamic-type intramembrane	112.0	1158
1160	MGCS36044_03278			-	IS30 family transposase	111.8	1160
1161	MGCS36044_03374			*proB*	glutamate 5-kinase ProB	111.8	1160
1162	MGCS36044_03092			-	IS1548 family transposase	111.0	1162
1163	MGCS36044_03160			*cas7*	type I-C CRISPR-associated protein Cas7/Csd2	111.0	1162
1164	MGCS36044_00340			-	CAAX amino terminal protease family membrane	110.8	1164
1165	MGCS36044_02238			*cls*	cardiolipin synthase	110.8	1164
1166	MGCS36044_01206			-	ABC transporter permease component	110.5	1166
1167	MGCS36044_02368			-	IS1548 family transposase	110.5	1166
1168	MGCS36044_04214			-	IS1548 family transposase	110.5	1166
1169	MGCS36044_03166			*cas5*	type I-C CRISPR-associated protein Cas5	110.3	1169
1170	MGCS36044_00288			*nox*	NAD(P)/FAD-dependent oxidoreductase Nox	109.8	1170
1171	MGCS36044_00534	Secreted	Virulence	*pulA_1*	cell surface pullulanase PulA	109.5	1171
1172	MGCS36044_02252			-	NAD-dependent deacetylase	109.5	1171
1173	MGCS36044_02964			-	TIGR02206 family membrane protein	109.5	1171
1174	MGCS36044_00380			-	PAS domain-containing protein	109.3	1174
1175	MGCS36044_00808			-	IS30 family transposase	109.3	1174
1176	MGCS36044_02862			*murA_2*	UDP-N-acetylglucosamine 1-carboxyvinyltransferase MurA	109.3	1174
1177	MGCS36044_00658			-	type II toxin-antitoxin system Phd/YefM family	108.5	1177
1178	MGCS36044_00512			*nrdI_1*	class Ib ribonucleoside-diphosphate reductase	108.3	1178
1179	MGCS36044_02486			-	DUF2974 domain-containing protein	107.8	1179

No.	Locus tag	Signal6P predicted(1)	Virulence(2)	Gene	Function	RPKMs(3)	RANK(4)
1180	MGCS36044_02636			-	rhodanese-related sulfurtransferase	107.8	1179
1181	MGCS36044_02938			*holA*	DNA polymerase III delta subunit HolA	107.5	1181
1182	MGCS36044_04250			*recF*	DNA replication/repair protein RecF	107.5	1181
1183	MGCS36044_03942			-	DUF975 family protein	107.3	1183
1184	MGCS36044_04226			*sdhB*	L-serinedehydratase beta subunit SdhB	107.0	1184
1185	MGCS36044_04272			*rlmH*	23S rRNA (pseudouridine(1915)-N(3))-methyltransferase RlmH	107.0	1184
1186	MGCS36044_02386			-	DUF3862 domain-containing lipoprotein	106.8	1186
1187	MGCS36044_01840	Secreted		-	cell surface extracellular antigen I/II family	106.5	1187
1188	MGCS36044_01130			-	IS1548 family transposase	106.0	1188
1189	MGCS36044_01208			-	ABC transporter permease component	106.0	1188
1190	MGCS36044_01596			-	IS1548 family transposase	106.0	1188
1191	MGCS36044_01732			*fruK*	1-phosphofructokinase FruK	106.0	1188
1192	MGCS36044_02462			-	DUF1524 domain-containing protein	105.3	1192
1193	MGCS36044_03772			*yhiN*	YhiN family predicted flavoprotein	105.3	1192
1194	MGCS36044_02320			-	DUF2130 domain-containing protein	105.0	1194
1195	MGCS36044_03846			-	DUF624 domain-containing protein	105.0	1194
1196	MGCS36044_00762			-	IS1548 family transposase	104.8	1196
1197	MGCS36044_01902			-	SAG1250 family conjugative relaxase	104.5	1197
1198	MGCS36044_03042			-	hypothetical protein	104.5	1197
1199	MGCS36044_03190			*aroGA*	bifunctional 3-deoxy-7-phosphoheptulonate	104.5	1197
1200	MGCS36044_02712			*rsuA_1*	ribosomal small subunit pseudouridine synthase	104.3	1200
1201	MGCS36044_03298		Virulence	*liaF*	three component system signal transduction membrane component protein	104.3	1200
1202	MGCS36044_01224			*paaJ*	acetyl-CoA acetyl transferase PaaJ	104.0	1202
1203	MGCS36044_01630			-	LTA synthase family protein	104.0	1202
1204	MGCS36044_04056			*hutG*	formiminoglutamase HutG	104.0	1202
1205	MGCS36044_01752			-	MdlB family multidrug ABC transporter ATPase and permease component	103.8	1205
1206	MGCS36044_01226			*ppsB*	long-chain fatty acid--CoA ligase PpsB	103.5	1206
1207	MGCS36044_02258			-	NADH-dependent flavin oxidoreductase	103.5	1206
1208	MGCS36044_01610			*rgpA*	glycosyltransferase family 1 protein RgpA	103.3	1208
1209	MGCS36044_02464			-	maltose/galactose O-acetyltransferase	103.3	1208
1210	MGCS36044_03354			-	DUF4059 family protein	103.0	1210
1211	MGCS36044_01674			-	IS30 family transposase	102.8	1211
1212	MGCS36044_02488			*spiA*	sakacin P immunity protein SpiA	102.8	1211
1213	MGCS36044_01250			-	IS1548 family transposase	102.3	1213
1214	MGCS36044_01786			*thyA*	thymidylate synthase ThyA	101.8	1214
1215	MGCS36044_04024			*ctsR*	CtsR family transcriptional regulator	101.8	1214
1216	MGCS36044_00026			*tilS*	tRNA lysidine(34) synthetase TilS	101.5	1216
1217	MGCS36044_01612			*rgpB*	glycosyltransferase family GT2 protein RgpB	101.3	1217
1218	MGCS36044_02206			*ylqF*	ribosome biogenesis GTPase YlqF	101.0	1218
1219	MGCS36044_03116			*asnA*	asparagine synthetase AsnA	101.0	1218
1220	MGCS36044_01890			*arsR*	cadmium efflux system metalloregulator ArsR/SmtB	100.8	1220

No.	Locus tag	Signal6P predicted(1)	Virulence(2)	Gene	Function	RPKMs(3)	RANK(4)
1221	MGCS36044_02254			-	protein-ADP-ribose hydrolase	100.8	1220
1222	MGCS36044_03240			*caiC*	CaiC family Acyl-CoA synthetase	100.5	1222
1223	MGCS36044_02346			*pcrA*	DNA helicase PcrA	100.0	1223
1224	MGCS36044_02408			*plsY*	glycerol-3-phosphate 1-O-acyltransferase PlsY	99.8	1224
1225	MGCS36044_03076			-	DUF910 domain-containing protein	99.8	1224
1226	MGCS36044_02974			*focA*	formate transporter FocA	99.0	1226
1227	MGCS36044_00452			-	MdlB family ABC transporter ATP-binding/permease	98.0	1227
1228	MGCS36044_01434			*dinG*	bifunctional DnaQ family exonuclease --	98.0	1227
1229	MGCS36044_01454			*agaS*	AgaS superfamily sugar isomerase SIS	98.0	1227
1230	MGCS36044_02398			-	DUF2969 domain-containing protein	98.0	1227
1231	MGCS36044_03306			*fmt*	methionyl-tRNA formyl transferase Fmt	98.0	1227
1232	MGCS36044_04012			-	hypothetical protein	98.0	1227
1233	MGCS36044_01584			-	ABC transporter ATP-binding component LolD-like	97.5	1233
1234	MGCS36044_00334			-	class I SAM-dependent methyltransferase	97.3	1234
1235	MGCS36044_01878			*copA_1*	copper-exporting ATPase CopA	97.3	1234
1236	MGCS36044_03676			*recD*	ATP-dependent DNA helicase RecD	97.3	1234
1237	MGCS36044_02000			*tdk*	thymidine kinase Tdk	96.8	1237
1238	MGCS36044_02876			-	DUF1294 domain-containing protein	96.8	1237
1239	MGCS36044_04054			*hutH*	histidine ammonia-lyase HutH	96.8	1237
1240	MGCS36044_03288			*cysK*	cysteine synthase A CysK	96.5	1240
1241	MGCS36044_03568			-	kinase/pyrophosphorylase	96.3	1241
1242	MGCS36044_01460			*bga*	beta-galactosidase Bga	95.8	1242
1243	MGCS36044_01092			*yozE*	YozE family protein	95.3	1243
1244	MGCS36044_02910			*yrrM*	YrrM family O-methyltransferase	95.0	1244
1245	MGCS36044_01090			*msrA*	peptide-methionine (S)-S-oxide reductase MsrA	94.8	1245
1246	MGCS36044_01266			*bglA*	6-phospho-beta-glucosidase BglA	94.5	1246
1247	MGCS36044_01582			-	ABC transporter permease	94.5	1246
1248	MGCS36044_03782			*tsaB*	tRNA (adenosine(37)-N6)-threonylcarbamoyltransferase complex dimerization subunit type 1 TsaB	94.5	1246
1249	MGCS36044_04048			*fhs_2*	formate--tetrahydrofolate ligase Fhs	94.5	1246
1250	MGCS36044_04002			*secE*	preprotein translocase subunit protein SecE	94.0	1250
1251	MGCS36044_02466			*ycjU*	YcjU family beta-phosphoglucomutase or related	93.8	1251
1252	MGCS36044_00298			*preA*	polyprenyl synthetase family protein PreA	93.0	1252
1253	MGCS36044_01972			-	GTP pyrophosphokinase family protein	93.0	1252
1254	MGCS36044_02204			*rnhB*	HII ribonuclease RnhB	93.0	1252
1255	MGCS36044_02844			-	phosphoglycolate phosphatase	93.0	1252
1256	MGCS36044_01766			*mvaK1*	mevalonate kinase MvaK1	92.8	1256
1257	MGCS36044_03698			-	beta-lactamase family protein	92.8	1256
1258	MGCS36044_03930	Lipo		-	CYK3 family lipoprotein putatively involved in cell division and chromosome partitioning	92.5	1258
1259	MGCS36044_00538			-	thioredoxin family protein	92.3	1259
1260	MGCS36044_01018			*lctP*	L-lactate permease LctP	92.0	1260

No.	Locus tag	Signal6P predicted[(1)]	Virulence[(2)]	Gene	Function	RPKMs[(3)]	RANK[(4)]
1261	MGCS36044_01824			*traG_1*	type IV secretory system conjugative DNA	92.0	1260
1262	MGCS36044_02416	Lipo		-	amino-acid ABC transporter substrate-binding	91.8	1262
1263	MGCS36044_00708			-	IS30 family transposase	91.5	1263
1264	MGCS36044_00838			-	class I SAM-dependent methyltransferase	91.5	1263
1265	MGCS36044_03228			-	PurR/LacI family transcriptional regulator	91.5	1263
1266	MGCS36044_03590			*asnB*	L-asparaginase AsnB	91.5	1263
1267	MGCS36044_01232			-	glutamine ABC transporter permease	91.3	1267
1268	MGCS36044_02602			*dadA*	FAD-binding oxidoreductase DadA	91.3	1267
1269	MGCS36044_03156			*cas1_2*	type I-C CRISPR-associated endonuclease Cas1	91.3	1267
1270	MGCS36044_03844			*nanA*	N-acetylneuraminate lyase	91.0	1270
1271	MGCS36044_04008			-	hypothetical protein	91.0	1270
1272	MGCS36044_00610			-	helix-turn-helix transcriptional regulator	90.0	1272
1273	MGCS36044_03068			-	DUF3165 family protein	89.8	1273
1274	MGCS36044_00126			*purH*	bifunctional formyltransferase/IMP cyclohydrolase PurH	89.5	1274
1275	MGCS36044_02686			*celB_2*	PTS cellobiose transporter IIC subunit CelB	89.5	1274
1276	MGCS36044_03142		Virulence	*yesN*	TCS DNA-binding response regulator YesN	89.5	1274
1277	MGCS36044_03596			*cbiO*	cobalt ECF transporter (A) ATPase component	88.8	1277
1278	MGCS36044_00396			-	GlpR-like transcriptional regulator protein	88.5	1278
1279	MGCS36044_01912	Secreted		*dacA_3*	secreted D,D-carboxypeptidase penicillin-binding	88.5	1278
1280	MGCS36044_02016			-	MdlB superfamily multidrug ABC transporter	88.5	1278
1281	MGCS36044_00280			-	XRE family ImmR-like transcriptional regulator	88.3	1281
1282	MGCS36044_02838			-	GNAT family N-acetyltransferase	88.3	1281
1283	MGCS36044_04044			*fctD*	glutamate formiminotransferase FctD	88.0	1283
1284	MGCS36044_04046			-	formiminotetrahydrofolate cyclodeaminase	87.8	1284
1285	MGCS36044_00870			*rsmG*	16S rRNA (guanine(527)-N(7))-methyltransferase	87.5	1285
1286	MGCS36044_02312			-	UPF0223 family protein	87.5	1285
1287	MGCS36044_02356	Lipo		-	amino acid ABC transporter substrate-binding lipoprotein	87.0	1287
1288	MGCS36044_03026			*feoB*	ferrous iron transport protein (B) FeoB	86.8	1288
1289	MGCS36044_02912			-	OFA family MFS transporter	86.0	1289
1290	MGCS36044_01750			-	MdlB family multidrug ABC transporter ATPase and	85.5	1290
1291	MGCS36044_03250			*recX*	recombination regulator RecX	85.5	1290
1292	MGCS36044_01754			*gdhA*	NADP-specific glutamate dehydrogenase GdhA	85.3	1292
1293	MGCS36044_01258			-	MFS transporter	85.0	1293
1294	MGCS36044_00938			-	ECF transporter S component	84.8	1294
1295	MGCS36044_02898			-	CPBP family intramembrane metalloprotease	84.8	1294
1296	MGCS36044_03788			*oppF_2*	oligopeptide ABC transporter ATPase OppF	84.5	1296
1297	MGCS36044_04050			*hutD*	histidine uptake and utilization HutD	84.5	1296
1298	MGCS36044_02134			*ylxM*	YlxM superfamily signal recognition particle	84.3	1298
1299	MGCS36044_02236			-	unknown	84.3	1298
1300	MGCS36044_02420			-	IS3 family transposase	84.3	1298
1301	MGCS36044_00970			-	DUF4298 domain-containing protein	83.8	1301

No.	Locus tag	Signal6P predicted[(1)]	Virulence[(2)]	Gene	Function	RPKMs[(3)]	RANK[(4)]
1302	MGCS36044_02090			-	DUF3173 family protein	83.8	1301
1303	MGCS36044_03696			-	PurR family transcriptional regulator	83.5	1303
1304	MGCS36044_02914			-	HAD family hydrolase	83.0	1304
1305	MGCS36044_01036			*brnQ_2*	branched-chain amino acid transport system II carrier protein BrnQ	82.8	1305
1306	MGCS36044_00398			-	DeoR-like transcriptional regulator protein	82.5	1306
1307	MGCS36044_01204			-	ABC transporter ATP-binding component	82.5	1306
1308	MGCS36044_01880			*copZ_1*	copper chaperone CopZ	82.5	1306
1309	MGCS36044_03558			-	drug/metabolite transporter superfamily protein	82.5	1306
1310	MGCS36044_03022			*phrB*	PhrB family deoxyribodipyrimidine photolyase	82.3	1310
1311	MGCS36044_02948			-	GIY-YIG catalytic domain-containing putative	82.0	1311
1312	MGCS36044_03320			*atoD*	butyrate-acetoacetate CoA-transferase alpha	82.0	1311
1313	MGCS36044_01668			*nifS_1*	cysteine desulfurase NifS	81.8	1313
1314	MGCS36044_04052			*potE*	PotE family amino acid transporter	81.5	1314
1315	MGCS36044_02018			-	MdlB superfamily multidrug ABC transporter ATP-binding protein	81.3	1315
1316	MGCS36044_03082			-	MdlB family multidrug ABC transporter	81.0	1316
1317	MGCS36044_04030			-	metallo-dependent amidohydrolase	81.0	1316
1318	MGCS36044_03752			-	hypothetical protein	80.8	1318
1319	MGCS36044_00944			-	RsmB/NOP family class I SAM-dependent RNA methyltransferase	79.5	1319
1320	MGCS36044_01826			-	hypothetical protein	79.3	1320
1321	MGCS36044_02468			*ykgJ*	YkgJ family cysteine cluster protein	79.3	1320
1322	MGCS36044_02688			-	DUF3284 domain-containing protein	79.3	1320
1323	MGCS36044_03162			*cas8*	type I-C CRISPR-associated protein Cas8c/Csd1	79.3	1320
1324	MGCS36044_03598			*cbiQ_1*	cobalt ECF transporter (T) transmembrane	79.3	1320
1325	MGCS36044_03848			-	DUF386 family protein	79.3	1320
1326	MGCS36044_02554			-	ABC transporter ATP-binding protein	79.0	1326
1327	MGCS36044_02690			*celC_2*	PTS cellobiose transporter IIA subunit CelC	79.0	1326
1328	MGCS36044_00608			-	site-specific integrase	78.8	1328
1329	MGCS36044_03282			*comFC*	ComFC family predicted	78.8	1328
1330	MGCS36044_02358			-	amino acid ABC transporter ATP-binding protein	78.3	1330
1331	MGCS36044_01234	Secreted		-	glutamine ABC transporter substrate-binding	78.0	1331
1332	MGCS36044_01828			*trbL*	conjugal transfer protein TrbL	78.0	1331
1333	MGCS36044_03736			-	MFS transporter	78.0	1331
1334	MGCS36044_01390			-	oligohyaluronate lyase	77.5	1334
1335	MGCS36044_04236			*cbiO2*	cobalt ABC transporter ATPase CbiO1	77.3	1335
1336	MGCS36044_02692			*celA_2*	PTS cellobiose transporter IIB subunit CelA	77.0	1336
1337	MGCS36044_02490			-	sakacin P family class II bacteriocin	76.8	1337
1338	MGCS36044_01380			-	DUF1934 domain-containing protein	76.5	1338
1339	MGCS36044_02860			*melB*	MelB family MFS transporter	76.5	1338
1340	MGCS36044_00670			-	hypothetical protein	76.3	1340
1341	MGCS36044_02322			-	aromatic acid exporter family protein	76.3	1340
1342	MGCS36044_02900			-	DUF3169 family protein	76.3	1340

No.	Locus tag	Signal6P predicted[(1)]	Virulence[(2)]	Gene	Function	RPKMs[(3)]	RANK[(4)]
1343	MGCS36044_01314			-	YdbC family protein	75.8	1343
1344	MGCS36044_01740			-	hypothetical protein	75.8	1343
1345	MGCS36044_02324			*hsdS*	type I restriction endonuclease subunit S	75.5	1345
1346	MGCS36044_00008			-	helix-turn-helix domain-containing protein	75.3	1346
1347	MGCS36044_03840			*rpiR*	RpiR family transcriptional regulator	74.8	1347
1348	MGCS36044_03084			-	SmdA family multidrug ABC transporter	74.5	1348
1349	MGCS36044_03168			*cas3*	CRISPR-associated helicase Cas3	74.3	1349
1350	MGCS36044_00550			-	nucleotidyltransferase family protein	73.8	1350
1351	MGCS36044_03094			-	SDR family oxidoreductase	73.8	1350
1352	MGCS36044_03498			-	SSRC34_2 RNA	73.5	1352
1353	MGCS36044_03660			*mutY*	A/G-specific adenine glycosylase MutY	73.5	1352
1354	MGCS36044_03906			-	5-formyltetrahydrofolate cyclo-ligase	73.5	1352
1355	MGCS36044_00108			*recO*	DNA repair protein RecO	73.0	1355
1356	MGCS36044_04234			*cbiQ_2*	cobalt ABC transporter permease CbiQ	73.0	1355
1357	MGCS36044_01222			-	acyl dehydratase	72.8	1357
1358	MGCS36044_00278			-	HNH endonuclease	72.5	1358
1359	MGCS36044_00602			-	L13_leader	72.5	1358
1360	MGCS36044_01868			-	hypothetical protein	72.3	1360
1361	MGCS36044_04244			*pqqF*	pitrilysin family predicted Zn-dependent	72.3	1360
1362	MGCS36044_04042			*hutU*	urocanate hydratase HutU	72.0	1362
1363	MGCS36044_04194			-	DUF4097 family protein	72.0	1362
1364	MGCS36044_04216			*mnmG*	tRNA uridine-5-carboxymethylaminomethyl(34)	72.0	1362
1365	MGCS36044_00458			-	PhoE family phosphoglycerate mutase. Region of difference 36044_ROD.2, putative MGE	71.8	1365
1366	MGCS36044_02310			-	inositol monophosphatase family protein	71.8	1365
1367	MGCS36044_03496			*cof*	Cof family hydrolase	71.8	1365
1368	MGCS36044_03322			*atoA*	acyl CoA:acetate/3-ketoacid CoA transferase beta	71.5	1368
1369	MGCS36044_02414			-	amino acid ABC transporter permease	71.3	1369
1370	MGCS36044_03790			*oppD_2*	oligopeptide ABC transporter ATPase OppD	70.8	1370
1371	MGCS36044_01822			-	hypothetical protein	70.5	1371
1372	MGCS36044_01034			*nrdE_1*	class 1b ribonucleoside-diphosphate reductase	70.3	1372
1373	MGCS36044_02944	Secreted		*plsC*	secreted 1-acyl-sn-glycerol-3-phosphate	70.3	1372
1374	MGCS36044_00738			-	alpha-amylase family glycosyl hydrolase	69.8	1374
1375	MGCS36044_02154			*xerS*	site-specific tyrosine recombinase XerS	69.8	1374
1376	MGCS36044_00748			*lytR*	TCS DNA-binding response regulator LytR	69.5	1376
1377	MGCS36044_01340	Lipo		-	DUF4430 domain-containing lipoprotein	69.5	1376
1378	MGCS36044_00770			-	putative metal homeostasis protein	69.3	1378
1379	MGCS36044_01370			-	FMN-binding domain containing L-lactate oxidase	69.0	1379
1380	MGCS36044_01836			-	nucleotidyl transferase AbiEii/AbiGii toxin	69.0	1379
1381	MGCS36044_00600			-	IS30 family transposase	68.8	1381
1382	MGCS36044_01906			-	unknown	68.8	1381
1383	MGCS36044_00656			-	Txe/YoeB family addiction module toxin	68.3	1383

No.	Locus tag	Signal6P predicted[(1)]	Virulence[(2)]	Gene	Function	RPKMs[(3)]	RANK[(4)]
1384	MGCS36044_00598			-	helix-turn-helix transcriptional regulator	68.0	1384
1385	MGCS36044_02856			-	beta-D-glucuronidase	68.0	1384
1386	MGCS36044_01720			*rimM*	ribosome maturation factor RimM	67.8	1386
1387	MGCS36044_03200			-	permease	67.8	1386
1388	MGCS36044_03904			*glpG*	GlpG family membrane associated serine protease	67.8	1386
1389	MGCS36044_00514		Virulence	*mga*	M protein trans-acting positive regulator Mga	67.5	1389
1390	MGCS36044_01244			*rnc*	ribonuclease III Rnc	67.5	1389
1391	MGCS36044_03308			*priA*	primosomal protein PriA	67.5	1389
1392	MGCS36044_03154			*cas2_2*	CRISPR-associated endonuclease Cas2	67.3	1392
1393	MGCS36044_01126		Virulence	*silA*	TCS DNA-binding response regulator SilA	67.0	1393
1394	MGCS36044_00034			-	IS30 family transposase	66.8	1394
1395	MGCS36044_01236			*glnQ_1*	glutamine ABC transporter ATPase GlnQ	66.8	1394
1396	MGCS36044_02360			-	amino acid ABC transporter permease	66.8	1394
1397	MGCS36044_01724			*trxB_1*	NAD(P)/FAD-dependent oxidoreductase	66.5	1397
1398	MGCS36044_03024			-	FeoB associated cysteine-rich protein	66.5	1397
1399	MGCS36044_03878			-	hypothetical protein	66.5	1397
1400	MGCS36044_01452			-	GntR family transcriptional regulator	66.0	1400
1401	MGCS36044_02170			-	BaeS family TCS histidine kinase sensor	66.0	1400
1402	MGCS36044_00746			*lytS*	TCS sensor histidine kinase LytS	65.8	1402
1403	MGCS36044_01030			*nrdF_1*	ribonucleotide-diphosphate reductase subunit	65.8	1402
1404	MGCS36044_02030			-	vicinal oxygen chelate (VOC) family protein	65.8	1402
1405	MGCS36044_04180			-	hypothetical protein	65.8	1402
1406	MGCS36044_04256			*trpS*	tryptophanyl-tRNA synthetase	65.8	1402
1407	MGCS36044_02592			-	hypothetical protein	65.5	1407
1408	MGCS36044_03978			-	IS30 family transposase	65.0	1408
1409	MGCS36044_02694			*bglG_2*	transcription antiterminator BglG	64.8	1409
1410	MGCS36044_01264			*bglF*	PTS beta-glucoside transporter IIBCA component	64.5	1410
1411	MGCS36044_01864			-	hypothetical protein	64.5	1410
1412	MGCS36044_03184			-	MOP/MATE family multidrug-resistance efflux	64.5	1410
1413	MGCS36044_03856			*nanE*	N-acetylmannosamine-6-phosphate 2-epimerase	64.5	1410
1414	MGCS36044_01780			-	HTH domain-containing putative transcriptional regulator	64.3	1414
1415	MGCS36044_01730			*fruR*	fructose operon transcriptional repressor	64.0	1415
1416	MGCS36044_04204			-	helix-turn-helix domain-containing transcriptional regulator	64.0	1415
1417	MGCS36044_03326			*gntT*	GntT family H+/gluconate symporter or related	63.8	1417
1418	MGCS36044_01310			-	PrtC family collagenase-like protease	63.5	1418
1419	MGCS36044_02352			*fieF*	FieF family cation diffusion facilitator family	63.5	1418
1420	MGCS36044_02858			-	sugar kinase	63.5	1418
1421	MGCS36044_03850			*ugpE_2*	carbohydrate ABC transporter permease UgpE-like	63.5	1418
1422	MGCS36044_03780			*rimI*	ribosomal-protein-alanine N-acetyltransferase	63.3	1422
1423	MGCS36044_02164			-	polyprenyl synthetase family protein	62.8	1423

No.	Locus tag	Signal6P predicted[1]	Virulence[2]	Gene	Function	RPKMs[3]	RANK[4]
1424	MGCS36044_02172			-	OmpR family TCS DNA-binding response regulator	62.8	1423
1425	MGCS36044_02924			-	MFS transporter	62.8	1423
1426	MGCS36044_03412			-	hypothetical protein	62.8	1423
1427	MGCS36044_00286			-	1,4-dihydroxy-2-naphthoate octaprenyltransferase	62.5	1427
1428	MGCS36044_01908			-	DUF3173 domain-containing protein	62.3	1428
1429	MGCS36044_02188			-	DUF3307 domain-containing protein	62.3	1428
1430	MGCS36044_03792			*oppC_2*	oligopeptide ABC transporter permease OppC	62.0	1430
1431	MGCS36044_03880			-	LytTR family transcriptional regulator	62.0	1430
1432	MGCS36044_04262			*yfhO*	YfhO family protein	62.0	1430
1433	MGCS36044_00942			*yhcC*	YhcC family Fe-S oxidoreductase	61.5	1433
1434	MGCS36044_01594	Lipo		-	ABC transporter substrate-binding lipoprotein	61.5	1433
1435	MGCS36044_02130			-	hypothetical protein	61.5	1433
1436	MGCS36044_02786			-	ORF6N domain-containing protein	61.5	1433
1437	MGCS36044_00958	Lipo		*fhuD*	iron-hydroxamate ABC transporter	61.3	1437
1438	MGCS36044_02658			*malA*	maltodextrose utilization protein MalA	61.3	1437
1439	MGCS36044_03892			-	ABC transporter ATPase/permease	61.0	1439
1440	MGCS36044_04258			*yitT*	membrane anchor protein YitT	61.0	1439
1441	MGCS36044_01494			*rodA*	rod shape-determining protein RodA	60.3	1441
1442	MGCS36044_02982			-	IS982 family transposase	60.3	1441
1443	MGCS36044_03318			*atoB*	3-ketoacyl-CoA thiolase/acetyl-CoA	60.3	1441
1444	MGCS36044_00164			-	IS30 family transposase	59.8	1444
1445	MGCS36044_03600			-	ECF transporter (S) specificity component	59.8	1444
1446	MGCS36044_03852			*ugpA_2*	carbohydrate ABC transporter permease UgpA-like	59.8	1444
1447	MGCS36044_00960			*fhuA*	ferrichrome ABC transporter ATP-binding protein	59.3	1447
1448	MGCS36044_03080			-	hypothetical protein	59.3	1447
1449	MGCS36044_04246			*pqqL*	pitrilysin family predicted Zn-dependent peptidase	59.3	1447
1450	MGCS36044_00596			-	hypothetical protein	59.0	1450
1451	MGCS36044_01032			*nrdI_2*	ribonucleotide reductase assembly protein NrdI	59.0	1450
1452	MGCS36044_01478			*gstA*	GstA superfamily glutathione-dependent	58.5	1452
1453	MGCS36044_01970			*yjbK*	YbjK superfamily CYTH domain-containing	58.5	1452
1454	MGCS36044_03868		Virulence	*fasA*	TCS response regulator protein	58.3	1454
1455	MGCS36044_03910			-	IS30 family transposase	58.3	1454
1456	MGCS36044_00228			-	MGCS36044_00228	58.0	1456
1457	MGCS36044_00662			-	conjugal transfer protein	58.0	1456
1458	MGCS36044_03202			*aroE*	shikimate dehydrogenase AroE	57.5	1458
1459	MGCS36044_03960			*bglG_3*	BglG family transcription antiterminator	57.5	1458
1460	MGCS36044_01870			-	XRE family HTH-type transcriptional regulator	57.3	1460
1461	MGCS36044_02308			*rsmF*	RsmF family rRNA cytosine-C5-methyltransferase	57.3	1460
1462	MGCS36044_00456			-	aminoglycoside 6-adenylyltransferase	57.0	1462
1463	MGCS36044_02046			-	SalY superfamily ABC transporter permease	57.0	1462

No.	Locus tag	Signal6P predicted[(1)]	Virulence[(2)]	Gene	Function	RPKMs[(3)]	RANK[(4)]
1464	MGCS36044_00536			-	hypothetical protein	56.8	1464
1465	MGCS36044_03802			-	YgaE family protein	56.5	1465
1466	MGCS36044_00906			-	SpoU family RNA methyltransferase	56.3	1466
1467	MGCS36044_03404			*ypbQ*	YpbQ family isoprenylcysteine carboxyl	56.3	1466
1468	MGCS36044_00018			-	RNA-binding S4 domain-containing protein	56.0	1468
1469	MGCS36044_01210			*dhaQ*	DhaKLM operon coactivator DhaQ	55.8	1469
1470	MGCS36044_02068			*dppF*	dipeptide ABC transport system ATP-binding DppF	55.8	1469
1471	MGCS36044_00562			-	major facilitator transporter family protein	55.5	1471
1472	MGCS36044_02422			-	GNAT family N-acetyltransferase	55.5	1471
1473	MGCS36044_03324			-	3-hydroxybutyrate dehydrogenase	55.5	1471
1474	MGCS36044_01010			-	PhnB family glyoxalase/bleomycin	55.3	1474
1475	MGCS36044_01838			-	type IV toxin-antitoxin system AbiEi family	55.3	1474
1476	MGCS36044_03958			*ulaG*	L-ascorbate utilization protein (G) UlaG	55.3	1474
1477	MGCS36044_04178			-	IS982 family transposase	55.3	1474
1478	MGCS36044_02428			-	PyrR RNA	55.0	1478
1479	MGCS36044_02684			-	hypothetical protein	55.0	1478
1480	MGCS36044_00454			-	type II toxin-antitoxin system PemK/MazF family	54.8	1480
1481	MGCS36044_01486			*pheA*	chorismate mutase PheA	54.8	1480
1482	MGCS36044_03890			*queH*	epoxyqueuosine reductase QueH	54.8	1480
1483	MGCS36044_03720			-	BaeS family TCS sensor histidine kinase	54.5	1483
1484	MGCS36044_00430			-	ATP-binding cassette domain-containing protein	54.3	1484
1485	MGCS36044_04192			-	DUF1700 domain-containing protein	54.3	1484
1486	MGCS36044_00384			-	PTS sugar transporter subunit IIC	54.0	1486
1487	MGCS36044_00392	Lipo		-	SignalP6 predicted lipid anchoring signal peptide. BMP family ABC transporter substrate-binding	54.0	1486
1488	MGCS36044_00664			-	cytoplasmic protein	53.5	1488
1489	MGCS36044_03494			*azgA*	AzgA family permease	53.5	1488
1490	MGCS36044_01876			*copY_1*	copper transport repressor CopY	53.3	1490
1491	MGCS36044_02166			-	NAD(P)/FAD-dependent oxidoreductase	53.3	1490
1492	MGCS36044_02418			-	GatA family amidase	53.3	1490
1493	MGCS36044_00528			*lrp*	PucR family transcriptional regulator/leucine	53.0	1493
1494	MGCS36044_01580			*ybgA*	YbgA family DUF1722 domain-containing protein	53.0	1493
1495	MGCS36044_02724			*paaI*	PaaI family thioesterase	53.0	1493
1496	MGCS36044_00842			-	MerR family transcriptional regulator	52.8	1496
1497	MGCS36044_02808			-	TetR/AcrR family transcriptional regulator	52.8	1496
1498	MGCS36044_02926			*rsuA_2*	rsuA_2ribosomal small subunit pseudouridine synthase RsuA	52.5	1498
1499	MGCS36044_03990			-	IS982 family transposase	52.5	1498
1500	MGCS36044_03894			-	ABC transporter ATPase/permease	52.3	1500
1501	MGCS36044_00510			-	PreQ1	52.0	1501
1502	MGCS36044_02584			*yloA*	YloA family predicted ribosome quality control	52.0	1501
1503	MGCS36044_03634			*scrR*	sucrose operon repressor ScrR	51.8	1503

No.	Locus tag	Signal6P predicted[(1)]	Virulence[(2)]	Gene	Function	RPKMs[(3)]	RANK[(4)]
1504	MGCS36044_03722			-	OmpR family TCS DNA-binding response regulator	51.8	1503
1505	MGCS36044_01856			-	DUF5965 family protein	51.5	1505
1506	MGCS36044_02788			-	DUF1413 domain-containing protein	51.5	1505
1507	MGCS36044_03510			-	acyltransferase family protein	51.5	1505
1508	MGCS36044_03912			-	Mga-related helix-turn-helix domain-containing	51.5	1505
1509	MGCS36044_00860			*metP_1*	methionine ABC transporter permease MetP	51.3	1509
1510	MGCS36044_03002	Secreted		-	extracellular cell surface putative nucleotidase	51.3	1509
1511	MGCS36044_04172			-	MFS transporter	51.3	1509
1512	MGCS36044_02916			-	GNAT family N-acetyltransferase	51.0	1512
1513	MGCS36044_01852			-	hypothetical protein	50.8	1513
1514	MGCS36044_02190			-	SatD family protein	50.8	1513
1515	MGCS36044_03580			-	cysteine hydrolase	50.8	1513
1516	MGCS36044_01736			-	putative peptidoglycan hydrolase	50.5	1516
1517	MGCS36044_01818			-	hypothetical protein	50.5	1516
1518	MGCS36044_02798			-	MdlB family multidrug ABC transporter ATPase and permease	50.5	1516
1519	MGCS36044_03028			*feoA*	ferrous iron transport protein (A) FeoA	50.5	1516
1520	MGCS36044_01858			-	DUF5945 family protein	50.3	1520
1521	MGCS36044_02234			*asd*	aspartate-semialdehyde dehydrogenase	50.3	1520
1522	MGCS36044_02934	Lipo		*blaA*	beta-lactamase-related serine hydrolase	50.3	1520
1523	MGCS36044_04218			-	NUDIX domain-containing protein	50.3	1520
1524	MGCS36044_04248			*ybcJ*	ribosome associated protein YbcJ	50.3	1520
1525	MGCS36044_01846			-	SNF2-related protein	49.8	1525
1526	MGCS36044_00162			-	MATE family multidrug efflux transporter	49.5	1526
1527	MGCS36044_01276			-	cupin domain-containing protein	49.5	1526
1528	MGCS36044_03350			*ansP*	AnsP family L-asparagine transporter and related	49.0	1528
1529	MGCS36044_01282	Lipo		*pstS*	phosphate ABC transporter substrate-binding	48.8	1529
1530	MGCS36044_02572	Secreted		-	putative secreted protein	48.3	1530
1531	MGCS36044_01834			-	phage tail tip lysozyme	48.0	1531
1532	MGCS36044_03870		Virulence	*fasC*	TCS histidine kinase	48.0	1531
1533	MGCS36044_00418			-	MccC family LD-carboxypeptidase	47.8	1533
1534	MGCS36044_01820			-	CPBP family intramembrane metalloprotease	47.8	1533
1535	MGCS36044_02562			*phnK*	PhnK family ABC transporter ATPase component	47.8	1533
1536	MGCS36044_00120			*purM*	phosphoribosylformylglycinamidine cyclo-ligase	47.0	1536
1537	MGCS36044_02648			-	IS30 family transposase	47.0	1536
1538	MGCS36044_03286			-	YIH1 family putative translation regulator	47.0	1536
1539	MGCS36044_03794			*oppB_2*	oligopeptide ABC transporter permease OppB	47.0	1536
1540	MGCS36044_00294			*cydD*	thiol reductant ABC exporter subunit CydD	46.8	1540
1541	MGCS36044_02326			-	ABC transporter permease	46.8	1540
1542	MGCS36044_03766			-	MerR/SoxR family transcriptional regulator	46.8	1540
1543	MGCS36044_00296			*cdyC*	thiol reductant ABC exporter subunit CydC	46.3	1543
1544	MGCS36044_01700			*carA*	carbamoyl-phosphate synthase small subunit CarA	46.3	1543

No.	Locus tag	Signal6P predicted[(1)]	Virulence[(2)]	Gene	Function	RPKMs[(3)]	RANK[(4)]
1545	MGCS36044_00858			*metN_1*	methionine ABC transporter ATP-binding protein	46.0	1545
1546	MGCS36044_04078			*catE*	catechol-2,3-dioxygenase CatE	45.8	1546
1547	MGCS36044_00136			-	DUF4041 domain-containing protein	45.5	1547
1548	MGCS36044_02110			-	hypothetical protein	45.5	1547
1549	MGCS36044_04148			-	cadmium efflux system accessory protein	45.0	1549
1550	MGCS36044_04186			-	thioredoxin family protein	44.8	1550
1551	MGCS36044_02076	Lipo		*dppA*	dipeptide-binding ABC transport system	44.5	1551
1552	MGCS36044_02806			-	ECF transporter S component	44.3	1552
1553	MGCS36044_02222			-	Uup family ATPase components of ABC transporters with duplicated ATPase domains	44.0	1553
1554	MGCS36044_02978			*niaR*	niacin-responsive transcriptional repressor	44.0	1553
1555	MGCS36044_01940			*maeR*	TCS signal transduction response regulator MaeR	43.8	1555
1556	MGCS36044_02108			-	hypothetical protein	43.8	1555
1557	MGCS36044_01702			*carB*	carbamoyl-phosphate synthase large subunit CarB	43.5	1557
1558	MGCS36044_01832			-	AAA family ATPase	43.5	1557
1559	MGCS36044_02328			-	ABC transporter ATP-binding protein LolD-like	43.5	1557
1560	MGCS36044_03982	Secreted		-	putative secreted protein	43.5	1557
1561	MGCS36044_00138			-	hypothetical protein	42.3	1561
1562	MGCS36044_00274			-	helix-turn-helix transcriptional regulator	42.3	1561
1563	MGCS36044_01948			*eriC*	EriC family voltage gated chloride channel	42.3	1561
1564	MGCS36044_01830			-	PrgI family protein	42.0	1564
1565	MGCS36044_02526			-	hypothetical protein	42.0	1564
1566	MGCS36044_03578			-	DUF156 family protein	42.0	1564
1567	MGCS36044_02570	Lipo		-	putative lipoprotein	41.8	1567
1568	MGCS36044_02512			*cas1_1*	type II CRISPR-associated endonuclease Cas1	41.5	1568
1569	MGCS36044_03754			-	type IV secretion system DNA-binding	41.5	1568
1570	MGCS36044_04174			-	arginase family protein	41.5	1568
1571	MGCS36044_00422			*proX*	prolyl-tRNA synthetase associated	41.3	1571
1572	MGCS36044_00016			-	oligosaccharide flippase family protein	41.0	1572
1573	MGCS36044_02702			-	hypothetical protein	41.0	1572
1574	MGCS36044_04016			*mutT*	8-oxo-dGTP diphosphatase, DNA mismatch repair	41.0	1572
1575	MGCS36044_00348			-	DUF4651 domain-containing protein	40.8	1575
1576	MGCS36044_02424			*pyrE*	orotate phosphoribosyltransferase PyrE	40.8	1575
1577	MGCS36044_02946			*trmN6*	tRNA1(Val) A37 N6-methylase TrmN6	40.8	1575
1578	MGCS36044_03922			*resA*	TlpA family protein disulfide reductase	40.8	1575
1579	MGCS36044_03926	Secreted	Virulence	*speG*	streptococcal pyrogenic exotoxin (G) SpeG	40.8	1575
1580	MGCS36044_00460			-	helix-turn-helix transcriptional regulator	40.5	1580
1581	MGCS36044_01158	Secreted		-	secreted transglutaminase	40.5	1580
1582	MGCS36044_01334			-	GH25 muramidase superfamily lysozyme	40.5	1580
1583	MGCS36044_01862			-	DUF5960 family protein	40.5	1580
1584	MGCS36044_01440			-	DUF1003 domain-containing protein	40.3	1584
1585	MGCS36044_01816			-	arsenate reductase	40.3	1584

No.	Locus tag	Signal6P predicted[(1)]	Virulence[(2)]	Gene	Function	RPKMs[(3)]	RANK[(4)]
1586	MGCS36044_00622			-	DNA cytosine methyltransferase	40.0	1586
1587	MGCS36044_02902			-	transcriptional regulator	40.0	1586
1588	MGCS36044_03232			*mngB*	alpha-mannosidase MngB	40.0	1586
1589	MGCS36044_03872		Virulence	*fasB*	TCS histidine kinase	40.0	1586
1590	MGCS36044_04150			-	DNA-binding HTH domain-containing	40.0	1586
1591	MGCS36044_00672	Pilin		-	putative lipoprotein	39.8	1591
1592	MGCS36044_00954			*fhuG*	iron ABC transporter permease FhuG	39.8	1591
1593	MGCS36044_00956			*fhuB*	iron ABC transporter permease FhuB	39.3	1593
1594	MGCS36044_04220			-	Spd-sr37 RNA	39.3	1593
1595	MGCS36044_01698			*pyrB*	aspartate transcarbomoylase PyrB	39.0	1595
1596	MGCS36044_01262			*bglG_1*	transcriptional antiterminator BglG	38.8	1596
1597	MGCS36044_02508			*csn2*	CRISPR-associated protein Csn2	38.8	1596
1598	MGCS36044_02564			-	ABC transporter permease component	38.8	1596
1599	MGCS36044_02716			-	MFS transporter	38.8	1596
1600	MGCS36044_01578			*osmC*	YhfA family OsmC-related salt-stress induced	38.5	1600
1601	MGCS36044_00014			*trcF*	transcription-repair coupling factor TcrF	38.0	1601
1602	MGCS36044_02660			*malD*	maltodextrin transport system permease protein	38.0	1601
1603	MGCS36044_00122			*purN*	phosphoribosylglycinamide formyltransferase	37.8	1603
1604	MGCS36044_02182			*citC*	citrate lyase ligase CitC	37.8	1603
1605	MGCS36044_02626			*miaA*	tRNA (adenosine(37)-N6)-dimethylallyltransferase	37.5	1605
1606	MGCS36044_00404			*celB_1*	PTS transporter cellobiose-specific IIC	37.3	1606
1607	MGCS36044_02100			-	XRE family HTH-type transcriptional regulator	37.3	1606
1608	MGCS36044_01952			-	NanC-like SGNH/GDSL hydrolase family protein	36.5	1608
1609	MGCS36044_01592			*aspB*	aspartate aminotransferase AspB	36.3	1609
1610	MGCS36044_01812			-	DNA (cytosine-5-)-methyltransferase	36.3	1609
1611	MGCS36044_00934			*trml*	TrmL family tRNA (cytosine34-2'-O-)-methyl	36.0	1611
1612	MGCS36044_02362			*phnA*	PnhA family zinc ribbon domain-containing	36.0	1611
1613	MGCS36044_03182			-	MarR family winged helix-turn-helix	36.0	1611
1614	MGCS36044_01854			-	toprim domain-containing protein	35.8	1614
1615	MGCS36044_01988			*guaC*	guanosine 5'-monophosphate oxidoreductase GuaC	35.8	1614
1616	MGCS36044_03456		Virulence	-	YSIRK-targeted surface antigen transcriptional	35.8	1614
1617	MGCS36044_03658			-	hypothetical protein	35.8	1614
1618	MGCS36044_00668			-	Cro/CI family transcriptional regulator	35.5	1618
1619	MGCS36044_00794	Secreted		*dacA_1*	secreted D-alanyl-D-alanine carboxypeptidase	35.5	1618
1620	MGCS36044_00864			*sstT*	serine/threonine transporter SstT	35.3	1620
1621	MGCS36044_02510			*cas2_1*	CRISPR-associated endoribonuclease Cas2	35.0	1621
1622	MGCS36044_03610	Lipo		*htsA*	heme ABC transporter substrate-binding	35.0	1621
1623	MGCS36044_02426			*pyrF*	orotidine-5'-phosphate decarboxylase PyrF	34.8	1623
1624	MGCS36044_03556			-	GlsB/YeaQ/YmgE family stress response membrane protein	34.8	1623
1625	MGCS36044_02158			-	1,4-dihydroxy-2-naphthoate polyprenyltransferase	34.5	1625
1626	MGCS36044_03194	Lipo		-	putative lipoprotein	34.3	1626

No.	Locus tag	Signal6P predicted[(1)]	Virulence[(2)]	Gene	Function	RPKMs[(3)]	RANK[(4)]
1627	MGCS36044_00478			-	site-specific integrase	34.0	1627
1628	MGCS36044_03718			-	OmpR family TCS DNA-binding response regulator	34.0	1627
1629	MGCS36044_00118			*purF*	amidophosphoribosyltransferase PurF	33.8	1629
1630	MGCS36044_03774			-	hypothetical protein	33.8	1629
1631	MGCS36044_00420			-	hypothetical protein	33.5	1631
1632	MGCS36044_00124			*vanZ*	VanZ family putative glycopeptide antibiotic	33.3	1632
1633	MGCS36044_02728	Lipo		*nlpA*	MetQ/NlpA family ABC transporter substrate-binding lipoprotein	33.0	1633
1634	MGCS36044_02706			-	DUF4044 domain-containing protein	32.8	1634
1635	MGCS36044_03612	Secreted		*shp*	heme-binding secreted protein Shp	32.8	1634
1636	MGCS36044_02566			-	ABC transporter substrate binding component	32.5	1636
1637	MGCS36044_04146			*cadD*	CadD family cadmium resistance transporter	32.5	1636
1638	MGCS36044_01168			-	hypothetical protein	32.3	1638
1639	MGCS36044_03222			-	hypothetical protein	32.3	1638
1640	MGCS36044_03712			-	Xre family helix-turn-helix transcriptional regulator	32.3	1638
1641	MGCS36044_03830			-	hypothetical protein	32.3	1638
1642	MGCS36044_02726			-	nucleoside phosphorylase family protein	32.0	1642
1643	MGCS36044_02732			*dcm*	DNA (cytosine-5-)-methyltransferase Dcm	32.0	1642
1644	MGCS36044_03188			-	hypothetical protein	32.0	1642
1645	MGCS36044_00402			*celA_1*	PTS transporter cellobiose-specific IIB	31.8	1645
1646	MGCS36044_00412			-	hypothetical protein	31.8	1645
1647	MGCS36044_01904			-	Cro/CI family transcriptional regulator	31.8	1645
1648	MGCS36044_01696			*pyrP*	uracil permease protein PyrP	31.5	1648
1649	MGCS36044_00438			-	bacteriocin immunity protein	31.3	1649
1650	MGCS36044_00654			-	replication initiation factor domain-containing	31.3	1649
1651	MGCS36044_00666			-	hypothetical protein	31.0	1651
1652	MGCS36044_02176			-	ABC transporter ATPase	31.0	1651
1653	MGCS36044_02514			*cas9*	type II CRISPR RNA-guided endonuclease Cas9	31.0	1651
1654	MGCS36044_02080			-	IS30 family transposase	30.8	1654
1655	MGCS36044_03938			-	CHY zinc finger domain-containing protein	30.8	1654
1656	MGCS36044_03030			-	amino acid ABC transporter ATP-binding protein	30.3	1656
1657	MGCS36044_04094			-	hypothetical protein	30.3	1656
1658	MGCS36044_04176			-	radical SAM protein	30.3	1656
1659	MGCS36044_02666	Lipo		*malX*	maltose/maltodextrin-binding lipoprotein MalX	30.0	1659
1660	MGCS36044_03716			-	BaeS family TCS sensor histidine kinase	30.0	1659
1661	MGCS36044_01284			*pstC*	phosphate ABC transporter, permease protein	29.8	1661
1662	MGCS36044_02698			*bglB_1*	6-phospho-beta-glucosidase BglB	29.8	1661
1663	MGCS36044_03574			-	CoA-disulfide reductase	29.8	1661
1664	MGCS36044_00630			-	ATP-binding protein	29.5	1664
1665	MGCS36044_01942			*maeK*	TCS signal transduction sensor histidine kinase	29.3	1665
1666	MGCS36044_02174			-	ABC transporter permease	29.3	1665
1667	MGCS36044_04198			-	hypothetical protein	29.3	1665

No.	Locus tag	Signal6P predicted[(1)]	Virulence[(2)]	Gene	Function	RPKMs[(3)]	RANK[(4)]
1668	MGCS36044_03032			-	amino acid ABC transporter permease	29.0	1668
1669	MGCS36044_01118			-	hypothetical protein	28.8	1669
1670	MGCS36044_02734			-	hypothetical protein	28.8	1669
1671	MGCS36044_03410			*nptA*	NtpA family Na/Pi cotransporter	28.8	1669
1672	MGCS36044_00116			*purL*	phosphoribosylformylglycinamidine synthase PurL	28.5	1672
1673	MGCS36044_01450	Secreted		*adcA*	zinc ABC transporter secreted substrate-binding	28.3	1673
1674	MGCS36044_02044			-	LolD superfamily ABC transporter ATP-binding	28.3	1673
1675	MGCS36044_03614	Secreted	Virulence	*shr*	heme-binding secreted protein Shr	28.3	1673
1676	MGCS36044_03908	Secreted	Virulence	*fbpB*	cell surface fibronectin binding protein (B)	28.0	1676
1677	MGCS36044_01382			-	SSRC10 RNA	27.8	1677
1678	MGCS36044_01800			-	Possible RNA. L10_leader	27.5	1678
1679	MGCS36044_04130			-	Uup family of ABC transporter with duplicated	27.5	1678
1680	MGCS36044_00628			-	SIR2 family protein	27.3	1680
1681	MGCS36044_01012			-	GloA family glyoxalase/bleomycin	27.3	1680
1682	MGCS36044_00626			-	MvaI/BcnI family restriction endonuclease	27.0	1682
1683	MGCS36044_00134			*purK*	5-(carboxyamino)imidazole ribonucleotide	26.8	1683
1684	MGCS36044_01994			*pbuX*	xanthine permease PbuX	26.8	1683
1685	MGCS36044_02084			*kefB*	Kef-type K+ transporter membrane component KefB	26.8	1683
1686	MGCS36044_02740			-	resolvase/recombinase family protein	26.8	1683
1687	MGCS36044_02976			*niaX*	niacin transporter NiaX	26.8	1683
1688	MGCS36044_03602			-	CydD-related ABC transporter ATPase/permease	26.8	1683
1689	MGCS36044_03632			*scrB*	sucrose-6-phosphate hydrolase ScrB	26.5	1689
1690	MGCS36044_01166			-	ABC transporter ATP-binding protein	26.3	1690
1691	MGCS36044_03936			*bioY*	biotin transporter BioY	26.3	1690
1692	MGCS36044_01956			*radC*	DNA repair protein RadC	25.8	1692
1693	MGCS36044_01502			*serB*	phosphoserine phosphatase SerB	25.5	1693
1694	MGCS36044_03220			-	COG3537 superfamily putative	25.5	1693
1695	MGCS36044_02042			*acrA*	AcrA superfamily multidrug efflux pump	25.3	1695
1696	MGCS36044_02804			*ecfT*	ECF transporter transmembrane protein EcfT	25.3	1695
1697	MGCS36044_00724			-	aspartate kinase	25.0	1697
1698	MGCS36044_03608			*htsB*	heme ABC transporter permease HtsB	25.0	1697
1699	MGCS36044_01212			*dhaS*	dihydroxyacetone kinase transcriptional	24.8	1699
1700	MGCS36044_02196			-	IS982 family transposase	24.8	1699
1701	MGCS36044_02350			*alsT*	sodium:alanine symporter family protein	24.8	1699
1702	MGCS36044_03572			-	rhodanese-like domain-containing protein	24.5	1702
1703	MGCS36044_01286			*pstA*	phosphate ABC transporter, permease protein PstA	24.3	1703
1704	MGCS36044_01814			-	hypothetical protein	24.3	1703
1705	MGCS36044_03692			-	carbohydrate ABC transporter permease UgpE-like	24.3	1703
1706	MGCS36044_03768			*dnaQ*	DNA polymerase III epsilon subunit DnaQ	24.3	1703
1707	MGCS36044_04072			-	Spy491738 RNA	24.3	1703
1708	MGCS36044_02736			-	RepA N-terminal domain-containing protein	24.0	1708

No.	Locus tag	Signal6P predicted[(1)]	Virulence[(2)]	Gene	Function	RPKMs[(3)]	RANK[(4)]
1709	MGCS36044_03686			-	alpha-glycosidase	24.0	1708
1710	MGCS36044_02802			*ecfA2*	EcfA2 family ECF transporter ATPase	23.8	1710
1711	MGCS36044_03198			-	hypothetical protein	23.8	1710
1712	MGCS36044_03896			-	MarR family transcriptional regulator	23.8	1710
1713	MGCS36044_00444			*flaR*	DNA topology modulation protein	23.5	1713
1714	MGCS36044_01718	Secreted		*sdrD*	SdrD superfamily cell surface extracellular	23.5	1713
1715	MGCS36044_02098			-	hypothetical protein	23.5	1713
1716	MGCS36044_02168			-	Gx transporter family protein	23.5	1713
1717	MGCS36044_00012			*pth*	aminoacyl-tRNA hydrolase Pth	23.3	1717
1718	MGCS36044_00320			*comYA*	competence system type II/IV secretion system	23.3	1717
1719	MGCS36044_00506			-	ECF transporter S component	23.3	1717
1720	MGCS36044_01288			*pstB*	phosphate ABC transporter ATP-binding protein	23.3	1717
1721	MGCS36044_02330			*tetR*	TetR family transcriptional regulator	23.3	1717
1722	MGCS36044_02112			-	helix-turn-helix domain-containing protein	23.0	1722
1723	MGCS36044_00436			-	Maf family protein	22.8	1723
1724	MGCS36044_01860			-	MGCS36044_018hypothetical protein60	22.8	1723
1725	MGCS36044_03316			-	LysR family transcriptional regulator	22.8	1723
1726	MGCS36044_04182			-	Rgg/GadR/MutR family transcriptional regulator	22.8	1723
1727	MGCS36044_00646			-	conjugal transfer protein	22.5	1727
1728	MGCS36044_03714	Lipo		-	chromosome assembly-related lipoprotein	22.5	1727
1729	MGCS36044_01418			-	transposase	22.3	1729
1730	MGCS36044_03750			-	HTH cro/C1-type domain-containing protein	22.3	1729
1731	MGCS36044_00660			-	MGCS36044_006FtsK/SpoIIIE domain-containing protein60	22.0	1731
1732	MGCS36044_03748			-	hypothetical protein	22.0	1731
1733	MGCS36044_02990			-	Rgg/GadR/MutR family transcriptional regulator	21.8	1733
1734	MGCS36044_04074			-	hypothetical protein	21.8	1733
1735	MGCS36044_00650			-	conjugal transfer protein	21.5	1735
1736	MGCS36044_03230			-	metal-independent alpha-mannosidase	21.5	1735
1737	MGCS36044_00382			*ridA*	RidA family protein	21.3	1737
1738	MGCS36044_01990			-	unknown	21.3	1737
1739	MGCS36044_04196			-	YhgE/Pip domain-containing protein	21.3	1737
1740	MGCS36044_00262			-	FRG domain-containing protein	21.0	1740
1741	MGCS36044_01290			*phoU_1*	phosphate signaling complex protein PhoU	21.0	1740
1742	MGCS36044_03234			-	ParB-like nuclease domain-containing protein	20.8	1742
1743	MGCS36044_02160	Lipo		*apbE*	ApbE family thiamine biosynthesis lipoprotein	20.5	1743
1744	MGCS36044_03694			-	carbohydrate ABC transporter permease UgpA-like	20.5	1743
1745	MGCS36044_00130			*purD*	phosphoribosylamine--glycine ligase PurD	20.3	1745
1746	MGCS36044_00508			*rihB*	pyrimidine-specific ribonucleoside hydrolase	20.3	1745
1747	MGCS36044_02664			*amyB*	cyclomaltodextrinase protein AmyB	20.3	1745
1748	MGCS36044_01038			-	hypothetical protein	20.0	1748
1749	MGCS36044_01694			*pyrR*	bifunctional pyrimidine regulatory	20.0	1748

No.	Locus tag	Signal6P predicted(1)	Virulence(2)	Gene	Function	RPKMs(3)	RANK(4)
1750	MGCS36044_02070			*dppD*	dipeptide ABC transport system ATP-binding protein DppD	20.0	1748
1751	MGCS36044_02800			-	MdlB family multidrug ABC transporter ATPase and permease	20.0	1748
1752	MGCS36044_02072			*dppC*	dipeptide ABC transport system permease protein	19.8	1752
1753	MGCS36044_00132			*purE*	5-(carboxyamino)imidazole ribonucleotide mutase	19.5	1753
1754	MGCS36044_00648			-	conjugal transfer protein	19.5	1753
1755	MGCS36044_02230			*norG*	PLP-dependent aminotransferase family protein	19.5	1753
1756	MGCS36044_02738			-	hypothetical protein. Region of difference 36044_ROD.7, putative MGE	19.5	1753
1757	MGCS36044_00652			-	conjugal transfer protein	19.3	1757
1758	MGCS36044_02954	Lipo		*metQ*	methionine uptake ABC transporter	19.3	1757
1759	MGCS36044_03338			*drpA*	DNA processing protein (A) DprA	19.3	1757
1760	MGCS36044_02106			-	hypothetical protein	19.0	1760
1761	MGCS36044_03460	Secreted		*aes*	Aes family secreted acetyl esterase/lipase	19.0	1760
1762	MGCS36044_01874			*cadD_1*	CadD family cadmium resistance transporter	18.8	1762
1763	MGCS36044_02178			-	S41 family peptidase	18.8	1762
1764	MGCS36044_00468			-	hypothetical protein	18.5	1764
1765	MGCS36044_00796			*dacA_2*	D-alanyl-D-alanine carboxypeptidase DacA	18.5	1764
1766	MGCS36044_03576			-	rhodanese-like domain-containing protein	18.5	1764
1767	MGCS36044_03604			-	CydC-related ABC transporter ATPase/permease	18.5	1764
1768	MGCS36044_04076			*yybR*	putative HTH-type transcriptional regulator	18.5	1764
1769	MGCS36044_03984			-	MdlB family ABC transporter ATP-binding/permease protein	18.3	1769
1770	MGCS36044_00862			*brnQ_1*	branched-chain amino acid transport system II	17.8	1770
1771	MGCS36044_00354			*ssb_1*	single-stranded DNA-binding protein Ssb	17.5	1771
1772	MGCS36044_03236			*yncA*	YncA family L-amino acid N-acyltransferase	17.5	1771
1773	MGCS36044_04070			*treR*	trehalose operon repressor TreR	17.5	1771
1774	MGCS36044_01416			-	IS3 family transposase	17.3	1774
1775	MGCS36044_02662			*malC*	maltodextrin transport system permease protein MalC	17.3	1774
1776	MGCS36044_03218			*nagC*	NagC family sugar kinase	17.3	1774
1777	MGCS36044_00856			*dapE*	M20/M25/M40 family metallo-hydrolase	17.0	1777
1778	MGCS36044_02074			*dppB*	dipeptide ABC transport system permease protein DppB	17.0	1777
1779	MGCS36044_02576			-	hypothetical protein	17.0	1777
1780	MGCS36044_03606			*htsC*	heme ABC transporter ATP-binding protein HtsC	17.0	1777
1781	MGCS36044_03928			-	Bacteria_small_SRP	17.0	1777
1782	MGCS36044_00338			-	elix-turn-helix transcriptional regulator	16.8	1782
1783	MGCS36044_04026			-	cspA RNA	16.8	1782
1784	MGCS36044_00854	Lipo		-	MetQ/NlpA family ABC transporter substrate-binding lipoprotein	16.5	1784
1785	MGCS36044_01992			*xpt*	xanthine phosphoribosyltransferase Xpt	16.5	1784
1786	MGCS36044_02040	Lipo		-	ABC transporter protein	15.8	1786
1787	MGCS36044_00446			-	GNAT family N-acetyltransferase	15.5	1787
1788	MGCS36044_03994	Secreted	Virulence	*slo*	secreted cholesterol-dependent cytolysin	15.5	1787

No.	Locus tag	Signal6P predicted[(1)]	Virulence[(2)]	Gene	Function	RPKMs[(3)]	RANK[(4)]
1789	MGCS36044_04188			-	NUDIX hydrolase	15.5	1787
1790	MGCS36044_02958			*metN_2*	methionine uptake ABC transporter ATP-binding	15.0	1790
1791	MGCS36044_02992			-	MFS transporter	15.0	1790
1792	MGCS36044_03144			*msrA/B*	Peptide methionine sulfoxide reductase	15.0	1790
1793	MGCS36044_03458			-	IS982 family transposase	15.0	1790
1794	MGCS36044_00642			-	DNA segregation ATPase, conjugal transfer	14.8	1794
1795	MGCS36044_02956			*metP_2*	methionine uptake ABC transporter permease MetP	14.8	1794
1796	MGCS36044_03742			-	hypothetical protein	14.8	1794
1797	MGCS36044_03838			-	cysteine hydrolase	14.8	1794
1798	MGCS36044_01888			-	DUF5960 family protein	14.5	1798
1799	MGCS36044_03702			-	putative sulfite exporter	14.3	1799
1800	MGCS36044_04190			*padR*	PadR family transcriptional regulator	14.0	1800
1801	MGCS36044_00400			*celC_1*	PTS transporter cellobiose-specific IIA	13.8	1801
1802	MGCS36044_02184			*oadA*	oxaloacetate decarboxylase subunit alpha OadA	13.8	1801
1803	MGCS36044_00640			-	conjugal transfer protein	13.5	1803
1804	MGCS36044_01260			-	Unknown	13.5	1803
1805	MGCS36044_02058	Secreted	Virulence	*scpA*	cell surface extracellular C5a peptidase ScpA	13.5	1803
1806	MGCS36044_03066			-	putative secreted protein	13.5	1803
1807	MGCS36044_03992	Secreted		-	TrbC/VirB2-related secreted effector protein	13.5	1803
1808	MGCS36044_00472			-	FtsK/SpoIIIE domain-containing protein	13.3	1808
1809	MGCS36044_01316			-	NAD(P)-binding domain-containing protein	13.3	1808
1810	MGCS36044_00128	Secreted		-	CHAP domain-containing protein	13.0	1810
1811	MGCS36044_01064			-	Spy490380c RNA	13.0	1810
1812	MGCS36044_00644			-	conjugal transfer protein	12.8	1812
1813	MGCS36044_01026	Secreted		*yoaK*	putative secreted YoaK family protein	12.8	1812
1814	MGCS36044_00624			-	helix-turn-helix transcriptional regulator	12.5	1814
1815	MGCS36044_02034	Lipo		-	streptococcal histidine triad (HIT) lipoprotein	12.5	1814
1816	MGCS36044_03876			-	MefA-related MFS transporter	12.5	1814
1817	MGCS36044_01950			*araC*	AraC family transcriptional regulator	12.3	1817
1818	MGCS36044_00434			-	hypothetical protein	12.0	1818
1819	MGCS36044_00474			-	Rep family protein	12.0	1818
1820	MGCS36044_02060			-	unknown	12.0	1818
1821	MGCS36044_02700			*pnuC*	nicotinamide riboside transporter PnuC	12.0	1818
1822	MGCS36044_04158			-	hypothetical protein	12.0	1818
1823	MGCS36044_00464			-	hypothetical protein	11.8	1823
1824	MGCS36044_02744			-	unknown	11.8	1823
1825	MGCS36044_04156			-	FtsK/SpoIIIE domain-containing protein	11.8	1823
1826	MGCS36044_00936			-	FMN RNA	11.5	1826
1827	MGCS36044_02750			-	PEP-utilizing enzyme	11.5	1826
1828	MGCS36044_03146	Lipo		*tlpA*	TlpA family protein disulfide reductase	11.5	1826
1829	MGCS36044_03996			*ifs*	nicotine adenine dinucleotide glycohydrolase	11.5	1826

No.	Locus tag	Signal6P predicted[(1)]	Virulence[(2)]	Gene	Function	RPKMs[(3)]	RANK[(4)]
1830	MGCS36044_00282			-	hypothetical protein	11.3	1830
1831	MGCS36044_00284			-	NUDIX domain-containing protein	11.0	1831
1832	MGCS36044_00688			-	hypothetical protein	11.0	1831
1833	MGCS36044_02922			*coiA*	competence protein CoiA	11.0	1831
1834	MGCS36044_03360	Secreted		*hisJ*	HisJ family amino acid ABC transporter	11.0	1831
1835	MGCS36044_03444			-	IS982 family transposase	11.0	1831
1836	MGCS36044_03962			*ulaF*	L-ribulose-5-phosphate 4-epimerase UlaF	11.0	1831
1837	MGCS36044_00470			-	hypothetical protein	10.8	1837
1838	MGCS36044_03914			-	hypothetical protein	10.8	1837
1839	MGCS36044_00618			-	bacterial mobilization protein	10.5	1839
1840	MGCS36044_00686			-	toxin-antitoxin system, toxin component, Fic	10.5	1839
1841	MGCS36044_02202			*dprA*	DNA-processing protein DprA	10.5	1839
1842	MGCS36044_02748			-	MATE family efflux transporter	10.5	1839
1843	MGCS36044_03998	Secreted	Virulence	*nga*	secreted nicotine adenine dinucleotide	10.5	1839
1844	MGCS36044_04154			-	hypothetical protein	10.5	1839
1845	MGCS36044_00638			-	putative conjugal transfer protein	10.3	1845
1846	MGCS36044_00676			-	XRE family transcriptional regulator	10.3	1845
1847	MGCS36044_04162			-	MerR family transcriptional regulator	10.3	1845
1848	MGCS36044_01116			-	Blp family class II bacteriocin with	10.0	1848
1849	MGCS36044_01692			-	PyrR RNA	10.0	1848
1850	MGCS36044_04160			-	MGCS36044_04160replication protein	10.0	1848
1851	MGCS36044_00268			-	Rep family plasmid replication protein	9.8	1851
1852	MGCS36044_03148			*ccdA*	thiol-disulfide oxidoreductase-associated membrane protein	9.8	1851
1853	MGCS36044_03964			*ulaE*	L-xylulose 5-phosphate 3-epimerase UlaE	9.8	1851
1854	MGCS36044_01152			-	CAAX amino terminal protease family protein	9.5	1854
1855	MGCS36044_01884			-	TIGR01906 family membrane protein	9.5	1854
1856	MGCS36044_00462	Secreted		-	cell surface PgrA surface exclusion	9.3	1856
1857	MGCS36044_00634			-	helix-turn-helix domain-containing protein	9.3	1856
1858	MGCS36044_00678			-	bacteriocin	9.3	1856
1859	MGCS36044_03970			*ulaB*	ascorbate-specific PTS transporter EIIB protein	9.3	1856
1860	MGCS36044_00466			-	hypothetical protein	9.0	1860
1861	MGCS36044_02208			-	MFS transporter	9.0	1860
1862	MGCS36044_00476			-	DUF771 domain-containing protein	8.8	1862
1863	MGCS36044_03966			*ulaD*	3-keto-L-gulonate 6-phosphate decarboxylase	8.8	1862
1864	MGCS36044_00616			-	DUF5962 domain-containing protein	8.5	1864
1865	MGCS36044_00852	Lipo		-	MetQ/NlpA family ABC transporter	8.5	1864
1866	MGCS36044_03500			-	SSRC34_1 RNA	8.5	1864
1867	MGCS36044_03776			*rpsN2*	30S ribosomal S14-2 protein RpsN2	8.5	1864
1868	MGCS36044_00416			-	hypothetical protein	8.3	1868
1869	MGCS36044_00614			-	phage replisome organizer N-terminal	8.3	1868
1870	MGCS36044_02124			-	site-specific integrase	8.3	1868
1871	MGCS36044_03916			-	hypothetical protein	8.3	1868

No.	Locus tag	Signal6P predicted[(1)]	Virulence[(2)]	Gene	Function	RPKMs[(3)]	RANK[(4)]
1872	MGCS36044_00114			*purC*	phosphoribosylaminoimidazolesuccinocarboxamide	8.0	1872
1873	MGCS36044_00620			-	transposase	8.0	1872
1874	MGCS36044_00680			-	lantibiotic dehydratase	8.0	1872
1875	MGCS36044_00722			*comX_1*	competence protein ComX	8.0	1872
1876	MGCS36044_00822			*comX_2*	competence protein ComX	8.0	1872
1877	MGCS36044_02116			-	hypothetical protein	8.0	1872
1878	MGCS36044_02104			-	hypothetical protein	7.8	1878
1879	MGCS36044_04152			-	sigma-70 family RNA polymerase sigma factor	7.8	1878
1880	MGCS36044_00144			-	quorum-sensing system DWW-type pheromone	7.3	1880
1881	MGCS36044_00636	Secreted		-	CHAP domain-containing protein	7.3	1880
1882	MGCS36044_02082			*femX*	FemABX-like family peptidoglycan interpeptide	7.3	1880
1883	MGCS36044_02114			-	hypothetical protein	7.3	1880
1884	MGCS36044_02516			-	tracrRNA RNA	7.3	1880
1885	MGCS36044_03972			*ulaA*	ascorbate-specific PTS transporter EIIC protein	7.3	1880
1886	MGCS36044_00682			-	lanthionine synthetase C family protein	7.0	1886
1887	MGCS36044_00684			-	lantibiotic transporter	7.0	1886
1888	MGCS36044_01148			-	putative bacteriocin with double-glycine leader peptide	7.0	1886
1889	MGCS36044_01886			-	hypothetical protein	7.0	1886
1890	MGCS36044_00674			-	ImmA/IrrE family metallo-endopeptidase	6.8	1890
1891	MGCS36044_01142			-	BlpM-like bacteriocin with double-glycine leader	6.8	1890
1892	MGCS36044_01150			-	hypothetical protein	6.8	1890
1893	MGCS36044_03710			-	Xre family helix-turn-helix transcriptional. Region of difference 36044_ROD.8, putative MGE	6.8	1890
1894	MGCS36044_00142			-	helix-turn-helix domain-containing protein	6.5	1894
1895	MGCS36044_01144			-	lactobin A/cerein 7B family class IIb	6.5	1894
1896	MGCS36044_02092			-	hypothetical protein	6.5	1894
1897	MGCS36044_00270			-	hypothetical protein	6.0	1897
1898	MGCS36044_02064			*htpA*	streptococcal histidine triad (HIT)	6.0	1897
1899	MGCS36044_02752			-	TetR/AcrR family transcriptional regulator	6.0	1897
1900	MGCS36044_03968			*ulaC*	ascorbate-specific PTS transporter EIIA protein	6.0	1897
1901	MGCS36044_03986			-	hypothetical protein	6.0	1897
1902	MGCS36044_01362			-	glycosyltransferase family 2 protein	5.8	1902
1903	MGCS36044_03758			-	replication protein	5.8	1902
1904	MGCS36044_02122			-	DUF3173 family protein	5.5	1904
1905	MGCS36044_03760			-	DUF3173 family protein	5.5	1904
1906	MGCS36044_02094			-	FtsK/SpoIIIE domain-containing protein	5.3	1906
1907	MGCS36044_00272			-	hypothetical protein	5.0	1907
1908	MGCS36044_01146			-	putative bacteriocin with double-glycine leader	5.0	1907
1909	MGCS36044_02118			-	Rep family protein	5.0	1907
1910	MGCS36044_01532			-	preQ1-II RNA	4.8	1910
1911	MGCS36044_00612			-	DNA-binding protein	4.5	1911

No.	Locus tag	Signal6P predicted(1)	Virulence(2)	Gene	Function	RPKMs(3)	RANK(4)
1912	MGCS36044_01356			-	hypothetical protein	4.5	1911
1913	MGCS36044_00322			*comYB*	competence system type II secretion system	4.3	1913
1914	MGCS36044_02062	Lipo	Virulence	*lmb*	bifunctional metal ABC transporter	4.3	1913
1915	MGCS36044_02096			-	hypothetical protein	4.3	1913
1916	MGCS36044_04170			-	parvulin-like peptidyl-prolyl isomerase	4.3	1913
1917	MGCS36044_01110			-	hypothetical protein	4.0	1917
1918	MGCS36044_02742			-	hypothetical protein	4.0	1917
1919	MGCS36044_00698			*gatB_1*	PTS galactitol transporter IIB component GatB	3.8	1919
1920	MGCS36044_00702			-	triose-phosphate isomerase	3.8	1919
1921	MGCS36044_00984			*livF*	branched-chain amino acid ABC transporter ATP-binding protein LivF	3.8	1919
1922	MGCS36044_00986			-	cystathionine beta-synthase (CBS)	3.8	1919
1923	MGCS36044_01040	Secreted		-	putative secreted protein	3.8	1919
1924	MGCS36044_01776	Secreted		-	extracellular cell wall anchored mucin-binding	3.8	1919
1925	MGCS36044_02102			-	hypothetical protein	3.8	1919
1926	MGCS36044_03284			*comFA*	ComFA family DNA/RNA helicase	3.8	1919
1927	MGCS36044_01140		Virulence	*silE*	streptococcal invasion locus pheromone	3.5	1927
1928	MGCS36044_01358			-	hypothetical protein	3.5	1927
1929	MGCS36044_00982			*livG*	branched-chain amino acid ABC transporter ATP-binding protein LivG	3.3	1929
1930	MGCS36044_01318			-	ammonium transporter	3.3	1929
1931	MGCS36044_01946			*maeE*	NAD-dependent malic enzyme MaeE	3.3	1929
1932	MGCS36044_02582			-	hypothetical protein	3.3	1929
1933	MGCS36044_02756			-	relaxase	3.3	1929
1934	MGCS36044_02866	Lipo	Virulence	*slr*	InlA-like streptococcal leucine rich lipoprotein	3.3	1929
1935	MGCS36044_03988			-	hypothetical protein	3.3	1929
1936	MGCS36044_00266			-	DUF3173 domain-containing protein	3.0	1936
1937	MGCS36044_00696			*gatC_1*	PTS galactitol transporter IIC component GatC	3.0	1936
1938	MGCS36044_00980			*livM*	branched-chain amino acid ABC transporter permease LivM	3.0	1936
1939	MGCS36044_01360			-	glycosyltransferase	3.0	1936
1940	MGCS36044_00704			-	YjbQ family protein	2.8	1940
1941	MGCS36044_01156			-	hypothetical protein	2.8	1940
1942	MGCS36044_01774			-	HTH domain-containing putative transcriptional	2.8	1940
1943	MGCS36044_00264			*xerC*	site-specific integrase	2.5	1943
1944	MGCS36044_00690			-	tyrosine-type site-specific	2.5	1943
1945	MGCS36044_00692			-	PRD domain/PTS transporter IIA domain protein	2.5	1943
1946	MGCS36044_01138		Virulence	*silD*	streptococcal invasion locus pheromone secretion	2.5	1943
1947	MGCS36044_01944			*maeP*	citrate/malate symporter protein MaeP	2.5	1943
1948	MGCS36044_02940			*comEC*	DNA internalization-related competence protein	2.5	1943
1949	MGCS36044_03756			-	hypothetical protein	2.5	1943
1950	MGCS36044_00330	Pilin		*comYF*	competence system protein ComYF	2.3	1950
1951	MGCS36044_00976	Lipo		*livK*	branched-chain amino acid ABC transporter	2.3	1950
1952	MGCS36044_00978			*livH*	branched-chain amino acid ABC transporter permease LivH	2.3	1950

No.	Locus tag	Signal6P predicted(1)	Virulence(2)	Gene	Function	RPKMs(3)	RANK(4)
1953	MGCS36044_02754			*mobC*	plasmid mobilization relaxosome protein MobC	2.3	1950
1954	MGCS36044_02942			*comEA*	DNA uptake competence protein ComEA	2.3	1950
1955	MGCS36044_00326	Pilin		*comYD*	competence system type II secretion system GspH	2.0	1955
1956	MGCS36044_00328	Pilin		*comYE*	competence system protein ComYE	1.8	1956
1957	MGCS36044_00332			*comYG*	competence system protein ComYG	1.8	1956
1958	MGCS36044_02764			*topB*	DNA topoisomerase III TopB	1.8	1956
1959	MGCS36044_02816			-	NTPase domain-containing protein	1.8	1956
1960	MGCS36044_02758			-	conjugative element protein	1.5	1960
1961	MGCS36044_02810			-	site-specific serine recombinase family protein	1.5	1960
1962	MGCS36044_02814			-	DUF3801 domain-containing protein	1.5	1960
1963	MGCS36044_00324	Pilin		*comYC*	competence system prepilin-type N-terminal	1.3	1963
1964	MGCS36044_01292	Secreted		-	putative secreted protein	1.3	1963
1965	MGCS36044_02812			*virD4*	type IV secretory system conjugative DNA transfer protein VirD4	1.3	1963
1966	MGCS36044_02746			-	sigma-70 family RNA polymerase sigma factor like	1.0	1966
1967	MGCS36044_02774			-	conjugal transfer protein	1.0	1966
1968	MGCS36044_02120			-	hypothetical protein	0.8	1968
1969	MGCS36044_02760			-	DNA methyltransferase	0.8	1968
1970	MGCS36044_02772			*virB4*	type IV secretory pathway component VirB4	0.8	1968
1971	MGCS36044_02776			*virB6*	VirB6-like conjugal transfer protein	0.8	1968
1972	MGCS36044_02778			-	Maff2 family protein	0.8	1968
1973	MGCS36044_02790			-	phage replisome organizer N-terminal domain-containing protein	0.8	1968
1974	MGCS36044_02818			-	RepA N-terminal domain-containing protein	0.8	1968
1975	MGCS36044_01154			-	hypothetical protein	0.5	1975
1976	MGCS36044_02766	Secreted		-	DUF4366 domain-containing predicted secreted	0.5	1975
1977	MGCS36044_02768			-	conjugal transfer protein	0.5	1975
1978	MGCS36044_02770			-	CHAP domain-containing protein	0.5	1975
1979	MGCS36044_02782			*traG_2*	conjugal transfer protein TraG	0.5	1975
1980	MGCS36044_02784			*tnpW*	TnpW family transposon-encoded protein	0.5	1975
1981	MGCS36044_02796			-	sigma-70 family RNA polymerase sigma factor like protein	0.5	1975
1982	MGCS36044_01134		Virulence	*silCR*	streptococcal invasion locus auto-inducing	0.3	1982
1983	MGCS36044_01136			*silC*	streptococcal invasion locus signaling peptide	0.3	1982
1984	MGCS36044_02780			-	single-stranded DNA-binding protein	0.3	1982
1985	MGCS36044_02794			-	DUF3847 domain-containing protein	0.3	1982
1986	MGCS36044_02792			*mobA*	MobA/MobL family protein	0.0	1986

MGCS36044 was grown *in vitro*, in rich media, and in quadruplicate. Genes were ranked by the mean transcript abundance calculated from four replicates, at early stationary (ES) growth phase (OD=2.0). Genes are ordered by transcript abundance rank, from highest (rank=1) to lowest.

(1) Genes predicted by SignalP 6.0 to have an export signal sequence
(2) Virulence refers to putative virulence genes
(3) RPKMs were assigned by EDGEPro
(4) Rank refers to the corresponding transcript abundance rank based on RPKMs
(5) Lipo, exported lipoprotein attached to the cell envelope

(6) TCS, two-component system

Table S2C. Ranked MGCS36089 genes during growth *in vitro* at mid-exponential phase

No.	Locus tag	Signal6P predicted[(1)]	Virulence[(2)]	Gene	Function	RPKMs[(3)]	RANK[(4)]
1	MGCS36089_03820		Virulence	*gapA*	glyceraldehyde-3-phosphate dehydrogenase GapA	17053.3	1
2	MGCS36089_01342			*tufA*	translation elongation factor Tu protein TufA	16607.8	2
3	MGCS36089_00222			*rplQ*	50S ribosomal L17 protein RplQ	11152.3	3
4	MGCS36089_00224			-	L17DE RNA	10888.0	4
5	MGCS36089_00218			*rpsK*	30S ribosomal S11 protein RpsK	10679.5	5
6	MGCS36089_01794			*rplL*	50S ribosomal L7/L12 protein RplL	10429.5	6
7	MGCS36089_00220			*rpoA*	DNA-directed RNA polymerase subunit alpha RpoA	10401.8	7
8	MGCS36089_01792			*rplJ*	50S ribosomal L10 protein RplJ	10286.0	8
9	MGCS36089_00194			*rpsZ*	type Z 30S ribosomal S14 protein RpsZ	10194.0	9
10	MGCS36089_01796			-	rli38 RNA	10019.3	10
11	MGCS36089_03346			-	RNaseP_bact_b RNA	9921.5	11
12	MGCS36089_00214			*rpmJ*	50S ribosomal L36 protein RpmJ	9336.3	12
13	MGCS36089_00192			*rplE*	50S ribosomal L5 protein RplE	9181.8	13
14	MGCS36089_01496		Virulence	*srrG*	streptolysin S small regulatory RNA SrrG	9151.8	14
15	MGCS36089_01494			*eno*	phosphopyruvate hydratase -- enolase protein	8768.8	15
16	MGCS36089_00178			*rplV*	50S ribosomal L22 protein RplV	8728.5	16
17	MGCS36089_00186			*rpsQ*	30S ribosomal S17 protein RpsQ	8476.5	17
18	MGCS36089_03668			*rpsF*	30S ribosomal S6 protein RpsF	8457.3	18
19	MGCS36089_00216			*rpsM*	30S ribosomal S13 protein RpsM	8400.3	19
20	MGCS36089_00182			*rplP*	50S ribosomal L29 protein RplP	8305.0	20
21	MGCS36089_00174			*rplB*	50S ribosomal L2 protein RplB	8285.3	21
22	MGCS36089_00180			*rpsC*	30S ribosomal S3 protein RpsC	8272.8	22
23	MGCS36089_00190			*rplX*	50S ribosomal L24 protein RplX	8247.0	23
24	MGCS36089_02900			*ptsH*	PTS transporter phosphocarrier protein PtsH	8049.3	24
25	MGCS36089_00204			*rpmD*	50S ribosomal L30 protein RpmD	7969.0	25
26	MGCS36089_00172			*rplW*	50S ribosomal L23 protein RplW	7954.5	26
27	MGCS36089_00754			*fba_2*	fructose-bisphosphate aldolase	7848.0	27
28	MGCS36089_00188			*rplN*	50S ribosomal L14 protein RplN	7844.3	28
29	MGCS36089_00198			*rplF*	50S ribosomal L6 protein RplF	7837.3	29
30	MGCS36089_03826			*rpsL*	30S ribosomal S12 protein RpsL	7757.8	30
31	MGCS36089_00170			*rplD*	50S ribosomal L4 protein RplD	7655.0	31
32	MGCS36089_00176			*rpsS*	30S ribosomal S19 protein RpsS	7578.8	32
33	MGCS36089_03824			*rpsG*	30S ribosomal S7 protein RpsG	7330.5	33
34	MGCS36089_01066			*rplA*	50S ribosomal L1 protein RplA	7170.3	34
35	MGCS36089_00604			*rpsI*	30S ribosomal S9 protein RpsI	7088.5	35
36	MGCS36089_01668			*prp*	ribosomal-processing cysteine protease Prp	7045.5	36
37	MGCS36089_01478			*rplS*	50S ribosomal L19 protein RpsL	7036.5	37
38	MGCS36089_00168			*rplC*	50S ribosomal L3 protein RplC	6783.5	38
39	MGCS36089_00208			*secY*	preprotein translocase subunit SecY	6650.8	39
40	MGCS36089_00196			*rpsH*	30S ribosomal S8 protein RpsH	6489.8	40

No.	Locus tag	Signal6P predicted(1)	Virulence(2)	Gene	Function	RPKMs(3)	RANK(4)
41	MGCS36089_00200			*rplR*	50S ribosomal L18 protein RplR	6349.3	41
42	MGCS36089_00602			*rplM*	50S ribosomal L13 protein RplM	6268.0	42
43	MGCS36089_02382			*rpsA*	30S ribosomal S1 protein RpsA	6200.0	43
44	MGCS36089_01704			-	KH domain-containing protein	6144.0	44
45	MGCS36089_04074			*rpsB*	30S ribosomal S2 protein RpsB	6048.8	45
46	MGCS36089_03666			*ssb_2*	single-stranded DNA-binding protein	5963.5	46
47	MGCS36089_03822			*fusA*	FusA family elongation factor EF-G	5915.0	47
48	MGCS36089_01458			*rpmE*	50S ribosomal L31 type B protein RpmE	5886.3	48
49	MGCS36089_00184			*rpmC*	50S ribosomal L16 protein RpmC	5860.5	49
50	MGCS36089_01064			*rplK*	50S ribosomal L11P protein RplK	5787.0	50
51	MGCS36089_04216			*rpsD*	30S ribosomal S4 protein RpsD	5570.8	51
52	MGCS36089_01638			*rplT*	50S ribosomal L20 protein RplT	5534.0	52
53	MGCS36089_03006			-	DNA-binding protein HU	5528.0	53
54	MGCS36089_02374			*ssrA*	transfer-messenger RNA, SsrA	5494.0	54
55	MGCS36089_00202			*rpsE*	30S ribosomal S5 protein RpsE	5255.3	55
56	MGCS36089_04158			*rpmGA*	50S ribosomal L33 protein RpmGA	5248.0	56
57	MGCS36089_03664			*rpsR*	30S ribosomal S18 protein RpsR	5171.0	57
58	MGCS36089_00104	Secreted		*sibA*	CHAP domain-containing protein/secreted	4996.5	58
59	MGCS36089_00562			*rpsO*	30S ribosomal S15 protein RpsO	4664.3	59
60	MGCS36089_01500		Virulence	-	sagA RNA	4617.8	60
61	MGCS36089_00166			*rpsJ*	30S ribosomal S10 protein RpsJ	4488.3	61
62	MGCS36089_02012			*ldh*	L-lactate dehydrogenase Ldh	4262.5	62
63	MGCS36089_01636			*rpmI*	50S ribosomal L35 protein RpmL	4208.5	63
64	MGCS36089_02360			*pyk*	pyruvate kinase Pyk	4174.0	64
65	MGCS36089_00212			*infA*	translation initiation factor IF-1 protein InfA	4131.3	65
66	MGCS36089_01670			*rpmA*	50S ribosomal L27 protein RpmA	4114.0	66
67	MGCS36089_04076			*tsf*	translation elongation factor Tsf	4076.3	67
68	MGCS36089_03460			*srtB*	pilus polymerization class B sortase SrtB	3973.3	68
69	MGCS36089_02898			*ptsI*	phosphoenolpyruvate--protein phosphotransferase	3937.3	69
70	MGCS36089_03548			*fabT*	transcriptional regulatory protein FabT	3906.8	70
71	MGCS36089_00206			*rplO*	50S ribosomal L15 protein RplO	3814.3	71
72	MGCS36089_02852	Secreted	Virulence	*spg*	extracellular cell surface IgG-binding streptococcal protein (G) SpG. Cell-wall anchoring predicted sortase	3752.0	72
73	MGCS36089_01552			*atpC*	ATP synthase epsilon subunit AtpC	3676.3	73
74	MGCS36089_02280			*rpsT*	30S ribosomal S20 protein RpsT	3675.3	74
75	MGCS36089_03458	Secreted		-	pilus ancillary/minor protein 2	3623.8	75
76	MGCS36089_00364	Secreted		-	secreted pilin backbone/major protein	3578.5	76
77	MGCS36089_02992			*gpmA*	phosphoglycerate mutase GpmA	3473.8	77
78	MGCS36089_01498		Virulence	*sagA*	streptolysin S precursor SagA	3445.3	78
79	MGCS36089_03550			*phaB*	enoyl-CoA hydratase protein PhaB	3333.5	79
80	MGCS36089_01634			*infC*	translation initiation factor InfC	3325.8	80
81	MGCS36089_03464			*sipA*	signal peptidase I SipA	3316.3	81
82	MGCS36089_03518			*manN*	PTS transporter mannose-specific IID component	3309.0	82

No.	Locus tag	Signal6P predicted[(1)]	Virulence[(2)]	Gene	Function	RPKMs[(3)]	RANK[(4)]
83	MGCS36089_01550			*atpD*	ATP synthase beta subunit AtpD	3277.8	83
84	MGCS36089_03462	Secreted		-	pilus backbone/major protein	3263.5	84
85	MGCS36089_00482			*ssrS*	6S RNA	3248.3	85
86	MGCS36089_01542			*atpF*	ATP synthase B subunit AtpF	3179.3	86
87	MGCS36089_01546			*atpA*	ATP synthase alpha chain, AtpA	3129.3	87
88	MGCS36089_00516	Secreted	Virulence	*emm*	cell surface M protein Emm	3092.5	88
89	MGCS36089_01666			*rplU*	50S ribosomal L21 protein RplU	3072.0	89
90	MGCS36089_01544			*atpH*	ATP synthase delta subunit AtpH	3001.5	90
91	MGCS36089_01548			*atpG*	ATP synthase gamma subunit AtpG	2980.8	91
92	MGCS36089_02362			*pfkA*	6-phosphofructokinase PfkA	2955.3	92
93	MGCS36089_01702			*rpsP*	30S ribosomal S16 protein RpsP	2951.5	93
94	MGCS36089_03546			*fabH*	3-oxoacyl-[acyl-carrier-protein] synthase	2862.5	94
95	MGCS36089_00736			*tig*	trigger factor molecular chaperone Tig	2802.3	95
96	MGCS36089_03816			*pgk*	phosphoglycerate kinase Pgk	2695.5	96
97	MGCS36089_01540			*atpB*	ATP synthase A subunit AtpB	2679.5	97
98	MGCS36089_03514			*manL*	PTS transporter mannose-specific IIB & IIA	2668.0	98
99	MGCS36089_03870			*rpmH*	50S ribosomal L34 protein RpmH	2656.3	99
100	MGCS36089_03516			*manM*	PTS transporter mannose-specific IIC component	2573.3	100
101	MGCS36089_03466	Secreted		-	pilus ancillary/minor protein 1	2482.5	101
102	MGCS36089_04118			*spxA_2*	transcriptional regulator SpxA	2302.3	102
103	MGCS36089_01352			*tpiA*	triose-phosphate isomerase TpiA	2235.8	103
104	MGCS36089_02920	Lipo[(5)]		*prsA*	peptidylprolyl isomerase lipoprotein PrsA	2221.5	104
105	MGCS36089_01586			*rpsU*	30S ribosomal S21 protein RpsU	2196.5	105
106	MGCS36089_03628	Secreted	Virulence	*isp2*	Isp-related CHAP domain-containing immunogenic	2133.3	106
107	MGCS36089_03530			*accC*	acetyl-CoA carboxylase biotin carboxylase	2128.0	107
108	MGCS36089_00316			*rpoC*	DNA-directed RNA polymerase subunit beta' RpoC	2123.0	108
109	MGCS36089_02682			*dltC*	D-alanine--poly(phosphoribitol) ligase subunit	2113.3	109
110	MGCS36089_01716			*apbA*	2-dehydropantoate 2-reductase	2111.3	110
111	MGCS36089_03544			*acpP_2*	acyl carrier protein AcpP	2094.8	111
112	MGCS36089_04052			*ahpF*	alkyl hydroperoxide reductase F subunit AhpF	2064.0	112
113	MGCS36089_03534			*accB*	acetyl-CoA carboxylase biotin carboxyl carrier	2025.8	113
114	MGCS36089_03060			-	RNA-binding protein	2009.0	114
115	MGCS36089_02684			*dltB*	D-alanyl-lipoteichoic acid biosynthesis protein	1963.0	115
116	MGCS36089_03532			*fabZ*	3-hydroxyacyl-ACP dehydratase FabZ	1918.5	116
117	MGCS36089_03528			*accA*	acetyl-CoA carboxylase, carboxyltransferase beta	1867.5	117
118	MGCS36089_03062			-	YggT family protein	1798.0	118
119	MGCS36089_01052	Lipo		*mtsA*	metal ABC transporter substrate-binding	1785.5	119
120	MGCS36089_02680			*dltD*	D-alanyl-lipoteichoic acid biosynthesis protein	1772.5	120
121	MGCS36089_04246			-	transglycosylase SLT domain-containing protein	1753.5	121
122	MGCS36089_03526			*accD*	acetyl-CoA carboxylase carboxyl transferase	1752.8	122
123	MGCS36089_03716			*glpF_2*	glycerol uptake facilitator GlpF	1746.8	123
124	MGCS36089_03058			*divIVA*	cell division protein DivIVA	1731.0	124

No.	Locus tag	Signal6P predicted[(1)]	Virulence[(2)]	Gene	Function	RPKMs[(3)]	RANK[(4)]
125	MGCS36089_03652			*efp*	translation elongation factor (P) Efp	1726.0	125
126	MGCS36089_03066			*yggS*	YggS family pyridoxal phosphate-dependent	1704.5	126
127	MGCS36089_02008			*fadH2*	FadH2 superfamily FAD-dependent oxidoreductase	1700.5	127
128	MGCS36089_03650			-	Asp23/Gls24 family envelope stress response	1685.5	128
129	MGCS36089_02686			*dltA*	D-alanine--poly(phosphoribitol) ligase subunit	1685.0	129
130	MGCS36089_02336			*yeaQ*	GlsB/YeaQ/YmgE family stress response membrane	1671.5	130
131	MGCS36089_03694			*pflB*	formate C-acetyltransferase	1661.5	131
132	MGCS36089_03932			*pgi*	Pgi family glucose-6-phosphate isomerase	1645.3	132
133	MGCS36089_03536			*fabF*	3-oxoacyl-[acyl-carrier-protein] synthase	1627.3	133
134	MGCS36089_03090			*dps*	DNA-binding starvation protein	1589.3	134
135	MGCS36089_02332			-	DUF2273 domain-containing protein	1578.8	135
136	MGCS36089_03538			*fabG_2*	3-ketoacyl-(acyl-carrier-protein) reductase	1562.0	136
137	MGCS36089_03068			*ftsZ*	cell division protein FtsZ	1560.0	137
138	MGCS36089_03540			*fabD*	malonyl CoA-acyl carrier protein transacylase	1543.0	138
139	MGCS36089_02326			-	Asp23/Gls24 family envelope stress response	1537.5	139
140	MGCS36089_03064			*sepF*	cell division protein SepF	1536.5	140
141	MGCS36089_03348			*gpsB*	cell division regulator GpsB	1505.0	141
142	MGCS36089_02330			-	Asp23/Gls24 family envelope stress response	1483.0	142
143	MGCS36089_03556			*dnaK*	molecular chaperone DnaK	1459.0	143
144	MGCS36089_01516		Virulence	*sagI*	streptolysin S export permease protein SagI	1452.3	144
145	MGCS36089_03648			*nusB*	transcription termination protein NusB	1446.3	145
146	MGCS36089_04050			*ahpC*	alkyl hydroperoxide reductase C subunit AhpC	1440.5	146
147	MGCS36089_02896			*gapN*	NADP-dependent glyceraldehyde-3-phosphate	1438.0	147
148	MGCS36089_00762			-	DAK2 domain-containing protein	1426.5	148
149	MGCS36089_04156			*rpmF*	50S ribosomal L32 protein RpmF	1423.5	149
150	MGCS36089_03828	Secreted		*prgA*	surface exclusion domain-containing secreted	1378.0	150
151	MGCS36089_03326			*rny*	ribonuclease (Y) Rny	1376.8	151
152	MGCS36089_01714			-	PTS transporter subunit IIC	1363.8	152
153	MGCS36089_02334			*amaP*	alkaline shock response membrane anchor protein	1360.3	153
154	MGCS36089_01798			-	hypothetical protein	1353.0	154
155	MGCS36089_03476			*infB*	translation initiation factor IF-2	1319.8	155
156	MGCS36089_01502		Virulence	*sagB*	streptolysin S biosynthesis protein SagB	1317.8	156
157	MGCS36089_01514		Virulence	*sagH*	streptolysin S export permease protein SagH	1311.8	157
158	MGCS36089_03542			*fabK*	enoyl-[acyl-carrier-protein] reductase protein	1308.0	158
159	MGCS36089_02328			-	CsbD family protein	1289.8	159
160	MGCS36089_00860			*htpX*	zinc metalloprotease HtpX	1268.8	160
161	MGCS36089_03810			*glnA*	glutamine synthetase GlnA	1265.0	161
162	MGCS36089_00366	Secreted		-	secreted pilin minor/ancillary protein	1250.0	162
163	MGCS36089_00030			*ftsH*	ATP-dependent zinc metalloprotease FtsH	1247.0	163
164	MGCS36089_03356			*pepC*	aminopeptidase (A) PepC	1240.8	164
165	MGCS36089_04288		Virulence	*htrA*	trypsin-like serine protease HtrA	1234.0	165
166	MGCS36089_04238			*mnmA*	tRNA 2-thiouridine(34) synthase MnmA	1228.0	166

No.	Locus tag	Signal6P predicted[(1)]	Virulence[(2)]	Gene	Function	RPKMs[(3)]	RANK[(4)]
167	MGCS36089_00106			*prs*	ribose-phosphate pyrophosphokinase PrsA	1225.3	167
168	MGCS36089_03634			*secA*	preprotein translocase subunit SecA	1218.3	168
169	MGCS36089_03082			*typA*	translational GTPase TypA	1217.3	169
170	MGCS36089_01588			*mscL*	large-conductance mechanosensitive channel	1215.5	170
171	MGCS36089_01508		Virulence	*sagE*	streptolysin S self-immunity protein SagE	1213.0	171
172	MGCS36089_04112			*ruvX*	Holliday junction resolvase RuvX	1207.8	172
173	MGCS36089_01512		Virulence	*sagG*	streptolysin S export protein SagG	1206.0	173
174	MGCS36089_03812			*glnR*	glutamine synthetase transcriptional repressor	1196.5	174
175	MGCS36089_02964			*deaD*	DEAD/DEAH box helicase	1196.3	175
176	MGCS36089_04236			*marC*	MarC family small neutral amino acid	1183.8	176
177	MGCS36089_01080	Secreted		*lysM*	LysM peptidoglycan-binding domain-containing	1182.3	177
178	MGCS36089_01510		Virulence	*sagF*	streptolysin S biosynthesis protein SagF	1179.5	178
179	MGCS36089_04268			*guaB*	IMP dehydrogenase GuaB	1179.0	179
180	MGCS36089_00378		Virulence	*fbp*	secreted fibronectin-binding protein. SignalP-6 predicted standard secretion signal	1177.5	180
181	MGCS36089_02610			*rfbC*	dTDP-4-dehydrorhamnose 3,5-epimerase RfbC	1174.0	181
182	MGCS36089_02608			*rfbB*	dTDP-glucose 4,6-dehydratase RfbB	1173.5	182
183	MGCS36089_01504		Virulence	*sagC*	streptolysin S biosynthesis protein SagC	1166.5	183
184	MGCS36089_00858			*lemA*	LemA family protein	1161.8	184
185	MGCS36089_00952			*upp*	uracil phosphoribosyltransferase Upp	1154.3	185
186	MGCS36089_03312			*pknB*	Stk1 family PASTA domain-containing Ser/Thr	1137.3	186
187	MGCS36089_02612			*rfbA*	glucose-1-phosphate thymidylyltransferase RfbA	1121.0	187
188	MGCS36089_01506		Virulence	*sagD*	streptolysin S biosynthesis protein SagD	1103.5	188
189	MGCS36089_04244			-	HAD hydrolase-like protein	1102.3	189
190	MGCS36089_00864		Virulence	*covR*	TCS[(6)] DNA-binding response regulator CovR	1093.8	190
191	MGCS36089_00368			*srtC_1*	class C sortase SrtC	1091.0	191
192	MGCS36089_03300			*cysK*	cysteine synthase A CysK	1080.5	192
193	MGCS36089_01418			*ftsX*	cell division permease-like protein FtsX	1075.3	193
194	MGCS36089_02688			*dltX*	teichoic acid D-Ala incorporation-associated	1069.8	194
195	MGCS36089_04082			*treB*	PTS transporter trehalose-specific EIIBC	1069.3	195
196	MGCS36089_00896			*yccA*	YccA family protein	1062.3	196
197	MGCS36089_01722			*fruA*	fructose-specific PTS transporter EIIC component	1059.5	197
198	MGCS36089_03818	Lipo		*lppC*	e(P4) family 5'-nucleotidase lipoprotein	1058.8	198
199	MGCS36089_01072			*frr*	ribosome recycling factor Frr	1054.8	199
200	MGCS36089_00376			*srtC_3*	class C sortase SrtC	1054.5	200
201	MGCS36089_03322			*rpoZ*	DNA-directed RNA polymerase omega subunit RpoZ	1054.0	201
202	MGCS36089_02694			*glnQ_2*	glutamine transport ATP-binding protein GlnQ	1050.0	202
203	MGCS36089_03426			*degV_2*	DegV family fatty acid-binding protein	1048.5	203
204	MGCS36089_02028	Secreted		-	DUF1002 domain-containing putative secreted	1042.0	204
205	MGCS36089_01308			*lysS*	lysyl-tRNA synthetase LysS	1030.8	205
206	MGCS36089_03324			*gmk*	guanylate kinase Gmk	1030.0	206
207	MGCS36089_02428			*xapA*	XapA family purine-nucleoside phosphorylase	1027.5	207
208	MGCS36089_02430			*arsC_2*	arsenate reductase ArsC	1026.3	208

No.	Locus tag	Signal6P predicted(1)	Virulence(2)	Gene	Function	RPKMs(3)	RANK(4)
209	MGCS36089_02542			*glmM*	phosphoglucosamine mutase GlmM	1026.0	209
210	MGCS36089_00210			*adk*	adenylate kinase protein Adk	1021.3	210
211	MGCS36089_02426			*deoD*	DeoD-type purine-nucleoside phosphorylase	1013.0	211
212	MGCS36089_02356			*glmS*	glutamine--fructose-6-phosphate transaminase	1007.3	212
213	MGCS36089_02286			*pepN*	lysyl aminopeptidase/alanine aminopeptidase	1001.5	213
214	MGCS36089_00756			*rpmB*	50S ribosomal L28 protein RpmB	992.5	214
215	MGCS36089_00308			*tyrS*	tyrosyl-tRNA synthetase TyrS	987.8	215
216	MGCS36089_03572			*gatB_2*	aspartyl-tRNA(Asn) or glutamyl-tRNA(Gln)	987.0	216
217	MGCS36089_02626			*apt*	adenine phosphoribosyltransferase Apt	984.0	217
218	MGCS36089_02208			-	apolipoprotein A1/A4/E family protein	983.0	218
219	MGCS36089_02432			*deoB*	phosphopentomutase DeoB	964.8	219
220	MGCS36089_02270			*cdd*	cytidine deaminase Cdd	957.3	220
221	MGCS36089_00794	Lipo		*oppA_1*	oligopeptide ABC transporter substrate-binding protein	953.0	221
222	MGCS36089_02262			-	putative nucleoside ABC transporter permease	943.3	222
223	MGCS36089_03368			-	GlnQ family polar amino acid ABC transporter	942.0	223
224	MGCS36089_03952			*tgt*	tRNA guanosine(34) transglycosylase Tgt	942.0	223
225	MGCS36089_02268	Lipo		-	putative nucleoside ABC transporter	941.3	225
226	MGCS36089_03070			*ftsA*	cell division protein FtsA	940.8	226
227	MGCS36089_03886			*gltX*	glutamate--tRNA ligase	938.3	227
228	MGCS36089_01538			*atpE*	ATP synthase C subunit AtpE	937.8	228
229	MGCS36089_04242			*sdhA*	L-serine dehydratase alpha subunit SdhA	937.0	229
230	MGCS36089_00370			*srtC_2*	class C sortase SrtC	933.3	230
231	MGCS36089_00800			*oppD_1*	oligopeptide ABC transporter permease protein	925.3	231
232	MGCS36089_03674			*trxA_2*	thioredoxin TrxA	923.3	232
233	MGCS36089_00372	Secreted		-	secreted pilin minor/ancillary protein	912.8	233
234	MGCS36089_00802			*oppF_1*	oligopeptide ABC transporter ATP-binding protein	910.5	234
235	MGCS36089_01488			*ezrA*	cell division septation ring formation regulator	902.8	235
236	MGCS36089_03482			*nusA*	transcription termination factor NusA	902.3	236
237	MGCS36089_02120			*guaA*	glutamine-hydrolyzing GMP synthase	902.0	237
238	MGCS36089_00390			*purA*	adenylosuccinate synthase PurA	896.3	238
239	MGCS36089_01698			-	TVP38 superfamily protein	881.3	239
240	MGCS36089_02264			-	putative nucleoside ABC transporter permease	881.3	239
241	MGCS36089_04114			-	IreB-related regulatory phosphoprotein	878.3	241
242	MGCS36089_03342			*mapZ*	MapZ family cell division site-positioning	877.8	242
243	MGCS36089_03798			*rnjA_2*	mRNA degradation ribonuclease RnjA	871.0	243
244	MGCS36089_03574			*gatA_2*	aspartyl-tRNA(Asn) or glutamyl-tRNA(Gln)	869.3	244
245	MGCS36089_00886			*greA*	transcription elongation factor GreA	858.8	245
246	MGCS36089_01416			*ftsE*	cell division ATP-binding protein FtsE	857.8	246
247	MGCS36089_03912			*galU*	UTP--glucose-1-phosphate uridylyltransferase	856.3	247
248	MGCS36089_03558			*grpE*	heat shock protein/nucleotide exchange factor	853.8	248
249	MGCS36089_00314			*rpoB*	DNA-directed RNA polymerase subunit beta RpoB	850.3	249
250	MGCS36089_00760			-	Asp23/Gls24 family envelope stress response	844.5	250

No.	Locus tag	Signal6P predicted[(1)]	Virulence[(2)]	Gene	Function	RPKMs[(3)]	RANK[(4)]
251	MGCS36089_00954			*clpP*	ATP-dependent Clp protease proteolytic subunit	842.8	251
252	MGCS36089_02288			*phoU_2*	phosphate signaling complex protein PhoU	841.3	252
253	MGCS36089_00576			*cysE*	serine O-acetyltransferase CysE	834.5	253
254	MGCS36089_03302			-	S1 RNA-binding domain-containing protein	826.8	254
255	MGCS36089_02916			*alaS*	alanine--tRNA synthetase AlaS	825.5	255
256	MGCS36089_02730			-	DUF1846 domain-containing protein	823.5	256
257	MGCS36089_00888	Lipo		*yidC_1*	membrane protein insertase lipoprotein YidC	821.8	257
258	MGCS36089_00936			*ppaC*	manganese-dependent inorganic pyrophosphatase	819.0	258
259	MGCS36089_01286			-	PspC domain-containing protein	817.0	259
260	MGCS36089_01648			-	YlbF/YmcA family competence regulator	816.0	260
261	MGCS36089_01966			*eutD*	phosphate acetyltransferase EutD	815.3	261
262	MGCS36089_03394			*tkt*	transketolase Tkt	814.5	262
263	MGCS36089_02290			*ptsB1*	phosphate ABC transporter ATP-binding protein	814.3	263
264	MGCS36089_04274			*uup*	Uup family ATPase components of ABC transporters	813.5	264
265	MGCS36089_02210			-	hypothetical protein	811.8	265
266	MGCS36089_03474			*rbfA*	30S ribosome-binding factor RbfA	811.0	266
267	MGCS36089_03314			*pppL*	Stp1/IreP family PP2C-type Ser/Thr phosphatase	810.8	267
268	MGCS36089_04110			-	DUF1292 domain-containing protein	807.0	268
269	MGCS36089_02558			*acoL*	dihydrolipoyl dehydrogenase AcoL	806.5	269
270	MGCS36089_01696			-	ABC transporter permease	805.3	270
271	MGCS36089_03478			-	YlxQ-related RNA-binding protein	800.5	271
272	MGCS36089_03490			*cotS*	CotS family thiamine kinase	796.5	272
273	MGCS36089_01782			*engB*	ribosome biogenesis GTP-binding protein EngB	788.8	273
274	MGCS36089_01426			*asnC*	asparaginyl-tRNA synthetase protein AsnC	784.0	274
275	MGCS36089_03956		Virulence	*perR*	peroxide-responsive transcriptional repressor	779.0	275
276	MGCS36089_02720			*pepS*	aminopeptidase PepS	776.3	276
277	MGCS36089_03776			*ybaB*	YbaB family DNA-binding protein	771.3	277
278	MGCS36089_03986			*leuS*	leucine--tRNA synthase LeuS	768.3	278
279	MGCS36089_03488			*trmB*	tRNA (guanosine(46)-N7)-methyltransferase TrmB	764.0	279
280	MGCS36089_03560			*hrcA*	heat-inducible transcriptional repressor HrcA	763.0	280
281	MGCS36089_03596			*alaT*	AlaT family aminotransferase	755.3	281
282	MGCS36089_02306			*spxA_1*	transcriptional regulator SpxA	751.5	282
283	MGCS36089_01720			*fruK*	1-phosphofructokinase FruK	746.8	283
284	MGCS36089_00312			-	Lacto-rpoB RNA	746.5	284
285	MGCS36089_02272			*deoC*	deoxyribose-phosphate aldolase DeoC	743.3	285
286	MGCS36089_02292			*ptsB2*	phosphate ABC transporter ATP-binding protein	720.0	286
287	MGCS36089_03552			*dnaJ*	chaperone protein DnaJ	719.3	287
288	MGCS36089_04224			*rplI*	50S ribosomal L9 protein RplI	719.0	288
289	MGCS36089_02562			*acoC*	dihydrolipoamide acetyltransferase AcoC	716.5	289
290	MGCS36089_00884			*mltG*	endolytic transglycosylase MltG	715.8	290
291	MGCS36089_01188			*thrS*	threonyl-tRNA synthetase ThrS	713.8	291
292	MGCS36089_00028			-	hypoxanthine-guanine phosphoribosyltransferase	711.0	292

No.	Locus tag	Signal6P predicted[(1)]	Virulence[(2)]	Gene	Function	RPKMs[(3)]	RANK[(4)]
293	MGCS36089_03480			-	YlxR family putative RNA-binding protein	709.8	293
294	MGCS36089_00574			-	polynucleotide phosphorylase/polyadenylase	709.5	294
295	MGCS36089_04290			*parB*	chromosome partitioning protein ParB	707.0	295
296	MGCS36089_01522			*queT*	Queuosine precursor transporter QueT	705.0	296
297	MGCS36089_02948			*sodA*	superoxide dismutase SodA	705.0	296
298	MGCS36089_01296			-	DUF3270 domain-containing protein	704.0	298
299	MGCS36089_02564			*acoB*	pyruvate dehydrogenase E1 component beta subunit	703.8	299
300	MGCS36089_01070			*pyrH*	UMP kinase PyrH	703.3	300
301	MGCS36089_03594			*codY*	CodY family GTP-sensing pleiotropic	703.0	301
302	MGCS36089_00336			*ackA*	acetate kinase AckA	699.3	302
303	MGCS36089_01726	Lipo		-	putative peptidoglycan hydrolase lipoprotein	696.5	303
304	MGCS36089_02266			-	putative nucleoside ABC transporter ATP-binding	692.8	304
305	MGCS36089_04142			*argS*	arginine--tRNA synthase ArgS	691.5	305
306	MGCS36089_03554			-	PfpI family predicted protease/amidase	687.8	306
307	MGCS36089_04122			*recA*	recombinase RecA	685.3	307
308	MGCS36089_00738			*rpoE*	DNA-directed RNA polymerase subunit delta RpoE	684.8	308
309	MGCS36089_04104			*nrdD_2*	anaerobic ribonucleoside-triphosphate reductase	684.8	308
310	MGCS36089_02212			-	CsbD family protein	683.0	310
311	MGCS36089_01094			-	Nudix superfamily phosphohydrolase	681.3	311
312	MGCS36089_03830			*purR*	pur operon repressor PurR	679.3	312
313	MGCS36089_03872			*jag*	RNA-binding protein Jag	676.5	313
314	MGCS36089_03370			*hisM*	HisM family amino acid ABC transporter permease	669.0	314
315	MGCS36089_00572			*pnp*	polyribonucleotide nucleotidyltransferase Pnp	667.5	315
316	MGCS36089_01362			*murM*	peptidoglycan lipid II--L-alanine ligase protein	664.3	316
317	MGCS36089_03408	Lipo		-	PepSY domain-containing lipoprotein	663.5	317
318	MGCS36089_04222			*dnaC*	replicative DNA helicase DnaC	663.0	318
319	MGCS36089_03964			*proW*	proline/glycine betaine ABC transporter ATPase	660.3	319
320	MGCS36089_01694			-	LolD superfamily ABC transporter ATPase	660.0	320
321	MGCS36089_00546			*proS*	prolyl-tRNA synthetase ProS	659.5	321
322	MGCS36089_00834			-	YebC/PmpR family DNA-binding transcriptional	658.8	322
323	MGCS36089_01360			*murN*	peptidoglycan lipid II-Ala--L-alanine ligase	657.0	323
324	MGCS36089_03046			-	DUF1797 family protein	657.0	323
325	MGCS36089_01364			-	sugar-phosphatase	655.5	325
326	MGCS36089_02904			*nrdE_2*	class 1b ribonucleoside-diphosphate reductase	651.5	326
327	MGCS36089_03000			*zntA*	ZntA family P-type heavy metal transporter	651.5	326
328	MGCS36089_00816			*nadD*	nicotinate-nucleotide adenylyltransferase NadD	650.0	328
329	MGCS36089_02918			-	LURP-one-related family protein	647.8	329
330	MGCS36089_03500			-	Cps2a family anionic cell wall polymer	647.0	330
331	MGCS36089_02206			-	LCB5 family diacylglycerol lipid kinase	646.5	331
332	MGCS36089_03056			*ileS*	isoleucine--tRNA synthetase IleS	642.8	332
333	MGCS36089_00778			*rgpG*	undecaprenyl/decaprenyl-phosphate alpha-N-acetylglucosaminyl 1-phosphate transferase RgpG	640.8	333
334	MGCS36089_04078			*pepO*	endopeptidase PepO	640.0	334

No.	Locus tag	Signal6P predicted(1)	Virulence(2)	Gene	Function	RPKMs(3)	RANK(4)
335	MGCS36089_02692	Lipo		*glnP_2*	glutamine-binding protein/glutamine transport	637.3	335
336	MGCS36089_01186			-	glycosyltransferase	636.5	336
337	MGCS36089_01294			-	YtxH domain-containing protein	636.0	337
338	MGCS36089_03354			*pbp1A*	bifunctional PG transglycosylase-transpeptidase	636.0	337
339	MGCS36089_00798			*oppC_1*	oligopeptide ABC transporter permease protein	634.3	339
340	MGCS36089_03412			*glyS*	glycine--tRNA ligase beta subunit GlyS	634.0	340
341	MGCS36089_01260			-	SPJ_0845 family protein	630.8	341
342	MGCS36089_02560			-	hypothetical protein	629.5	342
343	MGCS36089_02894			-	PgdA-like putative PG GlcNAc deacetylase	628.5	343
344	MGCS36089_00874			*der*	ribosome biogenesis GTPase Der	624.3	344
345	MGCS36089_01778			-	hypothetical protein	619.0	345
346	MGCS36089_02974			*prfC*	peptide chain release factor 3 PrfC	617.8	346
347	MGCS36089_02294			*ptsA*	phosphate ABC transporter permease PstA	616.5	347
348	MGCS36089_02624			*dnaD*	DNA replication protein DnaD	616.3	348
349	MGCS36089_04256			*rodZ*	cytoskeletal protein RodZ	616.0	349
350	MGCS36089_01244			*ftsY*	signal recognition particle-docking protein	615.8	350
351	MGCS36089_01732			*degV_1*	DegV family protein	613.8	351
352	MGCS36089_02438			*pepV*	dipeptidase PepV	612.3	352
353	MGCS36089_01014			*metS*	methionine--tRNA synthase MetS	609.3	353
354	MGCS36089_02434			*rpiA*	ribose-5-phosphate isomerase RpiA	607.3	354
355	MGCS36089_02906			*nrdF_2*	class 1b ribonucleoside-diphosphate reductase	606.8	355
356	MGCS36089_01414			*prfB*	peptide chain release factor 2 PrfB	606.3	356
357	MGCS36089_04252			*cbiO1*	cobalt ABC transporter ATPase CbiO2	603.3	357
358	MGCS36089_01054			*mtsB*	metal ABC transporter ATP-binding protein MtsB	602.8	358
359	MGCS36089_02258			*panT*	pantothenic acid transporter PanT	601.5	359
360	MGCS36089_03662			-	DUF1129 domain-containing protein	596.0	360
361	MGCS36089_03484			*rimP*	ribosome maturation factor RimP	595.5	361
362	MGCS36089_02370			-	ABC transporter permease	593.8	362
363	MGCS36089_01592			*rpoD*	RNA polymerase sigma factor RpoD	592.5	363
364	MGCS36089_01216			*glnP_1*	glutamine ABC transporter permease GlnP	591.3	364
365	MGCS36089_01092			*era*	GTPase Era	585.0	365
366	MGCS36089_03180			*valS*	valine--tRNA synthetase ValS	583.8	366
367	MGCS36089_01372			*mgtA*	MgtA superfamily cation-translocating P-type	582.0	367
368	MGCS36089_01632			-	L20_leader RNA	581.5	368
369	MGCS36089_00880			*murC*	UDP-N-acetylmuramate--L-alanine ligase MurC	579.5	369
370	MGCS36089_03052			-	DUF1827 family protein	579.5	369
371	MGCS36089_01556			*murA_1*	UDP-N-acetylglucosamine 1-carboxyvinyltransferase protein MurA	578.8	371
372	MGCS36089_01780			*clpX*	ATP-dependent Clp protease, ATP-binding subunit	576.8	372
373	MGCS36089_03304			-	putative bifunctional	576.3	373
374	MGCS36089_00538			*yajC*	preprotein translocase subunit YajC	575.5	374
375	MGCS36089_03524			*serS*	seryl-tRNA synthetase SerS	572.5	375
376	MGCS36089_00796			*oppB_1*	oligopeptide ABC transporter permease protein	572.0	376

No.	Locus tag	Signal6P predicted(1)	Virulence(2)	Gene	Function	RPKMs(3)	RANK(4)
377	MGCS36089_00820			-	cysteine hydrolase	568.3	377
378	MGCS36089_00866		Virulence	*covS*	TCS sensor kinase CovS	567.8	378
379	MGCS36089_01718			*fruR*	fructose operon transcriptional repressor	567.8	378
380	MGCS36089_04254			*pgsA*	CDP-diacylglycerol--glycerol-3-phosphate	567.5	380
381	MGCS36089_02622			*nth*	endonuclease III Nth	565.5	381
382	MGCS36089_01922			*potB*	spermidine putrescine ABC transport system	565.3	382
383	MGCS36089_01226		Virulence	*vicK*	TCS signal transduction sensor kinase VicK	561.8	383
384	MGCS36089_02998			-	Spy1186876 RNA	561.8	383
385	MGCS36089_03910			*gpsA*	NAD(P)H-dependent glycerol-3-phosphate	561.3	385
386	MGCS36089_01692			-	RND family transporter membrane fusion protein	561.0	386
387	MGCS36089_00024	Secreted		-	class A beta-lactamase-related serine hydrolase	560.0	387
388	MGCS36089_01218			-	glutamine ABC transporter permease	560.0	387
389	MGCS36089_01090			*dgkA*	diacylglycerol kinase DgkA	559.8	389
390	MGCS36089_04014			*nusG*	transcription antitermination protein NusG	559.8	389
391	MGCS36089_00818			*yqeK*	bis(5'-nucleosyl)-tetraphosphatase (symmetrical)	558.0	391
392	MGCS36089_03264			-	DUF402 domain-containing protein	555.8	392
393	MGCS36089_01594			-	metal-sulfur cluster assembly factor	555.5	393
394	MGCS36089_01220	Secreted		-	glutamine ABC transporter substrate-binding	554.8	394
395	MGCS36089_03388			-	hypothetical protein	554.8	394
396	MGCS36089_03782			-	DUF536 domain-containing protein	554.5	396
397	MGCS36089_03382			*mraW*	S-adenosyl-methyltransferase MraW	554.0	397
398	MGCS36089_03878		Virulence	*fasX*	FasBCAX signal transduction system small RNA	551.3	398
399	MGCS36089_03374			*cshB*	DEAD/DEAH box helicase	550.0	399
400	MGCS36089_02550			-	CobQ-like type 1 glutamine amidotransferase	549.3	400
401	MGCS36089_01920			*potA*	spermidine putrescine ABC transport system	548.0	401
402	MGCS36089_04036			*clpC*	ATP-dependent Clp protease ATP-binding subunit	547.0	402
403	MGCS36089_02846			*map*	methionyl aminopeptidase Map	546.8	403
404	MGCS36089_03364			*trxB_2*	thioredoxin-disulfide reductase TrxB	546.3	404
405	MGCS36089_03008			-	DUF2140 domain-containing protein	546.0	405
406	MGCS36089_00740			*pyrG*	CTP synthase Pyg	542.5	406
407	MGCS36089_01954			*nifS_2*	NifS superfamily cysteine desulfurase	542.0	407
408	MGCS36089_01284			-	SprT family protein	541.8	408
409	MGCS36089_00004			*dnaN*	DNA polymerase III subunit beta protein DnaN	541.3	409
410	MGCS36089_02296			*ptsC*	phosphate ABC transporter permease PstC	541.3	409
411	MGCS36089_02298	Lipo		*ptsS*	phosphate ABC transporter substrate-binding	540.5	411
412	MGCS36089_02976			-	TIGR02206 family membrane protein	540.3	412
413	MGCS36089_01640			*ltaS*	LTA synthase LtaS	538.5	413
414	MGCS36089_01424			*aspC*	aspartate aminotransferase protein AspC	537.8	414
415	MGCS36089_00894			*rnaY*	RnaY family HD domain-containing protein	536.5	415
416	MGCS36089_01088			*ybeY*	rRNA maturation RNase YbeY	534.5	416
417	MGCS36089_03100			*rlmN*	23S rRNA (adenine(2503)-C(2))-methyltransferase	533.3	417
418	MGCS36089_04032			*groEL*	chaperonin GroEL	533.0	418

No.	Locus tag	Signal6P predicted[(1)]	Virulence[(2)]	Gene	Function	RPKMs[(3)]	RANK[(4)]
419	MGCS36089_01300			-	PrtC family collagenase-like protease	532.5	419
420	MGCS36089_04266			*glcU*	glucose uptake permease GlcU	530.5	420
421	MGCS36089_01956			*ribP*	ribose-phosphate pyrophosphokinase RibP	530.0	421
422	MGCS36089_00356			-	deoxyadenosine kinase	529.3	422
423	MGCS36089_03292			*raiA*	ribosome-associated translation inhibitor RaiA	528.5	423
424	MGCS36089_00898			*yneF*	YneF family protein	525.5	424
425	MGCS36089_00582			*mrnC*	mini-ribonuclease 3 MrnC	525.0	425
426	MGCS36089_02774			-	helix-turn-helix domain-containing putative	524.8	426
427	MGCS36089_00556			*def*	peptide deformylase Def	524.0	427
428	MGCS36089_00554			-	NAD(P)H-dependent oxidoreductase	523.0	428
429	MGCS36089_01926			*potD*	spermidine putrescine ABC transport system	522.8	429
430	MGCS36089_01290			*lgt*	prolipoprotein diacylglyceryl transferase Lgt	522.0	430
431	MGCS36089_03358			*nadE*	ammonia-dependent NAD(+) synthetase NadE	522.0	430
432	MGCS36089_01056			*mtsC*	metal ABC transporter permease MtsC	518.0	432
433	MGCS36089_02388			-	DUF2969 domain-containing protein	516.0	433
434	MGCS36089_01558			*epuA*	DNA-directed RNA polymerase beta subunit EpuA	514.5	434
435	MGCS36089_03376			*mraY*	phospho-N-acetylmuramoyl-pentapeptide-	513.3	435
436	MGCS36089_04240			*sdhB*	L-serine dehydratase beta subunit SdhB	511.5	436
437	MGCS36089_01628			-	LysM peptidoglycan-binding domain-containing	511.3	437
438	MGCS36089_02256			*coaC*	phosphopantothenoylcysteine decarboxylase CoaC	511.3	437
439	MGCS36089_02844			*brkB*	BrkB family protein	511.0	439
440	MGCS36089_02016			*srtA*	class A sortase SrtA	510.8	440
441	MGCS36089_00358			*dus*	tRNA-dihydrouridine synthase Dus	509.8	441
442	MGCS36089_03072			*ftsQ*	cell division protein FtsQ/DivIB	509.8	441
443	MGCS36089_03360			*pncB*	nicotinate phosphoribosyltransferase PncB	509.8	441
444	MGCS36089_02962			*kup*	potassium uptake protein Kup	508.8	444
445	MGCS36089_02890			*udk*	uridine kinase Udk	508.5	445
446	MGCS36089_04120			-	hypothetical protein	508.5	445
447	MGCS36089_03502			-	GNAT family N-acetyltransferase	506.8	447
448	MGCS36089_02876			*metK*	methionine adenosyltransferase MetK	505.3	448
449	MGCS36089_02368			-	ABC transporter ATP-binding protein	504.8	449
450	MGCS36089_01626			*ebsA*	EbsA family pore-forming protein	504.3	450
451	MGCS36089_02392			*parC*	DNA topoisomerase IV subunit A ParC	504.0	451
452	MGCS36089_01924			*potC*	spermidine putrescine ABC transport system	498.8	452
453	MGCS36089_02128			*ffh*	signal recognition particle protein	498.0	453
454	MGCS36089_03012			-	DegV family EDD domain-containing protein	498.0	453
455	MGCS36089_03564			-	LD/DD carboxypeptidase family protein	497.0	455
456	MGCS36089_02224			*nrdD_1*	ATP cone domain-containing protein,	496.0	456
457	MGCS36089_01608			*rgpF*	alpha-L-Rha alpha-1,3-L-rhamnosyltransferase	495.8	457
458	MGCS36089_02518			-	CRISPR-DR22 RNA	495.3	458
459	MGCS36089_00906			-	CBS domain-containing protein	494.3	459
460	MGCS36089_01472			*fldA*	flavodoxin FldA	493.8	460

No.	Locus tag	Signal6P predicted[(1)]	Virulence[(2)]	Gene	Function	RPKMs[(3)]	RANK[(4)]
461	MGCS36089_02204		Virulence	*yqfA*	membrane channel forming/hemolysin III protein	491.5	461
462	MGCS36089_02192			*topA*	type I DNA topoisomerase TopA	489.5	462
463	MGCS36089_02566			*acoA*	Pyruvate dehydrogenase E1 component alpha	489.0	463
464	MGCS36089_01620			*galE*	UDP-glucose 4-epimerase GalE	488.0	464
465	MGCS36089_03010			-	SGNH-hydrolase superfamily of lipases and	488.0	464
466	MGCS36089_03378			*pbp2X*	PG transpeptidase class B penicillin-binding	487.5	466
467	MGCS36089_01074			*cvfB*	S1 RNA-binding domain-containing protein CvfB	487.0	467
468	MGCS36089_01044			*macP*	cell wall synthase accessory phosphoprotein	484.5	468
469	MGCS36089_01042			*nudF*	NUDIX hydrolase NudF	483.8	469
470	MGCS36089_00776			*mecA*	negative regulator of genetic competence,	480.3	470
471	MGCS36089_03712		Virulence	*cppA*	CppA family putative C3-glycoprotein degrading	479.5	471
472	MGCS36089_01180		Virulence	*ccpA*	catabolite control protein CcpA	478.3	472
473	MGCS36089_00310			*pbp1b*	bifunctional PG transglycosylase-transpeptidase,	477.0	473
474	MGCS36089_00912			*scp2*	segregation/condensation complex subunit (B)	476.5	474
475	MGCS36089_04152			*aspS*	aspartyl-tRNA synthetase	475.3	475
476	MGCS36089_02520			-	CRISPR-DR22 RNA	473.5	476
477	MGCS36089_01610			-	glycosyltransferase family 2 protein	473.3	477
478	MGCS36089_02132			-	FAD-binding oxidoreductase	472.0	478
479	MGCS36089_03576			*gatC_2*	aspartyl-tRNA(Asn) or glutamyl-tRNA(Gln)	469.3	479
480	MGCS36089_00878			-	hypothetical protein	468.8	480
481	MGCS36089_02282		Virulence	*ciaH*	TCS sensor histidine kinase protein CiaH	468.5	481
482	MGCS36089_00916			*yidD*	membrane protein insertion efficiency factor	468.0	482
483	MGCS36089_00836	Lipo		-	amino acid ABC transporter substrate-binding	467.5	483
484	MGCS36089_02574			*rnjA_1*	mRNA degradation ribonuclease RnjA	467.0	484
485	MGCS36089_00032			*plaP*	amino acid permease PlaP	465.5	485
486	MGCS36089_00580			*cysS*	cysteine--tRNA synthetase CysS	464.8	486
487	MGCS36089_03966			*proV*	proline/glycine betaine ABC transporter permease	464.8	486
488	MGCS36089_01918			*murB*	UDP-N-acetylmuramate dehydrogenase MurB	464.3	488
489	MGCS36089_00544			*rseP*	RIP metalloprotease RseP	464.0	489
490	MGCS36089_03186			-	VOC family protein	463.0	490
491	MGCS36089_01288			*hprK*	HPr(Ser) kinase/phosphatase HprK	462.0	491
492	MGCS36089_00822			*rsfS*	ribosome silencing factor RsfS	461.3	492
493	MGCS36089_02552			*lplA_2*	lipoate--protein ligase LplA	460.5	493
494	MGCS36089_03958			*yccU*	YccU family CoA-binding protein	460.0	494
495	MGCS36089_00768			-	ABC amino acid transporter ATP-binding protein	459.5	495
496	MGCS36089_03562			-	FlgJ-related putative peptidoglycan hydrolase	458.3	496
497	MGCS36089_03084			*pspE*	PspE family rhodanese-like domain-containing	455.3	497
498	MGCS36089_00112			*acpP_1*	acyl carrier protein AcpP	455.0	498
499	MGCS36089_01624			*pepT*	peptidase (T) PepT	454.0	499
500	MGCS36089_00908			*xerD_1*	site-specific tyrosine recombinase XerD	453.0	500
501	MGCS36089_02980			*ddl*	D-alanine--D-alanine ligase Ddl	452.8	501
502	MGCS36089_01596			*rmlD*	dTDP-4-dehydrorhamnose reductase protein RmlD	449.3	502

No.	Locus tag	Signal6P predicted[(1)]	Virulence[(2)]	Gene	Function	RPKMs[(3)]	RANK[(4)]
503	MGCS36089_02506			-	CRISPR-DR22 RNA	449.0	503
504	MGCS36089_00770	Secreted		-	ABC amino acid transporter substrate-binding protein	448.3	504
505	MGCS36089_01086			-	uracil DNA glycosylase superfamily protein	448.3	504
506	MGCS36089_00578			-	hypothetical protein	445.8	506
507	MGCS36089_00982			*rsmI*	16S rRNA (cytidine(1402)-2'-O)-methyltransferase	445.3	507
508	MGCS36089_01224		Virulence	*vicR*	TCS DNA-binding response regulator VicR	445.3	507
509	MGCS36089_01994			-	Sua5/YciO/YrdC/YwlC family protein ribosome	445.3	507
510	MGCS36089_03874	Lipo		*yidC_2*	YidC/Oxa1 family membrane protein insertase	445.3	507
511	MGCS36089_01046			*mtnN*	5'-methylthioadenosine/adenosylhomocysteine	443.5	511
512	MGCS36089_01644			*aroD*	type I 3-dehydroquinate dehydratase AroD	442.5	512
513	MGCS36089_01616			-	glycosyltransferase family 1 protein	442.0	513
514	MGCS36089_01604			*rgpD*	ABC transporter polysaccharide/polyol phosphate	441.5	514
515	MGCS36089_01606			*rgpE*	glycosyltransferase family GT2 protein RgpE	441.3	515
516	MGCS36089_01336			*ftsW*	cell division protein FtsW	441.0	516
517	MGCS36089_03520			-	DUF956 family protein	440.8	517
518	MGCS36089_01564			*pheT*	phenylalanyl-tRNA synthetase beta subunit PheT	440.0	518
519	MGCS36089_01996			-	GNAT family N-acetyltransferase	439.8	519
520	MGCS36089_01486			*gyrB*	DNA topoisomerase ATP-hydrolyzing B subunit	439.3	520
521	MGCS36089_03390			*ccmA*	CcmA family multidrug ABC transporter ATPase	439.3	520
522	MGCS36089_00750			-	alpha/beta hydrolase	437.8	522
523	MGCS36089_00872			*dnaI*	primosomal protein DnaI	437.8	522
524	MGCS36089_02508			-	CRISPR-DR22 RNA	437.8	522
525	MGCS36089_04226			-	DHH family phosphoesterase	437.0	525
526	MGCS36089_03188			-	helix-hairpin-helix domain-containing protein	436.3	526
527	MGCS36089_00550			*polC*	DNA polymerase III PolC	432.3	527
528	MGCS36089_00904			-	YfcE family metallophosphoesterase	431.3	528
529	MGCS36089_01952			-	DUF1831 domain-containing protein	431.3	528
530	MGCS36089_01184			-	glycosyltransferase	430.8	530
531	MGCS36089_03960			-	hypothetical protein	430.0	531
532	MGCS36089_01630			*cmk*	CMP kinase Cmk	429.5	532
533	MGCS36089_00814			*yhbY*	ribosome assembly RNA-binding protein YhbY	426.8	533
534	MGCS36089_02486			*lepA*	translation elongation factor 4 LepA	426.5	534
535	MGCS36089_02716			*obgE*	GTPase ObgE	425.8	535
536	MGCS36089_03074			*murG*	UDP-N-acetylglucosamine--N-acetylmuramyl-(pentapeptide) pyrophosphoryl-undecaprenol N-acetylglucosamine transferase MurG	424.5	536
537	MGCS36089_00910			*scp1*	segregation/condensation complex subunit (A)	424.3	537
538	MGCS36089_04116			-	SSRC41 RNA	423.8	538
539	MGCS36089_01614			-	DUF2142 domain-containing protein	423.5	539
540	MGCS36089_02424			-	LytR family transcriptional regulator	422.3	540
541	MGCS36089_00302			*adcR*	zinc-dependent MarR family transcriptional	422.0	541
542	MGCS36089_00306			*adcB*	metal ABC transporter permease AdcB	421.5	542
543	MGCS36089_01174			*gloA*	lactoylglutathione lyase protein GloA	419.3	543

No.	Locus tag	Signal6P predicted[(1)]	Virulence[(2)]	Gene	Function	RPKMs[(3)]	RANK[(4)]
544	MGCS36089_04080			*treC*	trehalose-6-phosphate hydrolase TreC	419.0	544
545	MGCS36089_00360			*hslO*	Hsp33 family molecular chaperone HslO	418.8	545
546	MGCS36089_02440			*nfnB*	NfnB family nitroreductase	418.8	545
547	MGCS36089_01292			-	COG4768 superfamily YoxC-like protein	418.3	547
548	MGCS36089_03660			-	CorA family divalent cation transport protein	418.3	547
549	MGCS36089_01048		Virulence	*mtsR*	metal-dependent transcriptional regulator MtsR	415.3	549
550	MGCS36089_01222			*glnQ_1*	glutamine ABC transporter ATPase GlnQ	415.0	550
551	MGCS36089_01658			*thiI*	thiamine biosynthesis/tRNA modification protein	415.0	550
552	MGCS36089_04106			-	DUF2079 domain-containing protein	414.5	552
553	MGCS36089_03876			*rnpA*	ribonuclease P protein component RnpA	412.8	553
554	MGCS36089_02014			*gyrA*	DNA gyrase subunit A GyrA	412.5	554
555	MGCS36089_01612			-	DUF2304 domain-containing protein	411.3	555
556	MGCS36089_00020			*divIC*	septum formation initiator family protein	410.3	556
557	MGCS36089_02504			-	CRISPR-DR22 RNA	409.8	557
558	MGCS36089_03148			-	B3/4 domain-containing protein	409.8	557
559	MGCS36089_03344			*rlmL*	23S rRNA N2-methylase RlmL	409.5	559
560	MGCS36089_03380			*ftsL*	cell division protein FtsL	408.8	560
561	MGCS36089_01034			-	ASCH domain-containing RNA-binding protein	408.5	561
562	MGCS36089_00522			*dtd*	D-tyrosyl-tRNA deacylase Dtd	408.3	562
563	MGCS36089_04034			*groES*	co-chaperone GroES	407.5	563
564	MGCS36089_03968			-	diacylglycerol kinase family lipid kinase	407.0	564
565	MGCS36089_01990			*prfA*	peptide chain release factor 1 PrfA	406.8	565
566	MGCS36089_02390			*bcaT*	branched-chain amino acid aminotransferase	406.5	566
567	MGCS36089_00592			-	DegV family protein	406.3	567
568	MGCS36089_04018			*pbp2A*	multimodular transpeptidase-transglycosylase	406.3	567
569	MGCS36089_00764			-	SPFH domain-containing protein	406.0	569
570	MGCS36089_04220			-	Veg family protein	405.5	570
571	MGCS36089_02394			-	hypothetical protein	404.0	571
572	MGCS36089_02502			-	CRISPR-DR22 RNA	403.5	572
573	MGCS36089_03098			-	VanZ like family glycopeptide antibiotic	403.5	572
574	MGCS36089_03414			*glyQ*	glycine--tRNA ligase alpha subunit GlyQ	403.3	574
575	MGCS36089_00590			-	NYN domain-containing protein	402.8	575
576	MGCS36089_00110			*plsX*	phosphate acyltransferase PlsX	401.5	576
577	MGCS36089_02010			-	FolA superfamily dihydrofolate reductase	400.5	577
578	MGCS36089_00384			-	PTS sugar transporter subunit IIC	398.5	578
579	MGCS36089_00938			-	cytoplasmic protein	398.3	579
580	MGCS36089_03680			-	colicin V production family protein	398.0	580
581	MGCS36089_00882			-	GNAT family N-acetyltransferase	397.8	581
582	MGCS36089_01058	Lipo		-	peptidylprolyl isomerase lipoprotein	397.3	582
583	MGCS36089_02972			-	Pleckstrin homology-like domain-containing	397.0	583
584	MGCS36089_03632			*acpS*	AcpS family provisional 4'-phosphopantetheinyl	397.0	583
585	MGCS36089_00010			*engD*	redox-regulated ATPase EngD	396.5	585

No.	Locus tag	Signal6P predicted[(1)]	Virulence[(2)]	Gene	Function	RPKMs[(3)]	RANK[(4)]
586	MGCS36089_00504			-	M42 family metallopeptidase	395.8	586
587	MGCS36089_02598			-	tetra tricopeptide repeat family protein	395.5	587
588	MGCS36089_02978			*murF*	UDP-N-acetylmuramoyl-tripeptide--D-alanyl-D-	395.5	587
589	MGCS36089_00900			*murI*	glutamate racemase MurI	394.3	589
590	MGCS36089_04154			*hisS*	histidine--tRNA synthase HisS	394.3	589
591	MGCS36089_00862			*yceD*	large ribosomal RNA subunit accumulation protein	392.5	591
592	MGCS36089_00870			*dnaB*	replication initiation and membrane attachment	392.3	592
593	MGCS36089_03102			*yutD*	YutD family protein of unknown function	391.5	593
594	MGCS36089_00812			*yqeH*	ribosome biogenesis GTPase YqeH	391.3	594
595	MGCS36089_01776			*dyr*	dihydrofolate reductase Dyr	391.3	594
596	MGCS36089_03182			-	AAA family ATPase	391.0	596
597	MGCS36089_03566			-	PhoE family broad specificity phosphatase	391.0	596
598	MGCS36089_00304			*adcC*	metal ABC transporter ATP-binding protein AdcC	390.5	598
599	MGCS36089_04230			*mnmG*	tRNA uridine-5-carboxymethylaminomethyl(34)	389.8	599
600	MGCS36089_01622			-	RfbX superfamily lipopolysaccharide biosynthesis	389.3	600
601	MGCS36089_04150			-	YitT family protein putative ABC transporter	389.3	600
602	MGCS36089_00852			*ktrA*	potassium uptake transporter gating subunit	389.0	602
603	MGCS36089_01618			-	LTA synthase family protein	388.8	603
604	MGCS36089_03498			-	chemotaxis protein	387.8	604
605	MGCS36089_03690			-	hypothetical protein	387.3	605
606	MGCS36089_00788			*sufB*	Fe-S cluster assembly protein SufB	387.0	606
607	MGCS36089_02498			-	CRISPR-DR22 RNA	386.5	607
608	MGCS36089_04198			-	DUF368 domain-containing protein	386.0	608
609	MGCS36089_00022			-	hypothetical protein	385.8	609
610	MGCS36089_00542			*cdsA*	phosphatidate cytidylyltransferase CdsA	384.3	610
611	MGCS36089_03190			-	DUF1912 family protein	383.8	611
612	MGCS36089_01312			-	PhoE superfamily phosphatase	383.3	612
613	MGCS36089_02000			-	nucleoid-associated bacterial family protein	383.3	612
614	MGCS36089_03838			*rpe*	ribulose-phosphate 3-epimerase Rpe	383.3	612
615	MGCS36089_01590			*dnaG*	DNA primase protein DnaG	381.5	615
616	MGCS36089_02340			-	Glycine RNA	381.5	615
617	MGCS36089_00152			*oatA*	acetyltransferase OatA	379.8	617
618	MGCS36089_02630			*dltE*	short-chain dehydrogenase DltE	379.0	618
619	MGCS36089_03202			*aroB*	3-dehydroquinate synthase protein AroB	378.3	619
620	MGCS36089_01950			-	DUF4649 domain-containing protein	377.8	620
621	MGCS36089_03962			*polA*	DNA polymerase I PolA	375.5	621
622	MGCS36089_01760			-	isopentenyl-diphosphate delta-isomerase	374.3	622
623	MGCS36089_03630			*alr*	alanine racemase Alr	374.3	622
624	MGCS36089_04100			*mviM*	MviM family predicted dehydrogenase	373.8	624
625	MGCS36089_00514		Virulence	*mga*	M protein trans-acting positive regulator Mga	373.0	625
626	MGCS36089_02882			*dnaX*	DNA polymerase III gamma/tau subunit DnaX	372.3	626
627	MGCS36089_03758			-	type I restriction-modification system (M)	371.3	627

No.	Locus tag	Signal6P predicted[(1)]	Virulence[(2)]	Gene	Function	RPKMs[(3)]	RANK[(4)]
628	MGCS36089_02996			*pyrD*	dihydroorotate dehydrogenase PyrD	370.8	628
629	MGCS36089_03492			*ecsB*	ABC exoprotein transporter permease EcsB	370.5	629
630	MGCS36089_02516			-	CRISPR-DR22 RNA	369.0	630
631	MGCS36089_02874			*murA_2*	UDP-N-acetylglucosamine	369.0	630
632	MGCS36089_00984			-	DUF5684 domain-containing protein	368.3	632
633	MGCS36089_01752			*sptS*	SptS-like TCS sensor histidine kinase	367.3	633
634	MGCS36089_02548			*murE_2*	UDP-N-acetylmuramoylalanyl-D-glutamate-2,	367.0	634
635	MGCS36089_01178			*pepQ*	Xaa-Pro dipeptidase protein PepQ	366.8	635
636	MGCS36089_02884			-	GAF domain containing protein	365.5	636
637	MGCS36089_00932		Virulence	*hlyX*	hemolysin family protein HylX	365.3	637
638	MGCS36089_03504			*tsaE*	tRNA(adenosine(37)-N6)-threonylcarbamoyltransferase TsaE	365.3	637
639	MGCS36089_01642			*rlmK*	23S rRNA methyltransferase RmlK	364.8	639
640	MGCS36089_03352			*recU*	Holliday junction resolvase RecU	364.5	640
641	MGCS36089_01526			*dagK*	diacylglycerol kinase family lipid kinase	364.0	641
642	MGCS36089_03946			-	metallo-beta-lactamase superfamily protein	361.8	642
643	MGCS36089_01992			*prmC*	peptide chain release factor N(5)-glutamine	361.3	643
644	MGCS36089_02324			-	GntR family transcriptional regulator	361.3	643
645	MGCS36089_04128			*tag*	DNA-3-methyladenine glycosylase Tag	360.8	645
646	MGCS36089_01314			*yccF*	YccF domain-containing protein	360.0	646
647	MGCS36089_02118			-	SpF66_s RNA	359.0	647
648	MGCS36089_00552			-	MarR family transcriptional regulator	358.3	648
649	MGCS36089_01036			-	Gfo/Idh/MocA family oxidoreductase	358.3	648
650	MGCS36089_01578			*rexA*	ATP-dependent nuclease A subunit RexA	357.5	650
651	MGCS36089_04148			-	YitT family protein putative ABC transporter	356.8	651
652	MGCS36089_01528			*pulA_2*	type I pullulanase PulA	356.3	652
653	MGCS36089_01282			*tex*	RNA-binding transcriptional accessory protein	355.5	653
654	MGCS36089_00980			*yabA*	DNA replication initiation control protein YabA	355.0	654
655	MGCS36089_03430			-	Cof-type HAD-IIB family phosphohydrolase	355.0	654
656	MGCS36089_01412			*queG*	epoxyqueuosine reductase QueG	354.5	656
657	MGCS36089_01998			*glyA*	serine hydroxymethyl transferase GlyA	354.0	657
658	MGCS36089_02284		Virulence	*ciaR*	TCS DNA-binding response regulator protein CiaR	353.0	658
659	MGCS36089_01916			*folK*	2-amino-4-hydroxy-6-hydroxymethyldihydropteridine pyrophosphokinase protein FolK	352.8	659
660	MGCS36089_00560			-	major facilitator transporter family protein	352.5	660
661	MGCS36089_02348	Lipo		-	amino acid ABC transporter substrate-binding	352.3	661
662	MGCS36089_01676			*rluD*	ribosomal large subunit pseudouridine synthase	351.8	662
663	MGCS36089_02944			-	DUF3114 domain-containing protein	351.0	663
664	MGCS36089_02632			*rnz*	ribonuclease Rnz	350.5	664
665	MGCS36089_00300			*ipk*	4-diphosphocytidyl-2-C-methyl-D-erythritol	349.8	665
666	MGCS36089_01316			-	aminoacyl-tRNA deacylase	349.8	665
667	MGCS36089_01040			*glmU*	bifunctional UDP-N-acetylglucosamine diphosphorylase/glucosamine-1-phosphate N-acetyltransferase GlmU	349.0	667
668	MGCS36089_03756			-	type I restriction-modification system	349.0	667

No.	Locus tag	Signal6P predicted[(1)]	Virulence[(2)]	Gene	Function	RPKMs[(3)]	RANK[(4)]
669	MGCS36089_01166			-	cell division protein FtsW-like protein	348.0	669
670	MGCS36089_04146			-	YitT family protein putative ABC transporter	347.8	670
671	MGCS36089_01176			-	NAD(P)H-dependent oxidoreductase	347.5	671
672	MGCS36089_01030			*fabG_1*	3-oxoacyl-ACP reductase FabG	347.0	672
673	MGCS36089_03894			-	AIM24 family protein	345.5	673
674	MGCS36089_02932			*pepF_2*	oligoendopeptidase (F) PepF	344.8	674
675	MGCS36089_01700			-	glycerophosphodiester phosphodiesterase	344.3	675
676	MGCS36089_03676			-	FAD-containing oxidoreductase	343.8	676
677	MGCS36089_00352			-	DUF4479 and tRNA-binding domain-containing	343.5	677
678	MGCS36089_03832			*cbf*	YhaM family 3'-5' exoribonuclease	343.0	678
679	MGCS36089_00784			*sufS*	cysteine desulfurase SufS	342.8	679
680	MGCS36089_01602			*rgpC*	ABC transporter polysaccharide/polyol phosphate	342.5	680
681	MGCS36089_03684			*rnhC*	HIII ribonuclease RnhC	341.8	681
682	MGCS36089_01434			*whiA*	cell division involved DNA-binding protein WhiA	341.5	682
683	MGCS36089_04024			*rluA_2*	RluA family pseudouridine synthase	341.5	682
684	MGCS36089_02510			-	CRISPR-DR22 RNA	339.8	684
685	MGCS36089_01032			-	DUF3977 family protein	338.8	685
686	MGCS36089_02572			*frmB*	FrmB family esterase	338.8	685
687	MGCS36089_02002			-	lysozyme family protein	338.0	687
688	MGCS36089_00786			*sufE*	SUF system NifU family Fe-S cluster assembly	337.8	688
689	MGCS36089_03410			-	DUF896 family protein	337.5	689
690	MGCS36089_04126			-	VOC family protein	337.5	689
691	MGCS36089_01770			*mvaS1*	hydroxymethylglutaryl-CoA reductase protein (1)	337.3	691
692	MGCS36089_04218			-	helix-turn-helix domain-containing	336.5	692
693	MGCS36089_01310			-	HAD family hydrolase	336.3	693
694	MGCS36089_03494			*ecsA*	ABC exoprotein transporter ATPase EcsA	336.0	694
695	MGCS36089_03086			*glcK*	glucokinase GlcK	335.8	695
696	MGCS36089_00392	Lipo		-	BMP family ABC transporter substrate-binding	335.5	696
697	MGCS36089_03682			-	hypothetical protein	334.8	697
698	MGCS36089_01228			*vicX*	zinc-dependent hydrolase protein VicX	334.5	698
699	MGCS36089_01462			*nrnA*	bifunctional oligoribonuclease/PAP phosphatase	334.3	699
700	MGCS36089_01948			*rex*	redox-sensing transcriptional repressor Rex	333.8	700
701	MGCS36089_02396			*parE*	DNA topoisomerase IV subunit B ParE	333.8	700
702	MGCS36089_00502			-	Udk family kinase	333.0	702
703	MGCS36089_01914			*folQ*	dihydroneopterin aldolase protein FolB	333.0	702
704	MGCS36089_00914			*rluB*	ribosomal large subunit pseudouridine synthase	332.8	704
705	MGCS36089_02514			-	CRISPR-DR22 RNA	332.3	705
706	MGCS36089_00140			*purB*	adenylosuccinate lyase PurB	332.0	706
707	MGCS36089_03808	Lipo		*oppA_2*	oligopeptide ABC transporter substrate-binding	332.0	706
708	MGCS36089_01256			-	DUF1980 domain-containing protein	330.8	708
709	MGCS36089_01964			*rluA_1*	RluA family pseudouridine synthase	330.5	709
710	MGCS36089_00850			*sstT*	serine/threonine transporter SstT	330.3	710

No.	Locus tag	Signal6P predicted[(1)]	Virulence[(2)]	Gene	Function	RPKMs[(3)]	RANK[(4)]
711	MGCS36089_03184			-	GNAT family N-acetyltransferase	330.0	711
712	MGCS36089_00902			*rdgB*	RdgB family non-canonical purine NTP	329.8	712
713	MGCS36089_02178			*gid*	tRNA (uracil-5-)-methyltransferase/glucose	328.8	713
714	MGCS36089_03080			-	DUF3165 family protein	328.5	714
715	MGCS36089_02260			*pgmA*	phospho-sugar mutase PgmA	328.3	715
716	MGCS36089_01170			*wcaA*	WcaA superfamily glycosyltransferase involved in	328.0	716
717	MGCS36089_01258			-	permease	327.8	717
718	MGCS36089_03840			*rsgA*	ribosome small subunit-dependent GTPase (A)	327.5	718
719	MGCS36089_01160			*secG*	preprotein translocase subunit SecG	326.5	719
720	MGCS36089_02198			*ylqF*	ribosome biogenesis GTPase YlqF	325.5	720
721	MGCS36089_03266			-	DUF960 domain-containing protein	325.5	720
722	MGCS36089_00588			*rmlB*	23S rRNA (guanosine(2251)-2'-O)-methyltransferase RlmB	325.3	722
723	MGCS36089_02512			-	CRISPR-DR22 RNA	324.8	723
724	MGCS36089_03686			*lepB_2*	signal peptidase I LepB	324.5	724
725	MGCS36089_04016			*secE*	preprotein translocase subunit protein SecE	324.5	724
726	MGCS36089_01242			-	Cof-type HAD-IIB family hydrolase	323.8	726
727	MGCS36089_03638			*manA*	mannose-6-phosphate isomerase ManA	323.8	726
728	MGCS36089_00350			*trxA_1*	thioredoxin family protein TrxA-like protein	323.5	728
729	MGCS36089_02662	Lipo		*malE*	maltose/maltodextrin ABC transport system	323.5	728
730	MGCS36089_01730			-	DUF1149 domain-containing protein	322.8	730
731	MGCS36089_01912			*folP*	dihydropteroate synthase protein FolP	322.8	730
732	MGCS36089_03658			*uvrA*	excinuclease ABC subunit (A) UvrA	321.3	732
733	MGCS36089_00002			*dnaA*	chromosomal replication initiator protein DnaA	318.8	733
734	MGCS36089_03416			*ypbQ*	YpbQ family isoprenylcysteine carboxyl	318.8	733
735	MGCS36089_02600			*perM*	PerM family predicted purR regulated permease	318.0	735
736	MGCS36089_01736			-	Uup family ATPase components of ABC transporters	317.8	736
737	MGCS36089_01910			*folE*	GTP cyclohydrolase I protein FolE	317.3	737
738	MGCS36089_02196			*rnhB*	HII ribonuclease RnhB	316.8	738
739	MGCS36089_01758			*mvaK2*	mevalonate kinase MvaK2	316.5	739
740	MGCS36089_00528			*msmK*	sn-glycerol-3-phosphate ABC transporter	314.5	740
741	MGCS36089_03366			-	DUF4059 family protein	314.5	740
742	MGCS36089_04132			*mdtH*	MdtH-related MFS multidrug resistance	314.3	742
743	MGCS36089_02350			-	amino acid ABC transporter ATP-binding protein	314.0	743
744	MGCS36089_02338			*pcrA*	DNA helicase PcrA	313.0	744
745	MGCS36089_01060			*ftsK*	cell division protein FtsK	312.3	745
746	MGCS36089_02636			*hflX*	GTP-binding protein HflX	310.5	746
747	MGCS36089_00346			*pepA*	glutamyl aminopeptidase PepA	310.3	747
748	MGCS36089_03032			-	phospho-sugar mutase	310.0	748
749	MGCS36089_03834			*rmuC*	DNA recombination protein RmuC	309.8	749
750	MGCS36089_00026			*tilS*	tRNA lysidine(34) synthetase TilS	309.5	750
751	MGCS36089_00994			*arsC_1*	arsenate reductase ArsC	309.5	750
752	MGCS36089_03316			*rsmB*	16S rRNA (cytosine(967)-C(5))-methyltransferase	307.8	752

No.	Locus tag	Signal6P predicted[(1)]	Virulence[(2)]	Gene	Function	RPKMs[(3)]	RANK[(4)]
753	MGCS36089_03372	Secreted		*hisJ*	HisJ family amino acid ABC transporter	307.8	752
754	MGCS36089_02690			*uvrB*	excinuclease ABC subunit UvrB	307.5	754
755	MGCS36089_02436			*mnmE*	MnmE family tRNA	307.3	755
756	MGCS36089_01908			*folC*	dihydrofolate synthase FolC	307.0	756
757	MGCS36089_01332			*pepF_1*	oligoendopeptidase PepF	306.8	757
758	MGCS36089_01156			-	DUF853 domain-containing protein	306.0	758
759	MGCS36089_01774			*thyA*	thymidylate synthase ThyA	305.8	759
760	MGCS36089_00782			*sufD*	Fe-S cluster assembly protein SufD	304.0	760
761	MGCS36089_03384			*proA*	glutamate-5-semialdehyde dehydrogenase ProA	303.5	761
762	MGCS36089_01524			*ligA*	NAD-dependent DNA ligase LigA	302.8	762
763	MGCS36089_00150			-	hypothetical protein	302.3	763
764	MGCS36089_02154	Lipo		-	FMN-binding protein, major membrane immunogen	301.5	764
765	MGCS36089_02544	Lipo		*ybbR*	YbbR family lipoprotein	301.0	765
766	MGCS36089_00846			*metP_1*	methionine ABC transporter permease MetP	300.3	766
767	MGCS36089_00958			-	YlbF family regulatory protein	299.8	767
768	MGCS36089_01232			*smc*	chromosome segregation protein Smc	299.5	768
769	MGCS36089_03050			-	NUDIX hydrolase	299.5	768
770	MGCS36089_01756			*mvaD*	diphosphomevalonate decarboxylase MvaD	297.5	770
771	MGCS36089_03200			*aroGA*	bifunctional 3-deoxy-7-phosphoheptulonate synthase/chorismate mutase AroGA	296.3	771
772	MGCS36089_03796			-	DUF1447 family protein	296.3	771
773	MGCS36089_01600			*rgpB*	glycosyltransferase family GT2 protein RgpB	296.0	773
774	MGCS36089_02240			*fhs_1*	formate--tetrahydrofolate ligase Fhs. CW-Pred predicted sortase C cell-wall anchoring	294.3	774
775	MGCS36089_00344			*proC*	pyrroline-5-carboxylate reductase ProC	294.0	775
776	MGCS36089_02254			*coaB*	phosphopantothenate--cysteine ligase CoaB	293.3	776
777	MGCS36089_01536			*glgA*	glycogen synthase GlgA	292.3	777
778	MGCS36089_02628			*recJ*	single-stranded-DNA-specific exonuclease RecJ	292.0	778
779	MGCS36089_02308			*ribF*	bifunctional riboflavin kinase/FAD synthetase	290.3	779
780	MGCS36089_04042			*cspA*	cold-shock protein CspA	290.3	779
781	MGCS36089_03250			-	LLM class flavin-dependent oxidoreductase	289.8	781
782	MGCS36089_04096			*nrdG*	anaerobic ribonucleoside-triphosphate reductase	289.8	781
783	MGCS36089_00484			*rarA*	replication-associated recombination protein A	289.3	783
784	MGCS36089_03836			*thiN*	thiamine diphosphokinase ThiN	288.5	784
785	MGCS36089_03600			-	HAD-related haloacid dehalogenase hydrolase	288.3	785
786	MGCS36089_00742			*lytS*	TCS sensor histidine kinase LytS	288.0	786
787	MGCS36089_00520			*relA*	bifunctional (p)ppGpp synthase/hydrolase RelA	286.0	787
788	MGCS36089_00976			*holB*	DNA polymerase III subunit delta' HolB	285.3	788
789	MGCS36089_01214			-	DUF3114 domain-containing protein	284.8	789
790	MGCS36089_01654			-	hypothetical protein	284.5	790
791	MGCS36089_02634			-	hypothetical protein	284.3	791
792	MGCS36089_00948			*murE_1*	UDP-N-acetylmuramoyl-L-alanyl-D-glutamate--L-lysine ligase MurE	284.0	792
793	MGCS36089_00854			*ktrB*	potassium uptake transporter channel subunit	283.8	793
794	MGCS36089_00960			-	YlbG family protein	283.5	794

No.	Locus tag	Signal6P predicted[(1)]	Virulence[(2)]	Gene	Function	RPKMs[(3)]	RANK[(4)]
795	MGCS36089_01946			-	gamma-glutamyl-gamma-aminobutyrate hydrolase	283.0	795
796	MGCS36089_00810			*yqeG*	HAD IIIA-type phosphatase YqeG	282.5	796
797	MGCS36089_02214			-	Uup family ATPase components of ABC transporters	282.3	797
798	MGCS36089_00146			*ruvB*	Holliday junction branch migration DNA helicase	281.8	798
799	MGCS36089_01734			-	tRNA CCA-pyrophosphorylase	281.0	799
800	MGCS36089_00876			*snf*	SWF/SNF family helicase	280.8	800
801	MGCS36089_02616			*zupT*	ZIP family metal transporter ZupT	280.5	801
802	MGCS36089_03432			-	YadS family trimeric intracellular cation	280.5	801
803	MGCS36089_04094			*yaaA*	peroxide stress protein YaaA	280.5	801
804	MGCS36089_02570			-	hypothetical protein	280.0	804
805	MGCS36089_03340			*luxS*	S-ribosylhomocysteine lyase	279.5	805
806	MGCS36089_01974			*osmF*	OsmF superfamily glycine/betaine transport	278.8	806
807	MGCS36089_01084			*phoH*	phosphate starvation-inducible protein PhoH	278.3	807
808	MGCS36089_01962			*nadK*	NAD kinase NadK	278.3	807
809	MGCS36089_02532			-	TIGR01906 family membrane protein	278.3	807
810	MGCS36089_02838			-	LCP family anionic cell polymer synthesis	278.3	807
811	MGCS36089_01598			*rgpA*	glycosyltransferase family 1 protein RgpA	277.0	811
812	MGCS36089_01784			-	hypothetical protein	277.0	811
813	MGCS36089_03944			*tadA*	tRNA adenosine(34) deaminase TadA	277.0	811
814	MGCS36089_03696			-	ElaA-related predicted N-acetyltransferase	276.8	814
815	MGCS36089_01562			*pheS*	phenylalanyl-tRNA synthetase alpha subunit PheS	276.0	815
816	MGCS36089_01464			-	GNAT family N-acetyltransferase	275.8	816
817	MGCS36089_02352			-	amino acid ABC transporter permease	275.8	816
818	MGCS36089_03898			*radA*	DNA repair protein RadA	275.8	816
819	MGCS36089_00844			*metN_1*	methionine ABC transporter ATP-binding protein	275.3	819
820	MGCS36089_01664			-	L21_leader RNA	275.3	819
821	MGCS36089_04102			-	hypothetical protein	275.3	819
822	MGCS36089_00744			*lytR*	TCS DNA-binding response regulator LytR	273.8	822
823	MGCS36089_01772			*mvaS2*	hydroxymethylglutaryl-CoA synthase protein (2)	273.8	822
824	MGCS36089_03094			-	MdlB family multidrug ABC transporter	273.5	824
825	MGCS36089_02942			*queA*	S-adenosylmethionine ribosyltransferase-isomerase QueA	273.0	825
826	MGCS36089_03678			*mutS2*	DNA mismatch repair endonuclease MutS2	273.0	825
827	MGCS36089_01330			*btuE*	BtuE superfamily glutathione peroxidase	271.8	827
828	MGCS36089_01484			-	HAD-IA family hydrolase	271.8	827
829	MGCS36089_01262			-	NUDIX hydrolase superfamily protein	271.3	829
830	MGCS36089_02496			-	CRISPR-DR22 RNA	271.0	830
831	MGCS36089_03900			*dut*	deoxyuridine 5'-triphosphate nucleotidohydrolase	271.0	830
832	MGCS36089_03362			*ansP*	AnsP family L-asparagine transporter	270.8	832
833	MGCS36089_04130			*ruvA*	Holliday junction ATP-dependent DNA helicase	270.5	833
834	MGCS36089_02420			-	hypothetical protein	270.3	834
835	MGCS36089_00292			*cydB*	cytochrome d ubiquinol oxidase subunit (II)	268.5	835
836	MGCS36089_03076			*murD*	UDP-N-acetylmuramoyl-L-alanine--D-glutamate	268.5	835

No.	Locus tag	Signal6P predicted[(1)]	Virulence[(2)]	Gene	Function	RPKMs[(3)]	RANK[(4)]
837	MGCS36089_02836			-	23S rRNA (uracil(1939)-C(5))-methyltransferase	267.8	837
838	MGCS36089_00790	Secreted		*dacA_1*	secreted D-alanyl-D-alanine carboxypeptidase	266.5	838
839	MGCS36089_00148			-	low molecular weight phosphotyrosine protein	266.3	839
840	MGCS36089_02138			-	heme response regulator HssR-like putative TCS	265.5	840
841	MGCS36089_02400			*pyrC*	dihydroorotase PyrC	265.0	841
842	MGCS36089_03386			*proB*	glutamate 5-kinase ProB	264.8	842
843	MGCS36089_02892			*srmB*	SrmB superfamily II DNA and RNA helicase	264.5	843
844	MGCS36089_00494			*rsmE*	16S rRNA (uracil(1498)-N(3))-methyltransferase	264.3	844
845	MGCS36089_00926			-	PgpB family membrane-associated phospholipid	264.0	845
846	MGCS36089_01162			*rnr*	exoribonuclease (R) Rnr	263.8	846
847	MGCS36089_03604			-	aldo/keto reductase	263.3	847
848	MGCS36089_04098			-	putative acetyltransferase	262.8	848
849	MGCS36089_03790			*tsaD*	tRNA (adenosine(37)-N6)-threonylcarbamoyltransferase complex transferase subunit TsaD	262.3	849
850	MGCS36089_00380			-	PAS domain-containing protein	261.5	850
851	MGCS36089_01710			*trmD*	tRNA (guanosine(37)-N1)-methyltransferase TrmD	261.0	851
852	MGCS36089_04020			-	translation initiation inhibitor	261.0	851
853	MGCS36089_02832			-	DinB family protein	260.3	853
854	MGCS36089_00950			*murJ*	peptidoglycan lipid-II intermediate flippase	260.0	854
855	MGCS36089_02534			*nagD*	NagD family hydrolase	259.0	855
856	MGCS36089_03608			*cbiO*	cobalt ECF transporter (A) ATPase component	259.0	855
857	MGCS36089_04232			-	NUDIX domain-containing protein	256.8	857
858	MGCS36089_00848			*brnQ_1*	branched-chain amino acid transport system II	255.8	858
859	MGCS36089_03306		Virulence	*liaR*	three component system signal transduction	255.5	859
860	MGCS36089_00540			*uppS*	UDP pyrophosphate synthase UppS	255.3	860
861	MGCS36089_00386			-	toxic anion resistance protein, tellurite	255.0	861
862	MGCS36089_03844			*rsmA*	16S rRNA (adenine(1518)-N(6)/adenine(1519)-N(6))- dimethyltransferase RsmA	255.0	861
863	MGCS36089_02358			*lepB_1*	signal peptidase I	254.5	863
864	MGCS36089_03030			*folD*	bifunctional methylenetetrahydrofolate dehydrogenase/methenyltetrahydrofolate cyclohydrolase FolD protein	254.5	863
865	MGCS36089_03718			*pepX*	PepX family Xaa-Pro dipeptidyl-peptidase	254.5	863
866	MGCS36089_02620			*trmK*	tRNA (adenine(22)-N(1))-methyltransferase TrmK	253.3	866
867	MGCS36089_04250			*cbiO2*	cobalt ABC transporter ATPase CbiO1	252.8	867
868	MGCS36089_00992			*ogt*	O6-methylguanine-DNA--protein-cysteine	252.3	868
869	MGCS36089_01906			*thrB*	ThrB family homoserine kinase	251.5	869
870	MGCS36089_01298			-	PrtC family collagenase-like protease	251.3	870
871	MGCS36089_01674			*lspA*	lipoprotein signal peptidase II LspA	251.3	870
872	MGCS36089_04134			*mutL*	DNA mismatch repair endonuclease MutL	250.3	872
873	MGCS36089_01554			-	DUF1146 domain-containing protein	249.8	873
874	MGCS36089_01750			*sptR*	SptR-like TCS DNA-binding response regulator	249.5	874
875	MGCS36089_00774			*uppP*	undecaprenyl pyrophosphate phosphatase UppP	249.3	875
876	MGCS36089_03972			*bglG_3*	BglG family transcription antiterminator	249.3	875
877	MGCS36089_01246			-	MFS transporter	249.0	877

No.	Locus tag	Signal6P predicted[(1)]	Virulence[(2)]	Gene	Function	RPKMs[(3)]	RANK[(4)]
878	MGCS36089_02494			-	CRISPR-DR22 RNA	248.8	878
879	MGCS36089_02618			-	Nif3-like dinuclear metal center hexameric	248.8	878
880	MGCS36089_00978			*yaaT*	cell fate regulator YaaT	248.0	880
881	MGCS36089_02982			*recR*	recombination mediator RecR	247.5	881
882	MGCS36089_02310			*truB*	tRNA pseudouridine(55) synthase TruB	247.3	882
883	MGCS36089_02648			-	rhodanese-related sulfurtransferase	247.3	882
884	MGCS36089_00006			-	DUF951 domain-containing protein	246.5	884
885	MGCS36089_00160			*thrC*	threonine synthase ThrC	246.3	885
886	MGCS36089_02058			-	hypothetical protein	245.5	886
887	MGCS36089_00362		Virulence	*rofA*	pilus transcriptional regulator RofA	244.3	887
888	MGCS36089_03122			*sdrC*	SdrC family PDZ domain-containing protein	244.0	888
889	MGCS36089_02546			-	DisA N domain-containing diadenylate cyclase	242.8	889
890	MGCS36089_02834			-	CD1845 family protein	242.8	889
891	MGCS36089_04138			-	YmcA-related cell fate/competence/biofilm	242.5	891
892	MGCS36089_03606			*recG*	ATP-dependent DNA helicase RecG	242.0	892
893	MGCS36089_03096			-	SmdA family multidrug ABC transporter	240.5	893
894	MGCS36089_01164			*smpB*	SsrA(tmRNA)-binding protein SmpB	240.3	894
895	MGCS36089_01986			*pptA*	phenylpyruvate tautomerase PptA	240.0	895
896	MGCS36089_03800			*oppF_2*	oligopeptide ABC transporter ATPase OppF	239.8	896
897	MGCS36089_03308		Virulence	*liaS*	three component system signal transduction	238.8	897
898	MGCS36089_03496			*hit*	HIT family protein	238.8	897
899	MGCS36089_03710			-	beta-lactamase family protein	238.5	899
900	MGCS36089_01432			*cofD*	CofD/YvcK superfamily 2-phospho-L-lactate	238.3	900
901	MGCS36089_03028			-	PfkB superfamily kinase	237.5	901
902	MGCS36089_03610			*cbiQ_1*	cobalt ECF transporter (T) transmembrane	237.0	902
903	MGCS36089_01652			-	dihydrofolate synthase	236.8	903
904	MGCS36089_04264			*recF*	DNA replication/repair protein RecF	236.8	903
905	MGCS36089_02148			-	NusG domain II-containing protein	236.5	905
906	MGCS36089_00154			*adhE*	bifunctional acetaldehyde-CoA/alcohol dehydrogenase AdhE	236.0	906
907	MGCS36089_01468			*add*	adenosine deaminase Add	235.5	907
908	MGCS36089_00512			*nrdI_1*	class Ib ribonucleoside-diphosphate reductase	234.5	908
909	MGCS36089_02602			*mutX*	8-oxo-dGTP diphosphatase MutX	234.3	909
910	MGCS36089_03016			*recN*	DNA repair protein RecN	234.3	909
911	MGCS36089_02248			*gcvH*	glycine cleavage system protein H GcvH	233.8	911
912	MGCS36089_02658			*malR*	maltose operon transcriptional repressor MalR	233.5	912
913	MGCS36089_01430			*rapZ*	RNase adapter RapZ	233.0	913
914	MGCS36089_02956	Secreted		*plsC*	secreted 1-acyl-sn-glycerol-3-phosphate	232.5	914
915	MGCS36089_04248			*cbiQ_2*	cobalt ABC transporter permease CbiQ	232.3	915
916	MGCS36089_00990			-	GNAT family N-acetyltransferase	231.5	916
917	MGCS36089_01746	Secreted		-	extracellular cell wall anchored bifunctional. Cell-wall anchoring predicted sortase	231.5	916
918	MGCS36089_00334			-	class I SAM-dependent methyltransferase	231.0	918
919	MGCS36089_02922			*yrrM*	YrrM family O-methyltransferase	230.5	919

No.	Locus tag	Signal6P predicted[(1)]	Virulence[(2)]	Gene	Function	RPKMs[(3)]	RANK[(4)]
920	MGCS36089_02142			-	cupin domain-containing protein	229.5	920
921	MGCS36089_01062			-	DUF3397 domain-containing protein	229.3	921
922	MGCS36089_03752			-	major facilitator superfamily protein	229.3	921
923	MGCS36089_00868			*nrdR*	transcriptional regulator NrdR	228.8	923
924	MGCS36089_01728			-	hypothetical protein	228.8	923
925	MGCS36089_01334			*ppc*	phosphoenolpyruvate carboxylase Ppc	227.3	925
926	MGCS36089_01420			-	MBL fold metallo-hydrolase	227.0	926
927	MGCS36089_01240			-	Cof-type HAD-IIB family hydrolase	226.8	927
928	MGCS36089_02474			*yeiH*	YeiH family uncharacterized membrane protein	226.3	928
929	MGCS36089_00946			*fhuA*	ferrichrome ABC transporter ATP-binding protein	226.0	929
930	MGCS36089_03720			-	pyridoxamine 5'-phosphate oxidase family	225.5	930
931	MGCS36089_04140			*argR_3*	arginine repressor ArgR	225.5	930
932	MGCS36089_03880		Virulence	*fasA*	TCS response regulator	225.0	932
933	MGCS36089_01988			*tdk*	thymidine kinase Tdk	224.8	933
934	MGCS36089_01560			*endA*	DNA-entry competence-associated nuclease EndA	224.5	934
935	MGCS36089_01158			-	multidrug efflux MFS transporter	223.0	935
936	MGCS36089_01010			-	hypothetical protein	220.8	936
937	MGCS36089_01900	Secreted		*dacA_3*	secreted D,D-carboxypeptidase penicillin-binding	220.3	937
938	MGCS36089_01268			*pnpS*	phosphate TCS signal transduction histidine	220.0	938
939	MGCS36089_03936			-	hypothetical protein	219.5	939
940	MGCS36089_02696			-	hypothetical protein	219.0	940
941	MGCS36089_00394			*pflC*	pyruvate formate-lyase activating enzyme PflC	217.8	941
942	MGCS36089_01534			*glgD*	glucose-1-phosphate adenylyltransferase subunit	217.8	941
943	MGCS36089_00414			*pgpA*	phosphatidylglycerophosphatase protein PgpA	217.5	943
944	MGCS36089_02722			-	CorA family divalent cation transport protein	217.5	943
945	MGCS36089_03128			*asnA*	asparagine synthetase AsnA	217.0	945
946	MGCS36089_03124			*coaD*	pantetheine-phosphate adenylyltransferase CoaD	216.5	946
947	MGCS36089_00492			*prmA*	50S ribosomal protein L11 methyltransferase	216.0	947
948	MGCS36089_02366			*yhcF*	YhcF family transcriptional regulator	216.0	947
949	MGCS36089_00486			*pabA*	aminodeoxychorismate/anthranilate synthase	215.8	949
950	MGCS36089_02960			-	GIY-YIG catalytic domain-containing putative	215.8	949
951	MGCS36089_02202			-	DUF1836 domain-containing protein	215.3	951
952	MGCS36089_02068	Lipo		*dppA*	dipeptide-binding ABC transport system	215.0	952
953	MGCS36089_00510			-	PreQ1 RNA	214.5	953
954	MGCS36089_03846			*rrmV*	5S rRNA maturation endonuclease RnmV	213.8	954
955	MGCS36089_04270			*trpS*	tryptophanyl-tRNA synthetase	213.5	955
956	MGCS36089_00856			*rsmG*	16S rRNA (guanine(527)-N(7))-methyltransferase	213.0	956
957	MGCS36089_03146			*argR_2*	arginine responsive transcriptional repressor	213.0	956
958	MGCS36089_02252			*lipL*	lipoate--protein ligase	212.8	958
959	MGCS36089_02642			-	C4-dicarboxylate ABC transporter	212.8	958
960	MGCS36089_02536			*fatA*	FatA family acyl-[acyl-carrier-protein]	212.3	960
961	MGCS36089_02314			-	aromatic acid exporter family protein	211.5	961

No.	Locus tag	Signal6P predicted[(1)]	Virulence[(2)]	Gene	Function	RPKMs[(3)]	RANK[(4)]
962	MGCS36089_02140			-	putative TCS histidine kinase sensor	210.8	962
963	MGCS36089_02576			*phnK*	PhnK family uncharacterized ABC transporter	210.8	962
964	MGCS36089_02130			-	hypothetical protein	210.5	964
965	MGCS36089_04272			*yitT*	putative membrane anchor protein YitT	210.3	965
966	MGCS36089_03612			-	ECF transporter (S) specificity component	210.0	966
967	MGCS36089_01650			*gorA*	glutathione reductase GorA	209.8	967
968	MGCS36089_02302			-	inositol monophosphatase family protein	209.8	967
969	MGCS36089_03510			-	SSRC34_2 RNA	209.8	967
970	MGCS36089_03802			*oppD_2*	oligopeptide ABC transporter ATPase OppD	208.8	970
971	MGCS36089_03804			*oppC_2*	oligopeptide ABC transporter permease OppC	208.8	970
972	MGCS36089_00496			-	LacI family DNA-binding transcriptional	207.8	972
973	MGCS36089_01672			*lysR*	LysR family transcriptional regulator	207.8	972
974	MGCS36089_03428			-	TetR/AcrR family transcriptional regulator	207.8	972
975	MGCS36089_03654			*comEB*	competence protein ComEB	207.8	972
976	MGCS36089_01970			-	Na+ driven multidrug efflux pump	207.3	976
977	MGCS36089_00018			-	RNA-binding S4 domain-containing protein	206.3	977
978	MGCS36089_01374			-	hypothetical protein	205.8	978
979	MGCS36089_04038			*ctsR*	CtsR family transcriptional regulator	205.8	978
980	MGCS36089_00488			*pabB*	para-aminobenzoate synthetase	205.0	980
981	MGCS36089_03882		Virulence	*fasC*	TCS histidine kinase	205.0	980
982	MGCS36089_00290			*cydA*	cytochrome ubiquinol oxidase subunit (I) CydA	204.5	982
983	MGCS36089_02402			*ung*	uracil-DNA glycosylase Ung	204.5	982
984	MGCS36089_03954			-	DUF975 family protein	204.5	982
985	MGCS36089_01972			*opuBA*	OpuBA superfamily glycine/betaine ABC	204.3	985
986	MGCS36089_01744			*fms*	peptide deformylase	204.0	986
987	MGCS36089_01936			*eriC*	EriC family voltage gated chloride channel	203.3	987
988	MGCS36089_01748			-	GTP pyrophosphokinase family protein	203.0	988
989	MGCS36089_01366			-	HD domain-containing phosphohydrolase	202.8	989
990	MGCS36089_01656			*nifS_1*	cysteine desulfurase NifS	202.5	990
991	MGCS36089_00506			-	ECF transporter S component	202.3	991
992	MGCS36089_01646			*aroC*	chorismate synthase AroC	201.8	992
993	MGCS36089_00944	Lipo		*fhuD*	iron-hydroxamate ABC transporter	201.3	993
994	MGCS36089_02902			*nrdH*	glutaredoxin-like protein NrdH	201.3	993
995	MGCS36089_01898			*xerD_2*	site-specific integrase	200.8	995
996	MGCS36089_03942	Lipo		-	CYK3 family lipoprotein putatively involved in	200.5	996
997	MGCS36089_04234			-	Spd-sr37 RNA	200.5	996
998	MGCS36089_02398			*plsY*	glycerol-3-phosphate 1-O-acyltransferase PlsY	199.8	998
999	MGCS36089_02124			*mngR*	MngR family DNA-binding transcriptional	199.5	999
1000	MGCS36089_01038			-	UhpC family MFS transporter	199.3	1000
1001	MGCS36089_01492			-	DUF1694 domain-containing protein	199.3	1000
1002	MGCS36089_02018			-	vicinal oxygen chelate (VOC) family protein	198.8	1002
1003	MGCS36089_01436			*pepD_1*	C69 family dipeptidase PepD	198.5	1003

No.	Locus tag	Signal6P predicted[(1)]	Virulence[(2)]	Gene	Function	RPKMs[(3)]	RANK[(4)]
1004	MGCS36089_02908			*clcB*	voltage-gated ClC-type chloride channel ClcB	197.3	1004
1005	MGCS36089_02492			-	CRISPR-DR22 RNA	196.5	1005
1006	MGCS36089_02274			*deoA*	pyrimidine-nucleoside phosphorylase DeoA	196.0	1006
1007	MGCS36089_03452			*copA*	copper-exporting ATPase CopA	195.8	1007
1008	MGCS36089_02134			-	helix-turn-helix transcriptional regulator	195.5	1008
1009	MGCS36089_03088			-	DUF910 domain-containing protein	195.3	1009
1010	MGCS36089_00288			*nox*	NAD(P)/FAD-dependent oxidoreductase Nox	194.8	1010
1011	MGCS36089_02448			-	NAD-dependent succinate-semialdehyde	194.8	1010
1012	MGCS36089_01942			-	AI-2E family transporter	194.5	1012
1013	MGCS36089_02126			*ylxM*	YlxM superfamily signal recognition particle	194.3	1013
1014	MGCS36089_02316			*hsdS*	type I restriction endonuclease subunit S	194.3	1013
1015	MGCS36089_02614			*dadA*	FAD-binding oxidoreductase DadA	194.3	1013
1016	MGCS36089_02984			*pbp2B*	penicillin-binding protein PBP2B/FtsI	194.3	1013
1017	MGCS36089_00780			*sufC*	Fe-S cluster assembly ATPase SufC	194.0	1017
1018	MGCS36089_02304			-	UPF0223 family protein	194.0	1017
1019	MGCS36089_03014	Secreted		-	putative extracellular cell surface	193.5	1019
1020	MGCS36089_02242			*lplA_1*	lipoate--protein ligase	193.3	1020
1021	MGCS36089_00732			-	mechanosensitive ion channel family protein	193.0	1021
1022	MGCS36089_01076			*msrA*	peptide-methionine (S)-S-oxide reductase MsrA	193.0	1021
1023	MGCS36089_04258			*pqqF*	pitrilysin family predicted Zn-dependent	193.0	1021
1024	MGCS36089_02866			*fadR*	FadR family DNA-binding transcriptional	192.5	1024
1025	MGCS36089_00558			-	Crp family cyclic nucleotide-binding	192.0	1025
1026	MGCS36089_03570			-	drug/metabolite transporter superfamily protein	190.3	1026
1027	MGCS36089_03784			*yhiN*	YhiN family predicted flavoprotein	190.0	1027
1028	MGCS36089_03794			*tsaB*	tRNA (adenosine(37)-N6)-threonylcarbamoyltransferase complex dimerization subunit type 1 TsaB	190.0	1027
1029	MGCS36089_02848			*spxR*	SpxR family CBS-HotDog domain-containing	189.3	1029
1030	MGCS36089_01532			*glgC*	glucose-1-phosphate adenylyltransferase subunit	188.8	1030
1031	MGCS36089_03020			*tlyA*	TlyA family RNA methyltransferase	188.5	1031
1032	MGCS36089_01688			*carA*	carbamoyl-phosphate synthase small subunit CarA	187.8	1032
1033	MGCS36089_03918			-	5-formyltetrahydrofolate cyclo-ligase	187.8	1032
1034	MGCS36089_03636			-	IS30 family transposase	187.5	1034
1035	MGCS36089_00396			-	GlpR-like transcriptional regulator protein	187.0	1035
1036	MGCS36089_00508			*rihB*	pyrimidine-specific ribonucleoside hydrolase	186.5	1036
1037	MGCS36089_00988			*serC*	3-phosphoserine/phosphohydroxythreonine	186.5	1036
1038	MGCS36089_01302			-	YdbC family protein	186.5	1036
1039	MGCS36089_02488			*ndk*	nucleoside-diphosphate kinase Ndk. 1212392..1212757. CRISPR with 6 repeat units	186.5	1036
1040	MGCS36089_01266			*pnpR*	phosphate TCS signal transduction response	186.0	1040
1041	MGCS36089_02244			-	NAD-dependent deacetylase	186.0	1040
1042	MGCS36089_00826			-	nucleotidyltransferase	185.5	1042
1043	MGCS36089_03814			-	YgaE family uncharacterized protein	185.3	1043
1044	MGCS36089_02538			*hemN*	HemN family coproporphyrinogen III oxidase or	185.0	1044

No.	Locus tag	Signal6P predicted(1)	Virulence(2)	Gene	Function	RPKMs(3)	RANK(4)
1045	MGCS36089_01082			*mycA*	oleate hydratase/myosin-crossreactive antigen	183.5	1045
1046	MGCS36089_00974			*tmk*	thymidylate kinase Tmk	183.3	1046
1047	MGCS36089_01740			-	MdlB family multidrug ABC transporter ATPase and	182.5	1047
1048	MGCS36089_01476			*clcA*	voltage-gated chloride channel family protein	182.0	1048
1049	MGCS36089_03508			*cof*	Cof family hydrolase	181.8	1049
1050	MGCS36089_04136			*mutS*	DNA mismatch repair protein MutS	181.8	1049
1051	MGCS36089_01686			*pyrB*	aspartate transcarbomoylase PyrB	180.8	1051
1052	MGCS36089_02230			*cls*	cardiolipin synthase	180.8	1051
1053	MGCS36089_04180			*pezT*	zeta toxin family protein PezT	180.8	1051
1054	MGCS36089_01904			*hom*	homoserine dehydrogenase Hom	180.3	1054
1055	MGCS36089_01576			*rexB*	ATP-dependent nuclease B subunit RexB	179.8	1055
1056	MGCS36089_01148			*mutM*	DNA-formamidopyrimidine glycosylase MutM	179.3	1056
1057	MGCS36089_01712			*trxB_1*	NAD(P)/FAD-dependent oxidoreductase	179.3	1056
1058	MGCS36089_02354			*phnA*	PnhA family zinc ribbon domain-containing	179.3	1056
1059	MGCS36089_00934			*pflA*	pyruvate formate-lyase activating enzyme PflA	178.8	1059
1060	MGCS36089_01754			*mvaK1*	mevalonate kinase MvaK1	178.8	1059
1061	MGCS36089_02880			*birA*	bifunctional biotin--[acetyl-CoA-carboxylase]	178.5	1061
1062	MGCS36089_03150			-	MmcQ/YjbR family DNA-binding protein	177.8	1062
1063	MGCS36089_01150			*coaE*	dephospho-CoA kinase CoaE	177.5	1063
1064	MGCS36089_03448			*glpR*	GlpR family DNA-binding transcriptional	177.3	1064
1065	MGCS36089_03038			*feoB*	ferrous iron transport protein (B) FeoB	177.0	1065
1066	MGCS36089_02450			*yjjG*	YjjG family noncanonical pyrimidine	176.5	1066
1067	MGCS36089_00388			-	hypothetical protein	175.3	1067
1068	MGCS36089_03018			*argR_1*	ArgR family transcriptional regulator	174.8	1068
1069	MGCS36089_02732			*fetB*	iron export ABC transporter permease subunit	174.5	1069
1070	MGCS36089_00426			-	hypothetical protein	174.3	1070
1071	MGCS36089_00890			*acyP*	acylphosphatase AcyP	174.3	1070
1072	MGCS36089_01960			-	GTP pyrophosphokinase family protein	174.3	1070
1073	MGCS36089_02136			*yhhX*	PRK10206 superfamily putative oxidoreductase	174.3	1070
1074	MGCS36089_00620			-	DNA cytosine methyltransferase	172.3	1074
1075	MGCS36089_01078			*yozE*	YozE family protein	172.3	1074
1076	MGCS36089_02250			-	NADH-dependent flavin oxidoreductase	172.3	1074
1077	MGCS36089_01902			-	polysaccharide deacetylase family protein	172.0	1077
1078	MGCS36089_03904			-	ABC transporter ATPase/permease	171.3	1078
1079	MGCS36089_03256			*thlA*	thiolase ThlA	170.8	1079
1080	MGCS36089_01684			*pyrP*	uracil permease protein PyrP	170.5	1080
1081	MGCS36089_02048	Lipo		-	TlpA-family protein	170.5	1080
1082	MGCS36089_00530			*dexB*	glucan 1,6-alpha-glucosidase DexB	170.3	1082
1083	MGCS36089_00986			*cutC*	copper homeostasis protein CutC	170.3	1082
1084	MGCS36089_03048			*clpA*	ATP-dependent Clp protease ATP-binding subunit	170.3	1082
1085	MGCS36089_01790			-	L10_leader RNA	169.0	1085
1086	MGCS36089_03916			*glpG*	GlpG family membrane associated serine protease	168.5	1086

No.	Locus tag	Signal6P predicted[(1)]	Virulence[(2)]	Gene	Function	RPKMs[(3)]	RANK[(4)]
1087	MGCS36089_03740			-	ABC transporter permease	167.8	1087
1088	MGCS36089_02246			-	protein-ADP-ribose hydrolase	166.8	1088
1089	MGCS36089_01984			-	thiamine biosynthesis protein ApbE-like protein	166.3	1089
1090	MGCS36089_03924			-	Mga-related helix-turn-helix domain-containing	166.3	1089
1091	MGCS36089_00772	Secreted		-	DUF2207 domain-containing secreted protein	166.0	1091
1092	MGCS36089_02444			*uvrC*	excinuclease ABC subunit UvrC	166.0	1091
1093	MGCS36089_00424			-	putative transcriptional regulator	165.8	1093
1094	MGCS36089_02554			-	DUF4097 family beta strand repeat-containing	165.8	1093
1095	MGCS36089_04182			-	helix-turn-helix domain-containing	165.0	1095
1096	MGCS36089_03022			*ispA*	IspA family geranylgeranyl pyrophosphate	164.8	1096
1097	MGCS36089_01182			*amyS*	alpha-amylase AmyS	164.5	1097
1098	MGCS36089_04054			*hutI*	imidazolonepropionase HutI	164.3	1098
1099	MGCS36089_03212			-	permease	163.3	1099
1100	MGCS36089_01460			-	IS30 family transposase	162.0	1100
1101	MGCS36089_02300			*rsmF*	RsmF family rRNA cytosine-C5-methyltransferase	162.0	1100
1102	MGCS36089_03714			-	putative sulfite exporter	161.8	1102
1103	MGCS36089_00656			-	type II toxin-antitoxin system Phd/YefM family	161.5	1103
1104	MGCS36089_00824			-	class I SAM-dependent methyltransferase	160.8	1104
1105	MGCS36089_03848			*tatD*	Tat protein secretion system quality control	160.8	1104
1106	MGCS36089_01022			*brnQ_2*	branched-chain amino acid transport system II	160.3	1106
1107	MGCS36089_02886			*prsW*	PrsW family glutamic-type intramembrane	160.3	1106
1108	MGCS36089_00382			*ridA*	RidA family protein	159.8	1108
1109	MGCS36089_00398			-	DeoR-like transcriptional regulator protein	159.5	1109
1110	MGCS36089_03778			-	MerR/SoxR family transcriptional regulator	159.5	1109
1111	MGCS36089_02172			-	PASTA domain-containing protein	158.8	1111
1112	MGCS36089_03318			*fmt*	methionyl-tRNA formyl transferase Fmt	158.8	1111
1113	MGCS36089_03310		Virulence	*liaF*	three component system signal transduction	158.5	1113
1114	MGCS36089_04286			*rlmH*	23S rRNA (pseudouridine(1915)-N(3))-methyltransferase RlmH	158.5	1113
1115	MGCS36089_02312			-	DUF2130 domain-containing protein	158.0	1115
1116	MGCS36089_02080			-	site-specific integrase	157.5	1116
1117	MGCS36089_03688			*recD*	ATP-dependent DNA helicase RecD	157.5	1116
1118	MGCS36089_04178			-	site-specific integrase	157.5	1116
1119	MGCS36089_01690			*carB*	carbamoyl-phosphate synthase large subunit CarB	157.3	1119
1120	MGCS36089_02888			-	DUF1294 domain-containing protein	157.0	1120
1121	MGCS36089_03742			-	ABC transporter ATP-binding protein	157.0	1120
1122	MGCS36089_01000			*exoA*	exodeoxyribonuclease III protein ExoA	156.8	1122
1123	MGCS36089_03806			*oppB_2*	oligopeptide ABC transporter permease OppB	156.3	1123
1124	MGCS36089_00428			-	FAD/NAD(P)-binding protein	156.0	1124
1125	MGCS36089_02004			-	MdlB superfamily multidrug ABC transporter	155.8	1125
1126	MGCS36089_03258			*fadB*	FadB family 3-hydroxyacyl-CoA dehydrogenase	155.3	1126
1127	MGCS36089_01708			*rimM*	ribosome maturation factor RimM	155.0	1127
1128	MGCS36089_03126			*rsmD*	16S rRNA (guanine(966)-N(2))-methyltransferase	155.0	1127

No.	Locus tag	Signal6P predicted[1]	Virulence[2]	Gene	Function	RPKMs[3]	RANK[4]
1129	MGCS36089_00730			-	DUF436 family protein	154.8	1129
1130	MGCS36089_03024			*xseB*	exodeoxyribonuclease VII small subunit XseB	154.8	1129
1131	MGCS36089_04260			*pqqL*	pitrilysin family predicted Zn-dependent	154.8	1129
1132	MGCS36089_01368			-	DUF1934 domain-containing protein	154.3	1132
1133	MGCS36089_02364			*dnaE*	DNA polymerase III subunit alpha DnaE	153.5	1133
1134	MGCS36089_00490			-	DUF3013 family protein	153.3	1134
1135	MGCS36089_02842			*aroA*	3-phosphoshikimate 1-carboxyvinyltransferase	153.3	1134
1136	MGCS36089_03210			-	permease	153.3	1134
1137	MGCS36089_02568			-	ABC transporter ATP-binding protein	151.8	1137
1138	MGCS36089_02342			*alsT*	sodium:alanine symporter family protein	151.5	1138
1139	MGCS36089_01358			-	FMN-binding domain containing L-lactate oxidase	151.3	1139
1140	MGCS36089_02840			*aroK*	shikimate kinase AroK	151.3	1139
1141	MGCS36089_01028			-	DUF2829 domain-containing protein	150.8	1141
1142	MGCS36089_02578			-	putative ABC transporter permease component	150.8	1141
1143	MGCS36089_02060			*dppF*	dipeptide ABC transport system ATP-binding	150.5	1143
1144	MGCS36089_02930			-	NAD(P)H-dependent oxidoreductase	150.3	1144
1145	MGCS36089_01212			*ppsB*	long-chain fatty acid--CoA ligase PpsB	149.8	1145
1146	MGCS36089_03938	Secreted	Virulence	*speG*	streptococcal pyrogenic exotoxin (G) SpeG	149.3	1146
1147	MGCS36089_03950			-	CHY zinc finger domain-containing protein	149.3	1146
1148	MGCS36089_01210			*paaJ*	acetyl-CoA acetyl transferase PaaJ	148.0	1148
1149	MGCS36089_02406	Lipo		-	amino-acid ABC transporter substrate-binding	148.0	1148
1150	MGCS36089_02376			-	SDUF3862 domain-containing lipoprotein. ignalP-6 predicted lipid anchoring signal peptide,	147.8	1150
1151	MGCS36089_00162			-	MATE family multidrug efflux transporter	147.5	1151
1152	MGCS36089_03656			*pepP*	PepP family Xaa-Pro peptidase	147.5	1151
1153	MGCS36089_00600			-	L13_leader RNA	147.3	1153
1154	MGCS36089_01738			-	MdlB family multidrug ABC transporter ATPase and	147.3	1153
1155	MGCS36089_03450			*copZ_2*	copper chaperone CopZ	146.8	1155
1156	MGCS36089_00034			-	IS30 family transposase	146.5	1156
1157	MGCS36089_00108			*recO*	DNA repair protein RecO	146.5	1156
1158	MGCS36089_00586	Secreted		-	putative secreted protein	146.5	1156
1159	MGCS36089_04276			*yfhO*	YfhO family protein	145.0	1159
1160	MGCS36089_01530			*glgB*	1,4-alpha-glucan branching protein GlgB	144.8	1160
1161	MGCS36089_02482			-	RnaY family phopsphodiesterase	144.8	1160
1162	MGCS36089_01422			*dinG*	bifunctional DnaQ family exonuclease -- ATP-dependent helicase DinG	144.5	1162
1163	MGCS36089_02372			-	TVP38/TMEM64 family protein	143.0	1163
1164	MGCS36089_02724			*rsuA_1*	ribosomal small subunit pseudouridine synthase	143.0	1163
1165	MGCS36089_03162			-	CorA family divalent cation transport protein	142.3	1165
1166	MGCS36089_03736	Secreted		-	putative secreted protein	142.3	1165
1167	MGCS36089_02006			-	MdlB superfamily multidrug ABC transporter	142.0	1167
1168	MGCS36089_01318			*thiT*	energy-coupled thiamine transporter ThiT	141.5	1168
1169	MGCS36089_03906			-	ABC transporter ATPase/permease	141.3	1169
1170	MGCS36089_03144			*fnr*	Crp/Fnr family transcriptional regulator	141.0	1170

No.	Locus tag	Signal6P predicted[(1)]	Virulence[(2)]	Gene	Function	RPKMs[(3)]	RANK[(4)]
1171	MGCS36089_01408			-	HAD family phosphatase	140.8	1171
1172	MGCS36089_00526			*lrp*	PucR family transcriptional regulator/leucine	140.3	1172
1173	MGCS36089_00016			-	oligosaccharide flippase family protein	139.8	1173
1174	MGCS36089_00158			-	hypothetical protein	139.8	1173
1175	MGCS36089_00668			-	hypothetical protein	139.5	1175
1176	MGCS36089_00930			-	RsmB/NOP family class I SAM-dependent RNA	139.5	1175
1177	MGCS36089_00450			-	hypothetical protein	139.3	1177
1178	MGCS36089_00296			*cdyC*	thiol reductant ABC exporter subunit CydC	138.0	1178
1179	MGCS36089_00342			-	hypothetical protein	138.0	1178
1180	MGCS36089_03152		Virulence	*yesM*	TCS sensor kinase YesM	137.8	1180
1181	MGCS36089_03792			*rimI*	ribosomal-protein-alanine N-acetyltransferase	137.5	1181
1182	MGCS36089_00728			-	ECF transporter S component	137.3	1182
1183	MGCS36089_00920			*trmI*	TrmL family tRNA (cytosine34-2'-O-)-methyl	137.3	1182
1184	MGCS36089_03598			*uspA*	UspA family nucleotide-binding universal stress	137.3	1182
1185	MGCS36089_00008			-	helix-turn-helix domain-containing protein	137.0	1185
1186	MGCS36089_02044	Secreted	Virulence	*isp*	secreted CHAP domain-containing immunogenic	136.5	1186
1187	MGCS36089_03748			-	MFS transporter	136.5	1186
1188	MGCS36089_01682			*pyrR*	bifunctional pyrimidine regulatory protein/uracil phosphoribosyltransferase PyrR	136.0	1188
1189	MGCS36089_02850			-	GNAT family N-acetyltransferase	135.5	1189
1190	MGCS36089_03252			*caiC*	CaiC family Acyl-CoA synthetase	135.5	1189
1191	MGCS36089_01482			*rodA*	rod shape-determining protein RodA	135.3	1191
1192	MGCS36089_02122			-	hypothetical protein	135.3	1191
1193	MGCS36089_03896			-	carbonic anhydrase	135.3	1191
1194	MGCS36089_01882			-	integrase catalytic subunit	135.0	1194
1195	MGCS36089_03422			*nptA*	NtpA family Na/Pi cotransporter	135.0	1194
1196	MGCS36089_03744			-	PLDc N-terminal domain-containing protein	135.0	1194
1197	MGCS36089_02668			*malH*	LacI family transcriptional regulatory protein	134.8	1197
1198	MGCS36089_00726			*thiD*	bifunctional hydroxymethylpyrimidine kinase/phosphomethylpyrimidine kinase PdxK	134.3	1198
1199	MGCS36089_02452			-	DUF1524 domain-containing protein	133.8	1199
1200	MGCS36089_03214			*aroE*	shikimate dehydrogenase AroE	133.8	1199
1201	MGCS36089_01660			*capA*	CapA family protein	133.0	1201
1202	MGCS36089_01786			*clpL*	ATP-dependent Clp protease ATP-binding subunit	132.5	1202
1203	MGCS36089_02666			*malG*	maltose/maltodextrin ABC transport system	132.5	1202
1204	MGCS36089_02580			-	putative ABC transporter substrate binding	131.3	1204
1205	MGCS36089_00940			*fhuG*	iron ABC transporter permease FhuG	131.0	1205
1206	MGCS36089_02228			-	asd RNA	131.0	1205
1207	MGCS36089_02946	Lipo		*blaA*	beta-lactamase-related serine hydrolase	130.8	1207
1208	MGCS36089_03746			-	Cro/CI family transcriptional regulator	130.8	1207
1209	MGCS36089_04026			-	hypothetical protein	129.8	1209
1210	MGCS36089_02596			*yloA*	YloA family predicted ribosome quality control	129.5	1210
1211	MGCS36089_03040			*feoA*	ferrous iron transport protein (A) FeoA	129.0	1211
1212	MGCS36089_02180			-	DUF3307 domain-containing protein	128.8	1212

No.	Locus tag	Signal6P predicted[(1)]	Virulence[(2)]	Gene	Function	RPKMs[(3)]	RANK[(4)]
1213	MGCS36089_01880			*cadA*	cadmium-translocating P-type ATPase CadA	128.5	1213
1214	MGCS36089_03290			-	IS30 family transposase	127.8	1214
1215	MGCS36089_02046			-	hypothetical protein	127.3	1215
1216	MGCS36089_00294			*cydD*	thiol reductant ABC exporter subunit CydD	127.0	1216
1217	MGCS36089_02606			-	Abi family CAAX protease self-immunity protein	126.8	1217
1218	MGCS36089_02640			-	DUF3042 family protein	126.5	1218
1219	MGCS36089_02484			*msrB*	peptide-methionine (R)-S-oxide reductase MsrB	126.3	1219
1220	MGCS36089_03424			-	hypothetical protein	126.3	1219
1221	MGCS36089_03902			*queH*	epoxyqueuosine reductase QueH	126.3	1219
1222	MGCS36089_00286			-	1,4-dihydroxy-2-naphthoate octaprenyltransferase	126.0	1222
1223	MGCS36089_01958			*yjbK*	YbjK superfamily CYTH domain-containing	126.0	1222
1224	MGCS36089_00670	Pilin		-	putative lipoprotein	125.8	1224
1225	MGCS36089_00548			-	nucleotidyltransferase family protein	125.3	1225
1226	MGCS36089_02156			-	polyprenyl synthetase family protein	125.3	1225
1227	MGCS36089_01572			-	ABC transporter ATP-binding component LolD-like	124.8	1227
1228	MGCS36089_02490			-	CRISPR-DR22 RNA	124.8	1227
1229	MGCS36089_01742			*gdhA*	NADP-specific glutamate dehydrogenase GdhA	124.3	1229
1230	MGCS36089_01870			*copB*	copper-exporting ATPase cCopB	124.3	1229
1231	MGCS36089_02734			*fetA*	iron export ABC transporter ATP-binding subunit	124.3	1229
1232	MGCS36089_04162			-	cadmium efflux system accessory protein	124.3	1229
1233	MGCS36089_02458			*ykgJ*	YkgJ family cysteine cluster protein	124.0	1233
1234	MGCS36089_00156			*adhP*	alcohol dehydrogenase AdhP	123.8	1234
1235	MGCS36089_00298			*preA*	polyprenyl synthetase family protein PreA	123.5	1235
1236	MGCS36089_03320			*priA*	primosomal protein PriA	123.0	1236
1237	MGCS36089_00014			*trcF*	transcription-repair coupling factor TcrF	122.8	1237
1238	MGCS36089_03260			*trmA*	TrmA RNA methyltransferase	122.8	1237
1239	MGCS36089_03738			-	GNAT family acetyltransferase	122.8	1237
1240	MGCS36089_01570			-	ABC transporter permease	122.5	1240
1241	MGCS36089_01724			-	putative peptidoglycan hydrolase	122.3	1241
1242	MGCS36089_03026			*xseA*	exodeoxyribonuclease VII large subunit XseA	122.3	1241
1243	MGCS36089_01254			*bglA*	6-phospho-beta-glucosidase BglA	121.5	1243
1244	MGCS36089_03692			*dinB*	DNA polymerase IV DinB	121.5	1243
1245	MGCS36089_01446			-	GntR family transcriptional regulator	121.3	1245
1246	MGCS36089_03262			*recX*	recombination regulator RecX	121.3	1245
1247	MGCS36089_04200			-	thioredoxin family protein	121.3	1245
1248	MGCS36089_00942			*fhuB*	iron ABC transporter permease FhuB	121.0	1248
1249	MGCS36089_02936			-	MFS transporter	120.8	1249
1250	MGCS36089_02062			*dppD*	dipeptide ABC transport system ATP-binding	120.5	1250
1251	MGCS36089_01002			*sdpI*	SdpI family immunity protein	120.3	1251
1252	MGCS36089_01768			-	HTH domain-containing putative transcriptional	120.0	1252
1253	MGCS36089_03852			*rpiR*	RpiR family transcriptional regulator	120.0	1252
1254	MGCS36089_04070			*hutG*	formiminoglutamase HutG	120.0	1252

No.	Locus tag	Signal6P predicted[(1)]	Virulence[(2)]	Gene	Function	RPKMs[(3)]	RANK[(4)]
1255	MGCS36089_04164			-	DNA-binding HTH domain-containing	120.0	1252
1256	MGCS36089_02556			-	PadR family transcriptional regulator	119.0	1256
1257	MGCS36089_01398	Secreted	Virulence	*hylB*	secreted hyaluronate lyase HylB	118.0	1257
1258	MGCS36089_02456			*ycjU*	YcjU family beta-phosphoglucomutase or related	117.5	1258
1259	MGCS36089_03164			-	Gfo/Idh/MocA family oxidoreductase. 1567148..1567775. CRISPR with 10 repeat units	117.5	1258
1260	MGCS36089_03646			*scrR*	sucrose operon repressor ScrR	117.5	1258
1261	MGCS36089_02404			-	amino acid ABC transporter permease	117.3	1261
1262	MGCS36089_00924			-	ECF transporter S component	117.0	1262
1263	MGCS36089_01574			-	neutral zinc metallopeptidase	117.0	1262
1264	MGCS36089_00138			-	hypothetical protein	116.8	1264
1265	MGCS36089_03948			*bioY*	biotin transporter BioY	116.8	1264
1266	MGCS36089_01068			-	hypothetical protein. SignalP-6 predicted standard secretion signal	116.0	1266
1267	MGCS36089_00892			-	SpoU family RNA methyltransferase	115.8	1267
1268	MGCS36089_03592			-	cysteine hydrolase	115.8	1267
1269	MGCS36089_00928			*yhcC*	YhcC family Fe-S oxidoreductase	115.0	1269
1270	MGCS36089_02986			*focA*	formate transporter FocA	115.0	1269
1271	MGCS36089_02040		Virulence	*irr*	TCS signal transduction DNA-binding response	114.5	1271
1272	MGCS36089_02412			-	GNAT family N-acetyltransferase	114.5	1271
1273	MGCS36089_00706			-	IS30 family transposase	114.0	1273
1274	MGCS36089_02344			*fieF*	FieF family cation diffusion facilitator family	114.0	1273
1275	MGCS36089_02910			-	CPBP family intramembrane metalloprotease	114.0	1273
1276	MGCS36089_01884			-	DUF4365 family protein	113.8	1276
1277	MGCS36089_02664			*malF*	maltose/maltodextrin ABC transport system	113.5	1277
1278	MGCS36089_03216			*lacZ*	beta-galactosidase LacZ	113.5	1277
1279	MGCS36089_04160			*cadD_2*	CadD family cadmium resistance transporter	113.3	1279
1280	MGCS36089_01856			-	hypothetical protein	112.8	1280
1281	MGCS36089_02042		Virulence	*ihk*	TCS signal transduction histidine kinase sensor	112.8	1280
1282	MGCS36089_01662			-	IS30 family transposase	112.3	1282
1283	MGCS36089_00164			-	IS30 family transposase	112.0	1283
1284	MGCS36089_01322			-	GH25 muramidase superfamily lysozyme	111.8	1284
1285	MGCS36089_03780			*dnaQ*	DNA polymerase III epsilon subunit DnaQ	111.3	1285
1286	MGCS36089_03454			*copY_2*	DNA-binding copper transport transcriptional	111.0	1286
1287	MGCS36089_02064			*dppC*	dipeptide ABC transport system permease protein	110.5	1287
1288	MGCS36089_03602			*asnB*	L-asparaginase AsnB	110.0	1288
1289	MGCS36089_03420			*nagA*	N-acetylglucosamine-6-phosphate deacetylase	109.8	1289
1290	MGCS36089_03036			-	FeoB associated cysteine-rich protein	109.5	1290
1291	MGCS36089_04022			-	hypothetical protein	109.3	1291
1292	MGCS36089_02990			*niaR*	niacin-responsive transcriptional repressor	109.0	1292
1293	MGCS36089_02422			-	LysR family transcriptional regulator	108.8	1293
1294	MGCS36089_04262			*ybcJ*	ribosome associated protein YbcJ	108.5	1294
1295	MGCS36089_00536			-	thioredoxin family protein	108.3	1295
1296	MGCS36089_01192			-	putative ABC transporter permease component	108.3	1295

No.	Locus tag	Signal6P predicted[(1)]	Virulence[(2)]	Gene	Function	RPKMs[(3)]	RANK[(4)]
1297	MGCS36089_00136			-	DUF4041 domain-containing protein	108.0	1297
1298	MGCS36089_03614			-	CydD-related ABC transporter ATPase/permease	107.3	1298
1299	MGCS36089_01194			-	putative ABC transporter permease component	107.0	1299
1300	MGCS36089_02988			*niaX*	niacin transporter NiaX	106.8	1300
1301	MGCS36089_02182			-	SatD family protein	106.5	1301
1302	MGCS36089_02958			*trmN6*	tRNA1(Val) A37 N6-methylase TrmN6	106.3	1302
1303	MGCS36089_02454			-	maltose/galactose O-acetyltransferase	106.0	1303
1304	MGCS36089_03732			-	BaeS family TCS sensor histidine kinase	106.0	1303
1305	MGCS36089_00596			-	helix-turn-helix transcriptional regulator	105.8	1305
1306	MGCS36089_03194			-	MOP/MATE family multidrug-resistance efflux	105.8	1305
1307	MGCS36089_02950			*holA*	DNA polymerase III delta subunit HolA	104.8	1307
1308	MGCS36089_00828			-	MerR family transcriptional regulator	104.3	1308
1309	MGCS36089_03154		Virulence	*yesN*	TCS DNA-binding response regulator YesN	104.0	1309
1310	MGCS36089_03222			*trxT*	Trx TCS operon protein TrxT	104.0	1309
1311	MGCS36089_03054			-	hypothetical protein	103.8	1311
1312	MGCS36089_02276			*rsmC*	class I SAM-dependent methyltransferase	103.5	1312
1313	MGCS36089_03522			-	acyltransferase family protein	103.5	1312
1314	MGCS36089_00280			-	XRE family ImmR-like transcriptional regulator	103.0	1314
1315	MGCS36089_01320			-	TPP RNA	103.0	1314
1316	MGCS36089_04124			*cinA*	competence/damage-inducible protein CinA	103.0	1314
1317	MGCS36089_04194			-	hypothetical protein	103.0	1314
1318	MGCS36089_02158			-	NAD(P)/FAD-dependent oxidoreductase	102.5	1318
1319	MGCS36089_00606			-	site-specific integrase	101.5	1319
1320	MGCS36089_02912			-	DUF3169 family protein	101.3	1320
1321	MGCS36089_02478			*spiA*	sakacin P immunity protein SpiA	100.8	1321
1322	MGCS36089_02540			-	hypothetical protein	100.0	1322
1323	MGCS36089_00608			-	helix-turn-helix transcriptional regulator	99.8	1323
1324	MGCS36089_01376			*regR*	transcriptional regulator RegR	99.8	1323
1325	MGCS36089_03218		Virulence	*trxR*	TCS DNA-binding response regulator protein TrxR	99.3	1325
1326	MGCS36089_03672			*mutY*	A/G-specific adenine glycosylase MutY	99.0	1326
1327	MGCS36089_01582	Lipo		-	ABC transporter substrate-binding lipoprotein	98.8	1327
1328	MGCS36089_03920	Secreted	Virulence	*fbpB*	cell surface fibronectin binding protein (B). Cell-wall anchoring predicted sortase	98.5	1328
1329	MGCS36089_02226			*asd*	aspartate-semialdehyde dehydrogenase	98.3	1329
1330	MGCS36089_00594			-	hypothetical protein	97.5	1330
1331	MGCS36089_02278			*coaA*	type I pantothenate kinase	97.5	1330
1332	MGCS36089_02924			-	OFA family MFS transporter	97.5	1330
1333	MGCS36089_02736			*paaI*	PaaI family thioesterase	97.3	1333
1334	MGCS36089_02714			-	hypothetical protein	96.5	1334
1335	MGCS36089_03332			*atoD*	butyrate-acetoacetate CoA-transferase alpha	96.5	1334
1336	MGCS36089_02726			-	putative thioesterase	96.3	1336
1337	MGCS36089_00598			-	IS30 family transposase	95.8	1337
1338	MGCS36089_03734			-	OmpR family TCS DNA-binding response regulator	95.3	1338

No.	Locus tag	Signal6P predicted[(1)]	Virulence[(2)]	Gene	Function	RPKMs[(3)]	RANK[(4)]
1339	MGCS36089_03034			*phrB*	PhrB family deoxyribodipyrimidine photolyase	95.0	1339
1340	MGCS36089_01394			*kdgK*	2-dehydro-3-deoxygluconokinase KdgK	94.5	1340
1341	MGCS36089_02092			-	XRE family HTH-type transcriptional regulator	94.5	1340
1342	MGCS36089_03940			-	Bacteria_small_SRP RNA	94.5	1340
1343	MGCS36089_01338			-	RpoE superfamily DNA-directed RNA polymerase	94.3	1343
1344	MGCS36089_00704			-	Cof-type HAD-IIB family hydrolase	93.8	1344
1345	MGCS36089_03890			-	hypothetical protein	93.8	1344
1346	MGCS36089_01328	Lipo		-	DUF4430 domain-containing lipoprotein	93.5	1346
1347	MGCS36089_02066			*dppB*	dipeptide ABC transport system permease protein	93.5	1346
1348	MGCS36089_01230			*rnc*	ribonuclease III Rnc	92.8	1348
1349	MGCS36089_03334			*atoA*	acyl CoA:acetate/3-ketoacid CoA transferase beta	92.5	1349
1350	MGCS36089_01976			*guaC*	guanosine 5'-monophosphate oxidoreductase GuaC	92.3	1350
1351	MGCS36089_03622	Lipo		*htsA*	heme ABC transporter substrate-binding	92.3	1350
1352	MGCS36089_02752			-	resolvase/recombinase family protein	91.8	1352
1353	MGCS36089_02408			-	GatA family amidase	91.3	1353
1354	MGCS36089_00440			-	hypothetical protein	90.8	1354
1355	MGCS36089_00654			-	Txe/YoeB family addiction module toxin	90.8	1354
1356	MGCS36089_03298			-	YIH1 family putative translation regulator	90.8	1354
1357	MGCS36089_03506			*azgA*	AzgA family permease	90.8	1354
1358	MGCS36089_01208			-	acyl dehydratase	90.5	1358
1359	MGCS36089_00418			-	MccC family LD-carboxypeptidase	89.8	1359
1360	MGCS36089_02476			-	DUF2974 domain-containing protein	89.8	1359
1361	MGCS36089_03914			-	DUF6198 family protein	89.8	1359
1362	MGCS36089_00348			-	DUF4651 domain-containing protein	89.5	1362
1363	MGCS36089_01466			*gstA*	GstA superfamily glutathione-dependent	89.5	1362
1364	MGCS36089_04008	Secreted	Virulence	*slo*	secreted cholesterol-dependent cytolysin	89.3	1364
1365	MGCS36089_03204	Lipo		-	putative lipoprotein	88.8	1365
1366	MGCS36089_03626	Secreted	Virulence	*shr*	heme-binding secreted protein Shr. Cell-wall anchoring predicted sortase	88.8	1365
1367	MGCS36089_02938			*rsuA_2*	ribosomal small subunit pseudouridine synthase	88.3	1367
1368	MGCS36089_01400			-	hypothetical protein	88.0	1368
1369	MGCS36089_02318			-	ABC transporter permease	88.0	1368
1370	MGCS36089_01190			-	putative ABC transporter ATP-binding component	87.8	1370
1371	MGCS36089_03892			-	LytTR family transcriptional regulator	87.8	1370
1372	MGCS36089_00410			*gldA*	glycerol dehydrogenase	87.5	1372
1373	MGCS36089_03708			-	PurR family transcriptional regulator	87.0	1373
1374	MGCS36089_01252			*bglF*	PTS beta-glucoside transporter IIBCA component	86.8	1374
1375	MGCS36089_03220		Virulence	*trxS*	TCS sensor histidine kinase TrxS	86.8	1374
1376	MGCS36089_01392			*rpiB*	RpiB/LacA/LacB family sugar-phosphate isomerase	86.5	1376
1377	MGCS36089_01474			*pheA*	chorismate mutase PheA	86.5	1376
1378	MGCS36089_02414			*pyrE*	orotate phosphoribosyltransferase PyrE	86.5	1376
1379	MGCS36089_02522			*csn2*	CRISPR-associated protein Csn2	86.3	1379

No.	Locus tag	Signal6P predicted[(1)]	Virulence[(2)]	Gene	Function	RPKMs[(3)]	RANK[(4)]
1380	MGCS36089_01396			*kgdA*	bifunctional 4-hydroxy-2-oxoglutarate (KHG) aldolase/2-dehydro-3-deoxy-phosphogluconate (KDPG) aldolase KdgA	85.8	1380
1381	MGCS36089_00340			-	CAAX amino terminal protease family membrane	84.8	1381
1382	MGCS36089_01568			-	YbgA family DUF1722 domain-containing protein	84.8	1381
1383	MGCS36089_02926			-	HAD family hydrolase	84.8	1381
1384	MGCS36089_01858			-	XRE family HTH-type transcriptional regulator	84.5	1384
1385	MGCS36089_00996			-	PhnB family glyoxalase/bleomycin	84.0	1385
1386	MGCS36089_00724			*truA*	tRNA pseudouridine(38-40) synthase TruA	83.8	1386
1387	MGCS36089_03568			-	GlsB/YeaQ/YmgE family stress response membrane	83.8	1386
1388	MGCS36089_00842			*dapE*	M20/M25/M40 family metallo-hydrolase,	83.0	1388
1389	MGCS36089_02410			-	IS3 family transposase	83.0	1388
1390	MGCS36089_02320			-	ABC transporter ATP-binding protein LolD-like	82.8	1390
1391	MGCS36089_02150			-	1,4-dihydroxy-2-naphthoate polyprenyltransferase	82.5	1391
1392	MGCS36089_01828			-	type IV toxin-antitoxin system AbiEi family	82.3	1392
1393	MGCS36089_02650			-	hypothetical protein	82.3	1392
1394	MGCS36089_04092			*catE*	catechol-2,3-dioxygenase CatE	82.3	1392
1395	MGCS36089_00278			-	HNH endonuclease	82.0	1395
1396	MGCS36089_02638			*miaA*	tRNA (adenosine(37)-N6)-dimethylallyltransferase	82.0	1395
1397	MGCS36089_03338			*gntT*	GntT family H+/gluconate symporter or related	82.0	1395
1398	MGCS36089_01518	Secreted		-	cell surface extracellular nuclease. Cell-wall anchoring predicted sortase	81.8	1398
1399	MGCS36089_03578			*ppdK*	pyruvate phosphate dikinase PpdK	81.8	1398
1400	MGCS36089_03786			-	hypothetical protein	81.5	1400
1401	MGCS36089_01566			*osmC*	YhfA family OsmC-related salt-stress induced	80.3	1401
1402	MGCS36089_02146			*xerS*	site-specific tyrosine recombinase XerS	80.0	1402
1403	MGCS36089_02798			-	ORF6N domain-containing protein	79.8	1403
1404	MGCS36089_03330			*atoB*	3-ketoacyl-CoA thiolase/acetyl-CoA	79.5	1404
1405	MGCS36089_03922			-	IS30 family transposase	79.5	1404
1406	MGCS36089_01196			*dhaQ*	DhaKLM operon coactivator DhaQ	79.3	1406
1407	MGCS36089_00408			*mipB*	fructose-6-phosphate aldolase MipB	79.0	1407
1408	MGCS36089_00228			-	IS30 family transposase	78.5	1408
1409	MGCS36089_03624	Secreted		*shp*	heme-binding secreted protein Shp.Cell-wall anchoring predicted sortase	78.3	1409
1410	MGCS36089_00412			-	hypothetical protein	77.8	1410
1411	MGCS36089_01826			-	nucleotidyl transferase AbiEii/AbiGii toxin	77.8	1410
1412	MGCS36089_03234			-	hypothetical protein	77.8	1410
1413	MGCS36089_03724			-	Xre family helix-turn-helix transcriptional	77.8	1410
1414	MGCS36089_00460			-	helix-turn-helix transcriptional regulator	77.5	1414
1415	MGCS36089_01206			*gla*	glycerol uptake facilitator protein Gla	77.5	1414
1416	MGCS36089_02104			-	helix-turn-helix domain-containing protein	77.3	1416
1417	MGCS36089_03764			-	hypothetical protein	77.3	1416
1418	MGCS36089_02052			-	SSRC38 RNA	76.3	1418
1419	MGCS36089_03590			-	DUF156 family protein	76.3	1418
1420	MGCS36089_02460	Lipo		-	extracellular solute-binding lipoprotein	75.8	1420

No.	Locus tag	Signal6P predicted[(1)]	Virulence[(2)]	Gene	Function	RPKMs[(3)]	RANK[(4)]
1421	MGCS36089_01814			*traG_1*	type IV secretory system conjugative DNA	75.5	1421
1422	MGCS36089_02526			*cas1_1*	type II CRISPR-associated endonuclease Cas1	75.3	1422
1423	MGCS36089_00766			-	putative metal homeostasis protein	74.8	1423
1424	MGCS36089_03620			*htsB*	heme ABC transporter permease HtsB	74.8	1423
1425	MGCS36089_02800			-	DUF1413 domain-containing protein	74.3	1425
1426	MGCS36089_00746			*lrgA*	antiholin-like murein hydrolase modulator LrgA	73.8	1426
1427	MGCS36089_01928			*maeR*	TCS signal transduction response regulator MaeR	73.8	1426
1428	MGCS36089_03616			-	CydC-related ABC transporter ATPase/permease	73.8	1426
1429	MGCS36089_00012			*pth*	aminoacyl-tRNA hydrolase Pth	73.5	1429
1430	MGCS36089_02480			-	sakacin P family class II bacteriocin	73.3	1430
1431	MGCS36089_02660			-	IS30 family transposase	73.0	1431
1432	MGCS36089_01438	Secreted		*adcA*	zinc ABC transporter secreted substrate-binding	72.8	1432
1433	MGCS36089_01888			-	MobC family plasmid mobilization relaxosome	72.5	1433
1434	MGCS36089_02416			*pyrF*	orotidine-5'-phosphate decarboxylase PyrF	72.5	1433
1435	MGCS36089_01204			*dhaM*	PTS-dependent dihydroxyacetone kinase	72.3	1435
1436	MGCS36089_02050	Secreted	Virulence	*scpA*	cell surface extracellular C5a peptidase ScpA	72.3	1435
1437	MGCS36089_02718			-	DUF4044 domain-containing protein	72.3	1435
1438	MGCS36089_04028	Secreted		*pepD_2*	secreted dipeptidase PepD	72.3	1435
1439	MGCS36089_02162			-	BaeS family TCS histidine kinase sensor	71.8	1439
1440	MGCS36089_04010			*ifs*	nicotine adenine dinucleotide glycohydrolase	71.8	1439
1441	MGCS36089_00458			-	PhoE family phosphoglycerate mutase	71.5	1441
1442	MGCS36089_00274			-	helix-turn-helix transcriptional regulator	70.8	1442
1443	MGCS36089_01840			-	DUF5966 family protein	70.8	1442
1444	MGCS36089_02860			*uxuA*	mannonate dehydratase	70.8	1442
1445	MGCS36089_03336			-	3-hydroxybutyrate dehydrogenase	70.5	1445
1446	MGCS36089_03762			-	HTH cro/C1-type domain-containing protein	69.8	1446
1447	MGCS36089_04196			-	Rgg/GadR/MutR family transcriptional regulator	69.8	1446
1448	MGCS36089_00748			*lrgB*	antiholin-like protein LrgB	69.5	1448
1449	MGCS36089_02858			-	SDR family D-mannonate oxidoreductase	69.5	1448
1450	MGCS36089_00666			-	Cro/CI family transcriptional regulator	69.3	1450
1451	MGCS36089_02090			-	hypothetical protein	68.8	1451
1452	MGCS36089_04208			-	DUF4097 family protein	68.8	1451
1453	MGCS36089_03582			*ccpN*	CcpN family CBS pair domain transcriptional	68.3	1453
1454	MGCS36089_01868			*copZ_1*	copper chaperone CopZ	67.8	1454
1455	MGCS36089_02038			-	SalY superfamily ABC transporter permease	67.8	1454
1456	MGCS36089_01982			*pbuX*	xanthine permease PbuX	67.5	1456
1457	MGCS36089_02082			-	DUF3173 family protein	66.0	1457
1458	MGCS36089_02644			-	glutathione S-transferase N-terminal	66.0	1457
1459	MGCS36089_02152	Lipo		*apbE*	ApbE family thiamine biosynthesis lipoprotein	65.8	1459
1460	MGCS36089_02524			*cas2_1*	CRISPR-associated endoribonuclease Cas2	65.8	1459
1461	MGCS36089_01818			*trbL*	conjugal transfer protein TrbL	65.5	1461
1462	MGCS36089_01370			-	SSRC10 RNA	65.3	1462

No.	Locus tag	Signal6P predicted[(1)]	Virulence[(2)]	Gene	Function	RPKMs[(3)]	RANK[(4)]
1463	MGCS36089_02868			-	beta-D-glucuronidase	65.3	1462
1464	MGCS36089_02928			-	GNAT family N-acetyltransferase	65.3	1462
1465	MGCS36089_01428			-	DUF1003 domain-containing protein	65.0	1465
1466	MGCS36089_04192			-	IS982 family transposase	65.0	1465
1467	MGCS36089_00126			*purH*	bifunctional phosphoribosylaminoimidazolecarboxamide formyltransferase/IMP cyclohydrolase PurH	64.8	1467
1468	MGCS36089_00956			-	DUF4298 domain-containing protein	64.5	1468
1469	MGCS36089_02914			-	transcriptional regulator	64.5	1468
1470	MGCS36089_02102			-	hypothetical protein	64.3	1470
1471	MGCS36089_04108			-	hypothetical protein	63.5	1471
1472	MGCS36089_02378			-	IS1182 family transposase	63.0	1472
1473	MGCS36089_03580			-	kinase/pyrophosphorylase	62.5	1473
1474	MGCS36089_04012	Secreted	Virulence	*nga*	secreted nicotine adenine dinucleotide	62.0	1474
1475	MGCS36089_00452			-	MdlB family ABC transporter ATP-binding/permease	61.8	1475
1476	MGCS36089_01678			-	membrane-associated alkaline phosphatase	61.8	1475
1477	MGCS36089_02654			*glgP*	maltodextrin phosphorylase protein GlgP	61.8	1475
1478	MGCS36089_01816			-	hypothetical protein	61.5	1478
1479	MGCS36089_01886			-	SAG1252 family conjugative relaxosome accessory	61.5	1478
1480	MGCS36089_03642			*scrA*	sucrose-specific PTS fused IIB/IIC/IIA component	61.3	1480
1481	MGCS36089_00448			-	radical SAM protein	61.0	1481
1482	MGCS36089_00628			-	ATP-binding protein	61.0	1481
1483	MGCS36089_01866			*kdpB*	Potassium-transporting ATPase ATP-binding	61.0	1481
1484	MGCS36089_00406			*pflD*	pyruvate formate-lyase protein PflD	60.8	1484
1485	MGCS36089_00524	Secreted	Virulence	*ska*	secreted streptokinase Ska	60.8	1484
1486	MGCS36089_01202			*dhaL*	dihydroxyacetone kinase subunit DhaL	60.5	1486
1487	MGCS36089_01440			-	GntR family transcriptional regulator	60.5	1486
1488	MGCS36089_02870			-	sugar kinase	60.0	1488
1489	MGCS36089_03618			*htsC*	heme ABC transporter ATP-binding protein HtsC	60.0	1488
1490	MGCS36089_02072			-	IS30 family transposase	59.8	1490
1491	MGCS36089_03908			-	MarR family transcriptional regulator	59.8	1490
1492	MGCS36089_03192			-	MarR family winged helix-turn-helix	59.5	1492
1493	MGCS36089_02164			-	OmpR family TCS DNA-binding response regulator	58.3	1493
1494	MGCS36089_01878			*arsR*	cadmium efflux system metalloregulator ArsR/SmtB	58.0	1494
1495	MGCS36089_02100			-	hypothetical protein	58.0	1494
1496	MGCS36089_02652			-	NADPH-dependent FMN reductase	57.3	1496
1497	MGCS36089_02994			-	IS982 family transposase	57.3	1496
1498	MGCS36089_02174			*citC*	citrate lyase ligase CitC	57.0	1498
1499	MGCS36089_02862			*uxaC*	uronate isomerase	57.0	1498
1500	MGCS36089_02462			-	YesN family TCS DNA-binding response regulator	56.8	1500
1501	MGCS36089_02738			-	nucleoside phosphorylase family protein	56.8	1500
1502	MGCS36089_03198			-	hypothetical protein	56.8	1500
1503	MGCS36089_00438			-	bacteriocin immunity protein	56.3	1503
1504	MGCS36089_03044			-	amino acid ABC transporter permease	56.3	1503

No.	Locus tag	Signal6P predicted[1]	Virulence[2]	Gene	Function	RPKMs[3]	RANK[4]
1505	MGCS36089_02322			*tetR*	TetR family transcriptional regulator	56.0	1505
1506	MGCS36089_01250			*bglG_1*	transcriptional antiterminator BglG	55.3	1506
1507	MGCS36089_01852			-	DUF5960 family protein	55.3	1506
1508	MGCS36089_01326			-	ECF transporter S component	55.0	1508
1509	MGCS36089_01890			-	SAG1250 family conjugative relaxase	55.0	1508
1510	MGCS36089_04144			-	Uup family of ABC transporter with duplicated	54.5	1510
1511	MGCS36089_00626			-	SIR2 family protein	53.8	1511
1512	MGCS36089_01838			-	DUF5962 family protein	53.8	1511
1513	MGCS36089_00422			*proX*	prolyl-tRNA synthetase associated	53.5	1513
1514	MGCS36089_01490			*serB*	phosphoserine phosphatase SerB	53.5	1513
1515	MGCS36089_00456			-	aminoglycoside 6-adenylyltransferase	53.0	1515
1516	MGCS36089_01812			-	hypothetical protein	53.0	1515
1517	MGCS36089_02346			-	IS1182 family transposase	53.0	1515
1518	MGCS36089_03760			-	hypothetical protein	53.0	1515
1519	MGCS36089_04186			-	MFS transporter	53.0	1515
1520	MGCS36089_02418			-	PyrR RNA	52.8	1520
1521	MGCS36089_03396			*fsa*	FSA family fructose-6-phosphate aldolase	52.8	1520
1522	MGCS36089_03468		Virulence	-	YSIRK-targeted surface antigen transcriptional	52.8	1520
1523	MGCS36089_04086			-	Spy491738 RNA	52.8	1520
1524	MGCS36089_01978			-	Purine RNA	52.5	1524
1525	MGCS36089_03042			-	amino acid ABC transporter ATP-binding protein	52.5	1524
1526	MGCS36089_03996	Secreted		-	putative secreted protein	52.5	1524
1527	MGCS36089_04206			-	DUF1700 domain-containing protein	52.5	1524
1528	MGCS36089_01834			-	hypothetical protein	52.3	1528
1529	MGCS36089_01832			-	calcium-binding protein	51.8	1529
1530	MGCS36089_00354			*ssb_1*	single-stranded DNA-binding protein Ssb	51.0	1530
1531	MGCS36089_02940			*nagB*	glucosamine-6-phosphate deaminase NagB	51.0	1530
1532	MGCS36089_03842			-	hypothetical protein	51.0	1530
1533	MGCS36089_03138			*arcB*	ornithine carbamoyltransferase ArcB	50.8	1533
1534	MGCS36089_01108			-	IS1182 family transposase	50.3	1534
1535	MGCS36089_00922			-	FMN RNA	50.0	1535
1536	MGCS36089_02528			*cas9*	type II CRISPR RNA-guided endonuclease Cas9	50.0	1535
1537	MGCS36089_03140			-	GNAT family N-acetyltransferase	49.8	1537
1538	MGCS36089_00722			-	aspartate kinase	49.5	1538
1539	MGCS36089_03248			*yncA*	YncA family L-amino acid N-acyltransferase	49.5	1538
1540	MGCS36089_01012	Secreted		*yoaK*	putative secreted YoaK family protein	49.3	1540
1541	MGCS36089_03586			-	CoA-disulfide reductase	49.3	1540
1542	MGCS36089_01470			-	IS1548 family transposase	49.0	1542
1543	MGCS36089_03092			-	hypothetical protein	49.0	1542
1544	MGCS36089_01200			*dhaK*	dihydroxyacetone kinase subunit DhaK	48.8	1544
1545	MGCS36089_01580			*aspB*	aspartate aminotransferase AspB	48.8	1544
1546	MGCS36089_02670			*malA*	maltodextrose utilization protein MalA	48.5	1546

No.	Locus tag	Signal6P predicted[(1)]	Virulence[(2)]	Gene	Function	RPKMs[(3)]	RANK[(4)]
1547	MGCS36089_00624			-	MvaI/BcnI family restriction endonuclease	48.3	1547
1548	MGCS36089_02446			-	HAD hydrolase family protein	48.3	1547
1549	MGCS36089_00478			-	site-specific integrase	48.0	1549
1550	MGCS36089_04004			-	IS982 family transposase	47.8	1550
1551	MGCS36089_00454			-	type II toxin-antitoxin system PemK/MazF family	47.5	1551
1552	MGCS36089_00262			-	FRG domain-containing protein	47.3	1552
1553	MGCS36089_00840	Lipo		-	MetQ/NlpA family ABC transporter	47.3	1552
1554	MGCS36089_01938			*araC*	AraC family transcriptional regulator	47.3	1552
1555	MGCS36089_04084			*treR*	trehalose operon repressor TreR	47.0	1555
1556	MGCS36089_02588	Secreted		*aphA*	secreted acid phosphatase AphA	46.5	1556
1557	MGCS36089_00518	Secreted		-	cell surface UshA family bifunctional. Cell-wall anchoring predicted sortase	46.3	1557
1558	MGCS36089_03142			*arcA*	arginine deiminase ArcA	46.3	1557
1559	MGCS36089_03472	Secreted		*aes*	Aes family secreted acetyl esterase/lipase	46.3	1557
1560	MGCS36089_01264			-	cupin domain-containing protein	46.0	1560
1561	MGCS36089_03106			-	SDR family oxidoreductase	46.0	1560
1562	MGCS36089_04214			-	IS1182 family transposase	46.0	1560
1563	MGCS36089_03132			-	IS110 family transposase	45.8	1563
1564	MGCS36089_03108			-	IS110 family transposase	45.3	1564
1565	MGCS36089_00318			-	DUF1033 domain-containing protein	45.0	1565
1566	MGCS36089_01410			*fbp2*	fructose-bisphosphatase Fbp2	45.0	1565
1567	MGCS36089_03934			*resA*	TlpA family protein disulfide reductase	45.0	1565
1568	MGCS36089_00468			-	hypothetical protein	44.8	1568
1569	MGCS36089_00444			*flaR*	DNA topology modulation protein	44.5	1569
1570	MGCS36089_01980			*xpt*	xanthine phosphoribosyltransferase Xpt	44.5	1569
1571	MGCS36089_00758			-	IS1548 family transposase	44.3	1571
1572	MGCS36089_01324			-	IS110 family transposase	44.3	1571
1573	MGCS36089_01198			*dhaS*	dihydroxyacetone kinase transcriptional	44.0	1573
1574	MGCS36089_02656			*malQ*	4-alpha-glucanotransferase (amylomaltase)	44.0	1573
1575	MGCS36089_01112		Virulence	*silA*	TCS DNA-binding response regulator SilA	43.8	1575
1576	MGCS36089_02464			-	YesM family TCS sensor histidine kinase	43.8	1575
1577	MGCS36089_02712			*pnuC*	nicotinamide riboside transporter PnuC	43.8	1575
1578	MGCS36089_02864			*eda*	Eda family bifunctional keto-hydroxyglutarate-aldolase/keto-deoxy-phosphogluconate aldolase	43.8	1575
1579	MGCS36089_02222			*norG*	PLP-dependent aminotransferase family protein	43.5	1579
1580	MGCS36089_03418			-	ARA1 family aldo/keto reductase	43.5	1579
1581	MGCS36089_00134			*purK*	5-(carboxyamino)imidazole ribonucleotide	43.3	1581
1582	MGCS36089_01864			*copY_1*	copper transport repressor CopY	43.0	1582
1583	MGCS36089_01706	Secreted		*sdrD*	SdrD superfamily cell surface extracellular. Cell-wall anchoring predicted sortase	42.8	1583
1584	MGCS36089_02076			*kefB*	Kef-type K+ transporter membrane component KefB	42.8	1583
1585	MGCS36089_03246			-	ParB-like nuclease domain-containing protein	42.8	1583
1586	MGCS36089_02026	Lipo		-	streptococcal histidine triad (HIT) lipoprotein.	42.5	1586
1587	MGCS36089_03236			*bglB_2*	6-phospho-beta-glucosidase BglB	42.3	1587
1588	MGCS36089_01830	Secreted		-	cell surface extracellular antigen I/II family. Cell-wall anchoring predicted sortase	42.0	1588

No.	Locus tag	Signal6P predicted[1]	Virulence[2]	Gene	Function	RPKMs[3]	RANK[4]
1589	MGCS36089_01894			-	pemK RNA	42.0	1588
1590	MGCS36089_04188			-	arginase family protein	42.0	1588
1591	MGCS36089_01340			-	sigma factor regulator	41.8	1591
1592	MGCS36089_02728			-	MFS transporter	41.8	1591
1593	MGCS36089_04040			-	cspA RNA	41.8	1591
1594	MGCS36089_02024			*lmb_2*	bifunctional metal ABC transporter substrate-binding lipoprotein/laminin-binding lipoprotein Lmb	41.5	1594
1595	MGCS36089_03208			-	hypothetical protein	41.3	1595
1596	MGCS36089_02604			-	hypothetical protein	41.0	1596
1597	MGCS36089_00446			-	GNAT family N-acetyltransferase	40.8	1597
1598	MGCS36089_02036			-	LolD superfamily ABC transporter ATP-binding	40.8	1597
1599	MGCS36089_02160			-	Gx transporter family protein	40.5	1599
1600	MGCS36089_02022			*lmb_1*	bifunctional metal ABC transporter substrate-binding lipoprotein/laminin-binding lipoprotein Lmb	40.0	1600
1601	MGCS36089_03110	Lipo		*rbsB*	D-ribose ABC transporter substrate-binding	39.5	1601
1602	MGCS36089_03328			-	LysR family transcriptional regulator	39.5	1601
1603	MGCS36089_01896			-	DUF3173 domain-containing protein	39.3	1603
1604	MGCS36089_04202			-	NUDIX hydrolase	39.3	1603
1605	MGCS36089_00430			-	ATP-binding cassette domain-containing protein	39.0	1605
1606	MGCS36089_00436			-	Maf family protein	38.8	1606
1607	MGCS36089_01024			-	hypothetical protein	38.8	1606
1608	MGCS36089_03350			*drpA*	DNA processing protein (A) DprA	38.8	1606
1609	MGCS36089_04190			-	radical SAM protein	38.8	1606
1610	MGCS36089_02854			-	BglX family glycosyl hydrolase	38.5	1610
1611	MGCS36089_04044			-	metallo-dependent amidohydrolase	38.5	1610
1612	MGCS36089_00998			-	GloA family glyoxalase/bleomycin	38.3	1612
1613	MGCS36089_03584			-	rhodanese-like domain-containing protein	38.3	1612
1614	MGCS36089_01892			-	Cro/CI family transcriptional regulator	37.3	1614
1615	MGCS36089_02740	Lipo		*nlpA*	MetQ/NlpA family ABC transporter	37.3	1614
1616	MGCS36089_03002			-	Rgg/GadR/MutR family transcriptional regulator	37.0	1616
1617	MGCS36089_01944			*radC*	DNA repair protein RadC	36.5	1617
1618	MGCS36089_03788			*rpsN2*	30S ribosomal S14-2 protein RpsN2	36.5	1617
1619	MGCS36089_04088			-	hypothetical protein	36.5	1617
1620	MGCS36089_00464			-	hypothetical protein	36.3	1620
1621	MGCS36089_00534			-	hypothetical protein	36.3	1620
1622	MGCS36089_02220			*pdxK*	pyridoxamine kinase PdxK	36.3	1620
1623	MGCS36089_03588			-	rhodanese-like domain-containing protein	36.3	1620
1624	MGCS36089_01154			-	hypothetical protein	36.0	1624
1625	MGCS36089_01940			-	NanC-like SGNH/GDSL hydrolase family protein	35.5	1625
1626	MGCS36089_02056			*htpA*	streptococcal histidine triad (HIT)	35.3	1626
1627	MGCS36089_01144	Secreted		-	secreted transglutaminase	35.0	1627
1628	MGCS36089_02034			*acrA*	AcrA superfamily multidrug efflux pump	35.0	1627
1629	MGCS36089_03730			-	OmpR family TCS DNA-binding response regulator	34.8	1629
1630	MGCS36089_00498			*ptsG*	PTS glucose-specific II ABC component	34.5	1630

No.	Locus tag	Signal6P predicted[(1)]	Virulence[(2)]	Gene	Function	RPKMs[(3)]	RANK[(4)]
1631	MGCS36089_04212			-	hypothetical protein	34.3	1631
1632	MGCS36089_01842			-	hypothetical protein	34.0	1632
1633	MGCS36089_03766			-	type IV secretion system DNA-binding	34.0	1632
1634	MGCS36089_00684			-	toxin-antitoxin system, toxin component, Fic	33.5	1634
1635	MGCS36089_01848			-	DUF5945 family protein	33.5	1634
1636	MGCS36089_01862			*cadD_1*	CadD family cadmium resistance transporter	33.5	1634
1637	MGCS36089_03726	Lipo		-	chromosome assembly-related lipoprotein	33.5	1634
1638	MGCS36089_00686			-	hypothetical protein	33.3	1638
1639	MGCS36089_01390			*idnO*	gluconate 5-dehydrogenase IdnO	33.3	1638
1640	MGCS36089_03728			-	BaeS family TCS sensor histidine kinase	33.3	1638
1641	MGCS36089_02218			-	ECF transporter S component	32.8	1641
1642	MGCS36089_02672			*malD*	maltodextrin transport system permease protein	32.8	1641
1643	MGCS36089_01016			*nrdF_1*	ribonucleotide-diphosphate reductase subunit	32.3	1643
1644	MGCS36089_00420			-	hypothetical protein	32.0	1644
1645	MGCS36089_02966	Lipo		*metQ*	methionine uptake ABC transporter	32.0	1644
1646	MGCS36089_03398			-	putative transcriptional regulator	31.8	1646
1647	MGCS36089_03640			*scrK*	fructokinase protein ScrK	31.8	1646
1648	MGCS36089_00466			-	hypothetical protein	31.5	1648
1649	MGCS36089_01846			-	DUF5965 family protein	31.5	1648
1650	MGCS36089_03644			*scrB*	sucrose-6-phosphate hydrolase ScrB	31.5	1648
1651	MGCS36089_00132			*purE*	5-(carboxyamino)imidazole ribonucleotide mutase	31.3	1651
1652	MGCS36089_02032	Lipo		-	ABC transporter protein	31.3	1651
1653	MGCS36089_00532	Secreted	Virulence	*pulA_1*	cell surface pullulanase PulA. Cell-wall anchoring predicted sortase	31.0	1653
1654	MGCS36089_01020			*nrdE_1*	class 1b ribonucleoside-diphosphate reductase	30.8	1654
1655	MGCS36089_01584			-	IS1548 family transposase	30.8	1654
1656	MGCS36089_03238			-	beta-N-acetylglucosaminidase	30.0	1656
1657	MGCS36089_01018			*nrdI_2*	ribonucleotide reductase assembly protein NrdI	29.8	1657
1658	MGCS36089_03926			-	hypothetical protein	29.5	1658
1659	MGCS36089_03970			*ulaG*	L-ascorbate utilization protein (G) UlaG	29.5	1658
1660	MGCS36089_04090			*yybR*	putative HTH-type transcriptional regulator	29.5	1658
1661	MGCS36089_00130			*purD*	phosphoribosylamine--glycine ligase PurD	29.3	1661
1662	MGCS36089_01930			*maeK*	TCS signal transduction sensor histidine kinase	29.3	1661
1663	MGCS36089_01820			-	PrgI family protein	29.0	1663
1664	MGCS36089_02176			*oadA*	oxaloacetate decarboxylase subunit alpha OadA	29.0	1663
1665	MGCS36089_02760			-	MATE family efflux transporter	29.0	1663
1666	MGCS36089_03112			*rbsC*	D-ribose ABC transporter permease RbsC	29.0	1663
1667	MGCS36089_03400			-	FadH2 family uncharacterized NAD(FAD)-dependent	29.0	1663
1668	MGCS36089_03170			*cas4*	CRISPR-associated protein Cas4	28.5	1668
1669	MGCS36089_01116			-	IS1548 family transposase	28.3	1669
1670	MGCS36089_03456			-	IS982 family transposase	28.3	1669
1671	MGCS36089_00282			-	Spy392987 RNA	28.0	1671
1672	MGCS36089_01406			-	transposase	28.0	1671

No.	Locus tag	Signal6P predicted[(1)]	Virulence[(2)]	Gene	Function	RPKMs[(3)]	RANK[(4)]
1673	MGCS36089_02098			-	hypothetical protein	28.0	1671
1674	MGCS36089_03698			-	alpha-glycosidase	28.0	1671
1675	MGCS36089_04210			-	YhgE/Pip domain-containing protein	28.0	1671
1676	MGCS36089_01248			-	Spy490483c RNA	27.8	1676
1677	MGCS36089_01824			-	phage tail tip lysozyme	27.5	1677
1678	MGCS36089_01822			-	AAA family ATPase	27.3	1678
1679	MGCS36089_01152			-	ABC transporter ATP-binding protein	27.0	1679
1680	MGCS36089_03854			*nanK*	N-acetylmannosamine kinase NanK	27.0	1679
1681	MGCS36089_04228			-	IS1548 family transposase	27.0	1679
1682	MGCS36089_02020			-	hypothetical protein. SignalP-6 predicted lipid anchoring signal peptide,	26.8	1682
1683	MGCS36089_03104			-	IS1548 family transposase	26.8	1682
1684	MGCS36089_03240			-	PurR/LacI family transcriptional regulator	26.8	1682
1685	MGCS36089_03988			-	IS1548 family transposase	26.8	1682
1686	MGCS36089_03114			*rbsA*	D-ribose transporter ATPase RbsA	26.3	1686
1687	MGCS36089_03402			*glpF_1*	GlpF family glycerol uptake facilitator	26.3	1686
1688	MGCS36089_04030			*mutT*	8-oxo-dGTP diphosphatase, DNA mismatch repair	26.3	1686
1689	MGCS36089_00462	Secreted		-	cell surface PgrA surface exclusion. Cell-wall anchoring predicted sortase	26.0	1689
1690	MGCS36089_01442			*agaS*	AgaS superfamily sugar isomerase SIS	26.0	1689
1691	MGCS36089_00128	Secreted		-	CHAP domain-containing protein	25.5	1691
1692	MGCS36089_02744			*dcm*	DNA (cytosine-5-)-methyltransferase Dcm	25.5	1691
1693	MGCS36089_02762			-	PEP-utilizing enzyme	25.5	1691
1694	MGCS36089_01304			-	NAD(P)-binding domain-containing protein	25.3	1694
1695	MGCS36089_03294			*comFC*	ComFC family predicted	25.3	1694
1696	MGCS36089_03404			*glpO*	type 1 glycerol-3-phosphate oxidase GlpO	25.3	1694
1697	MGCS36089_00434			-	hypothetical protein	25.0	1697
1698	MGCS36089_01238			-	IS1548 family transposase	25.0	1697
1699	MGCS36089_01808			-	hypothetical protein	25.0	1697
1700	MGCS36089_01844			-	TOPRIM (topoisomerase-primase) domain-containing protein	25.0	1697
1701	MGCS36089_02678	Lipo		*malX*	maltose/maltodextrin-binding lipoprotein MalX	25.0	1697
1702	MGCS36089_00500			*rgfB*	endonuclease/exonuclease/phosphatase family	24.8	1702
1703	MGCS36089_01098			-	hypothetical protein	24.5	1703
1704	MGCS36089_03754			-	hypothetical protein	24.5	1703
1705	MGCS36089_00570			*talA*	transaldolase TalA	24.3	1705
1706	MGCS36089_02968			*metP_2*	methionine uptake ABC transporter permease MetP	24.0	1706
1707	MGCS36089_00792			*dacA_2*	D-alanyl-D-alanine carboxypeptidase DacA	23.8	1707
1708	MGCS36089_02470			-	AgaB family mannose/fructose/N-acetylgalactosamine-specific component IIB	23.8	1707
1709	MGCS36089_02698			*celB_2*	PTS cellobiose transporter IIC subunit CelB	23.8	1707
1710	MGCS36089_03134			*arcT*	M20 family metallodipeptidase ArcT	23.8	1707
1711	MGCS36089_01764	Secreted		-	extracellular cell wall anchored mucin-binding. Cell-wall anchoring predicted sortase	23.5	1711
1712	MGCS36089_03168			*cas1_2*	type I-C CRISPR-associated endonuclease Cas1	23.5	1711
1713	MGCS36089_00124			*vanZ*	VanZ family putative glycopeptide antibiotic	23.3	1713

No.	Locus tag	Signal6P predicted[(1)]	Virulence[(2)]	Gene	Function	RPKMs[(3)]	RANK[(4)]
1714	MGCS36089_02584	Lipo		-	putative lipoprotein	23.3	1713
1715	MGCS36089_00652			-	replication initiation factor domain-containing	23.0	1715
1716	MGCS36089_01810			-	CPBP family intramembrane metalloprotease	23.0	1715
1717	MGCS36089_02466			*manZ*	ManZ family PTS mannose/fructose IID component	23.0	1715
1718	MGCS36089_00268			-	Rep family plasmid replication protein	22.8	1718
1719	MGCS36089_00472			-	FtsK/SpoIIIE domain-containing protein	22.5	1719
1720	MGCS36089_01104			-	hypothetical protein	22.5	1719
1721	MGCS36089_02468			*manY*	ManY family PTS mannose/fructose IIC component	22.5	1719
1722	MGCS36089_02676			*amyB*	cyclomaltodextrinase protein AmyB	22.5	1719
1723	MGCS36089_03172			*cas7*	type I-C CRISPR-associated protein Cas7/Csd2	22.5	1719
1724	MGCS36089_00682			-	lantibiotic transporter	22.3	1724
1725	MGCS36089_01836			-	SNF2-related protein	22.3	1724
1726	MGCS36089_02110			-	Rep family protein	22.3	1724
1727	MGCS36089_02856			-	phosphoglycolate phosphatase	22.0	1727
1728	MGCS36089_03928			-	hypothetical protein	22.0	1727
1729	MGCS36089_02194			*dprA*	DNA-processing protein DprA	21.8	1729
1730	MGCS36089_02586	Secreted		-	putative secreted protein	21.8	1729
1731	MGCS36089_03866	Lipo		*ugpB_2*	carbohydrate ABC transporter substrate-binding	21.5	1731
1732	MGCS36089_04006	Secreted		-	TrbC/VirB2-related secreted effector protein	21.5	1731
1733	MGCS36089_00676			-	bacteriocin	21.3	1733
1734	MGCS36089_00678			-	lantibiotic dehydratase	21.3	1733
1735	MGCS36089_02970			*metN_2*	methionine uptake ABC transporter ATP-binding	21.3	1733
1736	MGCS36089_00120			*purM*	phosphoribosylformylglycinamidine cyclo-ligase	21.0	1736
1737	MGCS36089_02166			-	ABC transporter permease	21.0	1736
1738	MGCS36089_02674			*malC*	maltodextrin transport system permease protein	21.0	1736
1739	MGCS36089_03118			*rbsK*	ribokinase RbsK	21.0	1736
1740	MGCS36089_03406			*glpK*	glycerol kinase GlpK	21.0	1736
1741	MGCS36089_04176			-	MerR family transcriptional regulator	21.0	1736
1742	MGCS36089_00122			*purN*	phosphoribosylglycinamide formyltransferase	20.8	1742
1743	MGCS36089_00622			-	helix-turn-helix transcriptional regulator	20.8	1742
1744	MGCS36089_03078			-	putative secreted protein. SignalP-6 predicted standard secretion signal	20.8	1742
1745	MGCS36089_03860			-	DUF386 family protein	20.8	1742
1746	MGCS36089_00680			-	lanthionine synthetase C family protein	20.5	1746
1747	MGCS36089_02168			-	ABC transporter ATPase	20.5	1746
1748	MGCS36089_01806			-	arsenate reductase	20.3	1748
1749	MGCS36089_02472			*manX*	ManX family PTS mannose/fructose IIA component	20.3	1748
1750	MGCS36089_03004			-	MFS transporter	20.0	1750
1751	MGCS36089_00838	Lipo		-	MetQ/NlpA family ABC transporter	19.8	1751
1752	MGCS36089_04174			-	replication protein	19.8	1751
1753	MGCS36089_00144			-	quorum-sensing system DWW-type pheromone	19.5	1753
1754	MGCS36089_00270			-	hypothetical protein	19.3	1754
1755	MGCS36089_01850			-	hypothetical protein	19.3	1754

No.	Locus tag	Signal6P predicted[(1)]	Virulence[(2)]	Gene	Function	RPKMs[(3)]	RANK[(4)]
1756	MGCS36089_02702			*celC_2*	PTS cellobiose transporter IIA subunit CelC	19.3	1754
1757	MGCS36089_02746			-	hypothetical protein	19.3	1754
1758	MGCS36089_00118			*purF*	amidophosphoribosyltransferase PurF	19.0	1758
1759	MGCS36089_00142			-	helix-turn-helix domain-containing protein	19.0	1758
1760	MGCS36089_00284			-	NUDIX domain-containing protein	19.0	1758
1761	MGCS36089_02054	Lipo	Virulence	*lmb_3*	bifunctional metal ABC transporter substrate-binding lipoprotein/laminin-binding lipoprotein Lmb.	19.0	1758
1762	MGCS36089_03130			*arcC*	carbamate kinase ArcC	19.0	1758
1763	MGCS36089_00470			-	hypothetical protein	18.8	1763
1764	MGCS36089_00674			-	XRE family transcriptional regulator	18.8	1763
1765	MGCS36089_02704			*celA_2*	PTS cellobiose transporter IIB subunit CelA	18.8	1763
1766	MGCS36089_03166			*cas2_2*	CRISPR-associated endonuclease Cas2	18.8	1763
1767	MGCS36089_03888			-	MefA-related MFS transporter	18.8	1763
1768	MGCS36089_04172			-	hypothetical protein	18.8	1763
1769	MGCS36089_02700			-	DUF3284 domain-containing protein	18.5	1769
1770	MGCS36089_03116			*rbsD*	D-ribose pyranase RbsD	18.5	1769
1771	MGCS36089_03136			*arcD*	arginine/ornithine antiporter protein ArcD	18.5	1769
1772	MGCS36089_03176			*cas5*	type I-C CRISPR-associated protein Cas5	18.5	1769
1773	MGCS36089_03512			-	SSRC34_1 RNA	18.5	1769
1774	MGCS36089_02706			*bglG_2*	transcription antiterminator BglG	18.3	1774
1775	MGCS36089_03868			*nanE*	N-acetylmannosamine-6-phosphate 2-epimerase	18.3	1774
1776	MGCS36089_02590			-	hypothetical protein	18.0	1776
1777	MGCS36089_03174			*cas8*	type I-C CRISPR-associated protein Cas8c/Csd1	18.0	1776
1778	MGCS36089_03858			-	DUF624 domain-containing protein	18.0	1776
1779	MGCS36089_04170			-	FtsK/SpoIIIE domain-containing protein	18.0	1776
1780	MGCS36089_01802			-	DNA (cytosine-5-)-methyltransferase	17.8	1780
1781	MGCS36089_03120			*rbsR*	ribose transport operon repressor RbsR	17.8	1780
1782	MGCS36089_03670			-	hypothetical protein	17.0	1782
1783	MGCS36089_00660			-	conjugal transfer protein	16.8	1783
1784	MGCS36089_02096			-	hypothetical protein	16.8	1783
1785	MGCS36089_02108			-	hypothetical protein	16.8	1783
1786	MGCS36089_03864			*ugpA_2*	carbohydrate ABC transporter permease UgpA-like	16.8	1783
1787	MGCS36089_00116			*purL*	phosphoribosylformylglycinamidine synthase PurL	16.5	1787
1788	MGCS36089_00662			-	cytoplasmic protein	16.5	1787
1789	MGCS36089_01680			-	PyrR RNA	16.5	1787
1790	MGCS36089_02106			-	hypothetical protein	16.5	1787
1791	MGCS36089_04204			*padR*	PadR family transcriptional regulator	16.5	1787
1792	MGCS36089_00568			-	PTS transporter IIC component UlaA-like protein	16.3	1792
1793	MGCS36089_00416			-	hypothetical protein	16.0	1793
1794	MGCS36089_00474			-	Rep family protein	16.0	1793
1795	MGCS36089_01520			-	preQ1-II RNA	16.0	1793
1796	MGCS36089_03856			*nanA*	N-acetylneuraminate lyase	16.0	1793
1797	MGCS36089_02074			*femX*	FemABX-like family peptidoglycan interpeptide	15.8	1797

No.	Locus tag	Signal6P predicted[(1)]	Virulence[(2)]	Gene	Function	RPKMs[(3)]	RANK[(4)]
1798	MGCS36089_02710			*bglB_1*	6-phospho-beta-glucosidase BglB	15.8	1797
1799	MGCS36089_02748			-	RepA N-terminal domain-containing protein	15.8	1797
1800	MGCS36089_03862			*ugpE_2*	carbohydrate ABC transporter permease UgpE-like	15.8	1797
1801	MGCS36089_00272			-	hypothetical protein	15.5	1801
1802	MGCS36089_02188			-	IS982 family transposase	15.5	1801
1803	MGCS36089_01270	Lipo		*pstS*	phosphate ABC transporter substrate-binding protein	15.3	1803
1804	MGCS36089_04072			-	AAA family ATPase domain-containing DNA-binding	15.3	1803
1805	MGCS36089_01444			*lacD_1*	tagatose-bisphosphate aldolase LacD-like	15.0	1805
1806	MGCS36089_02200			-	MFS transporter	15.0	1805
1807	MGCS36089_02934			*coiA*	competence protein CoiA	15.0	1805
1808	MGCS36089_01380			*agaD*	PTS transporter	14.8	1808
1809	MGCS36089_02116			-	site-specific integrase	14.8	1808
1810	MGCS36089_04168			-	hypothetical protein	14.5	1810
1811	MGCS36089_01102			-	Blp family class II bacteriocin with	14.3	1811
1812	MGCS36089_00320			*comYA*	competence system type II/IV secretion system	14.0	1812
1813	MGCS36089_01382			*agaW*	PTS transporter	14.0	1812
1814	MGCS36089_03244			*mngB*	alpha-mannosidase MngB	14.0	1812
1815	MGCS36089_00338			-	helix-turn-helix transcriptional regulator	13.8	1815
1816	MGCS36089_01384			*agaV*	PTS transporter	13.8	1815
1817	MGCS36089_02750			-	hypothetical protein	13.8	1815
1818	MGCS36089_03998			-	MdlB family ABC transporter ATP-binding/permease	13.8	1815
1819	MGCS36089_00476			-	DUF771 domain-containing protein	13.3	1819
1820	MGCS36089_00566			-	PTS transporter IIB component UlaB-like protein	13.3	1819
1821	MGCS36089_00672			-	ImmA/IrrE family metallo-endopeptidase	13.3	1819
1822	MGCS36089_01804			-	hypothetical protein	13.0	1822
1823	MGCS36089_03178			*cas3*	CRISPR-associated helicase Cas3	13.0	1822
1824	MGCS36089_01762			-	HTH domain-containing putative transcriptional	12.8	1824
1825	MGCS36089_02764			-	TetR/AcrR family transcriptional regulator	12.8	1824
1826	MGCS36089_03434			*lacD_2*	tagatose-bisphosphate aldolase LacD	12.8	1824
1827	MGCS36089_00720			*comX_1*	competence protein ComX	12.0	1827
1828	MGCS36089_00808			*comX_2*	competence protein ComX	12.0	1827
1829	MGCS36089_01276			*pstB*	phosphate ABC transporter ATP-binding protein	12.0	1827
1830	MGCS36089_00564			-	PTS transporter IIA component UlaC-like protein	11.8	1830
1831	MGCS36089_00664			-	hypothetical protein	11.5	1831
1832	MGCS36089_01138			-	CAAX amino terminal protease family protein	11.5	1831
1833	MGCS36089_03296			*comFA*	ComFA family DNA/RNA helicase	11.5	1831
1834	MGCS36089_04166			-	sigma-70 family RNA polymerase sigma factor	11.5	1831
1835	MGCS36089_01006			*lctO*	L-lactate oxidase LctO	11.3	1835
1836	MGCS36089_01278			*phoU_1*	phosphate signaling complex protein PhoU	11.3	1835
1837	MGCS36089_01386			*ugl*	unsaturated chondroitin disaccharide hydrolase	11.3	1835
1838	MGCS36089_00618			-	transposase	11.0	1838
1839	MGCS36089_01274			*pstA*	phosphate ABC transporter, permease protein	11.0	1838

No.	Locus tag	Signal6P predicted[(1)]	Virulence[(2)]	Gene	Function	RPKMs[(3)]	RANK[(4)]
1840	MGCS36089_02754			-	hypothetical protein	11.0	1838
1841	MGCS36089_03722			-	Xre family helix-turn-helix transcriptional regulator	11.0	1838
1842	MGCS36089_00616			-	bacterial mobilization protein	10.8	1842
1843	MGCS36089_01272			*pstC*	phosphate ABC transporter, permease protein	10.8	1842
1844	MGCS36089_01876			-	DUF5960 family protein	10.8	1842
1845	MGCS36089_00114			*purC*	phosphoribosylaminoimidazolesuccinocarboxamide	10.5	1845
1846	MGCS36089_01456			-	PTS sugar transport IIA subunit	10.3	1846
1847	MGCS36089_02094			-	hypothetical protein	10.3	1846
1848	MGCS36089_00700			-	triose-phosphate isomerase	9.8	1848
1849	MGCS36089_02084			-	hypothetical protein	9.8	1848
1850	MGCS36089_03156			*msrA/B*	Peptide methionine sulfoxide reductase	9.8	1848
1851	MGCS36089_03436			*lacC*	tagatose-6-phosphate kinase LacC	9.8	1848
1852	MGCS36089_00696			*gatB_1*	PTS galactitol transporter IIB component GatB	9.5	1852
1853	MGCS36089_01388			*agaF*	PTS transporter	9.5	1852
1854	MGCS36089_00614			-	DUF5962 domain-containing protein	9.3	1854
1855	MGCS36089_00644			-	conjugal transfer protein	9.3	1854
1856	MGCS36089_00688			-	tyrosine-type site-specific	9.3	1854
1857	MGCS36089_01306			-	ammonium transporter	9.3	1854
1858	MGCS36089_03438			*lacB*	galactose-6-phosphate isomerase subunit LacB	9.3	1854
1859	MGCS36089_00702			-	YjbQ family protein	9.0	1859
1860	MGCS36089_01096			-	hypothetical protein	9.0	1859
1861	MGCS36089_01378			-	oligohyaluronate lyase	9.0	1859
1862	MGCS36089_03442			*sgcC*	galactitol-specific PTS transporter IIB subunit	9.0	1859
1863	MGCS36089_00404			*celB_1*	PTS transporter cellobiose-specific IIC	8.8	1863
1864	MGCS36089_00612			-	phage replisome organizer N-terminal	8.8	1863
1865	MGCS36089_01142			-	hypothetical protein	8.8	1863
1866	MGCS36089_01454			-	PTS sugar transport IID subunit	8.8	1863
1867	MGCS36089_03704			-	carbohydrate ABC transporter permease UgpE-like	8.8	1863
1868	MGCS36089_00402			*celA_1*	PTS transporter cellobiose-specific IIB	8.5	1868
1869	MGCS36089_00648			-	conjugal transfer protein	8.5	1868
1870	MGCS36089_01448			*bga*	beta-galactosidase Bga	8.5	1868
1871	MGCS36089_01452			-	PTS sugar transport IIC subunit	8.5	1868
1872	MGCS36089_03232			-	COG3537 superfamily putative	8.5	1868
1873	MGCS36089_03440			*lacA*	galactose-6-phosphate isomerase subunit LacA	8.5	1868
1874	MGCS36089_00694			*gatC_1*	PTS galactitol transporter IIC component GatC	8.3	1874
1875	MGCS36089_02114			-	DUF3173 family protein	8.3	1874
1876	MGCS36089_02170			-	S41 family peptidase	8.3	1874
1877	MGCS36089_03242			-	metal-independent alpha-mannosidase	8.3	1874
1878	MGCS36089_04184			-	parvulin-like peptidyl-prolyl isomerase	8.3	1874
1879	MGCS36089_00658			-	FtsK/SpoIIIE domain-containing protein	8.0	1879
1880	MGCS36089_01004			*lctP*	L-lactate permease LctP	8.0	1879
1881	MGCS36089_02530			-	tracrRNA RNA	8.0	1879

No.	Locus tag	Signal6P predicted[(1)]	Virulence[(2)]	Gene	Function	RPKMs[(3)]	RANK[(4)]
1882	MGCS36089_02878	Lipo	Virulence	*slr*	InlA-like streptococcal leucine rich lipoprotein	8.0	1879
1883	MGCS36089_03444			*sgaB*	galactitol-specific PTS transporter IIC subunit	8.0	1879
1884	MGCS36089_03850			-	cysteine hydrolase	8.0	1879
1885	MGCS36089_00646			-	conjugal transfer protein	7.8	1885
1886	MGCS36089_00650			-	putative conjugal transfer protein	7.8	1885
1887	MGCS36089_01132			-	putative bacteriocin with double-glycine leader	7.5	1887
1888	MGCS36089_01136			-	hypothetical protein	7.5	1887
1889	MGCS36089_02112			-	hypothetical protein	7.5	1887
1890	MGCS36089_01872			-	TIGR01906 family membrane protein	7.3	1890
1891	MGCS36089_02086			-	FtsK/SpoIIIE domain-containing protein	7.3	1890
1892	MGCS36089_01026	Secreted		-	hypothetical protein	7.0	1892
1893	MGCS36089_01130			-	lactobin A/cerein 7B family class IIb	7.0	1892
1894	MGCS36089_01450			-	PTS sugar transport IIB subunit	7.0	1892
1895	MGCS36089_03158	Lipo		*tipA*	TlpA family protein disulfide reductase	7.0	1892
1896	MGCS36089_03160			*ccdA*	putative cytochrome c-type biogenesis protein	6.8	1896
1897	MGCS36089_00264			*xerC*	site-specific integrase	6.5	1897
1898	MGCS36089_01128			-	BlpM-like bacteriocin with double-glycine leader	6.5	1897
1899	MGCS36089_01404			-	IS3 family transposase	6.5	1897
1900	MGCS36089_03230			*nagC*	NagC family sugar kinase	6.5	1897
1901	MGCS36089_00266			-	DUF3173 domain-containing protein	6.3	1901
1902	MGCS36089_01934			*maeE*	NAD-dependent malic enzyme MaeE	6.3	1901
1903	MGCS36089_02088			-	hypothetical protein	6.3	1901
1904	MGCS36089_03706			-	carbohydrate ABC transporter permease UgpA-like	6.3	1901
1905	MGCS36089_03772			-	DUF3173 family protein	6.3	1901
1906	MGCS36089_00610			-	DNA-binding protein	6.0	1906
1907	MGCS36089_01134			-	putative bacteriocin with double-glycine leader	6.0	1906
1908	MGCS36089_03226			*ugpE_1*	glycerol-3-phosphate ABC transporter permease	6.0	1906
1909	MGCS36089_03446			-	PTS sugar transporter subunit IIA	6.0	1906
1910	MGCS36089_01874			-	hypothetical protein	5.8	1910
1911	MGCS36089_01932			*maeP*	Citrate/malate symporter protein MaeP	5.8	1910
1912	MGCS36089_03224	Lipo		*ugpB_1*	glycerol-3-phosphate ABC transporter	5.8	1910
1913	MGCS36089_02594			-	hypothetical protein	5.5	1913
1914	MGCS36089_03770			-	replication protein	5.5	1913
1915	MGCS36089_00640			-	DNA segregation ATPase, conjugal transfer	5.3	1915
1916	MGCS36089_00642			-	conjugal transfer protein	5.3	1915
1917	MGCS36089_03228			*ugpA_1*	sugar ABC transporter permease UgpA	5.3	1915
1918	MGCS36089_04000			-	hypothetical protein	5.3	1915
1919	MGCS36089_00690			-	PRD domain/PTS transporter IIA domain protein	5.0	1919
1920	MGCS36089_02766			*mobC*	plasmid mobilization relaxosome protein MobC	5.0	1919
1921	MGCS36089_00322			*comYB*	competence system type II secretion system	4.8	1921
1922	MGCS36089_00638			-	conjugal transfer protein	4.8	1921
1923	MGCS36089_01350			-	glycosyltransferase family 2 protein	4.8	1921

No.	Locus tag	Signal6P predicted[(1)]	Virulence[(2)]	Gene	Function	RPKMs[(3)]	RANK[(4)]
1924	MGCS36089_02952			*comEC*	DNA internalization-related competence protein	4.8	1921
1925	MGCS36089_01126		Virulence	*silE*	streptococcal invasion locus pheromone	4.5	1925
1926	MGCS36089_02768			-	relaxase	4.5	1925
1927	MGCS36089_02954			*comEA*	DNA uptake competence protein ComEA	4.5	1925
1928	MGCS36089_00400			*celC_1*	PTS transporter cellobiose-specific IIA	4.3	1928
1929	MGCS36089_00632			-	helix-turn-helix domain-containing protein	4.3	1928
1930	MGCS36089_00636			-	putative conjugal transfer protein	4.3	1928
1931	MGCS36089_00972			-	cystathionine beta-synthase (CBS)	4.0	1931
1932	MGCS36089_01236			-	hypothetical protein. CW-Pred predicted sortase A cell-wall anchoring motif, position 247..252	4.0	1931
1933	MGCS36089_01344			-	hypothetical protein	4.0	1931
1934	MGCS36089_00326	Pilin		*comYD*	competence system type II secretion system GspH	3.8	1934
1935	MGCS36089_00634	Secreted		-	CHAP domain-containing protein	3.8	1934
1936	MGCS36089_00968			*livG*	branched-chain amino acid ABC transporter	3.8	1934
1937	MGCS36089_00970			*livF*	branched-chain amino acid ABC transporter	3.5	1937
1938	MGCS36089_01124		Virulence	*silD*	streptococcal invasion locus pheromone secretion	3.5	1937
1939	MGCS36089_02776			*topB*	DNA topoisomerase III TopB	3.5	1937
1940	MGCS36089_00330	Pilin		*comYF*	competence system protein ComYF	3.3	1940
1941	MGCS36089_00966			*livM*	branched-chain amino acid ABC transporter	3.3	1940
1942	MGCS36089_01348			-	glycosyltransferase	3.3	1940
1943	MGCS36089_00328	Pilin		*comYE*	competence system protein ComYE	3.0	1943
1944	MGCS36089_01346			-	hypothetical protein	3.0	1943
1945	MGCS36089_02828			-	NTPase domain-containing protein	3.0	1943
1946	MGCS36089_04056			*hutU*	urocanate hydratase HutU	3.0	1943
1947	MGCS36089_00962	Lipo		*livK*	branched-chain amino acid ABC transporter	2.8	1947
1948	MGCS36089_02756			-	pemK RNA	2.8	1947
1949	MGCS36089_02872			*melB*	MelB family MFS transporter	2.8	1947
1950	MGCS36089_03974			*ulaF*	L-ribulose-5-phosphate 4-epimerase UlaF	2.8	1947
1951	MGCS36089_00964			*livH*	branched-chain amino acid ABC transporter	2.5	1951
1952	MGCS36089_03976			*ulaE*	L-xylulose 5-phosphate 3-epimerase UlaE	2.5	1951
1953	MGCS36089_00324	Pilin		-	competence system prepilin-type N-terminal	2.3	1953
1954	MGCS36089_01280	Secreted		-	hypothetical protein	2.3	1953
1955	MGCS36089_03768			-	hypothetical protein	2.3	1953
1956	MGCS36089_00332			*comYG*	competence system protein ComYG	2.0	1956
1957	MGCS36089_01140			-	hypothetical protein	2.0	1956
1958	MGCS36089_02770			-	conjugative element protein	2.0	1956
1959	MGCS36089_02830			-	RepA N-terminal domain-containing protein	2.0	1956
1960	MGCS36089_03978			*ulaD*	3-keto-L-gulonate 6-phosphate decarboxylase	2.0	1956
1961	MGCS36089_03982			*ulaB*	ascorbate-specific PTS transporter EIIB protein	2.0	1956
1962	MGCS36089_04062			*fhs_2*	formate--tetrahydrofolate ligase Fhs	2.0	1956
1963	MGCS36089_04064			*hutD*	histidine uptake and utilization HutD	2.0	1956
1964	MGCS36089_03980			*ulaC*	ascorbate-specific PTS transporter EIIA protein	1.8	1964
1965	MGCS36089_03984			*ulaA*	ascorbate-specific PTS transporter EIIC protein	1.8	1964

No.	Locus tag	Signal6P predicted[(1)]	Virulence[(2)]	Gene	Function	RPKMs[(3)]	RANK[(4)]
1966	MGCS36089_04058			*fctD*	glutamate formiminotransferase FctD	1.8	1964
1967	MGCS36089_04068			*hutH*	histidine ammonia-lyase HutH	1.8	1964
1968	MGCS36089_02826			-	DUF3801 domain-containing protein	1.5	1968
1969	MGCS36089_04066			*potE*	PotE family amino acid transporter	1.5	1968
1970	MGCS36089_02802			-	phage replisome organizer N-terminal	1.3	1970
1971	MGCS36089_02758			-	sigma-70 family RNA polymerase sigma factor like	1.0	1971
1972	MGCS36089_02772			-	DNA methyltransferase	1.0	1971
1973	MGCS36089_02794			*traG_2*	conjugal transfer protein TraG	1.0	1971
1974	MGCS36089_02804			*mobA*	MobA/MobL family protein	1.0	1971
1975	MGCS36089_02824			*virD4*	type IV secretory system conjugative DNA	1.0	1971
1976	MGCS36089_04002			-	hypothetical protein	1.0	1971
1977	MGCS36089_04060			-	formiminotetrahydrofolate cyclodeaminase	1.0	1971
1978	MGCS36089_01120		Virulence	*silCR*	streptococcal invasion locus auto-inducing pheromone peptide SilCR	0.8	1978
1979	MGCS36089_01122			*silC*	streptococcal invasion locus signaling peptide	0.8	1978
1980	MGCS36089_02778	Secreted		-	DUF4366 domain-containing predicted secreted	0.8	1978
1981	MGCS36089_02784			*virB4*	type IV secretory pathway component VirB4	0.8	1978
1982	MGCS36089_02786			-	conjugal transfer protein	0.8	1978
1983	MGCS36089_02796			*tnpW*	TnpW family transposon-encoded protein	0.8	1978
1984	MGCS36089_02808			-	sigma-70 family RNA polymerase sigma factor like	0.8	1978
1985	MGCS36089_02780			-	conjugal transfer protein	0.5	1985
1986	MGCS36089_02788			*virB6*	VirB6-like conjugal transfer protein	0.5	1985
1987	MGCS36089_02790			-	Maff2 family protein	0.5	1985
1988	MGCS36089_02792			-	single-stranded DNA-binding protein	0.5	1985
1989	MGCS36089_02782			-	CHAP domain-containing protein	0.3	1989
1990	MGCS36089_02822			-	site-specific serine recombinase family protein	0.3	1989
1991	MGCS36089_02806			-	DUF3847 domain-containing protein	0.0	1991
1992	MGCS36089_02810			-	MdlB family multidrug ABC transporter ATPase and	0.0	1991
1993	MGCS36089_02812			-	MdlB family multidrug ABC transporter ATPase and	0.0	1991
1994	MGCS36089_02814			*ecfA2*	EcfA2 family ECF transporter ATPase	0.0	1991
1995	MGCS36089_02816			*ecfT*	ECF transporter transmembrane protein EcfT	0.0	1991
1996	MGCS36089_02818			-	ECF transporter S component	0.0	1991
1997	MGCS36089_02820			-	TetR/AcrR family transcriptional regulator	0.0	1991

MGCS36089 was grown *in vitro*, in rich media, and in quadruplicate. Genes were ranked by the mean transcript abundance calculated from four replicates, at mid-exponential (ME) growth phase (OD=1.0). Genes are ordered by transcript abundance rank, from highest (rank=1) to lowest.

(1) Genes predicted by SignalP 6.0 to have an export signal sequence.

(2) Virulence refers to putative virulence genes.

(3) RPKMs were assigned by EDGEPro.

(4) Rank refers to the corresponding transcript abundance rank based on RPKMs.

(5) Lipo, exported lipoprotein attached to the cell envelope.

[6] TCS, two-component system.

Table S2D. Ranked MGCS36089 genes during growth *in vitro* at early stationary phase

No.	Locus tag	Signal6P predicted[(1)]	Virulence[(2)]	Gene	Function	RPKMs[(3)]	RANK[(4)]
1	MGCS36089_01500		Virulence	-	sagA RNA	101241.8	1
2	MGCS36089_01498		Virulence	*sagA*	streptolysin S precursor SagA	65244.3	2
3	MGCS36089_03346			-	RNaseP_bact_b RNA	53336.8	3
4	MGCS36089_02374			*ssrA*	transfer-messenger RNA, SsrA	45752.0	4
5	MGCS36089_01342			*tufA*	translation elongation factor Tu protein TufA	41341.5	5
6	MGCS36089_03820		Virulence	*gapA*	glyceraldehyde-3-phosphate dehydrogenase GapA	31610.3	6
7	MGCS36089_00482			*ssrS*	6S RNA	22743.0	7
8	MGCS36089_02336			*yeaQ*	GlsB/YeaQ/YmgE family stress response membrane	17574.5	8
9	MGCS36089_01496		Virulence	*srrG*	streptolysin S small regulatory RNA SrrG	12916.8	9
10	MGCS36089_01494			*eno*	phosphopyruvate hydratase -- enolase protein	12674.8	10
11	MGCS36089_02326			-	Asp23/Gls24 family envelope stress response	12346.8	11
12	MGCS36089_02328			-	CsbD family protein	11183.0	12
13	MGCS36089_02662	Lipo[(5)]		*malE*	maltose/maltodextrin ABC transport system	10488.0	13
14	MGCS36089_02208			-	apolipoprotein A1/A4/E family protein	9921.3	14
15	MGCS36089_02332			-	DUF2273 domain-containing protein	9115.3	15
16	MGCS36089_02210			-	hypothetical protein	9110.0	16
17	MGCS36089_02330			-	Asp23/Gls24 family envelope stress response	8852.3	17
18	MGCS36089_03138			*arcB*	ornithine carbamoyltransferase ArcB	8833.8	18
19	MGCS36089_03140			-	GNAT family N-acetyltransferase	8804.3	19
20	MGCS36089_03142			*arcA*	arginine deiminase ArcA	7847.5	20
21	MGCS36089_02334			*amaP*	alkaline shock response membrane anchor protein	7177.5	21
22	MGCS36089_02212			-	CsbD family protein	7009.0	22
23	MGCS36089_03090			*dps*	DNA-binding starvation protein	6587.8	23
24	MGCS36089_01502		Virulence	*sagB*	streptolysin S biosynthesis protein SagB	6528.3	24
25	MGCS36089_04052			*ahpF*	alkyl hydroperoxide reductase F subunit AhpF	5405.8	25
26	MGCS36089_00754			*fba_2*	fructose-bisphosphate aldolase	4422.8	26
27	MGCS36089_03348			*gpsB*	cell division regulator GpsB	4361.0	27
28	MGCS36089_03134			*arcT*	M20 family metallodipeptidase ArcT	4044.0	28
29	MGCS36089_02900			*ptsH*	PTS transporter phosphocarrier protein PtsH	4010.8	29
30	MGCS36089_00748			*lrgB*	antiholin-like protein LrgB	3586.5	30
31	MGCS36089_03694			*pflB*	formate C-acetyltransferase	3564.5	31
32	MGCS36089_04050			*ahpC*	alkyl hydroperoxide reductase C subunit AhpC	3544.3	32
33	MGCS36089_03136			*arcD*	arginine/ornithine antiporter protein ArcD	3388.8	33
34	MGCS36089_01510		Virulence	*sagF*	streptolysin S biosynthesis protein SagF	3385.5	34
35	MGCS36089_02852	Secreted	Virulence	*spg*	extracellular cell surface IgG-binding streptococcal protein (G) SpG. Cell-wall anchoring predicted sortase	3261.0	35
36	MGCS36089_03292			*raiA*	ribosome-associated translation inhibitor RaiA	3214.0	36
37	MGCS36089_01516		Virulence	*sagI*	streptolysin S export permease protein SagI	3041.8	37
38	MGCS36089_01508		Virulence	*sagE*	streptolysin S self-immunity protein SagE	2939.0	38
39	MGCS36089_03130			*arcC*	carbamate kinase ArcC	2935.0	39
40	MGCS36089_01514		Virulence	*sagH*	streptolysin S export permease protein SagH	2849.3	40

No.	Locus tag	Signal6P predicted(1)	Virulence(2)	Gene	Function	RPKMs(3)	RANK(4)
41	MGCS36089_00194			*rpsZ*	type Z 30S ribosomal S14 protein RpsZ	2806.0	41
42	MGCS36089_01504		Virulence	*sagC*	streptolysin S biosynthesis protein SagC	2750.8	42
43	MGCS36089_03556			*dnaK*	molecular chaperone DnaK	2647.5	43
44	MGCS36089_00192			*rplE*	50S ribosomal L5 protein RplE	2634.8	44
45	MGCS36089_03006			-	DNA-binding protein HU	2628.0	45
46	MGCS36089_01512		Virulence	*sagG*	streptolysin S export protein SagG	2575.5	46
47	MGCS36089_03408	Lipo		-	PepSY domain-containing lipoprotein	2448.3	47
48	MGCS36089_01506		Virulence	*sagD*	streptolysin S biosynthesis protein SagD	2416.0	48
49	MGCS36089_00190			*rplX*	50S ribosomal L24 protein RplX	2335.5	49
50	MGCS36089_00860			*htpX*	zinc metalloprotease HtpX	2226.8	50
51	MGCS36089_01696			-	ABC transporter permease	2189.5	51
52	MGCS36089_02012			*ldh*	L-lactate dehydrogenase Ldh	2153.3	52
53	MGCS36089_00178			*rplV*	50S ribosomal L22 protein RplV	2145.5	53
54	MGCS36089_00858			*lemA*	LemA family protein	2006.8	54
55	MGCS36089_00180			*rpsC*	30S ribosomal S3 protein RpsC	2004.3	55
56	MGCS36089_03822			*fusA*	FusA family elongation factor EF-G	2003.8	56
57	MGCS36089_01694			-	LolD superfamily ABC transporter ATPase	1947.0	57
58	MGCS36089_01698			-	TVP38 superfamily protein	1943.0	58
59	MGCS36089_00182			*rplP*	50S ribosomal L29 protein RplP	1926.5	59
60	MGCS36089_00188			*rplN*	50S ribosomal L14 protein RplN	1921.8	60
61	MGCS36089_02948			*sodA*	superoxide dismutase SodA	1883.5	61
62	MGCS36089_00186			*rpsQ*	30S ribosomal S17 protein RpsQ	1869.0	62
63	MGCS36089_01052	Lipo		*mtsA*	metal ABC transporter substrate-binding	1863.3	63
64	MGCS36089_03356			*pepC*	aminopeptidase (A) PepC	1835.8	64
65	MGCS36089_02008			*fadH2*	FadH2 superfamily FAD-dependent oxidoreductase	1797.5	65
66	MGCS36089_04118			*spxA_2*	transcriptional regulator SpxA	1773.8	66
67	MGCS36089_01542			*atpF*	ATP synthase B subunit AtpF	1759.8	67
68	MGCS36089_01546			*atpA*	ATP synthase alpha chain, AtpA	1741.5	68
69	MGCS36089_01692			-	RND family transporter membrane fusion protein	1715.8	69
70	MGCS36089_02430			*arsC_2*	arsenate reductase ArsC	1701.8	70
71	MGCS36089_03558			*grpE*	heat shock protein/nucleotide exchange factor	1700.0	71
72	MGCS36089_00176			*rpsS*	30S ribosomal S19 protein RpsS	1699.0	72
73	MGCS36089_00198			*rplF*	50S ribosomal L6 protein RplF	1682.0	73
74	MGCS36089_02432			*deoB*	phosphopentomutase DeoB	1659.3	74
75	MGCS36089_02428			*xapA*	XapA family purine-nucleoside phosphorylase	1655.3	75
76	MGCS36089_02448			-	NAD-dependent succinate-semialdehyde	1649.0	76
77	MGCS36089_03598			*uspA*	UspA family nucleotide-binding universal stress	1624.0	77
78	MGCS36089_00218			*rpsK*	30S ribosomal S11 protein RpsK	1595.0	78
79	MGCS36089_00174			*rplB*	50S ribosomal L2 protein RplB	1588.8	79
80	MGCS36089_00156			*adhP*	alcohol dehydrogenase AdhP	1568.0	80
81	MGCS36089_01544			*atpH*	ATP synthase delta subunit AtpH	1509.3	81
82	MGCS36089_01550			*atpD*	ATP synthase beta subunit AtpD	1498.5	82

No.	Locus tag	Signal6P predicted[(1)]	Virulence[(2)]	Gene	Function	RPKMs[(3)]	RANK[(4)]
83	MGCS36089_01552			*atpC*	ATP synthase epsilon subunit AtpC	1491.3	83
84	MGCS36089_00746			*lrgA*	antiholin-like murein hydrolase modulator LrgA	1476.3	84
85	MGCS36089_03132			-	IS110 family transposase	1459.8	85
86	MGCS36089_00158			-	hypothetical protein	1452.3	86
87	MGCS36089_03968			-	diacylglycerol kinase family lipid kinase	1448.5	87
88	MGCS36089_00170			*rplD*	50S ribosomal L4 protein RplD	1441.8	88
89	MGCS36089_00570			*talA*	transaldolase TalA	1441.3	89
90	MGCS36089_00172			*rplW*	50S ribosomal L23 protein RplW	1424.8	90
91	MGCS36089_01540			*atpB*	ATP synthase A subunit AtpB	1394.0	91
92	MGCS36089_02426			*deoD*	DeoD-type purine-nucleoside phosphorylase	1372.5	92
93	MGCS36089_01548			*atpG*	ATP synthase gamma subunit AtpG	1364.3	93
94	MGCS36089_03674			*trxA_2*	thioredoxin TrxA	1360.3	94
95	MGCS36089_00196			*rpsH*	30S ribosomal S8 protein RpsH	1356.5	95
96	MGCS36089_02666			*malG*	maltose/maltodextrin ABC transport system	1345.8	96
97	MGCS36089_02898			*ptsI*	phosphoenolpyruvate--protein phosphotransferase	1344.8	97
98	MGCS36089_00222			*rplQ*	50S ribosomal L17 protein RplQ	1338.8	98
99	MGCS36089_01324			-	IS110 family transposase	1337.8	99
100	MGCS36089_03108			-	IS110 family transposase	1329.3	100
101	MGCS36089_02360			*pyk*	pyruvate kinase Pyk	1327.0	101
102	MGCS36089_00204			*rpmD*	50S ribosomal L30 protein RpmD	1297.3	102
103	MGCS36089_03560			*hrcA*	heat-inducible transcriptional repressor HrcA	1289.3	103
104	MGCS36089_02992			*gpmA*	phosphoglycerate mutase GpmA	1283.5	104
105	MGCS36089_03826			*rpsL*	30S ribosomal S12 protein RpsL	1266.8	105
106	MGCS36089_00220			*rpoA*	DNA-directed RNA polymerase subunit alpha RpoA	1261.0	106
107	MGCS36089_01588			*mscL*	large-conductance mechanosensitive channel	1261.0	106
108	MGCS36089_01786			*clpL*	ATP-dependent Clp protease ATP-binding subunit	1254.8	108
109	MGCS36089_00208			*secY*	preprotein translocase subunit SecY	1250.5	109
110	MGCS36089_00216			*rpsM*	30S ribosomal S13 protein RpsM	1250.3	110
111	MGCS36089_00200			*rplR*	50S ribosomal L18 protein RplR	1248.3	111
112	MGCS36089_02684			*dltB*	D-alanyl-lipoteichoic acid biosynthesis protein	1235.8	112
113	MGCS36089_01794			*rplL*	50S ribosomal L7/L12 protein RplL	1234.8	113
114	MGCS36089_01352			*tpiA*	triose-phosphate isomerase TpiA	1232.5	114
115	MGCS36089_02682			*dltC*	D-alanine--poly(phosphoribitol) ligase subunit	1223.0	115
116	MGCS36089_00224			-	L17DE RNA	1189.3	116
117	MGCS36089_00214			*rpmJ*	50S ribosomal L36 protein RpmJ	1170.3	117
118	MGCS36089_00184			*rpmC*	50S ribosomal L16 protein RpmC	1168.5	118
119	MGCS36089_02686			*dltA*	D-alanine--poly(phosphoribitol) ligase subunit	1168.5	118
120	MGCS36089_03816			*pgk*	phosphoglycerate kinase Pgk	1148.0	120
121	MGCS36089_03460			*srtB*	pilus polymerization class B sortase SrtB	1141.0	121
122	MGCS36089_00154			*adhE*	bifunctional acetaldehyde-CoA/alcohol dehydrogenase AdhE	1136.3	122
123	MGCS36089_00896			*yccA*	YccA family protein	1121.5	123
124	MGCS36089_03452			*copA*	copper-exporting ATPase CopA	1118.3	124

No.	Locus tag	Signal6P predicted[(1)]	Virulence[(2)]	Gene	Function	RPKMs[(3)]	RANK[(4)]
125	MGCS36089_01478			*rplS*	50S ribosomal L19 protein RpsL	1116.3	125
126	MGCS36089_01796			-	rli38 RNA	1111.5	126
127	MGCS36089_00202			*rpsE*	30S ribosomal S5 protein RpsE	1096.8	127
128	MGCS36089_02270			*cdd*	cytidine deaminase Cdd	1089.3	128
129	MGCS36089_00168			*rplC*	50S ribosomal L3 protein RplC	1085.3	129
130	MGCS36089_03442			*sgcC*	galactitol-specific PTS transporter IIB subunit	1085.3	129
131	MGCS36089_03824			*rpsG*	30S ribosomal S7 protein RpsG	1079.0	131
132	MGCS36089_03458	Secreted		-	pilus ancillary/minor protein 2	1072.0	132
133	MGCS36089_03518			*manN*	PTS transporter mannose-specific IID component	1063.8	133
134	MGCS36089_01260			-	SPJ_0845 family protein	1060.5	134
135	MGCS36089_01206			*gla*	glycerol uptake facilitator protein Gla	1058.5	135
136	MGCS36089_01294			-	YtxH domain-containing protein	1058.3	136
137	MGCS36089_03534			*accB*	acetyl-CoA carboxylase biotin carboxyl carrier	1056.0	137
138	MGCS36089_02680			*dltD*	D-alanyl-lipoteichoic acid biosynthesis protein	1054.3	138
139	MGCS36089_02268	Lipo		-	putative nucleoside ABC transporter	1044.0	139
140	MGCS36089_03668			*rpsF*	30S ribosomal S6 protein RpsF	1040.5	140
141	MGCS36089_01204			*dhaM*	PTS-dependent dihydroxyacetone kinase	1036.5	141
142	MGCS36089_04074			*rpsB*	30S ribosomal S2 protein RpsB	1032.0	142
143	MGCS36089_03052			-	DUF1827 family protein	1025.0	143
144	MGCS36089_02664			*malF*	maltose/maltodextrin ABC transport system	1024.8	144
145	MGCS36089_03530			*accC*	acetyl-CoA carboxylase biotin carboxylase	1015.8	145
146	MGCS36089_00588			*rmlB*	23S rRNA (guanosine(2251)-2'-O)-methyltransferase RlmB	1011.0	146
147	MGCS36089_03048			*clpA*	ATP-dependent Clp protease ATP-binding subunit	1010.5	147
148	MGCS36089_00568			-	PTS transporter IIC component UlaA-like protein	1001.8	148
149	MGCS36089_02362			*pfkA*	6-phosphofructokinase PfkA	978.5	149
150	MGCS36089_01626			*ebsA*	EbsA family pore-forming protein	971.8	150
151	MGCS36089_01704			-	KH domain-containing protein	959.0	151
152	MGCS36089_01638			*rplT*	50S ribosomal L20 protein RplT	957.3	152
153	MGCS36089_01668			*prp*	ribosomal-processing cysteine protease Prp	952.5	153
154	MGCS36089_02698			*celB_2*	PTS cellobiose transporter IIC subunit CelB	941.8	154
155	MGCS36089_03932			*pgi*	Pgi family glucose-6-phosphate isomerase	941.8	154
156	MGCS36089_02272			*deoC*	deoxyribose-phosphate aldolase DeoC	934.0	156
157	MGCS36089_00450			-	hypothetical protein	928.5	157
158	MGCS36089_03444			*sgaB*	galactitol-specific PTS transporter IIC subunit	926.8	158
159	MGCS36089_02558			*acoL*	dihydrolipoyl dehydrogenase AcoL	925.3	159
160	MGCS36089_02382			*rpsA*	30S ribosomal S1 protein RpsA	912.5	160
161	MGCS36089_03462	Secreted		-	pilus backbone/major protein	903.0	161
162	MGCS36089_00604			*rpsI*	30S ribosomal S9 protein RpsI	886.0	162
163	MGCS36089_01286			-	PspC domain-containing protein	881.3	163
164	MGCS36089_02702			*celC_2*	PTS cellobiose transporter IIA subunit CelC	874.0	164
165	MGCS36089_03956		Virulence	*perR*	peroxide-responsive transcriptional repressor	871.8	165
166	MGCS36089_01066			*rplA*	50S ribosomal L1 protein RplA	866.8	166

No.	Locus tag	Signal6P predicted[(1)]	Virulence[(2)]	Gene	Function	RPKMs[(3)]	RANK[(4)]
167	MGCS36089_01628			-	LysM peptidoglycan-binding domain-containing	864.3	167
168	MGCS36089_03438			*lacB*	galactose-6-phosphate isomerase subunit LacB	858.0	168
169	MGCS36089_02896			*gapN*	NADP-dependent glyceraldehyde-3-phosphate	852.0	169
170	MGCS36089_02700			-	DUF3284 domain-containing protein	849.0	170
171	MGCS36089_03068			*ftsZ*	cell division protein FtsZ	840.0	171
172	MGCS36089_01792			*rplJ*	50S ribosomal L10 protein RplJ	836.8	172
173	MGCS36089_03548			*fabT*	transcriptional regulatory protein FabT	832.8	173
174	MGCS36089_02704			*celA_2*	PTS cellobiose transporter IIB subunit CelA	827.8	174
175	MGCS36089_03464			*sipA*	signal peptidase I SipA	824.5	175
176	MGCS36089_03516			*manM*	PTS transporter mannose-specific IIC component	823.8	176
177	MGCS36089_03536			*fabF*	3-oxoacyl-[acyl-carrier-protein] synthase	821.0	177
178	MGCS36089_03450			*copZ_2*	copper chaperone CopZ	817.8	178
179	MGCS36089_00590			-	NYN domain-containing protein	808.0	179
180	MGCS36089_03552			*dnaJ*	chaperone protein DnaJ	807.3	180
181	MGCS36089_03060			-	RNA-binding protein	807.0	181
182	MGCS36089_01202			*dhaL*	dihydroxyacetone kinase subunit DhaL	806.8	182
183	MGCS36089_02370			-	ABC transporter permease	799.3	183
184	MGCS36089_03532			*fabZ*	3-hydroxyacyl-ACP dehydratase FabZ	790.0	184
185	MGCS36089_00864		Virulence	*covR*	TCS[(6)] DNA-binding response regulator CovR	781.8	185
186	MGCS36089_03818	Lipo		*lppC*	e(P4) family 5'-nucleotidase lipoprotein	781.0	186
187	MGCS36089_00206			*rplO*	50S ribosomal L15 protein RplO	772.8	187
188	MGCS36089_04076			*tsf*	translation elongation factor Tsf	767.5	188
189	MGCS36089_01966			*eutD*	phosphate acetyltransferase EutD	765.0	189
190	MGCS36089_01458			*rpmE*	50S ribosomal L31 type B protein RpmE	758.3	190
191	MGCS36089_04112			*ruvX*	Holliday junction resolvase RuvX	757.8	191
192	MGCS36089_03546			*fabH*	3-oxoacyl-[acyl-carrier-protein] synthase	756.8	192
193	MGCS36089_00516	Secreted	Virulence	*emm*	cell surface M protein Emm	753.3	193
194	MGCS36089_00562			*rpsO*	30S ribosomal S15 protein RpsO	750.0	194
195	MGCS36089_02466			*manZ*	ManZ family PTS mannose/fructose IID component	750.0	194
196	MGCS36089_04082			*treB*	PTS transporter trehalose-specific EIIBC	748.8	196
197	MGCS36089_00736			*tig*	trigger factor molecular chaperone Tig	748.5	197
198	MGCS36089_03628	Secreted	Virulence	*isp2*	Isp-related CHAP domain-containing immunogenic	745.0	198
199	MGCS36089_04032			*groEL*	chaperonin GroEL	742.0	199
200	MGCS36089_02368			-	ABC transporter ATP-binding protein	741.5	200
201	MGCS36089_03454			*copY_2*	DNA-binding copper transport transcriptional	740.5	201
202	MGCS36089_03066			*yggS*	YggS family pyridoxal phosphate-dependent	739.5	202
203	MGCS36089_03436			*lacC*	tagatose-6-phosphate kinase LacC	738.3	203
204	MGCS36089_02118			-	SpF66_s RNA	734.0	204
205	MGCS36089_02286			*pepN*	lysyl aminopeptidase/alanine aminopeptidase	732.0	205
206	MGCS36089_03514			*manL*	PTS transporter mannose-specific IIB & IIA	730.8	206
207	MGCS36089_03418			-	ARA1 family aldo/keto reductase	728.0	207
208	MGCS36089_00602			*rplM*	50S ribosomal L13 protein RplM	726.0	208

No.	Locus tag	Signal6P predicted[(1)]	Virulence[(2)]	Gene	Function	RPKMs[(3)]	RANK[(4)]
209	MGCS36089_02468			*manY*	ManY family PTS mannose/fructose IIC component	721.8	209
210	MGCS36089_03528			*accA*	acetyl-CoA carboxylase, carboxyltransferase beta	709.0	210
211	MGCS36089_03538			*fabG_2*	3-ketoacyl-(acyl-carrier-protein) reductase	709.0	210
212	MGCS36089_04028	Secreted		*pepD_2*	secreted dipeptidase PepD	706.8	212
213	MGCS36089_00316			*rpoC*	DNA-directed RNA polymerase subunit beta' RpoC	702.0	213
214	MGCS36089_01536			*glgA*	glycogen synthase GlgA	701.8	214
215	MGCS36089_03064			*sepF*	cell division protein SepF	701.5	215
216	MGCS36089_03440			*lacA*	galactose-6-phosphate isomerase subunit LacA	700.5	216
217	MGCS36089_01636			*rpmI*	50S ribosomal L35 protein RpmL	699.0	217
218	MGCS36089_03554			-	PfpI family predicted protease/amidase	698.5	218
219	MGCS36089_02612			*rfbA*	glucose-1-phosphate thymidylyltransferase RfbA	698.0	219
220	MGCS36089_03962			*polA*	DNA polymerase I PolA	692.3	220
221	MGCS36089_03666			*ssb_2*	single-stranded DNA-binding protein	689.5	221
222	MGCS36089_02610			*rfbC*	dTDP-4-dehydrorhamnose 3,5-epimerase RfbC	688.5	222
223	MGCS36089_02324			-	GntR family transcriptional regulator	682.3	223
224	MGCS36089_01064			*rplK*	50S ribosomal L11P protein RplK	681.8	224
225	MGCS36089_00364	Secreted		-	secreted pilin backbone/major protein	681.5	225
226	MGCS36089_03650			-	Asp23/Gls24 family envelope stress response	679.3	226
227	MGCS36089_02562			*acoC*	dihydrolipoamide acetyltransferase AcoC	678.0	227
228	MGCS36089_00586	Secreted		-	putative secreted protein	670.8	228
229	MGCS36089_04216			*rpsD*	30S ribosomal S4 protein RpsD	661.0	229
230	MGCS36089_00762			-	DAK2 domain-containing protein	660.5	230
231	MGCS36089_03828	Secreted		*prgA*	surface exclusion domain-containing secreted	659.3	231
232	MGCS36089_02644			-	glutathione S-transferase N-terminal	659.0	232
233	MGCS36089_03058			*divIVA*	cell division protein DivIVA	657.3	233
234	MGCS36089_01156			-	DUF853 domain-containing protein	653.5	234
235	MGCS36089_02564			*acoB*	pyruvate dehydrogenase E1 component beta subunit	651.3	235
236	MGCS36089_03526			*accD*	acetyl-CoA carboxylase carboxyl transferase	650.0	236
237	MGCS36089_03958			*yccU*	YccU family CoA-binding protein	647.5	237
238	MGCS36089_03652			*efp*	translation elongation factor (P) Efp	637.3	238
239	MGCS36089_04158			*rpmGA*	50S ribosomal L33 protein RpmGA	635.0	239
240	MGCS36089_02470			-	AgaB family mannose/fructose/N-acetylgalactosamine-specific component IIB	633.8	240
241	MGCS36089_00166			*rpsJ*	30S ribosomal S10 protein RpsJ	633.0	241
242	MGCS36089_03446			-	PTS sugar transporter subunit IIA	631.0	242
243	MGCS36089_03062			-	YggT family protein	629.3	243
244	MGCS36089_03540			*fabD*	malonyl CoA-acyl carrier protein transacylase	622.5	244
245	MGCS36089_03550			*phaB*	enoyl-CoA hydratase protein PhaB	620.0	245
246	MGCS36089_03960			-	hypothetical protein	605.0	246
247	MGCS36089_01624			*pepT*	peptidase (T) PepT	604.0	247
248	MGCS36089_02224			*nrdD_1*	ATP cone domain-containing protein,	597.5	248
249	MGCS36089_02706			*bglG_2*	transcription antiterminator BglG	597.0	249
250	MGCS36089_00888	Lipo		*yidC_1*	membrane protein insertase lipoprotein YidC	595.0	250

No.	Locus tag	Signal6P predicted(1)	Virulence(2)	Gene	Function	RPKMs(3)	RANK(4)
251	MGCS36089_03664			*rpsR*	30S ribosomal S18 protein RpsR	592.5	251
252	MGCS36089_03466	Secreted		-	pilus ancillary/minor protein 1	591.8	252
253	MGCS36089_00890			*acyP*	acylphosphatase AcyP	590.5	253
254	MGCS36089_01702			*rpsP*	30S ribosomal S16 protein RpsP	587.5	254
255	MGCS36089_02256			*coaC*	phosphopantothenoylcysteine decarboxylase CoaC	587.3	255
256	MGCS36089_02608			*rfbB*	dTDP-glucose 4,6-dehydratase RfbB	581.3	256
257	MGCS36089_02688			*dltX*	teichoic acid D-Ala incorporation-associated	580.0	257
258	MGCS36089_02560			-	hypothetical protein	579.8	258
259	MGCS36089_01532			*glgC*	glucose-1-phosphate adenylyltransferase subunit	579.0	259
260	MGCS36089_02920	Lipo		*prsA*	peptidylprolyl isomerase lipoprotein PrsA	578.0	260
261	MGCS36089_01538			*atpE*	ATP synthase C subunit AtpE	577.5	261
262	MGCS36089_00528			*msmK*	sn-glycerol-3-phosphate ABC transporter	570.8	262
263	MGCS36089_02894			-	PgdA-like putative PG GlcNAc deacetylase	568.8	263
264	MGCS36089_00484			*rarA*	replication-associated recombination protein A	566.3	264
265	MGCS36089_03312			*pknB*	Stk1 family PASTA domain-containing Ser/Thr	554.5	265
266	MGCS36089_02258			*panT*	pantothenic acid transporter PanT	554.0	266
267	MGCS36089_01292			-	COG4768 superfamily YoxC-like protein	547.0	267
268	MGCS36089_01418			*ftsX*	cell division permease-like protein FtsX	546.0	268
269	MGCS36089_01180		Virulence	*ccpA*	catabolite control protein CcpA	541.8	269
270	MGCS36089_00954			*clpP*	ATP-dependent Clp protease proteolytic subunit	540.8	270
271	MGCS36089_01178			*pepQ*	Xaa-Pro dipeptidase protein PepQ	540.8	270
272	MGCS36089_02438			*pepV*	dipeptidase PepV	540.5	272
273	MGCS36089_01150			*coaE*	dephospho-CoA kinase CoaE	537.5	273
274	MGCS36089_01290			*lgt*	prolipoprotein diacylglyceryl transferase Lgt	537.3	274
275	MGCS36089_00894			*rnaY*	RnaY family HD domain-containing protein	535.3	275
276	MGCS36089_03542			*fabK*	enoyl-[acyl-carrier-protein] reductase protein	535.0	276
277	MGCS36089_04114			-	IreB-related regulatory phosphoprotein	535.0	276
278	MGCS36089_01534			*glgD*	glucose-1-phosphate adenylyltransferase subunit	534.3	278
279	MGCS36089_01716			*apbA*	2-dehydropantoate 2-reductase	533.0	279
280	MGCS36089_02434			*rpiA*	ribose-5-phosphate isomerase RpiA	533.0	279
281	MGCS36089_00212			*infA*	translation initiation factor IF-1 protein InfA	529.3	281
282	MGCS36089_03544			*acpP_2*	acyl carrier protein AcpP	528.8	282
283	MGCS36089_04288		Virulence	*htrA*	trypsin-like serine protease HtrA	526.8	283
284	MGCS36089_03342			*mapZ*	MapZ family cell division site-positioning	522.5	284
285	MGCS36089_00104	Secreted		*sibA*	CHAP domain-containing protein/secreted	521.3	285
286	MGCS36089_04034			*groES*	co-chaperone GroES	519.5	286
287	MGCS36089_01176			-	NAD(P)H-dependent oxidoreductase	515.3	287
288	MGCS36089_03596			*alaT*	AlaT family aminotransferase	511.8	288
289	MGCS36089_03394			*tkt*	transketolase Tkt	510.8	289
290	MGCS36089_01666			*rplU*	50S ribosomal L21 protein RplU	509.8	290
291	MGCS36089_01288			*hprK*	HPr(Ser) kinase/phosphatase HprK	509.0	291
292	MGCS36089_01174			*gloA*	lactoylglutathione lyase protein GloA	506.8	292

No.	Locus tag	Signal6P predicted(1)	Virulence(2)	Gene	Function	RPKMs(3)	RANK(4)
293	MGCS36089_00760			-	Asp23/Gls24 family envelope stress response	506.5	293
294	MGCS36089_01670			*rpmA*	50S ribosomal L27 protein RpmA	505.5	294
295	MGCS36089_01574			-	neutral zinc metallopeptidase	505.3	295
296	MGCS36089_01200			*dhaK*	dihydroxyacetone kinase subunit DhaK	501.0	296
297	MGCS36089_01386			*ugl*	unsaturated chondroitin disaccharide hydrolase	500.0	297
298	MGCS36089_03648			*nusB*	transcription termination protein NusB	499.5	298
299	MGCS36089_03434			*lacD_2*	tagatose-bisphosphate aldolase LacD	489.8	299
300	MGCS36089_01416			*ftsE*	cell division ATP-binding protein FtsE	487.5	300
301	MGCS36089_04104			*nrdD_2*	anaerobic ribonucleoside-triphosphate reductase	487.3	301
302	MGCS36089_03148			-	B3/4 domain-containing protein	486.8	302
303	MGCS36089_02142			-	cupin domain-containing protein	486.0	303
304	MGCS36089_01634			*infC*	translation initiation factor InfC	485.8	304
305	MGCS36089_00794	Lipo		*oppA_1*	oligopeptide ABC transporter substrate-binding protein	483.3	305
306	MGCS36089_03690			-	hypothetical protein	480.5	306
307	MGCS36089_00336			*ackA*	acetate kinase AckA	479.5	307
308	MGCS36089_01226		Virulence	*vicK*	TCS signal transduction sensor kinase VicK	479.3	308
309	MGCS36089_00952			*upp*	uracil phosphoribosyltransferase Upp	478.8	309
310	MGCS36089_03110	Lipo		*rbsB*	D-ribose ABC transporter substrate-binding	475.8	310
311	MGCS36089_01630			*cmk*	CMP kinase Cmk	473.3	311
312	MGCS36089_00934			*pflA*	pyruvate formate-lyase activating enzyme PflA	472.5	312
313	MGCS36089_01526			*dagK*	diacylglycerol kinase family lipid kinase	471.3	313
314	MGCS36089_01632			-	L20_leader RNA	467.5	314
315	MGCS36089_03912			*galU*	UTP--glucose-1-phosphate uridylyltransferase	465.5	315
316	MGCS36089_02424			-	LytR family transcriptional regulator	464.3	316
317	MGCS36089_03498			-	chemotaxis protein	463.8	317
318	MGCS36089_03676			-	FAD-containing oxidoreductase	462.3	318
319	MGCS36089_01648			-	YlbF/YmcA family competence regulator	461.5	319
320	MGCS36089_03870			*rpmH*	50S ribosomal L34 protein RpmH	457.3	320
321	MGCS36089_00312			-	Lacto-rpoB RNA	454.5	321
322	MGCS36089_04274			*uup*	Uup family ATPase components of ABC transporters	441.5	322
323	MGCS36089_01956			*ribP*	ribose-phosphate pyrophosphokinase RibP	434.5	323
324	MGCS36089_01592			*rpoD*	RNA polymerase sigma factor RpoD	434.0	324
325	MGCS36089_01054			*mtsB*	metal ABC transporter ATP-binding protein MtsB	430.5	325
326	MGCS36089_02650			-	hypothetical protein	428.3	326
327	MGCS36089_01530			*glgB*	1,4-alpha-glucan branching protein GlgB	425.3	327
328	MGCS36089_01224		Virulence	*vicR*	TCS DNA-binding response regulator VicR	423.8	328
329	MGCS36089_00566			-	PTS transporter IIB component UlaB-like protein	422.5	329
330	MGCS36089_03476			*infB*	translation initiation factor IF-2	422.5	329
331	MGCS36089_04054			*hutI*	imidazolonepropionase HutI	422.3	331
332	MGCS36089_01444			*lacD_1*	tagatose-bisphosphate aldolase LacD-like	422.0	332
333	MGCS36089_00106			*prs*	ribose-phosphate pyrophosphokinase PrsA	420.5	333
334	MGCS36089_04110			-	DUF1292 domain-containing protein	417.3	334

No.	Locus tag	Signal6P predicted[(1)]	Virulence[(2)]	Gene	Function	RPKMs[(3)]	RANK[(4)]
335	MGCS36089_01082			*mycA*	oleate hydratase/myosin-crossreactive antigen	417.0	335
336	MGCS36089_01528			*pulA_2*	type I pullulanase PulA	416.0	336
337	MGCS36089_03396			*fsa*	FSA family fructose-6-phosphate aldolase	415.8	337
338	MGCS36089_01752			*sptS*	SptS-like TCS sensor histidine kinase	415.0	338
339	MGCS36089_03634			*secA*	preprotein translocase subunit SecA	414.5	339
340	MGCS36089_03696			-	ElaA-related predicted N-acetyltransferase	414.0	340
341	MGCS36089_02262			-	putative nucleoside ABC transporter permease	411.5	341
342	MGCS36089_00764			-	SPFH domain-containing protein	407.3	342
343	MGCS36089_02206			-	LCB5 family diacylglycerol lipid kinase	406.3	343
344	MGCS36089_02356			*glmS*	glutamine--fructose-6-phosphate transaminase	405.0	344
345	MGCS36089_00390			*purA*	adenylosuccinate synthase PurA	401.5	345
346	MGCS36089_03810			*glnA*	glutamine synthetase GlnA	399.8	346
347	MGCS36089_02280			*rpsT*	30S ribosomal S20 protein RpsT	399.0	347
348	MGCS36089_04238			*mnmA*	tRNA 2-thiouridine(34) synthase MnmA	396.0	348
349	MGCS36089_01380			*agaD*	PTS transporter	392.8	349
350	MGCS36089_02566			*acoA*	Pyruvate dehydrogenase E1 component alpha	391.3	350
351	MGCS36089_03082			*typA*	translational GTPase TypA	389.8	351
352	MGCS36089_02542			*glmM*	phosphoglucosamine mutase GlmM	388.0	352
353	MGCS36089_01970			-	Na+ driven multidrug efflux pump	386.3	353
354	MGCS36089_00936			*ppaC*	manganese-dependent inorganic pyrophosphatase	385.3	354
355	MGCS36089_03070			*ftsA*	cell division protein FtsA	383.3	355
356	MGCS36089_02260			*pgmA*	phospho-sugar mutase PgmA	382.3	356
357	MGCS36089_03314			*pppL*	Stp1/IreP family PP2C-type Ser/Thr phosphatase	381.0	357
358	MGCS36089_01954			*nifS_2*	NifS superfamily cysteine desulfurase	380.3	358
359	MGCS36089_02420			-	hypothetical protein	380.3	358
360	MGCS36089_02730			-	DUF1846 domain-containing protein	378.3	360
361	MGCS36089_03050			-	NUDIX hydrolase	368.5	361
362	MGCS36089_03812			*glnR*	glutamine synthetase transcriptional repressor	368.3	362
363	MGCS36089_04122			*recA*	recombinase RecA	366.8	363
364	MGCS36089_01056			*mtsC*	metal ABC transporter permease MtsC	366.5	364
365	MGCS36089_02264			-	putative nucleoside ABC transporter permease	365.8	365
366	MGCS36089_01072			*frr*	ribosome recycling factor Frr	363.5	366
367	MGCS36089_01382			*agaW*	PTS transporter	361.8	367
368	MGCS36089_03524			*serS*	seryl-tRNA synthetase SerS	360.5	368
369	MGCS36089_00874			*der*	ribosome biogenesis GTPase Der	358.0	369
370	MGCS36089_02440			*nfnB*	NfnB family nitroreductase	357.5	370
371	MGCS36089_03304			-	putative bifunctional	355.3	371
372	MGCS36089_00030			*ftsH*	ATP-dependent zinc metalloprotease FtsH	351.8	372
373	MGCS36089_03564			-	LD/DD carboxypeptidase family protein	350.0	373
374	MGCS36089_02306			*spxA_1*	transcriptional regulator SpxA	348.0	374
375	MGCS36089_01090			*dgkA*	diacylglycerol kinase DgkA	347.5	375
376	MGCS36089_00494			*rsmE*	16S rRNA (uracil(1498)-N(3))-methyltransferase	345.8	376

No.	Locus tag	Signal6P predicted[(1)]	Virulence[(2)]	Gene	Function	RPKMs[(3)]	RANK[(4)]
377	MGCS36089_03388			-	hypothetical protein	345.0	377
378	MGCS36089_02552			*lplA_2*	lipoate--protein ligase LplA	344.8	378
379	MGCS36089_02366			*yhcF*	YhcF family transcriptional regulator	344.3	379
380	MGCS36089_02904			*nrdE_2*	class 1b ribonucleoside-diphosphate reductase	343.0	380
381	MGCS36089_02668			*malH*	LacI family transcriptional regulatory protein	341.3	381
382	MGCS36089_02884			-	GAF domain containing protein	340.5	382
383	MGCS36089_03830			*purR*	pur operon repressor PurR	337.5	383
384	MGCS36089_03012			-	DegV family EDD domain-containing protein	337.3	384
385	MGCS36089_02010			-	FolA superfamily dihydrofolate reductase	336.8	385
386	MGCS36089_03302			-	S1 RNA-binding domain-containing protein	336.3	386
387	MGCS36089_01088			*ybeY*	rRNA maturation RNase YbeY	334.8	387
388	MGCS36089_03798			*rnjA_2*	mRNA degradation ribonuclease RnjA	333.8	388
389	MGCS36089_02588	Secreted		*aphA*	secreted acid phosphatase AphA	331.5	389
390	MGCS36089_03910			*gpsA*	NAD(P)H-dependent glycerol-3-phosphate	330.8	390
391	MGCS36089_01186			-	glycosyltransferase	328.8	391
392	MGCS36089_00004			*dnaN*	DNA polymerase III subunit beta protein DnaN	328.0	392
393	MGCS36089_04078			*pepO*	endopeptidase PepO	326.8	393
394	MGCS36089_02254			*coaB*	phosphopantothenate--cysteine ligase CoaB	325.0	394
395	MGCS36089_04142			*argS*	arginine--tRNA synthase ArgS	324.8	395
396	MGCS36089_03838			*rpe*	ribulose-phosphate 3-epimerase Rpe	324.5	396
397	MGCS36089_02028	Secreted		-	DUF1002 domain-containing putative secreted	322.3	397
398	MGCS36089_01590			*dnaG*	DNA primase protein DnaG	321.5	398
399	MGCS36089_01384			*agaV*	PTS transporter	321.0	399
400	MGCS36089_04268			*guaB*	IMP dehydrogenase GuaB	320.0	400
401	MGCS36089_04120			-	hypothetical protein	319.3	401
402	MGCS36089_02266			-	putative nucleoside ABC transporter ATP-binding	318.3	402
403	MGCS36089_01048		Virulence	*mtsR*	metal-dependent transcriptional regulator MtsR	318.0	403
404	MGCS36089_02858			-	SDR family D-mannonate oxidoreductase	314.5	404
405	MGCS36089_03374			*cshB*	DEAD/DEAH box helicase	314.3	405
406	MGCS36089_01414			*prfB*	peptide chain release factor 2 PrfB	312.5	406
407	MGCS36089_01148			*mutM*	DNA-formamidopyrimidine glycosylase MutM	311.5	407
408	MGCS36089_03400			-	FadH2 family uncharacterized NAD(FAD)-dependent	310.0	408
409	MGCS36089_01262			-	NUDIX hydrolase superfamily protein	309.8	409
410	MGCS36089_01284			-	SprT family protein	309.8	409
411	MGCS36089_01308			*lysS*	lysyl-tRNA synthetase LysS	309.0	411
412	MGCS36089_03354			*pbp1A*	bifunctional PG transglycosylase-transpeptidase	308.5	412
413	MGCS36089_01086			-	uracil DNA glycosylase superfamily protein	308.0	413
414	MGCS36089_01594			-	metal-sulfur cluster assembly factor	306.8	414
415	MGCS36089_01398	Secreted	Virulence	*hylB*	secreted hyaluronate lyase HylB	306.5	415
416	MGCS36089_02690			*uvrB*	excinuclease ABC subunit UvrB	306.0	416
417	MGCS36089_01462			*nrnA*	bifunctional oligoribonuclease/PAP phosphatase	303.8	417
418	MGCS36089_02550			-	CobQ-like type 1 glutamine amidotransferase	303.5	418

No.	Locus tag	Signal6P predicted[1]	Virulence[2]	Gene	Function	RPKMs[3]	RANK[4]
419	MGCS36089_00564			-	PTS transporter IIA component UlaC-like protein	303.0	419
420	MGCS36089_01326			-	ECF transporter S component	301.8	420
421	MGCS36089_03112			*rbsC*	D-ribose ABC transporter permease RbsC	301.8	420
422	MGCS36089_02120			*guaA*	glutamine-hydrolyzing GMP synthase	300.5	422
423	MGCS36089_02726			-	putative thioesterase	300.3	423
424	MGCS36089_03572			*gatB_2*	aspartyl-tRNA(Asn) or glutamyl-tRNA(Gln)	300.3	423
425	MGCS36089_00314			*rpoB*	DNA-directed RNA polymerase subunit beta RpoB	300.0	425
426	MGCS36089_03886			*gltX*	glutamate--tRNA ligase	300.0	425
427	MGCS36089_01372			*mgtA*	MgtA superfamily cation-translocating P-type	298.8	427
428	MGCS36089_01700			-	glycerophosphodiester phosphodiesterase	297.8	428
429	MGCS36089_02846			*map*	methionyl aminopeptidase Map	297.8	428
430	MGCS36089_00550			*polC*	DNA polymerase III PolC	295.5	430
431	MGCS36089_01010			-	hypothetical protein	294.8	431
432	MGCS36089_01080	Secreted		*lysM*	LysM peptidoglycan-binding domain-containing	294.8	431
433	MGCS36089_03360			*pncB*	nicotinate phosphoribosyltransferase PncB	294.5	433
434	MGCS36089_04290			*parB*	chromosome partitioning protein ParB	293.3	434
435	MGCS36089_03496			*hit*	HIT family protein	292.8	435
436	MGCS36089_01094			-	Nudix superfamily phosphohydrolase	291.5	436
437	MGCS36089_03738			-	GNAT family acetyltransferase	291.5	436
438	MGCS36089_00302			*adcR*	zinc-dependent MarR family transcriptional	289.5	438
439	MGCS36089_03736	Secreted		-	putative secreted protein	289.5	438
440	MGCS36089_02882			*dnaX*	DNA polymerase III gamma/tau subunit DnaX	288.3	440
441	MGCS36089_00530			*dexB*	glucan 1,6-alpha-glucosidase DexB	288.0	441
442	MGCS36089_03840			*rsgA*	ribosome small subunit-dependent GTPase (A)	287.8	442
443	MGCS36089_01006			*lctO*	L-lactate oxidase LctO	287.3	443
444	MGCS36089_01524			*ligA*	NAD-dependent DNA ligase LigA	287.0	444
445	MGCS36089_01556			*murA_1*	UDP-N-acetylglucosamine 1-carboxyvinyltransferase protein MurA	286.5	445
446	MGCS36089_02282		Virulence	*ciaH*	TCS sensor histidine kinase protein CiaH	286.0	446
447	MGCS36089_00800			*oppD_1*	oligopeptide ABC transporter permease protein	285.5	447
448	MGCS36089_02626			*apt*	adenine phosphoribosyltransferase Apt	285.0	448
449	MGCS36089_03482			*nusA*	transcription termination factor NusA	285.0	448
450	MGCS36089_01244			*ftsY*	signal recognition particle-docking protein	284.0	450
451	MGCS36089_03678			*mutS2*	DNA mismatch repair endonuclease MutS2	283.3	451
452	MGCS36089_02472			*manX*	ManX family PTS mannose/fructose IIA component	283.0	452
453	MGCS36089_02284		Virulence	*ciaR*	TCS DNA-binding response regulator protein CiaR	282.3	453
454	MGCS36089_01340			-	sigma factor regulator	280.5	454
455	MGCS36089_01596			*rmlD*	dTDP-4-dehydrorhamnose reductase protein RmlD	279.8	455
456	MGCS36089_02204		Virulence	*yqfA*	membrane channel forming/hemolysin III protein	279.0	456
457	MGCS36089_03742			-	ABC transporter ATP-binding protein	278.5	457
458	MGCS36089_03008			-	DUF2140 domain-containing protein	278.0	458
459	MGCS36089_03000			*zntA*	ZntA family P-type heavy metal transporter	277.8	459
460	MGCS36089_01948			*rex*	redox-sensing transcriptional repressor Rex	277.5	460

No.	Locus tag	Signal6P predicted[(1)]	Virulence[(2)]	Gene	Function	RPKMs[(3)]	RANK[(4)]
461	MGCS36089_02860			*uxuA*	mannonate dehydratase	277.3	461
462	MGCS36089_03740			-	ABC transporter permease	277.3	461
463	MGCS36089_00576			*cysE*	serine O-acetyltransferase CysE	276.8	463
464	MGCS36089_03964			*proW*	proline/glycine betaine ABC transporter ATPase	276.8	463
465	MGCS36089_01092			*era*	GTPase Era	276.5	465
466	MGCS36089_00906			-	CBS domain-containing protein	276.0	466
467	MGCS36089_01488			*ezrA*	cell division septation ring formation regulator	276.0	466
468	MGCS36089_02696			-	hypothetical protein	276.0	466
469	MGCS36089_02774			-	helix-turn-helix domain-containing putative	275.8	469
470	MGCS36089_02916			*alaS*	alanine--tRNA synthetase AlaS	275.8	469
471	MGCS36089_04236			*marC*	MarC family small neutral amino acid	275.3	471
472	MGCS36089_03114			*rbsA*	D-ribose transporter ATPase RbsA	273.8	472
473	MGCS36089_00152			*oatA*	acetyltransferase OatA	272.8	473
474	MGCS36089_01782			*engB*	ribosome biogenesis GTP-binding protein EngB	272.3	474
475	MGCS36089_04126			-	VOC family protein	271.8	475
476	MGCS36089_03398			-	putative transcriptional regulator	270.0	476
477	MGCS36089_04242			*sdhA*	L-serine dehydratase alpha subunit SdhA	269.8	477
478	MGCS36089_01714			-	PTS transporter subunit IIC	269.5	478
479	MGCS36089_01258			-	permease	267.8	479
480	MGCS36089_03894			-	AIM24 family protein	267.5	480
481	MGCS36089_01034			-	ASCH domain-containing RNA-binding protein	267.3	481
482	MGCS36089_03986			*leuS*	leucine--tRNA synthase LeuS	267.0	482
483	MGCS36089_01750			*sptR*	SptR-like TCS DNA-binding response regulator	266.8	483
484	MGCS36089_02520			-	CRISPR-DR22 RNA	266.0	484
485	MGCS36089_02518			-	CRISPR-DR22 RNA	265.5	485
486	MGCS36089_03180			*valS*	valine--tRNA synthetase ValS	265.3	486
487	MGCS36089_00866		Virulence	*covS*	TCS sensor kinase CovS	265.0	487
488	MGCS36089_02460	Lipo		-	extracellular solute-binding lipoprotein	264.8	488
489	MGCS36089_03490			*cotS*	CotS family thiamine kinase	264.5	489
490	MGCS36089_00574			-	polynucleotide phosphorylase/polyadenylase	264.3	490
491	MGCS36089_03574			*gatA_2*	aspartyl-tRNA(Asn) or glutamyl-tRNA(Gln)	263.8	491
492	MGCS36089_01426			*asnC*	asparaginyl-tRNA synthetase protein AsnC	263.3	492
493	MGCS36089_02474			*yeiH*	YeiH family uncharacterized membrane protein	263.0	493
494	MGCS36089_03010			-	SGNH-hydrolase superfamily of lipases and	263.0	493
495	MGCS36089_01974			*osmF*	OsmF superfamily glycine/betaine transport	262.3	495
496	MGCS36089_00344			*proC*	pyrroline-5-carboxylate reductase ProC	262.0	496
497	MGCS36089_02720			*pepS*	aminopeptidase PepS	260.8	497
498	MGCS36089_02598			-	tetra tricopeptide repeat family protein	260.5	498
499	MGCS36089_04244			-	HAD hydrolase-like protein	260.0	499
500	MGCS36089_04036			*clpC*	ATP-dependent Clp protease ATP-binding subunit	259.8	500
501	MGCS36089_00834			-	YebC/PmpR family DNA-binding transcriptional	259.5	501
502	MGCS36089_02892			*srmB*	SrmB superfamily II DNA and RNA helicase	259.3	502

No.	Locus tag	Signal6P predicted[(1)]	Virulence[(2)]	Gene	Function	RPKMs[(3)]	RANK[(4)]
503	MGCS36089_02844			*brkB*	BrkB family protein	258.8	503
504	MGCS36089_04100			*mviM*	MviM family predicted dehydrogenase	258.0	504
505	MGCS36089_03146			*argR_2*	arginine responsive transcriptional repressor	257.5	505
506	MGCS36089_00730			-	DUF436 family protein	256.3	506
507	MGCS36089_02574			*rnjA_1*	mRNA degradation ribonuclease RnjA	256.0	507
508	MGCS36089_03946			-	metallo-beta-lactamase superfamily protein	256.0	507
509	MGCS36089_03390			*ccmA*	CcmA family multidrug ABC transporter ATPase	254.8	509
510	MGCS36089_00802			*oppF_1*	oligopeptide ABC transporter ATP-binding protein	254.5	510
511	MGCS36089_03402			*glpF_1*	GlpF family glycerol uptake facilitator	254.0	511
512	MGCS36089_03358			*nadE*	ammonia-dependent NAD(+) synthetase NadE	253.8	512
513	MGCS36089_04068			*hutH*	histidine ammonia-lyase HutH	253.5	513
514	MGCS36089_00300			*ipk*	4-diphosphocytidyl-2-C-methyl-D-erythritol	253.3	514
515	MGCS36089_03562			-	FlgJ-related putative peptidoglycan hydrolase	252.8	515
516	MGCS36089_01578			*rexA*	ATP-dependent nuclease A subunit RexA	251.8	516
517	MGCS36089_03448			*glpR*	GlpR family DNA-binding transcriptional	251.8	516
518	MGCS36089_02554			-	DUF4097 family beta strand repeat-containing	251.5	518
519	MGCS36089_01660			*capA*	CapA family protein	250.3	519
520	MGCS36089_03084			*pspE*	PspE family rhodanese-like domain-containing	250.3	519
521	MGCS36089_03566			-	PhoE family broad specificity phosphatase	250.3	519
522	MGCS36089_02932			*pepF_2*	oligoendopeptidase (F) PepF	249.8	522
523	MGCS36089_01256			-	DUF1980 domain-containing protein	249.5	523
524	MGCS36089_03478			-	YlxQ-related RNA-binding protein	249.3	524
525	MGCS36089_03744			-	PLDc N-terminal domain-containing protein	248.8	525
526	MGCS36089_00150			-	hypothetical protein	248.5	526
527	MGCS36089_00816			*nadD*	nicotinate-nucleotide adenylyltransferase NadD	248.5	526
528	MGCS36089_03746			-	Cro/CI family transcriptional regulator	248.5	526
529	MGCS36089_00572			*pnp*	polyribonucleotide nucleotidyltransferase Pnp	248.3	529
530	MGCS36089_03032			-	phospho-sugar mutase	247.0	530
531	MGCS36089_01296			-	DUF3270 domain-containing protein	246.8	531
532	MGCS36089_03056			*ileS*	isoleucine--tRNA synthetase IleS	246.8	531
533	MGCS36089_03322			*rpoZ*	DNA-directed RNA polymerase omega subunit RpoZ	245.5	533
534	MGCS36089_03872			*jag*	RNA-binding protein Jag	245.0	534
535	MGCS36089_00546			*proS*	prolyl-tRNA synthetase ProS	244.8	535
536	MGCS36089_01070			*pyrH*	UMP kinase PyrH	244.5	536
537	MGCS36089_00414			*pgpA*	phosphatidylglycerophosphatase protein PgpA	244.0	537
538	MGCS36089_01396			*kgdA*	bifunctional 4-hydroxy-2-oxoglutarate (KHG) aldolase/2-dehydro-3-deoxy-phosphogluconate (KDPG) aldolase KdgA	244.0	537
539	MGCS36089_03776			*ybaB*	YbaB family DNA-binding protein	244.0	537
540	MGCS36089_01184			-	glycosyltransferase	242.0	540
541	MGCS36089_02862			*uxaC*	uronate isomerase	242.0	540
542	MGCS36089_02138			-	heme response regulator HssR-like putative TCS	241.5	542
543	MGCS36089_02600			*perM*	PerM family predicted purR regulated permease	241.0	543
544	MGCS36089_04062			*fhs_2*	formate--tetrahydrofolate ligase Fhs	240.3	544

No.	Locus tag	Signal6P predicted[(1)]	Virulence[(2)]	Gene	Function	RPKMs[(3)]	RANK[(4)]
545	MGCS36089_03500			-	Cps2a family anionic cell wall polymer	240.0	545
546	MGCS36089_00492			*prmA*	50S ribosomal protein L11 methyltransferase	239.8	546
547	MGCS36089_03264			-	DUF402 domain-containing protein	239.0	547
548	MGCS36089_03952			*tgt*	tRNA guanosine(34) transglycosylase Tgt	238.0	548
549	MGCS36089_02906			*nrdF_2*	class 1b ribonucleoside-diphosphate reductase	237.8	549
550	MGCS36089_01732			*degV_1*	DegV family protein	237.5	550
551	MGCS36089_00862			*yceD*	large ribosomal RNA subunit accumulation protein	237.3	551
552	MGCS36089_01464			-	GNAT family N-acetyltransferase	237.0	552
553	MGCS36089_01388			*agaF*	PTS transporter	235.3	553
554	MGCS36089_02132			-	FAD-binding oxidoreductase	234.5	554
555	MGCS36089_03420			*nagA*	N-acetylglucosamine-6-phosphate deacetylase	234.0	555
556	MGCS36089_01030			*fabG_1*	3-oxoacyl-ACP reductase FabG	233.0	556
557	MGCS36089_03712		Virulence	*cppA*	CppA family putative C3-glycoprotein degrading	233.0	556
558	MGCS36089_04060			-	formiminotetrahydrofolate cyclodeaminase	232.0	558
559	MGCS36089_04116			-	SSRC41 RNA	231.8	559
560	MGCS36089_00304			*adcC*	metal ABC transporter ATP-binding protein AdcC	231.5	560
561	MGCS36089_01558			*epuA*	DNA-directed RNA polymerase beta subunit EpuA	231.0	561
562	MGCS36089_00110			*plsX*	phosphate acyltransferase PlsX	230.8	562
563	MGCS36089_00522			*dtd*	D-tyrosyl-tRNA deacylase Dtd	229.3	563
564	MGCS36089_04224			*rplI*	50S ribosomal L9 protein RplI	229.3	563
565	MGCS36089_01922			*potB*	spermidine putrescine ABC transport system	229.0	565
566	MGCS36089_03914			-	DUF6198 family protein	228.3	566
567	MGCS36089_03074			*murG*	UDP-N-acetylglucosamine--N-acetylmuramyl-(pentapeptide) pyrophosphoryl-undecaprenol N-acetylglucosamine transferase MurG	228.0	567
568	MGCS36089_01778			-	hypothetical protein	227.8	568
569	MGCS36089_00738			*rpoE*	DNA-directed RNA polymerase subunit delta RpoE	227.5	569
570	MGCS36089_00308			*tyrS*	tyrosyl-tRNA synthetase TyrS	227.0	570
571	MGCS36089_00538			*yajC*	preprotein translocase subunit YajC	226.5	571
572	MGCS36089_03832			*cbf*	YhaM family 3'-5' exoribonuclease	226.5	571
573	MGCS36089_00310			*pbp1b*	bifunctional PG transglycosylase-transpeptidase,	225.5	573
574	MGCS36089_01362			*murM*	peptidoglycan lipid II--L-alanine ligase protein	224.8	574
575	MGCS36089_03412			*glyS*	glycine--tRNA ligase beta subunit GlyS	224.8	574
576	MGCS36089_00306			*adcB*	metal ABC transporter permease AdcB	224.5	576
577	MGCS36089_03326			*rny*	ribonuclease (Y) Rny	224.5	576
578	MGCS36089_01972			*opuBA*	OpuBA superfamily glycine/betaine ABC	224.3	578
579	MGCS36089_04058			*fctD*	glutamate formiminotransferase FctD	224.3	578
580	MGCS36089_03632			*acpS*	AcpS family provisional 4'-phosphopantetheinyl	224.0	580
581	MGCS36089_04080			*treC*	trehalose-6-phosphate hydrolase TreC	224.0	580
582	MGCS36089_00486			*pabA*	aminodeoxychorismate/anthranilate synthase	223.5	582
583	MGCS36089_01952			-	DUF1831 domain-containing protein	223.5	582
584	MGCS36089_03600			-	HAD-related haloacid dehalogenase hydrolase	223.0	584
585	MGCS36089_01032			-	DUF3977 family protein	222.8	585
586	MGCS36089_01412			*queG*	epoxyqueuosine reductase QueG	222.5	586

No.	Locus tag	Signal6P predicted[(1)]	Virulence[(2)]	Gene	Function	RPKMs[(3)]	RANK[(4)]
587	MGCS36089_01424			*aspC*	aspartate aminotransferase protein AspC	222.0	587
588	MGCS36089_01586			*rpsU*	30S ribosomal S21 protein RpsU	222.0	587
589	MGCS36089_00872			*dnaI*	primosomal protein DnaI	221.8	589
590	MGCS36089_00904			-	YfcE family metallophosphoesterase	221.3	590
591	MGCS36089_04064			*hutD*	histidine uptake and utilization HutD	220.0	591
592	MGCS36089_00346			*pepA*	glutamyl aminopeptidase PepA	219.8	592
593	MGCS36089_03630			*alr*	alanine racemase Alr	219.5	593
594	MGCS36089_00908			*xerD_1*	site-specific tyrosine recombinase XerD	219.3	594
595	MGCS36089_03488			*trmB*	tRNA (guanosine(46)-N7)-methyltransferase TrmB	218.3	595
596	MGCS36089_00818			*yqeK*	bis(5'-nucleosyl)-tetraphosphatase (symmetrical)	217.8	596
597	MGCS36089_02864			*eda*	Eda family bifunctional keto-hydroxyglutarate-aldolase/keto-deoxy-phosphogluconate aldolase	217.5	597
598	MGCS36089_01486			*gyrB*	DNA topoisomerase ATP-hydrolyzing B subunit	217.3	598
599	MGCS36089_00002			*dnaA*	chromosomal replication initiator protein DnaA	217.0	599
600	MGCS36089_03324			*gmk*	guanylate kinase Gmk	216.5	600
601	MGCS36089_03344			*rlmL*	23S rRNA N2-methylase RlmL	216.3	601
602	MGCS36089_01920			*potA*	spermidine putrescine ABC transport system	215.3	602
603	MGCS36089_03836			*thiN*	thiamine diphosphokinase ThiN	214.5	603
604	MGCS36089_01640			*ltaS*	LTA synthase LtaS	214.0	604
605	MGCS36089_02716			*obgE*	GTPase ObgE	214.0	604
606	MGCS36089_00750			-	alpha/beta hydrolase	213.8	606
607	MGCS36089_00366	Secreted		-	secreted pilin minor/ancillary protein	213.0	607
608	MGCS36089_03072			*ftsQ*	cell division protein FtsQ/DivIB	212.0	608
609	MGCS36089_00982			*rsmI*	16S rRNA (cytidine(1402)-2'-O)-methyltransferase	211.3	609
610	MGCS36089_00784			*sufS*	cysteine desulfurase SufS	211.0	610
611	MGCS36089_01880			*cadA*	cadmium-translocating P-type ATPase CadA	211.0	610
612	MGCS36089_01780			*clpX*	ATP-dependent Clp protease, ATP-binding subunit	210.8	612
613	MGCS36089_03432			-	YadS family trimeric intracellular cation	210.3	613
614	MGCS36089_04066			*potE*	PotE family amino acid transporter	209.8	614
615	MGCS36089_04098			-	putative acetyltransferase	209.8	614
616	MGCS36089_00782			*sufD*	Fe-S cluster assembly protein SufD	209.5	616
617	MGCS36089_01472			*fldA*	flavodoxin FldA	209.0	617
618	MGCS36089_03120			*rbsR*	ribose transport operon repressor RbsR	209.0	617
619	MGCS36089_04246			-	transglycosylase SLT domain-containing protein	208.5	619
620	MGCS36089_01036			-	Gfo/Idh/MocA family oxidoreductase	208.3	620
621	MGCS36089_00112			*acpP_1*	acyl carrier protein AcpP	207.8	621
622	MGCS36089_01950			-	DUF4649 domain-containing protein	207.8	621
623	MGCS36089_02506			-	CRISPR-DR22 RNA	207.5	623
624	MGCS36089_00376			*srtC_3*	class C sortase SrtC	206.8	624
625	MGCS36089_01364			-	sugar-phosphatase	206.8	624
626	MGCS36089_02128			*ffh*	signal recognition particle protein	206.8	624
627	MGCS36089_03116			*rbsD*	D-ribose pyranase RbsD	206.5	627
628	MGCS36089_03430			-	Cof-type HAD-IIB family phosphohydrolase	206.5	627

No.	Locus tag	Signal6P predicted[(1)]	Virulence[(2)]	Gene	Function	RPKMs[(3)]	RANK[(4)]
629	MGCS36089_03834			*rmuC*	DNA recombination protein RmuC	206.3	629
630	MGCS36089_01394			*kdgK*	2-dehydro-3-deoxygluconokinase KdgK	205.8	630
631	MGCS36089_01994			-	Sua5/YciO/YrdC/YwlC family protein ribosome	205.3	631
632	MGCS36089_01996			-	GNAT family N-acetyltransferase	204.3	632
633	MGCS36089_01946			-	gamma-glutamyl-gamma-aminobutyrate hydrolase	204.0	633
634	MGCS36089_03638			*manA*	mannose-6-phosphate isomerase ManA	204.0	633
635	MGCS36089_00796			*oppB_1*	oligopeptide ABC transporter permease protein	203.5	635
636	MGCS36089_01338			-	RpoE superfamily DNA-directed RNA polymerase	203.5	635
637	MGCS36089_00898			*yneF*	YneF family protein	203.3	637
638	MGCS36089_03182			-	AAA family ATPase	203.3	637
639	MGCS36089_00910			*scp1*	segregation/condensation complex subunit (A)	203.0	639
640	MGCS36089_02504			-	CRISPR-DR22 RNA	203.0	639
641	MGCS36089_02290			*ptsB1*	phosphate ABC transporter ATP-binding protein	202.5	641
642	MGCS36089_00378		Virulence	*fbp*	secreted fibronectin-binding protein. SignalP-6 predicted standard secretion signal	202.3	642
643	MGCS36089_04024			*rluA_2*	RluA family pseudouridine synthase	202.0	643
644	MGCS36089_03016			*recN*	DNA repair protein RecN	201.5	644
645	MGCS36089_04222			*dnaC*	replicative DNA helicase DnaC	201.0	645
646	MGCS36089_02502			-	CRISPR-DR22 RNA	200.8	646
647	MGCS36089_03474			*rbfA*	30S ribosome-binding factor RbfA	200.5	647
648	MGCS36089_03966			*proV*	proline/glycine betaine ABC transporter permease	200.3	648
649	MGCS36089_01166			-	cell division protein FtsW-like protein	200.0	649
650	MGCS36089_02058			-	hypothetical protein	199.8	650
651	MGCS36089_00820			-	cysteine hydrolase	199.5	651
652	MGCS36089_04018			*pbp2A*	multimodular transpeptidase-transglycosylase	199.0	652
653	MGCS36089_01332			*pepF_1*	oligoendopeptidase PepF	198.5	653
654	MGCS36089_03404			*glpO*	type 1 glycerol-3-phosphate oxidase GlpO	198.3	654
655	MGCS36089_00798			*oppC_1*	oligopeptide ABC transporter permease protein	198.0	655
656	MGCS36089_00912			*scp2*	segregation/condensation complex subunit (B)	197.5	656
657	MGCS36089_02654			*glgP*	maltodextrin phosphorylase protein GlgP	196.8	657
658	MGCS36089_00148			-	low molecular weight phosphotyrosine protein	196.5	658
659	MGCS36089_00728			-	ECF transporter S component	196.5	658
660	MGCS36089_03480			-	YlxR family putative RNA-binding protein	196.3	660
661	MGCS36089_03682			-	hypothetical protein	196.3	660
662	MGCS36089_03236			*bglB_2*	6-phospho-beta-glucosidase BglB	196.0	662
663	MGCS36089_04128			*tag*	DNA-3-methyladenine glycosylase Tag	196.0	662
664	MGCS36089_02972			-	Pleckstrin homology-like domain-containing	195.5	664
665	MGCS36089_02998			-	Spy1186876 RNA	195.3	665
666	MGCS36089_03118			*rbsK*	ribokinase RbsK	195.3	665
667	MGCS36089_00916			*yidD*	membrane protein insertion efficiency factor	195.0	667
668	MGCS36089_02140			-	putative TCS histidine kinase sensor	195.0	667
669	MGCS36089_02462			-	YesN family TCS DNA-binding response regulator	193.8	669
670	MGCS36089_02694			*glnQ_2*	glutamine transport ATP-binding protein GlnQ	193.5	670

No.	Locus tag	Signal6P predicted[1]	Virulence[2]	Gene	Function	RPKMs[3]	RANK[4]
671	MGCS36089_03680			-	colicin V production family protein	193.5	670
672	MGCS36089_03594			*codY*	CodY family GTP-sensing pleiotropic	192.5	672
673	MGCS36089_00028			-	hypoxanthine-guanine phosphoribosyltransferase	192.0	673
674	MGCS36089_00554			-	NAD(P)H-dependent oxidoreductase	192.0	673
675	MGCS36089_01676			*rluD*	ribosomal large subunit pseudouridine synthase	192.0	673
676	MGCS36089_00726			*thiD*	bifunctional hydroxymethylpyrimidine kinase/phosphomethylpyrimidine kinase PdxK	191.5	676
677	MGCS36089_03186			-	VOC family protein	191.5	676
678	MGCS36089_01360			*murN*	peptidoglycan lipid II-Ala--L-alanine ligase	191.3	678
679	MGCS36089_01910			*folE*	GTP cyclohydrolase I protein FolE	191.3	678
680	MGCS36089_03716			*glpF_2*	glycerol uptake facilitator GlpF	190.5	680
681	MGCS36089_00958			-	YlbF family regulatory protein	190.3	681
682	MGCS36089_00774			*uppP*	undecaprenyl pyrophosphate phosphatase UppP	189.8	682
683	MGCS36089_02602			*mutX*	8-oxo-dGTP diphosphatase MutX	189.5	683
684	MGCS36089_02016			*srtA*	class A sortase SrtA	189.3	684
685	MGCS36089_00948			*murE_1*	UDP-N-acetylmuramoyl-L-alanyl-D-glutamate--L-lysine ligase MurE	189.0	685
686	MGCS36089_01318			*thiT*	energy-coupled thiamine transporter ThiT	189.0	685
687	MGCS36089_03364			*trxB_2*	thioredoxin-disulfide reductase TrxB	189.0	685
688	MGCS36089_00880			*murC*	UDP-N-acetylmuramate--L-alanine ligase MurC	188.8	688
689	MGCS36089_02288			*phoU_2*	phosphate signaling complex protein PhoU	188.5	689
690	MGCS36089_04138			-	YmcA-related cell fate/competence/biofilm	188.0	690
691	MGCS36089_03944			*tadA*	tRNA adenosine(34) deaminase TadA	187.3	691
692	MGCS36089_04140			*argR_3*	arginine repressor ArgR	187.3	691
693	MGCS36089_00498			*ptsG*	PTS glucose-specific II ABC component	187.0	693
694	MGCS36089_00146			*ruvB*	Holliday junction branch migration DNA helicase	186.8	694
695	MGCS36089_01908			*folC*	dihydrofolate synthase FolC	186.8	694
696	MGCS36089_02048	Lipo		-	TlpA-family protein	186.5	696
697	MGCS36089_02508			-	CRISPR-DR22 RNA	186.3	697
698	MGCS36089_02548			*murE_2*	UDP-N-acetylmuramoylalanyl-D-glutamate-2,	186.0	698
699	MGCS36089_03368			-	GlnQ family polar amino acid ABC transporter	186.0	698
700	MGCS36089_00592			-	DegV family protein	185.8	700
701	MGCS36089_01456			-	PTS sugar transport IIA subunit	185.8	700
702	MGCS36089_04156			*rpmF*	50S ribosomal L32 protein RpmF	185.8	700
703	MGCS36089_00358			*dus*	tRNA-dihydrouridine synthase Dus	185.5	703
704	MGCS36089_03662			-	DUF1129 domain-containing protein	185.5	703
705	MGCS36089_02488			*ndk*	nucleoside-diphosphate kinase Ndk. 1212392..1212757. CRISPR with 6 repeat units	185.3	705
706	MGCS36089_02498			-	CRISPR-DR22 RNA	185.3	705
707	MGCS36089_01884			-	DUF4365 family protein	185.0	707
708	MGCS36089_00778			*rgpG*	undecaprenyl/decaprenyl-phosphate alpha-N-acetylglucosaminyl 1-phosphate transferase RgpG	184.8	708
709	MGCS36089_02732			*fetB*	iron export ABC transporter permease subunit	184.8	708
710	MGCS36089_00556			*def*	peptide deformylase Def	184.3	710
711	MGCS36089_02516			-	CRISPR-DR22 RNA	184.0	711
712	MGCS36089_00368			*srtC_1*	class C sortase SrtC	183.5	712

No.	Locus tag	Signal6P predicted[(1)]	Virulence[(2)]	Gene	Function	RPKMs[(3)]	RANK[(4)]
713	MGCS36089_00544			*rseP*	RIP metalloprotease RseP	183.5	712
714	MGCS36089_01912			*folP*	dihydropteroate synthase protein FolP	183.5	712
715	MGCS36089_04096			*nrdG*	anaerobic ribonucleoside-triphosphate reductase	183.5	712
716	MGCS36089_02446			-	HAD hydrolase family protein	183.3	716
717	MGCS36089_03020			*tlyA*	TlyA family RNA methyltransferase	183.3	716
718	MGCS36089_01232			*smc*	chromosome segregation protein Smc	182.3	718
719	MGCS36089_03100			*rlmN*	23S rRNA (adenine(2503)-C(2))-methyltransferase	182.3	718
720	MGCS36089_01882			-	integrase catalytic subunit	182.0	720
721	MGCS36089_02918			-	LURP-one-related family protein	181.5	721
722	MGCS36089_04132			*mdtH*	MdtH-related MFS mutidrug resistance	181.5	721
723	MGCS36089_04256			*rodZ*	cytoskeletal protein RodZ	181.5	721
724	MGCS36089_00902			*rdgB*	RdgB family non-canonical purine NTP	181.3	724
725	MGCS36089_02292			*ptsB2*	phosphate ABC transporter ATP-binding protein	180.8	725
726	MGCS36089_03162			-	CorA family divalent cation transport protein	180.5	726
727	MGCS36089_04154			*hisS*	histidine--tRNA synthase HisS	180.3	727
728	MGCS36089_00870			*dnaB*	replication initiation and membrane attachment	180.0	728
729	MGCS36089_02310			*truB*	tRNA pseudouridine(55) synthase TruB	180.0	728
730	MGCS36089_01722			*fruA*	fructose-specific PTS transporter EIIC component	179.5	730
731	MGCS36089_02658			*malR*	maltose operon transcriptional repressor MalR	179.5	730
732	MGCS36089_00356			-	deoxyadenosine kinase	179.0	732
733	MGCS36089_00994			*arsC_1*	arsenate reductase ArsC	178.8	733
734	MGCS36089_03642			*scrA*	sucrose-specific PTS fused IIB/IIC/IIA component	178.3	734
735	MGCS36089_00980			*yabA*	DNA replication initiation control protein YabA	178.0	735
736	MGCS36089_03502			-	GNAT family N-acetyltransferase	177.8	736
737	MGCS36089_04152			*aspS*	aspartyl-tRNA synthetase	177.8	736
738	MGCS36089_00210			*adk*	adenylate kinase protein Adk	177.5	738
739	MGCS36089_03606			*recG*	ATP-dependent DNA helicase RecG	177.5	738
740	MGCS36089_01158			-	multidrug efflux MFS transporter	177.3	740
741	MGCS36089_00836	Lipo		-	amino acid ABC transporter substrate-binding	177.0	741
742	MGCS36089_03352			*recU*	Holliday junction resolvase RecU	177.0	741
743	MGCS36089_00772	Secreted		-	DUF2207 domain-containing secreted protein	176.8	743
744	MGCS36089_00900			*murI*	glutamate racemase MurI	176.8	743
745	MGCS36089_01084			*phoH*	phosphate starvation-inducible protein PhoH	176.3	745
746	MGCS36089_01924			*potC*	spermidine putrescine ABC transport system	176.3	745
747	MGCS36089_03658			*uvrA*	excinuclease ABC subunit (A) UvrA	176.3	745
748	MGCS36089_01650			*gorA*	glutathione reductase GorA	176.0	748
749	MGCS36089_00884			*mltG*	endolytic transglycosylase MltG	175.3	749
750	MGCS36089_01454			-	PTS sugar transport IID subunit	174.8	750
751	MGCS36089_00914			*rluB*	ribosomal large subunit pseudouridine synthase	174.3	751
752	MGCS36089_02556			-	PadR family transcriptional regulator	174.0	752
753	MGCS36089_01990			*prfA*	peptide chain release factor 1 PrfA	173.8	753
754	MGCS36089_02044	Secreted	Virulence	*isp*	secreted CHAP domain-containing immunogenic	173.3	754

No.	Locus tag	Signal6P predicted(1)	Virulence(2)	Gene	Function	RPKMs(3)	RANK(4)
755	MGCS36089_01664			-	L21_leader RNA	173.0	755
756	MGCS36089_01242			-	Cof-type HAD-IIB family hydrolase	171.8	756
757	MGCS36089_02880			*birA*	bifunctional biotin--[acetyl-CoA-carboxylase]	171.0	757
758	MGCS36089_03484			*rimP*	ribosome maturation factor RimP	171.0	757
759	MGCS36089_01918			*murB*	UDP-N-acetylmuramate dehydrogenase MurB	170.8	759
760	MGCS36089_03604			-	aldo/keto reductase	170.3	760
761	MGCS36089_04056			*hutU*	urocanate hydratase HutU	170.3	760
762	MGCS36089_00786			*sufE*	SUF system NifU family Fe-S cluster assembly	170.0	762
763	MGCS36089_00960			-	YlbG family protein	169.8	763
764	MGCS36089_02172			-	PASTA domain-containing protein	169.8	763
765	MGCS36089_01420			-	MBL fold metallo-hydrolase	168.8	765
766	MGCS36089_01058	Lipo		-	peptidylprolyl isomerase lipoprotein	167.5	766
767	MGCS36089_02080			-	site-specific integrase	167.5	766
768	MGCS36089_02274			*deoA*	pyrimidine-nucleoside phosphorylase DeoA	167.5	766
769	MGCS36089_03046			-	DUF1797 family protein	167.5	766
770	MGCS36089_03184			-	GNAT family N-acetyltransferase	167.5	766
771	MGCS36089_00984			-	DUF5684 domain-containing protein	167.3	771
772	MGCS36089_02308			*ribF*	bifunctional riboflavin kinase/FAD synthetase	167.0	772
773	MGCS36089_01906			*thrB*	ThrB family homoserine kinase	166.5	773
774	MGCS36089_00986			*cutC*	copper homeostasis protein CutC	166.3	774
775	MGCS36089_01446			-	GntR family transcriptional regulator	165.5	775
776	MGCS36089_03144			*fnr*	Crp/Fnr family transcriptional regulator	165.5	775
777	MGCS36089_00938			-	cytoplasmic protein	165.3	777
778	MGCS36089_00520			*relA*	bifunctional (p)ppGpp synthase/hydrolase RelA	165.0	778
779	MGCS36089_00428			-	FAD/NAD(P)-binding protein	164.5	779
780	MGCS36089_02486			*lepA*	translation elongation factor 4 LepA	164.3	780
781	MGCS36089_01832			-	calcium-binding protein	163.8	781
782	MGCS36089_02722			-	CorA family divalent cation transport protein	163.8	781
783	MGCS36089_00488			*pabB*	para-aminobenzoate synthetase	163.5	783
784	MGCS36089_00814			*yhbY*	ribosome assembly RNA-binding protein YhbY	163.5	783
785	MGCS36089_01834			-	hypothetical protein	163.5	783
786	MGCS36089_01044			*macP*	cell wall synthase accessory phosphoprotein	163.0	786
787	MGCS36089_02964			*deaD*	DEAD/DEAH box helicase	163.0	786
788	MGCS36089_00342			-	hypothetical protein	161.8	788
789	MGCS36089_01330			*btuE*	BtuE superfamily glutathione peroxidase	161.8	788
790	MGCS36089_02656			*malQ*	4-alpha-glucanotransferase (amylomaltase)	161.8	788
791	MGCS36089_01654			-	hypothetical protein	161.5	791
792	MGCS36089_00776			*mecA*	negative regulator of genetic competence,	161.3	792
793	MGCS36089_01452			-	PTS sugar transport IIC subunit	161.0	793
794	MGCS36089_03250			-	LLM class flavin-dependent oxidoreductase	160.0	794
795	MGCS36089_01014			*metS*	methionine--tRNA synthase MetS	159.8	795
796	MGCS36089_01214			-	DUF3114 domain-containing protein	159.8	795

No.	Locus tag	Signal6P predicted[(1)]	Virulence[(2)]	Gene	Function	RPKMs[(3)]	RANK[(4)]
797	MGCS36089_02136			*yhhX*	PRK10206 superfamily putative oxidoreductase	159.5	797
798	MGCS36089_03086			*glcK*	glucokinase GlcK	159.5	797
799	MGCS36089_01914			*folQ*	dihydroneopterin aldolase protein FolB	159.3	799
800	MGCS36089_02014			*gyrA*	DNA gyrase subunit A GyrA	159.3	799
801	MGCS36089_03718			*pepX*	PepX family Xaa-Pro dipeptidyl-peptidase	159.3	799
802	MGCS36089_00812			*yqeH*	ribosome biogenesis GTPase YqeH	159.0	802
803	MGCS36089_03378			*pbp2X*	PG transpeptidase class B penicillin-binding	159.0	802
804	MGCS36089_01182			*amyS*	alpha-amylase AmyS	158.5	804
805	MGCS36089_02606			-	Abi family CAAX protease self-immunity protein	158.5	804
806	MGCS36089_02624			*dnaD*	DNA replication protein DnaD	157.0	806
807	MGCS36089_01870			*copB*	copper-exporting ATPase cCopB	156.5	807
808	MGCS36089_01170			*wcaA*	WcaA superfamily glycosyltransferase involved in cell wall biosynthesis	156.3	808
809	MGCS36089_00822			*rsfS*	ribosome silencing factor RsfS	155.8	809
810	MGCS36089_01042			*nudF*	NUDIX hydrolase NudF	155.5	810
811	MGCS36089_00582			*mrnC*	mini-ribonuclease 3 MrnC	155.3	811
812	MGCS36089_03022			*ispA*	IspA family geranylgeranyl pyrophosphate	154.8	812
813	MGCS36089_03382			*mraW*	S-adenosyl-methyltransferase MraW	154.8	812
814	MGCS36089_01336			*ftsW*	cell division protein FtsW	153.8	814
815	MGCS36089_02148			-	NusG domain II-containing protein	153.8	814
816	MGCS36089_03578			*ppdK*	pyruvate phosphate dikinase PpdK	153.0	816
817	MGCS36089_02942			*queA*	S-adenosylmethionine ribosyltransferase-isomerase QueA	152.8	817
818	MGCS36089_01228			*vicX*	zinc-dependent hydrolase protein VicX	152.5	818
819	MGCS36089_02394			-	hypothetical protein	152.5	818
820	MGCS36089_02734			*fetA*	iron export ABC transporter ATP-binding subunit	152.0	820
821	MGCS36089_03316			*rsmB*	16S rRNA (cytosine(967)-C(5))-methyltransferase	152.0	820
822	MGCS36089_03376			*mraY*	phospho-N-acetylmuramoyl-pentapeptide-	152.0	820
823	MGCS36089_04178			-	site-specific integrase	152.0	820
824	MGCS36089_02294			*ptsA*	phosphate ABC transporter permease PstA	151.8	824
825	MGCS36089_00788			*sufB*	Fe-S cluster assembly protein SufB	151.5	825
826	MGCS36089_00926			-	PgpB family membrane-associated phospholipid	151.5	825
827	MGCS36089_03720			-	pyridoxamine 5'-phosphate oxidase family	151.5	825
828	MGCS36089_01748			-	GTP pyrophosphokinase family protein	151.3	828
829	MGCS36089_02464			-	YesM family TCS sensor histidine kinase	151.3	828
830	MGCS36089_03684			*rnhC*	HIII ribonuclease RnhC	151.3	828
831	MGCS36089_03024			*xseB*	exodeoxyribonuclease VII small subunit XseB	151.0	831
832	MGCS36089_00552			-	MarR family transcriptional regulator	150.8	832
833	MGCS36089_02046			-	hypothetical protein	150.5	833
834	MGCS36089_01434			*whiA*	cell division involved DNA-binding protein WhiA	150.3	834
835	MGCS36089_03406			*glpK*	glycerol kinase GlpK	150.3	834
836	MGCS36089_02000			-	nucleoid-associated bacterial family protein	149.8	836
837	MGCS36089_02400			*pyrC*	dihydroorotase PyrC	149.8	836
838	MGCS36089_01998			*glyA*	serine hydroxymethyl transferase GlyA	149.5	838

No.	Locus tag	Signal6P predicted[(1)]	Virulence[(2)]	Gene	Function	RPKMs[(3)]	RANK[(4)]
839	MGCS36089_00882			-	GNAT family N-acetyltransferase	149.0	839
840	MGCS36089_04102			-	hypothetical protein	148.0	840
841	MGCS36089_04130			*ruvA*	Holliday junction ATP-dependent DNA helicase	148.0	840
842	MGCS36089_00886			*greA*	transcription elongation factor GreA	147.8	842
843	MGCS36089_00370			*srtC_2*	class C sortase SrtC	147.5	843
844	MGCS36089_01916			*folK*	2-amino-4-hydroxy-6-hydroxymethyldihydropteridine pyrophosphokinase protein FolK	147.5	843
845	MGCS36089_00542			*cdsA*	phosphatidate cytidylyltransferase CdsA	147.3	845
846	MGCS36089_02534			*nagD*	NagD family hydrolase	147.3	845
847	MGCS36089_03102			*yutD*	YutD family protein of unknown function	147.0	847
848	MGCS36089_03256			*thlA*	thiolase ThlA	146.5	848
849	MGCS36089_02572			*frmB*	FrmB family esterase	146.3	849
850	MGCS36089_01770			*mvaS1*	hydroxymethylglutaryl-CoA reductase protein (1)	146.0	850
851	MGCS36089_03874	Lipo		*yidC_2*	YidC/Oxa1 family membrane protein insertase	146.0	850
852	MGCS36089_01926			*potD*	spermidine putrescine ABC transport system	145.3	852
853	MGCS36089_02630			*dltE*	short-chain dehydrogenase DltE	145.3	852
854	MGCS36089_03758			-	type I restriction-modification system (M)	145.3	852
855	MGCS36089_00362		Virulence	*rofA*	pilus transcriptional regulator RofA	145.0	855
856	MGCS36089_04254			*pgsA*	CDP-diacylglycerol--glycerol-3-phosphate	144.8	856
857	MGCS36089_00410			*gldA*	glycerol dehydrogenase	144.5	857
858	MGCS36089_03370			*hisM*	HisM family amino acid ABC transporter permease	144.5	857
859	MGCS36089_00372	Secreted		-	secreted pilin minor/ancillary protein	144.3	859
860	MGCS36089_01608			*rgpF*	alpha-L-Rha alpha-1,3-L-rhamnosyltransferase	144.3	859
861	MGCS36089_01986			*pptA*	phenylpyruvate tautomerase PptA	144.0	861
862	MGCS36089_03306		Virulence	*liaR*	three component system signal transduction	144.0	861
863	MGCS36089_02248			*gcvH*	glycine cleavage system protein H GcvH	143.8	863
864	MGCS36089_04014			*nusG*	transcription antitermination protein NusG	143.5	864
865	MGCS36089_02240			*fhs_1*	formate--tetrahydrofolate ligase Fhs. CW-Pred predicted sortase C cell-wall anchoring	143.3	865
866	MGCS36089_03876			*rnpA*	ribonuclease P protein component RnpA	143.3	865
867	MGCS36089_00992			*ogt*	O6-methylguanine-DNA--protein-cysteine	143.0	867
868	MGCS36089_03492			*ecsB*	ABC exoprotein transporter permease EcsB	143.0	867
869	MGCS36089_03494			*ecsA*	ABC exoprotein transporter ATPase EcsA	143.0	867
870	MGCS36089_01622			-	RfbX superfamily lipopolysaccharide biosynthesis	142.5	870
871	MGCS36089_03170			*cas4*	CRISPR-associated protein Cas4	142.5	870
872	MGCS36089_01468			*add*	adenosine deaminase Add	142.3	872
873	MGCS36089_01776			*dyr*	dihydrofolate reductase Dyr	142.3	872
874	MGCS36089_00990			-	GNAT family N-acetyltransferase	142.0	874
875	MGCS36089_02544	Lipo		*ybbR*	YbbR family lipoprotein	142.0	874
876	MGCS36089_04134			*mutL*	DNA mismatch repair endonuclease MutL	142.0	874
877	MGCS36089_01046			*mtnN*	5'-methylthioadenosine/adenosylhomocysteine	141.8	877
878	MGCS36089_00878			-	hypothetical protein	141.5	878
879	MGCS36089_03384			*proA*	glutamate-5-semialdehyde dehydrogenase ProA	141.5	878
880	MGCS36089_04070			*hutG*	formiminoglutamase HutG	141.3	880

No.	Locus tag	Signal6P predicted[(1)]	Virulence[(2)]	Gene	Function	RPKMs[(3)]	RANK[(4)]
881	MGCS36089_00408			*mipB*	fructose-6-phosphate aldolase MipB	141.0	881
882	MGCS36089_01460			-	IS30 family transposase	140.3	882
883	MGCS36089_02632			*rnz*	ribonuclease Rnz	140.3	882
884	MGCS36089_02496			-	CRISPR-DR22 RNA	140.0	884
885	MGCS36089_02514			-	CRISPR-DR22 RNA	140.0	884
886	MGCS36089_01188			*thrS*	threonyl-tRNA synthetase ThrS	139.8	886
887	MGCS36089_03238			-	beta-N-acetylglucosaminidase	139.5	887
888	MGCS36089_03854			*nanK*	N-acetylmannosamine kinase NanK	139.3	888
889	MGCS36089_01074			*cvfB*	S1 RNA-binding domain-containing protein CvfB	138.8	889
890	MGCS36089_02296			*ptsC*	phosphate ABC transporter permease PstC	138.8	889
891	MGCS36089_00386			-	toxic anion resistance protein, tellurite	138.5	891
892	MGCS36089_00448			-	radical SAM protein	138.5	891
893	MGCS36089_00810			*yqeG*	HAD IIIA-type phosphatase YqeG	138.3	893
894	MGCS36089_01484			-	HAD-IA family hydrolase	138.3	893
895	MGCS36089_02392			*parC*	DNA topoisomerase IV subunit A ParC	138.3	893
896	MGCS36089_02510			-	CRISPR-DR22 RNA	138.3	893
897	MGCS36089_00558			-	Crp family cyclic nucleotide-binding	138.0	897
898	MGCS36089_00580			*cysS*	cysteine--tRNA synthetase CysS	138.0	897
899	MGCS36089_01644			*aroD*	type I 3-dehydroquinate dehydratase AroD	138.0	897
900	MGCS36089_04150			-	YitT family protein putative ABC transporter	138.0	897
901	MGCS36089_04252			*cbiO1*	cobalt ABC transporter ATPase CbiO2	138.0	897
902	MGCS36089_02622			*nth*	endonuclease III Nth	137.8	902
903	MGCS36089_02652			-	NADPH-dependent FMN reductase	137.8	902
904	MGCS36089_01992			*prmC*	peptide chain release factor N(5)-glutamine	137.5	904
905	MGCS36089_00578			-	hypothetical protein	137.3	905
906	MGCS36089_01314			*yccF*	YccF domain-containing protein	137.3	905
907	MGCS36089_01652			-	dihydrofolate synthase	137.3	905
908	MGCS36089_01902			-	polysaccharide deacetylase family protein	137.3	905
909	MGCS36089_04106			-	DUF2079 domain-containing protein	137.3	905
910	MGCS36089_02634			-	hypothetical protein	137.0	910
911	MGCS36089_00976			*holB*	DNA polymerase III subunit delta' HolB	136.8	911
912	MGCS36089_03782			-	DUF536 domain-containing protein	136.8	911
913	MGCS36089_01746	Secreted		-	extracellular cell wall anchored bifunctional. Cell-wall anchoring predicted sortase	136.5	913
914	MGCS36089_01898			*xerD_2*	site-specific integrase	136.5	913
915	MGCS36089_02640			-	DUF3042 family protein	136.5	913
916	MGCS36089_02980			*ddl*	D-alanine--D-alanine ligase Ddl	136.5	913
917	MGCS36089_04180			*pezT*	zeta toxin family protein PezT	136.5	913
918	MGCS36089_01564			*pheT*	phenylalanyl-tRNA synthetase beta subunit PheT	136.3	918
919	MGCS36089_02854			-	BglX family glycosyl hydrolase	136.3	918
920	MGCS36089_03098			-	VanZ like family glycopeptide antibiotic	135.8	920
921	MGCS36089_00518	Secreted		-	cell surface UshA family bifunctional. Cell-wall anchoring predicted sortase	135.5	921
922	MGCS36089_02962			*kup*	potassium uptake protein Kup	135.5	921

No.	Locus tag	Signal6P predicted[(1)]	Virulence[(2)]	Gene	Function	RPKMs[(3)]	RANK[(4)]
923	MGCS36089_01268			*pnpS*	phosphate TCS signal transduction histidine	135.3	923
924	MGCS36089_00318			-	DUF1033 domain-containing protein	135.0	924
925	MGCS36089_02512			-	CRISPR-DR22 RNA	134.8	925
926	MGCS36089_02876			*metK*	methionine adenosyltransferase MetK	134.0	926
927	MGCS36089_01450			-	PTS sugar transport IIB subunit	133.8	927
928	MGCS36089_01162			*rnr*	exoribonuclease (R) Rnr	133.5	928
929	MGCS36089_01772			*mvaS2*	hydroxymethylglutaryl-CoA synthase protein (2)	133.5	928
930	MGCS36089_01060			*ftsK*	cell division protein FtsK	133.3	930
931	MGCS36089_01374			-	hypothetical protein	133.3	930
932	MGCS36089_01678			-	membrane-associated alkaline phosphatase	133.3	930
933	MGCS36089_01108			-	IS1182 family transposase	133.0	933
934	MGCS36089_01888			-	MobC family plasmid mobilization relaxosome	133.0	933
935	MGCS36089_03844			*rsmA*	16S rRNA (adenine(1518)-N(6)/adenine(1519)-N(6))- dimethyltransferase RsmA	133.0	933
936	MGCS36089_04020			-	translation initiation inhibitor	132.8	936
937	MGCS36089_00360			*hslO*	Hsp33 family molecular chaperone HslO	132.0	937
938	MGCS36089_02134			-	helix-turn-helix transcriptional regulator	132.0	937
939	MGCS36089_02636			*hflX*	GTP-binding protein HflX	131.3	939
940	MGCS36089_01004			*lctP*	L-lactate permease LctP	131.0	940
941	MGCS36089_01410			*fbp2*	fructose-bisphosphatase Fbp2	131.0	940
942	MGCS36089_02974			*prfC*	peptide chain release factor 3 PrfC	131.0	940
943	MGCS36089_01492			-	DUF1694 domain-containing protein	130.8	943
944	MGCS36089_02372			-	TVP38/TMEM64 family protein	130.5	944
945	MGCS36089_03576			*gatC_2*	aspartyl-tRNA(Asn) or glutamyl-tRNA(Gln)	130.5	944
946	MGCS36089_00500			*rgfB*	endonuclease/exonuclease/phosphatase family	130.3	946
947	MGCS36089_03150			-	MmcQ/YjbR family DNA-binding protein	130.3	946
948	MGCS36089_01606			*rgpE*	glycosyltransferase family GT2 protein RgpE	130.0	948
949	MGCS36089_00740			*pyrG*	CTP synthase Pyg	129.8	949
950	MGCS36089_01756			*mvaD*	diphosphomevalonate decarboxylase MvaD	129.3	950
951	MGCS36089_01392			*rpiB*	RpiB/LacA/LacB family sugar-phosphate isomerase	128.3	951
952	MGCS36089_03026			*xseA*	exodeoxyribonuclease VII large subunit XseA	128.3	951
953	MGCS36089_02378			-	IS1182 family transposase	127.8	953
954	MGCS36089_03380			*ftsL*	cell division protein FtsL	127.8	953
955	MGCS36089_02532			-	TIGR01906 family membrane protein	127.5	955
956	MGCS36089_01334			*ppc*	phosphoenolpyruvate carboxylase Ppc	127.3	956
957	MGCS36089_01720			*fruK*	1-phosphofructokinase FruK	127.3	956
958	MGCS36089_01736			-	Uup family ATPase components of ABC transporters	127.3	956
959	MGCS36089_03018			*argR_1*	ArgR family transcriptional regulator	126.8	959
960	MGCS36089_01604			*rgpD*	ABC transporter polysaccharide/polyol phosphate	126.5	960
961	MGCS36089_03426			*degV_2*	DegV family fatty acid-binding protein	126.5	960
962	MGCS36089_01240			-	Cof-type HAD-IIB family hydrolase	126.0	962
963	MGCS36089_01904			*hom*	homoserine dehydrogenase Hom	126.0	962
964	MGCS36089_02832			-	DinB family protein	126.0	962

No.	Locus tag	Signal6P predicted[(1)]	Virulence[(2)]	Gene	Function	RPKMs[(3)]	RANK[(4)]
965	MGCS36089_03188			-	helix-hairpin-helix domain-containing protein	126.0	962
966	MGCS36089_03654			*comEB*	competence protein ComEB	126.0	962
967	MGCS36089_00424			-	putative transcriptional regulator	125.8	967
968	MGCS36089_00496			-	LacI family DNA-binding transcriptional	125.8	967
969	MGCS36089_01610			-	glycosyltransferase family 2 protein	125.5	969
970	MGCS36089_01760			-	isopentenyl-diphosphate delta-isomerase	125.5	969
971	MGCS36089_03076			*murD*	UDP-N-acetylmuramoyl-L-alanine--D-glutamate	125.5	969
972	MGCS36089_03172			*cas7*	type I-C CRISPR-associated protein Cas7/Csd2	125.5	969
973	MGCS36089_00978			*yaaT*	cell fate regulator YaaT	125.3	973
974	MGCS36089_01436			*pepD_1*	C69 family dipeptidase PepD	125.3	973
975	MGCS36089_01744			*fms*	peptide deformylase	125.3	973
976	MGCS36089_00490			-	DUF3013 family protein	125.0	976
977	MGCS36089_03202			*aroB*	3-dehydroquinate synthase protein AroB	125.0	976
978	MGCS36089_03258			*fadB*	FadB family 3-hydroxyacyl-CoA dehydrogenase	125.0	976
979	MGCS36089_01798			-	hypothetical protein	124.3	979
980	MGCS36089_03414			*glyQ*	glycine--tRNA ligase alpha subunit GlyQ	124.3	979
981	MGCS36089_03858			-	DUF624 domain-containing protein	123.8	981
982	MGCS36089_02242			*lplA_1*	lipoate--protein ligase	123.3	982
983	MGCS36089_03164			-	Gfo/Idh/MocA family oxidoreductase. 1567148..1567775. CRISPR with 10 repeat units	123.3	982
984	MGCS36089_03266			-	DUF960 domain-containing protein	123.3	982
985	MGCS36089_02444			*uvrC*	excinuclease ABC subunit UvrC	123.0	985
986	MGCS36089_02192			*topA*	type I DNA topoisomerase TopA	122.8	986
987	MGCS36089_03660			-	CorA family divalent cation transport protein	122.8	986
988	MGCS36089_01164			*smpB*	SsrA(tmRNA)-binding protein SmpB	122.3	988
989	MGCS36089_03308		Virulence	*liaS*	three component system signal transduction	122.3	988
990	MGCS36089_01300			-	PrtC family collagenase-like protease	122.0	990
991	MGCS36089_00292			*cydB*	cytochrome d ubiquinol oxidase subunit (II)	121.8	991
992	MGCS36089_01062			-	DUF3397 domain-containing protein	121.8	991
993	MGCS36089_02944			-	DUF3114 domain-containing protein	121.8	991
994	MGCS36089_03224	Lipo		*ugpB_1*	glycerol-3-phosphate ABC transporter	121.8	991
995	MGCS36089_01642			*rlmK*	23S rRNA methyltransferase RmlK	121.5	995
996	MGCS36089_00006			-	DUF951 domain-containing protein	121.3	996
997	MGCS36089_00426			-	hypothetical protein	121.3	996
998	MGCS36089_02298	Lipo		*ptsS*	phosphate ABC transporter substrate-binding	121.0	998
999	MGCS36089_02570			-	hypothetical protein	120.8	999
1000	MGCS36089_03030			*folD*	bifunctional methylenetetrahydrofolate dehydrogenase/methenyltetrahydrofolate cyclohydrolase FolD protein	120.8	999
1001	MGCS36089_03176			*cas5*	type I-C CRISPR-associated protein Cas5	120.8	999
1002	MGCS36089_03846			*rrmV*	5S rRNA maturation endonuclease RnmV	120.8	999
1003	MGCS36089_01282			*tex*	RNA-binding transcriptional accessory protein	120.5	1003
1004	MGCS36089_01432			*cofD*	CofD/YvcK superfamily 2-phospho-L-lactate	120.5	1003
1005	MGCS36089_04182			-	helix-turn-helix domain-containing	120.3	1005
1006	MGCS36089_00406			*pflD*	pyruvate formate-lyase protein PflD	120.0	1006

No.	Locus tag	Signal6P predicted[(1)]	Virulence[(2)]	Gene	Function	RPKMs[(3)]	RANK[(4)]
1007	MGCS36089_01312			-	PhoE superfamily phosphatase	120.0	1006
1008	MGCS36089_02494			-	CRISPR-DR22 RNA	120.0	1006
1009	MGCS36089_03216			*lacZ*	beta-galactosidase LacZ	119.8	1009
1010	MGCS36089_02450			*yjjG*	YjjG family noncanonical pyrimidine	119.5	1010
1011	MGCS36089_01984			-	thiamine biosynthesis protein ApbE-like protein	119.3	1011
1012	MGCS36089_02628			*recJ*	single-stranded-DNA-specific exonuclease RecJ	119.3	1011
1013	MGCS36089_02358			*lepB_1*	signal peptidase I	119.0	1013
1014	MGCS36089_01266			*pnpR*	phosphate TCS signal transduction response	118.8	1014
1015	MGCS36089_02346			-	IS1182 family transposase	118.8	1014
1016	MGCS36089_00160			*thrC*	threonine synthase ThrC	118.5	1016
1017	MGCS36089_03936			-	hypothetical protein	118.3	1017
1018	MGCS36089_00932		Virulence	*hlyX*	hemolysin family protein HylX	118.0	1018
1019	MGCS36089_02482			-	RnaY family phopsphodiesterase	118.0	1018
1020	MGCS36089_00032			*plaP*	amino acid permease PlaP	117.8	1020
1021	MGCS36089_01658			*thiI*	thiamine biosynthesis/tRNA modification protein	117.8	1020
1022	MGCS36089_01316			-	aminoacyl-tRNA deacylase	117.5	1022
1023	MGCS36089_01430			*rapZ*	RNase adapter RapZ	117.5	1022
1024	MGCS36089_01518	Secreted		-	cell surface extracellular nuclease. Cell-wall anchoring predicted sortase	117.5	1022
1025	MGCS36089_01674			*lspA*	lipoprotein signal peptidase II LspA	117.5	1022
1026	MGCS36089_01730			-	DUF1149 domain-containing protein	117.5	1022
1027	MGCS36089_04240			*sdhB*	L-serine dehydratase beta subunit SdhB	117.5	1022
1028	MGCS36089_00876			*snf*	SWF/SNF family helicase	117.3	1028
1029	MGCS36089_01028			-	DUF2829 domain-containing protein	117.3	1028
1030	MGCS36089_00780			*sufC*	Fe-S cluster assembly ATPase SufC	117.0	1030
1031	MGCS36089_01962			*nadK*	NAD kinase NadK	116.8	1031
1032	MGCS36089_02710			*bglB_1*	6-phospho-beta-glucosidase BglB	116.8	1031
1033	MGCS36089_02996			*pyrD*	dihydroorotate dehydrogenase PyrD	116.8	1031
1034	MGCS36089_00388			-	hypothetical protein	116.3	1034
1035	MGCS36089_01616			-	glycosyltransferase family 1 protein	116.3	1034
1036	MGCS36089_03504			*tsaE*	tRNA(adenosine(37)-N6)-threonylcarbamoyltransferase TsaE	116.3	1034
1037	MGCS36089_03582			*ccpN*	CcpN family CBS pair domain transcriptional	116.3	1034
1038	MGCS36089_02396			*parE*	DNA topoisomerase IV subunit B ParE	116.0	1038
1039	MGCS36089_02642			-	C4-dicarboxylate ABC transporter	115.8	1039
1040	MGCS36089_04226			-	DHH family phosphoesterase	115.8	1039
1041	MGCS36089_01002			*sdpI*	SdpI family immunity protein	115.3	1041
1042	MGCS36089_01758			*mvaK2*	mevalonate kinase MvaK2	115.3	1041
1043	MGCS36089_03756			-	type I restriction-modification system	115.0	1043
1044	MGCS36089_01320			-	TPP RNA	114.3	1044
1045	MGCS36089_01734			-	tRNA CCA-pyrophosphorylase	114.3	1044
1046	MGCS36089_04220			-	Veg family protein	114.3	1044
1047	MGCS36089_00024	Secreted		-	class A beta-lactamase-related serine hydrolase	114.0	1047
1048	MGCS36089_00950			*murJ*	peptidoglycan lipid-II intermediate flippase	114.0	1047

No.	Locus tag	Signal6P predicted[(1)]	Virulence[(2)]	Gene	Function	RPKMs[(3)]	RANK[(4)]
1049	MGCS36089_03340			*luxS*	S-ribosylhomocysteine lyase	114.0	1047
1050	MGCS36089_02836			-	23S rRNA (uracil(1939)-C(5))-methyltransferase	113.8	1050
1051	MGCS36089_03808	Lipo		*oppA_2*	oligopeptide ABC transporter substrate-binding	113.5	1051
1052	MGCS36089_01522			*queT*	Queuosine precursor transporter QueT	113.3	1052
1053	MGCS36089_02890			*udk*	uridine kinase Udk	113.3	1052
1054	MGCS36089_01576			*rexB*	ATP-dependent nuclease B subunit RexB	113.0	1054
1055	MGCS36089_01964			*rluA_1*	RluA family pseudouridine synthase	113.0	1054
1056	MGCS36089_02692	Lipo		*glnP_2*	glutamine-binding protein/glutamine transport	113.0	1054
1057	MGCS36089_02838			-	LCP family anionic cell polymer synthesis	113.0	1054
1058	MGCS36089_04214			-	IS1182 family transposase	113.0	1054
1059	MGCS36089_03686			*lepB_2*	signal peptidase I LepB	112.8	1059
1060	MGCS36089_04136			*mutS*	DNA mismatch repair protein MutS	112.8	1059
1061	MGCS36089_01400			-	hypothetical protein	112.5	1061
1062	MGCS36089_03028			-	PfkB superfamily kinase	112.0	1062
1063	MGCS36089_02402			*ung*	uracil-DNA glycosylase Ung	111.8	1063
1064	MGCS36089_03796			-	DUF1447 family protein	111.8	1063
1065	MGCS36089_04198			-	DUF368 domain-containing protein	111.8	1063
1066	MGCS36089_00852			*ktrA*	potassium uptake transporter gating subunit	111.5	1066
1067	MGCS36089_01000			*exoA*	exodeoxyribonuclease III protein ExoA	111.5	1066
1068	MGCS36089_02002			-	lysozyme family protein	111.5	1066
1069	MGCS36089_02536			*fatA*	FatA family acyl-[acyl-carrier-protein]	111.5	1066
1070	MGCS36089_01040			*glmU*	bifunctional UDP-N-acetylglucosamine diphosphorylase/glucosamine-1-phosphate N-acetyltransferase GlmU	111.0	1070
1071	MGCS36089_01620			*galE*	UDP-glucose 4-epimerase GalE	111.0	1070
1072	MGCS36089_03856			*nanA*	N-acetylneuraminate lyase	110.3	1072
1073	MGCS36089_01886			-	SAG1252 family conjugative relaxosome accessory	109.5	1073
1074	MGCS36089_01068			-	hypothetical protein. SignalP-6 predicted standard secretion signal	109.0	1074
1075	MGCS36089_03252			*caiC*	CaiC family Acyl-CoA synthetase	109.0	1074
1076	MGCS36089_01448			*bga*	beta-galactosidase Bga	108.8	1076
1077	MGCS36089_02178			*gid*	tRNA (uracil-5-)-methyltransferase/glucose	108.8	1076
1078	MGCS36089_02866			*fadR*	FadR family DNA-binding transcriptional	108.8	1076
1079	MGCS36089_03386			*proB*	glutamate 5-kinase ProB	108.5	1079
1080	MGCS36089_00010			*engD*	redox-regulated ATPase EngD	108.3	1080
1081	MGCS36089_03636			-	IS30 family transposase	108.3	1080
1082	MGCS36089_03866	Lipo		*ugpB_2*	carbohydrate ABC transporter substrate-binding	108.3	1080
1083	MGCS36089_01160			*secG*	preprotein translocase subunit SecG	108.0	1083
1084	MGCS36089_02546			-	DisA N domain-containing diadenylate cyclase	108.0	1083
1085	MGCS36089_02978			*murF*	UDP-N-acetylmuramoyl-tripeptide--D-alanyl-D-	107.5	1085
1086	MGCS36089_04094			*yaaA*	peroxide stress protein YaaA	107.5	1085
1087	MGCS36089_03152		Virulence	*yesM*	TCS sensor kinase YesM	107.3	1087
1088	MGCS36089_02492			-	CRISPR-DR22 RNA	107.0	1088
1089	MGCS36089_00352			-	DUF4479 and tRNA-binding domain-containing	106.5	1089
1090	MGCS36089_02244			-	NAD-dependent deacetylase	106.5	1089

No.	Locus tag	Signal6P predicted[(1)]	Virulence[(2)]	Gene	Function	RPKMs[(3)]	RANK[(4)]
1091	MGCS36089_04072			-	AAA family ATPase domain-containing DNA-binding	105.8	1091
1092	MGCS36089_00340			-	CAAX amino terminal protease family membrane	105.5	1092
1093	MGCS36089_01390			*idnO*	gluconate 5-dehydrogenase IdnO	105.5	1092
1094	MGCS36089_01942			-	AI-2E family transporter	105.5	1092
1095	MGCS36089_02436			*mnmE*	MnmE family tRNA	105.3	1095
1096	MGCS36089_00504			-	M42 family metallopeptidase	104.8	1096
1097	MGCS36089_01890			-	SAG1250 family conjugative relaxase	104.8	1096
1098	MGCS36089_01216			*glnP_1*	glutamine ABC transporter permease GlnP	104.5	1098
1099	MGCS36089_00452			-	MdlB family ABC transporter ATP-binding/permease	104.0	1099
1100	MGCS36089_01554			-	DUF1146 domain-containing protein	103.5	1100
1101	MGCS36089_01560			*endA*	DNA-entry competence-associated nuclease EndA	103.3	1101
1102	MGCS36089_02364			*dnaE*	DNA polymerase III subunit alpha DnaE	103.3	1101
1103	MGCS36089_02616			*zupT*	ZIP family metal transporter ZupT	103.3	1101
1104	MGCS36089_03938	Secreted	Virulence	*speG*	streptococcal pyrogenic exotoxin (G) SpeG	103.3	1101
1105	MGCS36089_03692			*dinB*	DNA polymerase IV DinB	103.0	1105
1106	MGCS36089_00540			*uppS*	UDP pyrophosphate synthase UppS	102.5	1106
1107	MGCS36089_02982			*recR*	recombination mediator RecR	102.5	1106
1108	MGCS36089_00854			*ktrB*	potassium uptake transporter channel subunit	102.0	1108
1109	MGCS36089_01562			*pheS*	phenylalanyl-tRNA synthetase alpha subunit PheS	102.0	1108
1110	MGCS36089_02124			*mngR*	MngR family DNA-binding transcriptional	102.0	1108
1111	MGCS36089_03410			-	DUF896 family protein	102.0	1108
1112	MGCS36089_03122			*sdrC*	SdrC family PDZ domain-containing protein	101.5	1112
1113	MGCS36089_03896			-	carbonic anhydrase	101.5	1112
1114	MGCS36089_01662			-	IS30 family transposase	101.3	1114
1115	MGCS36089_02252			*lipL*	lipoate--protein ligase	101.0	1115
1116	MGCS36089_02842			*aroA*	3-phosphoshikimate 1-carboxyvinyltransferase	101.0	1115
1117	MGCS36089_03218		Virulence	*trxR*	TCS DNA-binding response regulator protein TrxR	101.0	1115
1118	MGCS36089_03300			*cysK*	cysteine synthase A CysK	101.0	1115
1119	MGCS36089_02484			*msrB*	peptide-methionine (R)-S-oxide reductase MsrB	100.8	1119
1120	MGCS36089_03898			*radA*	DNA repair protein RadA	100.8	1119
1121	MGCS36089_00724			*truA*	tRNA pseudouridine(38-40) synthase TruA	100.5	1121
1122	MGCS36089_04148			-	YitT family protein putative ABC transporter	100.5	1121
1123	MGCS36089_04266			*glcU*	glucose uptake permease GlcU	100.5	1121
1124	MGCS36089_00868			*nrdR*	transcriptional regulator NrdR	100.3	1124
1125	MGCS36089_01646			*aroC*	chorismate synthase AroC	100.3	1124
1126	MGCS36089_03900			*dut*	deoxyuridine 5'-triphosphate nucleotidohydrolase	100.3	1124
1127	MGCS36089_03520			-	DUF956 family protein	100.0	1127
1128	MGCS36089_01366			-	HD domain-containing phosphohydrolase	99.8	1128
1129	MGCS36089_00394			*pflC*	pyruvate formate-lyase activating enzyme PflC	99.5	1129
1130	MGCS36089_02340			-	Glycine RNA	99.3	1130
1131	MGCS36089_02042		Virulence	*ihk*	TCS signal transduction histidine kinase sensor	98.8	1131
1132	MGCS36089_01840			-	DUF5966 family protein	98.3	1132

No.	Locus tag	Signal6P predicted[(1)]	Virulence[(2)]	Gene	Function	RPKMs[(3)]	RANK[(4)]
1133	MGCS36089_02618			-	Nif3-like dinuclear metal center hexameric	98.3	1132
1134	MGCS36089_03670			-	hypothetical protein	97.8	1134
1135	MGCS36089_02154	Lipo		-	FMN-binding protein, major membrane immunogen	97.5	1135
1136	MGCS36089_02848			*spxR*	SpxR family CBS-HotDog domain-containing	97.5	1135
1137	MGCS36089_01442			*agaS*	AgaS superfamily sugar isomerase SIS	97.3	1137
1138	MGCS36089_02202			-	DUF1836 domain-containing protein	97.3	1137
1139	MGCS36089_02908			*clcB*	voltage-gated ClC-type chloride channel ClcB	97.3	1137
1140	MGCS36089_03656			*pepP*	PepP family Xaa-Pro peptidase	96.5	1140
1141	MGCS36089_03764			-	hypothetical protein	96.3	1141
1142	MGCS36089_03848			*tatD*	Tat protein secretion system quality control	96.3	1141
1143	MGCS36089_00656			-	type II toxin-antitoxin system Phd/YefM family	96.0	1143
1144	MGCS36089_00706			-	IS30 family transposase	96.0	1143
1145	MGCS36089_01218			-	glutamine ABC transporter permease	96.0	1143
1146	MGCS36089_01376			*regR*	transcriptional regulator RegR	96.0	1143
1147	MGCS36089_01572			-	ABC transporter ATP-binding component LolD-like	95.3	1147
1148	MGCS36089_01672			*lysR*	LysR family transcriptional regulator	95.3	1147
1149	MGCS36089_02834			-	CD1845 family protein	95.0	1149
1150	MGCS36089_03790			*tsaD*	tRNA (adenosine(37)-N6)-threonylcarbamoyltransferase complex transferase subunit TsaD	95.0	1149
1151	MGCS36089_00440			-	hypothetical protein	94.8	1151
1152	MGCS36089_01726	Lipo		-	putative peptidoglycan hydrolase lipoprotein	94.8	1151
1153	MGCS36089_00502			-	Udk family kinase	94.3	1153
1154	MGCS36089_00704			-	Cof-type HAD-IIB family hydrolase	94.3	1153
1155	MGCS36089_01408			-	HAD family phosphatase	94.0	1155
1156	MGCS36089_01614			-	DUF2142 domain-containing protein	94.0	1155
1157	MGCS36089_02902			*nrdH*	glutaredoxin-like protein NrdH	94.0	1155
1158	MGCS36089_04124			*cinA*	competence/damage-inducible protein CinA	93.8	1158
1159	MGCS36089_00768			-	ABC amino acid transporter ATP-binding protein	93.0	1159
1160	MGCS36089_00974			*tmk*	thymidylate kinase Tmk	93.0	1159
1161	MGCS36089_01618			-	LTA synthase family protein	93.0	1159
1162	MGCS36089_01784			-	hypothetical protein	93.0	1159
1163	MGCS36089_00770	Secreted		-	ABC amino acid transporter substrate-binding protein	92.8	1163
1164	MGCS36089_01476			*clcA*	voltage-gated chloride channel family protein	92.8	1163
1165	MGCS36089_03168			*cas1_2*	type I-C CRISPR-associated endonuclease Cas1	92.8	1163
1166	MGCS36089_03318			*fmt*	methionyl-tRNA formyl transferase Fmt	92.8	1163
1167	MGCS36089_01878			*arsR*	cadmium efflux system metalloregulator ArsR/SmtB	92.5	1167
1168	MGCS36089_03710			-	beta-lactamase family protein	92.5	1167
1169	MGCS36089_03954			-	DUF975 family protein	92.5	1167
1170	MGCS36089_01570			-	ABC transporter permease	92.0	1170
1171	MGCS36089_04286			*rlmH*	23S rRNA (pseudouridine(1915)-N(3))-methyltransferase RlmH	92.0	1170
1172	MGCS36089_01710			*trmD*	tRNA (guanosine(37)-N1)-methyltransferase TrmD	91.8	1172
1173	MGCS36089_02874			*murA_2*	UDP-N-acetylglucosamine	91.8	1172

No.	Locus tag	Signal6P predicted[(1)]	Virulence[(2)]	Gene	Function	RPKMs[(3)]	RANK[(4)]
1174	MGCS36089_00290			*cydA*	cytochrome ubiquinol oxidase subunit (I) CydA	91.5	1174
1175	MGCS36089_00532	Secreted	Virulence	*pulA_1*	cell surface pullulanase PulA. Cell-wall anchoring predicted sortase	91.5	1174
1176	MGCS36089_01830	Secreted		-	cell surface extracellular antigen I/II family. Cell-wall anchoring predicted sortase	91.0	1176
1177	MGCS36089_03260			*trmA*	TrmA RNA methyltransferase	91.0	1176
1178	MGCS36089_02476			-	DUF2974 domain-containing protein	90.8	1178
1179	MGCS36089_00140			*purB*	adenylosuccinate lyase PurB	90.3	1179
1180	MGCS36089_00988			*serC*	3-phosphoserine/phosphohydroxythreonine	90.3	1179
1181	MGCS36089_02198			*ylqF*	ribosome biogenesis GTPase YlqF	90.0	1181
1182	MGCS36089_03200			*aroGA*	bifunctional 3-deoxy-7-phosphoheptulonate synthase/chorismate mutase AroGA	90.0	1181
1183	MGCS36089_04146			-	YitT family protein putative ABC transporter	90.0	1181
1184	MGCS36089_02040		Virulence	*irr*	TCS signal transduction DNA-binding response	89.8	1184
1185	MGCS36089_02130			-	hypothetical protein	89.8	1184
1186	MGCS36089_03290			-	IS30 family transposase	89.8	1184
1187	MGCS36089_00020			*divIC*	septum formation initiator family protein	89.5	1187
1188	MGCS36089_02538			*hemN*	HemN family coproporphyrinogen III oxidase or	89.5	1187
1189	MGCS36089_03752			-	major facilitator superfamily protein	89.3	1189
1190	MGCS36089_04230			*mnmG*	tRNA uridine-5-carboxymethylaminomethyl(34)	89.3	1189
1191	MGCS36089_00732			-	mechanosensitive ion channel family protein	89.0	1191
1192	MGCS36089_02250			-	NADH-dependent flavin oxidoreductase	88.8	1192
1193	MGCS36089_02620			*trmK*	tRNA (adenine(22)-N(1))-methyltransferase TrmK	88.8	1192
1194	MGCS36089_03174			*cas8*	type I-C CRISPR-associated protein Cas8c/Csd1	88.8	1192
1195	MGCS36089_01602			*rgpC*	ABC transporter polysaccharide/polyol phosphate	88.5	1195
1196	MGCS36089_00350			*trxA_1*	thioredoxin family protein TrxA-like protein	88.3	1196
1197	MGCS36089_01310			-	HAD family hydrolase	88.0	1197
1198	MGCS36089_02228			-	asd RNA	88.0	1197
1199	MGCS36089_02278			*coaA*	type I pantothenate kinase	87.8	1199
1200	MGCS36089_02840			*aroK*	shikimate kinase AroK	86.8	1200
1201	MGCS36089_02930			-	NAD(P)H-dependent oxidoreductase	86.3	1201
1202	MGCS36089_02976			-	TIGR02206 family membrane protein	86.3	1201
1203	MGCS36089_02390			*bcaT*	branched-chain amino acid aminotransferase	86.0	1203
1204	MGCS36089_01112		Virulence	*silA*	TCS DNA-binding response regulator SilA	85.8	1204
1205	MGCS36089_01612			-	DUF2304 domain-containing protein	85.8	1204
1206	MGCS36089_02422			-	LysR family transcriptional regulator	85.8	1204
1207	MGCS36089_02910			-	CPBP family intramembrane metalloprotease	85.8	1204
1208	MGCS36089_01814			*traG_1*	type IV secretory system conjugative DNA	85.3	1208
1209	MGCS36089_02984			*pbp2B*	penicillin-binding protein PBP2B/FtsI	85.3	1208
1210	MGCS36089_00288			*nox*	NAD(P)/FAD-dependent oxidoreductase Nox	85.0	1210
1211	MGCS36089_01838			-	DUF5962 family protein	85.0	1210
1212	MGCS36089_02246			-	protein-ADP-ribose hydrolase	84.8	1212
1213	MGCS36089_03124			*coaD*	pantetheine-phosphate adenylyltransferase CoaD	84.8	1212
1214	MGCS36089_03688			*recD*	ATP-dependent DNA helicase RecD	84.8	1212
1215	MGCS36089_01212			*ppsB*	long-chain fatty acid--CoA ligase PpsB	84.5	1215

No.	Locus tag	Signal6P predicted[(1)]	Virulence[(2)]	Gene	Function	RPKMs[(3)]	RANK[(4)]
1216	MGCS36089_02230			*cls*	cardiolipin synthase	84.5	1215
1217	MGCS36089_03178			*cas3*	CRISPR-associated helicase Cas3	84.3	1217
1218	MGCS36089_04026			-	hypothetical protein	84.3	1217
1219	MGCS36089_03240			-	PurR/LacI family transcriptional regulator	84.0	1219
1220	MGCS36089_02912			-	DUF3169 family protein	83.5	1220
1221	MGCS36089_00512			*nrdI_1*	class Ib ribonucleoside-diphosphate reductase	83.3	1221
1222	MGCS36089_02856			-	phosphoglycolate phosphatase	83.3	1221
1223	MGCS36089_00380			-	PAS domain-containing protein	83.0	1223
1224	MGCS36089_02220			*pdxK*	pyridoxamine kinase PdxK	83.0	1223
1225	MGCS36089_01220	Secreted		-	glutamine ABC transporter substrate-binding	82.8	1225
1226	MGCS36089_01210			*paaJ*	acetyl-CoA acetyl transferase PaaJ	82.5	1226
1227	MGCS36089_01774			*thyA*	thymidylate synthase ThyA	82.5	1226
1228	MGCS36089_02452			-	DUF1524 domain-containing protein	82.5	1226
1229	MGCS36089_03190			-	DUF1912 family protein	82.0	1229
1230	MGCS36089_03428			-	TetR/AcrR family transcriptional regulator	82.0	1229
1231	MGCS36089_00524	Secreted	Virulence	*ska*	secreted streptokinase Ska	81.8	1231
1232	MGCS36089_00826			-	nucleotidyltransferase	81.8	1231
1233	MGCS36089_04038			*ctsR*	CtsR family transcriptional regulator	81.8	1231
1234	MGCS36089_02312			-	DUF2130 domain-containing protein	81.5	1234
1235	MGCS36089_04264			*recF*	DNA replication/repair protein RecF	81.5	1234
1236	MGCS36089_00022			-	hypothetical protein	81.3	1236
1237	MGCS36089_02648			-	rhodanese-related sulfurtransferase	81.3	1236
1238	MGCS36089_00924			-	ECF transporter S component	80.5	1238
1239	MGCS36089_02338			*pcrA*	DNA helicase PcrA	80.0	1239
1240	MGCS36089_03860			-	DUF386 family protein	80.0	1239
1241	MGCS36089_02376			-	SDUF3862 domain-containing lipoprotein. ignalP-6 predicted lipid anchoring signal peptide,	79.8	1241
1242	MGCS36089_01038			-	UhpC family MFS transporter	79.5	1242
1243	MGCS36089_04044			-	metallo-dependent amidohydrolase	79.5	1242
1244	MGCS36089_00280			-	XRE family ImmR-like transcriptional regulator	79.3	1244
1245	MGCS36089_01598			*rgpA*	glycosyltransferase family 1 protein RgpA	79.3	1244
1246	MGCS36089_03580			-	kinase/pyrophosphorylase	79.3	1244
1247	MGCS36089_01600			*rgpB*	glycosyltransferase family GT2 protein RgpB	78.8	1247
1248	MGCS36089_03222			*trxT*	Trx TCS operon protein TrxT	78.8	1247
1249	MGCS36089_00608			-	helix-turn-helix transcriptional regulator	78.5	1249
1250	MGCS36089_00396			-	GlpR-like transcriptional regulator protein	78.3	1250
1251	MGCS36089_01988			*tdk*	thymidine kinase Tdk	78.3	1250
1252	MGCS36089_00164			-	IS30 family transposase	78.0	1252
1253	MGCS36089_03154		Virulence	*yesN*	TCS DNA-binding response regulator YesN	78.0	1252
1254	MGCS36089_00514		Virulence	*mga*	M protein trans-acting positive regulator Mga	77.8	1254
1255	MGCS36089_02886			*prsW*	PrsW family glutamic-type intramembrane	77.8	1254
1256	MGCS36089_03942	Lipo		-	CYK3 family lipoprotein putatively invloved in	77.8	1254
1257	MGCS36089_01192			-	putative ABC transporter permease component	77.5	1257

No.	Locus tag	Signal6P predicted[(1)]	Virulence[(2)]	Gene	Function	RPKMs[(3)]	RANK[(4)]
1258	MGCS36089_00756			*rpmB*	50S ribosomal L28 protein RpmB	77.3	1258
1259	MGCS36089_03784			*yhiN*	YhiN family predicted flavoprotein	76.3	1259
1260	MGCS36089_02456			*ycjU*	YcjU family beta-phosphoglucomutase or related	75.8	1260
1261	MGCS36089_01422			*dinG*	bifunctional DnaQ family exonuclease -- ATP-dependent helicase DinG	75.5	1261
1262	MGCS36089_00026			*tilS*	tRNA lysidine(34) synthetase TilS	74.8	1262
1263	MGCS36089_00744			*lytR*	TCS DNA-binding response regulator LytR	74.8	1262
1264	MGCS36089_01816			-	hypothetical protein	74.5	1264
1265	MGCS36089_01960			-	GTP pyrophosphokinase family protein	74.5	1264
1266	MGCS36089_03080			-	DUF3165 family protein	74.3	1266
1267	MGCS36089_01078			*yozE*	YozE family protein	74.0	1267
1268	MGCS36089_01740			-	MdlB family multidrug ABC transporter ATPase and	73.8	1268
1269	MGCS36089_00334			-	class I SAM-dependent methyltransferase	73.5	1269
1270	MGCS36089_00596			-	helix-turn-helix transcriptional regulator	73.5	1269
1271	MGCS36089_04022			-	hypothetical protein	73.0	1271
1272	MGCS36089_03570			-	drug/metabolite transporter superfamily protein	72.8	1272
1273	MGCS36089_00600			-	L13_leader RNA	72.5	1273
1274	MGCS36089_00742			*lytS*	TCS sensor histidine kinase LytS	72.3	1274
1275	MGCS36089_01900	Secreted		*dacA_3*	secreted D,D-carboxypeptidase penicillin-binding	72.3	1274
1276	MGCS36089_02454			-	maltose/galactose O-acetyltransferase	72.3	1274
1277	MGCS36089_01246			-	MFS transporter	72.0	1277
1278	MGCS36089_01656			*nifS_1*	cysteine desulfurase NifS	72.0	1277
1279	MGCS36089_01718			*fruR*	fructose operon transcriptional repressor	71.5	1279
1280	MGCS36089_00298			*preA*	polyprenyl synthetase family protein PreA	71.3	1280
1281	MGCS36089_00398			-	DeoR-like transcriptional regulator protein	71.3	1280
1282	MGCS36089_02196			*rnhB*	HII ribonuclease RnhB	71.3	1280
1283	MGCS36089_02888			-	DUF1294 domain-containing protein	71.3	1280
1284	MGCS36089_03220		Virulence	*trxS*	TCS sensor histidine kinase TrxS	71.0	1284
1285	MGCS36089_02478			*spiA*	sakacin P immunity protein SpiA	70.5	1285
1286	MGCS36089_02950			*holA*	DNA polymerase III delta subunit HolA	70.5	1285
1287	MGCS36089_02004			-	MdlB superfamily multidrug ABC transporter	70.3	1287
1288	MGCS36089_03608			*cbiO*	cobalt ECF transporter (A) ATPase component	70.3	1287
1289	MGCS36089_02406	Lipo		-	amino-acid ABC transporter substrate-binding	70.0	1289
1290	MGCS36089_03262			*recX*	recombination regulator RecX	70.0	1289
1291	MGCS36089_03294			*comFC*	ComFC family predicted	70.0	1289
1292	MGCS36089_03366			-	DUF4059 family protein	70.0	1289
1293	MGCS36089_01868			*copZ_1*	copper chaperone CopZ	69.8	1293
1294	MGCS36089_02126			*ylxM*	YlxM superfamily signal recognition particle	69.8	1293
1295	MGCS36089_01020			*nrdE_1*	class 1b ribonucleoside-diphosphate reductase	69.5	1295
1296	MGCS36089_02614			*dadA*	FAD-binding oxidoreductase DadA	69.5	1295
1297	MGCS36089_02724			*rsuA_1*	ribosomal small subunit pseudouridine synthase	69.5	1295
1298	MGCS36089_03038			*feoB*	ferrous iron transport protein (B) FeoB	69.5	1295
1299	MGCS36089_03640			*scrK*	fructokinase protein ScrK	69.5	1295

No.	Locus tag	Signal6P predicted[(1)]	Virulence[(2)]	Gene	Function	RPKMs[(3)]	RANK[(4)]
1300	MGCS36089_01222			*glnQ_1*	glutamine ABC transporter ATPase GlnQ	69.3	1300
1301	MGCS36089_02304			-	UPF0223 family protein	69.3	1300
1302	MGCS36089_02398			*plsY*	glycerol-3-phosphate 1-O-acyltransferase PlsY	69.3	1300
1303	MGCS36089_03800			*oppF_2*	oligopeptide ABC transporter ATPase OppF	69.3	1300
1304	MGCS36089_00956			-	DUF4298 domain-containing protein	69.0	1304
1305	MGCS36089_01328	Lipo		-	DUF4430 domain-containing lipoprotein	68.5	1305
1306	MGCS36089_02490			-	CRISPR-DR22 RNA	68.5	1305
1307	MGCS36089_03602			*asnB*	L-asparaginase AsnB	68.5	1305
1308	MGCS36089_00598			-	IS30 family transposase	68.3	1308
1309	MGCS36089_02924			-	OFA family MFS transporter	68.3	1308
1310	MGCS36089_01754			*mvaK1*	mevalonate kinase MvaK1	68.0	1310
1311	MGCS36089_02218			-	ECF transporter S component	67.8	1311
1312	MGCS36089_02348	Lipo		-	amino acid ABC transporter substrate-binding	67.8	1311
1313	MGCS36089_04016			*secE*	preprotein translocase subunit protein SecE	67.5	1313
1314	MGCS36089_01866			*kdpB*	Potassium-transporting ATPase ATP-binding	67.3	1314
1315	MGCS36089_00930			-	RsmB/NOP family class I SAM-dependent RNA	67.0	1315
1316	MGCS36089_00536			-	thioredoxin family protein	66.8	1316
1317	MGCS36089_02146			*xerS*	site-specific tyrosine recombinase XerS	66.8	1316
1318	MGCS36089_02940			*nagB*	glucosamine-6-phosphate deaminase NagB	66.5	1318
1319	MGCS36089_00668			-	hypothetical protein	66.3	1319
1320	MGCS36089_01194			-	putative ABC transporter permease component	66.3	1319
1321	MGCS36089_01742			*gdhA*	NADP-specific glutamate dehydrogenase GdhA	66.3	1319
1322	MGCS36089_01818			*trbL*	conjugal transfer protein TrbL	66.3	1319
1323	MGCS36089_03610			*cbiQ_1*	cobalt ECF transporter (T) transmembrane	66.3	1319
1324	MGCS36089_04042			*cspA*	cold-shock protein CspA	65.8	1324
1325	MGCS36089_00606			-	site-specific integrase	65.5	1325
1326	MGCS36089_03126			*rsmD*	16S rRNA (guanine(966)-N(2))-methyltransferase	65.5	1325
1327	MGCS36089_03332			*atoD*	butyrate-acetoacetate CoA-transferase alpha	65.5	1325
1328	MGCS36089_01358			-	FMN-binding domain containing L-lactate oxidase	65.3	1328
1329	MGCS36089_00458			-	PhoE family phosphoglycerate mutase	65.0	1329
1330	MGCS36089_02458			*ykgJ*	YkgJ family cysteine cluster protein	65.0	1329
1331	MGCS36089_03310		Virulence	*liaF*	three component system signal transduction	65.0	1329
1332	MGCS36089_00278			-	HNH endonuclease	64.8	1332
1333	MGCS36089_03034			*phrB*	PhrB family deoxyribodipyrimidine photolyase	64.8	1332
1334	MGCS36089_01076			*msrA*	peptide-methionine (S)-S-oxide reductase MsrA	64.5	1334
1335	MGCS36089_02922			*yrrM*	YrrM family O-methyltransferase	64.5	1334
1336	MGCS36089_02926			-	HAD family hydrolase	64.5	1334
1337	MGCS36089_03166			*cas2_2*	CRISPR-associated endonuclease Cas2	64.5	1334
1338	MGCS36089_01470			-	IS1548 family transposase	64.3	1338
1339	MGCS36089_02276			*rsmC*	class I SAM-dependent methyltransferase	64.3	1338
1340	MGCS36089_02316			*hsdS*	type I restriction endonuclease subunit S	64.3	1338
1341	MGCS36089_02850			-	GNAT family N-acetyltransferase	64.0	1341

No.	Locus tag	Signal6P predicted[(1)]	Virulence[(2)]	Gene	Function	RPKMs[(3)]	RANK[(4)]
1342	MGCS36089_03094			-	MdlB family multidrug ABC transporter	64.0	1341
1343	MGCS36089_00824			-	class I SAM-dependent methyltransferase	63.8	1343
1344	MGCS36089_01022			*brnQ_2*	branched-chain amino acid transport system II	63.8	1343
1345	MGCS36089_01116			-	IS1548 family transposase	63.8	1343
1346	MGCS36089_02350			-	amino acid ABC transporter ATP-binding protein	63.8	1343
1347	MGCS36089_03088			-	DUF910 domain-containing protein	63.5	1347
1348	MGCS36089_03794			*tsaB*	tRNA (adenosine(37)-N6)-threonylcarbamoyltransferase complex dimerization subunit type 1 TsaB	63.3	1348
1349	MGCS36089_03868			*nanE*	N-acetylmannosamine-6-phosphate 2-epimerase	63.0	1349
1350	MGCS36089_02388			-	DUF2969 domain-containing protein	62.8	1350
1351	MGCS36089_02314			-	aromatic acid exporter family protein	62.5	1351
1352	MGCS36089_01738			-	MdlB family multidrug ABC transporter ATPase and	62.3	1352
1353	MGCS36089_01812			-	hypothetical protein	62.3	1352
1354	MGCS36089_03320			*priA*	primosomal protein PriA	62.3	1352
1355	MGCS36089_04192			-	IS982 family transposase	62.3	1352
1356	MGCS36089_02522			*csn2*	CRISPR-associated protein Csn2	61.8	1356
1357	MGCS36089_03092			-	hypothetical protein	61.8	1356
1358	MGCS36089_03096			-	SmdA family multidrug ABC transporter	61.3	1358
1359	MGCS36089_03104			-	IS1548 family transposase	61.3	1358
1360	MGCS36089_03988			-	IS1548 family transposase	61.3	1358
1361	MGCS36089_00548			-	nucleotidyltransferase family protein	61.0	1361
1362	MGCS36089_01016			*nrdF_1*	ribonucleotide-diphosphate reductase subunit	61.0	1361
1363	MGCS36089_01856			-	hypothetical protein	61.0	1361
1364	MGCS36089_03918			-	5-formyltetrahydrofolate cyclo-ligase	61.0	1361
1365	MGCS36089_04228			-	IS1548 family transposase	61.0	1361
1366	MGCS36089_00008			-	helix-turn-helix domain-containing protein	60.8	1366
1367	MGCS36089_03862			*ugpE_2*	carbohydrate ABC transporter permease UgpE-like	60.8	1366
1368	MGCS36089_01270	Lipo		*pstS*	phosphate ABC transporter substrate-binding protein	60.5	1368
1369	MGCS36089_01440			-	GntR family transcriptional regulator	60.5	1368
1370	MGCS36089_01728			-	hypothetical protein	60.5	1368
1371	MGCS36089_02410			-	IS3 family transposase	60.5	1368
1372	MGCS36089_02526			*cas1_1*	type II CRISPR-associated endonuclease Cas1	60.5	1368
1373	MGCS36089_03802			*oppD_2*	oligopeptide ABC transporter ATPase OppD	60.3	1373
1374	MGCS36089_03940			-	Bacteria_small_SRP RNA	60.3	1373
1375	MGCS36089_00034			-	IS30 family transposase	60.0	1375
1376	MGCS36089_01208			-	acyl dehydratase	60.0	1375
1377	MGCS36089_02960			-	GIY-YIG catalytic domain-containing putative	59.8	1377
1378	MGCS36089_00856			*rsmG*	16S rRNA (guanine(527)-N(7))-methyltransferase	59.5	1378
1379	MGCS36089_01254			*bglA*	6-phospho-beta-glucosidase BglA	59.5	1378
1380	MGCS36089_00654			-	Txe/YoeB family addiction module toxin	59.3	1380
1381	MGCS36089_01826			-	nucleotidyl transferase AbiEii/AbiGii toxin	59.3	1380
1382	MGCS36089_02006			-	MdlB superfamily multidrug ABC transporter	59.3	1380
1383	MGCS36089_03334			*atoA*	acyl CoA:acetate/3-ketoacid CoA transferase beta	59.3	1380

No.	Locus tag	Signal6P predicted[(1)]	Virulence[(2)]	Gene	Function	RPKMs[(3)]	RANK[(4)]
1384	MGCS36089_03672			*mutY*	A/G-specific adenine glycosylase MutY	59.3	1380
1385	MGCS36089_04232			-	NUDIX domain-containing protein	59.3	1380
1386	MGCS36089_02994			-	IS982 family transposase	59.0	1386
1387	MGCS36089_04248			*cbiQ_2*	cobalt ABC transporter permease CbiQ	58.5	1387
1388	MGCS36089_04250			*cbiO2*	cobalt ABC transporter ATPase CbiO1	58.5	1387
1389	MGCS36089_02180			-	DUF3307 domain-containing protein	58.3	1389
1390	MGCS36089_02404			-	amino acid ABC transporter permease	58.3	1389
1391	MGCS36089_02586	Secreted		-	putative secreted protein	58.3	1389
1392	MGCS36089_03212			-	permease	58.0	1392
1393	MGCS36089_01190			-	putative ABC transporter ATP-binding component	57.8	1393
1394	MGCS36089_02162			-	BaeS family TCS histidine kinase sensor	57.8	1393
1395	MGCS36089_02164			-	OmpR family TCS DNA-binding response regulator	57.5	1395
1396	MGCS36089_02302			-	inositol monophosphatase family protein	57.5	1395
1397	MGCS36089_02352			-	amino acid ABC transporter permease	57.5	1395
1398	MGCS36089_03916			*glpG*	GlpG family membrane associated serine protease	57.5	1395
1399	MGCS36089_00384			-	PTS sugar transporter subunit IIC	57.3	1399
1400	MGCS36089_03054			-	hypothetical protein	57.0	1400
1401	MGCS36089_03194			-	MOP/MATE family multidrug-resistance efflux	57.0	1400
1402	MGCS36089_01894			-	pemK RNA	56.5	1402
1403	MGCS36089_01368			-	DUF1934 domain-containing protein	56.0	1403
1404	MGCS36089_03924			-	Mga-related helix-turn-helix domain-containing	56.0	1403
1405	MGCS36089_00758			-	IS1548 family transposase	55.8	1405
1406	MGCS36089_03852			*rpiR*	RpiR family transcriptional regulator	55.8	1405
1407	MGCS36089_01584			-	IS1548 family transposase	55.5	1407
1408	MGCS36089_00108			*recO*	DNA repair protein RecO	55.3	1408
1409	MGCS36089_00126			*purH*	bifunctional phosphoribosylaminoimidazolecarboxamide formyltransferase/IMP cyclohydrolase PurH	55.3	1408
1410	MGCS36089_02524			*cas2_1*	CRISPR-associated endoribonuclease Cas2	55.3	1408
1411	MGCS36089_03864			*ugpA_2*	carbohydrate ABC transporter permease UgpA-like	55.3	1408
1412	MGCS36089_03922			-	IS30 family transposase	55.3	1408
1413	MGCS36089_00594			-	hypothetical protein	55.0	1413
1414	MGCS36089_01018			*nrdI_2*	ribonucleotide reductase assembly protein NrdI	55.0	1413
1415	MGCS36089_02412			-	GNAT family N-acetyltransferase	55.0	1413
1416	MGCS36089_02568			-	ABC transporter ATP-binding protein	54.5	1416
1417	MGCS36089_03210			-	permease	54.3	1417
1418	MGCS36089_03338			*gntT*	GntT family H+/gluconate symporter or related	54.3	1417
1419	MGCS36089_04270			*trpS*	tryptophanyl-tRNA synthetase	54.3	1417
1420	MGCS36089_00228			-	IS30 family transposase	54.0	1420
1421	MGCS36089_01238			-	IS1548 family transposase	53.8	1421
1422	MGCS36089_01896			-	DUF3173 domain-containing protein	53.8	1421
1423	MGCS36089_04258			*pqqF*	pitrilysin family predicted Zn-dependent	53.5	1423
1424	MGCS36089_01302			-	YdbC family protein	53.0	1424
1425	MGCS36089_03748			-	MFS transporter	53.0	1424

No.	Locus tag	Signal6P predicted[(1)]	Virulence[(2)]	Gene	Function	RPKMs[(3)]	RANK[(4)]
1426	MGCS36089_04194			-	hypothetical protein	53.0	1424
1427	MGCS36089_04004			-	IS982 family transposase	52.8	1427
1428	MGCS36089_02082			-	DUF3173 family protein	52.5	1428
1429	MGCS36089_03128			*asnA*	asparagine synthetase AsnA	52.5	1428
1430	MGCS36089_03892			-	LytTR family transcriptional regulator	52.5	1428
1431	MGCS36089_01712			*trxB_1*	NAD(P)/FAD-dependent oxidoreductase	52.0	1431
1432	MGCS36089_02986			*focA*	formate transporter FocA	52.0	1431
1433	MGCS36089_01230			*rnc*	ribonuclease III Rnc	51.8	1433
1434	MGCS36089_02604			-	hypothetical protein	51.8	1433
1435	MGCS36089_02670			*malA*	maltodextrose utilization protein MalA	51.8	1433
1436	MGCS36089_03708			-	PurR family transcriptional regulator	51.8	1433
1437	MGCS36089_03890			-	hypothetical protein	51.8	1433
1438	MGCS36089_01298			-	PrtC family collagenase-like protease	51.5	1438
1439	MGCS36089_02344			*fieF*	FieF family cation diffusion facilitator family	51.5	1438
1440	MGCS36089_03766			-	type IV secretion system DNA-binding	51.5	1438
1441	MGCS36089_03804			*oppC_2*	oligopeptide ABC transporter permease OppC	51.5	1438
1442	MGCS36089_00454			-	type II toxin-antitoxin system PemK/MazF family	51.3	1442
1443	MGCS36089_00944	Lipo		*fhuD*	iron-hydroxamate ABC transporter	51.3	1442
1444	MGCS36089_01858			-	XRE family HTH-type transcriptional regulator	51.3	1442
1445	MGCS36089_01688			*carA*	carbamoyl-phosphate synthase small subunit CarA	51.0	1445
1446	MGCS36089_02480			-	sakacin P family class II bacteriocin	51.0	1445
1447	MGCS36089_03904			-	ABC transporter ATPase/permease	51.0	1445
1448	MGCS36089_04276			*yfhO*	YfhO family protein	51.0	1445
1449	MGCS36089_02122			-	hypothetical protein	50.8	1449
1450	MGCS36089_02584	Lipo		-	putative lipoprotein	50.8	1449
1451	MGCS36089_02660			-	IS30 family transposase	50.8	1449
1452	MGCS36089_00560			-	major facilitator transporter family protein	50.3	1452
1453	MGCS36089_01864			*copY_1*	copper transport repressor CopY	50.3	1452
1454	MGCS36089_02018			-	vicinal oxygen chelate (VOC) family protein	50.3	1452
1455	MGCS36089_02956	Secreted		*plsC*	secreted 1-acyl-sn-glycerol-3-phosphate	50.3	1452
1456	MGCS36089_02868			-	beta-D-glucuronidase	50.0	1456
1457	MGCS36089_03228			*ugpA_1*	sugar ABC transporter permease UgpA	50.0	1456
1458	MGCS36089_03330			*atoB*	3-ketoacyl-CoA thiolase/acetyl-CoA	50.0	1456
1459	MGCS36089_04208			-	DUF4097 family protein	50.0	1456
1460	MGCS36089_01482			*rodA*	rod shape-determining protein RodA	49.8	1460
1461	MGCS36089_03612			-	ECF transporter (S) specificity component	49.5	1461
1462	MGCS36089_00286			-	1,4-dihydroxy-2-naphthoate octaprenyltransferase	49.3	1462
1463	MGCS36089_01582	Lipo		-	ABC transporter substrate-binding lipoprotein	48.8	1463
1464	MGCS36089_01768			-	HTH domain-containing putative transcriptional	48.8	1463
1465	MGCS36089_03106			-	SDR family oxidoreductase	48.8	1463
1466	MGCS36089_02528			*cas9*	type II CRISPR RNA-guided endonuclease Cas9	48.3	1466
1467	MGCS36089_00534			-	hypothetical protein	48.0	1467

No.	Locus tag	Signal6P predicted[(1)]	Virulence[(2)]	Gene	Function	RPKMs[(3)]	RANK[(4)]
1468	MGCS36089_01808			-	hypothetical protein	48.0	1467
1469	MGCS36089_01828			-	type IV toxin-antitoxin system AbiEi family	48.0	1467
1470	MGCS36089_01836			-	SNF2-related protein	48.0	1467
1471	MGCS36089_03424			-	hypothetical protein	48.0	1467
1472	MGCS36089_02182			-	SatD family protein	47.8	1472
1473	MGCS36089_00456			-	aminoglycoside 6-adenylyltransferase	47.5	1473
1474	MGCS36089_00526			*lrp*	PucR family transcriptional regulator/leucine	47.5	1473
1475	MGCS36089_00946			*fhuA*	ferrichrome ABC transporter ATP-binding protein	47.3	1475
1476	MGCS36089_00928			*yhcC*	YhcC family Fe-S oxidoreductase	47.0	1476
1477	MGCS36089_02300			*rsmF*	RsmF family rRNA cytosine-C5-methyltransferase	47.0	1476
1478	MGCS36089_03040			*feoA*	ferrous iron transport protein (A) FeoA	47.0	1476
1479	MGCS36089_03882		Virulence	*fasC*	TCS histidine kinase	47.0	1476
1480	MGCS36089_00430			-	ATP-binding cassette domain-containing protein	46.8	1480
1481	MGCS36089_00766			-	putative metal homeostasis protein	46.5	1481
1482	MGCS36089_03508			*cof*	Cof family hydrolase	46.5	1481
1483	MGCS36089_00660			-	conjugal transfer protein	46.3	1483
1484	MGCS36089_03214			*aroE*	shikimate dehydrogenase AroE	46.0	1484
1485	MGCS36089_00510			-	PreQ1 RNA	45.8	1485
1486	MGCS36089_01466			*gstA*	GstA superfamily glutathione-dependent	45.8	1485
1487	MGCS36089_02318			-	ABC transporter permease	45.8	1485
1488	MGCS36089_04186			-	MFS transporter	45.8	1485
1489	MGCS36089_01196			*dhaQ*	DhaKLM operon coactivator DhaQ	45.5	1489
1490	MGCS36089_01708			*rimM*	ribosome maturation factor RimM	45.5	1489
1491	MGCS36089_02320			-	ABC transporter ATP-binding protein LolD-like	45.3	1491
1492	MGCS36089_00018			-	RNA-binding S4 domain-containing protein	45.0	1492
1493	MGCS36089_03362			*ansP*	AnsP family L-asparagine transporter	45.0	1492
1494	MGCS36089_03646			*scrR*	sucrose operon repressor ScrR	45.0	1492
1495	MGCS36089_03814			-	YgaE family uncharacterized protein	45.0	1492
1496	MGCS36089_03996	Secreted		-	putative secreted protein	45.0	1492
1497	MGCS36089_03416			*ypbQ*	YpbQ family isoprenylcysteine carboxyl	44.8	1497
1498	MGCS36089_00892			-	SpoU family RNA methyltransferase	44.5	1498
1499	MGCS36089_02060			*dppF*	dipeptide ABC transport system ATP-binding	44.5	1498
1500	MGCS36089_03336			-	3-hydroxybutyrate dehydrogenase	44.5	1498
1501	MGCS36089_03906			-	ABC transporter ATPase/permease	44.5	1498
1502	MGCS36089_02408			-	GatA family amidase	44.3	1502
1503	MGCS36089_03920	Secreted	Virulence	*fbpB*	cell surface fibronectin binding protein (B). Cell-wall anchoring predicted sortase	44.3	1502
1504	MGCS36089_03970			*ulaG*	L-ascorbate utilization protein (G) UlaG	44.3	1502
1505	MGCS36089_03972			*bglG_3*	BglG family transcription antiterminator	44.3	1502
1506	MGCS36089_01690			*carB*	carbamoyl-phosphate synthase large subunit CarB	44.0	1506
1507	MGCS36089_02576			*phnK*	PhnK family uncharacterized ABC transporter	44.0	1506
1508	MGCS36089_02936			-	MFS transporter	44.0	1506
1509	MGCS36089_01272			*pstC*	phosphate ABC transporter, permease protein	43.8	1509

No.	Locus tag	Signal6P predicted[(1)]	Virulence[(2)]	Gene	Function	RPKMs[(3)]	RANK[(4)]
1510	MGCS36089_03510			-	SSRC34_2 RNA	43.8	1509
1511	MGCS36089_00846			*metP_1*	methionine ABC transporter permease MetP	43.5	1511
1512	MGCS36089_01474			*pheA*	chorismate mutase PheA	43.5	1511
1513	MGCS36089_02914			-	transcriptional regulator	43.3	1513
1514	MGCS36089_03592			-	cysteine hydrolase	43.3	1513
1515	MGCS36089_02156			-	polyprenyl synthetase family protein	43.0	1515
1516	MGCS36089_02414			*pyrE*	orotate phosphoribosyltransferase PyrE	43.0	1515
1517	MGCS36089_02418			-	PyrR RNA	43.0	1515
1518	MGCS36089_00392	Lipo		-	BMP family ABC transporter substrate-binding	42.8	1518
1519	MGCS36089_01958			*yjbK*	YbjK superfamily CYTH domain-containing	42.8	1518
1520	MGCS36089_02870			-	sugar kinase	42.8	1518
1521	MGCS36089_03506			*azgA*	AzgA family permease	42.8	1518
1522	MGCS36089_04218			-	helix-turn-helix domain-containing	42.8	1518
1523	MGCS36089_00844			*metN_1*	methionine ABC transporter ATP-binding protein	42.5	1523
1524	MGCS36089_01846			-	DUF5965 family protein	42.5	1523
1525	MGCS36089_02800			-	DUF1413 domain-containing protein	42.3	1525
1526	MGCS36089_03880		Virulence	*fasA*	TCS response regulator	42.3	1525
1527	MGCS36089_01810			-	CPBP family intramembrane metalloprotease	42.0	1527
1528	MGCS36089_03792			*rimI*	ribosomal-protein-alanine N-acetyltransferase	42.0	1527
1529	MGCS36089_00662			-	cytoplasmic protein	41.8	1529
1530	MGCS36089_01686			*pyrB*	aspartate transcarbomoylase PyrB	41.8	1529
1531	MGCS36089_02038			-	SalY superfamily ABC transporter permease	41.8	1529
1532	MGCS36089_04260			*pqqL*	pitrilysin family predicted Zn-dependent	41.8	1529
1533	MGCS36089_04272			*yitT*	putative membrane anchor protein YitT	41.8	1529
1534	MGCS36089_01724			-	putative peptidoglycan hydrolase	41.5	1534
1535	MGCS36089_03732			-	BaeS family TCS sensor histidine kinase	41.5	1534
1536	MGCS36089_00996			-	PhnB family glyoxalase/bleomycin	41.3	1536
1537	MGCS36089_00162			-	MATE family multidrug efflux transporter	41.0	1537
1538	MGCS36089_01252			*bglF*	PTS beta-glucoside transporter IIBCA component	41.0	1537
1539	MGCS36089_01824			-	phage tail tip lysozyme	41.0	1537
1540	MGCS36089_01842			-	hypothetical protein	41.0	1537
1541	MGCS36089_03778			-	MerR/SoxR family transcriptional regulator	41.0	1537
1542	MGCS36089_02596			*yloA*	YloA family predicted ribosome quality control	40.8	1542
1543	MGCS36089_02226			*asd*	aspartate-semialdehyde dehydrogenase	40.5	1543
1544	MGCS36089_03734			-	OmpR family TCS DNA-binding response regulator	40.3	1544
1545	MGCS36089_01568			-	YbgA family DUF1722 domain-containing protein	40.0	1545
1546	MGCS36089_01848			-	DUF5945 family protein	40.0	1545
1547	MGCS36089_02938			*rsuA_2*	ribosomal small subunit pseudouridine synthase	40.0	1545
1548	MGCS36089_03014	Secreted		-	putative extracellular cell surface	39.8	1548
1549	MGCS36089_00294			*cydD*	thiol reductant ABC exporter subunit CydD	39.0	1549
1550	MGCS36089_00296			*cdyC*	thiol reductant ABC exporter subunit CydC	39.0	1549
1551	MGCS36089_00420			-	hypothetical protein	39.0	1549

No.	Locus tag	Signal6P predicted[(1)]	Virulence[(2)]	Gene	Function	RPKMs[(3)]	RANK[(4)]
1552	MGCS36089_03244			*mngB*	alpha-mannosidase MngB	38.8	1552
1553	MGCS36089_02214			-	Uup family ATPase components of ABC transporters	38.3	1553
1554	MGCS36089_03226			*ugpE_1*	glycerol-3-phosphate ABC transporter permease	38.3	1553
1555	MGCS36089_03522			-	acyltransferase family protein	38.3	1553
1556	MGCS36089_03806			*oppB_2*	oligopeptide ABC transporter permease OppB	38.3	1553
1557	MGCS36089_01806			-	arsenate reductase	38.0	1557
1558	MGCS36089_02158			-	NAD(P)/FAD-dependent oxidoreductase	37.8	1558
1559	MGCS36089_02798			-	ORF6N domain-containing protein	37.8	1558
1560	MGCS36089_02946	Lipo		*blaA*	beta-lactamase-related serine hydrolase	37.8	1558
1561	MGCS36089_01264			-	cupin domain-containing protein	37.3	1561
1562	MGCS36089_01322			-	GH25 muramidase superfamily lysozyme	37.3	1561
1563	MGCS36089_01852			-	DUF5960 family protein	37.3	1561
1564	MGCS36089_02540			-	hypothetical protein	37.3	1561
1565	MGCS36089_03934			*resA*	TlpA family protein disulfide reductase	37.0	1565
1566	MGCS36089_04234			-	Spd-sr37 RNA	37.0	1565
1567	MGCS36089_04262			*ybcJ*	ribosome associated protein YbcJ	37.0	1565
1568	MGCS36089_03298			-	YIH1 family putative translation regulator	36.8	1568
1569	MGCS36089_00670	Pilin		-	putative lipoprotein	36.5	1569
1570	MGCS36089_02416			*pyrF*	orotidine-5'-phosphate decarboxylase PyrF	36.5	1569
1571	MGCS36089_01820			-	PrgI family protein	36.3	1571
1572	MGCS36089_02740	Lipo		*nlpA*	MetQ/NlpA family ABC transporter	36.3	1571
1573	MGCS36089_01822			-	AAA family ATPase	36.0	1573
1574	MGCS36089_02928			-	GNAT family N-acetyltransferase	36.0	1573
1575	MGCS36089_03036			-	FeoB associated cysteine-rich protein	36.0	1573
1576	MGCS36089_00942			*fhuB*	iron ABC transporter permease FhuB	35.8	1576
1577	MGCS36089_02102			-	hypothetical protein	35.8	1576
1578	MGCS36089_00828			-	MerR family transcriptional regulator	35.5	1578
1579	MGCS36089_00120			*purM*	phosphoribosylformylglycinamidine cyclo-ligase	35.3	1579
1580	MGCS36089_00920			*trml*	TrmL family tRNA (cytosine34-2'-O-)-methyl	35.3	1579
1581	MGCS36089_02736			*paaI*	PaaI family thioesterase	35.3	1579
1582	MGCS36089_03902			*queH*	epoxyqueuosine reductase QueH	35.3	1579
1583	MGCS36089_00418			-	MccC family LD-carboxypeptidase	35.0	1583
1584	MGCS36089_00620			-	DNA cytosine methyltransferase	35.0	1583
1585	MGCS36089_01940			-	NanC-like SGNH/GDSL hydrolase family protein	35.0	1583
1586	MGCS36089_03950			-	CHY zinc finger domain-containing protein	35.0	1583
1587	MGCS36089_04162			-	cadmium efflux system accessory protein	35.0	1583
1588	MGCS36089_00136			-	DUF4041 domain-containing protein	34.5	1588
1589	MGCS36089_03622	Lipo		*htsA*	heme ABC transporter substrate-binding	34.3	1589
1590	MGCS36089_04188			-	arginase family protein	34.3	1589
1591	MGCS36089_02100			-	hypothetical protein	34.0	1591
1592	MGCS36089_02714			-	hypothetical protein	34.0	1591
1593	MGCS36089_02746			-	hypothetical protein	34.0	1591

No.	Locus tag	Signal6P predicted[(1)]	Virulence[(2)]	Gene	Function	RPKMs[(3)]	RANK[(4)]
1594	MGCS36089_00016			-	oligosaccharide flippase family protein	33.8	1594
1595	MGCS36089_01928			*maeR*	TCS signal transduction response regulator MaeR	33.5	1595
1596	MGCS36089_00940			*fhuG*	iron ABC transporter permease FhuG	33.3	1596
1597	MGCS36089_01274			*pstA*	phosphate ABC transporter, permease protein	33.3	1596
1598	MGCS36089_03878		Virulence	*fasX*	FasBCAX signal transduction system small RNA	33.3	1596
1599	MGCS36089_04092			*catE*	catechol-2,3-dioxygenase CatE	33.3	1596
1600	MGCS36089_00274			-	helix-turn-helix transcriptional regulator	33.0	1600
1601	MGCS36089_00850			*sstT*	serine/threonine transporter SstT	33.0	1600
1602	MGCS36089_01684			*pyrP*	uracil permease protein PyrP	33.0	1600
1603	MGCS36089_03044			-	amino acid ABC transporter permease	33.0	1600
1604	MGCS36089_02672			*malD*	maltodextrin transport system permease protein	32.8	1604
1605	MGCS36089_03192			-	MarR family winged helix-turn-helix	32.5	1605
1606	MGCS36089_01936			*eriC*	EriC family voltage gated chloride channel	32.3	1606
1607	MGCS36089_02578			-	putative ABC transporter permease component	32.3	1606
1608	MGCS36089_01844			-	TOPRIM (topoisomerase-primase) domain-containing protein	32.0	1608
1609	MGCS36089_02072			-	IS30 family transposase	32.0	1608
1610	MGCS36089_00138			-	hypothetical protein	31.8	1610
1611	MGCS36089_01378			-	oligohyaluronate lyase	31.8	1610
1612	MGCS36089_01802			-	DNA (cytosine-5-)-methyltransferase	31.8	1610
1613	MGCS36089_03042			-	amino acid ABC transporter ATP-binding protein	31.8	1610
1614	MGCS36089_01428			-	DUF1003 domain-containing protein	31.5	1614
1615	MGCS36089_02068	Lipo		*dppA*	dipeptide-binding ABC transport system	31.5	1614
1616	MGCS36089_02990			*niaR*	niacin-responsive transcriptional repressor	31.5	1614
1617	MGCS36089_04206			-	DUF1700 domain-containing protein	31.5	1614
1618	MGCS36089_00790	Secreted		*dacA_1*	secreted D-alanyl-D-alanine carboxypeptidase	31.3	1618
1619	MGCS36089_02150			-	1,4-dihydroxy-2-naphthoate polyprenyltransferase	31.3	1618
1620	MGCS36089_02638			*miaA*	tRNA (adenosine(37)-N6)-dimethylallyltransferase	31.3	1618
1621	MGCS36089_03624	Secreted		*shp*	heme-binding secreted protein Shp.Cell-wall anchoring predicted sortase	31.3	1618
1622	MGCS36089_04030			*mutT*	8-oxo-dGTP diphosphatase, DNA mismatch repair	31.3	1618
1623	MGCS36089_00014			*trcF*	transcription-repair coupling factor TcrF	31.0	1623
1624	MGCS36089_00422			*proX*	prolyl-tRNA synthetase associated	31.0	1623
1625	MGCS36089_02354			*phnA*	PnhA family zinc ribbon domain-containing	31.0	1623
1626	MGCS36089_03948			*bioY*	biotin transporter BioY	31.0	1623
1627	MGCS36089_01276			*pstB*	phosphate ABC transporter ATP-binding protein	30.8	1627
1628	MGCS36089_02958			*trmN6*	tRNA1(Val) A37 N6-methylase TrmN6	30.5	1628
1629	MGCS36089_02728			-	MFS transporter	30.0	1629
1630	MGCS36089_00652			-	replication initiation factor domain-containing	29.8	1630
1631	MGCS36089_01580			*aspB*	aspartate aminotransferase AspB	29.8	1630
1632	MGCS36089_02744			*dcm*	DNA (cytosine-5-)-methyltransferase Dcm	29.8	1630
1633	MGCS36089_04164			-	DNA-binding HTH domain-containing	29.8	1630
1634	MGCS36089_00506			-	ECF transporter S component	29.5	1634
1635	MGCS36089_00460			-	helix-turn-helix transcriptional regulator	29.0	1635

No.	Locus tag	Signal6P predicted[(1)]	Virulence[(2)]	Gene	Function	RPKMs[(3)]	RANK[(4)]
1636	MGCS36089_01154			-	hypothetical protein	29.0	1635
1637	MGCS36089_02580			-	putative ABC transporter substrate binding	29.0	1635
1638	MGCS36089_01566			*osmC*	YhfA family OsmC-related salt-stress induced	28.5	1638
1639	MGCS36089_01976			*guaC*	guanosine 5'-monophosphate oxidoreductase GuaC	28.5	1638
1640	MGCS36089_00478			-	site-specific integrase	28.3	1640
1641	MGCS36089_00508			*rihB*	pyrimidine-specific ribonucleoside hydrolase	28.0	1641
1642	MGCS36089_01706	Secreted		*sdrD*	SdrD superfamily cell surface extracellular. Cell-wall anchoring predicted sortase	28.0	1641
1643	MGCS36089_02174			*citC*	citrate lyase ligase CitC	28.0	1641
1644	MGCS36089_02718			-	DUF4044 domain-containing protein	27.8	1644
1645	MGCS36089_00666			-	Cro/CI family transcriptional regulator	27.5	1645
1646	MGCS36089_00122			*purN*	phosphoribosylglycinamide formyltransferase	27.3	1646
1647	MGCS36089_02748			-	RepA N-terminal domain-containing protein	27.3	1646
1648	MGCS36089_02076			*kefB*	Kef-type K+ transporter membrane component KefB	26.8	1648
1649	MGCS36089_03204	Lipo		-	putative lipoprotein	26.8	1648
1650	MGCS36089_03586			-	CoA-disulfide reductase	26.8	1648
1651	MGCS36089_00320			*comYA*	competence system type II/IV secretion system	26.5	1651
1652	MGCS36089_03468		Virulence	-	YSIRK-targeted surface antigen transcriptional	26.5	1651
1653	MGCS36089_03730			-	OmpR family TCS DNA-binding response regulator	26.5	1651
1654	MGCS36089_01144	Secreted		-	secreted transglutaminase	26.3	1654
1655	MGCS36089_01250			*bglG_1*	transcriptional antiterminator BglG	26.3	1654
1656	MGCS36089_01982			*pbuX*	xanthine permease PbuX	26.3	1654
1657	MGCS36089_02188			-	IS982 family transposase	26.3	1654
1658	MGCS36089_03568			-	GlsB/YeaQ/YmgE family stress response membrane	26.3	1654
1659	MGCS36089_03626	Secreted	Virulence	*shr*	heme-binding secreted protein Shr. Cell-wall anchoring predicted sortase	26.3	1654
1660	MGCS36089_00348			-	DUF4651 domain-containing protein	26.0	1660
1661	MGCS36089_00628			-	ATP-binding protein	26.0	1660
1662	MGCS36089_03842			-	hypothetical protein	25.8	1662
1663	MGCS36089_02752			-	resolvase/recombinase family protein	25.5	1663
1664	MGCS36089_03724			-	Xre family helix-turn-helix transcriptional	25.3	1664
1665	MGCS36089_04200			-	thioredoxin family protein	25.3	1664
1666	MGCS36089_03614			-	CydD-related ABC transporter ATPase/permease	25.0	1666
1667	MGCS36089_04190			-	radical SAM protein	25.0	1666
1668	MGCS36089_01278			*phoU_1*	phosphate signaling complex protein PhoU	24.8	1668
1669	MGCS36089_03728			-	BaeS family TCS sensor histidine kinase	24.8	1668
1670	MGCS36089_00118			*purF*	amidophosphoribosyltransferase PurF	24.5	1670
1671	MGCS36089_03644			*scrB*	sucrose-6-phosphate hydrolase ScrB	24.5	1670
1672	MGCS36089_02166			-	ABC transporter permease	24.3	1672
1673	MGCS36089_02168			-	ABC transporter ATPase	24.3	1672
1674	MGCS36089_01930			*maeK*	TCS signal transduction sensor histidine kinase	24.0	1674
1675	MGCS36089_02092			-	XRE family HTH-type transcriptional regulator	24.0	1674
1676	MGCS36089_03198			-	hypothetical protein	23.8	1676
1677	MGCS36089_03590			-	DUF156 family protein	23.8	1676

No.	Locus tag	Signal6P predicted[(1)]	Virulence[(2)]	Gene	Function	RPKMs[(3)]	RANK[(4)]
1678	MGCS36089_00624			-	MvaI/BcnI family restriction endonuclease	23.5	1678
1679	MGCS36089_00626			-	SIR2 family protein	23.3	1679
1680	MGCS36089_01152			-	ABC transporter ATP-binding protein	23.0	1680
1681	MGCS36089_03786			-	hypothetical protein	23.0	1680
1682	MGCS36089_01438	Secreted		*adcA*	zinc ABC transporter secreted substrate-binding	22.8	1682
1683	MGCS36089_02750			-	hypothetical protein	22.8	1682
1684	MGCS36089_00412			-	hypothetical protein	22.5	1684
1685	MGCS36089_01944			*radC*	DNA repair protein RadC	22.5	1684
1686	MGCS36089_00438			-	bacteriocin immunity protein	22.3	1686
1687	MGCS36089_02034			*acrA*	AcrA superfamily multidrug efflux pump	22.3	1686
1688	MGCS36089_02036			-	LolD superfamily ABC transporter ATP-binding	22.3	1686
1689	MGCS36089_04160			*cadD_2*	CadD family cadmium resistance transporter	22.3	1686
1690	MGCS36089_00134			*purK*	5-(carboxyamino)imidazole ribonucleotide	22.0	1690
1691	MGCS36089_02062			*dppD*	dipeptide ABC transport system ATP-binding	22.0	1690
1692	MGCS36089_03780			*dnaQ*	DNA polymerase III epsilon subunit DnaQ	22.0	1690
1693	MGCS36089_04212			-	hypothetical protein	22.0	1690
1694	MGCS36089_00998			-	GloA family glyoxalase/bleomycin	21.8	1694
1695	MGCS36089_01892			-	Cro/CI family transcriptional regulator	21.8	1694
1696	MGCS36089_02738			-	nucleoside phosphorylase family protein	21.8	1694
1697	MGCS36089_03620			*htsB*	heme ABC transporter permease HtsB	21.8	1694
1698	MGCS36089_01490			*serB*	phosphoserine phosphatase SerB	21.5	1698
1699	MGCS36089_01850			-	hypothetical protein	21.5	1698
1700	MGCS36089_03242			-	metal-independent alpha-mannosidase	21.5	1698
1701	MGCS36089_00382			*ridA*	RidA family protein	21.3	1701
1702	MGCS36089_01682			*pyrR*	bifunctional pyrimidine regulatory protein/uracil phosphoribosyltransferase PyrR	20.8	1702
1703	MGCS36089_03584			-	rhodanese-like domain-containing protein	20.8	1702
1704	MGCS36089_00664			-	hypothetical protein	20.5	1704
1705	MGCS36089_01104			-	hypothetical protein	20.5	1704
1706	MGCS36089_02152	Lipo		*apbE*	ApbE family thiamine biosynthesis lipoprotein	20.5	1704
1707	MGCS36089_02988			*niaX*	niacin transporter NiaX	20.5	1704
1708	MGCS36089_03760			-	hypothetical protein	20.5	1704
1709	MGCS36089_00848			*brnQ_1*	branched-chain amino acid transport system II	20.3	1709
1710	MGCS36089_02342			*alsT*	sodium:alanine symporter family protein	20.3	1709
1711	MGCS36089_04144			-	Uup family of ABC transporter with duplicated	20.3	1709
1712	MGCS36089_00116			*purL*	phosphoribosylformylglycinamidine synthase PurL	20.0	1712
1713	MGCS36089_01804			-	hypothetical protein	20.0	1712
1714	MGCS36089_03616			-	CydC-related ABC transporter ATPase/permease	19.8	1714
1715	MGCS36089_03762			-	HTH cro/C1-type domain-containing protein	19.8	1714
1716	MGCS36089_03998			-	MdlB family ABC transporter ATP-binding/permease	19.8	1714
1717	MGCS36089_00644			-	conjugal transfer protein	19.5	1717
1718	MGCS36089_02064			*dppC*	dipeptide ABC transport system permease protein	19.5	1717
1719	MGCS36089_02322			*tetR*	TetR family transcriptional regulator	19.5	1717

No.	Locus tag	Signal6P predicted(1)	Virulence(2)	Gene	Function	RPKMs(3)	RANK(4)
1720	MGCS36089_01198			*dhaS*	dihydroxyacetone kinase transcriptional	19.3	1720
1721	MGCS36089_04108			-	hypothetical protein	19.3	1720
1722	MGCS36089_00012			*pth*	aminoacyl-tRNA hydrolase Pth	19.0	1722
1723	MGCS36089_03422			*nptA*	NtpA family Na/Pi cotransporter	19.0	1722
1724	MGCS36089_01790			-	L10_leader RNA	18.8	1724
1725	MGCS36089_02104			-	helix-turn-helix domain-containing protein	18.8	1724
1726	MGCS36089_02160			-	Gx transporter family protein	18.8	1724
1727	MGCS36089_03246			-	ParB-like nuclease domain-containing protein	18.8	1724
1728	MGCS36089_00436			-	Maf family protein	18.5	1728
1729	MGCS36089_01370			-	SSRC10 RNA	18.5	1728
1730	MGCS36089_00444			*flaR*	DNA topology modulation protein	18.0	1730
1731	MGCS36089_00646			-	conjugal transfer protein	18.0	1730
1732	MGCS36089_03588			-	rhodanese-like domain-containing protein	18.0	1730
1733	MGCS36089_00124			*vanZ*	VanZ family putative glycopeptide antibiotic	17.8	1733
1734	MGCS36089_00404			*celB_1*	PTS transporter cellobiose-specific IIC	17.8	1733
1735	MGCS36089_00648			-	conjugal transfer protein	17.8	1733
1736	MGCS36089_00722			-	aspartate kinase	17.8	1733
1737	MGCS36089_03472	Secreted		*aes*	Aes family secreted acetyl esterase/lipase	17.8	1733
1738	MGCS36089_04196			-	Rgg/GadR/MutR family transcriptional regulator	17.8	1733
1739	MGCS36089_02678	Lipo		*malX*	maltose/maltodextrin-binding lipoprotein MalX	17.5	1739
1740	MGCS36089_03698			-	alpha-glycosidase	17.5	1739
1741	MGCS36089_03726	Lipo		-	chromosome assembly-related lipoprotein	17.3	1741
1742	MGCS36089_03908			-	MarR family transcriptional regulator	17.3	1741
1743	MGCS36089_04086			-	Spy491738 RNA	17.3	1741
1744	MGCS36089_01406			-	transposase	17.0	1744
1745	MGCS36089_01978			-	Purine RNA	17.0	1744
1746	MGCS36089_03208			-	hypothetical protein	17.0	1744
1747	MGCS36089_03328			-	LysR family transcriptional regulator	17.0	1744
1748	MGCS36089_00402			*celA_1*	PTS transporter cellobiose-specific IIB	16.8	1748
1749	MGCS36089_00338			-	helix-turn-helix transcriptional regulator	16.3	1749
1750	MGCS36089_03002			-	Rgg/GadR/MutR family transcriptional regulator	16.3	1749
1751	MGCS36089_03232			-	COG3537 superfamily putative	16.3	1749
1752	MGCS36089_03618			*htsC*	heme ABC transporter ATP-binding protein HtsC	16.3	1749
1753	MGCS36089_04210			-	YhgE/Pip domain-containing protein	16.3	1749
1754	MGCS36089_00262			-	FRG domain-containing protein	16.0	1754
1755	MGCS36089_00658			-	FtsK/SpoIIIE domain-containing protein	16.0	1754
1756	MGCS36089_00130			*purD*	phosphoribosylamine--glycine ligase PurD	15.8	1756
1757	MGCS36089_02066			*dppB*	dipeptide ABC transport system permease protein	15.8	1756
1758	MGCS36089_00468			-	hypothetical protein	15.5	1758
1759	MGCS36089_03248			*yncA*	YncA family L-amino acid N-acyltransferase	15.5	1758
1760	MGCS36089_00132			*purE*	5-(carboxyamino)imidazole ribonucleotide mutase	15.0	1760
1761	MGCS36089_00650			-	putative conjugal transfer protein	15.0	1760

No.	Locus tag	Signal6P predicted[(1)]	Virulence[(2)]	Gene	Function	RPKMs[(3)]	RANK[(4)]
1762	MGCS36089_01980			*xpt*	xanthine phosphoribosyltransferase Xpt	15.0	1760
1763	MGCS36089_02050	Secreted	Virulence	*scpA*	cell surface extracellular C5a peptidase ScpA	15.0	1760
1764	MGCS36089_03156			*msrA/B*	Peptide methionine sulfoxide reductase	15.0	1760
1765	MGCS36089_03714			-	putative sulfite exporter	15.0	1760
1766	MGCS36089_01862			*cadD_1*	CadD family cadmium resistance transporter	14.8	1766
1767	MGCS36089_03234			-	hypothetical protein	14.5	1767
1768	MGCS36089_03850			-	cysteine hydrolase	14.5	1767
1769	MGCS36089_04008	Secreted	Virulence	*slo*	secreted cholesterol-dependent cytolysin	14.5	1767
1770	MGCS36089_04088			-	hypothetical protein	14.5	1767
1771	MGCS36089_00792			*dacA_2*	D-alanyl-D-alanine carboxypeptidase DacA	14.3	1771
1772	MGCS36089_02170			-	S41 family peptidase	14.3	1771
1773	MGCS36089_02222			*norG*	PLP-dependent aminotransferase family protein	14.3	1771
1774	MGCS36089_03350			*drpA*	DNA processing protein (A) DprA	14.3	1771
1775	MGCS36089_04084			*treR*	trehalose operon repressor TreR	14.3	1771
1776	MGCS36089_00842			*dapE*	M20/M25/M40 family metallo-hydrolase,	14.0	1776
1777	MGCS36089_01876			-	DUF5960 family protein	14.0	1776
1778	MGCS36089_04006	Secreted		-	TrbC/VirB2-related secreted effector protein	14.0	1776
1779	MGCS36089_04090			*yybR*	putative HTH-type transcriptional regulator	14.0	1776
1780	MGCS36089_02676			*amyB*	cyclomaltodextrinase protein AmyB	13.8	1780
1781	MGCS36089_02966	Lipo		*metQ*	methionine uptake ABC transporter	13.8	1780
1782	MGCS36089_00446			-	GNAT family N-acetyltransferase	13.5	1782
1783	MGCS36089_00474			-	Rep family protein	13.5	1782
1784	MGCS36089_01012	Secreted		*yoaK*	putative secreted YoaK family protein	13.5	1782
1785	MGCS36089_02098			-	hypothetical protein	13.5	1782
1786	MGCS36089_03004			-	MFS transporter	13.5	1782
1787	MGCS36089_02090			-	hypothetical protein	13.3	1787
1788	MGCS36089_03372	Secreted		*hisJ*	HisJ family amino acid ABC transporter	13.3	1787
1789	MGCS36089_01024			-	hypothetical protein	13.0	1789
1790	MGCS36089_02590			-	hypothetical protein	13.0	1789
1791	MGCS36089_03078			-	putative secreted protein. SignalP-6 predicted standard secretion signal	13.0	1789
1792	MGCS36089_00354			*ssb_1*	single-stranded DNA-binding protein Ssb	12.8	1792
1793	MGCS36089_00640			-	DNA segregation ATPase, conjugal transfer	12.8	1792
1794	MGCS36089_04170			-	FtsK/SpoIIIE domain-containing protein	12.8	1792
1795	MGCS36089_04172			-	hypothetical protein	12.8	1792
1796	MGCS36089_01098			-	hypothetical protein	12.5	1796
1797	MGCS36089_02024			*lmb_2*	bifunctional metal ABC transporter substrate-binding lipoprotein/laminin-binding lipoprotein Lmb	12.5	1796
1798	MGCS36089_00472			-	FtsK/SpoIIIE domain-containing protein	12.3	1798
1799	MGCS36089_02026	Lipo		-	streptococcal histidine triad (HIT) lipoprotein.	12.3	1798
1800	MGCS36089_02176			*oadA*	oxaloacetate decarboxylase subunit alpha OadA	12.0	1800
1801	MGCS36089_04202			-	NUDIX hydrolase	12.0	1800
1802	MGCS36089_02712			*pnuC*	nicotinamide riboside transporter PnuC	11.5	1802
1803	MGCS36089_03158	Lipo		*tipA*	TlpA family protein disulfide reductase	11.5	1802

No.	Locus tag	Signal6P predicted[(1)]	Virulence[(2)]	Gene	Function	RPKMs[(3)]	RANK[(4)]
1804	MGCS36089_04012	Secreted	Virulence	*nga*	secreted nicotine adenine dinucleotide	11.5	1802
1805	MGCS36089_00684			-	toxin-antitoxin system, toxin component, Fic	11.3	1805
1806	MGCS36089_00840	Lipo		-	MetQ/NlpA family ABC transporter	11.3	1805
1807	MGCS36089_01248			-	Spy490483c RNA	11.3	1805
1808	MGCS36089_01938			*araC*	AraC family transcriptional regulator	11.3	1805
1809	MGCS36089_02032	Lipo		-	ABC transporter protein	11.3	1805
1810	MGCS36089_03888			-	MefA-related MFS transporter	11.3	1805
1811	MGCS36089_02022			*lmb_1*	bifunctional metal ABC transporter substrate-binding lipoprotein/laminin-binding lipoprotein Lmb	11.0	1811
1812	MGCS36089_03230			*nagC*	NagC family sugar kinase	11.0	1811
1813	MGCS36089_04010			*ifs*	nicotine adenine dinucleotide glycohydrolase	11.0	1811
1814	MGCS36089_00638			-	conjugal transfer protein	10.8	1814
1815	MGCS36089_02674			*malC*	maltodextrin transport system permease protein	10.8	1814
1816	MGCS36089_02970			*metN_2*	methionine uptake ABC transporter ATP-binding	10.8	1814
1817	MGCS36089_04040			-	cspA RNA	10.8	1814
1818	MGCS36089_04168			-	hypothetical protein	10.8	1814
1819	MGCS36089_04174			-	replication protein	10.8	1814
1820	MGCS36089_00434			-	hypothetical protein	10.5	1820
1821	MGCS36089_00622			-	helix-turn-helix transcriptional regulator	10.5	1820
1822	MGCS36089_02762			-	PEP-utilizing enzyme	10.5	1820
1823	MGCS36089_02968			*metP_2*	methionine uptake ABC transporter permease MetP	10.5	1820
1824	MGCS36089_04176			-	MerR family transcriptional regulator	10.5	1820
1825	MGCS36089_02052			-	SSRC38 RNA	10.3	1825
1826	MGCS36089_00922			-	FMN RNA	10.0	1826
1827	MGCS36089_01304			-	NAD(P)-binding domain-containing protein	10.0	1826
1828	MGCS36089_00642			-	conjugal transfer protein	9.8	1828
1829	MGCS36089_00674			-	XRE family transcriptional regulator	9.8	1828
1830	MGCS36089_03704			-	carbohydrate ABC transporter permease UgpE-like	9.8	1828
1831	MGCS36089_00282			-	Spy392987 RNA	9.5	1831
1832	MGCS36089_00284			-	NUDIX domain-containing protein	9.5	1831
1833	MGCS36089_03160			*ccdA*	putative cytochrome c-type biogenesis protein	9.5	1831
1834	MGCS36089_03456			-	IS982 family transposase	9.5	1831
1835	MGCS36089_00470			-	hypothetical protein	9.3	1835
1836	MGCS36089_00476			-	DUF771 domain-containing protein	9.3	1835
1837	MGCS36089_00686			-	hypothetical protein	9.3	1835
1838	MGCS36089_03706			-	carbohydrate ABC transporter permease UgpA-like	9.3	1835
1839	MGCS36089_04166			-	sigma-70 family RNA polymerase sigma factor	9.3	1835
1840	MGCS36089_00636			-	putative conjugal transfer protein	9.0	1840
1841	MGCS36089_02020			-	hypothetical protein. SignalP-6 predicted lipid anchoring signal peptide,	9.0	1840
1842	MGCS36089_02194			*dprA*	DNA-processing protein DprA	9.0	1840
1843	MGCS36089_02760			-	MATE family efflux transporter	9.0	1840
1844	MGCS36089_00128	Secreted		-	CHAP domain-containing protein	8.8	1844
1845	MGCS36089_00616			-	bacterial mobilization protein	8.8	1844

No.	Locus tag	Signal6P predicted[(1)]	Virulence[(2)]	Gene	Function	RPKMs[(3)]	RANK[(4)]
1846	MGCS36089_02108			-	hypothetical protein	8.8	1844
1847	MGCS36089_02200			-	MFS transporter	8.8	1844
1848	MGCS36089_01872			-	TIGR01906 family membrane protein	8.5	1848
1849	MGCS36089_03788			*rpsN2*	30S ribosomal S14-2 protein RpsN2	8.5	1848
1850	MGCS36089_03926			-	hypothetical protein	8.5	1848
1851	MGCS36089_00416			-	hypothetical protein	8.3	1851
1852	MGCS36089_04000			-	hypothetical protein	8.3	1851
1853	MGCS36089_00268			-	Rep family plasmid replication protein	8.0	1853
1854	MGCS36089_00720			*comX_1*	competence protein ComX	8.0	1853
1855	MGCS36089_00808			*comX_2*	competence protein ComX	8.0	1853
1856	MGCS36089_00464			-	hypothetical protein	7.8	1856
1857	MGCS36089_00614			-	DUF5962 domain-containing protein	7.8	1856
1858	MGCS36089_02934			*coiA*	competence protein CoiA	7.8	1856
1859	MGCS36089_04204			*padR*	PadR family transcriptional regulator	7.8	1856
1860	MGCS36089_01138			-	CAAX amino terminal protease family protein	7.5	1860
1861	MGCS36089_02116			-	site-specific integrase	7.5	1860
1862	MGCS36089_01680			-	PyrR RNA	7.3	1862
1863	MGCS36089_01764	Secreted		-	extracellular cell wall anchored mucin-binding. Cell-wall anchoring predicted sortase	7.3	1862
1864	MGCS36089_03754			-	hypothetical protein	7.3	1862
1865	MGCS36089_00144			-	quorum-sensing system DWW-type pheromone	7.0	1865
1866	MGCS36089_00400			*celC_1*	PTS transporter cellobiose-specific IIA	7.0	1865
1867	MGCS36089_00612			-	phage replisome organizer N-terminal	7.0	1865
1868	MGCS36089_00678			-	lantibiotic dehydratase	7.0	1865
1869	MGCS36089_00618			-	transposase	6.8	1869
1870	MGCS36089_00682			-	lantibiotic transporter	6.8	1869
1871	MGCS36089_00632			-	helix-turn-helix domain-containing protein	6.5	1871
1872	MGCS36089_00634	Secreted		-	CHAP domain-containing protein	6.5	1871
1873	MGCS36089_00676			-	bacteriocin	6.5	1871
1874	MGCS36089_02106			-	hypothetical protein	6.5	1871
1875	MGCS36089_02756			-	pemK RNA	6.5	1871
1876	MGCS36089_03974			*ulaF*	L-ribulose-5-phosphate 4-epimerase UlaF	6.5	1871
1877	MGCS36089_03976			*ulaE*	L-xylulose 5-phosphate 3-epimerase UlaE	6.5	1871
1878	MGCS36089_00142			-	helix-turn-helix domain-containing protein	6.3	1878
1879	MGCS36089_00462	Secreted		-	cell surface PgrA surface exclusion. Cell-wall anchoring predicted sortase	6.3	1878
1880	MGCS36089_00672			-	ImmA/IrrE family metallo-endopeptidase	6.3	1878
1881	MGCS36089_00680			-	lanthionine synthetase C family protein	6.3	1878
1882	MGCS36089_01102			-	Blp family class II bacteriocin with	6.3	1878
1883	MGCS36089_01136			-	hypothetical protein	6.3	1878
1884	MGCS36089_02530			-	tracrRNA RNA	6.3	1878
1885	MGCS36089_00838	Lipo		-	MetQ/NlpA family ABC transporter	6.0	1885
1886	MGCS36089_01134			-	putative bacteriocin with double-glycine leader	6.0	1885
1887	MGCS36089_01350			-	glycosyltransferase family 2 protein	6.0	1885

No.	Locus tag	Signal6P predicted[(1)]	Virulence[(2)]	Gene	Function	RPKMs[(3)]	RANK[(4)]
1888	MGCS36089_01874			-	hypothetical protein	6.0	1885
1889	MGCS36089_02872			*melB*	MelB family MFS transporter	6.0	1885
1890	MGCS36089_03928			-	hypothetical protein	6.0	1885
1891	MGCS36089_00466			-	hypothetical protein	5.8	1891
1892	MGCS36089_02074			*femX*	FemABX-like family peptidoglycan interpeptide	5.8	1891
1893	MGCS36089_02084			-	hypothetical protein	5.8	1891
1894	MGCS36089_03772			-	DUF3173 family protein	5.8	1891
1895	MGCS36089_03978			*ulaD*	3-keto-L-gulonate 6-phosphate decarboxylase	5.8	1891
1896	MGCS36089_03982			*ulaB*	ascorbate-specific PTS transporter EIIB protein	5.8	1891
1897	MGCS36089_00270			-	hypothetical protein	5.5	1897
1898	MGCS36089_01128			-	BlpM-like bacteriocin with double-glycine leader	5.5	1897
1899	MGCS36089_02096			-	hypothetical protein	5.5	1897
1900	MGCS36089_02110			-	Rep family protein	5.5	1897
1901	MGCS36089_03770			-	replication protein	5.5	1897
1902	MGCS36089_02056			*htpA*	streptococcal histidine triad (HIT)	5.3	1902
1903	MGCS36089_02114			-	DUF3173 family protein	5.3	1902
1904	MGCS36089_03722			-	Xre family helix-turn-helix transcriptional regulator	5.3	1902
1905	MGCS36089_03984			*ulaA*	ascorbate-specific PTS transporter EIIC protein	5.3	1902
1906	MGCS36089_01130			-	lactobin A/cerein 7B family class IIb	5.0	1906
1907	MGCS36089_01344			-	hypothetical protein	4.8	1907
1908	MGCS36089_02764			-	TetR/AcrR family transcriptional regulator	4.8	1907
1909	MGCS36089_00114			*purC*	phosphoribosylaminoimidazolesuccinocarboxamide	4.5	1909
1910	MGCS36089_00610			-	DNA-binding protein	4.5	1909
1911	MGCS36089_03296			*comFA*	ComFA family DNA/RNA helicase	4.5	1909
1912	MGCS36089_00696			*gatB_1*	PTS galactitol transporter IIB component GatB	4.3	1912
1913	MGCS36089_01520			-	preQ1-II RNA	4.3	1912
1914	MGCS36089_02086			-	FtsK/SpoIIIE domain-containing protein	4.3	1912
1915	MGCS36089_02088			-	hypothetical protein	4.3	1912
1916	MGCS36089_03512			-	SSRC34_1 RNA	4.3	1912
1917	MGCS36089_00322			*comYB*	competence system type II secretion system	4.0	1917
1918	MGCS36089_01126		Virulence	*silE*	streptococcal invasion locus pheromone	4.0	1917
1919	MGCS36089_01132			-	putative bacteriocin with double-glycine leader	4.0	1917
1920	MGCS36089_04002			-	hypothetical protein	4.0	1917
1921	MGCS36089_00272			-	hypothetical protein	3.8	1921
1922	MGCS36089_00700			-	triose-phosphate isomerase	3.8	1921
1923	MGCS36089_00702			-	YjbQ family protein	3.8	1921
1924	MGCS36089_01142			-	hypothetical protein	3.8	1921
1925	MGCS36089_01404			-	IS3 family transposase	3.8	1921
1926	MGCS36089_02054	Lipo	Virulence	*lmb_3*	bifunctional metal ABC transporter substrate-binding lipoprotein/laminin-binding lipoprotein Lmb.	3.8	1921
1927	MGCS36089_02768			-	relaxase	3.8	1921
1928	MGCS36089_03980			*ulaC*	ascorbate-specific PTS transporter EIIA protein	3.8	1921
1929	MGCS36089_00968			*livG*	branched-chain amino acid ABC transporter	3.5	1929

No.	Locus tag	Signal6P predicted(1)	Virulence(2)	Gene	Function	RPKMs(3)	RANK(4)
1930	MGCS36089_01026	Secreted		-	hypothetical protein	3.5	1929
1931	MGCS36089_00972			-	cystathionine beta-synthase (CBS)	3.3	1931
1932	MGCS36089_01306			-	ammonium transporter	3.3	1931
1933	MGCS36089_01346			-	hypothetical protein	3.3	1931
1934	MGCS36089_02754			-	hypothetical protein	3.3	1931
1935	MGCS36089_02878	Lipo	Virulence	*slr*	InlA-like streptococcal leucine rich lipoprotein	3.3	1931
1936	MGCS36089_00694			*gatC_1*	PTS galactitol transporter IIC component GatC	3.0	1936
1937	MGCS36089_00970			*livF*	branched-chain amino acid ABC transporter	3.0	1936
1938	MGCS36089_01348			-	glycosyltransferase	3.0	1936
1939	MGCS36089_01932			*maeP*	Citrate/malate symporter protein MaeP	3.0	1936
1940	MGCS36089_01934			*maeE*	NAD-dependent malic enzyme MaeE	3.0	1936
1941	MGCS36089_02094			-	hypothetical protein	3.0	1936
1942	MGCS36089_00266			-	DUF3173 domain-containing protein	2.8	1942
1943	MGCS36089_00326	Pilin		*comYD*	competence system type II secretion system GspH	2.8	1942
1944	MGCS36089_00330	Pilin		*comYF*	competence system protein ComYF	2.8	1942
1945	MGCS36089_00966			*livM*	branched-chain amino acid ABC transporter	2.8	1942
1946	MGCS36089_02594			-	hypothetical protein	2.8	1942
1947	MGCS36089_04184			-	parvulin-like peptidyl-prolyl isomerase	2.8	1942
1948	MGCS36089_00964			*livH*	branched-chain amino acid ABC transporter	2.5	1948
1949	MGCS36089_01096			-	hypothetical protein	2.5	1948
1950	MGCS36089_01762			-	HTH domain-containing putative transcriptional	2.5	1948
1951	MGCS36089_00328	Pilin		*comYE*	competence system protein ComYE	2.3	1951
1952	MGCS36089_00332			*comYG*	competence system protein ComYG	2.3	1951
1953	MGCS36089_00688			-	tyrosine-type site-specific	2.3	1951
1954	MGCS36089_00962	Lipo		*livK*	branched-chain amino acid ABC transporter	2.3	1951
1955	MGCS36089_01124		Virulence	*silD*	streptococcal invasion locus pheromone secretion	2.3	1951
1956	MGCS36089_01236			-	hypothetical protein. CW-Pred predicted sortase A cell-wall anchoring motif, position 247..252	2.3	1951
1957	MGCS36089_02952			*comEC*	DNA internalization-related competence protein	2.3	1951
1958	MGCS36089_03768			-	hypothetical protein	2.3	1951
1959	MGCS36089_00690			-	PRD domain/PTS transporter IIA domain protein	2.0	1959
1960	MGCS36089_02766			*mobC*	plasmid mobilization relaxosome protein MobC	2.0	1959
1961	MGCS36089_02954			*comEA*	DNA uptake competence protein ComEA	2.0	1959
1962	MGCS36089_00264			*xerC*	site-specific integrase	1.8	1962
1963	MGCS36089_00324	Pilin		-	competence system prepilin-type N-terminal	1.8	1962
1964	MGCS36089_02770			-	conjugative element protein	1.8	1962
1965	MGCS36089_01280	Secreted		-	hypothetical protein	1.3	1965
1966	MGCS36089_02776			*topB*	DNA topoisomerase III TopB	1.3	1965
1967	MGCS36089_02828			-	NTPase domain-containing protein	1.3	1965
1968	MGCS36089_02112			-	hypothetical protein	1.0	1968
1969	MGCS36089_02758			-	sigma-70 family RNA polymerase sigma factor like	1.0	1968
1970	MGCS36089_02772			-	DNA methyltransferase	1.0	1968
1971	MGCS36089_02784			*virB4*	type IV secretory pathway component VirB4	1.0	1968

No.	Locus tag	Signal6P predicted[(1)]	Virulence[(2)]	Gene	Function	RPKMs[(3)]	RANK[(4)]
1972	MGCS36089_02802			-	phage replisome organizer N-terminal	1.0	1968
1973	MGCS36089_02824			*virD4*	type IV secretory system conjugative DNA	1.0	1968
1974	MGCS36089_02826			-	DUF3801 domain-containing protein	1.0	1968
1975	MGCS36089_02778	Secreted		-	DUF4366 domain-containing predicted secreted	0.8	1975
1976	MGCS36089_02786			-	conjugal transfer protein	0.8	1975
1977	MGCS36089_02788			*virB6*	VirB6-like conjugal transfer protein	0.8	1975
1978	MGCS36089_02830			-	RepA N-terminal domain-containing protein	0.8	1975
1979	MGCS36089_01140			-	hypothetical protein	0.5	1979
1980	MGCS36089_02790			-	Maff2 family protein	0.5	1979
1981	MGCS36089_02792			-	single-stranded DNA-binding protein	0.5	1979
1982	MGCS36089_02794			*traG_2*	conjugal transfer protein TraG	0.5	1979
1983	MGCS36089_02782			-	CHAP domain-containing protein	0.3	1983
1984	MGCS36089_02808			-	sigma-70 family RNA polymerase sigma factor like	0.3	1983
1985	MGCS36089_02822			-	site-specific serine recombinase family protein	0.3	1983
1986	MGCS36089_01120		Virulence	*silCR*	streptococcal invasion locus auto-inducing pheromone peptide SilCR	0.0	1986
1987	MGCS36089_01122			*silC*	streptococcal invasion locus signaling peptide	0.0	1986
1988	MGCS36089_02780			-	conjugal transfer protein	0.0	1986
1989	MGCS36089_02796			*tnpW*	TnpW family transposon-encoded protein	0.0	1986
1990	MGCS36089_02804			*mobA*	MobA/MobL family protein	0.0	1986
1991	MGCS36089_02806			-	DUF3847 domain-containing protein	0.0	1986
1992	MGCS36089_02810			-	MdlB family multidrug ABC transporter ATPase and	0.0	1986
1993	MGCS36089_02812			-	MdlB family multidrug ABC transporter ATPase and	0.0	1986
1994	MGCS36089_02814			*ecfA2*	EcfA2 family ECF transporter ATPase	0.0	1986
1995	MGCS36089_02816			*ecfT*	ECF transporter transmembrane protein EcfT	0.0	1986
1996	MGCS36089_02818			-	ECF transporter S component	0.0	1986
1997	MGCS36089_02820			-	TetR/AcrR family transcriptional regulator	0.0	1986

MGCS36089 was grown *in vitro*, in rich media, and in quadruplicate. Genes were ranked by the mean transcript abundance calculated from four replicates, at early stationary (ES) growth phase (OD=2.0). Genes are ordered by transcript abundance rank, from highest (rank=1) to lowest.

(1) Genes predicted by SignalP 6.0 to have an export signal sequence.
(2) Virulence refers to putative virulence genes.
(3) RPKMs were assigned by EDGEPro.
(4) Rank refers to the corresponding transcript abundance rank based on RPKMs.
(5) Lipo, exported lipoprotein attached to the cell envelope.
(6) TCS, two-component system.

Table S3. Virulence gene expression of MGCS36089 grown *in vitro* during two growth phases

No.	Locus tag	Gene	Function	MGCS36089 ME RPKM(1)	MGCS36089 ES RPKM(2)	Fold ME/ES(3)
1	MGCS36089_03878	***fasX***	FasBCA regulatory RNA	551.2	33.25	**16.6**
2	MGCS36089_04008	***slo***	secreted cholesterol-dependent cytolysin	89.2	14.5	**6.2**
3	MGCS36089_00378	***fbp***	secreted fibronectin-binding protein	1177.5	202.2	**5.8**
4	MGCS36089_04012	***nga***	secreted nicotine adenine dinucleotide	62	11.5	**5.4**
5	MGCS36089_03880	***fasA***	TCS(4) response regulator	225	42.2	**5.3**
6	MGCS36089_02050	***scpA***	cell surface extracellular C5a peptidase	72.2	15	**4.8**
7	MGCS36089_00514	***mga***	M protein trans-acting positive regulator	373	77.7	**4.8**
8	MGCS36089_03882	***fasC***	TCS histidine kinase	205	47	**4.4**
9	MGCS36089_00516	***emm***	cell surface M protein	3092.5	753.2	**4.1**
10	MGCS36089_03626	***shr***	heme-binding secreted protein	88.7	26.2	**3.4**
11	MGCS36089_00932	***hlyX***	hemolysin family protein	365.2	118	**3.1**
12	MGCS36089_03310	***liaF***	LiaFSR membrane component protein	158.5	65	**2.4**
13	MGCS36089_04288	***htrA***	trypsin-like serine protease	1234	526.7	**2.3**
14	MGCS36089_03920	***fbpB***	cell surface fibronectin binding protein	98.5	44.2	**2.2**
15	MGCS36089_00866	***covS***	TCS sensor kinase CovS	567.7	265	**2.1**
16	MGCS36089_03712	***cppA***	CppA family putative C3-glycoprotein degrading protein	479.5	233	**2.1**
17	MGCS36089_03308	***liaS***	LiaFSR histidine kinase	238.7	122.2	**2.0**
18	MGCS36089_03306	***liaR***	LiaFSR response regulator	255.5	144	**1.8**
19	MGCS36089_00362	***rofA***	pilus transcriptional regulator	244.2	145	**1.7**
20	MGCS36089_02282	***ciaH***	TCS histidine kinase	468.5	286	**1.6**
21	MGCS36089_01124	***silD***	streptococcal invasion locus pheromone secretion accessory protein	3.5	2.25	**1.6**
22	MGCS36089_03938	***speG***	streptococcal pyrogenic exotoxin	149.25	103.25	**1.4**
23	MGCS36089_00864	***covR***	TCS DNA-binding response regulator	1093.75	781.75	**1.4**
24	MGCS36089_03154	***yesN***	TCS DNA-binding response regulator	104	78	**1.3**
25	MGCS36089_01048	***mtsR***	metal-dependent transcriptional regulator	415.25	318	**1.3**
26	MGCS36089_03152	***yesM***	TCS sensor kinase	137.75	107.25	**1.3**
27	MGCS36089_02040	***irr***	TCS response regulator	114.5	89.7	**1.3**
28	MGCS36089_02284	***ciaR***	TCS DNA-binding response regulator protein CiaR	353	282.2	**1.3**
29	MGCS36089_03220	***trxS***	TCS sensor histidine kinase TrxS	86.7	71	**1.2**
30	MGCS36089_01226	***vicK***	TCS signal transduction histidine kinase	561.7	479.2	**1.2**
31	MGCS36089_02852	***spg***	extracellular cell surface IgG-binding protein	3752	3261	**1.2**
32	MGCS36089_02042	***ihk***	TCS histidine kinase	112.7	98.7	**1.1**
33	MGCS36089_01126	***silE***	streptococcal invasion locus pheromone	4.5	4	**1.1**
34	MGCS36089_01224	***vicR***	TCS response regulator	445.2	423.7	**1.1**
35	MGCS36089_03218	***trxR***	TCS response regulator	99.2	101	**1.0**
36	MGCS36089_03956	***perR***	peroxide-responsive transcriptional repressor	779	871.7	**-1.1**
37	MGCS36089_01180	***ccpA***	catabolite control protein	478.2	541.7	**-1.1**
38	MGCS36089_00524	***ska***	secreted streptokinase	60.7	81.7	**-1.3**
39	MGCS36089_01496	***srrG***	streptolysin S small regulatory RNA	9151.7	12916.8	**-1.4**
40	MGCS36089_03820	***gapA***	glyceraldehyde-3-phosphate dehydrogenase	17053.3	31610.3	**-1.9**
41	MGCS36089_01112	***silA***	TCS response regulator	43.7	85.7	**-2.0**

No.	Locus tag	Gene	Function	MGCS36089 ME RPKM(1)	MGCS36089 ES RPKM(2)	Fold ME/ES(3)
42	MGCS36089_01516	***sagI***	streptolysin S export permease protein	1452.2	3041.7	**-2.1**
43	MGCS36089_01512	***sagG***	streptolysin S export protein	1206	2575.5	**-2.1**
44	MGCS36089_01514	***sagH***	streptolysin S export permease protein	1311.7	2849.2	**-2.2**
45	MGCS36089_01506	***sagD***	streptolysin S biosynthesis protein	1103.5	2416	**-2.2**
46	MGCS36089_01504	***sagC***	streptolysin S biosynthesis protein	1166.5	2750.7	**-2.4**
47	MGCS36089_01508	***sagE***	streptolysin S self-immunity protein	1213	2939	**-2.4**
48	MGCS36089_01398	***hylB***	secreted hyaluronate lyase	118	306.5	**-2.6**
49	MGCS36089_01510	***sagF***	streptolysin S biosynthesis protein	1179.5	3385.5	**-2.9**
50	MGCS36089_01502	***sagB***	streptolysin S biosynthesis protein	1317.7	6528.2	**-5.0**
51	MGCS36089_01498	***sagA***	streptolysin S precursor protein	3445.2	65244.3	**-18.9**
52	MGCS36089_01500	***sagA_RNA***	sagA RNA	4617.7	101242	**-21.9**

(1) RPKMs correspond to the mean of four replicates. ME, mid exponential phase
(2) ES, early stationary phase
(3) Fold was calculated dividing the mean RPKM value at mid-exponential phase by the mean RPKM value at early stationary phase
(4) TCS, two-component signal transduction system

Table S4A. Ranked MGCS36044 genes during *in vivo* infection of NHP 15

No.	Locus tag	SignalP6 predicted[1]	Virulence[2]	Gene	Function	RPKMs in NHP 15[3]	RANK[4]
1	MGCS36044_00482			*ssrS*	6S RNA	8616570	1
2	MGCS36044_02384			*ssrA*	transfer-messenger RNA	639848	2
3	MGCS36044_03334			-	RNaseP_bact_b RNA	611011	3
4	MGCS36044_01512		Virulence	*sagA_RNA*	sagA RNA	371794	4
5	MGCS36044_00484			*rarA*	replication-associated recombination protein A RarA	232868	5
6	MGCS36044_03776			*rpsN2*	30S ribosomal S14-2 protein RpsN2	217608	6
7	MGCS36044_02216			-	apolipoprotein A1/A4/E family protein	167426	7
8	MGCS36044_01510		Virulence	*sagA*	streptolysin S precursor SagA	126117	8
9	MGCS36044_02056	Lipo[5]		-	TlpA-family protein	123552	9
10	MGCS36044_01522		Virulence	*sagF*	streptolysin S biosynthesis protein SagF	121997	10
11	MGCS36044_01354			*tufA*	translation elongation factor Tu protein TufA	113443	11
12	MGCS36044_01520		Virulence	*sagE*	streptolysin S self-immunity protein SagE	103032	12
13	MGCS36044_02344			*yeaQ*	GlsB/YeaQ/YmgE family stress response membrane protein	102149	13
14	MGCS36044_02052	Secreted	Virulence	*isp*	secreted CHAP domain-containing immunogenic protein	95595	14
15	MGCS36044_01526		Virulence	*sagH*	streptolysin S export permease protein SagH	94131	15
16	MGCS36044_02054			-	hypothetical protein	92268	16
17	MGCS36044_01524		Virulence	*sagG*	streptolysin S export protein SagG	75230	17
18	MGCS36044_02046			-	SalY superfamily ABC transporter permease component	72114	18
19	MGCS36044_02342			*amaP*	alkaline shock response membrane anchor protein AmaP	69887	19
20	MGCS36044_01514		Virulence	*sagB*	streptolysin S biosynthesis protein SagB	65592	20
21	MGCS36044_01518		Virulence	*sagD*	streptolysin S biosynthesis protein SagD	62924	21
22	MGCS36044_01528		Virulence	*sagI*	streptolysin S export permease protein SagI	60765	22
23	MGCS36044_02670			*dltC*	D-alanine--poly(phosphoribitol) ligase subunit DltC	58748	23
24	MGCS36044_00752			*lrgB*	antiholin-like protein LrgB	58220	24
25	MGCS36044_03808		Virulence	*gapA*	glyceraldehyde-3-phosphate dehydrogenase GapA	57988	25
26	MGCS36044_03546			*grpE*	heat shock protein/nucleotide exchange factor GrpE	57637	26
27	MGCS36044_02048		Virulence	*irr*	TCS[6] signal transduction DNA-binding response regulator Irr	56644	27
28	MGCS36044_03544			*dnaK*	molecular chaperone DnaK	54646	28
29	MGCS36044_02340			-	DUF2273 domain-containing protein	54340	29

No.	Locus tag	SignalP6 predicted[1]	Virulence[2]	Gene	Function	RPKMs in NHP 15[3]	RANK[4]
30	MGCS36044_00770			-	putative metal homeostasis protein	52042	30
31	MGCS36044_02050		Virulence	*ihk*	TCS signal transduction histidine kinase sensor Ihk	47929	31
32	MGCS36044_02338			-	Asp23/Gls24 family envelope stress response protein	47388	32
33	MGCS36044_03548			*hrcA*	heat-inducible transcriptional repressor HrcA	46678	33
34	MGCS36044_03336			*gpsB*	cell division regulator GpsB	45768	34
35	MGCS36044_04104			*spxA_2*	transcriptional regulator SpxA	43756	35
36	MGCS36044_01516		Virulence	*sagC*	streptolysin S biosynthesis protein SagC	42497	36
37	MGCS36044_00872			*lemA*	LemA family protein	42365	37
38	MGCS36044_03146	Lipo		*tlpA*	TlpA family protein disulfide reductase lipoprotein	40700	38
39	MGCS36044_02220			-	CsbD family protein	38860	39
40	MGCS36044_02042			*acrA*	AcrA superfamily multidrug efflux pump	38736	40
41	MGCS36044_02218			-	hypothetical protein	38633	41
42	MGCS36044_02334			-	Asp23/Gls24 family envelope stress response protein	38405	42
43	MGCS36044_02044			-	LolD superfamily ABC transporter ATP-binding component	38253	43
44	MGCS36044_02668			*dltD*	D-alanyl-lipoteichoic acid biosynthesis protein DltD	38031	44
45	MGCS36044_03144			*msrA/B*	Peptide methionine sulfoxide reductase MsrA/MsrB	37614	45
46	MGCS36044_02040	Lipo		-	ABC transporter protein	35507	46
47	MGCS36044_01066	Lipo		*mtsA*	metal ABC transporter substrate-binding lipoprotein MtsA	33964	47
48	MGCS36044_02126			-	SpF66_s RNA	33685	48
49	MGCS36044_02672			*dltB*	D-alanyl-lipoteichoic acid biosynthesis protein DltB	32082	49
50	MGCS36044_03280			*raiA*	ribosome-associated translation inhibitor RaiA	31846	50
51	MGCS36044_03396	Lipo		-	PepSY domain-containing lipoprotein	31415	51
52	MGCS36044_00450			-	hypothetical protein	31133	52
53	MGCS36044_04038			*ahpF*	alkyl hydroperoxide reductase F subunit AhpF	31080	53
54	MGCS36044_02066			-	hypothetical protein	30653	54
55	MGCS36044_04020			*groES*	co-chaperone GroES	28635	55
56	MGCS36044_02994			-	DNA-binding protein HU	27696	56
57	MGCS36044_02674			*dltA*	D-alanine--poly(phosphoribitol) ligase subunit DltA	25495	57
58	MGCS36044_01710			-	TVP38 superfamily protein	24856	58
59	MGCS36044_00190			*rplX*	50S ribosomal L24 protein RplX	24852	59
60	MGCS36044_01352			-	sigma factor regulator	24686	60

No.	Locus tag	SignalP6 predicted(1)	Virulence(2)	Gene	Function	RPKMs in NHP 15(3)	RANK(4)
61	MGCS36044_03148			*ccdA*	putative cytochrome c-type biogenesis protein	24319	61
62	MGCS36044_00318			-	DUF1033 domain-containing protein	23578	62
63	MGCS36044_00516	Secreted	Virulence	*emm*	cell surface M protein Emm	22915	63
64	MGCS36044_01506			*eno*	phosphopyruvate hydratase -- enolase protein Eno	22124	64
65	MGCS36044_00186			*rpsQ*	30S ribosomal S17 protein RpsQ	22078	65
66	MGCS36044_03128			-	GNAT family N-acetyltransferase	21295	66
67	MGCS36044_04098			*ruvX*	Holliday junction resolvase RuvX	21055	67
68	MGCS36044_03126			*arcB*	ornithine carbamoyltransferase ArcB	21044	68
69	MGCS36044_01508		Virulence	*srrG*	streptolysin S small regulatory RNA SrrG	20059	69
70	MGCS36044_00874			*htpX*	zinc metalloprotease HtpX	19889	70
71	MGCS36044_01716			-	KH domain-containing protein	18873	71
72	MGCS36044_03662			*trxA_2*	thioredoxin TrxA	18406	72
73	MGCS36044_01708			-	ABC transporter permease	18379	73
74	MGCS36044_02888			*ptsH*	PTS transporter phosphocarrier protein PtsH	17442	74
75	MGCS36044_00526	Secreted	Virulence	*ska*	secreted streptokinase Ska	17400	75
76	MGCS36044_01020			*lctO*	L-lactate oxidase LctO	17283	76
77	MGCS36044_00184			*rpmC*	50S ribosomal L16 protein RpmC	17184	77
78	MGCS36044_00194			*rpsZ*	type Z 30S ribosomal S14 protein RpsZ	17154	78
79	MGCS36044_04036			*ahpC*	alkyl hydroperoxide reductase C subunit AhpC	17066	79
80	MGCS36044_01350			-	RpoE superfamily DNA-directed RNA polymerase specialized sigma subunit, sigma24-like	16870	80
81	MGCS36044_01600			*mscL*	large-conductance mechanosensitive channel protein MscL	16759	81
82	MGCS36044_04096			-	DUF1292 domain-containing protein	16690	82
83	MGCS36044_00198			*rplF*	50S ribosomal L6 protein RplF	16546	83
84	MGCS36044_00172			*rplW*	50S ribosomal L23 protein RplW	16462	84
85	MGCS36044_00910			*yccA*	YccA family protein	16238	85
86	MGCS36044_02676			*dltX*	teichoic acid D-Ala incorporation-associated protein DltX	16205	86
87	MGCS36044_02336			-	CsbD family protein	16122	87
88	MGCS36044_00192			*rplE*	50S ribosomal L5 protein RplE	16117	88
89	MGCS36044_01450	Secreted		*adcA*	zinc ABC transporter secreted substrate-binding protein AdcA	15923	89
90	MGCS36044_03122			*arcT*	M20 family metallodipeptidase ArcT	15836	90
91	MGCS36044_03344			*pepC*	aminopeptidase (A) PepC	15770	91

No.	Locus tag	SignalP6 predicted(1)	Virulence(2)	Gene	Function	RPKMs in NHP 15(3)	RANK(4)
92	MGCS36044_01490			*rplS*	50S ribosomal L19 protein RpsL	15652	92
93	MGCS36044_03130			*arcA*	arginine deiminase ArcA	15387	93
94	MGCS36044_00196			*rpsH*	30S ribosomal S8 protein RpsH	14924	94
95	MGCS36044_01704			-	RND family transporter membrane fusion protein	14661	95
96	MGCS36044_00208			*secY*	preprotein translocase subunit SecY	14521	96
97	MGCS36044_03078			*dps*	translational GTPase TypA	14214	97
98	MGCS36044_00204			*rpmD*	50S ribosomal L30 protein RpmD	14156	98
99	MGCS36044_00202			*rpsE*	30S ribosomal S5 protein RpsE	14063	99
100	MGCS36044_02840	Secreted	Virulence	*spg*	extracellular cell surface IgG-binding streptococcal protein (G) SpG	13922	100
101	MGCS36044_00200			*rplR*	50S ribosomal L18 protein RplR	13869	101
102	MGCS36044_03636			*nusB*	transcription termination protein NusB	13785	102
103	MGCS36044_01298			-	PspC domain-containing protein	13694	103
104	MGCS36044_01706			-	LolD superfamily ABC transporter ATPase	13496	104
105	MGCS36044_03944		Virulence	*perR*	peroxide-responsive transcriptional repressor PerR	13476	105
106	MGCS36044_03810			*fusA*	FusA family elongation factor EF-G	13402	106
107	MGCS36044_03616	Secreted	Virulence	*isp2*	Isp-related CHAP domain-containing immunogenic secreted protein	13368	107
108	MGCS36044_00188			*rplN*	50S ribosomal L14 protein RplN	13182	108
109	MGCS36044_04018			*groEL*	chaperonin GroEL	13028	109
110	MGCS36044_01598			*rpsU*	30S ribosomal S21 protein RpsU	12524	110
111	MGCS36044_03036			*clpA*	ATP-dependent Clp protease ATP-binding subunit ClpA	12258	111
112	MGCS36044_01306			-	YtxH domain-containing protein	12053	112
113	MGCS36044_01680			*prp*	ribosomal-processing cysteine protease Prp	11998	113
114	MGCS36044_03514			*accD*	acetyl-CoA carboxylase carboxyl transferase alpha subunit AccD	11995	114
115	MGCS36044_01032			*nrdI_2*	ribonucleotide reductase assembly protein NrdI	11915	115
116	MGCS36044_01714			*rpsP*	30S ribosomal S16 protein RpsP	11865	116
117	MGCS36044_03518			*accC*	acetyl-CoA carboxylase biotin carboxylase subunit AccC	11663	117
118	MGCS36044_04276			*parB*	chromosome partitioning protein ParB	11561	118
119	MGCS36044_03540			*dnaJ*	chaperone protein DnaJ	11480	119
120	MGCS36044_01470			*rpmE*	50S ribosomal L31 type B protein RpmE	11427	120
121	MGCS36044_00182			*rplP*	50S ribosomal L29 protein RplP	11375	121
122	MGCS36044_01682			*rpmA*	50S ribosomal L27 protein RpmA	11302	122

No.	Locus tag	SignalP6 predicted[1]	Virulence[2]	Gene	Function	RPKMs in NHP 15[3]	RANK[4]
123	MGCS36044_01272			-	SPJ_0845 family protein	10643	123
124	MGCS36044_03124			*arcD*	arginine/ornithine antiporter protein ArcD	10474	124
125	MGCS36044_02292		Virulence	*ciaR*	TCS DNA-binding response regulator protein CiaR	10459	125
126	MGCS36044_00180			*rpsC*	30S ribosomal S3 protein RpsC	10379	126
127	MGCS36044_01734			*fruA*	fructose-specific PTS transporter EIIC component FruA	10280	127
128	MGCS36044_01978			*eutD*	phosphate acetyltransferase EutD	10039	128
129	MGCS36044_02290		Virulence	*ciaH*	TCS sensor histidine kinase protein CiaH	9979	129
130	MGCS36044_04090			*nrdD_2*	anaerobic ribonucleoside-triphosphate reductase NrdD	9952	130
131	MGCS36044_04274		Virulence	*htrA*	trypsin-like serine protease HtrA	9904	131
132	MGCS36044_03300			*pknB*	Stk1 family PASTA domain-containing Ser/Thr kinase	9761	132
133	MGCS36044_01180			-	cell division protein FtsW-like	9704	133
134	MGCS36044_02546			-	hypothetical protein	9655	134
135	MGCS36044_01034			*nrdE_1*	class 1b ribonucleoside-diphosphate reductase subunit alpha NrdE	9575	135
136	MGCS36044_04100			-	IreB-related regulatory phosphoprotein	9469	136
137	MGCS36044_03958			*ulaG*	L-ascorbate utilization protein (G) UlaG	9449	137
138	MGCS36044_03812			*rpsG*	30S ribosomal S7 protein RpsG	9409	138
139	MGCS36044_01300			*hprK*	HPr(Ser) kinase/phosphatase HprK	9292	139
140	MGCS36044_02380			-	ABC transporter permease	9248	140
141	MGCS36044_00178			*rplV*	50S ribosomal L22 protein RplV	9126	141
142	MGCS36044_02550			*acoB*	pyruvate dehydrogenase E1 component beta subunit AcoB	9020	142
143	MGCS36044_01660			-	YlbF/YmcA family competence regulator	8919	143
144	MGCS36044_00764			-	Asp23/Gls24 family envelope stress response protein	8857	144
145	MGCS36044_01798			*clpL*	ATP-dependent Clp protease ATP-binding subunit ClpL	8806	145
146	MGCS36044_02480			-	AgaB family mannose/fructose/N-acetylgalactosamine-specific component IIB	8785	146
147	MGCS36044_02548			*acoC*	dihydrolipoamide acetyltransferase AcoC	8757	147
148	MGCS36044_03488			-	Cps2a family anionic cell wall polymer biosynthesis enzyme	8748	148
149	MGCS36044_01018			*lctP*	L-lactate permease LctP	8628	149
150	MGCS36044_03102			*rbsA*	D-ribose transporter ATPase RbsA	8561	150
151	MGCS36044_00750			*lrgA*	antiholin-like murein hydrolase modulator LrgA	8560	151
152	MGCS36044_01384			*mgtA*	MgtA superfamily cation-translocating P-type ATPase	8554	152
153	MGCS36044_03520			*fabZ*	3-hydroxyacyl-ACP dehydratase FabZ	8536	153

No.	Locus tag	SignalP6 predicted(1)	Virulence(2)	Gene	Function	RPKMs in NHP 15(3)	RANK(4)
154	MGCS36044_02024			*ldh*	L-lactate dehydrogenase Ldh	8496	154
155	MGCS36044_02378			-	ABC transporter ATP-binding protein	8378	155
156	MGCS36044_00768			-	SPFH domain-containing protein	8366	156
157	MGCS36044_01364			*tpiA*	triose-phosphate isomerase TpiA	8342	157
158	MGCS36044_00878		Virulence	*covR*	TCS DNA-binding response regulator CovR	8340	158
159	MGCS36044_01332			-	TPP RNA	8277	159
160	MGCS36044_00604			*rplM*	50S ribosomal L13 protein RplM	8255	160
161	MGCS36044_00170			*rplD*	50S ribosomal L4 protein RplD	8141	161
162	MGCS36044_03034			-	DUF1797 family protein	7971	162
163	MGCS36044_01030			*nrdF_1*	ribonucleotide-diphosphate reductase subunit beta NrdF	7932	163
164	MGCS36044_02376			*yhcF*	YhcF family transcriptional regulator	7912	164
165	MGCS36044_03098	Lipo		*rbsB*	D-ribose ABC transporter substrate-binding lipoprotein RbsB	7908	165
166	MGCS36044_03020			-	phospho-sugar mutase	7898	166
167	MGCS36044_03040			-	DUF1827 family protein	7870	167
168	MGCS36044_02478			*manY*	ManY family PTS mannose/fructose IIC component	7736	168
169	MGCS36044_01330			*thiT*	energy-coupled thiamine transporter ThiT	7712	169
170	MGCS36044_02070			*dppD*	dipeptide ABC transport system ATP-binding protein DppD	7707	170
171	MGCS36044_00766			-	DAK2 domain-containing protein	7688	171
172	MGCS36044_02902			-	transcriptional regulator	7643	172
173	MGCS36044_01562			*atpD*	ATP synthase beta subunit AtpD	7629	173
174	MGCS36044_01548			*glgA*	glycogen synthase GlgA	7618	174
175	MGCS36044_03536			*fabT*	transcriptional regulatory protein FabT	7592	175
176	MGCS36044_03638			-	Asp23/Gls24 family envelope stress response protein	7589	176
177	MGCS36044_03138			-	MmcQ/YjbR family DNA-binding protein	7571	177
178	MGCS36044_03378			*ccmA*	CcmA family multidrug ABC transporter ATPase component	7562	178
179	MGCS36044_01304			-	COG4768 superfamily YoxC-like protein	7558	179
180	MGCS36044_03376			-	hypothetical protein	7511	180
181	MGCS36044_01068			*mtsB*	metal ABC transporter ATP-binding protein MtsB	7500	181
182	MGCS36044_03048			-	RNA-binding protein	7438	182
183	MGCS36044_03534			*fabH*	3-oxoacyl-[acyl-carrier-protein] synthase protein FabH	7379	183
184	MGCS36044_03878			-	hypothetical protein	7363	184

No.	Locus tag	SignalP6 predicted[1]	Virulence[2]	Gene	Function	RPKMs in NHP 15[3]	RANK[4]
185	MGCS36044_01688			*rluD*	ribosomal large subunit pseudouridine synthase RluD	7353	185
186	MGCS36044_03622			*secA*	preprotein translocase subunit SecA	7350	186
187	MGCS36044_02364			*glmS*	glutamine--fructose-6-phosphate transaminase (isomerizing)	7312	187
188	MGCS36044_01170			-	DUF853 domain-containing protein	7302	188
189	MGCS36044_04060			*rpsB*	30S ribosomal S2 protein RpsB	7238	189
190	MGCS36044_00174			*rplB*	50S ribosomal L2 protein RplB	7237	190
191	MGCS36044_01554			*atpF*	ATP synthase B subunit AtpF	7181	191
192	MGCS36044_04062			*tsf*	translation elongation factor Tsf	7134	192
193	MGCS36044_03466			-	YlxQ-related RNA-binding protein	7093	193
194	MGCS36044_03956			-	diacylglycerol kinase family lipid kinase	7057	194
195	MGCS36044_01678			*rplU*	50S ribosomal L21 protein RplU	7055	195
196	MGCS36044_03814			*rpsL*	30S ribosomal S12 protein RpsL	7048	196
197	MGCS36044_03948			-	hypothetical protein	7029	197
198	MGCS36044_01546			*glgD*	glucose-1-phosphate adenylyltransferase subunit GlgC	7019	198
199	MGCS36044_02348			-	glycine RNA	6976	199
200	MGCS36044_00564			*rpsO*	30S ribosomal S15 protein RpsO	6934	200
201	MGCS36044_00176			*rpsS*	30S ribosomal S19 protein RpsS	6904	201
202	MGCS36044_02544			*acoL*	dihydrolipoyl dehydrogenase AcoL	6897	202
203	MGCS36044_01640			-	LysM peptidoglycan-binding domain-containing protein	6875	203
204	MGCS36044_03586			*uspA*	UspA family nucleotide-binding universal stress protein	6863	204
205	MGCS36044_00220			*rpoA*	DNA-directed RNA polymerase subunit alpha RpoA	6817	205
206	MGCS36044_03464			*infB*	translation initiation factor IF-2	6798	206
207	MGCS36044_00316			*rpoC*	DNA-directed RNA polymerase subunit beta' RpoC	6778	207
208	MGCS36044_03314			*rny*	ribonuclease (Y) Rny	6743	208
209	MGCS36044_00168			*rplC*	50S ribosomal L3 protein RplC	6726	209
210	MGCS36044_03542			-	PfpI family predicted protease/amidase	6725	210
211	MGCS36044_02886			*ptsI*	phosphoenolpyruvate--protein phosphotransferase PtsI	6720	211
212	MGCS36044_03364			*mraY*	phospho-N-acetylmuramoyl-pentapeptide- translocase MraY	6711	212
213	MGCS36044_00158			-	hypothetical protein	6708	213
214	MGCS36044_02020			*fadH2*	FadH2 superfamily FAD-dependent oxidoreductase	6701	214
215	MGCS36044_02898			-	CPBP family intramembrane metalloprotease	6675	215

No.	Locus tag	SignalP6 predicted[(1)]	Virulence[(2)]	Gene	Function	RPKMs in NHP 15[(3)]	RANK[(4)]
216	MGCS36044_02458			-	NAD-dependent succinate-semialdehyde dehydrogenase	6659	216
217	MGCS36044_01662			*gorA*	glutathione reductase GorA	6633	217
218	MGCS36044_02370			*pyk*	pyruvate kinase Pyk	6619	218
219	MGCS36044_02270			-	putative nucleoside ABC transporter permease	6603	219
220	MGCS36044_04004			*pbp2A*	multimodular transpeptidase-transglycosylase penicillin-binding protein Pbp2A	6597	220
221	MGCS36044_01564			*atpC*	ATP synthase epsilon subunit AtpC	6576	221
222	MGCS36044_02476			*manZ*	ManZ family PTS mannose/fructose IID component	6561	222
223	MGCS36044_00030			*ftsH*	ATP-dependent zinc metalloprotease FtsH	6559	223
224	MGCS36044_02010			*glyA*	serine hydroxymethyl transferase GlyA	6557	224
225	MGCS36044_00494			*rsmE*	16S rRNA (uracil(1498)-N(3))-methyltransferase RsmE	6547	225
226	MGCS36044_02980			*gpmA*	phosphoglycerate mutase GpmA	6497	226
227	MGCS36044_00908			*rnaY*	RnaY family HD domain-containing protein	6483	227
228	MGCS36044_02140			-	FAD-binding oxidoreductase	6483	227
229	MGCS36044_01080			*rplA*	50S ribosomal L1 protein RplA	6429	229
230	MGCS36044_03656			*rpsF*	30S ribosomal S6 protein RpsF	6413	230
231	MGCS36044_02008			-	GNAT family N-acetyltransferase	6387	231
232	MGCS36044_03946			*yccU*	YccU family CoA-binding protein	6364	232
233	MGCS36044_04082			*nrdG*	anaerobic ribonucleoside-triphosphate reductase activating protein NrdG	6358	233
234	MGCS36044_03382			*tkt*	transketolase Tkt	6344	234
235	MGCS36044_00206			*rplO*	50S ribosomal L15 protein RplO	6282	235
236	MGCS36044_03054			*yggS*	YggS family pyridoxal phosphate-dependent enzyme	6182	236
237	MGCS36044_03858			*rpmH*	50S ribosomal L34 protein RpmH	6177	237
238	MGCS36044_00606			*rpsI*	30S ribosomal S9 protein RpsI	6088	238
239	MGCS36044_02598			*rfbC*	dTDP-4-dehydrorhamnose 3,5-epimerase RfbC	6085	239
240	MGCS36044_02272			-	putative nucleoside ABC transporter permease	6065	240
241	MGCS36044_01302			*lgt*	prolipoprotein diacylglyceryl transferase Lgt	6029	241
242	MGCS36044_02266			*panT*	pantothenic acid transporter PanT	6024	242
243	MGCS36044_04022			*clpC*	ATP-dependent Clp protease ATP-binding subunit ClpC	6023	243
244	MGCS36044_00166			*rpsJ*	30S ribosomal S10 protein RpsJ	6011	244
245	MGCS36044_01560			*atpG*	ATP synthase gamma subunit AtpG	5997	245
246	MGCS36044_01732			*fruK*	1-phosphofructokinase FruK	5990	246

No.	Locus tag	SignalP6 predicted[(1)]	Virulence[(2)]	Gene	Function	RPKMs in NHP 15[(3)]	RANK[(4)]
247	MGCS36044_01802			*rplJ*	50S ribosomal L10 protein RplJ	5984	247
248	MGCS36044_03302			*pppL*	Stp1/IreP family PP2C-type Ser/Thr phosphatase	5953	248
249	MGCS36044_03724	Secreted		-	putative secreted protein	5952	249
250	MGCS36044_03860			*jag*	RNA-binding protein Jag	5931	250
251	MGCS36044_02006			-	Sua5/YciO/YrdC/YwlC family protein ribosome maturation factor	5927	251
252	MGCS36044_03640			*efp*	translation elongation factor (P) Efp	5881	252
253	MGCS36044_01556			*atpH*	ATP synthase delta subunit AtpH	5860	253
254	MGCS36044_02064			*htpA*	streptococcal histidine triad (HIT)	5769	254
255	MGCS36044_03104			*rbsD*	D-ribose pyranase RbsD	5764	255
256	MGCS36044_03516			*accA*	acetyl-CoA carboxylase, carboxyltransferase beta subunit AccA	5761	256
257	MGCS36044_01078			*rplK*	50S ribosomal L11P protein RplK	5732	257
258	MGCS36044_01712			-	glycerophosphodiester phosphodiesterase	5702	258
259	MGCS36044_02392			*rpsA*	30S ribosomal S1 protein RpsA	5665	259
260	MGCS36044_03654			*ssb_2*	single-stranded DNA-binding protein	5609	260
261	MGCS36044_01428			*ftsE*	cell division ATP-binding protein FtsE	5607	261
262	MGCS36044_00966			*upp*	uracil phosphoribosyltransferase Upp	5589	262
263	MGCS36044_02076	Lipo		*dppA*	dipeptide-binding ABC transport system substrate-binding lipoprotein DppA	5587	263
264	MGCS36044_01094	Secreted		*lysM*	LysM peptidoglycan-binding domain-containing secreted protein	5576	264
265	MGCS36044_00740			*tig*	trigger factor molecular chaperone Tig	5554	265
266	MGCS36044_01220			*gla*	glycerol uptake facilitator protein Gla	5551	266
267	MGCS36044_00968			*clpP*	ATP-dependent Clp protease proteolytic subunit ClpP	5537	267
268	MGCS36044_03786			*rnjA_2*	mRNA degradation ribonuclease RnjA	5533	268
269	MGCS36044_00214			*rpmJ*	50S ribosomal L36 protein RpmJ	5516	269
270	MGCS36044_02908	Lipo		*prsA*	peptidylprolyl isomerase lipoprotein PrsA	5508	270
271	MGCS36044_00336			*ackA*	acetate kinase AckA	5487	271
272	MGCS36044_03136			-	B3/4 domain-containing protein	5464	272
273	MGCS36044_02436			*deoD*	DeoD-type purine-nucleoside phosphorylase	5455	273
274	MGCS36044_02430			-	hypothetical protein	5444	274
275	MGCS36044_01216			*dhaL*	dihydroxyacetone kinase subunit DhaL	5430	275
276	MGCS36044_03106			*rbsK*	ribokinase RbsK	5416	276
277	MGCS36044_03816	Secreted		*prgA*	surface exclusion domain-containing secreted protein PrgA	5414	277

No.	Locus tag	SignalP6 predicted[1]	Virulence[2]	Gene	Function	RPKMs in NHP 15[3]	RANK[4]
278	MGCS36044_03142		Virulence	*yesN*	TCS DNA-binding response regulator YesN	5405	278
279	MGCS36044_02072			*dppC*	dipeptide ABC transport system permease protein DppC	5375	279
280	MGCS36044_03468			-	YlxR family putative RNA-binding protein	5371	280
281	MGCS36044_01062		Virulence	*mtsR*	metal-dependent transcriptional regulator MtsR	5370	281
282	MGCS36044_04254			*guaB*	IMP dehydrogenase GuaB	5353	282
283	MGCS36044_03490			-	GNAT family N-acetyltransferase	5338	283
284	MGCS36044_02214			-	LCB5 family diacylglycerol lipid kinase	5334	284
285	MGCS36044_01558			*atpA*	ATP synthase alpha chain, AtpA	5330	285
286	MGCS36044_03118			*arcC*	carbamate kinase ArcC	5319	286
287	MGCS36044_01256			*ftsY*	signal recognition particle-docking protein FtsY	5317	287
288	MGCS36044_03462			*rbfA*	30S ribosome-binding factor RbfA	5289	288
289	MGCS36044_03056			*ftsZ*	cell division protein FtsZ	5278	289
290	MGCS36044_04102			-	SSRC41 RNA	5245	290
291	MGCS36044_02068			*dppF*	dipeptide ABC transport system ATP-binding protein DppF	5233	291
292	MGCS36044_00414			*pgpA*	phosphatidylglycerophosphatase protein PgpA	5211	292
293	MGCS36044_02372			*pfkA*	6-phosphofructokinase PfkA	5210	293
294	MGCS36044_04002			*secE*	preprotein translocase subunit protein SecE	5184	294
295	MGCS36044_03140		Virulence	*yesM*	TCS sensor kinase YesM	5181	295
296	MGCS36044_03804			*pgk*	phosphoglycerate kinase Pgk	5167	296
297	MGCS36044_02552			*acoA*	Pyruvate dehydrogenase E1 component alpha subunit AcoA	5132	297
298	MGCS36044_03100			*rbsC*	D-ribose ABC transporter permease RbsC	5100	298
299	MGCS36044_01430			*ftsX*	cell division permease-like protein FtsX	5097	299
300	MGCS36044_00302			*adcR*	zinc-dependent MarR family transcriptional regulator/adhesin competence repressor AdcR	5091	300
301	MGCS36044_03522			*accB*	acetyl-CoA carboxylase biotin carboxyl carrier protein AccB	5082	301
302	MGCS36044_03828			*rsgA*	ribosome small subunit-dependent GTPase (A) RgsA	5074	302
303	MGCS36044_02834			*map*	methionyl aminopeptidase Map	5041	303
304	MGCS36044_00912			*yneF*	YneF family protein	5040	304
305	MGCS36044_03900			*galU*	UTP--glucose-1-phosphate uridylyltransferase GalU	5037	305
306	MGCS36044_03528			*fabD*	malonyl CoA-acyl carrier protein transacylase protein FabD	5035	306
307	MGCS36044_00502			-	Udk family kinase	5030	307
308	MGCS36044_03330			*mapZ*	MapZ family cell division site-positioning protein	5016	308

No.	Locus tag	SignalP6 predicted(1)	Virulence(2)	Gene	Function	RPKMs in NHP 15(3)	RANK(4)
309	MGCS36044_03682			*pflB*	formate C-acetyltransferase	5006	309
310	MGCS36044_01686			*lspA*	lipoprotein signal peptidase II LspA	4997	310
311	MGCS36044_03212	Lipo		*ugpB_1*	glycerol-3-phosphate ABC transporter substrate-binding lipoprotein UgpB	4994	311
312	MGCS36044_03806	Lipo		*lppC*	e(P4) family 5'-nucleotidase lipoprotein	4963	312
313	MGCS36044_02900			-	DUF3169 family protein	4907	313
314	MGCS36044_02808			-	TetR/AcrR family transcriptional regulator	4893	314
315	MGCS36044_03596			*cbiO*	cobalt ECF transporter (A) ATPase component CbiO	4891	315
316	MGCS36044_00104	Secreted		*sibA*	CHAP domain-containing protein/secreted immunoglobulin-binding protein SibA	4887	316
317	MGCS36044_03252			-	DUF402 domain-containing protein	4879	317
318	MGCS36044_02014			-	lysozyme family protein	4867	318
319	MGCS36044_00148			-	low molecular weight phosphotyrosine protein phosphatase	4847	319
320	MGCS36044_00866			*ktrA*	potassium uptake transporter gating subunit KtrA	4842	320
321	MGCS36044_00218			*rpsK*	30S ribosomal S11 protein RpsK	4838	321
322	MGCS36044_01690			-	membrane-associated alkaline phosphatase	4821	322
323	MGCS36044_03356			-	GlnQ family polar amino acid ABC transporter ATPase	4785	323
324	MGCS36044_00578			*cysE*	serine O-acetyltransferase CysE	4779	324
325	MGCS36044_02882			-	PgdA-like putative PG GlcNAc deacetylase	4777	325
326	MGCS36044_01164			*coaE*	dephospho-CoA kinase CoaE	4767	326
327	MGCS36044_00518	Secreted		-	cell surface UshA family bifunctional 2',3'-cyclic-nucleotide 2'-phosphodiesterase/3'-nucleotidase	4737	327
328	MGCS36044_03524			*fabF*	3-oxoacyl-[acyl-carrier-protein] synthase protein FabF	4727	328
329	MGCS36044_03506			*manN*	PTS transporter mannose-specific IID component protein ManN	4726	329
330	MGCS36044_03550			-	FlgJ-related putative peptidoglycan hydrolase	4706	330
331	MGCS36044_02264			*coaC*	phosphopantothenoylcysteine decarboxylase CoaC	4704	331
332	MGCS36044_01008			*arsC_1*	arsenate reductase ArsC	4692	332
333	MGCS36044_02440			*arsC_2*	arsenate reductase ArsC	4655	333
334	MGCS36044_03384			*fsa*	FSA family fructose-6-phosphate aldolase	4638	334
335	MGCS36044_00392	Lipo		-	BMP family ABC transporter substrate-binding lipoprotein	4635	335
336	MGCS36044_00314			*rpoB*	DNA-directed RNA polymerase subunit beta RpoB	4632	336
337	MGCS36044_00122			*purN*	phosphoribosylglycinamide formyltransferase PurN	4599	337
338	MGCS36044_02276	Lipo		-	putative nucleoside ABC transporter substrate-binding lipoprotein	4561	338

No.	Locus tag	SignalP6 predicted(1)	Virulence(2)	Gene	Function	RPKMs in NHP 15(3)	RANK(4)
339	MGCS36044_03310			*rpoZ*	DNA-directed RNA polymerase omega subunit RpoZ	4559	339
340	MGCS36044_03046			*divIVA*	cell division protein DivIVA	4549	340
341	MGCS36044_02274			-	putative nucleoside ABC transporter ATP-binding protein	4544	341
342	MGCS36044_00222			*rplQ*	50S ribosomal L17 protein RplQ	4530	342
343	MGCS36044_03708			-	pyridoxamine 5'-phosphate oxidase family protein	4480	343
344	MGCS36044_00520			*relA*	bifunctional (p)ppGpp synthase/hydrolase RelA	4461	344
345	MGCS36044_02280			*deoC*	deoxyribose-phosphate aldolase DeoC	4446	345
346	MGCS36044_00150			-	hypothetical protein	4445	346
347	MGCS36044_01218			*dhaM*	PTS-dependent dihydroxyacetone kinase phosphotransferase subunit DhaM	4425	347
348	MGCS36044_02586			-	tetra tricopeptide repeat family protein	4385	348
349	MGCS36044_03826			*rpe*	ribulose-phosphate 3-epimerase Rpe	4353	349
350	MGCS36044_03592			-	aldo/keto reductase	4343	350
351	MGCS36044_00848			-	YebC/PmpR family DNA-binding transcriptional regulator	4317	351
352	MGCS36044_03818			*purR*	pur operon repressor PurR	4315	352
353	MGCS36044_02162	Lipo		-	FMN-binding protein, major membrane immunogen, membrane-anchored lipoprotein	4306	353
354	MGCS36044_03678			-	hypothetical protein	4306	353
355	MGCS36044_00120			*purM*	phosphoribosylformylglycinamidine cyclo-ligase PurM	4267	355
356	MGCS36044_02632			-	glutathione S-transferase N-terminal domain-containing protein	4261	356
357	MGCS36044_02232			*nrdD_1*	ATP cone domain-containing protein, ribonucleoside-triphosphate reductase	4258	357
358	MGCS36044_01544			*glgC*	glucose-1-phosphate adenylyltransferase subunit GlgD	4247	358
359	MGCS36044_03342			*pbp1A*	bifunctional PG transglycosylase-transpeptidase class A penicillin-binding protein PBP1A	4232	359
360	MGCS36044_03974			*leuS*	leucine--tRNA synthase LeuS	4230	360
361	MGCS36044_01086			*frr*	ribosome recycling factor Frr	4223	361
362	MGCS36044_00902	Lipo		*yidC_1*	membrane protein insertase lipoprotein YidC	4220	362
363	MGCS36044_00758			*fba_2*	fructose-bisphosphate aldolase	4216	363
364	MGCS36044_00510			-	PreQ1 RNA	4204	364
365	MGCS36044_00836			*rsfS*	ribosome silencing factor RsfS	4191	365
366	MGCS36044_02294			*pepN*	lysyl aminopeptidase/alanine aminopeptidase PepN	4156	366
367	MGCS36044_03820			*cbf*	YhaM family 3'-5' exoribonuclease	4139	367
368	MGCS36044_03016			-	PfkB superfamily kinase	4131	368

No.	Locus tag	SignalP6 predicted[1]	Virulence[2]	Gene	Function	RPKMs in NHP 15[3]	RANK[4]
369	MGCS36044_04252			*glcU*	glucose uptake permease GlcU	4105	369
370	MGCS36044_01024			-	hypothetical protein	4083	370
371	MGCS36044_04084			-	putative acetyltransferase	4080	371
372	MGCS36044_03594			*recG*	ATP-dependent DNA helicase RecG	4068	372
373	MGCS36044_02012			-	nucleoid-associated bacterial family protein	4019	373
374	MGCS36044_02798			-	MdlB family multidrug ABC transporter ATPase and permease	4019	373
375	MGCS36044_01316			-	NAD(P)-binding domain-containing protein	4017	375
376	MGCS36044_00496			-	LacI family DNA-binding transcriptional regulator	4001	376
377	MGCS36044_02704			*obgE*	GTPase ObgE	4000	377
378	MGCS36044_02150			-	cupin domain-containing protein	3997	378
379	MGCS36044_01426			*prfB*	peptide chain release factor 2 PrfB	3982	379
380	MGCS36044_00584			*mrnC*	mini-ribonuclease 3 MrnC	3967	380
381	MGCS36044_00300			*ipk*	4-diphosphocytidyl-2-C-methyl-D-erythritol kinase Ipk	3953	381
382	MGCS36044_03366			*pbp2X*	PG transpeptidase class B penicillin-binding protein PBP2X/FtsI	3952	382
383	MGCS36044_00028			-	hypoxanthine-guanine phosphoribosyltransferase	3941	383
384	MGCS36044_00904			*acyP*	acylphosphatase AcyP	3938	384
385	MGCS36044_01046			-	DUF3977 family protein	3925	385
386	MGCS36044_03418			-	Cof-type HAD-IIB family phosphohydrolase	3923	386
387	MGCS36044_02538			*lplA_2*	lipoate--protein ligase LplA	3915	387
388	MGCS36044_03584			*alaT*	AlaT family aminotransferase	3905	388
389	MGCS36044_02036	Secreted		-	DUF1002 domain-containing putative secreted protein	3900	389
390	MGCS36044_02596			*rfbB*	dTDP-glucose 4,6-dehydratase RfbB	3899	390
391	MGCS36044_03882			-	AIM24 family protein	3829	391
392	MGCS36044_04260			*uup*	Uup family ATPase components of ABC transporters with duplicated ATPase domains	3827	392
393	MGCS36044_00950			*ppaC*	manganese-dependent inorganic pyrophosphatase PpaC	3824	393
394	MGCS36044_01240		Virulence	*vicK*	TCS signal transduction sensor kinase VicK	3823	394
395	MGCS36044_00540			*yajC*	preprotein translocase subunit YajC	3816	395
396	MGCS36044_04086			*mviM*	MviM family predicted dehydrogenase	3811	396
397	MGCS36044_03292			-	putative bifunctional phosphatase/peptidyl-prolyl cis-trans isomerase	3798	397
398	MGCS36044_02438			*xapA*	XapA family purine-nucleoside phosphorylase	3776	398
399	MGCS36044_01684			*lysR*	LysR family transcriptional regulator	3770	399

No.	Locus tag	SignalP6 predicted[(1)]	Virulence[(2)]	Gene	Function	RPKMs in NHP 15[(3)]	RANK[(4)]
400	MGCS36044_02074			*dppB*	dipeptide ABC transport system permease protein DppB	3769	400
401	MGCS36044_01192			*pepQ*	Xaa-Pro dipeptidase protein PepQ	3758	401
402	MGCS36044_03552			-	LD/DD carboxypeptidase family protein	3756	402
403	MGCS36044_00798	Lipo		*oppA_1*	oligopeptide ABC transporter substrate-binding lipoprotein OppA	3753	403
404	MGCS36044_03532			*acpP_2*	acyl carrier protein AcpP	3753	403
405	MGCS36044_03470			*nusA*	transcription termination factor NusA	3718	405
406	MGCS36044_02528			*glmM*	phosphoglucosamine mutase GlmM	3697	406
407	MGCS36044_02442			*deoB*	phosphopentomutase DeoB	3684	407
408	MGCS36044_02988			*zntA*	ZntA family P-type heavy metal transporter ATPase	3675	408
409	MGCS36044_02866	Lipo	Virulence	*slr*	InlA-like streptococcal leucine rich lipoprotein Slr	3653	409
410	MGCS36044_04180			-	hypothetical protein	3650	410
411	MGCS36044_00996			*rsmI*	16S rRNA (cytidine(1402)-2'-O)-methyltransferase RsmL	3636	411
412	MGCS36044_00888			*der*	ribosome biogenesis GTPase Der	3628	412
413	MGCS36044_01806			-	rli38 RNA	3626	413
414	MGCS36044_00306			*adcB*	metal ABC transporter permease AdcB	3613	414
415	MGCS36044_00804			*oppD_1*	oligopeptide ABC transporter permease protein OppD	3607	415
416	MGCS36044_03440			*copA_2*	copper-exporting ATPase CopA	3607	415
417	MGCS36044_02636			-	rhodanese-related sulfurtransferase	3595	417
418	MGCS36044_00390			*purA*	adenylosuccinate synthase PurA	3578	418
419	MGCS36044_00780			*mecA*	negative regulator of genetic competence, adaptor protein MecA	3565	419
420	MGCS36044_01228			-	DUF3114 domain-containing protein	3556	420
421	MGCS36044_02650	Lipo		*malE*	maltose/maltodextrin ABC transport system substrate-binding lipoprotein MalE	3552	421
422	MGCS36044_00312			-	Lacto-rpoB RNA	3532	422
423	MGCS36044_03664			-	FAD-containing oxidoreductase	3528	423
424	MGCS36044_03526			*fabG_2*	3-ketoacyl-(acyl-carrier-protein) reductase protein FabG	3521	424
425	MGCS36044_02936			*sodA*	superoxide dismutase SodA	3520	425
426	MGCS36044_03530			*fabK*	Enoyl-[acyl-carrier-protein] reductase protein FabK	3519	426
427	MGCS36044_00304			*adcC*	metal ABC transporter ATP-binding protein AdcC	3518	427
428	MGCS36044_01060			*mtnN*	5'-methylthioadenosine/adenosylhomocysteine nucleosidase MtnN	3518	427
429	MGCS36044_01804			*rplL*	50S ribosomal L7/L12 protein RplL	3514	429
430	MGCS36044_00588	Secreted		-	putative secreted protein	3513	430

No.	Locus tag	SignalP6 predicted[1]	Virulence[2]	Gene	Function	RPKMs in NHP 15[3]	RANK[4]
431	MGCS36044_04202			*rpsD*	30S ribosomal S4 protein RpsD	3507	431
432	MGCS36044_03362			*cshB*	DEAD/DEAH box helicase	3498	432
433	MGCS36044_03058			*ftsA*	cell division protein FtsA	3486	433
434	MGCS36044_02806			-	ECF transporter S component	3474	434
435	MGCS36044_01424			*queG*	epoxyqueuosine reductase QueG	3467	435
436	MGCS36044_02278			*cdd*	cytidine deaminase Cdd	3466	436
437	MGCS36044_01552			*atpB*	ATP synthase A subunit AtpB	3465	437
438	MGCS36044_00152			*oatA*	acetyltransferase OatA	3454	438
439	MGCS36044_04128			*argS*	arginine--tRNA synthase ArgS	3446	439
440	MGCS36044_03372			*proA*	glutamate-5-semialdehyde dehydrogenase ProA	3434	440
441	MGCS36044_00972			-	YlbF family regulatory protein	3429	441
442	MGCS36044_02762			-	helix-turn-helix domain-containing putative transcriptional regulator	3428	442
443	MGCS36044_03928			-	Bacteria_small_SRP RNA	3424	443
444	MGCS36044_03920			*pgi*	Pgi family glucose-6-phosphate isomerase	3402	444
445	MGCS36044_04108			*recA*	recombinase RecA	3398	445
446	MGCS36044_01606			-	metal-sulfur cluster assembly factor	3390	446
447	MGCS36044_02318			*truB*	tRNA pseudouridine(55) synthase TruB	3365	447
448	MGCS36044_02062	Lipo	Virulence	*lmb*	bifunctional metal ABC transporter substrate-binding lipoprotein/laminin-binding lipoprotein Lmb	3341	448
449	MGCS36044_01162			*mutM*	DNA-formamidopyrimidine glycosylase MutM	3340	449
450	MGCS36044_02864			*metK*	methionine adenosyltransferase MetK	3330	450
451	MGCS36044_02530	Lipo		*ybbR*	YbbR family lipoprotein	3319	451
452	MGCS36044_02708			*pepS*	aminopeptidase PepS	3314	452
453	MGCS36044_00126			*purH*	bifunctional phosphoribosylaminoimidazolecarboxamide formyltransferase/IMP cyclohydrolase PurH	3306	453
454	MGCS36044_03824			*thiN*	thiamine diphosphokinase ThiN	3304	454
455	MGCS36044_03492			*tsaE*	tRNA (adenosine(37)-N6)-threonylcarbamoyltransferase TsaE	3297	455
456	MGCS36044_03050			-	YggT family protein	3286	456
457	MGCS36044_02128			*guaA*	glutamine-hydrolyzing GMP synthase	3271	457
458	MGCS36044_03224			*bglB_2*	6-phospho-beta-glucosidase BglB	3256	458
459	MGCS36044_03504			*manM*	PTS transporter mannose-specific IIC component protein ManM	3247	459
460	MGCS36044_01438			*asnC*	asparaginyl-tRNA synthetase protein AsnC	3244	460

No.	Locus tag	SignalP6 predicted[1]	Virulence[2]	Gene	Function	RPKMs in NHP 15[3]	RANK[4]
461	MGCS36044_03112			*coaD*	pantetheine-phosphate adenylyltransferase CoaD	3244	460
462	MGCS36044_01070			*mtsC*	metal ABC transporter permease MtsC	3241	462
463	MGCS36044_01194		Virulence	*ccpA*	catabolite control protein CcpA	3238	463
464	MGCS36044_00530			*msmK*	sn-glycerol-3-phosphate ABC transporter ATP-binding protein MsmK	3233	464
465	MGCS36044_00212			*infA*	translation initiation factor IF-1 protein InfA	3230	465
466	MGCS36044_03420			-	YadS family trimeric intracellular cation channel protein	3211	466
467	MGCS36044_00594			-	DegV family protein	3194	467
468	MGCS36044_02296			*phoU_2*	phosphate signaling complex protein PhoU	3193	468
469	MGCS36044_03374			*proB*	glutamate 5-kinase ProB	3192	469
470	MGCS36044_03618			*alr*	alanine racemase Alr	3192	469
471	MGCS36044_01200			-	Glycosyltransferase	3177	471
472	MGCS36044_02482			*manX*	ManX family PTS mannose/fructose IIA component	3146	472
473	MGCS36044_02872			-	GAF domain containing protein	3136	473
474	MGCS36044_00490			-	DUF3013 family protein	3130	474
475	MGCS36044_01542			*glgB*	1,4-alpha-glucan branching protein GlgB	3120	475
476	MGCS36044_02848			*uxuA*	mannonate dehydratase	3120	475
477	MGCS36044_02410			*pyrC*	dihydroorotase PyrC	3118	477
478	MGCS36044_00864			*sstT*	serine/threonine transporter SstT	3117	478
479	MGCS36044_04064			*pepO*	endopeptidase PepO	3117	478
480	MGCS36044_03390			*glpF_1*	GlpF family glycerol uptake facilitator and related aquaporins	3110	480
481	MGCS36044_01048			-	ASCH domain-containing RNA-binding protein	3106	481
482	MGCS36044_00658			-	type II toxin-antitoxin system Phd/YefM family antitoxin	3097	482
483	MGCS36044_02180			-	PASTA domain-containing protein	3094	483
484	MGCS36044_03442			*copY_2*	DNA-binding copper transport transcriptional repressor CopY	3092	484
485	MGCS36044_03052			*sepF*	cell division protein SepF	3080	485
486	MGCS36044_02594			-	Abi family CAAX protease self-immunity protein	3078	486
487	MGCS36044_00216			*rpsM*	30S ribosomal S13 protein RpsM	3049	487
488	MGCS36044_04144			*rpmGA*	50S ribosomal L33 protein RpmGA	3049	487
489	MGCS36044_02448			*pepV*	dipeptidase PepV	3045	489
490	MGCS36044_02536			-	CobQ-like type 1 glutamine amidotransferase	3043	490
491	MGCS36044_02016			-	MdlB superfamily multidrug ABC transporter permease	3037	491

No.	Locus tag	SignalP6 predicted[1]	Virulence[2]	Gene	Function	RPKMs in NHP 15[3]	RANK[4]
492	MGCS36044_03666			*mutS2*	DNA mismatch repair endonuclease MutS2	3033	492
493	MGCS36044_02212			*yqfA*	membrane channel forming/hemolysin III protein YqfA	3022	493
494	MGCS36044_02600			*rfbA*	glucose-1-phosphate thymidylyltransferase RfbA	3011	494
495	MGCS36044_01756			*fms*	peptide deformylase	3007	495
496	MGCS36044_02492			-	RnaY family phosphodiesterase	2991	496
497	MGCS36044_02998			-	SGNH-hydrolase superfamily of lipases and esterases protein	2987	497
498	MGCS36044_02588			*perM*	PerM family predicted purR regulated permease	2985	498
499	MGCS36044_01004			-	GNAT family N-acetyltransferase	2976	499
500	MGCS36044_00670			-	hypothetical protein	2972	500
501	MGCS36044_01102			*ybeY*	rRNA maturation RNase YbeY	2949	501
502	MGCS36044_02718			-	DUF1846 domain-containing protein	2948	502
503	MGCS36044_00224			-	L17DE RNA	2943	503
504	MGCS36044_04238			*cbiO1*	cobalt ABC transporter ATPase CbiO2	2941	504
505	MGCS36044_01586			-	neutral zinc metallopeptidase	2932	505
506	MGCS36044_00572			*talA*	transaldolase TalA	2926	506
507	MGCS36044_03304			*rsmB*	16S rRNA (cytosine(967)-C(5))-methyltransferase RsmB	2924	507
508	MGCS36044_02928			*nagB*	glucosamine-6-phosphate deaminase NagB	2919	508
509	MGCS36044_01386			-	hypothetical protein	2914	509
510	MGCS36044_02880			*srmB*	SrmB superfamily II DNA and RNA helicase	2910	510
511	MGCS36044_01790			-	hypothetical protein	2903	511
512	MGCS36044_02920			*pepF_2*	oligoendopeptidase (F) PepF	2890	512
513	MGCS36044_02560			*rnjA_1*	mRNA degradation ribonuclease RnjA	2885	513
514	MGCS36044_02960			-	Pleckstrin homology-like domain-containing protein	2885	513
515	MGCS36044_01198			-	Glycosyltransferase	2876	515
516	MGCS36044_02894			*nrdF_2*	class 1b ribonucleoside-diphosphate reductase beta subunit NrdF	2871	516
517	MGCS36044_02132			*mngR*	MngR family DNA-binding transcriptional regulator	2842	517
518	MGCS36044_01188			*gloA*	lactoylglutathione lyase protein GloA	2824	518
519	MGCS36044_03150			-	CorA family divalent cation transport protein	2801	519
520	MGCS36044_01636			*pepT*	peptidase (T) PepT	2800	520
521	MGCS36044_02574	Secreted		*aphA*	secreted acid phosphatase AphA	2798	521
522	MGCS36044_01638			*ebsA*	EbsA family pore-forming protein	2793	522

No.	Locus tag	SignalP6 predicted(1)	Virulence(2)	Gene	Function	RPKMs in NHP 15(3)	RANK(4)
523	MGCS36044_03560			*gatB_2*	aspartyl-tRNA(Asn) or glutamyl-tRNA(Gln) amidotransferase B subunit GatB	2790	523
524	MGCS36044_01320			*lysS*	lysyl-tRNA synthetase LysS	2787	524
525	MGCS36044_03290			-	S1 RNA-binding domain-containing protein	2786	525
526	MGCS36044_02852			*eda*	Eda family bifunctional keto-hydroxyglutarate-aldolase/keto-deoxy-phosphogluconate aldolase	2770	526
527	MGCS36044_00032			*plaP*	amino acid permease PlaP	2769	527
528	MGCS36044_02434			-	LytR family transcriptional regulator	2753	528
529	MGCS36044_01604			*rpoD*	RNA polymerase sigma factor RpoD	2749	529
530	MGCS36044_00492			*prmA*	50S ribosomal protein L11 methyltransferase PrmA	2745	530
531	MGCS36044_03296		Virulence	*liaS*	three component system signal transduction sensor histidine kinase protein LiaS	2733	531
532	MGCS36044_00742			*rpoE*	DNA-directed RNA polymerase subunit delta RpoE	2724	532
533	MGCS36044_02884			*gapN*	NADP-dependent glyceraldehyde-3-phosphate dehydrogenase GapN	2723	533
534	MGCS36044_02188			-	DUF3307 domain-containing protein	2716	534
535	MGCS36044_01568			*murA_1*	UDP-N-acetylglucosamine 1-carboxyvinyltransferase protein MurA	2714	535
536	MGCS36044_03486			-	chemotaxis protein	2712	536
537	MGCS36044_00880		Virulence	*covS*	TCS sensor kinase CovS	2710	537
538	MGCS36044_03950			*polA*	DNA polymerase I PolA	2689	538
539	MGCS36044_00876			*yceD*	large ribosomal RNA subunit accumulation protein YceD	2687	539
540	MGCS36044_04088			-	hypothetical protein	2687	539
541	MGCS36044_03278			-	IS30 family transposase	2683	541
542	MGCS36044_03352			*trxB_2*	thioredoxin-disulfide reductase TrxB	2679	542
543	MGCS36044_03554			-	PhoE family broad specificity phosphatase	2679	542
544	MGCS36044_01474			*nrnA*	bifunctional oligoribonuclease/PAP phosphatase NrnA	2675	544
545	MGCS36044_01176			*rnr*	exoribonuclease (R) Rnr	2673	545
546	MGCS36044_03406			-	ARA1 family aldo/keto reductase	2673	545
547	MGCS36044_01238		Virulence	*vicR*	TCS DNA-binding response regulator VicR	2660	547
548	MGCS36044_03822			*rmuC*	DNA recombination protein RmuC	2652	548
549	MGCS36044_04242			*rodZ*	cytoskeletal protein RodZ	2593	549
550	MGCS36044_01006			*ogt*	O6-methylguanine-DNA--protein-cysteine methyltransferase Ogt	2585	550
551	MGCS36044_01280			*pnpS*	phosphate TCS signal transduction histidine kinase PnpS	2581	551
552	MGCS36044_01674			-	IS30 family transposase	2572	552

No.	Locus tag	SignalP6 predicted(1)	Virulence(2)	Gene	Function	RPKMs in NHP 15(3)	RANK(4)
553	MGCS36044_03004			*recN*	DNA repair protein RecN	2566	553
554	MGCS36044_02236			-	asd RNA	2562	554
555	MGCS36044_01934			*potB*	spermidine putrescine ABC transport system permease protein PotB	2560	555
556	MGCS36044_01468			-	PTS sugar transport IIA subunit	2554	556
557	MGCS36044_01336			-	transposase IS116/IS110/IS902 family protein	2550	557
558	MGCS36044_01296			-	SprT family protein	2547	558
559	MGCS36044_03612	Secreted		*shp*	heme-binding secreted protein Shp	2544	559
560	MGCS36044_03704			*glpF_2*	glycerol uptake facilitator GlpF	2540	560
561	MGCS36044_00754			-	alpha/beta hydrolase	2532	561
562	MGCS36044_01650			*rplT*	50S ribosomal L20 protein RplT	2521	562
563	MGCS36044_00486			*pabA*	aminodeoxychorismate/anthranilate synthase component II PabA	2508	563
564	MGCS36044_01096			*mycA*	oleate hydratase/myosin-crossreactive antigen MycA	2495	564
565	MGCS36044_03994	Secreted	Virulence	*slo*	secreted cholesterol-dependent cytolysin streptolysin O Slo	2484	565
566	MGCS36044_01644			-	L20_leader RNA	2480	566
567	MGCS36044_00830			*nadD*	nicotinate-nucleotide adenylyltransferase NadD	2472	567
568	MGCS36044_00522			*dtd*	D-tyrosyl-tRNA deacylase Dtd	2466	568
569	MGCS36044_03018			*folD*	bifunctional methylenetetrahydrofolate dehydrogenase/methenyltetrahydrofolate cyclohydrolase FolD	2456	569
570	MGCS36044_03312			*gmk*	guanylate kinase Gmk	2449	570
571	MGCS36044_03120			-	IS110 family transposase	2443	571
572	MGCS36044_00498			*ptsG*	PTS glucose-specific II ABC component	2440	572
573	MGCS36044_03306			*fmt*	methionyl-tRNA formyl transferase Fmt	2435	573
574	MGCS36044_02472			-	YesN family TCS DNA-binding response regulator	2434	574
575	MGCS36044_03502			*manL*	PTS transporter mannose-specific IIB & IIA component protein ManL	2430	575
576	MGCS36044_03400			*glyS*	glycine--tRNA ligase beta subunit GlyS	2422	576
577	MGCS36044_03388			-	FadH2 family NAD(FAD)-dependent dehydrogenase	2421	577
578	MGCS36044_04232			-	transglycosylase SLT domain-containing protein	2420	578
579	MGCS36044_03874			*gltX*	glutamate--tRNA ligase	2407	579
580	MGCS36044_01800			-	L10_leader RNA	2405	580
581	MGCS36044_04168			-	helix-turn-helix domain-containing transcriptional regulator	2398	581
582	MGCS36044_01758	Secreted		-	extracellular cell wall anchored bifunctional metallophosphatase/5'-nucleotidase	2393	582
583	MGCS36044_02870			*dnaX*	DNA polymerase III gamma/tau subunit DnaX	2388	583

No.	Locus tag	SignalP6 predicted[1]	Virulence[2]	Gene	Function	RPKMs in NHP 15[3]	RANK[4]
584	MGCS36044_00552			*polC*	DNA polymerase III PolC	2386	584
585	MGCS36044_00556			-	NAD(P)H-dependent oxidoreductase	2385	585
586	MGCS36044_00546			*rseP*	RIP metalloprotease RseP	2383	586
587	MGCS36044_00592			-	NYN domain-containing protein	2382	587
588	MGCS36044_01002			*serC*	3-phosphoserine/phosphohydroxythreonine transaminase SerC	2373	588
589	MGCS36044_01050			-	Gfo/Idh/MocA family oxidoreductase	2359	589
590	MGCS36044_01628			-	glycosyltransferase family 1 protein	2357	590
591	MGCS36044_00106			*prs*	ribose-phosphate pyrophosphokinase PrsA	2355	591
592	MGCS36044_00118			*purF*	amidophosphoribosyltransferase PurF	2352	592
593	MGCS36044_01036			*brnQ_2*	branched-chain amino acid transport system II carrier protein BrnQ	2347	593
594	MGCS36044_03588			-	HAD-related haloacid dehalogenase hydrolase	2346	594
595	MGCS36044_00004			*dnaN*	DNA polymerase III subunit beta protein DnaN	2344	595
596	MGCS36044_01252			-	Cof-type HAD-IIB family hydrolase	2341	596
597	MGCS36044_00548			*proS*	prolyl-tRNA synthetase ProS	2340	597
598	MGCS36044_04106			-	hypothetical protein	2334	598
599	MGCS36044_00886			*dnaI*	primosomal protein DnaI	2330	599
600	MGCS36044_00824			*yqeG*	HAD IIIA-type phosphatase YqeG	2327	600
601	MGCS36044_00504			-	M42 family metallopeptidase	2325	601
602	MGCS36044_03294		Virulence	*liaR*	three component system signal transduction response regulator protein LiaR	2319	602
603	MGCS36044_00806			*oppF_1*	oligopeptide ABC transporter ATP-binding protein OppF	2311	603
604	MGCS36044_01058			*macP*	cell wall synthase accessory phosphoprotein MacP	2305	604
605	MGCS36044_04166			*pezT*	zeta toxin family protein PezT	2304	605
606	MGCS36044_03038			-	NUDIX hydrolase	2296	606
607	MGCS36044_01274			-	NUDIX hydrolase superfamily protein	2290	607
608	MGCS36044_01028			*metS*	methionine--tRNA synthase MetS	2286	608
609	MGCS36044_03368			*ftsL*	cell division protein FtsL	2283	609
610	MGCS36044_03620			*acpS*	AcpS family provisional 4'-phosphopantetheinyl transferase	2276	610
611	MGCS36044_03898			*gpsA*	NAD(P)H-dependent glycerol-3-phosphate dehydrogenase GpsA	2269	611
612	MGCS36044_01676			-	L21_leader RNA	2268	612
613	MGCS36044_04056			*hutG*	formiminoglutamase HutG	2267	613
614	MGCS36044_00794	Secreted		*dacA_1*	secreted D-alanyl-D-alanine carboxypeptidase DacA	2258	614

No.	Locus tag	SignalP6 predicted(1)	Virulence(2)	Gene	Function	RPKMs in NHP 15(3)	RANK(4)
615	MGCS36044_01648			*rpmI*	50S ribosomal L35 protein RpmL	2257	615
616	MGCS36044_01794			*engB*	ribosome biogenesis GTP-binding protein EngB	2254	616
617	MGCS36044_01348			*ftsW*	cell division protein FtsW	2249	617
618	MGCS36044_02314			*spxA_1*	transcriptional regulator SpxA	2247	618
619	MGCS36044_02002			*prfA*	peptide chain release factor 1 PrfA	2243	619
620	MGCS36044_01484			*fldA*	flavodoxin FldA	2242	620
621	MGCS36044_01314			-	YdbC family protein	2238	621
622	MGCS36044_02316			*ribF*	bifunctional riboflavin kinase/FAD synthetase RibF	2238	621
623	MGCS36044_03602			-	CydD-related ABC transporter ATPase/permease	2238	621
624	MGCS36044_03720			-	BaeS family TCS sensor histidine kinase	2228	624
625	MGCS36044_03610	Lipo		*htsA*	heme ABC transporter substrate-binding lipoprotein HtsA	2223	625
626	MGCS36044_03044			*ileS*	isoleucine--tRNA synthetase IleS	2222	626
627	MGCS36044_01500			*ezrA*	cell division septation ring formation regulator EzrA	2216	627
628	MGCS36044_03348			*pncB*	nicotinate phosphoribosyltransferase PncB	2210	628
629	MGCS36044_03880			-	LytTR family transcriptional regulator	2207	629
630	MGCS36044_01764			*sptS*	SptS-like TCS sensor histidine kinase	2202	630
631	MGCS36044_03538			*phaB*	enoyl-CoA hydratase protein PhaB	2200	631
632	MGCS36044_01278			*pnpR*	phosphate TCS signal transduction response regulator PnpR	2195	632
633	MGCS36044_02962			*prfC*	peptide chain release factor 3 PrfC	2195	632
634	MGCS36044_01760			-	GTP pyrophosphokinase family protein	2192	634
635	MGCS36044_01178			*smpB*	SsrA(tmRNA)-binding protein SmpB	2191	635
636	MGCS36044_00394			*pflC*	pyruvate formate-lyase activating enzyme PflC	2186	636
637	MGCS36044_00940			-	PgpB family membrane-associated phospholipid phosphatase	2185	637
638	MGCS36044_00346			*pepA*	glutamyl aminopeptidase PepA	2184	638
639	MGCS36044_02136			*ffh*	signal recognition particle protein	2180	639
640	MGCS36044_03354			-	DUF4059 family protein	2172	640
641	MGCS36044_04080			*yaaA*	peroxide stress protein YaaA	2170	641
642	MGCS36044_01530	Secreted		-	cell surface extracellular nuclease	2167	642
643	MGCS36044_00558			*def*	peptide deformylase Def	2166	643
644	MGCS36044_02682			*glnQ_2*	glutamine transport ATP-binding protein GlnQ	2166	643
645	MGCS36044_03096			-	IS110 family transposase	2165	645

No.	Locus tag	SignalP6 predicted[1]	Virulence[2]	Gene	Function	RPKMs in NHP 15[3]	RANK[4]
646	MGCS36044_00802			*oppC_1*	oligopeptide ABC transporter permease protein OppC	2161	646
647	MGCS36044_00154			*adhE*	bifunctional acetaldehyde-CoA/alcohol dehydrogenase AdhE	2147	647
648	MGCS36044_03070			*typA*	translational GTPase TypA	2145	648
649	MGCS36044_04028			*cspA*	cold-shock protein CspA	2144	649
650	MGCS36044_00656			-	Txe/YoeB family addiction module toxin	2139	650
651	MGCS36044_02268			*pgmA*	phospho-sugar mutase PgmA	2129	651
652	MGCS36044_04228			*sdhA*	L-serine dehydratase alpha subunit SdhA	2122	652
653	MGCS36044_03952			*proW*	proline/glycine betaine ABC transporter ATPase component ProV	2120	653
654	MGCS36044_04014	Secreted		*pepD_2*	secreted dipeptidase PepD	2114	654
655	MGCS36044_00448			-	radical SAM protein	2113	655
656	MGCS36044_01590			*rexA*	ATP-dependent nuclease A subunit RexA	2110	656
657	MGCS36044_00994			*yabA*	DNA replication initiation control protein YabA	2107	657
658	MGCS36044_03430			*sgcC*	galactitol-specific PTS transporter IIB subunit SgcC	2100	658
659	MGCS36044_03966			*ulaD*	3-keto-L-gulonate 6-phosphate decarboxylase protein UlaD	2095	659
660	MGCS36044_01476			-	GNAT family N-acetyltransferase	2081	660
661	MGCS36044_00542			*uppS*	UDP pyrophosphate synthase UppS	2068	661
662	MGCS36044_03108			*rbsR*	ribose transport operon repressor RbsR	2068	661
663	MGCS36044_04184			-	DUF368 domain-containing protein	2063	663
664	MGCS36044_01718	Secreted		*sdrD*	SdrD superfamily cell surface extracellular protein	2062	664
665	MGCS36044_02614			*apt*	adenine phosphoribosyltransferase Apt	2062	664
666	MGCS36044_03580			-	cysteine hydrolase	2060	666
667	MGCS36044_00310			*pbp1B*	bifunctional PG transglycosylase-transpeptidase, penicillin-binding protein PBP1B	2046	667
668	MGCS36044_01634			-	RfbX superfamily lipopolysaccharide biosynthesis protein	2044	668
669	MGCS36044_01550			*atpE*	ATP synthase C subunit AtpE	2042	669
670	MGCS36044_03476			*trmB*	tRNA (guanosine(46)-N7)-methyltransferase TrmB	2033	670
671	MGCS36044_01882			*copB*	copper-exporting ATPase cCopB	2032	671
672	MGCS36044_01728			*apbA*	2-dehydropantoate 2-reductase	2024	672
673	MGCS36044_02892			*nrdE_2*	class 1b ribonucleoside-diphosphate reductase alpha subunit NrdE	2024	672
674	MGCS36044_00884			*dnaB*	replication initiation and membrane attachment protein DnaB	2023	674
675	MGCS36044_01104			*dgkA*	diacylglycerol kinase DgkA	2008	675
676	MGCS36044_01214			*dhaK*	dihydroxyacetone kinase subunit DhaK	2005	676

No.	Locus tag	SignalP6 predicted[(1)]	Virulence[(2)]	Gene	Function	RPKMs in NHP 15[(3)]	RANK[(4)]
677	MGCS36044_00732			-	DUF436 family protein	2003	677
678	MGCS36044_01620			*rgpF*	alpha-L-Rha alpha-1,3-L-rhamnosyltransferase RgpF	1990	678
679	MGCS36044_01954			-	AI-2E family transporter	1979	679
680	MGCS36044_03598			*cbiQ_1*	cobalt ECF transporter (T) transmembrane component CbiQ	1977	680
681	MGCS36044_03062			*murG*	UDP-N-acetylglucosamine--N-acetylmuramyl- (pentapeptide) pyrophosphoryl-undecaprenol N-acetylglucosamine transferase MurG	1974	681
682	MGCS36044_04092			-	DUF2079 domain-containing protein	1972	682
683	MGCS36044_02146			-	TCS DNA-binding response regulator (heme response regulator HssR-like)	1966	683
684	MGCS36044_01876			*copY_1*	copper transport repressor CopY	1965	684
685	MGCS36044_00570			-	PTS transporter IIC component UlaA-like	1963	685
686	MGCS36044_00344			*proC*	pyrroline-5-carboxylate reductase ProC	1958	686
687	MGCS36044_01622			-	glycosyltransferase family 2 protein	1956	687
688	MGCS36044_00426			-	hypothetical protein	1941	688
689	MGCS36044_03646			*uvrA*	excinuclease ABC subunit (A) UvrA	1939	689
690	MGCS36044_03938			-	CHY zinc finger domain-containing protein	1938	690
691	MGCS36044_01614			*rgpC*	ABC transporter polysaccharide/polyol phosphate export permease RgpC	1936	691
692	MGCS36044_03940			*tgt*	tRNA guanosine(34) transglycosylase Tgt	1934	692
693	MGCS36044_02518			-	TIGR01906 family membrane protein	1932	693
694	MGCS36044_03346			*nadE*	ammonia-dependent NAD(+) synthetase NadE	1924	694
695	MGCS36044_03600			-	ECF transporter (S) specificity component	1923	695
696	MGCS36044_03472			*rimP*	ribosome maturation factor RimP	1918	696
697	MGCS36044_04006			-	translation initiation inhibitor	1916	697
698	MGCS36044_01984			*opuBA*	OpuBA superfamily glycine/betaine ABC transporter ATP-binding protein	1912	698
699	MGCS36044_00890			*snf*	SWF/SNF family helicase	1909	699
700	MGCS36044_02832			*brkB*	BrkB family protein	1909	699
701	MGCS36044_03110			*sdrC*	SdrC family PDZ domain-containing protein	1909	699
702	MGCS36044_04230			-	HAD hydrolase-like protein	1909	699
703	MGCS36044_03114			*rsmD*	16S rRNA (guanine(966)-N(2))-methyltransferase RsmD	1906	703
704	MGCS36044_02366			*lepB_1*	signal peptidase I	1898	704
705	MGCS36044_02332			-	GntR family transcriptional regulator	1897	705
706	MGCS36044_01190			-	NAD(P)H-dependent oxidoreductase	1888	706
707	MGCS36044_02494			*msrB*	peptide-methionine (R)-S-oxide reductase MsrB	1887	707

No.	Locus tag	SignalP6 predicted(1)	Virulence(2)	Gene	Function	RPKMs in NHP 15(3)	RANK(4)
708	MGCS36044_03206		Virulence	*trxR*	TCS DNA-binding response regulator protein TrxR	1887	707
709	MGCS36044_03216			*ugpA_1*	sugar ABC transporter permease UgpA	1881	709
710	MGCS36044_01608			*rmlD*	dTDP-4-dehydrorhamnose reductase protein RmlD	1874	710
711	MGCS36044_03740			-	major facilitator superfamily protein	1872	711
712	MGCS36044_01932			*potA*	spermidine putrescine ABC transport system ATP-binding protein PotA	1871	712
713	MGCS36044_03332			*rlmL*	23S rRNA G2445 N2-methylase RlmL	1868	713
714	MGCS36044_02532			-	DisA N domain-containing diadenylate cyclase	1866	714
715	MGCS36044_01540			*pulA2*	type I pullulanase PulA	1865	715
716	MGCS36044_02728	Lipo		*nlpA*	MetQ/NlpA family ABC transporter substrate-binding lipoprotein	1863	716
717	MGCS36044_03674			*lepB_2*	signal peptidase I LepB	1863	716
718	MGCS36044_01432			-	MBL fold metallo-hydrolase	1861	718
719	MGCS36044_02470	Lipo		-	extracellular solute-binding lipoprotein	1860	719
720	MGCS36044_01342			*btuE*	BtuE superfamily glutathione peroxidase	1858	720
721	MGCS36044_01436			*aspC*	aspartate aminotransferase protein AspC	1858	720
722	MGCS36044_02444			*rpiA*	ribose-5-phosphate isomerase RpiA	1856	722
723	MGCS36044_01504			-	DUF1694 domain-containing protein	1854	723
724	MGCS36044_01792			*clpX*	ATP-dependent Clp protease, ATP-binding subunit ClpX	1848	724
725	MGCS36044_01242			*vicX*	zinc-dependent hydrolase protein VicX	1847	725
726	MGCS36044_01472			-	IS30 family transposase	1847	725
727	MGCS36044_01982			-	Na+ driven multidrug efflux pump	1840	727
728	MGCS36044_01880			*copZ_1*	copper chaperone CopZ	1835	728
729	MGCS36044_01796			-	hypothetical protein	1834	729
730	MGCS36044_03606			*htsC*	heme ABC transporter ATP-binding protein HtsC	1823	730
731	MGCS36044_02498			*Ndk*	nucleoside-diphosphate kinase Ndk	1810	731
732	MGCS36044_03764			*ybaB*	YbaB family DNA-binding protein	1807	732
733	MGCS36044_03942			-	DUF975 family protein	1803	733
734	MGCS36044_02190			-	SatD family protein	1801	734
735	MGCS36044_03562			*gatA_2*	aspartyl-tRNA(Asn) or glutamyl-tRNA(Gln) amidotransferase A subunit GatA	1798	735
736	MGCS36044_00576			-	polynucleotide phosphorylase/polyadenylase	1792	736
737	MGCS36044_01108			-	Nudix superfamily phosphohydrolase	1786	737
738	MGCS36044_00560			-	Crp family cyclic nucleotide-binding domain-containing protein	1782	738

No.	Locus tag	SignalP6 predicted[1]	Virulence[2]	Gene	Function	RPKMs in NHP 15[3]	RANK[4]
739	MGCS36044_01246			*smc*	chromosome segregation protein Smc	1780	739
740	MGCS36044_03608			*htsB*	heme ABC transporter permease HtsB	1779	740
741	MGCS36044_02306	Lipo		*ptsS*	phosphate ABC transporter substrate-binding lipoprotein PstS	1771	741
742	MGCS36044_01382			-	SSRC10 RNA	1768	742
743	MGCS36044_02200			*topA*	type I DNA topoisomerase TopA	1766	743
744	MGCS36044_04132			-	YitT family protein putative ABC transporter ATPase	1765	744
745	MGCS36044_01642			*cmk*	CMP kinase Cmk	1764	745
746	MGCS36044_01482			-	IS1548 family transposase	1761	746
747	MGCS36044_04120			*mutL*	DNA mismatch repair endonuclease MutL	1760	747
748	MGCS36044_02288			*rpsT*	30S ribosomal S20 protein RpsT	1756	748
749	MGCS36044_02850			*uxaC*	uronate isomerase	1752	749
750	MGCS36044_01074			*ftsK*	cell division protein FtsK	1748	750
751	MGCS36044_01056			*nudF*	NUDIX hydrolase NudF	1740	751
752	MGCS36044_01748			-	Uup family ATPase components of ABC transporters with duplicated ATPase domains	1738	752
753	MGCS36044_00590			*rmlB*	23S rRNA (guanosine(2251)-2'-O)-methyltransferase RlmB	1737	753
754	MGCS36044_03214			*ugpE_1*	glycerol-3-phosphate ABC transporter permease UgpE	1737	753
755	MGCS36044_01480			*add*	adenosine deaminase Add	1736	755
756	MGCS36044_02610			*nth*	endonuclease III Nth	1734	756
757	MGCS36044_00532			*dexB*	glucan 1,6-alpha-glucosidase DexB	1732	757
758	MGCS36044_03566			*ppdK*	pyruvate phosphate dikinase PpdK	1732	757
759	MGCS36044_02302			*ptsA*	phosphate ABC transporter permease PstA	1729	759
760	MGCS36044_03394			*glpK*	glycerol kinase GlpK	1727	760
761	MGCS36044_02460			*yjjG*	YjjG family noncanonical pyrimidine nucleotidase	1721	761
762	MGCS36044_03192			*aroB*	3-dehydroquinate synthase protein AroB	1717	762
763	MGCS36044_00140			*purB*	adenylosuccinate lyase PurB	1716	763
764	MGCS36044_01986			*osmF*	OsmF superfamily glycine/betaine transport system permease protein	1716	763
765	MGCS36044_03936			*bioY*	biotin transporter BioY	1713	765
766	MGCS36044_04012			-	hypothetical protein	1708	766
767	MGCS36044_02248			*fhs_1*	formate--tetrahydrofolate ligase Fhs	1701	767
768	MGCS36044_02558			*frmB*	FrmB family esterase	1694	768
769	MGCS36044_02678			*uvrB*	excinuclease ABC subunit UvrB	1692	769

No.	Locus tag	SignalP6 predicted(1)	Virulence(2)	Gene	Function	RPKMs in NHP 15(3)	RANK(4)
770	MGCS36044_02656			*malH*	LacI family transcriptional regulatory protein MalH	1690	770
771	MGCS36044_03684			-	ElaA-related predicted N-acetyltransferase	1690	770
772	MGCS36044_00554			-	MarR family transcriptional regulator	1683	772
773	MGCS36044_03706			*pepX*	PepX family Xaa-Pro dipeptidyl-peptidase	1677	773
774	MGCS36044_01962			-	DUF4649 domain-containing protein	1662	774
775	MGCS36044_00124			*vanZ*	VanZ family putative glycopeptide antibiotic resistance protein	1659	775
776	MGCS36044_00998			-	DUF5684 domain-containing protein	1657	776
777	MGCS36044_02658			*malA*	maltodextrose utilization protein MalA	1657	776
778	MGCS36044_03962			*ulaF*	L-ribulose-5-phosphate 4-epimerase UlaF	1655	778
779	MGCS36044_04054			*hutH*	histidine ammonia-lyase HutH	1641	779
780	MGCS36044_00358			*dus*	tRNA-dihydrouridine synthase Dus	1633	780
781	MGCS36044_01738	Lipo		-	putative peptidoglycan hydrolase lipoprotein	1633	780
782	MGCS36044_00424			-	putative transcriptional regulator	1629	782
783	MGCS36044_02358			-	amino acid ABC transporter ATP-binding protein	1624	783
784	MGCS36044_00290			*cydA*	cytochrome ubiquinol oxidase subunit (I) CydA	1621	784
785	MGCS36044_00278			-	HNH endonuclease	1618	785
786	MGCS36044_01184			*wcaA*	WcaA superfamily glycosyltransferase involved in cell wall biosynthesis	1614	786
787	MGCS36044_04046			-	formiminotetrahydrofolate cyclodeaminase	1612	787
788	MGCS36044_00882			*nrdR*	transcriptional regulator NrdR	1611	788
789	MGCS36044_02262			*coaB*	phosphopantothenate--cysteine ligase CoaB	1611	788
790	MGCS36044_01744			*degV_1*	DegV family protein	1608	790
791	MGCS36044_01538			*dagK*	diacylglycerol kinase family lipid kinase	1598	791
792	MGCS36044_01966			*nifS_2*	NifS superfamily cysteine desulfurase	1598	791
793	MGCS36044_01498			*gyrB*	DNA topoisomerase ATP-hydrolyzing B subunit GyrB	1596	793
794	MGCS36044_02360			-	amino acid ABC transporter permease	1594	794
795	MGCS36044_02654			*malG*	maltose/maltodextrin ABC transport system permease protein MalG	1585	795
796	MGCS36044_03010			*ispA*	IspA family geranylgeranyl pyrophosphate synthase	1584	796
797	MGCS36044_00948			*pflA*	pyruvate formate-lyase activating enzyme PflA	1576	797
798	MGCS36044_02412			*ung*	uracil-DNA glycosylase Ung	1572	798
799	MGCS36044_03736			-	MFS transporter	1571	799
800	MGCS36044_03428			*lacA*	galactose-6-phosphate isomerase subunit LacA	1563	800

No.	Locus tag	SignalP6 predicted[1]	Virulence[2]	Gene	Function	RPKMs in NHP 15[3]	RANK[4]
801	MGCS36044_03392			*glpO*	type 1 glycerol-3-phosphate oxidase GlpO	1561	801
802	MGCS36044_02628			-	DUF3042 family protein	1559	802
803	MGCS36044_01174			*secG*	preprotein translocase subunit SecG	1555	803
804	MGCS36044_03432			*sgaB*	galactitol-specific PTS transporter IIC subunit SgaB	1555	803
805	MGCS36044_00582			*cysS*	cysteine--tRNA synthetase CysS	1554	805
806	MGCS36044_01072	Lipo		-	peptidylprolyl isomerase lipoprotein	1552	806
807	MGCS36044_00352			-	DUF4479 and tRNA-binding domain-containing protein	1551	807
808	MGCS36044_03116			*asnA*	asparagine synthetase AsnA	1551	807
809	MGCS36044_03370			*mraW*	S-adenosyl-methyltransferase MraW	1550	809
810	MGCS36044_00488			*pabB*	para-aminobenzoate synthetase component I/4-amino-4-deoxychorismate lyase PabB	1548	810
811	MGCS36044_01488			*clcA*	voltage-gated chloride channel family protein ClcA	1546	811
812	MGCS36044_02298			*ptsB1*	phosphate ABC transporter ATP-binding protein PstB1	1545	812
813	MGCS36044_02004			*prmC*	peptide chain release factor N(5)-glutamine methyltransferase PrmC	1535	813
814	MGCS36044_02804			*ecfT*	ECF transporter transmembrane protein EcfT	1534	814
815	MGCS36044_03014			*xseA*	exodeoxyribonuclease VII large subunit XseA	1533	815
816	MGCS36044_02800			-	MdlB family multidrug ABC transporter ATPase and permease	1524	816
817	MGCS36044_03862	Lipo		*yidC_2*	YidC/Oxa1 family membrane protein insertase lipoprotein	1524	816
818	MGCS36044_04040			*hutI*	imidazolonepropionase HutI	1517	818
819	MGCS36044_01914			-	polysaccharide deacetylase family protein	1515	819
820	MGCS36044_01582			-	ABC transporter permease	1505	820
821	MGCS36044_04140			*hisS*	histidine--tRNA synthase HisS	1495	821
822	MGCS36044_03570			*ccpN*	CcpN family CBS pair domain transcriptional regulator	1491	822
823	MGCS36044_03204			*lacZ*	beta-galactosidase LacZ	1484	823
824	MGCS36044_01616			*rgpD*	ABC transporter polysaccharide/polyol phosphate ATPase component RgpD	1480	824
825	MGCS36044_03894			-	ABC transporter ATPase/permease	1478	825
826	MGCS36044_03484			*hit*	HIT family protein	1472	826
827	MGCS36044_02356	Lipo		-	amino acid ABC transporter substrate-binding lipoprotein	1471	827
828	MGCS36044_03208		Virulence	*trxS*	TCS sensor histidine kinase TrxS	1470	828
829	MGCS36044_00962			*murE_1*	UDP-N-acetylmuramoyl-L-alanyl-D-glutamate--L- lysine ligase MurE	1465	829
830	MGCS36044_03714	Lipo		-	chromosome assembly-related lipoprotein	1462	830
831	MGCS36044_02028			*srtA*	class A sortase SrtA	1450	831

No.	Locus tag	SignalP6 predicted(1)	Virulence(2)	Gene	Function	RPKMs in NHP 15(3)	RANK(4)
832	MGCS36044_02646			*malR*	maltose operon transcriptional repressor MalR	1445	832
833	MGCS36044_01652			*ltaS*	LTA synthase LtaS	1444	833
834	MGCS36044_01340	Lipo		-	DUF4430 domain-containing lipoprotein	1443	834
835	MGCS36044_00524			-	ISAs1 family transposase	1442	835
836	MGCS36044_02534			*murE_2*	UDP-N-acetylmuramoylalanyl-D-glutamate-2, 6-diaminopimelate ligase MurE	1442	835
837	MGCS36044_00156			*adhP*	alcohol dehydrogenase AdhP	1441	837
838	MGCS36044_00342			-	hypothetical protein	1441	837
839	MGCS36044_03652			*rpsR*	30S ribosomal S18 protein RpsR	1437	839
840	MGCS36044_04136			-	YitT family protein putative ABC transporter ATPase	1434	840
841	MGCS36044_03170			*valS*	valine--tRNA synthetase ValS	1428	841
842	MGCS36044_01596			-	IS1548 family transposase	1419	842
843	MGCS36044_02484			*yeiH*	YeiH family membrane protein	1418	843
844	MGCS36044_04000			*nusG*	transcription antitermination protein NusG	1418	843
845	MGCS36044_01090			*msrA*	peptide-methionine (S)-S-oxide reductase MsrA	1417	845
846	MGCS36044_03000			-	DegV family EDD domain-containing protein	1414	846
847	MGCS36044_00734			-	mechanosensitive ion channel family protein	1412	847
848	MGCS36044_00762			-	IS1548 family transposase	1410	848
849	MGCS36044_02986			-	Spy1186876 RNA	1407	849
850	MGCS36044_03132			*fnr*	Crp/Fnr family transcriptional regulator	1403	850
851	MGCS36044_02432			-	LysR family transcriptional regulator	1402	851
852	MGCS36044_02956			*metP_2*	methionine uptake ABC transporter permease MetP	1401	852
853	MGCS36044_02282			*deoA*	pyrimidine-nucleoside phosphorylase DeoA	1400	853
854	MGCS36044_01250			-	IS1548 family transposase	1398	854
855	MGCS36044_00292			*cydB*	cytochrome d ubiquinol oxidase subunit (II) CydB	1396	855
856	MGCS36044_03414			*degV_2*	DegV family fatty acid-binding protein	1395	856
857	MGCS36044_02026			*gyrA*	DNA gyrase subunit A GyrA	1388	857
858	MGCS36044_01106			*era*	GTPase Era	1387	858
859	MGCS36044_03744			-	type I restriction-modification system endonuclease (S) specificity subunit	1380	859
860	MGCS36044_00280			-	XRE family ImmR-like transcriptional regulator	1376	860
861	MGCS36044_00580			-	hypothetical protein	1376	860
862	MGCS36044_01968			*ribP*	ribose-phosphate pyrophosphokinase RibP	1367	862

No.	Locus tag	SignalP6 predicted[1]	Virulence[2]	Gene	Function	RPKMs in NHP 15[3]	RANK[4]
863	MGCS36044_03134			*argR_2*	arginine responsive transcriptional repressor ArgR	1357	863
864	MGCS36044_01664			-	dihydrofolate synthase	1356	864
865	MGCS36044_02368			-	IS1548 family transposase	1353	865
866	MGCS36044_03092			-	IS1548 family transposase	1353	865
867	MGCS36044_04214			-	IS1548 family transposase	1353	865
868	MGCS36044_01270			-	permease	1352	868
869	MGCS36044_01910			*xerD_2*	site-specific integrase	1351	869
870	MGCS36044_03358			*hisM*	HisM family amino acid ABC transporter permease	1347	870
871	MGCS36044_03672			*rnhC*	HIII ribonuclease RnhC	1344	871
872	MGCS36044_03888			*dut*	deoxyuridine 5'-triphosphate nucleotidohydrolase protein Dut	1339	872
873	MGCS36044_02896			*clcB*	voltage-gated ClC-type chloride channel ClcB	1335	873
874	MGCS36044_03722			-	OmpR family TCS DNA-binding response regulator	1331	874
875	MGCS36044_04208			*dnaC*	replicative DNA helicase DnaC	1330	875
876	MGCS36044_03604			-	CydC-related ABC transporter ATPase/permease	1325	876
877	MGCS36044_00992			*yaaT*	cell fate regulator YaaT	1324	877
878	MGCS36044_01632			*galE*	UDP-glucose 4-epimerase GalE	1301	878
879	MGCS36044_03626			*manA*	mannose-6-phosphate isomerase ManA	1300	879
880	MGCS36044_00574			*pnp*	polyribonucleotide nucleotidyltransferase Pnp	1298	880
881	MGCS36044_01254			-	Cof-type HAD-IIB family hydrolase	1297	881
882	MGCS36044_03324			-	3-hydroxybutyrate dehydrogenase	1297	881
883	MGCS36044_00898			*mltG*	endolytic transglycosylase MltG	1296	883
884	MGCS36044_01658			*aroC*	chorismate synthase AroC	1296	883
885	MGCS36044_01772			-	isopentenyl-diphosphate delta-isomerase	1296	883
886	MGCS36044_01206			-	ABC transporter permease component	1289	886
887	MGCS36044_04118			*mdtH*	MdtH-related MFS multidrug resistance transporter	1285	887
888	MGCS36044_00568			-	PTS transporter IIB component UlaB-like	1280	888
889	MGCS36044_02238			*cls*	cardiolipin synthase	1280	888
890	MGCS36044_03892			-	ABC transporter ATPase/permease	1275	890
891	MGCS36044_00500			*rgfB*	endonuclease/exonuclease/phosphatase family protein	1273	891
892	MGCS36044_00708			-	IS30 family transposase	1273	891
893	MGCS36044_02304			*ptsC*	phosphate ABC transporter permease PstC	1273	891

No.	Locus tag	SignalP6 predicted[1]	Virulence[2]	Gene	Function	RPKMs in NHP 15[3]	RANK[4]
894	MGCS36044_03494			*azgA*	AzgA family permease	1272	894
895	MGCS36044_01100			-	uracil DNA glycosylase superfamily protein	1270	895
896	MGCS36044_03238			-	LLM class flavin-dependent oxidoreductase	1269	896
897	MGCS36044_03866		Virulence	*fasX*	FasX RNA	1269	896
898	MGCS36044_02968			*ddl*	D-alanine--D-alanine ligase Ddl	1264	898
899	MGCS36044_03934			-	metallo-beta-lactamase superfamily protein	1260	899
900	MGCS36044_00320			*comYA*	competence system type II/IV secretion system protein ComYA	1258	900
901	MGCS36044_00146			*ruvB*	Holliday junction branch migration DNA helicase RvuB	1257	901
902	MGCS36044_04042			*hutU*	urocanate hydratase HutU	1249	902
903	MGCS36044_01268			-	DUF1980 domain-containing protein	1242	903
904	MGCS36044_00946		Virulence	*hlyX*	hemolysin family protein HylX	1235	904
905	MGCS36044_01536			*ligA*	NAD-dependent DNA ligase LigA	1235	904
906	MGCS36044_03398			*ynzC*	DUF896 family protein	1234	906
907	MGCS36044_01052			-	UhpC family MFS transporter	1231	907
908	MGCS36044_01584			-	ABC transporter ATP-binding component LolD-like	1231	907
909	MGCS36044_01130			-	IS1548 family transposase	1230	909
910	MGCS36044_03386			-	putative transcriptional regulator	1229	910
911	MGCS36044_00350			*trxA_1*	thioredoxin family protein TrxA-like	1227	911
912	MGCS36044_00772			-	ABC amino acid transporter ATP-binding protein	1227	911
913	MGCS36044_01770			*mvaK2*	mevalonate kinase MvaK2	1227	911
914	MGCS36044_01044			*fabG_1*	3-oxoacyl-ACP reductase FabG	1223	914
915	MGCS36044_03448			*srtB*	pilus polymerization class B sortase SrtB	1223	914
916	MGCS36044_02720			*fetB*	iron export ABC transporter permease subunit FetB	1222	916
917	MGCS36044_00808			-	IS30 family transposase	1214	917
918	MGCS36044_02144			*yhhX*	PRK10206 superfamily putative oxidoreductase	1213	918
919	MGCS36044_03328			*luxS*	S-ribosylhomocysteine lyase	1211	919
920	MGCS36044_02904			*alaS*	alanine--tRNA synthetase AlaS	1200	920
921	MGCS36044_03408			*nagA*	N-acetylglucosamine-6-phosphate deacetylase NagA	1199	921
922	MGCS36044_00600			-	IS30 family transposase	1198	922
923	MGCS36044_01236			*glnQ_1*	glutamine ABC transporter ATPase GlnQ	1192	923
924	MGCS36044_01646			*infC*	translation initiation factor InfC	1192	923

No.	Locus tag	SignalP6 predicted[1]	Virulence[2]	Gene	Function	RPKMs in NHP 15[3]	RANK[4]
925	MGCS36044_01618			*rgpE*	glycosyltransferase family GT2 protein RgpE	1190	925
926	MGCS36044_01462			-	PTS sugar transport IIB subunit	1189	926
927	MGCS36044_01624			-	DUF2304 domain-containing protein	1188	927
928	MGCS36044_04048			*fhs_2*	formate--tetrahydrofolate ligase Fhs	1179	928
929	MGCS36044_00828			*yhbY*	ribosome assembly RNA-binding protein YhbY	1176	929
930	MGCS36044_01000			*cutC*	copper homeostasis protein CutC	1173	930
931	MGCS36044_02722			*fetA*	iron export ABC transporter ATP-binding subunit FetA	1169	931
932	MGCS36044_00022			-	hypothetical protein	1168	932
933	MGCS36044_00116			*purL*	phosphoribosylformylglycinamidine synthase PurL	1166	933
934	MGCS36044_00760			*rpmB*	50S ribosomal L28 protein RpmB	1166	933
935	MGCS36044_02300			*ptsB2*	phosphate ABC transporter ATP-binding protein PstB2	1160	935
936	MGCS36044_01938			*potD*	spermidine putrescine ABC transport system substrate-binding protein PotD	1157	936
937	MGCS36044_03770			-	DUF536 domain-containing protein	1156	937
938	MGCS36044_03668			-	colicin V production family protein	1155	938
939	MGCS36044_04210			*rplI*	50S ribosomal L9 protein RplI	1154	939
940	MGCS36044_04272			*rlmH*	23S rRNA (pseudouridine(1915)-N(3))-methyltransferase RlmH	1154	939
941	MGCS36044_03568			-	kinase/pyrophosphorylase	1153	941
942	MGCS36044_00024	Secreted		-	class A beta-lactamase-related serine hydrolase	1146	942
943	MGCS36044_01326			*yccF*	YccF domain-containing protein	1141	943
944	MGCS36044_02846			-	SDR family D-mannonate oxidoreductase	1137	944
945	MGCS36044_00918			-	YfcE family metallophosphoesterase	1134	945
946	MGCS36044_03426			*lacB*	galactose-6-phosphate isomerase subunit LacB	1131	946
947	MGCS36044_01926			*folQ*	dihydroneopterin aldolase protein FolB	1129	947
948	MGCS36044_02504			-	CRISPR-DR22 RNA	1129	947
949	MGCS36044_02506			-	CRISPR-DR22 RNA	1129	947
950	MGCS36044_02496			*lepA*	translation elongation factor 4 LepA	1127	950
951	MGCS36044_01098			*phoH*	phosphate starvation-inducible protein PhoH	1125	951
952	MGCS36044_02450			*nfnB*	NfnB family nitroreductase	1125	951
953	MGCS36044_01752			-	MdlB family multidrug ABC transporter ATPase and permease component	1119	953
954	MGCS36044_03188			-	hypothetical protein	1116	954
955	MGCS36044_01936			*potC*	spermidine putrescine ABC transport system permease protein PotC	1115	955

No.	Locus tag	SignalP6 predicted[(1)]	Virulence[(2)]	Gene	Function	RPKMs in NHP 15[(3)]	RANK[(4)]
956	MGCS36044_00506			-	ECF transporter S component	1111	956
957	MGCS36044_02156			-	NusG domain II-containing protein	1109	957
958	MGCS36044_01464			-	PTS sugar transport IIC subunit	1108	958
959	MGCS36044_03954			*proV*	proline/glycine betaine ABC transporter permease component ProW	1107	959
960	MGCS36044_00868			*ktrB*	potassium uptake transporter channel subunit KtrB	1103	960
961	MGCS36044_02402			*parC*	DNA topoisomerase IV subunit A ParC	1102	961
962	MGCS36044_03932			*tadA*	tRNA adenosine(34) deaminase TadA	1101	962
963	MGCS36044_02950			*kup*	potassium uptake protein Kup	1100	963
964	MGCS36044_03726			-	GNAT family acetyltransferase	1099	964
965	MGCS36044_00952			-	cytoplasmic protein	1098	965
966	MGCS36044_02000			*tdk*	thymidine kinase Tdk	1098	965
967	MGCS36044_01410	Secreted	Virulence	*hylB*	secreted hyaluronate lyase HylB	1095	967
968	MGCS36044_02996			-	DUF2140 domain-containing protein	1093	968
969	MGCS36044_04010			*rluA_2*	RluA family pseudouridine synthase	1080	969
970	MGCS36044_00598			-	helix-turn-helix transcriptional regulator	1077	970
971	MGCS36044_02186			*gid*	tRNA (uracil-5-)-methyltransferase/glucose inhibited division protein (A) GidA	1076	971
972	MGCS36044_04112			-	VOC family protein	1073	972
973	MGCS36044_00956			*fhuB*	iron ABC transporter permease FhuB	1071	973
974	MGCS36044_01294			*tex*	RNA-binding transcriptional accessory protein Tex	1059	974
975	MGCS36044_00544			*cdsA*	phosphatidate cytidylyltransferase CdsA	1050	975
976	MGCS36044_01394			*agaW*	PTS transporter hyaluronate-oligosaccharide-specific IIC component AgaW	1046	976
977	MGCS36044_04114			*tag*	DNA-3-methyladenine glycosylase Tag	1045	977
978	MGCS36044_03864			*rnpA*	ribonuclease P protein component RnpA	1044	978
979	MGCS36044_04142			*rpmF*	50S ribosomal L32 protein RpmF	1044	978
980	MGCS36044_00308			*tyrS*	tyrosyl-tRNA synthetase TyrS	1040	980
981	MGCS36044_03670			-	hypothetical protein	1039	981
982	MGCS36044_01084			*pyrH*	UMP kinase PyrH	1038	982
983	MGCS36044_03834			*rrmV*	5S rRNA maturation endonuclease RnmV	1038	982
984	MGCS36044_01702			*carB*	carbamoyl-phosphate synthase large subunit CarB	1032	984
985	MGCS36044_00508			*rihB*	pyrimidine-specific ribonucleoside hydrolase RihB	1029	985
986	MGCS36044_01376			-	sugar-phosphatase	1029	985

No.	Locus tag	SignalP6 predicted(1)	Virulence(2)	Gene	Function	RPKMs in NHP 15(3)	RANK(4)
987	MGCS36044_03074			*glcK*	glucokinase GlcK	1029	985
988	MGCS36044_03438			*copZ_2*	copper chaperone CopZ	1028	988
989	MGCS36044_03478			*cotS*	CotS family thiamine kinase	1026	989
990	MGCS36044_00914			*murI*	glutamate racemase MurI	1024	990
991	MGCS36044_03902			-	DUF6198 family protein	1023	991
992	MGCS36044_02142			-	helix-turn-helix transcriptional regulator	1022	992
993	MGCS36044_04240			*pgsA*	CDP-diacylglycerol--glycerol-3-phosphate 3-phosphatidyltransferase PgsA	1021	993
994	MGCS36044_01602			*dnaG*	DNA primase protein DnaG	1019	994
995	MGCS36044_03998	Secreted	Virulence	*nga*	secreted nicotine adenine dinucleotide glycohydrolase Nga	1019	994
996	MGCS36044_03970			*ulaB*	ascorbate-specific PTS transporter EIIB protein UlaB	1018	996
997	MGCS36044_00782			*rgpG*	undecaprenyl/decaprenyl-phosphate alpha-N-acetylglucosaminyl 1-phosphate transferase RgpG	1015	997
998	MGCS36044_01374			*murM*	peptidoglycan lipid II--L-alanine ligase protein MurM	1014	998
999	MGCS36044_03930	Lipo		-	CYK3 family lipoprotein putatively involved in cell division and chromosome partitioning	1014	998
1000	MGCS36044_04236			*cbiO2*	cobalt ABC transporter ATPase CbiO1	1014	998
1001	MGCS36044_03200			-	permease	1013	1001
1002	MGCS36044_01328			-	aminoacyl-tRNA deacylase	1012	1002
1003	MGCS36044_04124			-	YmcA-related cell fate/competence/biofilm development regulator	1002	1003
1004	MGCS36044_02502			-	CRISPR-DR22 RNA	994	1004
1005	MGCS36044_01956			*radC*	DNA repair protein RadC	993	1005
1006	MGCS36044_01282	Lipo		*pstS*	phosphate ABC transporter substrate-binding lipoprotein PstS	988	1006
1007	MGCS36044_01196			*amyS*	alpha-amylase AmyS	985	1007
1008	MGCS36044_03340			*recU*	Holliday junction resolvase RecU	984	1008
1009	MGCS36044_00164			-	IS30 family transposase	983	1009
1010	MGCS36044_01788			*dyr*	dihydrofolate reductase Dyr	983	1009
1011	MGCS36044_04226			*sdhB*	L-serinedehydratase beta subunit SdhB	982	1011
1012	MGCS36044_02034	Lipo		-	streptococcal histidine triad (HIT) lipoprotein	980	1012
1013	MGCS36044_01700			*carA*	carbamoyl-phosphate synthase small subunit CarA	979	1013
1014	MGCS36044_03254			-	DUF960 domain-containing protein	977	1014
1015	MGCS36044_03072			*pspE*	PspE family rhodanese-like domain-containing protein	972	1015
1016	MGCS36044_03176			-	VOC family protein	971	1016

No.	Locus tag	SignalP6 predicted(1)	Virulence(2)	Gene	Function	RPKMs in NHP 15(3)	RANK(4)
1017	MGCS36044_03884			-	carbonic anhydrase	970	1017
1018	MGCS36044_00744			*pyrG*	CTP synthase Pyg	967	1018
1019	MGCS36044_03968			*ulaC*	ascorbate-specific PTS transporter EIIA protein UlaC	967	1018
1020	MGCS36044_00006			-	DUF951 domain-containing protein	966	1020
1021	MGCS36044_03910			-	IS30 family transposase	963	1021
1022	MGCS36044_04138			*aspS*	aspartyl-tRNA synthetase	958	1022
1023	MGCS36044_04016			*mutT*	8-oxo-dGTP diphosphatase, DNA mismatch repair protein MutT	956	1023
1024	MGCS36044_03906			-	5-formyltetrahydrofolate cyclo-ligase	953	1024
1025	MGCS36044_03732			-	PLDc N-terminal domain-containing protein	952	1025
1026	MGCS36044_01088			*cvfB*	S1 RNA-binding domain-containing protein CvfB	948	1026
1027	MGCS36044_02984			*pyrD*	dihydroorotate dehydrogenase PyrD	946	1027
1028	MGCS36044_01878			*copA_1*	copper-exporting ATPase CopA	928	1028
1029	MGCS36044_01014			*exoA*	exodeoxyribonuclease III protein ExoA	923	1029
1030	MGCS36044_00340			-	CAAX amino terminal protease family membrane protein	922	1030
1031	MGCS36044_04052			*potE*	PotE family amino acid transporter	918	1031
1032	MGCS36044_02702			-	hypothetical protein	916	1032
1033	MGCS36044_03022			*phrB*	PhrB family deoxyribodipyrimidine photolyase	916	1032
1034	MGCS36044_02930			*queA*	S-adenosylmethionine ribosyltransferase-isomerase QueA	914	1034
1035	MGCS36044_03446	Secreted		-	pilus ancillary/minor protein 2	914	1034
1036	MGCS36044_02250			*lplA_1*	lipoate--protein ligase	911	1036
1037	MGCS36044_02542			-	PadR family transcriptional regulator	908	1037
1038	MGCS36044_01960			*rex*	redox-sensing transcriptional repressor Rex	904	1038
1039	MGCS36044_00974			-	YlbG family protein	902	1039
1040	MGCS36044_02612			*dnaD*	DNA replication protein DnaD	901	1040
1041	MGCS36044_03964			*ulaE*	L-xylulose 5-phosphate 3-epimerase UlaE	896	1041
1042	MGCS36044_03060			*ftsQ*	cell division protein FtsQ/DivIB	894	1042
1043	MGCS36044_02474			-	YesM family TCS sensor histidine kinase	890	1043
1044	MGCS36044_03582			*codY*	CodY family GTP-sensing pleiotropic transcriptional regulator	890	1043
1045	MGCS36044_03076			-	DUF910 domain-containing protein	888	1045
1046	MGCS36044_03578			-	DUF156 family protein	886	1046
1047	MGCS36044_04072			-	Spy491738 RNA	882	1047

No.	Locus tag	SignalP6 predicted[1]	Virulence[2]	Gene	Function	RPKMs in NHP 15[3]	RANK[4]
1048	MGCS36044_03226			-	beta-N-acetylglucosaminidase	878	1048
1049	MGCS36044_03402			*glyQ*	glycine--tRNA ligase alpha subunit GlyQ	878	1048
1050	MGCS36044_00800			*oppB_1*	oligopeptide ABC transporter permease protein OppB	873	1050
1051	MGCS36044_01226			*ppsB*	long-chain fatty acid--CoA ligase PpsB	869	1051
1052	MGCS36044_02572	Secreted		-	putative secreted protein	868	1052
1053	MGCS36044_04050			*hutD*	histidine uptake and utilization HutD	865	1053
1054	MGCS36044_02964			-	TIGR02206 family membrane protein	860	1054
1055	MGCS36044_02826			-	LCP family anionic cell polymer synthesis enzyme	854	1055
1056	MGCS36044_01730			*fruR*	fructose operon transcriptional repressor protein FruR	851	1056
1057	MGCS36044_03734			-	Cro/CI family transcriptional regulator	847	1057
1058	MGCS36044_04218			-	NUDIX domain-containing protein	845	1058
1059	MGCS36044_03152			-	Gfo/Idh/MocA family oxidoreductase	844	1059
1060	MGCS36044_02322			-	aromatic acid exporter family protein	842	1060
1061	MGCS36044_04074			-	hypothetical protein	839	1061
1062	MGCS36044_04058			-	AAA family ATPase domain-containing DNA-binding transcriptional activator	838	1062
1063	MGCS36044_04110			*cinA*	competence/damage-inducible protein CinA	835	1063
1064	MGCS36044_01466			-	PTS sugar transport IID subunit	834	1064
1065	MGCS36044_01994			*pbuX*	xanthine permease PbuX	833	1065
1066	MGCS36044_03790			*oppD_2*	oligopeptide ABC transporter ATPase OppD	830	1066
1067	MGCS36044_01698			*pyrB*	aspartate transcarbomoylase PyrB	826	1067
1068	MGCS36044_02468			*ykgJ*	YkgJ family cysteine cluster protein	817	1068
1069	MGCS36044_03924			-	hypothetical protein	816	1069
1070	MGCS36044_04164			-	site-specific integrase	816	1069
1071	MGCS36044_00294			*cydD*	thiol reductant ABC exporter subunit CydD	812	1071
1072	MGCS36044_03624			-	IS30 family transposase	810	1072
1073	MGCS36044_02404			-	hypothetical protein	808	1073
1074	MGCS36044_01344			*pepF_1*	oligoendopeptidase PepF	807	1074
1075	MGCS36044_03416			-	TetR/AcrR family transcriptional regulator	807	1074
1076	MGCS36044_02912			-	OFA family MFS transporter	805	1076
1077	MGCS36044_01996			-	thiamine biosynthesis protein ApbE-like	804	1077
1078	MGCS36044_02464			-	maltose/galactose O-acetyltransferase	804	1077

No.	Locus tag	SignalP6 predicted[1]	Virulence[2]	Gene	Function	RPKMs in NHP 15[3]	RANK[4]
1079	MGCS36044_01670			*thiI*	thiamine biosynthesis/tRNA modification protein ThiI	803	1079
1080	MGCS36044_03508			-	DUF956 family protein	803	1079
1081	MGCS36044_04044			*fctD*	glutamate formiminotransferase FctD	803	1079
1082	MGCS36044_00958	Lipo		*fhuD*	iron-hydroxamate ABC transporter substrate-binding lipoprotein FhuD	801	1082
1083	MGCS36044_03628			*scrK*	fructokinase protein ScrK	801	1082
1084	MGCS36044_00034			-	IS30 family transposase	799	1084
1085	MGCS36044_00596			-	hypothetical protein	792	1085
1086	MGCS36044_00990			*holB*	DNA polymerase III subunit delta' HolB	792	1085
1087	MGCS36044_00298			*preA*	polyprenyl synthetase family protein PreA	789	1087
1088	MGCS36044_00778			*uppP*	undecaprenyl pyrophosphate phosphatase UppP	789	1087
1089	MGCS36044_03972			*ulaA*	ascorbate-specific PTS transporter EIIC protein UlaA	787	1089
1090	MGCS36044_00784			*sufC*	Fe-S cluster assembly ATPase SufC	784	1090
1091	MGCS36044_03424			*lacC*	tagatose-6-phosphate kinase LacC	783	1091
1092	MGCS36044_03996			*ifs*	nicotine adenine dinucleotide glycohydrolase inhibitor Ifs	781	1092
1093	MGCS36044_02138			-	hypothetical protein	779	1093
1094	MGCS36044_00826			*yqeH*	ribosome biogenesis GTPase YqeH	776	1094
1095	MGCS36044_03630			*scrA*	sucrose-specific PTS fused IIB/IIC/IIA component ScrA	775	1095
1096	MGCS36044_01630			-	LTA synthase family protein	774	1096
1097	MGCS36044_01742			-	DUF1149 domain-containing protein	772	1097
1098	MGCS36044_00916			*rdgB*	RdgB family non-canonical purine NTP pyrophosphatase	768	1098
1099	MGCS36044_02018			-	MdlB superfamily multidrug ABC transporter ATP-binding protein	768	1098
1100	MGCS36044_02540			-	DUF4097 family beta strand repeat-containing protein	768	1098
1101	MGCS36044_02454			*uvrC*	excinuclease ABC subunit UvrC	767	1101
1102	MGCS36044_03008			*tlyA*	TlyA family RNA methyltransferase	765	1102
1103	MGCS36044_02932			-	DUF3114 domain-containing protein	758	1103
1104	MGCS36044_03006			*argR_1*	ArgR family transcriptional regulator	757	1104
1105	MGCS36044_03590			*asnB*	L-asparaginase AsnB	757	1104
1106	MGCS36044_00850	Lipo		-	amino acid ABC transporter substrate-binding lipoprotein	755	1106
1107	MGCS36044_01460			*bga*	beta-galactosidase Bga	755	1106
1108	MGCS36044_00534	Secreted	Virulence	*pulA_1*	cell surface pullulanase PulA	750	1108
1109	MGCS36044_02712			*rsuA_1*	ribosomal small subunit pseudouridine synthase RsuA	749	1109

No.	Locus tag	SignalP6 predicted[1]	Virulence[2]	Gene	Function	RPKMs in NHP 15[3]	RANK[4]
1110	MGCS36044_01912	Secreted		*dacA_3*	secreted D,D-carboxypeptidase penicillin-binding protein DacA	748	1110
1111	MGCS36044_00774	Secreted		-	ABC amino acid transporter substrate-binding secreted protein	742	1111
1112	MGCS36044_02088			-	site-specific integrase	742	1111
1113	MGCS36044_00010			*engD*	redox-regulated ATPase EngD	735	1113
1114	MGCS36044_02134			*ylxM*	YlxM superfamily signal recognition particle associated DNA-binding protein	735	1113
1115	MGCS36044_02320			-	DUF2130 domain-containing protein	734	1115
1116	MGCS36044_00862			*brnQ_1*	branched-chain amino acid transport system II carrier protein BrnQ	733	1116
1117	MGCS36044_00920			-	CBS domain-containing protein	732	1117
1118	MGCS36044_00112			*acpP_1*	acyl carrier protein AcpP	730	1118
1119	MGCS36044_01964			-	DUF1831 domain-containing protein	729	1119
1120	MGCS36044_03088			*rlmN*	23S rRNA (adenine(2503)-C(2))-methyltransferase RlmN	728	1120
1121	MGCS36044_03696			-	PurR family transcriptional regulator	723	1121
1122	MGCS36044_02652			*malF*	maltose/maltodextrin ABC transport system permease protein MalF	721	1122
1123	MGCS36044_02620			*rnz*	ribonuclease Rnz	717	1123
1124	MGCS36044_04234			*cbiQ_2*	cobalt ABC transporter permease CbiQ	717	1123
1125	MGCS36044_03746			-	type I restriction-modification system (M) methylase subunit	716	1125
1126	MGCS36044_03800			*glnR*	glutamine synthetase transcriptional repressor GlnR	713	1126
1127	MGCS36044_01654			*rlmK*	23S rRNA methyltransferase RmlK	707	1127
1128	MGCS36044_04134			-	YitT family protein putative ABC transporter ATPase	707	1127
1129	MGCS36044_03796	Lipo		*oppA_2*	oligopeptide ABC transporter substrate-binding lipoprotein OppA	705	1129
1130	MGCS36044_00360			*hslO*	Hsp33 family molecular chaperone HslO	704	1130
1131	MGCS36044_02824			-	23S rRNA (uracil(1939)-C(5))-methyltransferase RlmD	692	1131
1132	MGCS36044_01762			*sptR*	SptR-like TCS DNA-binding response regulator	689	1132
1133	MGCS36044_02638			-	hypothetical protein	689	1132
1134	MGCS36044_01736			-	putative peptidoglycan hydrolase	687	1134
1135	MGCS36044_01892			*cadA*	cadmium-translocating P-type ATPase CadA	683	1135
1136	MGCS36044_03832			*rsmA*	16S rRNA (adenine(1518)-N(6)/adenine(1519)-N(6))- dimethyltransferase RsmA	680	1136
1137	MGCS36044_02490			-	sakacin P family class II bacteriocin	678	1137
1138	MGCS36044_02486			-	DUF2974 domain-containing protein	677	1138
1139	MGCS36044_03614	Secreted	Virulence	*shr*	heme-binding secreted protein Shr	677	1138
1140	MGCS36044_04116			*ruvA*	Holliday junction ATP-dependent DNA helicase RuvA	676	1140

No.	Locus tag	SignalP6 predicted(1)	Virulence(2)	Gene	Function	RPKMs in NHP 15(3)	RANK(4)
1141	MGCS36044_01920			*folC*	dihydrofolate synthase FolC	674	1141
1142	MGCS36044_03298		Virulence	*liaF*	three component system signal transduction membrane component protein LiaF	672	1142
1143	MGCS36044_01478			*gstA*	GstA superfamily glutathione-dependent disulfide-bond oxidoreductase	670	1143
1144	MGCS36044_02130			-	hypothetical protein	669	1144
1145	MGCS36044_00728			*thiD*	bifunctional hydroxymethylpyrimidine kinase/phosphomethylpyrimidine kinase PdxK	665	1145
1146	MGCS36044_01958			-	gamma-glutamyl-gamma-aminobutyrate hydrolase family protein	665	1145
1147	MGCS36044_02382			-	TVP38/TMEM64 family protein	665	1145
1148	MGCS36044_02802			*ecfA2*	EcfA2 family ECF transporter ATPase	664	1148
1149	MGCS36044_01612			*rgpB*	glycosyltransferase family GT2 protein RgpB	661	1149
1150	MGCS36044_03030			-	amino acid ABC transporter ATP-binding protein	661	1149
1151	MGCS36044_03728			-	ABC transporter permease	654	1151
1152	MGCS36044_02854			*fadR*	FadR family DNA-binding transcriptional regulator	652	1152
1153	MGCS36044_00428			-	FAD/NAD(P)-binding protein	650	1153
1154	MGCS36044_02878			*udk*	uridine kinase Udk	650	1153
1155	MGCS36044_01324			-	PhoE superfamily phosphatase	648	1155
1156	MGCS36044_03282			*comFC*	ComFC family predicted amidophosphoribosyltransferase	648	1155
1157	MGCS36044_03698			-	beta-lactamase family protein	645	1157
1158	MGCS36044_02416	Lipo		-	amino-acid ABC transporter substrate-binding lipoprotein	643	1158
1159	MGCS36044_01204			-	ABC transporter ATP-binding component	640	1159
1160	MGCS36044_01570			*epuA*	DNA-directed RNA polymerase beta subunit EpuA	640	1159
1161	MGCS36044_00746			*lytS*	TCS sensor histidine kinase LytS	639	1161
1162	MGCS36044_00834			-	cysteine hydrolase	639	1161
1163	MGCS36044_03012			*xseB*	exodeoxyribonuclease VII small subunit XseB	635	1163
1164	MGCS36044_00026			*tilS*	tRNA lysidine(34) synthetase TilS	633	1164
1165	MGCS36044_01924			*folP*	dihydropteroate synthase protein FolP	633	1164
1166	MGCS36044_03896			-	MarR family transcriptional regulator	629	1166
1167	MGCS36044_01768			*mvaD*	diphosphomevalonate decarboxylase MvaD	625	1167
1168	MGCS36044_02624			*hflX*	GTP-binding protein HflX	622	1168
1169	MGCS36044_01400			*agaF*	PTS transporter hyaluronate-oligosaccharide-specific IIA component AgaF	621	1169
1170	MGCS36044_01594	Lipo		-	ABC transporter substrate-binding lipoprotein	618	1170

No.	Locus tag	SignalP6 predicted(1)	Virulence(2)	Gene	Function	RPKMs in NHP 15(3)	RANK(4)
1171	MGCS36044_02516			-	tracrRNA RNA	618	1170
1172	MGCS36044_02680	Lipo		*glnP_2*	glutamine-binding protein/glutamine transport system permease GlnP	612	1172
1173	MGCS36044_03558			-	drug/metabolite transporter superfamily protein	611	1173
1174	MGCS36044_01224			*paaJ*	acetyl-CoA acetyl transferase PaaJ	610	1174
1175	MGCS36044_03870		Virulence	*fasC*	signal transduction sensor histidine kinase protein FasC	608	1175
1176	MGCS36044_02488			*spiA*	sakacin P immunity protein SpiA	605	1176
1177	MGCS36044_04222			*marC*	MarC family small neutral amino acid transporter	602	1177
1178	MGCS36044_00786			*sufD*	Fe-S cluster assembly protein SufD	600	1178
1179	MGCS36044_04206			-	Veg family protein	599	1179
1180	MGCS36044_03232			*mngB*	alpha-mannosidase MngB	595	1180
1181	MGCS36044_04250			*recF*	DNA replication/repair protein RecF	595	1180
1182	MGCS36044_00894			*murC*	UDP-N-acetylmuramate--L-alanine ligase MurC	593	1182
1183	MGCS36044_01398			*ugl*	unsaturated chondroitin disaccharide hydrolase Ugl	592	1183
1184	MGCS36044_00356			-	deoxyadenosine kinase	591	1184
1185	MGCS36044_03512			*serS*	seryl-tRNA synthetase SerS	591	1184
1186	MGCS36044_01202			*thrS*	threonyl-tRNA synthetase ThrS	587	1186
1187	MGCS36044_02090			-	DUF3173 family protein	587	1186
1188	MGCS36044_02748			-	MATE family efflux transporter	587	1186
1189	MGCS36044_01054			*glmU*	bifunctional UDP-N-acetylglucosamine diphosphorylase/glucosamine-1-phosphate N-acetyltransferase GlmU	585	1189
1190	MGCS36044_03642			*comEB*	competence protein ComEB	581	1190
1191	MGCS36044_02060			-	SSRC38 RNA	580	1191
1192	MGCS36044_02324			*hsdS*	type I restriction endonuclease subunit S	578	1192
1193	MGCS36044_01338			-	ECF transporter S component	571	1193
1194	MGCS36044_02890			*nrdH*	glutaredoxin-like protein NrdH	569	1194
1195	MGCS36044_04030			-	metallo-dependent amidohydrolase	567	1195
1196	MGCS36044_01422			*fbp2*	fructose-bisphosphatase Fbp2	566	1196
1197	MGCS36044_02446			*mnmE*	MnmE family tRNA uridine-5-carboxymethylaminomethyl(34) synthesis GTPase	566	1196
1198	MGCS36044_01458			-	GntR family transcriptional regulator	561	1198
1199	MGCS36044_04224			*mnmA*	tRNA 2-thiouridine(34) synthase MnmA	561	1198
1200	MGCS36044_02022			-	FolA superfamily dihydrofolate reductase	559	1200

No.	Locus tag	SignalP6 predicted(1)	Virulence(2)	Gene	Function	RPKMs in NHP 15(3)	RANK(4)
1201	MGCS36044_00602			-	L13_leader RNA	557	1201
1202	MGCS36044_03782			*tsaB*	tRNA (adenosine(37)-N6)-threonylcarbamoyltransferase complex dimerization subunit type 1 TsaB	556	1202
1203	MGCS36044_00964			*murJ*	peptidoglycan lipid-II intermediate flippase MurJ	553	1203
1204	MGCS36044_01838			-	type IV toxin-antitoxin system AbiEi family antitoxin	553	1203
1205	MGCS36044_03064			*murD*	UDP-N-acetylmuramoyl-L-alanine--D-glutamate ligase MurD	553	1203
1206	MGCS36044_02642			*glgP*	maltodextrin phosphorylase protein GlgP	551	1206
1207	MGCS36044_03692			-	carbohydrate ABC transporter permease UgpE-like	551	1206
1208	MGCS36044_01454			*agaS*	AgaS superfamily sugar isomerase SIS domain-containing protein	550	1208
1209	MGCS36044_00900			*greA*	transcription elongation factor GreA	544	1209
1210	MGCS36044_02252			-	NAD-dependent deacetylase	544	1209
1211	MGCS36044_03730			-	ABC transporter ATP-binding protein	544	1209
1212	MGCS36044_03676			*recD*	ATP-dependent DNA helicase RecD	543	1212
1213	MGCS36044_00748			*lytR*	TCS DNA-binding response regulator LytR	542	1213
1214	MGCS36044_01672			*capA*	CapA family protein	541	1214
1215	MGCS36044_03904			*glpG*	GlpG family membrane associated serine protease	538	1215
1216	MGCS36044_02622			-	hypothetical protein	537	1216
1217	MGCS36044_03172			-	AAA family ATPase	536	1217
1218	MGCS36044_01588			*rexB*	ATP-dependent nuclease B subunit RexB	535	1218
1219	MGCS36044_04122			*mutS*	DNA mismatch repair protein MutS	534	1219
1220	MGCS36044_01448			*pepD_1*	C69 family dipeptidase PepD	533	1220
1221	MGCS36044_03288			*cysK*	cysteine synthase A CysK	533	1220
1222	MGCS36044_01172			-	multidrug efflux MFS transporter	532	1222
1223	MGCS36044_03198			-	hypothetical protein	531	1223
1224	MGCS36044_00296			*cdyC*	thiol reductant ABC exporter subunit CydC	530	1224
1225	MGCS36044_01496			-	HAD-IA family hydrolase	530	1224
1226	MGCS36044_03244			*thlA*	thiolase ThlA	530	1224
1227	MGCS36044_03778			*tsaD*	tRNA (adenosine(37)-N6)-threonylcarbamoyltransferase complex transferase subunit TsaD	530	1224
1228	MGCS36044_00776	Secreted		-	DUF2207 domain-containing secreted protein	527	1228
1229	MGCS36044_02926			*rsuA_2*	ribosomal small subunit pseudouridine synthase RsuA	523	1229
1230	MGCS36044_00228			-	IS30 family transposase	520	1230

No.	Locus tag	SignalP6 predicted[1]	Virulence[2]	Gene	Function	RPKMs in NHP 15[3]	RANK[4]
1231	MGCS36044_01782			*mvaS1*	hydroxymethylglutaryl-CoA reductase protein (1) MvaS1	520	1230
1232	MGCS36044_01998			*pptA*	phenylpyruvate tautomerase PptA	520	1230
1233	MGCS36044_02408			*plsY*	glycerol-3-phosphate 1-O-acyltransferase PlsY	520	1230
1234	MGCS36044_01230			*glnP_1*	glutamine ABC transporter permease GlnP	518	1234
1235	MGCS36044_01406			*kdgK*	2-dehydro-3-deoxygluconokinase KdgK	518	1234
1236	MGCS36044_00896			-	GNAT family N-acetyltransferase	514	1236
1237	MGCS36044_02604			*zupT*	ZIP family metal transporter ZupT	514	1236
1238	MGCS36044_02590			*mutX*	8-oxo-dGTP diphosphatase MutX	512	1238
1239	MGCS36044_03482			*ecsA*	ABC exoprotein transporter ATPase EcsA	512	1238
1240	MGCS36044_03178			-	helix-hairpin-helix domain-containing protein	509	1240
1241	MGCS36044_03026			*feoB*	ferrous iron transport protein (B) FeoB	507	1241
1242	MGCS36044_02606			-	Nif3-like dinuclear metal center hexameric protein	500	1242
1243	MGCS36044_02740			-	resolvase/recombinase family protein	500	1242
1244	MGCS36044_03718			-	OmpR family TCS DNA-binding response regulator	493	1244
1245	MGCS36044_02844			-	phosphoglycolate phosphatase	492	1245
1246	MGCS36044_03222			-	hypothetical protein	491	1246
1247	MGCS36044_02562			*phnK*	PhnK family ABC transporter ATPase component	488	1247
1248	MGCS36044_00402			*celA_1*	PTS transporter cellobiose-specific IIB component CelA	484	1248
1249	MGCS36044_02862			*murA_2*	UDP-N-acetylglucosamine 1-carboxyvinyltransferase MurA	484	1248
1250	MGCS36044_00002			*dnaA*	chromosomal replication initiator protein DnaA	483	1250
1251	MGCS36044_01442			*rapZ*	RNase adapter RapZ	483	1250
1252	MGCS36044_01412			-	hypothetical protein	481	1252
1253	MGCS36044_01754			*gdhA*	NADP-specific glutamate dehydrogenase GdhA	479	1253
1254	MGCS36044_01572			*endA*	DNA-entry competence-associated nuclease EndA	478	1254
1255	MGCS36044_04024			*ctsR*	CtsR family transcriptional regulator	478	1254
1256	MGCS36044_03042			-	hypothetical protein	477	1256
1257	MGCS36044_02422			-	GNAT family N-acetyltransferase	469	1257
1258	MGCS36044_01396			*agaV*	PTS transporter hyaluronate-oligosaccharide-specific IIB component AgaV	466	1258
1259	MGCS36044_03842			*nanK*	N-acetylmannosamine kinase NanK	466	1258
1260	MGCS36044_01290			*phoU_1*	phosphate signaling complex protein PhoU	462	1260
1261	MGCS36044_02640			-	NADPH-dependent FMN reductase	458	1261

No.	Locus tag	SignalP6 predicted(1)	Virulence(2)	Gene	Function	RPKMs in NHP 15(3)	RANK(4)
1262	MGCS36044_01378			-	HD domain-containing phosphohydrolase	457	1262
1263	MGCS36044_03868		Virulence	*fasA*	signal transduction response regulator protein FasA	457	1262
1264	MGCS36044_01452			-	GntR family transcriptional regulator	455	1264
1265	MGCS36044_00406			*pflD*	pyruvate formate-lyase protein PflD	454	1265
1266	MGCS36044_03094			-	SDR family oxidoreductase	449	1266
1267	MGCS36044_00512			*nrdI_1*	class Ib ribonucleoside-diphosphate reductase assembly flavoprotein NrdI	444	1267
1268	MGCS36044_01988			*guaC*	guanosine 5'-monophosphate oxidoreductase GuaC	441	1268
1269	MGCS36044_02308			*rsmF*	RsmF family rRNA cytosine-C5-methyltransferase	441	1268
1270	MGCS36044_02386			-	DUF3862 domain-containing lipoprotein	441	1268
1271	MGCS36044_02398			-	DUF2969 domain-containing protein	441	1268
1272	MGCS36044_02944	Secreted		*plsC*	secreted 1-acyl-sn-glycerol-3-phosphate acyltransferase PlsC	440	1272
1273	MGCS36044_02786			-	ORF6N domain-containing protein	438	1273
1274	MGCS36044_00788			*sufS*	cysteine desulfurase SufS	436	1274
1275	MGCS36044_00452			-	MdlB family ABC transporter ATP-binding/permease protein	435	1275
1276	MGCS36044_01312			-	PrtC family collagenase-like protease	435	1275
1277	MGCS36044_01922			*folE*	GTP cyclohydrolase I protein FolE	434	1277
1278	MGCS36044_01992			*xpt*	xanthine phosphoribosyltransferase Xpt	432	1278
1279	MGCS36044_00134			*purK*	5-(carboxyamino)imidazole ribonucleotide synthase PurK	431	1279
1280	MGCS36044_01322			-	HAD family hydrolase	431	1279
1281	MGCS36044_01308			-	DUF3270 domain-containing protein	428	1281
1282	MGCS36044_00960			*fhuA*	ferrichrome ABC transporter ATP-binding protein FhuA	426	1282
1283	MGCS36044_01346			*ppc*	phosphoenolpyruvate carboxylase Ppc	426	1282
1284	MGCS36044_00440			-	hypothetical protein	423	1284
1285	MGCS36044_00860			*metP_1*	methionine ABC transporter permease MetP	423	1284
1286	MGCS36044_02058	Secreted	Virulence	*scpA*	cell surface extracellular C5a peptidase ScpA	422	1286
1287	MGCS36044_02466			*ycjU*	YcjU family beta-phosphoglucomutase or related phosphatase	416	1287
1288	MGCS36044_02860			*melB*	MelB family MFS transporter	416	1287
1289	MGCS36044_00944			-	RsmB/NOP family class I SAM-dependent RNA methyltransferase	413	1289
1290	MGCS36044_01370			-	FMN-binding domain containing L-lactate oxidase	411	1290
1291	MGCS36044_03456			-	YSIRK-targeted surface antigen transcriptional regulator	410	1291
1292	MGCS36044_03322			*atoA*	acyl CoA:acetate/3-ketoacid CoA transferase beta subunit protein AtoA	409	1292

No.	Locus tag	SignalP6 predicted(1)	Virulence(2)	Gene	Function	RPKMs in NHP 15(3)	RANK(4)
1293	MGCS36044_03982	Secreted		-	putative secreted protein	406	1293
1294	MGCS36044_02630			-	C4-dicarboxylate ABC transporter	404	1294
1295	MGCS36044_01918			*thrB*	ThrB family homoserine kinase	403	1295
1296	MGCS36044_01016			*sdpI*	SdpI family immunity protein	402	1296
1297	MGCS36044_03788			*oppF_2*	oligopeptide ABC transporter ATPase OppF	402	1296
1298	MGCS36044_00384			-	PTS sugar transporter subunit IIC	401	1298
1299	MGCS36044_01746			-	tRNA CCA-pyrophosphorylase	396	1299
1300	MGCS36044_02876			-	DUF1294 domain-containing protein	396	1299
1301	MGCS36044_00404			*celB_1*	PTS transporter cellobiose-specific IIC component protein CelB	392	1301
1302	MGCS36044_01388			*regR*	transcriptional regulator RegR	391	1302
1303	MGCS36044_01208			-	ABC transporter permease component	390	1303
1304	MGCS36044_01456			*lacD_1*	tagatose-bisphosphate aldolase LacD-like	389	1304
1305	MGCS36044_01740			-	hypothetical protein	388	1305
1306	MGCS36044_00380			-	PAS domain-containing protein	385	1306
1307	MGCS36044_03564			*gatC_2*	aspartyl-tRNA(Asn) or glutamyl-tRNA(Gln) amidotransferase C subunit GatC	385	1306
1308	MGCS36044_03650			-	DUF1129 domain-containing protein	384	1308
1309	MGCS36044_00954			*fhuG*	iron ABC transporter permease FhuG	383	1309
1310	MGCS36044_03854	Lipo		*ugpB_2*	carbohydrate ABC transporter substrate-binding lipoprotein UgpB-like	382	1310
1311	MGCS36044_02428			-	PyrR RNA	381	1311
1312	MGCS36044_01372			*murN*	peptidoglycan lipid II-Ala--L-alanine ligase protein MurN	378	1312
1313	MGCS36044_01870			-	XRE family HTH-type transcriptional regulator	378	1312
1314	MGCS36044_02148			-	TCS histidine kinase sensor	378	1312
1315	MGCS36044_02952			*deaD*	DEAD/DEAH box helicase	378	1312
1316	MGCS36044_03248			*trmA*	TrmA RNA methyltransferase	378	1312
1317	MGCS36044_03850			*ugpE_2*	carbohydrate ABC transporter permease UgpE-like	376	1317
1318	MGCS36044_00832			*yqeK*	bis(5'-nucleosyl)-tetraphosphatase (symmetrical) YqeK	375	1318
1319	MGCS36044_03422			*lacD_2*	tagatose-bisphosphate aldolase LacD	375	1318
1320	MGCS36044_02970			*recR*	recombination mediator RecR	374	1320
1321	MGCS36044_00858			*metN_1*	methionine ABC transporter ATP-binding protein MetN	372	1321
1322	MGCS36044_01334			-	GH25 muramidase superfamily lysozyme	370	1322
1323	MGCS36044_01042			-	DUF2829 domain-containing protein	367	1323

No.	Locus tag	SignalP6 predicted(1)	Virulence(2)	Gene	Function	RPKMs in NHP 15(3)	RANK(4)
1324	MGCS36044_01916			*hom*	homoserine dehydrogenase Hom	367	1323
1325	MGCS36044_04076			*yybR*	putative HTH-type transcriptional regulator YybR	367	1323
1326	MGCS36044_00988			*tmk*	thymidylate kinase Tmk	365	1326
1327	MGCS36044_03798			*glnA*	glutamine synthetase GlnA	363	1327
1328	MGCS36044_04186			-	thioredoxin family protein	363	1327
1329	MGCS36044_01896			-	DUF4365 family protein	361	1329
1330	MGCS36044_03716			-	BaeS family TCS sensor histidine kinase	361	1329
1331	MGCS36044_03702			-	putative sulfite exporter	360	1331
1332	MGCS36044_03836			*tatD*	Tat protein secretion system quality control protein TatD	360	1331
1333	MGCS36044_04212			-	DHH family phosphoesterase	360	1331
1334	MGCS36044_03210			*trxT*	Trx TCS operon protein TrxT	359	1334
1335	MGCS36044_01118			-	hypothetical protein	356	1335
1336	MGCS36044_02346			*pcrA*	DNA helicase PcrA	354	1336
1337	MGCS36044_02406			*parE*	DNA topoisomerase IV subunit B ParE	354	1336
1338	MGCS36044_02868			*birA*	bifunctional biotin--[acetyl-CoA-carboxylase] ligase/biotin operon repressor BirA	354	1336
1339	MGCS36044_03082			-	MdlB family multidrug ABC transporter ATP-binding protein	353	1339
1340	MGCS36044_03658			-	hypothetical protein	351	1340
1341	MGCS36044_02616			*recJ*	single-stranded-DNA-specific exonuclease RecJ	349	1341
1342	MGCS36044_02820			-	DinB family protein	348	1342
1343	MGCS36044_02842			-	BglX family glycosyl hydrolase	348	1342
1344	MGCS36044_02906			-	LURP-one-related family protein	348	1342
1345	MGCS36044_03978			-	IS30 family transposase	347	1345
1346	MGCS36044_02874			*prsW*	PrsW family glutamic-type intramembrane protease	346	1346
1347	MGCS36044_03660			*mutY*	A/G-specific adenine glycosylase MutY	345	1347
1348	MGCS36044_00738			-	alpha-amylase family glycosyl hydrolase	342	1348
1349	MGCS36044_01288			*pstB*	phosphate ABC transporter ATP-binding protein PstB	341	1349
1350	MGCS36044_00130			*purD*	phosphoribosylamine--glycine ligase PurD	340	1350
1351	MGCS36044_02648			-	IS30 family transposase	339	1351
1352	MGCS36044_01666			-	hypothetical protein	337	1352
1353	MGCS36044_00730			-	ECF transporter S component	336	1353
1354	MGCS36044_04256			*trpS*	tryptophanyl-tRNA synthetase	335	1354

No.	Locus tag	SignalP6 predicted[1]	Virulence[2]	Gene	Function	RPKMs in NHP 15[3]	RANK[4]
1355	MGCS36044_00566			-	PTS transporter IIA component UlaC-like	332	1355
1356	MGCS36044_01420			-	HAD family phosphatase	332	1355
1357	MGCS36044_02328			-	ABC transporter ATP-binding protein LolD-like	332	1355
1358	MGCS36044_01434			*dinG*	bifunctional DnaQ family exonuclease -- ATP-dependent helicase DinG	330	1358
1359	MGCS36044_01656			*aroD*	type I 3-dehydroquinate dehydratase AroD	329	1359
1360	MGCS36044_03680			*dinB*	DNA polymerase IV DinB	329	1359
1361	MGCS36044_01284			*pstC*	phosphate ABC transporter, permease protein PstC	328	1361
1362	MGCS36044_00970			-	DUF4298 domain-containing protein	325	1362
1363	MGCS36044_02972			*pbp2B*	penicillin-binding protein PBP2B/FtsI	323	1363
1364	MGCS36044_03024			-	FeoB associated cysteine-rich protein	322	1364
1365	MGCS36044_00934			*trmI*	TrmL family tRNA (cytosine34-2'-O-)-methyl transferase	321	1365
1366	MGCS36044_01408			*kgdA*	bifunctional 4-hydroxy-2-oxoglutarate (KHG) aldolase/2-dehydro-3-deoxy-phosphogluconate (KDPG) aldolase KdgA	318	1366
1367	MGCS36044_02714			-	putative thioesterase	318	1366
1368	MGCS36044_03634			*scrR*	sucrose operon repressor ScrR	317	1368
1369	MGCS36044_03032			-	amino acid ABC transporter permease	316	1369
1370	MGCS36044_00160			*thrC*	threonine synthase ThrC	313	1370
1371	MGCS36044_02602			*dadA*	FAD-binding oxidoreductase DadA	313	1370
1372	MGCS36044_04126			*argR_3*	arginine repressor ArgR	313	1370
1373	MGCS36044_00924			*scp1*	segregation/condensation complex subunit (A) ScpA/Scp1	312	1373
1374	MGCS36044_02204			*rnhB*	HII ribonuclease RnhB	310	1374
1375	MGCS36044_02462			-	DUF1524 domain-containing protein	310	1374
1376	MGCS36044_01974			*nadK*	NAD kinase NadK	309	1376
1377	MGCS36044_02254			-	protein-ADP-ribose hydrolase	309	1376
1378	MGCS36044_02958			*metN_2*	methionine uptake ABC transporter ATP-binding protein MetN	309	1376
1379	MGCS36044_02374			*dnaE*	DNA polymerase III subunit alpha DnaE	307	1379
1380	MGCS36044_01318			-	ammonium transporter	306	1380
1381	MGCS36044_02856			-	beta-D-glucuronidase	306	1380
1382	MGCS36044_00378		Virulence	*fbp*	secreted fibronectin-binding protein	305	1382
1383	MGCS36044_02206			*ylqF*	ribosome biogenesis GTPase YlqF	305	1382
1384	MGCS36044_01286			*pstA*	phosphate ABC transporter, permease protein PstA	304	1384
1385	MGCS36044_02456			-	HAD hydrolase family protein	303	1385

No.	Locus tag	SignalP6 predicted[1]	Virulence[2]	Gene	Function	RPKMs in NHP 15[3]	RANK[4]
1386	MGCS36044_03434			-	PTS sugar transporter subunit IIA	300	1386
1387	MGCS36044_01696			*pyrP*	uracil permease protein PyrP	299	1387
1388	MGCS36044_02750			-	PEP-utilizing enzyme	298	1388
1389	MGCS36044_03202			*aroE*	shikimate dehydrogenase AroE	298	1388
1390	MGCS36044_00842			-	MerR family transcriptional regulator	296	1390
1391	MGCS36044_01156			-	hypothetical protein	296	1390
1392	MGCS36044_02828			*aroK*	shikimate kinase AroK	296	1390
1393	MGCS36044_02644			*malQ*	4-alpha-glucanotransferase (amylomaltase) protein MalQ	295	1393
1394	MGCS36044_03912			-	Mga-related helix-turn-helix domain-containing transcriptional regulator	295	1393
1395	MGCS36044_01868			-	hypothetical protein	294	1395
1396	MGCS36044_00288			*nox*	NAD(P)/FAD-dependent oxidoreductase Nox	293	1396
1397	MGCS36044_03410			*nptA*	NtpA family Na/Pi cotransporter	293	1396
1398	MGCS36044_02160	Lipo		*apbE*	ApbE family thiamine biosynthesis lipoprotein	290	1398
1399	MGCS36044_03852			*ugpA_2*	carbohydrate ABC transporter permease UgpA-like	289	1399
1400	MGCS36044_00672	Pilin		-	putative lipoprotein	288	1400
1401	MGCS36044_03498			-	SSRC34_2 RNA	288	1400
1402	MGCS36044_03090			*yutD*	YutD family protein of unknown function	287	1402
1403	MGCS36044_03326			*gntT*	GntT family H+/gluconate symporter or related permease	287	1402
1404	MGCS36044_03772			*yhiN*	YhiN family predicted flavoprotein	287	1402
1405	MGCS36044_03644			*pepP*	PepP family Xaa-Pro peptidase	285	1405
1406	MGCS36044_03856			*nanE*	N-acetylmannosamine-6-phosphate 2-epimerase NanE	282	1406
1407	MGCS36044_01276			-	cupin domain-containing protein	280	1407
1408	MGCS36044_02924			-	MFS transporter	278	1408
1409	MGCS36044_03086			-	VanZ like family glycopeptide antibiotic resistance protein	274	1409
1410	MGCS36044_01722			*trmD*	tRNA (guanosine(37)-N1)-methyltransferase TrmD	272	1410
1411	MGCS36044_02256			*gcvH*	glycine cleavage system protein H GcvH	271	1411
1412	MGCS36044_01726			-	PTS transporter subunit IIC	270	1412
1413	MGCS36044_02914			-	HAD family hydrolase	269	1413
1414	MGCS36044_00906			-	SpoU family RNA methyltransferase	267	1414
1415	MGCS36044_02710			-	CorA family divalent cation transport protein	267	1414
1416	MGCS36044_01010			-	PhnB family glyoxalase/bleomycin resistance/extradiol dioxygenase family protein	265	1416

No.	Locus tag	SignalP6 predicted(1)	Virulence(2)	Gene	Function	RPKMs in NHP 15(3)	RANK(4)
1417	MGCS36044_03240			*caiC*	CaiC family Acyl-CoA synthetase (AMP-forming)/AMP-acid ligase II	265	1416
1418	MGCS36044_03886			*radA*	DNA repair protein RadA	265	1416
1419	MGCS36044_04262			*yfhO*	YfhO family protein	265	1416
1420	MGCS36044_03228			-	PurR/LacI family transcriptional regulator	263	1420
1421	MGCS36044_00938			-	ECF transporter S component	261	1421
1422	MGCS36044_00398			-	DeoR-like transcriptional regulator protein	260	1422
1423	MGCS36044_00138			-	hypothetical protein	259	1423
1424	MGCS36044_02352			*fieF*	FieF family cation diffusion facilitator family transporter	258	1424
1425	MGCS36044_03926	Secreted	Virulence	*speG*	streptococcal pyrogenic exotoxin (G) SpeG	258	1424
1426	MGCS36044_01626			-	DUF2142 domain-containing protein	255	1426
1427	MGCS36044_01784			*mvaS2*	hydroxymethylglutaryl-CoA synthase protein (2) MvaS2	254	1427
1428	MGCS36044_00396			-	GlpR-like transcriptional regulator protein	252	1428
1429	MGCS36044_03404			*ypbQ*	YpbQ family isoprenylcysteine carboxyl methyltransferase family protein	252	1428
1430	MGCS36044_00926			*scp2*	segregation/condensation complex subunit (B) ScpB/Scp2	251	1430
1431	MGCS36044_00284			-	NUDIX domain-containing protein	248	1431
1432	MGCS36044_01486			*pheA*	chorismate mutase PheA	246	1432
1433	MGCS36044_01930			*murB*	UDP-N-acetylmuramate dehydrogenase MurB	246	1432
1434	MGCS36044_00388			-	hypothetical protein	245	1434
1435	MGCS36044_00838			-	class I SAM-dependent methyltransferase	245	1434
1436	MGCS36044_01444			*cofD*	CofD/YvcK superfamily 2-phospho-L-lactate transferase	244	1436
1437	MGCS36044_02520			*nagD*	NagD family hydrolase	244	1436
1438	MGCS36044_02724			*paaI*	PaaI family thioesterase	244	1436
1439	MGCS36044_02726			-	nucleoside phosphorylase family protein	244	1436
1440	MGCS36044_00136			-	DUF4041 domain-containing protein	242	1440
1441	MGCS36044_01392			*agaD*	PTS transporter hyaluronate-oligosaccharide-specific IID component AgaD	241	1441
1442	MGCS36044_03412			-	hypothetical protein	241	1441
1443	MGCS36044_02522			*fatA*	FatA family acyl-[acyl-carrier-protein] thioesterase	240	1443
1444	MGCS36044_00418			-	MccC family LD-carboxypeptidase	239	1444
1445	MGCS36044_01446			*whiA*	cell division involved DNA-binding protein WhiA	239	1444
1446	MGCS36044_02388			-	IS1182 family transposase	239	1444
1447	MGCS36044_00444			*flaR*	DNA topology modulation protein	238	1447

No.	Locus tag	SignalP6 predicted[1]	Virulence[2]	Gene	Function	RPKMs in NHP 15[3]	RANK[4]
1448	MGCS36044_03436			*glpR*	GlpR family DNA-binding transcriptional regulator of sugar metabolism	238	1447
1449	MGCS36044_00840			-	nucleotidyltransferase	235	1449
1450	MGCS36044_01076			-	DUF3397 domain-containing protein	235	1449
1451	MGCS36044_02310			-	inositol monophosphatase family protein	235	1449
1452	MGCS36044_02858			-	sugar kinase	235	1449
1453	MGCS36044_02258			-	NADH-dependent flavin oxidoreductase	230	1453
1454	MGCS36044_02976			*niaX*	niacin transporter NiaX	228	1454
1455	MGCS36044_02918			-	NAD(P)H-dependent oxidoreductase	227	1455
1456	MGCS36044_03154			*cas2_2*	CRISPR-associated endonuclease Cas2	227	1455
1457	MGCS36044_02312			-	UPF0223 family protein	225	1457
1458	MGCS36044_00936			-	FMN RNA	224	1458
1459	MGCS36044_02618			*dltE*	short-chain dehydrogenase DltE	224	1458
1460	MGCS36044_00412			-	hypothetical protein	222	1460
1461	MGCS36044_01266			*bglA*	6-phospho-beta-glucosidase BglA	220	1461
1462	MGCS36044_00790			*sufE*	SUF system NifU family Fe-S cluster assembly protein SufE	218	1462
1463	MGCS36044_03028			*feoA*	ferrous iron transport protein (A) FeoA	217	1463
1464	MGCS36044_00386			-	toxic anion resistance protein, tellurite resistance protein	216	1464
1465	MGCS36044_02910			*yrrM*	YrrM family O-methyltransferase	216	1464
1466	MGCS36044_00334			-	class I SAM-dependent methyltransferase	215	1466
1467	MGCS36044_01894			-	integrase catalytic subunit	215	1466
1468	MGCS36044_04216			*mnmG*	tRNA uridine-5-carboxymethylaminomethyl(34) synthesis enzyme MnmG	213	1468
1469	MGCS36044_03320			*atoD*	butyrate-acetoacetate CoA-transferase alpha subunit protein AtoD	210	1469
1470	MGCS36044_00008			-	helix-turn-helix domain-containing protein	209	1470
1471	MGCS36044_02210			-	DUF1836 domain-containing protein	209	1470
1472	MGCS36044_03080			-	hypothetical protein	209	1470
1473	MGCS36044_00468			-	hypothetical protein	207	1473
1474	MGCS36044_01668			*nifS_1*	cysteine desulfurase NifS	207	1473
1475	MGCS36044_02570	Lipo		-	putative lipoprotein	207	1473
1476	MGCS36044_02524			*hemN*	HemN family coproporphyrinogen III oxidase or related Fe-S oxidoreductase	206	1476
1477	MGCS36044_03794			*oppB_2*	oligopeptide ABC transporter permease OppB	206	1476
1478	MGCS36044_01948			*eriC*	EriC family voltage gated chloride channel protein	205	1478

No.	Locus tag	SignalP6 predicted[1]	Virulence[2]	Gene	Function	RPKMs in NHP 15[3]	RANK[4]
1479	MGCS36044_02608			*trmK*	tRNA (adenine(22)-N(1))-methyltransferase TrmK	204	1479
1480	MGCS36044_01750			-	MdlB family multidrug ABC transporter ATPase and permease component	203	1480
1481	MGCS36044_03318			*atoB*	3-ketoacyl-CoA thiolase/acetyl-CoA acetyltransferase protein AtoB	202	1481
1482	MGCS36044_03784			-	DUF1447 family protein	202	1481
1483	MGCS36044_01976			*rluA_1*	RluA family pseudouridine synthase	200	1483
1484	MGCS36044_02260			*lipL*	lipoate--protein ligase	200	1483
1485	MGCS36044_03174			-	GNAT family N-acetyltransferase	197	1485
1486	MGCS36044_02592			-	hypothetical protein	196	1486
1487	MGCS36044_02326			-	ABC transporter permease	194	1487
1488	MGCS36044_02414			-	amino acid ABC transporter permease	192	1488
1489	MGCS36044_00348			-	DUF4651 domain-containing protein	191	1489
1490	MGCS36044_04172			-	MFS transporter	189	1490
1491	MGCS36044_00286			-	1,4-dihydroxy-2-naphthoate octaprenyltransferase	188	1491
1492	MGCS36044_00922			*xerD_1*	site-specific tyrosine recombinase XerD	188	1491
1493	MGCS36044_01890			*arsR*	cadmium efflux system metalloregulator ArsR/SmtB family transcription factor	185	1493
1494	MGCS36044_01122			-	IS1182 family transposase	182	1494
1495	MGCS36044_03848			-	DUF386 family protein	182	1494
1496	MGCS36044_03308			*priA*	primosomal protein PriA	180	1496
1497	MGCS36044_00870			*rsmG*	16S rRNA (guanine(527)-N(7))-methyltransferase RsmG	179	1497
1498	MGCS36044_04194			-	DUF4097 family protein	179	1497
1499	MGCS36044_02822			-	CD1845 family protein	177	1499
1500	MGCS36044_03184			-	MOP/MATE family multidrug-resistance efflux pump	177	1499
1501	MGCS36044_00622			-	DNA cytosine methyltransferase	176	1501
1502	MGCS36044_00338			-	helix-turn-helix transcriptional regulator	175	1502
1503	MGCS36044_01092			*yozE*	YozE family protein	174	1503
1504	MGCS36044_01692			-	PyrR RNA	174	1503
1505	MGCS36044_00422			*proX*	prolyl-tRNA synthetase associated domain-containing protein ProX	173	1505
1506	MGCS36044_03694			-	carbohydrate ABC transporter permease UgpA-like	173	1505
1507	MGCS36044_02938			*holA*	DNA polymerase III delta subunit HolA	172	1507
1508	MGCS36044_03700		Virulence	*cppA*	CppA family putative C3-glycoprotein degrading proteinase	172	1507
1509	MGCS36044_01148			-	putative bacteriocin with double-glycine leader peptide	171	1509

No.	Locus tag	SignalP6 predicted[1]	Virulence[2]	Gene	Function	RPKMs in NHP 15[3]	RANK[4]
1510	MGCS36044_02584			*yloA*	YloA family predicted ribosome quality control complex (RQC) component	171	1509
1511	MGCS36044_01404			*rpiB*	RpiB/LacA/LacB family sugar-phosphate isomerase	170	1511
1512	MGCS36044_01786			*thyA*	thymidylate synthase ThyA	169	1512
1513	MGCS36044_01940			*maeR*	TCS signal transduction response regulator MaeR	169	1512
1514	MGCS36044_02954	Lipo		*metQ*	methionine uptake ABC transporter substrate-binding lipoprotein MetQ	168	1514
1515	MGCS36044_01972			-	GTP pyrophosphokinase family protein	167	1515
1516	MGCS36044_03992	Secreted		-	TrbC/VirB2-related secreted effector protein	165	1516
1517	MGCS36044_01836			-	nucleotidyl transferase AbiEii/AbiGii toxin family	163	1517
1518	MGCS36044_00210			*adk*	adenylate kinase protein Adk	162	1518
1519	MGCS36044_03802			-	YgaE family protein	162	1518
1520	MGCS36044_02738			-	hypothetical protein	161	1520
1521	MGCS36044_00892			-	hypothetical protein	158	1521
1522	MGCS36044_02110			-	hypothetical protein	157	1522
1523	MGCS36044_03190			*aroGA*	bifunctional 3-deoxy-7-phosphoheptulonate synthase/chorismate mutase AroGA	157	1522
1524	MGCS36044_03792			*oppC_2*	oligopeptide ABC transporter permease OppC	155	1524
1525	MGCS36044_03876			-	MefA-related MFS transporter	153	1525
1526	MGCS36044_03234			-	ParB-like nuclease domain-containing protein	151	1526
1527	MGCS36044_03890			*queH*	epoxyqueuosine reductase QueH	150	1527
1528	MGCS36044_00610			-	helix-turn-helix transcriptional regulator	149	1528
1529	MGCS36044_04078			*catE*	catechol-2,3-dioxygenase CatE	149	1528
1530	MGCS36044_02830			*aroA*	3-phosphoshikimate 1-carboxyvinyltransferase AroA	146	1530
1531	MGCS36044_02284			*rsmC*	class I SAM-dependent methyltransferase	145	1531
1532	MGCS36044_01578			*osmC*	YhfA family OsmC-related salt-stress induced protein	143	1532
1533	MGCS36044_00408			*mipB*	fructose-6-phosphate aldolase MipB	142	1533
1534	MGCS36044_01576			*pheT*	phenylalanyl-tRNA synthetase beta subunit PheT	141	1534
1535	MGCS36044_02556			-	hypothetical protein	141	1534
1536	MGCS36044_02286			*coaA*	type I pantothenate kinase	140	1536
1537	MGCS36044_01724			*trxB_1*	NAD(P)/FAD-dependent oxidoreductase	139	1537
1538	MGCS36044_00128	Secreted		-	CHAP domain-containing protein	137	1538
1539	MGCS36044_01494			*rodA*	rod shape-determining protein RodA	137	1538
1540	MGCS36044_00456			-	aminoglycoside 6-adenylyltransferase	136	1540

No.	Locus tag	SignalP6 predicted[1]	Virulence[2]	Gene	Function	RPKMs in NHP 15[3]	RANK[4]
1541	MGCS36044_01234	Secreted		-	glutamine ABC transporter substrate-binding secreted protein	136	1540
1542	MGCS36044_00446			-	GNAT family N-acetyltransferase	132	1542
1543	MGCS36044_01262			*bglG_1*	transcriptional antiterminator BglG	128	1543
1544	MGCS36044_00470			-	hypothetical protein	127	1544
1545	MGCS36044_03460	Secreted		*aes*	Aes family secreted acetyl esterase/lipase	127	1544
1546	MGCS36044_02916			-	GNAT family N-acetyltransferase	125	1546
1547	MGCS36044_01888			-	DUF5960 family protein	124	1547
1548	MGCS36044_02104			-	hypothetical protein	124	1547
1549	MGCS36044_02330			*tetR*	TetR family transcriptional regulator	123	1549
1550	MGCS36044_01720			*rimM*	ribosome maturation factor RimM	121	1550
1551	MGCS36044_02564			-	ABC transporter permease component	121	1550
1552	MGCS36044_02030			-	vicinal oxygen chelate (VOC) family protein	120	1552
1553	MGCS36044_02182			*citC*	citrate lyase ligase CitC	117	1553
1554	MGCS36044_01592			*aspB*	aspartate aminotransferase AspB	116	1554
1555	MGCS36044_00630			-	ATP-binding protein	115	1555
1556	MGCS36044_02500			-	CRISPR-DR22 RNA	115	1555
1557	MGCS36044_02424			*pyrE*	orotate phosphoribosyltransferase PyrE	113	1557
1558	MGCS36044_01126		Virulence	*silA*	TCS DNA-binding response regulator SilA	112	1558
1559	MGCS36044_01766			*mvaK1*	mevalonate kinase MvaK1	112	1558
1560	MGCS36044_03480			*ecsB*	ABC exoprotein transporter permease EcsB	111	1560
1561	MGCS36044_00362		Virulence	*rofA*	pilus transcriptional regulator RofA	110	1561
1562	MGCS36044_02350			*alsT*	sodium:alanine symporter family protein	110	1561
1563	MGCS36044_00562			-	major facilitator transporter family protein	109	1563
1564	MGCS36044_01534			*queT*	Queuosine precursor transporter QueT	108	1564
1565	MGCS36044_03686			-	alpha-glycosidase	108	1564
1566	MGCS36044_03068			-	DUF3165 family protein	107	1566
1567	MGCS36044_03250			*recX*	recombination regulator RecX	107	1566
1568	MGCS36044_04026			-	cspA RNA	107	1566
1569	MGCS36044_02660			*malD*	maltodextrin transport system permease protein MalD	106	1569
1570	MGCS36044_03084			-	SmdA family multidrug ABC transporter ATPase/permease	106	1569
1571	MGCS36044_00550			-	nucleotidyltransferase family protein	105	1571

No.	Locus tag	SignalP6 predicted[1]	Virulence[2]	Gene	Function	RPKMs in NHP 15[3]	RANK[4]
1572	MGCS36044_02170			-	BaeS family TCS histidine kinase sensor	105	1571
1573	MGCS36044_03350			*ansP*	AnsP family L-asparagine transporter and related permeases	104	1573
1574	MGCS36044_03712			-	Xre family helix-turn-helix transcriptional regulator	102	1574
1575	MGCS36044_01168			-	hypothetical protein	101	1575
1576	MGCS36044_02400			*bcaT*	branched-chain amino acid aminotransferase protein BcaT	100	1576
1577	MGCS36044_00466			-	hypothetical protein	99	1577
1578	MGCS36044_01610			*rgpA*	glycosyltransferase family 1 protein RgpA	99	1577
1579	MGCS36044_03572			-	rhodanese-like domain-containing protein	98	1579
1580	MGCS36044_00538			-	thioredoxin family protein	97	1580
1581	MGCS36044_01064			-	Spy490380c RNA	86	1581
1582	MGCS36044_01258			-	MFS transporter	85	1582
1583	MGCS36044_02684			-	hypothetical protein	82	1583
1584	MGCS36044_01694			*pyrR*	bifunctional pyrimidine regulatory protein/uracil phosphoribosyltransferase PyrR	81	1584
1585	MGCS36044_02836			*spxR*	SpxR family CBS-HotDog domain-containing transcription factor	81	1584
1586	MGCS36044_03846			-	DUF624 domain-containing protein	80	1586
1587	MGCS36044_02788			-	DUF1413 domain-containing protein	77	1587
1588	MGCS36044_02978			*niaR*	niacin-responsive transcriptional repressor NadR	75	1588
1589	MGCS36044_03632			*scrB*	sucrose-6-phosphate hydrolase ScrB	75	1588
1590	MGCS36044_01856			-	DUF5965 family protein	74	1590
1591	MGCS36044_04258			*yitT*	membrane anchor protein YitT	73	1591
1592	MGCS36044_00624			-	helix-turn-helix transcriptional regulator	72	1592
1593	MGCS36044_00792			*sufB*	Fe-S cluster assembly protein SufB	72	1592
1594	MGCS36044_03556			-	GlsB/YeaQ/YmgE family stress response membrane protein	72	1592
1595	MGCS36044_01848			-	DUF5962 family protein	71	1595
1596	MGCS36044_02084			*kefB*	Kef-type K+ transporter membrane component KefB	71	1595
1597	MGCS36044_04244			*pqqF*	pitrilysin family predicted Zn-dependent peptidase	70	1597
1598	MGCS36044_01952			-	NanC-like SGNH/GDSL hydrolase family protein	69	1598
1599	MGCS36044_02666	Lipo		*malX*	maltose/maltodextrin-binding lipoprotein MalX	69	1598
1600	MGCS36044_04094			-	hypothetical protein	69	1598
1601	MGCS36044_00458			-	PhoE family phosphoglycerate mutase	68	1601
1602	MGCS36044_04068			*treB*	PTS transporter trehalose-specific EIIBC component TreB	68	1601

No.	Locus tag	SignalP6 predicted[1]	Virulence[2]	Gene	Function	RPKMs in NHP 15[3]	RANK[4]
1603	MGCS36044_00706			-	Cof-type HAD-IIB family hydrolase	66	1603
1604	MGCS36044_02626			*miaA*	tRNA (adenosine(37)-N6)-dimethylallyltransferase MiaA	66	1603
1605	MGCS36044_02706			-	DUF4044 domain-containing protein	66	1603
1606	MGCS36044_03496			*cof*	Cof family hydrolase	66	1603
1607	MGCS36044_00282			-	Spy392987 RNA	65	1607
1608	MGCS36044_01012			-	GloA family glyoxalase/bleomycin resistance/extradiol dioxygenase family protein	65	1607
1609	MGCS36044_01244			*rnc*	ribonuclease III Rnc	65	1607
1610	MGCS36044_03180			-	DUF1912 family protein	65	1607
1611	MGCS36044_00704			-	YjbQ family protein	64	1611
1612	MGCS36044_02172			-	OmpR family TCS DNA-binding response regulator	64	1611
1613	MGCS36044_02420			-	IS3 family transposase	64	1611
1614	MGCS36044_03780			*rimI*	ribosomal-protein-alanine N-acetyltransferase RimI	64	1611
1615	MGCS36044_04008			-	hypothetical protein	64	1611
1616	MGCS36044_04190			*padR*	PadR family transcriptional regulator	64	1611
1617	MGCS36044_00928			*rluB*	ribosomal large subunit pseudouridine synthase RluB	63	1617
1618	MGCS36044_00942			*yhcC*	YhcC family Fe-S oxidoreductase	63	1617
1619	MGCS36044_02164			-	polyprenyl synthetase family protein	62	1619
1620	MGCS36044_04204			-	helix-turn-helix domain-containing transcriptional regulator	62	1619
1621	MGCS36044_00454			-	type II toxin-antitoxin system PemK/MazF family toxin	61	1621
1622	MGCS36044_02158			-	1,4-dihydroxy-2-naphthoate polyprenyltransferase	61	1621
1623	MGCS36044_02354			-	IS1182 family transposase	61	1621
1624	MGCS36044_04248			*ybcJ*	ribosome associated protein YbcJ	61	1621
1625	MGCS36044_00726			*truA*	tRNA pseudouridine(38-40) synthase TruA	60	1625
1626	MGCS36044_02694			*bglG_2*	transcription antiterminator BglG	60	1625
1627	MGCS36044_00430			-	ATP-binding cassette domain-containing protein	59	1627
1628	MGCS36044_02934	Lipo		*blaA*	beta-lactamase-related serine hydrolase lipoprotein	59	1627
1629	MGCS36044_04148			-	cadmium efflux system accessory protein	58	1629
1630	MGCS36044_01928			*folK*	2-amino-4-hydroxy-6- hydroxymethyldihydropteridine pyrophosphokinase protein FolK	57	1630
1631	MGCS36044_01808			-	hypothetical protein	56	1631
1632	MGCS36044_02576			-	hypothetical protein	56	1631

No.	Locus tag	SignalP6 predicted[(1)]	Virulence[(2)]	Gene	Function	RPKMs in NHP 15[(3)]	RANK[(4)]
1633	MGCS36044_00368			*srtC_1*	class C sortase SrtC	55	1633
1634	MGCS36044_00438			-	bacteriocin immunity protein	55	1633
1635	MGCS36044_01904			-	Cro/CI family transcriptional regulator	55	1633
1636	MGCS36044_02166			-	NAD(P)/FAD-dependent oxidoreductase	55	1633
1637	MGCS36044_02426			*pyrF*	orotidine-5'-phosphate decarboxylase PyrF	55	1633
1638	MGCS36044_02228			*pdxK*	pyridoxamine kinase PdxK	54	1638
1639	MGCS36044_03840			*rpiR*	RpiR family transcriptional regulator	54	1638
1640	MGCS36044_03872		Virulence	*fasB*	signal transduction sensor histidine kinase protein FasB	54	1638
1641	MGCS36044_04176			-	radical SAM protein	53	1641
1642	MGCS36044_00626			-	MvaI/BcnI family restriction endonuclease	52	1642
1643	MGCS36044_01780			-	HTH domain-containing putative transcriptional regulator	52	1642
1644	MGCS36044_02174			-	ABC transporter permease	52	1642
1645	MGCS36044_02510			*cas2_1*	CRISPR-associated endoribonuclease Cas2	52	1642
1646	MGCS36044_03450	Secreted		-	pilus backbone/major protein	52	1642
1647	MGCS36044_01440			-	DUF1003 domain-containing protein	51	1647
1648	MGCS36044_03510			-	acyltransferase family protein	51	1647
1649	MGCS36044_01210			*dhaQ*	DhaKLM operon coactivator DhaQ	50	1649
1650	MGCS36044_01222			-	acyl dehydratase	50	1649
1651	MGCS36044_02108			-	hypothetical protein	50	1649
1652	MGCS36044_02664			*amyB*	cyclomaltodextrinase protein AmyB	50	1649
1653	MGCS36044_03158			*cas4*	CRISPR-associated protein Cas4	50	1649
1654	MGCS36044_03768			*dnaQ*	DNA polymerase III epsilon subunit DnaQ	50	1649
1655	MGCS36044_00366	Secreted		-	secreted pilin minor/ancillary protein	49	1655
1656	MGCS36044_00686			-	toxin-antitoxin system, toxin component, Fic domain protein	49	1655
1657	MGCS36044_02966			*murF*	UDP-N-acetylmuramoyl-tripeptide--D-alanyl-D- alanine ligase MurF	49	1655
1658	MGCS36044_02974			*focA*	formate transporter FocA	49	1655
1659	MGCS36044_01166			-	ABC transporter ATP-binding protein	47	1659
1660	MGCS36044_02512			*cas1_1*	type II CRISPR-associated endonuclease Cas1	47	1659
1661	MGCS36044_04174			-	arginase family protein	47	1659
1662	MGCS36044_01580			*ybgA*	YbgA family DUF1722 domain-containing protein	43	1662
1663	MGCS36044_00012			*pth*	aminoacyl-tRNA hydrolase Pth	0	1663

No.	Locus tag	SignalP6 predicted(1)	Virulence(2)	Gene	Function	RPKMs in NHP 15(3)	RANK(4)
1664	MGCS36044_00014			*trcF*	transcription-repair coupling factor TcrF	0	1663
1665	MGCS36044_00016			-	oligosaccharide flippase family protein MurJ-like	0	1663
1666	MGCS36044_00018			-	RNA-binding S4 domain-containing protein	0	1663
1667	MGCS36044_00020			*divIC*	septum formation initiator family protein FtsB/DivIC	0	1663
1668	MGCS36044_00108			*recO*	DNA repair protein RecO	0	1663
1669	MGCS36044_00110			*plsX*	phosphate acyltransferase PlsX	0	1663
1670	MGCS36044_00114			*purC*	phosphoribosylaminoimidazolesuccinocarboxamide synthase PurC	0	1663
1671	MGCS36044_00132			*purE*	5-(carboxyamino)imidazole ribonucleotide mutase PurE	0	1663
1672	MGCS36044_00142			-	helix-turn-helix domain-containing protein	0	1663
1673	MGCS36044_00144			-	quorum-sensing system DWW-type pheromone	0	1663
1674	MGCS36044_00162			-	MATE family multidrug efflux transporter	0	1663
1675	MGCS36044_00262			-	FRG domain-containing protein	0	1663
1676	MGCS36044_00264			*xerC*	site-specific integrase	0	1663
1677	MGCS36044_00266			-	DUF3173 domain-containing protein	0	1663
1678	MGCS36044_00268			-	Rep family plasmid replication protein	0	1663
1679	MGCS36044_00270			-	hypothetical protein	0	1663
1680	MGCS36044_00272			-	hypothetical protein	0	1663
1681	MGCS36044_00274			-	helix-turn-helix transcriptional regulator	0	1663
1682	MGCS36044_00322			*comYB*	competence system type II secretion system protein ComYB	0	1663
1683	MGCS36044_00324	Pilin		*comYC*	competence system prepilin-type N-terminal cleavage/methylation domain-containing protein ComYC	0	1663
1684	MGCS36044_00326	Pilin		*comYD*	competence system type II secretion system GspH family protein ComYD	0	1663
1685	MGCS36044_00328	Pilin		*comYE*	competence system protein ComYE	0	1663
1686	MGCS36044_00330	Pilin		*comYF*	competence system protein ComYF	0	1663
1687	MGCS36044_00332			*comYG*	competence system protein ComYG	0	1663
1688	MGCS36044_00354			*ssb_1*	single-stranded DNA-binding protein Ssb	0	1663
1689	MGCS36044_00364	Secreted		-	secreted pilin backbone/major protein	0	1663
1690	MGCS36044_00370			*srtC_2*	class C sortase SrtC	0	1663
1691	MGCS36044_00372	Secreted		-	secreted pilin minor/ancillary protein	0	1663
1692	MGCS36044_00376			*srtC_3*	class C sortase SrtC	0	1663
1693	MGCS36044_00382			*ridA*	RidA family protein	0	1663
1694	MGCS36044_00400			*celC_1*	PTS transporter cellobiose-specific IIA component protein CelC	0	1663

No.	Locus tag	SignalP6 predicted(1)	Virulence(2)	Gene	Function	RPKMs in NHP 15(3)	RANK(4)
1695	MGCS36044_00416			-	hypothetical protein	0	1663
1696	MGCS36044_00420			-	hypothetical protein	0	1663
1697	MGCS36044_00434			-	hypothetical protein	0	1663
1698	MGCS36044_00436			-	Maf family protein	0	1663
1699	MGCS36044_00460			-	helix-turn-helix transcriptional regulator	0	1663
1700	MGCS36044_00462	Secreted		-	cell surface PgrA surface exclusion domain-containing protein	0	1663
1701	MGCS36044_00464			-	hypothetical protein	0	1663
1702	MGCS36044_00472			-	FtsK/SpoIIIE domain-containing protein	0	1663
1703	MGCS36044_00474			-	Rep family protein	0	1663
1704	MGCS36044_00476			-	DUF771 domain-containing protein	0	1663
1705	MGCS36044_00478			-	site-specific integrase	0	1663
1706	MGCS36044_00514		Virulence	*mga*	M protein trans-acting positive regulator Mga	0	1663
1707	MGCS36044_00528			*lrp*	PucR family transcriptional regulator/leucine rich protein Lrp	0	1663
1708	MGCS36044_00536			-	hypothetical protein	0	1663
1709	MGCS36044_00608			-	site-specific integrase	0	1663
1710	MGCS36044_00612			-	DNA-binding protein	0	1663
1711	MGCS36044_00614			-	phage replisome organizer N-terminal domain-containing protein	0	1663
1712	MGCS36044_00616			-	DUF5962 domain-containing protein	0	1663
1713	MGCS36044_00618			-	bacterial mobilization protein	0	1663
1714	MGCS36044_00620			-	transposase	0	1663
1715	MGCS36044_00628			-	SIR2 family protein	0	1663
1716	MGCS36044_00634			-	helix-turn-helix domain-containing protein	0	1663
1717	MGCS36044_00636	Secreted		-	CHAP domain-containing protein	0	1663
1718	MGCS36044_00638			-	putative conjugal transfer protein	0	1663
1719	MGCS36044_00640			-	conjugal transfer protein	0	1663
1720	MGCS36044_00642			-	DNA segregation ATPase, conjugal transfer protein	0	1663
1721	MGCS36044_00644			-	conjugal transfer protein	0	1663
1722	MGCS36044_00646			-	conjugal transfer protein	0	1663
1723	MGCS36044_00648			-	conjugal transfer protein	0	1663
1724	MGCS36044_00650			-	conjugal transfer protein	0	1663
1725	MGCS36044_00652			-	putative conjugal transfer protein	0	1663

No.	Locus tag	SignalP6 predicted[(1)]	Virulence[(2)]	Gene	Function	RPKMs in NHP 15[(3)]	RANK[(4)]
1726	MGCS36044_00654			-	replication initiation factor domain-containing protein	0	1663
1727	MGCS36044_00660			-	FtsK/SpoIIIE domain-containing protein	0	1663
1728	MGCS36044_00662			-	conjugal transfer protein	0	1663
1729	MGCS36044_00664			-	cytoplasmic protein	0	1663
1730	MGCS36044_00666			-	hypothetical protein	0	1663
1731	MGCS36044_00668			-	Cro/CI family transcriptional regulator	0	1663
1732	MGCS36044_00674			-	ImmA/IrrE family metallo-endopeptidase	0	1663
1733	MGCS36044_00676			-	XRE family transcriptional regulator	0	1663
1734	MGCS36044_00678			-	bacteriocin	0	1663
1735	MGCS36044_00680			-	lantibiotic dehydratase	0	1663
1736	MGCS36044_00682			-	lanthionine synthetase C family protein	0	1663
1737	MGCS36044_00684			-	lantibiotic transporter	0	1663
1738	MGCS36044_00688			-	hypothetical protein	0	1663
1739	MGCS36044_00690			-	tyrosine-type site-specific recombinase/integrase	0	1663
1740	MGCS36044_00692			-	PRD domain/PTS transporter IIA domain protein	0	1663
1741	MGCS36044_00696			*gatC_1*	PTS galactitol transporter IIC component GatC	0	1663
1742	MGCS36044_00698			*gatB_1*	PTS galactitol transporter IIB component GatB	0	1663
1743	MGCS36044_00702			-	triose-phosphate isomerase	0	1663
1744	MGCS36044_00722			*comX_1*	competence protein ComX	0	1663
1745	MGCS36044_00724			-	aspartate kinase	0	1663
1746	MGCS36044_00796			*dacA_2*	D-alanyl-D-alanine carboxypeptidase DacA	0	1663
1747	MGCS36044_00822			*comX_2*	competence protein ComX	0	1663
1748	MGCS36044_00852	Lipo		-	MetQ/NlpA family ABC transporter substrate-binding lipoprotein	0	1663
1749	MGCS36044_00854	Lipo		-	MetQ/NlpA family ABC transporter substrate-binding lipoprotein	0	1663
1750	MGCS36044_00856			*dapE*	M20/M25/M40 family metallo-hydrolase, succinyl-diaminopimelate desuccinylase DapE	0	1663
1751	MGCS36044_00930			*yidD*	membrane protein insertion efficiency factor YidD	0	1663
1752	MGCS36044_00976	Lipo		*livK*	branched-chain amino acid ABC transporter substrate-binding lipoprotein LivK	0	1663
1753	MGCS36044_00978			*livH*	branched-chain amino acid ABC transporter permease LivH	0	1663
1754	MGCS36044_00980			*livM*	branched-chain amino acid ABC transporter permease LivM	0	1663
1755	MGCS36044_00982			*livG*	branched-chain amino acid ABC transporter ATP-binding protein LivG	0	1663
1756	MGCS36044_00984			*livF*	branched-chain amino acid ABC transporter ATP-binding protein LivF	0	1663

No.	Locus tag	SignalP6 predicted[1]	Virulence[2]	Gene	Function	RPKMs in NHP 15[3]	RANK[4]
1757	MGCS36044_00986			-	cystathionine beta-synthase (CBS) domain-containing protein	0	1663
1758	MGCS36044_01026	Secreted		*yoaK*	putative secreted YoaK family protein	0	1663
1759	MGCS36044_01038			-	hypothetical protein	0	1663
1760	MGCS36044_01040	Secreted		-	putative secreted protein	0	1663
1761	MGCS36044_01110			-	hypothetical protein	0	1663
1762	MGCS36044_01116			-	Blp family class II bacteriocin with double-glycine leader peptide	0	1663
1763	MGCS36044_01134		Virulence	*silCR*	streptococcal invasion locus auto-inducing pheromone peptide SilCR	0	1663
1764	MGCS36044_01136			*silC*	streptococcal invasion locus signaling peptide SilC	0	1663
1765	MGCS36044_01138		Virulence	*silD*	streptococcal invasion locus pheromone secretion accessory protein SilD	0	1663
1766	MGCS36044_01140		Virulence	*silE*	streptococcal invasion locus pheromone processing peptide cleavage/export ABC transporter SilE	0	1663
1767	MGCS36044_01142			-	BlpM-like bacteriocin with double-glycine leader peptide	0	1663
1768	MGCS36044_01144			-	lactobin A/cerein 7B family class IIb bacteriocin with double-glycine secretion leader peptide	0	1663
1769	MGCS36044_01146			-	putative bacteriocin with double-glycine leader peptide	0	1663
1770	MGCS36044_01150			-	hypothetical protein	0	1663
1771	MGCS36044_01152			-	CAAX amino terminal protease family protein	0	1663
1772	MGCS36044_01154			-	hypothetical protein	0	1663
1773	MGCS36044_01158	Secreted		-	secreted transglutaminase	0	1663
1774	MGCS36044_01212			*dhaS*	dihydroxyacetone kinase transcriptional activator DhaS	0	1663
1775	MGCS36044_01232			-	glutamine ABC transporter permease	0	1663
1776	MGCS36044_01260			-	Spy490483c RNA	0	1663
1777	MGCS36044_01264			*bglF*	PTS beta-glucoside transporter IIBCA component BglF	0	1663
1778	MGCS36044_01292	Secreted		-	putative secreted protein	0	1663
1779	MGCS36044_01310			-	PrtC family collagenase-like protease	0	1663
1780	MGCS36044_01356			-	hypothetical protein	0	1663
1781	MGCS36044_01358			-	hypothetical protein	0	1663
1782	MGCS36044_01360			-	glycosyltransferase	0	1663
1783	MGCS36044_01362			-	glycosyltransferase family 2 protein	0	1663
1784	MGCS36044_01380			-	DUF1934 domain-containing protein	0	1663
1785	MGCS36044_01390			-	oligohyaluronate lyase	0	1663
1786	MGCS36044_01402			*idnO*	gluconate 5-dehydrogenase IdnO	0	1663

No.	Locus tag	SignalP6 predicted(1)	Virulence(2)	Gene	Function	RPKMs in NHP 15(3)	RANK(4)
1787	MGCS36044_01416			-	IS3 family transposase	0	1663
1788	MGCS36044_01418			-	transposase	0	1663
1789	MGCS36044_01502			*serB*	phosphoserine phosphatase SerB	0	1663
1790	MGCS36044_01532			-	preQ1-II RNA	0	1663
1791	MGCS36044_01566			-	DUF1146 domain-containing protein	0	1663
1792	MGCS36044_01574			*pheS*	phenylalanyl-tRNA synthetase alpha subunit PheS	0	1663
1793	MGCS36044_01774			-	HTH domain-containing putative transcriptional regulator	0	1663
1794	MGCS36044_01776	Secreted		-	extracellular cell wall anchored mucin-binding (MucBP) domain-containing protein	0	1663
1795	MGCS36044_01812			-	DNA (cytosine-5-)-methyltransferase	0	1663
1796	MGCS36044_01814			-	hypothetical protein	0	1663
1797	MGCS36044_01816			-	arsenate reductase	0	1663
1798	MGCS36044_01818			-	hypothetical protein	0	1663
1799	MGCS36044_01820			-	CPBP family intramembrane metalloprotease	0	1663
1800	MGCS36044_01822			-	hypothetical protein	0	1663
1801	MGCS36044_01824			*traG_1*	type IV secretory system conjugative DNA transfer family protein TraG	0	1663
1802	MGCS36044_01826			-	hypothetical protein	0	1663
1803	MGCS36044_01828			*trbL*	conjugal transfer protein TrbL	0	1663
1804	MGCS36044_01830			-	PrgI family protein	0	1663
1805	MGCS36044_01832			-	AAA family ATPase	0	1663
1806	MGCS36044_01834			-	phage tail tip lysozyme	0	1663
1807	MGCS36044_01840	Secreted		-	cell surface extracellular antigen I/II family adhesin	0	1663
1808	MGCS36044_01842			-	calcium-binding protein	0	1663
1809	MGCS36044_01844			-	hypothetical protein	0	1663
1810	MGCS36044_01846			-	SNF2-related protein	0	1663
1811	MGCS36044_01850			-	DUF5966 family protein	0	1663
1812	MGCS36044_01852			-	hypothetical protein	0	1663
1813	MGCS36044_01854			-	toprim domain-containing protein	0	1663
1814	MGCS36044_01858			-	DUF5945 family protein	0	1663
1815	MGCS36044_01860			-	hypothetical protein	0	1663
1816	MGCS36044_01862			-	DUF5960 family protein	0	1663
1817	MGCS36044_01864			-	hypothetical protein	0	1663

No.	Locus tag	SignalP6 predicted[1]	Virulence[2]	Gene	Function	RPKMs in NHP 15[3]	RANK[4]
1818	MGCS36044_01874			*cadD_1*	CadD family cadmium resistance transporter	0	1663
1819	MGCS36044_01884			-	TIGR01906 family membrane protein	0	1663
1820	MGCS36044_01886			-	hypothetical protein	0	1663
1821	MGCS36044_01898			-	SAG1252 family conjugative relaxosome accessory protein	0	1663
1822	MGCS36044_01900			-	MobC family plasmid mobilization relaxosome protein	0	1663
1823	MGCS36044_01902			-	SAG1250 family conjugative relaxase	0	1663
1824	MGCS36044_01906			-	pemK RNA	0	1663
1825	MGCS36044_01908			-	DUF3173 domain-containing protein	0	1663
1826	MGCS36044_01942			*maeK*	TCS signal transduction sensor histidine kinase MaeK	0	1663
1827	MGCS36044_01944			*maeP*	Citrate/malate symporter protein MaeP	0	1663
1828	MGCS36044_01946			*maeE*	NAD-dependent malic enzyme MaeE	0	1663
1829	MGCS36044_01950			*araC*	AraC family transcriptional regulator	0	1663
1830	MGCS36044_01970			*yjbK*	YbjK superfamily CYTH domain-containing protein	0	1663
1831	MGCS36044_01990			-	purine RNA	0	1663
1832	MGCS36044_02080			-	IS30 family transposase	0	1663
1833	MGCS36044_02082			*femX*	FemABX-like family peptidoglycan interpeptide bridge formation enzyme	0	1663
1834	MGCS36044_02092			-	hypothetical protein	0	1663
1835	MGCS36044_02094			-	FtsK/SpoIIIE domain-containing protein	0	1663
1836	MGCS36044_02096			-	hypothetical protein	0	1663
1837	MGCS36044_02098			-	hypothetical protein	0	1663
1838	MGCS36044_02100			-	XRE family HTH-type transcriptional regulator	0	1663
1839	MGCS36044_02102			-	hypothetical protein	0	1663
1840	MGCS36044_02106			-	hypothetical protein	0	1663
1841	MGCS36044_02112			-	helix-turn-helix domain-containing protein	0	1663
1842	MGCS36044_02114			-	hypothetical protein	0	1663
1843	MGCS36044_02116			-	hypothetical protein	0	1663
1844	MGCS36044_02118			-	Rep family protein	0	1663
1845	MGCS36044_02120			-	hypothetical protein	0	1663
1846	MGCS36044_02122			-	DUF3173 family protein	0	1663
1847	MGCS36044_02124			-	site-specific integrase	0	1663
1848	MGCS36044_02154			*xerS*	site-specific tyrosine recombinase XerS	0	1663

No.	Locus tag	SignalP6 predicted[1]	Virulence[2]	Gene	Function	RPKMs in NHP 15[3]	RANK[4]
1849	MGCS36044_02168			-	Gx transporter family protein	0	1663
1850	MGCS36044_02176			-	ABC transporter ATPase	0	1663
1851	MGCS36044_02178			-	S41 family peptidase	0	1663
1852	MGCS36044_02184			*oadA*	oxaloacetate decarboxylase subunit alpha OadA	0	1663
1853	MGCS36044_02196			-	IS982 family transposase	0	1663
1854	MGCS36044_02202			*dprA*	DNA-processing protein DprA	0	1663
1855	MGCS36044_02208			-	MFS transporter	0	1663
1856	MGCS36044_02222			-	Uup family ATPase components of ABC transporters with duplicated ATPase domains	0	1663
1857	MGCS36044_02226			-	ECF transporter S component	0	1663
1858	MGCS36044_02230			*norG*	PLP-dependent aminotransferase family protein	0	1663
1859	MGCS36044_02234			*asd*	aspartate-semialdehyde dehydrogenase	0	1663
1860	MGCS36044_02362			*phnA*	PnhA family zinc ribbon domain-containing protein	0	1663
1861	MGCS36044_02418			-	GatA family amidase	0	1663
1862	MGCS36044_02508			*csn2*	CRISPR-associated protein Csn2	0	1663
1863	MGCS36044_02514			*cas9*	type II CRISPR RNA-guided endonuclease Cas9	0	1663
1864	MGCS36044_02526			-	hypothetical protein	0	1663
1865	MGCS36044_02554			-	ABC transporter ATP-binding protein	0	1663
1866	MGCS36044_02566			-	ABC transporter substrate binding component	0	1663
1867	MGCS36044_02582			-	hypothetical protein	0	1663
1868	MGCS36044_02662			*malC*	maltodextrin transport system permease protein MalC	0	1663
1869	MGCS36044_02686			*celB_2*	PTS cellobiose transporter IIC subunit CelB	0	1663
1870	MGCS36044_02688			-	DUF3284 domain-containing protein	0	1663
1871	MGCS36044_02690			*celC_2*	PTS cellobiose transporter IIA subunit CelC	0	1663
1872	MGCS36044_02692			*celA_2*	PTS cellobiose transporter IIB subunit CelA	0	1663
1873	MGCS36044_02698			*bglB_1*	6-phospho-beta-glucosidase BglB	0	1663
1874	MGCS36044_02700			*pnuC*	nicotinamide riboside transporter PnuC	0	1663
1875	MGCS36044_02716			-	MFS transporter	0	1663
1876	MGCS36044_02732			*dcm*	DNA (cytosine-5-)-methyltransferase Dcm	0	1663
1877	MGCS36044_02734			-	hypothetical protein	0	1663
1878	MGCS36044_02736			-	RepA N-terminal domain-containing protein	0	1663
1879	MGCS36044_02742			-	hypothetical protein	0	1663

No.	Locus tag	SignalP6 predicted[1]	Virulence[2]	Gene	Function	RPKMs in NHP 15[3]	RANK[4]
1880	MGCS36044_02744			-	pemK RNA	0	1663
1881	MGCS36044_02746			-	sigma-70 family RNA polymerase sigma factor like protein	0	1663
1882	MGCS36044_02752			-	TetR/AcrR family transcriptional regulator	0	1663
1883	MGCS36044_02754			*mobC*	plasmid mobilization relaxosome protein MobC	0	1663
1884	MGCS36044_02756			-	relaxase	0	1663
1885	MGCS36044_02758			-	conjugative element protein	0	1663
1886	MGCS36044_02760			-	DNA methyltransferase	0	1663
1887	MGCS36044_02764			*topB*	DNA topoisomerase III TopB	0	1663
1888	MGCS36044_02766	Secreted		-	DUF4366 domain-containing predicted secreted protein	0	1663
1889	MGCS36044_02768			-	conjugal transfer protein	0	1663
1890	MGCS36044_02770			-	CHAP domain-containing protein	0	1663
1891	MGCS36044_02772			*virB4*	type IV secretory pathway component VirB4	0	1663
1892	MGCS36044_02774			-	conjugal transfer protein	0	1663
1893	MGCS36044_02776			*virB6*	VirB6-like conjugal transfer protein	0	1663
1894	MGCS36044_02778			-	Maff2 family protein	0	1663
1895	MGCS36044_02780			-	single-stranded DNA-binding protein	0	1663
1896	MGCS36044_02782			*traG_2*	conjugal transfer protein TraG	0	1663
1897	MGCS36044_02784			*tnpW*	TnpW family transposon-encoded protein	0	1663
1898	MGCS36044_02790			-	phage replisome organizer N-terminal domain-containing protein	0	1663
1899	MGCS36044_02792			*mobA*	MobA/MobL family protein	0	1663
1900	MGCS36044_02794			-	DUF3847 domain-containing protein	0	1663
1901	MGCS36044_02796			-	sigma-70 family RNA polymerase sigma factor like protein	0	1663
1902	MGCS36044_02810			-	site-specific serine recombinase family protein	0	1663
1903	MGCS36044_02812			*virD4*	type IV secretory system conjugative DNA transfer protein VirD4	0	1663
1904	MGCS36044_02814			-	DUF3801 domain-containing protein	0	1663
1905	MGCS36044_02816			-	NTPase domain-containing protein	0	1663
1906	MGCS36044_02818			-	RepA N-terminal domain-containing protein	0	1663
1907	MGCS36044_02838			-	GNAT family N-acetyltransferase	0	1663
1908	MGCS36044_02922			*coiA*	competence protein CoiA	0	1663
1909	MGCS36044_02940			*comEC*	DNA internalization-related competence protein ComEC	0	1663
1910	MGCS36044_02942			*comEA*	DNA uptake competence protein ComEA	0	1663

No.	Locus tag	SignalP6 predicted[(1)]	Virulence[(2)]	Gene	Function	RPKMs in NHP 15[(3)]	RANK[(4)]
1911	MGCS36044_02946			*trmN6*	tRNA1(Val) A37 N6-methylase TrmN6	0	1663
1912	MGCS36044_02948			-	GIY-YIG catalytic domain-containing putative endonuclease	0	1663
1913	MGCS36044_02982			-	IS982 family transposase	0	1663
1914	MGCS36044_02990			-	Rgg/GadR/MutR family transcriptional regulator	0	1663
1915	MGCS36044_02992			-	MFS transporter	0	1663
1916	MGCS36044_03002	Secreted		-	extracellular cell surface putative nucleotidase	0	1663
1917	MGCS36044_03066			-	putative secreted protein	0	1663
1918	MGCS36044_03156			*cas1_2*	type I-C CRISPR-associated endonuclease Cas1	0	1663
1919	MGCS36044_03160			*cas7*	type I-C CRISPR-associated protein Cas7/Csd2	0	1663
1920	MGCS36044_03162			*cas8*	type I-C CRISPR-associated protein Cas8c/Csd1	0	1663
1921	MGCS36044_03164			*csd1*	Csd1 family CRISPR-associated protein	0	1663
1922	MGCS36044_03166			*cas5*	type I-C CRISPR-associated protein Cas5	0	1663
1923	MGCS36044_03168			*cas3*	CRISPR-associated helicase Cas3	0	1663
1924	MGCS36044_03182			-	MarR family winged helix-turn-helix transcriptional regulator	0	1663
1925	MGCS36044_03194	Lipo		-	putative lipoprotein	0	1663
1926	MGCS36044_03218			*nagC*	NagC family sugar kinase	0	1663
1927	MGCS36044_03220			-	COG3537 superfamily putative alpha-1,2-mannosidase	0	1663
1928	MGCS36044_03230			-	metal-independent alpha-mannosidase	0	1663
1929	MGCS36044_03236			*yncA*	YncA family L-amino acid N-acyltransferase	0	1663
1930	MGCS36044_03284			*comFA*	ComFA family DNA/RNA helicase	0	1663
1931	MGCS36044_03286			-	YIH1 family putative translation regulator	0	1663
1932	MGCS36044_03316			-	LysR family transcriptional regulator	0	1663
1933	MGCS36044_03338			*drpA*	DNA processing protein (A) DprA	0	1663
1934	MGCS36044_03360	Secreted		*hisJ*	HisJ family amino acid ABC transporter substrate-binding secreted protein	0	1663
1935	MGCS36044_03444			-	IS982 family transposase	0	1663
1936	MGCS36044_03452			*sipA*	signal peptidase I SipA	0	1663
1937	MGCS36044_03454	Secreted		-	pilus ancillary/minor protein 1	0	1663
1938	MGCS36044_03458			-	IS982 family transposase	0	1663
1939	MGCS36044_03500			-	SSRC34_1 RNA	0	1663
1940	MGCS36044_03574			-	CoA-disulfide reductase	0	1663
1941	MGCS36044_03576			-	rhodanese-like domain-containing protein	0	1663

No.	Locus tag	SignalP6 predicted[1]	Virulence[2]	Gene	Function	RPKMs in NHP 15[3]	RANK[4]
1942	MGCS36044_03648			-	CorA family divalent cation transport protein	0	1663
1943	MGCS36044_03710			-	Xre family helix-turn-helix transcriptional regulator	0	1663
1944	MGCS36044_03742			-	hypothetical protein	0	1663
1945	MGCS36044_03748			-	hypothetical protein	0	1663
1946	MGCS36044_03750			-	HTH cro/C1-type domain-containing protein	0	1663
1947	MGCS36044_03752			-	hypothetical protein	0	1663
1948	MGCS36044_03754			-	type IV secretion system DNA-binding domain-containing protein	0	1663
1949	MGCS36044_03756			-	hypothetical protein	0	1663
1950	MGCS36044_03758			-	replication protein	0	1663
1951	MGCS36044_03760			-	DUF3173 family protein	0	1663
1952	MGCS36044_03766			-	MerR/SoxR family transcriptional regulator	0	1663
1953	MGCS36044_03774			-	hypothetical protein	0	1663
1954	MGCS36044_03830			-	hypothetical protein	0	1663
1955	MGCS36044_03838			-	cysteine hydrolase	0	1663
1956	MGCS36044_03844			*nanA*	N-acetylneuraminate lyase	0	1663
1957	MGCS36044_03908	Secreted	Virulence	*fbpB*	cell surface fibronectin binding protein (B) FbpB	0	1663
1958	MGCS36044_03914			-	hypothetical protein	0	1663
1959	MGCS36044_03916			-	hypothetical protein	0	1663
1960	MGCS36044_03922			*resA*	TlpA family protein disulfide reductase	0	1663
1961	MGCS36044_03960			*bglG_3*	BglG family transcription antiterminator	0	1663
1962	MGCS36044_03984			-	MdlB family ABC transporter ATP-binding/permease protein	0	1663
1963	MGCS36044_03986			-	hypothetical protein	0	1663
1964	MGCS36044_03988			-	hypothetical protein	0	1663
1965	MGCS36044_03990			-	IS982 family transposase	0	1663
1966	MGCS36044_04066			*treC*	trehalose-6-phosphate hydrolase TreC	0	1663
1967	MGCS36044_04070			*treR*	trehalose operon repressor TreR	0	1663
1968	MGCS36044_04130			-	Uup family of ABC transporter with duplicated ATPase domains	0	1663
1969	MGCS36044_04146			*cadD*	CadD family cadmium resistance transporter	0	1663
1970	MGCS36044_04150			-	DNA-binding HTH domain-containing transcriptional regulator	0	1663
1971	MGCS36044_04152			-	sigma-70 family RNA polymerase sigma factor	0	1663
1972	MGCS36044_04154			-	hypothetical protein	0	1663

No.	Locus tag	SignalP6 predicted(1)	Virulence(2)	Gene	Function	RPKMs in NHP 15(3)	RANK(4)
1973	MGCS36044_04156			-	FtsK/SpoIIIE domain-containing protein	0	1663
1974	MGCS36044_04158			-	hypothetical protein	0	1663
1975	MGCS36044_04160			-	replication protein	0	1663
1976	MGCS36044_04162			-	MerR family transcriptional regulator	0	1663
1977	MGCS36044_04170			-	parvulin-like peptidyl-prolyl isomerase	0	1663
1978	MGCS36044_04178			-	IS982 family transposase	0	1663
1979	MGCS36044_04182			-	Rgg/GadR/MutR family transcriptional regulator	0	1663
1980	MGCS36044_04188			-	NUDIX hydrolase	0	1663
1981	MGCS36044_04192			-	DUF1700 domain-containing protein	0	1663
1982	MGCS36044_04196			-	YhgE/Pip domain-containing protein	0	1663
1983	MGCS36044_04198			-	hypothetical protein	0	1663
1984	MGCS36044_04200			-	IS1182 family transposase	0	1663
1985	MGCS36044_04220			-	Spd-sr37 RNA	0	1663
1986	MGCS36044_04246			*pqqL*	pitrilysin family predicted Zn-dependent peptidase	0	1663

MGCS36044 was recovered from NHP 15 biopsies. 1,662 genes had mapped transcript reads whereas 324 genes did not.

(1) Genes predicted by SignalP 6.0 to have an export signal sequence
(2) Virulence refers to putative virulence genes
(3) RPKMs were assigned by EDGEPro
(4) Rank refers to the corresponding transcript abundance rank based on RPKMs
(5) Lipo, exported lipoprotein attached to the cell envelope
(6) TCS, two-component system

Table S4B. Ranked MGCS36089 genes during *in vivo* infection of NHP 14

No.	Locus tag	SignalP6 predicted(1)	Virulence(2)	Gene	Function	RPKMs in NHP 14(3)	RANK(4)
1	MGCS36089_00482			*ssrS*	6S RNA	4576592	1
2	MGCS36089_02374			*ssrA*	transfer-messenger RNA, SsrA	378078	2
3	MGCS36089_03788			*rpsN2*	30S ribosomal S14-2 protein RpsN2	231769	3
4	MGCS36089_01500		Virulence	*sagA_RNA*	sagA noncoding RNA	201461	4
5	MGCS36089_03346			-	RNaseP_bact_b RNA	193399	5
6	MGCS36089_02048	Lipo(5)		-	TlpA-family protein	123562	6
7	MGCS36089_02044	Secreted	Virulence	*isp*	secreted CHAP domain-containing immunogenic	97636	7
8	MGCS36089_01342			*tufA*	translation elongation factor Tu protein TufA	97314	8
9	MGCS36089_02046			-	hypothetical protein	83948	9
10	MGCS36089_02208			-	apolipoprotein A1/A4/E family protein	76637	10
11	MGCS36089_00766			-	putative metal homeostasis protein	71895	11
12	MGCS36089_00484			*rarA*	replication-associated recombination protein A	66157	12
13	MGCS36089_02682			*dltC*	D-alanine--poly(phosphoribitol) ligase subunit	64722	13
14	MGCS36089_03820		Virulence	*gapA*	glyceraldehyde-3-phosphate dehydrogenase GapA	59301	14
15	MGCS36089_02038			-	SalY superfamily ABC transporter permease	55950	15
16	MGCS36089_01510		Virulence	*sagF*	streptolysin S biosynthesis protein SagF	54926	16
17	MGCS36089_01498		Virulence	*sagA*	streptolysin S precursor SagA	46725	17
18	MGCS36089_02040		Virulence	*irr*	TCS(6) signal transduction DNA-binding response	46634	18
19	MGCS36089_02042		Virulence	*ihk*	TCS signal transduction histidine kinase sensor	45490	19
20	MGCS36089_03558			*grpE*	heat shock protein/nucleotide exchange factor	44883	20
21	MGCS36089_03556			*dnaK*	molecular chaperone DnaK	44498	21
22	MGCS36089_01508		Virulence	*sagE*	streptolysin S self-immunity protein SagE	43118	22
23	MGCS36089_02336			*yeaQ*	GlsB/YeaQ/YmgE family stress response membrane	39401	23
24	MGCS36089_01514		Virulence	*sagH*	streptolysin S export permease protein SagH	37680	24
25	MGCS36089_02680			*dltD*	D-alanyl-lipoteichoic acid biosynthesis protein	36824	25
26	MGCS36089_03526			*accD*	acetyl-CoA carboxylase carboxyl transferase	33294	26
27	MGCS36089_01512		Virulence	*sagG*	streptolysin S export protein SagG	32916	27
28	MGCS36089_03560			*hrcA*	heat-inducible transcriptional repressor HrcA	31626	28
29	MGCS36089_02684			*dltB*	D-alanyl-lipoteichoic acid biosynthesis protein	30495	29
30	MGCS36089_02036			-	LolD superfamily ABC transporter ATP-binding	30165	30

No.	Locus tag	SignalP6 predicted[1]	Virulence[2]	Gene	Function	RPKMs in NHP 14[3]	RANK[4]
31	MGCS36089_03006			-	DNA-binding protein HU	30076	31
32	MGCS36089_03530			*accC*	acetyl-CoA carboxylase biotin carboxylase	29203	32
33	MGCS36089_03736	Secreted		-	putative secreted protein	28939	33
34	MGCS36089_02058			-	hypothetical protein	28472	34
35	MGCS36089_01052	Lipo		*mtsA*	metal ABC transporter substrate-binding	27650	35
36	MGCS36089_02034			*acrA*	AcrA superfamily multidrug efflux pump	27132	36
37	MGCS36089_01502		Virulence	*sagB*	streptolysin S biosynthesis protein SagB	26478	37
38	MGCS36089_02334			*amaP*	alkaline shock response membrane anchor protein	25863	38
39	MGCS36089_03158	Lipo		*tlpA*	TlpA family protein disulfide reductase	25836	39
40	MGCS36089_01516		Virulence	*sagI*	streptolysin S export permease protein SagI	25012	40
41	MGCS36089_02900			*ptsH*	PTS transporter phosphocarrier protein PtsH	24326	41
42	MGCS36089_00190			*rplX*	50S ribosomal L24 protein RplX	24104	42
43	MGCS36089_01494			*eno*	phosphopyruvate hydratase -- enolase protein	23537	43
44	MGCS36089_01478			*rplS*	50S ribosomal L19 protein RpsL	23287	44
45	MGCS36089_01506		Virulence	*sagD*	streptolysin S biosynthesis protein SagD	22948	45
46	MGCS36089_04034			*groES*	co-chaperone GroES	22812	46
47	MGCS36089_02032	Lipo		-	ABC transporter protein	22447	47
48	MGCS36089_03292			*raiA*	ribosome-associated translation inhibitor RaiA	22403	48
49	MGCS36089_02210			-	hypothetical protein	22315	49
50	MGCS36089_03532			*fabZ*	3-hydroxyacyl-ACP dehydratase FabZ	22277	50
51	MGCS36089_01496		Virulence	*srrG*	streptolysin S small regulatory RNA SrrG	22065	51
52	MGCS36089_02686			*dltA*	D-alanine--poly(phosphoribitol) ligase subunit	21645	52
53	MGCS36089_04118			*spxA_2*	transcriptional regulator SpxA	21558	53
54	MGCS36089_02212			-	CsbD family protein	21353	54
55	MGCS36089_02332			-	DUF2273 domain-containing protein	21226	55
56	MGCS36089_00194			*rpsZ*	type Z 30S ribosomal S14 protein RpsZ	20598	56
57	MGCS36089_03156			*msrA/B*	Peptide methionine sulfoxide reductase	20534	57
58	MGCS36089_00748			*lrgB*	antiholin-like protein LrgB	20316	58
59	MGCS36089_03822			*fusA*	FusA family elongation factor EF-G	19261	59
60	MGCS36089_02326			-	Asp23/Gls24 family envelope stress response	19127	60
61	MGCS36089_01704			-	KH domain-containing protein	18706	61
62	MGCS36089_01504		Virulence	*sagC*	streptolysin S biosynthesis protein SagC	18370	62

No.	Locus tag	SignalP6 predicted[1]	Virulence[2]	Gene	Function	RPKMs in NHP 14[3]	RANK[4]
63	MGCS36089_00186			*rpsQ*	30S ribosomal S17 protein RpsQ	18308	63
64	MGCS36089_00192			*rplE*	50S ribosomal L5 protein RplE	18038	64
65	MGCS36089_03628	Secreted	Virulence	*isp2*	Isp-related CHAP domain-containing immunogenic	18037	65
66	MGCS36089_01586			*rpsU*	30S ribosomal S21 protein RpsU	17970	66
67	MGCS36089_02330			-	Asp23/Gls24 family envelope stress response	17887	67
68	MGCS36089_00858			*lemA*	LemA family protein	17671	68
69	MGCS36089_04052			*ahpF*	alkyl hydroperoxide reductase F subunit AhpF	17647	69
70	MGCS36089_02118			-	SpF66_s RNA	16931	70
71	MGCS36089_00318			-	DUF1033 domain-containing protein	16513	71
72	MGCS36089_03534			*accB*	acetyl-CoA carboxylase biotin carboxyl carrier	16240	72
73	MGCS36089_01340			-	sigma factor regulator	16000	73
74	MGCS36089_03824			*rpsG*	30S ribosomal S7 protein RpsG	15888	74
75	MGCS36089_01438	Secreted		*adcA*	zinc ABC transporter secreted substrate-binding	15649	75
76	MGCS36089_03732			-	BaeS family TCS sensor histidine kinase	15487	76
77	MGCS36089_00896			*yccA*	YccA family protein	15436	77
78	MGCS36089_04032			*groEL*	chaperonin GroEL	15397	78
79	MGCS36089_03546			*fabH*	3-oxoacyl-[acyl-carrier-protein] synthase	15385	79
80	MGCS36089_03648			*nusB*	transcription termination protein NusB	15287	80
81	MGCS36089_03536			*fabF*	3-oxoacyl-[acyl-carrier-protein] synthase	15143	81
82	MGCS36089_03956		Virulence	*perR*	peroxide-responsive transcriptional repressor	15081	82
83	MGCS36089_01294			-	YtxH domain-containing protein	14961	83
84	MGCS36089_00172			*rplW*	50S ribosomal L23 protein RplW	14643	84
85	MGCS36089_00182			*rplP*	50S ribosomal L29 protein RplP	14419	85
86	MGCS36089_00450			-	hypothetical protein	14236	86
87	MGCS36089_00188			*rplN*	50S ribosomal L14 protein RplN	14174	87
88	MGCS36089_03408	Lipo		-	PepSY domain-containing lipoprotein	14137	88
89	MGCS36089_02012			*ldh*	L-lactate dehydrogenase Ldh	14057	89
90	MGCS36089_01458			*rpmE*	50S ribosomal L31 type B protein RpmE	13971	90
91	MGCS36089_03674			*trxA_2*	thioredoxin TrxA	13652	91
92	MGCS36089_00602			*rplM*	50S ribosomal L13 protein RplM	13639	92
93	MGCS36089_03540			*fabD*	malonyl CoA-acyl carrier protein transacylase	13353	93
94	MGCS36089_03348			*gpsB*	cell division regulator GpsB	13290	94

No.	Locus tag	SignalP6 predicted[1]	Virulence[2]	Gene	Function	RPKMs in NHP 14[3]	RANK[4]
95	MGCS36089_00198			*rplF*	50S ribosomal L6 protein RplF	13232	95
96	MGCS36089_02382			*rpsA*	30S ribosomal S1 protein RpsA	13186	96
97	MGCS36089_03548			*fabT*	transcriptional regulatory protein FabT	13150	97
98	MGCS36089_03528			*accA*	acetyl-CoA carboxylase, carboxyltransferase beta	13049	98
99	MGCS36089_04112			*ruvX*	Holliday junction resolvase RuvX	13032	99
100	MGCS36089_03356			*pepC*	aminopeptidase (A) PepC	12985	100
101	MGCS36089_01668			*prp*	ribosomal-processing cysteine protease Prp	12622	101
102	MGCS36089_01698			-	TVP38 superfamily protein	12495	102
103	MGCS36089_01020			*nrdE_1*	class 1b ribonucleoside-diphosphate reductase	12469	103
104	MGCS36089_03552			*dnaJ*	chaperone protein DnaJ	12469	103
105	MGCS36089_00604			*rpsI*	30S ribosomal S9 protein RpsI. 272472..308964. Region of difference 36089_ROD.3, putative MGE	12434	105
106	MGCS36089_01018			*nrdI_2*	ribonucleotide reductase assembly protein NrdI	12394	106
107	MGCS36089_00184			*rpmC*	50S ribosomal L16 protein RpmC	12388	107
108	MGCS36089_03160			*ccdA*	putative cytochrome c-type biogenesis protein	12386	108
109	MGCS36089_02852	Secreted	Virulence	*spg*	extracellular cell surface IgG-binding streptococcal protein (G) SpG. Cell-wall anchoring predicted sortase	12344	109
110	MGCS36089_00180			*rpsC*	30S ribosomal S3 protein RpsC	12254	110
111	MGCS36089_01588			*mscL*	large-conductance mechanosensitive channel	12240	111
112	MGCS36089_02688			*dltX*	teichoic acid D-Ala incorporation-associated	11995	112
113	MGCS36089_03542			*fabK*	enoyl-[acyl-carrier-protein] reductase protein	11917	113
114	MGCS36089_04104			*nrdD_2*	anaerobic ribonucleoside-triphosphate reductase	11887	114
115	MGCS36089_00516	Secreted	Virulence	*emm*	cell surface M protein Emm. Cell-wall anchoring predicted sortase	11857	115
116	MGCS36089_01702			*rpsP*	30S ribosomal S16 protein RpsP	11770	116
117	MGCS36089_03950			-	CHY zinc finger domain-containing protein	11704	117
118	MGCS36089_00196			*rpsH*	30S ribosomal S8 protein RpsH	11693	118
119	MGCS36089_02056			*htpA*	streptococcal histidine triad (HIT)	11654	119
120	MGCS36089_00562			*rpsO*	30S ribosomal S15 protein RpsO	11634	120
121	MGCS36089_01670			*rpmA*	50S ribosomal L27 protein RpmA	11572	121
122	MGCS36089_02206			-	LCB5 family diacylglycerol lipid kinase	11478	122
123	MGCS36089_00178			*rplV*	50S ribosomal L22 protein RplV	11473	123
124	MGCS36089_03048			*clpA*	ATP-dependent Clp protease ATP-binding subunit	11454	124
125	MGCS36089_01166			-	cell division protein FtsW-like protein	11402	125

No.	Locus tag	SignalP6 predicted(1)	Virulence(2)	Gene	Function	RPKMs in NHP 14(3)	RANK(4)
126	MGCS36089_03826			*rpsL*	30S ribosomal S12 protein RpsL	11331	126
127	MGCS36089_03312			*pknB*	Stk1 family PASTA domain-containing Ser/Thr	11275	127
128	MGCS36089_00200			*rplR*	50S ribosomal L18 protein RplR	11216	128
129	MGCS36089_00202			*rpsE*	30S ribosomal S5 protein RpsE	11018	129
130	MGCS36089_00208			*secY*	preprotein translocase subunit SecY	10946	130
131	MGCS36089_00170			*rplD*	50S ribosomal L4 protein RplD	10861	131
132	MGCS36089_01696			-	ABC transporter permease	10793	132
133	MGCS36089_01006			*lctO*	L-lactate oxidase LctO	10760	133
134	MGCS36089_03500			-	Cps2a family anionic cell wall polymer	10644	134
135	MGCS36089_00204			*rpmD*	50S ribosomal L30 protein RpmD	10550	135
136	MGCS36089_03650			-	Asp23/Gls24 family envelope stress response	10533	136
137	MGCS36089_04110			-	DUF1292 domain-containing protein	10464	137
138	MGCS36089_01338			-	RpoE superfamily DNA-directed RNA polymerase	10440	138
139	MGCS36089_04050			*ahpC*	alkyl hydroperoxide reductase C subunit AhpC	10303	139
140	MGCS36089_04076			*tsf*	translation elongation factor Tsf	10299	140
141	MGCS36089_00168			*rplC*	50S ribosomal L3 protein RplC	10155	141
142	MGCS36089_01066			*rplA*	50S ribosomal L1 protein RplA	10037	142
143	MGCS36089_02258			*panT*	pantothenic acid transporter PanT	10022	143
144	MGCS36089_01016			*nrdF_1*	ribonucleotide-diphosphate reductase subunit	9863	144
145	MGCS36089_00316			*rpoC*	DNA-directed RNA polymerase subunit beta' RpoC	9850	145
146	MGCS36089_03538			*fabG_2*	3-ketoacyl-(acyl-carrier-protein) reductase	9794	146
147	MGCS36089_04246			-	transglycosylase SLT domain-containing protein	9727	147
148	MGCS36089_01304			-	NAD(P)-binding domain-containing protein	9711	148
149	MGCS36089_02062			*dppD*	dipeptide ABC transport system ATP-binding	9520	149
150	MGCS36089_03634			*secA*	preprotein translocase subunit SecA	9397	150
151	MGCS36089_03948			*bioY*	biotin transporter BioY	9364	151
152	MGCS36089_03138			*arcB*	ornithine carbamoyltransferase ArcB	9333	152
153	MGCS36089_02560			-	hypothetical protein	9300	153
154	MGCS36089_01080	Secreted		*lysM*	LysM peptidoglycan-binding domain-containing	9265	154
155	MGCS36089_02992			*gpmA*	phosphoglycerate mutase GpmA	9220	155
156	MGCS36089_00158			-	hypothetical protein	9102	156
157	MGCS36089_00176			*rpsS*	30S ribosomal S19 protein RpsS	9031	157

No.	Locus tag	SignalP6 predicted[1]	Virulence[2]	Gene	Function	RPKMs in NHP 14[3]	RANK[4]
158	MGCS36089_01288			*hprK*	HPr(Ser) kinase/phosphatase HprK	8986	158
159	MGCS36089_03518			*manN*	PTS transporter mannose-specific IID component	8956	159
160	MGCS36089_00104	Secreted		*sibA*	CHAP domain-containing protein/secreted	8949	160
161	MGCS36089_02360			*pyk*	pyruvate kinase Pyk	8933	161
162	MGCS36089_03032			-	phospho-sugar mutase	8854	162
163	MGCS36089_01786			*clpL*	ATP-dependent Clp protease ATP-binding subunit	8758	163
164	MGCS36089_00860			*htpX*	zinc metalloprotease HtpX	8750	164
165	MGCS36089_04074			*rpsB*	30S ribosomal S2 protein RpsB	8609	165
166	MGCS36089_00174			*rplB*	50S ribosomal L2 protein RplB	8603	166
167	MGCS36089_01966			*eutD*	phosphate acetyltransferase EutD	8598	167
168	MGCS36089_00762			-	DAK2 domain-containing protein	8445	168
169	MGCS36089_01244			*ftsY*	signal recognition particle-docking protein	8351	169
170	MGCS36089_01692			-	RND family transporter membrane fusion protein	8333	170
171	MGCS36089_01260			-	SPJ_0845 family protein	8289	171
172	MGCS36089_03140			-	GNAT family N-acetyltransferase	8153	172
173	MGCS36089_02898			*ptsI*	phosphoenolpyruvate--protein phosphotransferase	7889	173
174	MGCS36089_03970			*ulaG*	L-ascorbate utilization protein (G) UlaG	7830	174
175	MGCS36089_03872			*jag*	RNA-binding protein Jag	7801	175
176	MGCS36089_04036			*clpC*	ATP-dependent Clp protease ATP-binding subunit	7664	176
177	MGCS36089_02878	Lipo	Virulence	*Slr*	InlA-like streptococcal leucine rich lipoprotein	7644	177
178	MGCS36089_03052			-	DUF1827 family protein	7639	178
179	MGCS36089_01064			*rplK*	50S ribosomal L11P protein RplK	7612	179
180	MGCS36089_01694			-	LolD superfamily ABC transporter ATPase	7584	180
181	MGCS36089_02562			*acoC*	dihydrolipoamide acetyltransferase AcoC	7558	181
182	MGCS36089_01286			-	PspC domain-containing protein	7497	182
183	MGCS36089_02362			*pfkA*	6-phosphofructokinase PfkA	7480	183
184	MGCS36089_01352			*tpiA*	triose-phosphate isomerase TpiA	7467	184
185	MGCS36089_04096			*nrdG*	anaerobic ribonucleoside-triphosphate reductase	7434	185
186	MGCS36089_03326			*rny*	ribonuclease (Y) Rny	7404	186
187	MGCS36089_02564			*acoB*	pyruvate dehydrogenase E1 component beta subunit	7380	187
188	MGCS36089_03376			*mraY*	phospho-N-acetylmuramoyl-pentapeptide-	7349	188
189	MGCS36089_02282		Virulence	*ciaH*	TCS sensor histidine kinase protein CiaH	7340	189

No.	Locus tag	SignalP6 predicted[(1)]	Virulence[(2)]	Gene	Function	RPKMs in NHP 14[(3)]	RANK[(4)]
190	MGCS36089_03734			-	OmpR family TCS DNA-binding response regulator	7338	190
191	MGCS36089_00994			*arsC_1*	arsenate reductase ArsC	7313	191
192	MGCS36089_02356			*glmS*	glutamine--fructose-6-phosphate transaminase	7265	192
193	MGCS36089_01648			-	YlbF/YmcA family competence regulator	7263	193
194	MGCS36089_01372			*mgtA*	MgtA superfamily cation-translocating P-type	7262	194
195	MGCS36089_03142			*arcA*	arginine deiminase ArcA	7242	195
196	MGCS36089_00154			*adhE*	bifunctional acetaldehyde-CoA/alcohol dehydrogenase AdhE	7240	196
197	MGCS36089_01290			*lgt*	prolipoprotein diacylglyceryl transferase Lgt	7215	197
198	MGCS36089_02328			-	CsbD family protein	7209	198
199	MGCS36089_01320			-	TPP RNA	7153	199
200	MGCS36089_04016			*secE*	preprotein translocase subunit protein SecE	7105	200
201	MGCS36089_01792			*rplJ*	50S ribosomal L10 protein RplJ	7066	201
202	MGCS36089_04290			*parB*	chromosome partitioning protein ParB	7065	202
203	MGCS36089_00220			*rpoA*	DNA-directed RNA polymerase subunit alpha RpoA	7036	203
204	MGCS36089_01666			*rplU*	50S ribosomal L21 protein RplU	7031	204
205	MGCS36089_02204			*yqfA*	membrane channel forming/hemolysin III protein	7026	205
206	MGCS36089_00494			*rsmE*	16S rRNA (uracil(1498)-N(3))-methyltransferase	7014	206
207	MGCS36089_03562			-	FlgJ-related putative peptidoglycan hydrolase	6994	207
208	MGCS36089_02262			-	putative nucleoside ABC transporter permease	6987	208
209	MGCS36089_03828	Secreted		*prgA*	surface exclusion domain-containing secreted	6944	209
210	MGCS36089_03474			*rbfA*	30S ribosome-binding factor RbfA	6927	210
211	MGCS36089_03150			-	MmcQ/YjbR family DNA-binding protein	6867	211
212	MGCS36089_00306			*adcB*	metal ABC transporter permease AdcB	6850	212
213	MGCS36089_03314			*pppL*	Stp1/IreP family PP2C-type Ser/Thr phosphatase	6847	213
214	MGCS36089_02132			-	FAD-binding oxidoreductase	6712	214
215	MGCS36089_03342			*mapZ*	MapZ family cell division site-positioning	6659	215
216	MGCS36089_00218			*rpsK*	30S ribosomal S11 protein RpsK	6655	216
217	MGCS36089_01004			*lctP*	L-lactate permease LctP	6648	217
218	MGCS36089_01536			*glgA*	glycogen synthase GlgA	6640	218
219	MGCS36089_04158			*rpmGA*	50S ribosomal L33 protein RpmGA. 2088217..2104677. Region of difference 36089_ROD.9, putative MGE	6629	219
220	MGCS36089_00864		Virulence	*covR*	TCS DNA-binding response regulator CovR	6613	220
221	MGCS36089_03544			*acpP_2*	acyl carrier protein AcpP	6612	221

No.	Locus tag	SignalP6 predicted[(1)]	Virulence[(2)]	Gene	Function	RPKMs in NHP 14[(3)]	RANK[(4)]
222	MGCS36089_01722			*fruA*	fructose-specific PTS transporter EIIC component	6592	222
223	MGCS36089_02558			*acoL*	dihydrolipoyl dehydrogenase AcoL	6591	223
224	MGCS36089_02920	Lipo		*prsA*	peptidylprolyl isomerase lipoprotein PrsA	6570	224
225	MGCS36089_01156			-	DUF853 domain-containing protein	6561	225
226	MGCS36089_00736			*tig*	trigger factor molecular chaperone Tig	6549	226
227	MGCS36089_03388			-	hypothetical protein	6527	227
228	MGCS36089_00760			-	Asp23/Gls24 family envelope stress response	6490	228
229	MGCS36089_01318			*thiT*	energy-coupled thiamine transporter ThiT	6490	228
230	MGCS36089_00304			*adcC*	metal ABC transporter ATP-binding protein AdcC	6486	230
231	MGCS36089_03912			*galU*	UTP--glucose-1-phosphate uridylyltransferase	6478	231
232	MGCS36089_03564			-	LD/DD carboxypeptidase family protein	6464	232
233	MGCS36089_03322			*rpoZ*	DNA-directed RNA polymerase omega subunit RpoZ	6434	233
234	MGCS36089_03046			-	DUF1797 family protein	6399	234
235	MGCS36089_00336			*ackA*	acetate kinase AckA	6389	235
236	MGCS36089_03986			*leuS*	leucine--tRNA synthase LeuS	6352	236
237	MGCS36089_00030			*ftsH*	ATP-dependent zinc metalloprotease FtsH	6327	237
238	MGCS36089_02054	Lipo	Virulence	*lmb*	bifunctional metal ABC transporter substrate-binding lipoprotein/laminin-binding lipoprotein Lmb	6318	238
239	MGCS36089_02426			*deoD*	DeoD-type purine-nucleoside phosphorylase	6313	239
240	MGCS36089_03958			*yccU*	YccU family CoA-binding protein	6312	240
241	MGCS36089_01706	Secreted		*sdrD*	SdrD superfamily cell surface extracellular. CW-Pred predicted sortase. Cell-wall anchoring	6305	241
242	MGCS36089_02154	Lipo		-	FMN-binding protein, major membrane immunogen	6302	242
243	MGCS36089_03394			*tkt*	transketolase Tkt	6292	243
244	MGCS36089_00954			*clpP*	ATP-dependent Clp protease proteolytic subunit	6279	244
245	MGCS36089_03502			-	GNAT family N-acetyltransferase	6268	245
246	MGCS36089_00894			*rnaY*	RnaY family HD domain-containing protein	6257	246
247	MGCS36089_04288		Virulence	*htrA*	trypsin-like serine protease HtrA	6256	247
248	MGCS36089_02610			*rfbC*	dTDP-4-dehydrorhamnose 3,5-epimerase RfbC	6188	248
249	MGCS36089_04268			*guaB*	IMP dehydrogenase GuaB	6182	249
250	MGCS36089_00852			*ktrA*	potassium uptake transporter gating subunit	6164	250
251	MGCS36089_01150			*coaE*	dephospho-CoA kinase CoaE	6118	251
252	MGCS36089_01550			*atpD*	ATP synthase beta subunit AtpD	6082	252

No.	Locus tag	SignalP6 predicted(1)	Virulence(2)	Gene	Function	RPKMs in NHP 14(3)	RANK(4)
253	MGCS36089_01292			-	COG4768 superfamily YoxC-like protein	6059	253
254	MGCS36089_03066			*yggS*	YggS family pyridoxal phosphate-dependent	6026	254
255	MGCS36089_01628			-	LysM peptidoglycan-binding domain-containing	6020	255
256	MGCS36089_02284		Virulence	*ciaR*	TCS DNA-binding response regulator protein CiaR	6000	256
257	MGCS36089_01650			*gorA*	glutathione reductase GorA	5978	257
258	MGCS36089_03476			*infB*	translation initiation factor IF-2	5948	258
259	MGCS36089_02286			*pepN*	lysyl aminopeptidase/alanine aminopeptidase	5936	259
260	MGCS36089_03798			*rnjA_2*	mRNA degradation ribonuclease RnjA	5894	260
261	MGCS36089_03060			-	RNA-binding protein	5880	261
262	MGCS36089_02064			*dppC*	dipeptide ABC transport system permease protein	5875	262
263	MGCS36089_02256			*coaC*	phosphopantothenoylcysteine decarboxylase CoaC	5842	263
264	MGCS36089_00496			-	LacI family DNA-binding transcriptional	5835	264
265	MGCS36089_02060			*dppF*	dipeptide ABC transport system ATP-binding	5800	265
266	MGCS36089_00952			*upp*	uracil phosphoribosyltransferase Upp	5794	266
267	MGCS36089_00166			*rpsJ*	30S ribosomal S10 protein RpsJ	5789	267
268	MGCS36089_00888	Lipo		*yidC_1*	membrane protein insertase lipoprotein YidC	5769	268
269	MGCS36089_00302			*adcR*	zinc-dependent MarR family transcriptional	5726	269
270	MGCS36089_03652			*efp*	translation elongation factor (P) Efp	5713	270
271	MGCS36089_03110	Lipo		*rbsB*	D-ribose ABC transporter substrate-binding	5709	271
272	MGCS36089_03554			-	PfpI family predicted protease/amidase	5698	272
273	MGCS36089_03304			-	putative bifunctional	5691	273
274	MGCS36089_02542			*glmM*	phosphoglucosamine mutase GlmM	5673	274
275	MGCS36089_02730			-	DUF1846 domain-containing protein	5643	275
276	MGCS36089_02008			*fadH2*	FadH2 superfamily FAD-dependent oxidoreductase	5633	276
277	MGCS36089_00794	Lipo		*oppA_1*	oligopeptide ABC transporter substrate-binding	5619	277
278	MGCS36089_01054			*mtsB*	metal ABC transporter ATP-binding protein MtsB	5600	278
279	MGCS36089_02716			*obgE*	GTPase ObgE	5571	279
280	MGCS36089_01716			*apbA*	2-dehydropantoate 2-reductase	5563	280
281	MGCS36089_02428			*xapA*	XapA family purine-nucleoside phosphorylase	5548	281
282	MGCS36089_00850			*sstT*	serine/threonine transporter SstT	5542	282
283	MGCS36089_03816			*pgk*	phosphoglycerate kinase Pgk	5542	282
284	MGCS36089_01534			*glgD*	glucose-1-phosphate adenylyltransferase subunit	5517	284

No.	Locus tag	SignalP6 predicted[1]	Virulence[2]	Gene	Function	RPKMs in NHP 14[3]	RANK[4]
285	MGCS36089_03960			-	hypothetical protein	5511	285
286	MGCS36089_00754			*fba_2*	fructose-bisphosphate aldolase	5494	286
287	MGCS36089_01046			*mtnN*	5'-methylthioadenosine/adenosylhomocysteine	5482	287
288	MGCS36089_02068	Lipo		*dppA*	dipeptide-binding ABC transport system	5481	288
289	MGCS36089_03478			-	YlxQ-related RNA-binding protein	5459	289
290	MGCS36089_04216			*rpsD*	30S ribosomal S4 protein RpsD	5453	290
291	MGCS36089_03090			*dps*	DNA-binding starvation protein	5448	291
292	MGCS36089_00898			*yneF*	YneF family protein	5426	292
293	MGCS36089_01796			-	rli38. . 870743..921250. Region of difference 36089_ROD.4, putative MGE	5409	293
294	MGCS36089_00834			-	YebC/PmpR family DNA-binding transcriptional	5388	294
295	MGCS36089_03968			-	diacylglycerol kinase family lipid kinase	5328	295
296	MGCS36089_02264			-	putative nucleoside ABC transporter permease	5293	296
297	MGCS36089_02306			*spxA_1*	transcriptional regulator SpxA	5286	297
298	MGCS36089_01542			*atpF*	ATP synthase B subunit AtpF	5272	298
299	MGCS36089_02608			*rfbB*	dTDP-glucose 4,6-dehydratase RfbB	5262	299
300	MGCS36089_01996			-	GNAT family N-acetyltransferase	5251	300
301	MGCS36089_03726	Lipo		-	chromosome assembly-related lipoprotein	5244	301
302	MGCS36089_03374			*cshB*	DEAD/DEAH box helicase	5240	302
303	MGCS36089_01794			*rplL*	50S ribosomal L7/L12 protein RplL	5236	303
304	MGCS36089_03148			-	B3/4 domain-containing protein	5233	304
305	MGCS36089_03604			-	aldo/keto reductase	5188	305
306	MGCS36089_03608			*cbiO*	cobalt ECF transporter (A) ATPase component	5177	306
307	MGCS36089_03690			-	hypothetical protein	5171	307
308	MGCS36089_00414			*pgpA*	phosphatidylglycerophosphatase protein PgpA	5162	308
309	MGCS36089_02370			-	ABC transporter permease	5150	309
310	MGCS36089_03390			*ccmA*	CcmA family multidrug ABC transporter ATPase	5149	310
311	MGCS36089_00764			-	SPFH domain-containing protein	5122	311
312	MGCS36089_02430			*arsC_2*	arsenate reductase ArsC	5119	312
313	MGCS36089_03598			*uspA*	UspA family nucleotide-binding universal stress	5105	313
314	MGCS36089_04098			-	putative acetyltransferase	5100	314
315	MGCS36089_01744			*fms*	peptide deformylase	5085	315
316	MGCS36089_03028			-	PfkB superfamily kinase	5074	316

No.	Locus tag	SignalP6 predicted[(1)]	Virulence[(2)]	Gene	Function	RPKMs in NHP 14[(3)]	RANK[(4)]
317	MGCS36089_00312			-	Lacto-rpoB RNA	5071	317
318	MGCS36089_00390			*purA*	adenylosuccinate synthase PurA	5055	318
319	MGCS36089_00800			*oppD_1*	oligopeptide ABC transporter permease protein	5050	319
320	MGCS36089_03378			*pbp2X*	PG transpeptidase class B penicillin-binding	5028	320
321	MGCS36089_03010			-	SGNH-hydrolase superfamily of lipases and	4987	321
322	MGCS36089_00576			*cysE*	serine O-acetyltransferase CysE	4983	322
323	MGCS36089_03630			*alr*	alanine racemase Alr	4960	323
324	MGCS36089_03716			*glpF_2*	glycerol uptake facilitator GlpF	4938	324
325	MGCS36089_03068			*ftsZ*	cell division protein FtsZ	4912	325
326	MGCS36089_03134			*arcT*	M20 family metallodipeptidase ArcT	4910	326
327	MGCS36089_04100			*mviM*	MviM family predicted dehydrogenase	4892	327
328	MGCS36089_01546			*atpA*	ATP synthase alpha chain, AtpA	4878	328
329	MGCS36089_04266			*glcU*	glucose uptake permease GlcU	4871	329
330	MGCS36089_02894			-	PgdA-like putative PG GlcNAc deacetylase	4861	330
331	MGCS36089_02268	Lipo		-	putative nucleoside ABC transporter	4839	331
332	MGCS36089_03668			*rpsF*	30S ribosomal S6 protein RpsF	4834	332
333	MGCS36089_03430			-	Cof-type HAD-IIB family phosphohydrolase	4831	333
334	MGCS36089_03572			*gatB_2*	aspartyl-tRNA(Asn) or glutamyl-tRNA(Gln)	4820	334
335	MGCS36089_02368			-	ABC transporter ATP-binding protein	4815	335
336	MGCS36089_03840			*rsgA*	ribosome small subunit-dependent GTPase (A)	4815	335
337	MGCS36089_01426			*asnC*	asparaginyl-tRNA synthetase protein AsnC	4792	337
338	MGCS36089_00936			*ppaC*	manganese-dependent inorganic pyrophosphatase	4780	338
339	MGCS36089_03932			*pgi*	Pgi family glucose-6-phosphate isomerase	4748	339
340	MGCS36089_03354			*pbp1A*	bifunctional PG transglycosylase-transpeptidase	4747	340
341	MGCS36089_00990			-	GNAT family N-acetyltransferase	4735	341
342	MGCS36089_03324			*gmk*	guanylate kinase Gmk	4734	342
343	MGCS36089_01676			*rluD*	ribosomal large subunit pseudouridine synthase	4732	343
344	MGCS36089_04018			*pbp2A*	multimodular transpeptidase-transglycosylase	4711	344
345	MGCS36089_01552			*atpC*	ATP synthase epsilon subunit AtpC	4705	345
346	MGCS36089_00152			*oatA*	acetyltransferase OatA	4673	346
347	MGCS36089_00958			-	YlbF family regulatory protein	4667	347
348	MGCS36089_03596			*alaT*	AlaT family aminotransferase	4660	348

No.	Locus tag	SignalP6 predicted(1)	Virulence(2)	Gene	Function	RPKMs in NHP 14(3)	RANK(4)
349	MGCS36089_03886			*gltX*	glutamate--tRNA ligase	4648	349
350	MGCS36089_00122			*purN*	phosphoribosylglycinamide formyltransferase	4637	350
351	MGCS36089_03694			*pflB*	formate C-acetyltransferase	4622	351
352	MGCS36089_02550			-	CobQ-like type 1 glutamine amidotransferase	4583	352
353	MGCS36089_03152		Virulence	*yesM*	TCS sensor kinase YesM	4560	353
354	MGCS36089_03516			*manM*	PTS transporter mannose-specific IIC component	4527	354
355	MGCS36089_02400			*pyrC*	dihydroorotase PyrC	4513	355
356	MGCS36089_03550			*phaB*	enoyl-CoA hydratase protein PhaB	4503	356
357	MGCS36089_04114			-	IreB-related regulatory phosphoprotein	4484	357
358	MGCS36089_03016			*recN*	DNA repair protein RecN	4461	358
359	MGCS36089_02224			*nrdD_1*	ATP cone domain-containing protein,	4459	359
360	MGCS36089_03720			-	pyridoxamine 5'-phosphate oxidase family	4456	360
361	MGCS36089_01308			*lysS*	lysyl-tRNA synthetase LysS	4448	361
362	MGCS36089_00224			-	L17DE RNA	4435	362
363	MGCS36089_00528			*msmK*	sn-glycerol-3-phosphate ABC transporter	4418	363
364	MGCS36089_00522			*dtd*	D-tyrosyl-tRNA deacylase Dtd	4410	364
365	MGCS36089_00032			*plaP*	amino acid permease PlaP	4407	365
366	MGCS36089_00992			*ogt*	O6-methylguanine-DNA--protein-cysteine	4392	366
367	MGCS36089_03606			*recG*	ATP-dependent DNA helicase RecG	4390	367
368	MGCS36089_00982			*rsmI*	16S rRNA (cytidine(1402)-2'-O)-methyltransferase	4351	368
369	MGCS36089_02120			*guaA*	glutamine-hydrolyzing GMP synthase	4337	369
370	MGCS36089_01206			*gla*	glycerol uptake facilitator protein Gla	4326	370
371	MGCS36089_00150			-	hypothetical protein	4324	371
372	MGCS36089_03114			*rbsA*	D-ribose transporter ATPase RbsA	4296	372
373	MGCS36089_02002			-	lysozyme family protein	4280	373
374	MGCS36089_01720			*fruK*	1-phosphofructokinase FruK	4279	374
375	MGCS36089_03666			*ssb_2*	single-stranded DNA-binding protein	4276	375
376	MGCS36089_01284			-	SprT family protein	4273	376
377	MGCS36089_01746	Secreted		-	extracellular cell wall anchored bifunctional. Cell-wall anchoring predicted sortase	4270	377
378	MGCS36089_00582			*mrnC*	mini-ribonuclease 3 MrnC	4266	378
379	MGCS36089_00148			-	low molecular weight phosphotyrosine protein	4265	379
380	MGCS36089_01148			*mutM*	DNA-formamidopyrimidine glycosylase MutM	4258	380

No.	Locus tag	SignalP6 predicted[1]	Virulence[2]	Gene	Function	RPKMs in NHP 14[3]	RANK[4]
381	MGCS36089_03870			*rpmH*	50S ribosomal L34 protein RpmH	4229	381
382	MGCS36089_01544			*atpH*	ATP synthase delta subunit AtpH	4215	382
383	MGCS36089_02720			*pepS*	aminopeptidase PepS	4212	383
384	MGCS36089_01994			-	Sua5/YciO/YrdC/YwlC family protein ribosome	4210	384
385	MGCS36089_03676			-	FAD-containing oxidoreductase	4210	384
386	MGCS36089_01922			*potB*	spermidine putrescine ABC transport system	4195	386
387	MGCS36089_00222			*rplQ*	50S ribosomal L17 protein RplQ	4191	387
388	MGCS36089_01414			*prfB*	peptide chain release factor 2 PrfB	4178	388
389	MGCS36089_04142			*argS*	arginine--tRNA synthase ArgS	4171	389
390	MGCS36089_01548			*atpG*	ATP synthase gamma subunit AtpG	4165	390
391	MGCS36089_04274			*uup*	Uup family ATPase components of ABC transporters	4155	391
392	MGCS36089_02468			*manY*	ManY family PTS mannose/fructose IIC component	4147	392
393	MGCS36089_02466			*manZ*	ManZ family PTS mannose/fructose IID component	4128	393
394	MGCS36089_02270			*cdd*	cytidine deaminase Cdd	4106	394
395	MGCS36089_01636			*rpmI*	50S ribosomal L35 protein RpmL	4104	395
396	MGCS36089_02266			-	putative nucleoside ABC transporter ATP-binding	4084	396
397	MGCS36089_01418			*ftsX*	cell division permease-like protein FtsX	4076	397
398	MGCS36089_00120			*purM*	phosphoribosylformylglycinamidine cyclo-ligase	4075	398
399	MGCS36089_03818	Lipo		*lppC*	e(P4) family 5'-nucleotidase lipoprotein	4051	399
400	MGCS36089_03364			*trxB_2*	thioredoxin-disulfide reductase TrxB	4049	400
401	MGCS36089_02432			*deoB*	phosphopentomutase DeoB	4047	401
402	MGCS36089_00874			*der*	ribosome biogenesis GTPase Der	4041	402
403	MGCS36089_02846			*map*	methionyl aminopeptidase Map	4040	403
404	MGCS36089_02066			*dppB*	dipeptide ABC transport system permease protein	4017	404
405	MGCS36089_04122			*recA*	recombinase RecA	4007	405
406	MGCS36089_02366			*yhcF*	YhcF family transcriptional regulator	3994	406
407	MGCS36089_04252			*cbiO1*	cobalt ABC transporter ATPase CbiO2	3983	407
408	MGCS36089_02488			*ndk*	nucleoside-diphosphate kinase Ndk. 1212392..1212757. CRISPR with 6 repeat units	3969	408
409	MGCS36089_00492			*prmA*	50S ribosomal protein L11 methyltransferase	3962	409
410	MGCS36089_03432			-	YadS family trimeric intracellular cation	3955	410
411	MGCS36089_03838			*rpe*	ribulose-phosphate 3-epimerase Rpe	3934	411
412	MGCS36089_00300			*ipk*	4-diphosphocytidyl-2-C-methyl-D-erythritol	3933	412

No.	Locus tag	SignalP6 predicted(1)	Virulence(2)	Gene	Function	RPKMs in NHP 14(3)	RANK(4)
413	MGCS36089_01998			*glyA*	serine hydroxymethyl transferase GlyA	3905	413
414	MGCS36089_02124			*mngR*	MngR family DNA-binding transcriptional	3900	414
415	MGCS36089_01186			-	glycosyltransferase	3899	415
416	MGCS36089_02774			-	helix-turn-helix domain-containing putative	3897	416
417	MGCS36089_00394			*pflC*	pyruvate formate-lyase activating enzyme PflC	3893	417
418	MGCS36089_00502			-	Udk family kinase	3885	418
419	MGCS36089_04042			*cspA*	cold-shock protein CspA	3874	419
420	MGCS36089_03592			-	cysteine hydrolase	3864	420
421	MGCS36089_01204			*dhaM*	PTS-dependent dihydroxyacetone kinase	3851	421
422	MGCS36089_01416			*ftsE*	cell division ATP-binding protein FtsE	3846	422
423	MGCS36089_03624	Secreted		*shp*	heme-binding secreted protein Shp. Cell-wall anchoring predicted sortase	3838	423
424	MGCS36089_02662	Lipo		*malE*	maltose/maltodextrin ABC transport system	3831	424
425	MGCS36089_02448			-	NAD-dependent succinate-semialdehyde	3819	425
426	MGCS36089_02882			*dnaX*	DNA polymerase III gamma/tau subunit DnaX	3818	426
427	MGCS36089_03236			*bglB_2*	6-phospho-beta-glucosidase BglB	3805	427
428	MGCS36089_03622	Lipo		*htsA*	heme ABC transporter substrate-binding	3787	428
429	MGCS36089_01226		Virulence	*vicK*	TCS signal transduction sensor kinase VicK	3770	429
430	MGCS36089_03154		Virulence	*yesN*	TCS DNA-binding response regulator YesN	3768	430
431	MGCS36089_02438			*pepV*	dipeptidase PepV	3743	431
432	MGCS36089_00214			*rpmJ*	50S ribosomal L36 protein RpmJ	3736	432
433	MGCS36089_02694			*glnQ_2*	glutamine transport ATP-binding protein GlnQ	3732	433
434	MGCS36089_02574			*rnjA_1*	mRNA degradation ribonuclease RnjA	3730	434
435	MGCS36089_01202			*dhaL*	dihydroxyacetone kinase subunit DhaL	3728	435
436	MGCS36089_00392	Lipo		-	BMP family ABC transporter substrate-binding	3716	436
437	MGCS36089_02424			-	LytR family transcriptional regulator	3714	437
438	MGCS36089_00776			*mecA*	negative regulator of genetic competence,	3711	438
439	MGCS36089_03830			*purR*	pur operon repressor PurR	3698	439
440	MGCS36089_02028	Secreted		-	DUF1002 domain-containing putative secreted	3696	440
441	MGCS36089_02552			*lplA_2*	lipoate--protein ligase LplA	3693	441
442	MGCS36089_03730			-	OmpR family TCS DNA-binding response regulator	3686	442
443	MGCS36089_00556			*def*	peptide deformylase Def	3685	443
444	MGCS36089_02288			*phoU_2*	phosphate signaling complex protein PhoU	3677	444

No.	Locus tag	SignalP6 predicted(1)	Virulence(2)	Gene	Function	RPKMs in NHP 14(3)	RANK(4)
445	MGCS36089_02598			-	tetra tricopeptide repeat family protein	3673	445
446	MGCS36089_03776			*ybaB*	YbaB family DNA-binding protein	3630	446
447	MGCS36089_03514			*manL*	PTS transporter mannose-specific IIB & IIA	3625	447
448	MGCS36089_03728			-	BaeS family TCS sensor histidine kinase	3624	448
449	MGCS36089_01700			-	glycerophosphodiester phosphodiesterase	3599	449
450	MGCS36089_02192			*topA*	type I DNA topoisomerase TopA	3590	450
451	MGCS36089_02892			*srmB*	SrmB superfamily II DNA and RNA helicase	3586	451
452	MGCS36089_03058			*divIVA*	cell division protein DivIVA	3551	452
453	MGCS36089_01042			*nudF*	NUDIX hydrolase NudF	3542	453
454	MGCS36089_00344			*proC*	pyrroline-5-carboxylate reductase ProC	3540	454
455	MGCS36089_04256			*rodZ*	cytoskeletal protein RodZ	3538	455
456	MGCS36089_03384			*proA*	glutamate-5-semialdehyde dehydrogenase ProA	3531	456
457	MGCS36089_02626			*apt*	adenine phosphoribosyltransferase Apt	3514	457
458	MGCS36089_01014			*metS*	methionine--tRNA synthase MetS	3508	458
459	MGCS36089_01184			-	Glycosyltransferase	3506	459
460	MGCS36089_03302			-	S1 RNA-binding domain-containing protein	3503	460
461	MGCS36089_04194			-	hypothetical protein	3502	461
462	MGCS36089_01530			*glgB*	1,4-alpha-glucan branching protein GlgB	3497	462
463	MGCS36089_02974			*prfC*	peptide chain release factor 3 PrfC	3491	463
464	MGCS36089_01424			*aspC*	aspartate aminotransferase protein AspC	3485	464
465	MGCS36089_02470			-	AgaB family mannose/fructose/N-acetylgalactosamine-specific component IIB	3477	465
466	MGCS36089_01032			-	DUF3977 family protein	3471	466
467	MGCS36089_00126			*purH*	bifunctional phosphoribosylaminoimidazolecarboxamide formyltransferase/IMP cyclohydrolase PurH	3461	467
468	MGCS36089_01638			*rplT*	50S ribosomal L20 protein RplT	3452	468
469	MGCS36089_00546			*proS*	prolyl-tRNA synthetase ProS	3447	469
470	MGCS36089_03910			*gpsA*	NAD(P)H-dependent glycerol-3-phosphate	3439	470
471	MGCS36089_02308			*ribF*	bifunctional riboflavin kinase/FAD synthetase	3437	471
472	MGCS36089_01782			*engB*	ribosome biogenesis GTP-binding protein EngB	3436	472
473	MGCS36089_00538			*yajC*	preprotein translocase subunit YajC	3430	473
474	MGCS36089_03264			-	DUF402 domain-containing protein	3430	473
475	MGCS36089_03368			-	GlnQ family polar amino acid ABC transporter	3424	475

No.	Locus tag	SignalP6 predicted(1)	Virulence(2)	Gene	Function	RPKMs in NHP 14(3)	RANK(4)
476	MGCS36089_02940			*nagB*	glucosamine-6-phosphate deaminase NagB	3418	476
477	MGCS36089_00988			*serC*	3-phosphoserine/phosphohydroxythreonine	3411	477
478	MGCS36089_03082			*typA*	translational GTPase TypA	3407	478
479	MGCS36089_01162			*rnr*	exoribonuclease (R) Rnr	3406	479
480	MGCS36089_00738			*rpoE*	DNA-directed RNA polymerase subunit delta RpoE	3400	480
481	MGCS36089_00822			*rsfS*	ribosome silencing factor RsfS	3400	480
482	MGCS36089_01784			-	hypothetical protein	3393	482
483	MGCS36089_00352			-	DUF4479 and tRNA-binding domain-containing	3382	483
484	MGCS36089_00346			*pepA*	glutamyl aminopeptidase PepA	3381	484
485	MGCS36089_02896			*gapN*	NADP-dependent glyceraldehyde-3-phosphate	3378	485
486	MGCS36089_03566			-	PhoE family broad specificity phosphatase	3378	485
487	MGCS36089_03832			*cbf*	YhaM family 3'-5' exoribonuclease	3359	487
488	MGCS36089_04156			*rpmF*	50S ribosomal L32 protein RpmF	3351	488
489	MGCS36089_01330			*btuE*	BtuE superfamily glutathione peroxidase	3341	489
490	MGCS36089_04244			-	HAD hydrolase-like protein	3336	490
491	MGCS36089_02340			-	Glycine RNA	3330	491
492	MGCS36089_03316			*rsmB*	16S rRNA (cytosine(967)-C(5))-methyltransferase	3326	492
493	MGCS36089_01608			*rgpF*	alpha-L-Rha alpha-1,3-L-rhamnosyltransferase	3324	493
494	MGCS36089_01022			*brnQ_2*	branched-chain amino acid transport system II	3318	494
495	MGCS36089_00554			-	NAD(P)H-dependent oxidoreductase	3316	495
496	MGCS36089_03062			-	YggT family protein	3310	496
497	MGCS36089_03112			*rbsC*	D-ribose ABC transporter permease RbsC	3291	497
498	MGCS36089_02280			*rpsT*	30S ribosomal S20 protein RpsT	3289	498
499	MGCS36089_02272			*deoC*	deoxyribose-phosphate aldolase DeoC	3281	499
500	MGCS36089_03600			-	HAD-related haloacid dehalogenase hydrolase	3280	500
501	MGCS36089_00028			-	hypoxanthine-guanine phosphoribosyltransferase	3275	501
502	MGCS36089_02544	Lipo		*ybbR*	YbbR family lipoprotein	3268	502
503	MGCS36089_03136			*arcD*	arginine/ornithine antiporter protein ArcD	3265	503
504	MGCS36089_00314			*rpoB*	DNA-directed RNA polymerase subunit beta RpoB	3262	504
505	MGCS36089_03480			-	YlxR family putative RNA-binding protein	3253	505
506	MGCS36089_02000			-	nucleoid-associated bacterial family protein	3242	506
507	MGCS36089_02600			*perM*	PerM family predicted purR regulated permease	3242	506

No.	Locus tag	SignalP6 predicted(1)	Virulence(2)	Gene	Function	RPKMs in NHP 14(3)	RANK(4)
508	MGCS36089_04094			*yaaA*	peroxide stress protein YaaA	3235	508
509	MGCS36089_03008			-	DUF2140 domain-containing protein	3232	509
510	MGCS36089_04198			-	DUF368 domain-containing protein	3228	510
511	MGCS36089_02566			*acoA*	Pyruvate dehydrogenase E1 component alpha	3218	511
512	MGCS36089_01214			-	DUF3114 domain-containing protein	3217	512
513	MGCS36089_03664			*rpsR*	30S ribosomal S18 protein RpsR	3215	513
514	MGCS36089_01528			*pulA_2*	type I pullulanase PulA	3203	514
515	MGCS36089_02420			-	hypothetical protein	3190	515
516	MGCS36089_03162			-	CorA family divalent cation transport protein	3189	516
517	MGCS36089_03396			*fsa*	FSA family fructose-6-phosphate aldolase	3189	516
518	MGCS36089_02612			*rfbA*	glucose-1-phosphate thymidylyltransferase RfbA	3184	518
519	MGCS36089_00550			*polC*	DNA polymerase III PolC	3172	519
520	MGCS36089_03116			*rbsD*	D-ribose pyranase RbsD	3169	520
521	MGCS36089_00592			-	DegV family protein	3168	521
522	MGCS36089_02142			-	cupin domain-containing protein	3165	522
523	MGCS36089_00206			*rplO*	50S ribosomal L15 protein RplO	3159	523
524	MGCS36089_03834			*rmuC*	DNA recombination protein RmuC	3155	524
525	MGCS36089_01044			*macP*	cell wall synthase accessory phosphoprotein	3151	525
526	MGCS36089_00106			*prs*	ribose-phosphate pyrophosphokinase PrsA	3150	526
527	MGCS36089_01616			-	glycosyltransferase family 1 protein	3148	527
528	MGCS36089_01178			*pepQ*	Xaa-Pro dipeptidase protein PepQ	3139	528
529	MGCS36089_03574			*gatA_2*	aspartyl-tRNA(Asn) or glutamyl-tRNA(Gln)	3130	529
530	MGCS36089_03610			*cbiQ_1*	cobalt ECF transporter (T) transmembrane	3105	530
531	MGCS36089_01034			-	ASCH domain-containing RNA-binding protein	3104	531
532	MGCS36089_01374			-	hypothetical protein	3102	532
533	MGCS36089_03482			*nusA*	transcription termination factor NusA	3089	533
534	MGCS36089_03452			*copA*	copper-exporting ATPase CopA	3078	534
535	MGCS36089_02876			*metK*	methionine adenosyltransferase MetK	3076	535
536	MGCS36089_02128			*ffh*	signal recognition particle protein	3073	536
537	MGCS36089_01170			*wcaA*	WcaA superfamily glycosyltransferase involved in	3069	537
538	MGCS36089_03952			*tgt*	tRNA guanosine(34) transglycosylase Tgt	3065	538
539	MGCS36089_00926			-	PgpB family membrane-associated phospholipid	3062	539

No.	Locus tag	SignalP6 predicted(1)	Virulence(2)	Gene	Function	RPKMs in NHP 14(3)	RANK(4)
540	MGCS36089_03962			*polA*	DNA polymerase I PolA	3053	540
541	MGCS36089_00292			*cydB*	cytochrome d ubiquinol oxidase subunit (II)	3050	541
542	MGCS36089_00544			*rseP*	RIP metalloprotease RseP	3040	542
543	MGCS36089_03012			-	DegV family EDD domain-containing protein	3029	543
544	MGCS36089_03070			*ftsA*	cell division protein FtsA	3019	544
545	MGCS36089_03308		Virulence	*liaS*	three component system signal transduction	3009	545
546	MGCS36089_00816			*nadD*	nicotinate-nucleotide adenylyltransferase NadD	3008	546
547	MGCS36089_02884			-	GAF domain containing protein	3005	547
548	MGCS36089_02916			*alaS*	alanine--tRNA synthetase AlaS	3002	548
549	MGCS36089_01224		Virulence	*vicR*	TCS DNA-binding response regulator VicR	2977	549
550	MGCS36089_01920			*potA*	spermidine putrescine ABC transport system	2976	550
551	MGCS36089_02172			-	PASTA domain-containing protein	2972	551
552	MGCS36089_01556			*murA_1*	UDP-N-acetylglucosamine 1-carboxyvinyltransferase protein MurA	2958	552
553	MGCS36089_02310			*truB*	tRNA pseudouridine(55) synthase TruB	2952	553
554	MGCS36089_00498			*ptsG*	PTS glucose-specific II ABC component	2940	554
555	MGCS36089_01610			-	glycosyltransferase family 2 protein	2938	555
556	MGCS36089_00410			*gldA*	glycerol dehydrogenase	2931	556
557	MGCS36089_02648			-	rhodanese-related sulfurtransferase	2931	556
558	MGCS36089_03030			*folD*	bifunctional methylenetetrahydrofolate dehydrogenase/methenyltetrahydrofolate cyclohydrolase FolD protein	2931	556
559	MGCS36089_01072			*frr*	ribosome recycling factor Frr	2928	559
560	MGCS36089_01232			*smc*	chromosome segregation protein Smc	2928	559
561	MGCS36089_00890			*acyP*	acylphosphatase AcyP	2925	561
562	MGCS36089_01596			*rmlD*	dTDP-4-dehydrorhamnose reductase protein RmlD	2922	562
563	MGCS36089_02906			*nrdF_2*	class 1b ribonucleoside-diphosphate reductase	2911	563
564	MGCS36089_03380			*ftsL*	cell division protein FtsL	2897	564
565	MGCS36089_00798			*oppC_1*	oligopeptide ABC transporter permease protein	2894	565
566	MGCS36089_00490			-	DUF3013 family protein	2885	566
567	MGCS36089_01632			-	L20_leader RNA	2881	567
568	MGCS36089_02290			*ptsB1*	phosphate ABC transporter ATP-binding protein	2876	568
569	MGCS36089_02532			-	TIGR01906 family membrane protein	2871	569
570	MGCS36089_03202			*aroB*	3-dehydroquinate synthase protein AroB	2868	570

No.	Locus tag	SignalP6 predicted[1]	Virulence[2]	Gene	Function	RPKMs in NHP 14[3]	RANK[4]
571	MGCS36089_01036			-	Gfo/Idh/MocA family oxidoreductase	2858	571
572	MGCS36089_02644			-	glutathione S-transferase N-terminal	2858	571
573	MGCS36089_02350			-	amino acid ABC transporter ATP-binding protein	2857	573
574	MGCS36089_04008	Secreted	Virulence	*slo*	secreted cholesterol-dependent cytolysin	2851	574
575	MGCS36089_01540			*atpB*	ATP synthase A subunit AtpB	2847	575
576	MGCS36089_01532			*glgC*	glucose-1-phosphate adenylyltransferase subunit	2845	576
577	MGCS36089_03594			*codY*	CodY family GTP-sensing pleiotropic	2830	577
578	MGCS36089_00140			*purB*	adenylosuccinate lyase PurB	2824	578
579	MGCS36089_01060			*ftsK*	cell division protein FtsK	2819	579
580	MGCS36089_03632			*acpS*	AcpS family provisional 4'-phosphopantetheinyl	2817	580
581	MGCS36089_01594			-	metal-sulfur cluster assembly factor	2809	581
582	MGCS36089_01174			*gloA*	lactoylglutathione lyase protein GloA	2796	582
583	MGCS36089_04102			-	hypothetical protein	2785	583
584	MGCS36089_03836			*thiN*	thiamine diphosphokinase ThiN	2779	584
585	MGCS36089_04070			*hutG*	formiminoglutamase HutG	2773	585
586	MGCS36089_02944			-	DUF3114 domain-containing protein	2770	586
587	MGCS36089_03412			*glyS*	glycine--tRNA ligase beta subunit GlyS	2769	587
588	MGCS36089_02294			*ptsA*	phosphate ABC transporter permease PstA	2764	588
589	MGCS36089_02972			-	Pleckstrin homology-like domain-containing	2758	589
590	MGCS36089_01778			-	hypothetical protein	2755	590
591	MGCS36089_04116			-	SSRC41 RNA	2754	591
592	MGCS36089_00310			*pbp1b*	bifunctional PG transglycosylase-transpeptidase,	2753	592
593	MGCS36089_01488			*ezrA*	cell division septation ring formation regulator	2751	593
594	MGCS36089_02434			*rpiA*	ribose-5-phosphate isomerase RpiA	2750	594
595	MGCS36089_01336			*ftsW*	cell division protein FtsW	2744	595
596	MGCS36089_01942			-	AI-2E family transporter	2742	596
597	MGCS36089_03056			*ileS*	isoleucine--tRNA synthetase IleS	2738	597
598	MGCS36089_00216			*rpsM*	30S ribosomal S13 protein RpsM	2737	598
599	MGCS36089_02860			*uxuA*	mannonate dehydratase	2735	599
600	MGCS36089_01578			*rexA*	ATP-dependent nuclease A subunit RexA	2728	600
601	MGCS36089_00486			*pabA*	aminodeoxychorismate/anthranilate synthase	2721	601
602	MGCS36089_01486			*gyrB*	DNA topoisomerase ATP-hydrolyzing B subunit	2716	602

No.	Locus tag	SignalP6 predicted(1)	Virulence(2)	Gene	Function	RPKMs in NHP 14(3)	RANK(4)
603	MGCS36089_03808	Lipo		*oppA_2*	oligopeptide ABC transporter substrate-binding	2713	603
604	MGCS36089_04078			*pepO*	endopeptidase PepO	2712	604
605	MGCS36089_01048		Virulence	*mtsR*	metal-dependent transcriptional regulator MtsR	2692	605
606	MGCS36089_01732			*degV_1*	DegV family protein	2686	606
607	MGCS36089_00280			-	XRE family ImmR-like transcriptional regulator	2679	607
608	MGCS36089_01192			-	putative ABC transporter permease component	2679	607
609	MGCS36089_01456			-	PTS sugar transport IIA subunit	2678	609
610	MGCS36089_03064			*sepF*	cell division protein SepF	2676	610
611	MGCS36089_01472			*fldA*	flavodoxin FldA	2669	611
612	MGCS36089_00866		Virulence	*covS*	TCS sensor kinase CovS	2664	612
613	MGCS36089_00750			-	alpha/beta hydrolase	2643	613
614	MGCS36089_03306		Virulence	*liaR*	three component system signal transduction	2610	614
615	MGCS36089_03488			*trmB*	tRNA (guanosine(46)-N7)-methyltransferase TrmB	2602	615
616	MGCS36089_04106			-	DUF2079 domain-containing protein	2601	616
617	MGCS36089_03358			*nadE*	ammonia-dependent NAD(+) synthetase NadE	2587	617
618	MGCS36089_02668			*malH*	LacI family transcriptional regulatory protein	2585	618
619	MGCS36089_03118			*rbsK*	ribokinase RbsK	2585	618
620	MGCS36089_01780			*clpX*	ATP-dependent Clp protease, ATP-binding subunit	2583	620
621	MGCS36089_00884			*mltG*	endolytic transglycosylase MltG	2580	621
622	MGCS36089_00872			*dnaI*	primosomal protein DnaI	2579	622
623	MGCS36089_02254			*coaB*	phosphopantothenate--cysteine ligase CoaB	2577	623
624	MGCS36089_03130			*arcC*	carbamate kinase ArcC	2570	624
625	MGCS36089_04014			*nusG*	transcription antitermination protein NusG	2564	625
626	MGCS36089_03678			*mutS2*	DNA mismatch repair endonuclease MutS2	2562	626
627	MGCS36089_02260			*pgmA*	phospho-sugar mutase PgmA	2558	627
628	MGCS36089_03360			*pncB*	nicotinate phosphoribosyltransferase PncB	2544	628
629	MGCS36089_02622			*nth*	endonuclease III Nth	2541	629
630	MGCS36089_01658			*thiI*	thiamine biosynthesis/tRNA modification protein	2537	630
631	MGCS36089_00802			*oppF_1*	oligopeptide ABC transporter ATP-binding protein	2533	631
632	MGCS36089_02180			-	DUF3307 domain-containing protein	2526	632
633	MGCS36089_03620			*htsB*	heme ABC transporter permease HtsB	2516	633
634	MGCS36089_02516			-	CRISPR-DR22 RNA	2512	634

No.	Locus tag	SignalP6 predicted(1)	Virulence(2)	Gene	Function	RPKMs in NHP 14(3)	RANK(4)
635	MGCS36089_01672			*lysR*	LysR family transcriptional regulator	2499	635
636	MGCS36089_01622			-	RfbX superfamily lipopolysaccharide biosynthesis	2498	636
637	MGCS36089_01526			*dagK*	diacylglycerol kinase family lipid kinase	2494	637
638	MGCS36089_00870			*dnaB*	replication initiation and membrane attachment	2491	638
639	MGCS36089_00740			*pyrG*	CTP synthase Pyg	2487	639
640	MGCS36089_00730			-	DUF436 family protein	2481	640
641	MGCS36089_00942			*fhuB*	iron ABC transporter permease FhuB	2481	640
642	MGCS36089_01620			*galE*	UDP-glucose 4-epimerase GalE	2465	642
643	MGCS36089_01164			*smpB*	SsrA(tmRNA)-binding protein SmpB	2463	643
644	MGCS36089_04242			*sdhA*	L-serine dehydratase alpha subunit SdhA	2458	644
645	MGCS36089_01592			*rpoD*	RNA polymerase sigma factor RpoD	2457	645
646	MGCS36089_03612			-	ECF transporter (S) specificity component	2456	646
647	MGCS36089_00520			*relA*	bifunctional (p)ppGpp synthase/hydrolase RelA	2447	647
648	MGCS36089_03426			*degV_2*	DegV family fatty acid-binding protein	2427	648
649	MGCS36089_00406			*pflD*	pyruvate formate-lyase protein PflD	2423	649
650	MGCS36089_01626			*ebsA*	EbsA family pore-forming protein	2416	650
651	MGCS36089_02910			-	CPBP family intramembrane metalloprotease	2407	651
652	MGCS36089_03894			-	AIM24 family protein	2399	652
653	MGCS36089_01674			*lspA*	lipoprotein signal peptidase II LspA	2392	653
654	MGCS36089_01640			*ltaS*	LTA synthase LtaS	2387	654
655	MGCS36089_00356			-	deoxyadenosine kinase	2385	655
656	MGCS36089_03942	Lipo		-	CYK3 family lipoprotein	2369	656
657	MGCS36089_01088			*ybeY*	rRNA maturation RNase YbeY	2362	657
658	MGCS36089_03050			-	NUDIX hydrolase	2356	658
659	MGCS36089_03344			*rlmL*	23S rRNA N2-methylase RlmL	2343	659
660	MGCS36089_02508			-	CRISPR-DR22 RNA	2342	660
661	MGCS36089_01082			*mycA*	oleate hydratase/myosin-crossreactive antigen	2336	661
662	MGCS36089_03684			*rnhC*	HIII ribonuclease RnhC	2334	662
663	MGCS36089_03696			-	ElaA-related predicted N-acetyltransferase	2325	663
664	MGCS36089_01736			-	Uup family ATPase components of ABC transporters	2322	664
665	MGCS36089_03782			-	DUF536 domain-containing protein	2304	665
666	MGCS36089_01180		Virulence	*ccpA*	catabolite control protein CcpA	2303	666

No.	Locus tag	SignalP6 predicted(1)	Virulence(2)	Gene	Function	RPKMs in NHP 14(3)	RANK(4)
667	MGCS36089_02016			*srtA*	class A sortase SrtA	2301	667
668	MGCS36089_03382			*mraW*	S-adenosyl-methyltransferase MraW	2301	667
669	MGCS36089_00960			-	YlbG family protein	2297	669
670	MGCS36089_03386			*proB*	glutamate 5-kinase ProB	2295	670
671	MGCS36089_01010			-	hypothetical protein	2293	671
672	MGCS36089_00668			-	hypothetical protein	2288	672
673	MGCS36089_03614			-	CydD-related ABC transporter ATPase/permease	2286	673
674	MGCS36089_01726	Lipo		-	putative peptidoglycan hydrolase lipoprotein	2280	674
675	MGCS36089_01760			-	isopentenyl-diphosphate delta-isomerase	2280	674
676	MGCS36089_03420			*nagA*	N-acetylglucosamine-6-phosphate deacetylase	2279	676
677	MGCS36089_00570			*talA*	transaldolase TalA	2275	677
678	MGCS36089_03918			-	5-formyltetrahydrofolate cyclo-ligase	2265	678
679	MGCS36089_02624			*dnaD*	DNA replication protein DnaD	2264	679
680	MGCS36089_01412			*queG*	epoxyqueuosine reductase QueG	2260	680
681	MGCS36089_00358			*dus*	tRNA-dihydrouridine synthase Dus	2256	681
682	MGCS36089_02658			*malR*	maltose operon transcriptional repressor MalR	2256	681
683	MGCS36089_00530			*dexB*	glucan 1,6-alpha-glucosidase DexB	2254	683
684	MGCS36089_01228			*vicX*	zinc-dependent hydrolase protein VicX	2251	684
685	MGCS36089_02514			-	CRISPR-DR22 RNA	2251	684
686	MGCS36089_03504			*tsaE*	tRNA(adenosine(37)-N6)-threonylcarbamoyltransferase TsaE	2251	684
687	MGCS36089_00844			*metN_1*	methionine ABC transporter ATP-binding protein	2245	687
688	MGCS36089_03454			*copY_2*	DNA-binding copper transport transcriptional	2242	688
689	MGCS36089_00572			*pnp*	polyribonucleotide nucleotidyltransferase Pnp	2212	689
690	MGCS36089_01990			*prfA*	peptide chain release factor 1 PrfA	2209	690
691	MGCS36089_03946			-	metallo-beta-lactamase superfamily protein	2203	691
692	MGCS36089_00290			*cydA*	cytochrome ubiquinol oxidase subunit (I) CydA	2195	692
693	MGCS36089_02740	Lipo		*nlpA*	MetQ/NlpA family ABC transporter	2194	693
694	MGCS36089_03498			-	chemotaxis protein	2192	694
695	MGCS36089_02690			*uvrB*	excinuclease ABC subunit UvrB	2180	695
696	MGCS36089_03686			*lepB_2*	signal peptidase I LepB	2176	696
697	MGCS36089_01758			*mvaK2*	mevalonate kinase MvaK2	2166	697
698	MGCS36089_00156			*adhP*	alcohol dehydrogenase AdhP	2163	698

No.	Locus tag	SignalP6 predicted[1]	Virulence[2]	Gene	Function	RPKMs in NHP 14[3]	RANK[4]
699	MGCS36089_00408			*mipB*	fructose-6-phosphate aldolase MipB	2163	698
700	MGCS36089_02296			*ptsC*	phosphate ABC transporter permease PstC	2163	698
701	MGCS36089_03180			*valS*	valine--tRNA synthetase ValS	2137	701
702	MGCS36089_02292			*ptsB2*	phosphate ABC transporter ATP-binding protein	2133	702
703	MGCS36089_00118			*purF*	amidophosphoribosyltransferase PurF	2131	703
704	MGCS36089_00746			*lrgA*	antiholin-like murein hydrolase modulator LrgA	2127	704
705	MGCS36089_01868			*copZ_1*	copper chaperone CopZ	2122	705
706	MGCS36089_00732			-	mechanosensitive ion channel family protein	2118	706
707	MGCS36089_01926			*potD*	spermidine putrescine ABC transport system	2118	706
708	MGCS36089_02022			*lmb_1*	bifunctional metal ABC transporter substrate-binding lipoprotein/laminin-binding lipoprotein Lmb	2115	708
709	MGCS36089_04180			*pezT*	zeta toxin family protein PezT	2111	709
710	MGCS36089_00024	Secreted		-	class A beta-lactamase-related serine hydrolase	2107	710
711	MGCS36089_02512			-	CRISPR-DR22 RNA	2104	711
712	MGCS36089_01924			*potC*	spermidine putrescine ABC transport system	2094	712
713	MGCS36089_00004			*dnaN*	DNA polymerase III subunit beta protein DnaN	2081	713
714	MGCS36089_00980			*yabA*	DNA replication initiation control protein YabA	2081	713
715	MGCS36089_04128			*tag*	DNA-3-methyladenine glycosylase Tag	2068	715
716	MGCS36089_03402			*glpF_1*	GlpF family glycerol uptake facilitator	2066	716
717	MGCS36089_03964			*proW*	proline/glycine betaine ABC transporter ATPase	2065	717
718	MGCS36089_03626	Secreted	Virulence	*shr*	heme-binding secreted protein Shr. Cell-wall anchoring predicted sortase	2057	718
719	MGCS36089_00932		Virulence	*hlyX*	hemolysin family protein HylX	2056	719
720	MGCS36089_02352			-	amino acid ABC transporter permease	2056	719
721	MGCS36089_00350			*trxA_1*	thioredoxin family protein TrxA-like protein	2048	721
722	MGCS36089_02298	Lipo		*ptsS*	phosphate ABC transporter substrate-binding protein	2047	722
723	MGCS36089_01664			-	L21_leader RNA	2046	723
724	MGCS36089_02004			-	MdlB superfamily multidrug ABC transporter	2046	723
725	MGCS36089_02392			*parC*	DNA topoisomerase IV subunit A ParC	2040	725
726	MGCS36089_00552			-	MarR family transcriptional regulator	2038	726
727	MGCS36089_00948			*murE_1*	UDP-N-acetylmuramoyl-L-alanyl-D-glutamate--L- lysine ligase MurE	2029	727
728	MGCS36089_03890			-	hypothetical protein	2029	727
729	MGCS36089_00380			-	PAS domain-containing protein	2028	729

No.	Locus tag	SignalP6 predicted(1)	Virulence(2)	Gene	Function	RPKMs in NHP 14(3)	RANK(4)
730	MGCS36089_03074			*murG*	UDP-N-acetylglucosamine--N-acetylmuramyl- (pentapeptide) pyrophosphoryl-undecaprenol N-acetylglucosamine transferase MurG	2028	729
731	MGCS36089_02904			*nrdE_2*	class 1b ribonucleoside-diphosphate reductase	2023	731
732	MGCS36089_02348	Lipo		-	amino acid ABC transporter substrate-binding protein	2015	732
733	MGCS36089_02844			*brkB*	BrkB family protein	2015	732
734	MGCS36089_00984			-	DUF5684 domain-containing protein	2014	734
735	MGCS36089_03418			-	ARA1 family aldo/keto reductase	2003	735
736	MGCS36089_00342			-	hypothetical protein	1992	736
737	MGCS36089_00848			*brnQ_1*	branched-chain amino acid transport system II	1984	737
738	MGCS36089_03954			-	DUF975 family protein	1983	738
739	MGCS36089_01492			-	DUF1694 domain-containing protein	1982	739
740	MGCS36089_04020			-	translation initiation inhibitor	1981	740
741	MGCS36089_03120			*rbsR*	ribose transport operon repressor RbsR	1978	741
742	MGCS36089_02932			*pepF_2*	oligoendopeptidase (F) PepF	1972	742
743	MGCS36089_03874	Lipo		*yidC_2*	YidC/Oxa1 family membrane protein insertase	1965	743
744	MGCS36089_01630			*cmk*	CMP kinase Cmk	1964	744
745	MGCS36089_04254			*pgsA*	CDP-diacylglycerol--glycerol-3-phosphate	1960	745
746	MGCS36089_00578			-	hypothetical protein	1958	746
747	MGCS36089_03802			*oppD_2*	oligopeptide ABC transporter ATPase OppD	1948	747
748	MGCS36089_02838			-	LCP family anionic cell polymer synthesis	1940	748
749	MGCS36089_01460			-	IS30 family transposase	1936	749
750	MGCS36089_01992			*prmC*	peptide chain release factor N(5)-glutamine	1936	749
751	MGCS36089_02948			*sodA*	superoxide dismutase SodA	1920	751
752	MGCS36089_00846			*metP_1*	methionine ABC transporter permease MetP	1917	752
753	MGCS36089_01194			-	putative ABC transporter permease component	1917	752
754	MGCS36089_01176			-	NAD(P)H-dependent oxidoreductase	1914	754
755	MGCS36089_01652			-	dihydrofolate synthase	1913	755
756	MGCS36089_01328	Lipo		-	DUF4430 domain-containing lipoprotein	1909	756
757	MGCS36089_00976			*holB*	DNA polymerase III subunit delta' HolB	1908	757
758	MGCS36089_03738			-	GNAT family acetyltransferase	1906	758
759	MGCS36089_00288			*nox*	NAD(P)/FAD-dependent oxidoreductase Nox	1903	759
760	MGCS36089_00768			-	ABC amino acid transporter ATP-binding protein	1895	760

No.	Locus tag	SignalP6 predicted[(1)]	Virulence[(2)]	Gene	Function	RPKMs in NHP 14[(3)]	RANK[(4)]
761	MGCS36089_01606			*rgpE*	glycosyltransferase family GT2 protein RgpE	1892	761
762	MGCS36089_01462			*nrnA*	bifunctional oligoribonuclease/PAP phosphatase	1888	762
763	MGCS36089_02436			*mnmE*	MnmE family tRNA	1887	763
764	MGCS36089_00574			-	polynucleotide phosphorylase/polyadenylase	1883	764
765	MGCS36089_03188			-	helix-hairpin-helix domain-containing protein	1883	764
766	MGCS36089_03944			*tadA*	tRNA adenosine(34) deaminase TadA	1880	766
767	MGCS36089_01602			*rgpC*	ABC transporter polysaccharide/polyol phosphate	1878	767
768	MGCS36089_01302			-	YdbC family protein	1871	768
769	MGCS36089_03026			*xseA*	exodeoxyribonuclease VII large subunit XseA	1871	768
770	MGCS36089_02518			-	CRISPR-DR22 RNA	1867	770
771	MGCS36089_01974			*osmF*	OsmF superfamily glycine/betaine transport	1864	771
772	MGCS36089_01590			*dnaG*	DNA primase protein DnaG	1859	772
773	MGCS36089_02472			*manX*	ManX family PTS mannose/fructose IIA component	1858	773
774	MGCS36089_01104			-	hypothetical protein	1852	774
775	MGCS36089_02122			-	hypothetical protein	1848	775
776	MGCS36089_00940			*fhuG*	iron ABC transporter permease FhuG	1846	776
777	MGCS36089_00756			*rpmB*	50S ribosomal L28 protein RpmB	1844	777
778	MGCS36089_04154			*hisS*	histidine--tRNA synthase HisS	1844	777
779	MGCS36089_03914			-	DUF6198 family protein	1833	779
780	MGCS36089_01262			-	NUDIX hydrolase superfamily protein	1830	780
781	MGCS36089_02474			*yeiH*	YeiH family uncharacterized membrane protein	1830	780
782	MGCS36089_01464			-	GNAT family N-acetyltransferase	1826	782
783	MGCS36089_02228			-	asd RNA	1819	783
784	MGCS36089_02138			-	heme response regulator HssR-like putative TCS	1816	784
785	MGCS36089_00978			*yaaT*	cell fate regulator YaaT	1814	785
786	MGCS36089_03266			-	DUF960 domain-containing protein	1814	785
787	MGCS36089_01468			*add*	adenosine deaminase Add	1807	787
788	MGCS36089_01524			*ligA*	NAD-dependent DNA ligase LigA	1805	788
789	MGCS36089_01094			-	Nudix superfamily phosphohydrolase	1802	789
790	MGCS36089_03618			*htsC*	heme ABC transporter ATP-binding protein HtsC	1801	790
791	MGCS36089_01240			-	Cof-type HAD-IIB family hydrolase	1798	791
792	MGCS36089_02486			*lepA*	translation elongation factor 4 LepA	1791	792

No.	Locus tag	SignalP6 predicted(1)	Virulence(2)	Gene	Function	RPKMs in NHP 14(3)	RANK(4)
793	MGCS36089_00986			*cutC*	copper homeostasis protein CutC	1789	793
794	MGCS36089_01268			*pnpS*	phosphate TCS signal transduction histidine	1789	793
795	MGCS36089_02148			-	NusG domain II-containing protein	1779	795
796	MGCS36089_01038			-	UhpC family MFS transporter	1769	796
797	MGCS36089_03460			*srtB*	pilus polymerization class B sortase SrtB	1766	797
798	MGCS36089_03940			-	Bacteria_small_SRP RNA	1761	798
799	MGCS36089_02874			*murA_2*	UDP-N-acetylglucosamine	1760	799
800	MGCS36089_04224			*rplI*	50S ribosomal L9 protein RplI	1757	800
801	MGCS36089_02502			-	CRISPR-DR22 RNA.	1753	801
802	MGCS36089_04138			-	YmcA-related cell fate/competence/biofilm	1746	802
803	MGCS36089_01188			*thrS*	threonyl-tRNA synthetase ThrS	1745	803
804	MGCS36089_01752			*sptS*	SptS-like TCS sensor histidine kinase	1744	804
805	MGCS36089_02942			*queA*	S-adenosylmethionine ribosyltransferase-isomerase QueA	1740	805
806	MGCS36089_00518	Secreted		-	cell surface UshA family bifunctional. Cell-wall anchoring predicted sortase	1735	806
807	MGCS36089_01624			*pepT*	peptidase (T) PepT	1730	807
808	MGCS36089_03020			*tlyA*	TlyA family RNA methyltransferase	1730	807
809	MGCS36089_03748			-	MFS transporter	1724	809
810	MGCS36089_03718			*pepX*	PepX family Xaa-Pro dipeptidyl-peptidase	1720	810
811	MGCS36089_01742			*gdhA*	NADP-specific glutamate dehydrogenase GdhA	1718	811
812	MGCS36089_01056			*mtsC*	metal ABC transporter permease MtsC	1710	812
813	MGCS36089_01574			-	neutral zinc metallopeptidase	1706	813
814	MGCS36089_01242			-	Cof-type HAD-IIB family hydrolase	1702	814
815	MGCS36089_02202			-	DUF1836 domain-containing protein	1700	815
816	MGCS36089_02980			*ddl*	D-alanine--D-alanine ligase Ddl	1697	816
817	MGCS36089_00124			*vanZ*	VanZ family putative glycopeptide antibiotic	1694	817
818	MGCS36089_03752			-	major facilitator superfamily protein	1692	818
819	MGCS36089_00360			*hslO*	Hsp33 family molecular chaperone HslO	1691	819
820	MGCS36089_00500			*rgfB*	endonuclease/exonuclease/phosphatase family	1691	819
821	MGCS36089_04146			-	YitT family protein putative ABC transporter	1690	821
822	MGCS36089_02240			*fhs_1*	formate--tetrahydrofolate ligase Fhs. CW-Pred predicted sortase C cell-wall anchoring	1686	822
823	MGCS36089_02510			-	CRISPR-DR22 RNA	1684	823

No.	Locus tag	SignalP6 predicted(1)	Virulence(2)	Gene	Function	RPKMs in NHP 14(3)	RANK(4)
824	MGCS36089_01970			-	Na+ driven multidrug efflux pump	1682	824
825	MGCS36089_03658			*uvrA*	excinuclease ABC subunit (A) UvrA	1681	825
826	MGCS36089_03882		Virulence	*fasC*	signal transduction sensor histidine kinase	1681	825
827	MGCS36089_01058	Lipo		-	peptidylprolyl isomerase lipoprotein	1680	827
828	MGCS36089_02908			*clcB*	voltage-gated ClC-type chloride channel ClcB	1670	828
829	MGCS36089_02914			-	transcriptional regulator	1668	829
830	MGCS36089_01748			-	GTP pyrophosphokinase family protein	1667	830
831	MGCS36089_00876			*snf*	SWF/SNF family helicase	1659	831
832	MGCS36089_01972			*opuBA*	OpuBA superfamily glycine/betaine ABC	1655	832
833	MGCS36089_04026			-	hypothetical protein	1655	832
834	MGCS36089_02178			*gid*	tRNA (uracil-5-)-methyltransferase/glucose	1650	834
835	MGCS36089_01690			*carB*	carbamoyl-phosphate synthase large subunit CarB	1637	835
836	MGCS36089_03072			*ftsQ*	cell division protein FtsQ/DivIB	1637	835
837	MGCS36089_03458	Secreted		-	pilus ancillary/minor protein 2	1637	835
838	MGCS36089_00022			-	hypothetical protein	1628	838
839	MGCS36089_03146			*argR_2*	arginine responsive transcriptional repressor	1628	838
840	MGCS36089_03756			-	type I restriction-modification system	1627	840
841	MGCS36089_03144			*fnr*	Crp/Fnr family transcriptional regulator	1625	841
842	MGCS36089_03880		Virulence	*fasA*	signal transduction response regulator protein	1625	841
843	MGCS36089_00424			-	putative transcriptional regulator	1622	843
844	MGCS36089_03642			*scrA*	sucrose-specific PTS fused IIB/IIC/IIA component	1621	844
845	MGCS36089_00308			*tyrS*	tyrosyl-tRNA synthetase TyrS	1620	845
846	MGCS36089_01518	Secreted		-	cell surface extracellular nuclease. Cell-wall anchoring predicted sortase	1615	846
847	MGCS36089_00742			*lytS*	TCS sensor histidine kinase LytS	1609	847
848	MGCS36089_01362			*murM*	peptidoglycan lipid II--L-alanine ligase protein	1608	848
849	MGCS36089_01382			*agaW*	PTS transporter	1601	849
850	MGCS36089_01296			-	DUF3270 domain-containing protein	1598	850
851	MGCS36089_00790	Secreted		*dacA_1*	secreted D-alanyl-D-alanine carboxypeptidase	1594	851
852	MGCS36089_01898			*xerD_2*	site-specific integrase	1594	851
853	MGCS36089_01900	Secreted		*dacA_3*	secreted D,D-carboxypeptidase penicillin-binding	1591	853
854	MGCS36089_02460	Lipo		-	extracellular solute-binding lipoprotein	1590	854
855	MGCS36089_04150			-	YitT family protein putative ABC transporter	1590	854

No.	Locus tag	SignalP6 predicted(1)	Virulence(2)	Gene	Function	RPKMs in NHP 14(3)	RANK(4)
856	MGCS36089_03400			-	FadH2 family uncharacterized NAD(FAD)-dependent	1582	856
857	MGCS36089_03250			-	LLM class flavin-dependent oxidoreductase	1576	857
858	MGCS36089_00558			-	Crp family cyclic nucleotide-binding	1570	858
859	MGCS36089_02962			*kup*	potassium uptake protein Kup	1568	859
860	MGCS36089_01618			-	LTA synthase family protein	1565	860
861	MGCS36089_02412			-	GNAT family N-acetyltransferase	1565	860
862	MGCS36089_02506			-	CRISPR-DR22 RNA	1561	862
863	MGCS36089_00412			-	hypothetical protein	1559	863
864	MGCS36089_03744			-	PLDc N-terminal domain-containing protein	1559	863
865	MGCS36089_01484			-	HAD-IA family hydrolase	1555	865
866	MGCS36089_00654			-	Txe/YoeB family addiction module toxin	1553	866
867	MGCS36089_00796			*oppB_1*	oligopeptide ABC transporter permease protein	1552	867
868	MGCS36089_02964			*deaD*	DEAD/DEAH box helicase	1547	868
869	MGCS36089_01956			*ribP*	ribose-phosphate pyrophosphokinase RibP	1543	869
870	MGCS36089_00542			*cdsA*	phosphatidate cytidylyltransferase CdsA	1541	870
871	MGCS36089_00448			-	radical SAM protein	1538	871
872	MGCS36089_03876			*rnpA*	ribonuclease P protein component RnpA	1538	871
873	MGCS36089_01190			-	putative ABC transporter ATP-binding component	1536	873
874	MGCS36089_01678			-	membrane-associated alkaline phosphatase	1523	874
875	MGCS36089_03710			-	beta-lactamase family protein	1516	875
876	MGCS36089_02230			*cls*	cardiolipin synthase	1514	876
877	MGCS36089_03790			*tsaD*	tRNA (adenosine(37)-N6)-threonylcarbamoyltransferase complex transferase subunit TsaD	1511	877
878	MGCS36089_00726			*thiD*	bifunctional hydroxymethylpyrimidine kinase/phosphomethylpyrimidine kinase PdxK	1510	878
879	MGCS36089_02014			*gyrA*	DNA gyrase subunit A GyrA	1509	879
880	MGCS36089_03340			*luxS*	S-ribosylhomocysteine lyase	1507	880
881	MGCS36089_03300			*cysK*	cysteine synthase A CysK	1505	881
882	MGCS36089_01258			-	permease	1502	882
883	MGCS36089_00882			-	GNAT family N-acetyltransferase	1501	883
884	MGCS36089_00938			-	cytoplasmic protein	1501	883
885	MGCS36089_03424			-	hypothetical protein	1501	883
886	MGCS36089_03022			*ispA*	IspA family geranylgeranyl pyrophosphate	1493	886

No.	Locus tag	SignalP6 predicted(1)	Virulence(2)	Gene	Function	RPKMs in NHP 14(3)	RANK(4)
887	MGCS36089_00540			*uppS*	UDP pyrophosphate synthase UppS	1491	887
888	MGCS36089_00854			*ktrB*	potassium uptake transporter channel subunit	1487	888
889	MGCS36089_04126			-	VOC family protein	1487	888
890	MGCS36089_02650			-	hypothetical protein	1480	890
891	MGCS36089_02440			*nfnB*	NfnB family nitroreductase	1475	891
892	MGCS36089_04182			-	helix-turn-helix domain-containing	1462	892
893	MGCS36089_02912			-	DUF3169 family protein	1460	893
894	MGCS36089_00426			-	hypothetical protein	1458	894
895	MGCS36089_02586	Secreted		-	putative secreted protein	1457	895
896	MGCS36089_01312			-	PhoE superfamily phosphatase	1455	896
897	MGCS36089_03398			-	putative transcriptional regulator	1437	897
898	MGCS36089_01600			*rgpB*	glycosyltransferase family GT2 protein RgpB	1435	898
899	MGCS36089_01864			*copY_1*	copper transport repressor CopY	1435	898
900	MGCS36089_01572			-	ABC transporter ATP-binding component LolD-like	1432	900
901	MGCS36089_00596			-	helix-turn-helix transcriptional regulator	1426	901
902	MGCS36089_02182			-	SatD family protein	1423	902
903	MGCS36089_03924			-	Mga-related helix-turn-helix domain-containing	1423	902
904	MGCS36089_01946			-	gamma-glutamyl-gamma-aminobutyrate hydrolase	1421	904
905	MGCS36089_04012	Secreted	Virulence	*nga*	secreted nicotine adenine dinucleotide	1415	905
906	MGCS36089_02998			-	Spy1186876 RNA	1414	906
907	MGCS36089_01870			*copB*	copper-exporting ATPase cCopB	1412	907
908	MGCS36089_00770	Secreted		-	ABC amino acid transporter substrate-binding	1406	908
909	MGCS36089_01604			*rgpD*	ABC transporter polysaccharide/polyol phosphate	1399	909
910	MGCS36089_01902			-	polysaccharide deacetylase family protein	1398	910
911	MGCS36089_01954			*nifS_2*	NifS superfamily cysteine desulfurase	1398	910
912	MGCS36089_03044			-	amino acid ABC transporter permease	1398	910
913	MGCS36089_04238			*mnmA*	tRNA 2-thiouridine(34) synthase MnmA	1398	910
914	MGCS36089_01332			*pepF_1*	oligoendopeptidase PepF	1395	914
915	MGCS36089_04134			*mutL*	DNA mismatch repair endonuclease MutL	1392	915
916	MGCS36089_00778			*rgpG*	undecaprenyl/decaprenyl-phosphate alpha-N-acetylglucosaminyl 1-phosphate transferase RgpG	1388	916
917	MGCS36089_03590			-	DUF156 family protein	1387	917
918	MGCS36089_01634			*infC*	translation initiation factor InfC	1384	918

No.	Locus tag	SignalP6 predicted[(1)]	Virulence[(2)]	Gene	Function	RPKMs in NHP 14[(3)]	RANK[(4)]
919	MGCS36089_02396			*parE*	DNA topoisomerase IV subunit B ParE	1381	919
920	MGCS36089_02636			*hflX*	GTP-binding protein HflX	1371	920
921	MGCS36089_04088			-	hypothetical protein	1371	920
922	MGCS36089_01070			*pyrH*	UMP kinase PyrH	1366	922
923	MGCS36089_02534			*nagD*	NagD family hydrolase	1360	923
924	MGCS36089_02548			*murE_2*	UDP-N-acetylmuramoylalanyl-D-glutamate-2,	1359	924
925	MGCS36089_02862			*uxaC*	uronate isomerase	1359	924
926	MGCS36089_01776			*dyr*	dihydrofolate reductase Dyr	1358	926
927	MGCS36089_02632			*rnz*	ribonuclease Rnz	1357	927
928	MGCS36089_03484			*rimP*	ribosome maturation factor RimP	1356	928
929	MGCS36089_03654			*comEB*	competence protein ComEB	1356	928
930	MGCS36089_01040			*glmU*	bifunctional UDP-N-acetylglucosamine diphosphorylase/glucosamine-1-phosphate N-acetyltransferase GlmU	1355	930
931	MGCS36089_02394			-	hypothetical protein	1352	931
932	MGCS36089_04152			*aspS*	aspartyl-tRNA synthetase	1352	931
933	MGCS36089_01210			*paaJ*	acetyl-CoA acetyl transferase PaaJ	1349	933
934	MGCS36089_02628			*recJ*	single-stranded-DNA-specific exonuclease RecJ	1348	934
935	MGCS36089_03038			*feoB*	ferrous iron transport protein (B) FeoB	1348	934
936	MGCS36089_01646			*aroC*	chorismate synthase AroC	1346	936
937	MGCS36089_04220			-	Veg family protein	1341	937
938	MGCS36089_01090			*dgkA*	diacylglycerol kinase DgkA	1340	938
939	MGCS36089_02666			*malG*	maltose/maltodextrin ABC transport system	1332	939
940	MGCS36089_00820			-	cysteine hydrolase	1327	940
941	MGCS36089_00934			*pflA*	pyruvate formate-lyase activating enzyme PflA	1327	940
942	MGCS36089_01756			*mvaD*	diphosphomevalonate decarboxylase MvaD	1327	940
943	MGCS36089_03438			*lacB*	galactose-6-phosphate isomerase subunit LacB	1326	943
944	MGCS36089_02312			-	DUF2130 domain-containing protein	1320	944
945	MGCS36089_03846			*rrmV*	5S rRNA maturation endonuclease RnmV	1317	945
946	MGCS36089_03024			*xseB*	exodeoxyribonuclease VII small subunit XseB	1316	946
947	MGCS36089_02402			*ung*	uracil-DNA glycosylase Ung	1315	947
948	MGCS36089_04200			-	thioredoxin family protein	1313	948
949	MGCS36089_00212			*infA*	translation initiation factor IF-1 protein InfA	1312	949

No.	Locus tag	SignalP6 predicted[1]	Virulence[2]	Gene	Function	RPKMs in NHP 14[3]	RANK[4]
950	MGCS36089_03442			*sgcC*	galactitol-specific PTS transporter IIB subunit	1311	950
951	MGCS36089_03800			*oppF_2*	oligopeptide ABC transporter ATPase OppF	1311	950
952	MGCS36089_02444			*uvrC*	excinuclease ABC subunit UvrC	1310	952
953	MGCS36089_03688			*recD*	ATP-dependent DNA helicase RecD	1309	953
954	MGCS36089_00488			*pabB*	para-aminobenzoate synthetase	1306	954
955	MGCS36089_01364			-	sugar-phosphatase	1303	955
956	MGCS36089_04086			-	Spy491738 RNA	1303	955
957	MGCS36089_00946			*fhuA*	ferrichrome ABC transporter ATP-binding protein	1302	957
958	MGCS36089_01160			*secG*	preprotein translocase subunit SecG	1300	958
959	MGCS36089_03900			*dut*	deoxyuridine 5'-triphosphate nucleotidohydrolase	1298	959
960	MGCS36089_03680			-	colicin V production family protein	1297	960
961	MGCS36089_02630			*dltE*	short-chain dehydrogenase DltE	1296	961
962	MGCS36089_00146			*ruvB*	Holliday junction branch migration DNA helicase	1292	962
963	MGCS36089_02606			-	Abi family CAAX protease self-immunity protein	1291	963
964	MGCS36089_01570			-	ABC transporter permease	1286	964
965	MGCS36089_02588	Secreted		*aphA*	secreted acid phosphatase AphA	1276	965
966	MGCS36089_01582	Lipo		-	ABC transporter substrate-binding lipoprotein	1272	966
967	MGCS36089_00774			*uppP*	undecaprenyl pyrophosphate phosphatase UppP	1268	967
968	MGCS36089_00728			-	ECF transporter S component	1266	968
969	MGCS36089_01092			*era*	GTPase Era	1258	969
970	MGCS36089_00814			*yhbY*	ribosome assembly RNA-binding protein YhbY	1256	970
971	MGCS36089_03428			-	TetR/AcrR family transcriptional regulator	1254	971
972	MGCS36089_03122			*sdrC*	SdrC family PDZ domain-containing protein	1251	972
973	MGCS36089_03938	Secreted	Virulence	*speG*	streptococcal pyrogenic exotoxin (G) SpeG	1247	973
974	MGCS36089_04132			*mdtH*	MdtH-related MFS multidrug resistance	1247	973
975	MGCS36089_02358			*lepB_1*	signal peptidase I	1243	975
976	MGCS36089_04038			*ctsR*	CtsR family transcriptional regulator	1242	976
977	MGCS36089_01282			*tex*	RNA-binding transcriptional accessory protein	1240	977
978	MGCS36089_02462			-	YesN family TCS DNA-binding response regulator	1240	977
979	MGCS36089_02864			*eda*	Eda family bifunctional keto-hydroxyglutarate-aldolase/keto-deoxy-phosphogluconate aldolase	1237	979
980	MGCS36089_00744			*lytR*	TCS DNA-binding response regulator LytR	1236	980
981	MGCS36089_01612			-	DUF2304 domain-containing protein	1235	981

No.	Locus tag	SignalP6 predicted[1]	Virulence[2]	Gene	Function	RPKMs in NHP 14[3]	RANK[4]
982	MGCS36089_04024			*rluA_2*	RluA family pseudouridine synthase	1233	982
983	MGCS36089_02858			-	SDR family D-mannonate oxidoreductase	1231	983
984	MGCS36089_00944	Lipo		*fhuD*	iron-hydroxamate ABC transporter	1226	984
985	MGCS36089_02274			*deoA*	pyrimidine-nucleoside phosphorylase DeoA	1226	984
986	MGCS36089_03966			*proV*	proline/glycine betaine ABC transporter permease	1226	984
987	MGCS36089_02546			-	DisA N domain-containing diadenylate cyclase	1224	987
988	MGCS36089_04222			*dnaC*	replicative DNA helicase DnaC	1224	987
989	MGCS36089_00026			*tilS*	tRNA lysidine(34) synthetase TilS	1223	989
990	MGCS36089_02482			-	RnaY family phosphodiesterase	1221	990
991	MGCS36089_00010			*engD*	redox-regulated ATPase EngD	1217	991
992	MGCS36089_01654			-	hypothetical protein	1215	992
993	MGCS36089_01662			-	IS30 family transposase	1213	993
994	MGCS36089_03602			*asnB*	L-asparaginase AsnB	1210	994
995	MGCS36089_03000			*zntA*	ZntA family P-type heavy metal transporter	1207	995
996	MGCS36089_03578			*ppdK*	pyruvate phosphate dikinase PpdK	1207	995
997	MGCS36089_01450			-	PTS sugar transport IIB subunit	1206	997
998	MGCS36089_03844			*rsmA*	16S rRNA (adenine(1518)-N(6)/adenine(1519)-N(6))-dimethyltransferase RsmA	1203	998
999	MGCS36089_03450			*copZ_2*	copper chaperone CopZ	1202	999
1000	MGCS36089_04250			*cbiO2*	cobalt ABC transporter ATPase CbiO1	1196	1000
1001	MGCS36089_03616			-	CydC-related ABC transporter ATPase/permease	1192	1001
1002	MGCS36089_02450			*yjjG*	YjjG family noncanonical pyrimidine	1187	1002
1003	MGCS36089_00706			-	IS30 family transposase	1186	1003
1004	MGCS36089_02996			*pyrD*	dihydroorotate dehydrogenase PyrD	1186	1003
1005	MGCS36089_01000			*exoA*	exodeoxyribonuclease III protein ExoA	1185	1005
1006	MGCS36089_00294			*cydD*	thiol reductant ABC exporter subunit CydD	1179	1006
1007	MGCS36089_02654			*glgP*	maltodextrin phosphorylase protein GlgP	1178	1007
1008	MGCS36089_01158			-	multidrug efflux MFS transporter	1176	1008
1009	MGCS36089_01266			*pnpR*	phosphate TCS signal transduction response	1170	1009
1010	MGCS36089_01644			*aroD*	type I 3-dehydroquinate dehydratase AroD	1168	1010
1011	MGCS36089_00510			-	PreQ1 RNA	1167	1011
1012	MGCS36089_01790			-	L10_leader RNA	1166	1012
1013	MGCS36089_01212			*ppsB*	long-chain fatty acid--CoA ligase PpsB	1164	1013

No.	Locus tag	SignalP6 predicted(1)	Virulence(2)	Gene	Function	RPKMs in NHP 14(3)	RANK(4)
1014	MGCS36089_02692	Lipo		*glnP_2*	glutamine-binding protein/glutamine transport	1161	1014
1015	MGCS36089_03290			-	IS30 family transposase	1157	1015
1016	MGCS36089_03404			*glpO*	type 1 glycerol-3-phosphate oxidase GlpO	1157	1015
1017	MGCS36089_00868			*nrdR*	transcriptional regulator NrdR	1156	1017
1018	MGCS36089_04130			*ruvA*	Holliday junction ATP-dependent DNA helicase	1152	1018
1019	MGCS36089_00340			-	CAAX amino terminal protease family membrane	1148	1019
1020	MGCS36089_00504			-	M42 family metallopeptidase	1146	1020
1021	MGCS36089_01398	Secreted	Virulence	*hylB*	secreted hyaluronate lyase HylB	1146	1020
1022	MGCS36089_03582			*ccpN*	CcpN family CBS pair domain transcriptional	1146	1020
1023	MGCS36089_02714			-	hypothetical protein	1138	1023
1024	MGCS36089_00428			-	FAD/NAD(P)-binding protein	1132	1024
1025	MGCS36089_03318			*fmt*	methionyl-tRNA formyl transferase Fmt	1132	1024
1026	MGCS36089_03444			*sgaB*	galactitol-specific PTS transporter IIC subunit	1132	1024
1027	MGCS36089_01316			-	aminoacyl-tRNA deacylase	1130	1027
1028	MGCS36089_00320			*comYA*	competence system type II/IV secretion system	1129	1028
1029	MGCS36089_02880			*birA*	bifunctional biotin--[acetyl-CoA-carboxylase]	1129	1028
1030	MGCS36089_03042			-	amino acid ABC transporter ATP-binding protein	1125	1030
1031	MGCS36089_01688			*carA*	carbamoyl-phosphate synthase small subunit CarA	1123	1031
1032	MGCS36089_00580			*cysS*	cysteine--tRNA synthetase CysS	1121	1032
1033	MGCS36089_03496			*hit*	HIT family protein	1121	1032
1034	MGCS36089_04264			*recF*	DNA replication/repair protein RecF	1116	1034
1035	MGCS36089_02956	Secreted		*plsC*	secreted 1-acyl-sn-glycerol-3-phosphate	1115	1035
1036	MGCS36089_00278			-	HNH endonuclease	1114	1036
1037	MGCS36089_04090			*yybR*	putative HTH-type transcriptional regulator	1111	1037
1038	MGCS36089_00116			*purL*	phosphoribosylformylglycinamidine synthase PurL	1110	1038
1039	MGCS36089_00880			*murC*	UDP-N-acetylmuramate--L-alanine ligase MurC	1104	1039
1040	MGCS36089_03182			-	AAA family ATPase	1104	1039
1041	MGCS36089_00296			*cdyC*	thiol reductant ABC exporter subunit CydC	1103	1041
1042	MGCS36089_03100			*rlmN*	23S rRNA (adenine(2503)-C(2))-methyltransferase	1103	1041
1043	MGCS36089_03198			-	hypothetical protein	1102	1043
1044	MGCS36089_00164			-	IS30 family transposase	1097	1044
1045	MGCS36089_00786			*sufE*	SUF system NifU family Fe-S cluster assembly	1094	1045

No.	Locus tag	SignalP6 predicted[1]	Virulence[2]	Gene	Function	RPKMs in NHP 14[3]	RANK[4]
1046	MGCS36089_03746			-	Cro/CI family transcriptional regulator	1088	1046
1047	MGCS36089_02520			-	CRISPR-DR22 RNA	1086	1047
1048	MGCS36089_04248			*cbiQ_2*	cobalt ABC transporter permease CbiQ	1086	1047
1049	MGCS36089_01030			*fabG_1*	3-oxoacyl-ACP reductase FabG	1084	1049
1050	MGCS36089_01614			-	DUF2142 domain-containing protein	1082	1050
1051	MGCS36089_01734			-	tRNA CCA-pyrophosphorylase	1077	1051
1052	MGCS36089_03086			*glcK*	glucokinase GlcK	1074	1052
1053	MGCS36089_00334			-	class I SAM-dependent methyltransferase	1068	1053
1054	MGCS36089_01944			*radC*	DNA repair protein RadC	1065	1054
1055	MGCS36089_02476			-	DUF2974 domain-containing protein	1065	1054
1056	MGCS36089_02198			*ylqF*	ribosome biogenesis GTPase YlqF	1059	1056
1057	MGCS36089_02324			-	GntR family transcriptional regulator	1059	1056
1058	MGCS36089_04286			*rlmH*	23S rRNA (pseudouridine(1915)-N(3))-methyltransferase RlmH	1059	1056
1059	MGCS36089_02398			*plsY*	glycerol-3-phosphate 1-O-acyltransferase PlsY	1054	1059
1060	MGCS36089_01948			*rex*	redox-sensing transcriptional repressor Rex	1053	1060
1061	MGCS36089_00002			*dnaA*	chromosomal replication initiator protein DnaA	1048	1061
1062	MGCS36089_00404			*celB_1*	PTS transporter cellobiose-specific IIC	1047	1062
1063	MGCS36089_02134			-	helix-turn-helix transcriptional regulator	1044	1063
1064	MGCS36089_02890			*udk*	uridine kinase Udk	1044	1063
1065	MGCS36089_01182			*amyS*	alpha-amylase AmyS	1038	1065
1066	MGCS36089_03906			-	ABC transporter ATPase/permease	1036	1066
1067	MGCS36089_03014	Secreted		-	putative extracellular cell surface	1034	1067
1068	MGCS36089_00568			-	PTS transporter IIC component UlaA-like protein	1033	1068
1069	MGCS36089_03238			-	beta-N-acetylglucosaminidase	1033	1068
1070	MGCS36089_01656			*nifS_1*	cysteine desulfurase NifS	1030	1070
1071	MGCS36089_02136			*yhhX*	PRK10206 superfamily putative oxidoreductase. 1039009..1044144. Region of difference 36089_ROD.6, putative MGE	1028	1071
1072	MGCS36089_00862			*yceD*	large ribosomal RNA subunit accumulation protein	1027	1072
1073	MGCS36089_00298			*preA*	polyprenyl synthetase family protein PreA	1022	1073
1074	MGCS36089_01476			*clcA*	voltage-gated chloride channel family protein	1022	1073
1075	MGCS36089_03524			*serS*	seryl-tRNA synthetase SerS	1018	1075
1076	MGCS36089_04072			-	AAA family ATPase domain-containing DNA-binding	1012	1076

No.	Locus tag	SignalP6 predicted(1)	Virulence(2)	Gene	Function	RPKMs in NHP 14(3)	RANK(4)
1077	MGCS36089_02484			*msrB*	peptide-methionine (R)-S-oxide reductase MsrB	1006	1077
1078	MGCS36089_02976			-	TIGR02206 family membrane protein	1005	1078
1079	MGCS36089_02130			-	hypothetical protein	1004	1079
1080	MGCS36089_03218		Virulence	*trxR*	TCS DNA-binding response regulator protein TrxR	1003	1080
1081	MGCS36089_03422			*nptA*	NtpA family Na/Pi cotransporter	1000	1081
1082	MGCS36089_02196			*rnhB*	HII ribonuclease RnhB	993	1082
1083	MGCS36089_01770			*mvaS1*	hydroxymethylglutaryl-CoA reductase protein (1)	992	1083
1084	MGCS36089_02988			*niaX*	niacin transporter NiaX	992	1083
1085	MGCS36089_02338			*pcrA*	DNA helicase PcrA	989	1085
1086	MGCS36089_03758			-	type I restriction-modification system (M)	989	1085
1087	MGCS36089_03506			*azgA*	AzgA family permease	985	1087
1088	MGCS36089_02080			-	site-specific integrase	979	1088
1089	MGCS36089_02422			-	LysR family transcriptional regulator	975	1089
1090	MGCS36089_02836			-	23S rRNA (uracil(1939)-C(5))-methyltransferase	975	1089
1091	MGCS36089_01560			*endA*	DNA-entry competence-associated nuclease EndA	974	1091
1092	MGCS36089_03164			-	Gfo/Idh/MocA family oxidoreductase. 1567148..1567775. CRISPR with 10 repeat units	972	1092
1093	MGCS36089_03132			-	IS110 family transposase	968	1093
1094	MGCS36089_01714			-	PTS transporter subunit IIC	967	1094
1095	MGCS36089_03094			-	MdlB family multidrug ABC transporter	965	1095
1096	MGCS36089_02376			-	SDUF3862 domain-containing lipoprotein. ignalP-6 predicted lipid anchoring signal peptide,	960	1096
1097	MGCS36089_02670			*malA*	maltodextrose utilization protein MalA	958	1097
1098	MGCS36089_02572			*frmB*	FrmB family esterase	957	1098
1099	MGCS36089_01642			*rlmK*	23S rRNA methyltransferase RmlK	955	1099
1100	MGCS36089_03200			*aroGA*	bifunctional 3-deoxy-7-phosphoheptulonate synthase/chorismate mutase AroGA	953	1100
1101	MGCS36089_03580			-	kinase/pyrophosphorylase	952	1101
1102	MGCS36089_01256			-	DUF1980 domain-containing protein	948	1102
1103	MGCS36089_03124			*coaD*	pantetheine-phosphate adenylyltransferase CoaD	947	1103
1104	MGCS36089_03570			-	drug/metabolite transporter superfamily protein	946	1104
1105	MGCS36089_00878			-	hypothetical protein	943	1105
1106	MGCS36089_03982			*ulaB*	ascorbate-specific PTS transporter EIIB protein	943	1105
1107	MGCS36089_02010			-	FolA superfamily dihydrofolate reductase	942	1107

No.	Locus tag	SignalP6 predicted(1)	Virulence(2)	Gene	Function	RPKMs in NHP 14(3)	RANK(4)
1108	MGCS36089_01324			-	IS110 family transposase	937	1108
1109	MGCS36089_01718			*fruR*	fructose operon transcriptional repressor	936	1109
1110	MGCS36089_00444			*flaR*	DNA topology modulation protein	932	1110
1111	MGCS36089_02454			-	maltose/galactose O-acetyltransferase	930	1111
1112	MGCS36089_03414			*glyQ*	glycine--tRNA ligase alpha subunit GlyQ	930	1111
1113	MGCS36089_03352			*recU*	Holliday junction resolvase RecU	929	1113
1114	MGCS36089_03708			-	PurR family transcriptional regulator	928	1114
1115	MGCS36089_01360			*murN*	peptidoglycan lipid II-Ala--L-alanine ligase	927	1115
1116	MGCS36089_03108			-	IS110 family transposase	925	1116
1117	MGCS36089_03916			*glpG*	GlpG family membrane associated serine protease	924	1117
1118	MGCS36089_04054			*hutI*	imidazolonepropionase HutI	921	1118
1119	MGCS36089_03258			*fadB*	FadB family 3-hydroxyacyl-CoA dehydrogenase	916	1119
1120	MGCS36089_02156			-	polyprenyl synthetase family protein	912	1120
1121	MGCS36089_00598			-	IS30 family transposase	910	1121
1122	MGCS36089_01400			-	hypothetical protein	910	1121
1123	MGCS36089_03212			-	permease	908	1123
1124	MGCS36089_03216			*lacZ*	beta-galactosidase LacZ	908	1123
1125	MGCS36089_03214			*aroE*	shikimate dehydrogenase AroE	907	1125
1126	MGCS36089_02052			-	SSRC38 RNA	905	1126
1127	MGCS36089_03220		Virulence	*trxS*	TCS sensor histidine kinase TrxS	903	1127
1128	MGCS36089_04240			*sdhB*	L-serine dehydratase beta subunit SdhB	903	1127
1129	MGCS36089_02602			*mutX*	8-oxo-dGTP diphosphatase MutX	902	1129
1130	MGCS36089_03310		Virulence	*liaF*	three component system signal transduction	901	1130
1131	MGCS36089_04178			-	site-specific integrase	898	1131
1132	MGCS36089_03682			-	hypothetical protein	896	1132
1133	MGCS36089_02026	Lipo		-	streptococcal histidine triad (HIT) lipoprotein	893	1133
1134	MGCS36089_00996			-	PhnB family glyoxalase/bleomycin	892	1134
1135	MGCS36089_03370			*hisM*	HisM family amino acid ABC transporter permease	890	1135
1136	MGCS36089_01576			*rexB*	ATP-dependent nuclease B subunit RexB	885	1136
1137	MGCS36089_02388			-	DUF2969 domain-containing protein	883	1137
1138	MGCS36089_01394			*kdgK*	2-dehydro-3-deoxygluconokinase KdgK	882	1138
1139	MGCS36089_01084			*phoH*	phosphate starvation-inducible protein PhoH	881	1139

No.	Locus tag	SignalP6 predicted[1]	Virulence[2]	Gene	Function	RPKMs in NHP 14[3]	RANK[4]
1140	MGCS36089_00128	Secreted		-	CHAP domain-containing protein	878	1140
1141	MGCS36089_02006			-	MdlB superfamily multidrug ABC transporter	878	1140
1142	MGCS36089_02554			-	DUF4097 family beta strand repeat-containing	877	1142
1143	MGCS36089_04124			*cinA*	competence/damage-inducible protein CinA	875	1143
1144	MGCS36089_00810			*yqeG*	HAD IIIA-type phosphatase YqeG	874	1144
1145	MGCS36089_03854			*nanK*	N-acetylmannosamine kinase NanK	874	1144
1146	MGCS36089_01740			-	MdlB family multidrug ABC transporter ATPase and	873	1146
1147	MGCS36089_02614			*dadA*	FAD-binding oxidoreductase DadA	870	1147
1148	MGCS36089_03656			*pepP*	PepP family Xaa-Pro peptidase	865	1148
1149	MGCS36089_04028	Secreted		*pepD_2*	secreted dipeptidase PepD	861	1149
1150	MGCS36089_01074			*cvfB*	S1 RNA-binding domain-containing protein CvfB	858	1150
1151	MGCS36089_02496			-	CRISPR-DR22 RNA	858	1150
1152	MGCS36089_02498			-	CRISPR-DR22 RNA	858	1150
1153	MGCS36089_01686			*pyrB*	aspartate transcarbomoylase PyrB	857	1153
1154	MGCS36089_02984			*pbp2B*	penicillin-binding protein PBP2B/FtsI	856	1154
1155	MGCS36089_01452			-	PTS sugar transport IIC subunit	854	1155
1156	MGCS36089_01936			*eriC*	EriC family voltage gated chloride channel	851	1156
1157	MGCS36089_03336			-	3-hydroxybutyrate dehydrogenase	851	1156
1158	MGCS36089_00772	Secreted		-	DUF2207 domain-containing secreted protein	850	1158
1159	MGCS36089_00458			-	PhoE family phosphoglycerate mutase. 194241..203280. Region of difference 36089_ROD.2, putative MGE	848	1159
1160	MGCS36089_00440			-	hypothetical protein	846	1160
1161	MGCS36089_00892			-	SpoU family RNA methyltransferase	845	1161
1162	MGCS36089_01420			-	MBL fold metallo-hydrolase	845	1161
1163	MGCS36089_00384			-	PTS sugar transporter subunit IIC	842	1163
1164	MGCS36089_01086			-	uracil DNA glycosylase superfamily protein	836	1164
1165	MGCS36089_02866			*fadR*	FadR family DNA-binding transcriptional	834	1165
1166	MGCS36089_00134			*purK*	5-(carboxyamino)imidazole ribonucleotide	833	1166
1167	MGCS36089_03672			*mutY*	A/G-specific adenine glycosylase MutY	830	1167
1168	MGCS36089_01564			*pheT*	phenylalanyl-tRNA synthetase beta subunit PheT	818	1168
1169	MGCS36089_03406			*glpK*	glycerol kinase GlpK	816	1169
1170	MGCS36089_01750			*sptR*	SptR-like TCS DNA-binding response regulator	815	1170

No.	Locus tag	SignalP6 predicted(1)	Virulence(2)	Gene	Function	RPKMs in NHP 14(3)	RANK(4)
1171	MGCS36089_00532	Secreted	Virulence	*pulA_1*	cell surface pullulanase PulA. Cell-wall anchoring predicted sortase	814	1171
1172	MGCS36089_01558			*epuA*	DNA-directed RNA polymerase beta subunit EpuA	812	1172
1173	MGCS36089_04276			*yfhO*	YfhO family protein	811	1173
1174	MGCS36089_04010			*ifs*	nicotine adenine dinucleotide glycohydrolase	808	1174
1175	MGCS36089_03126			*rsmD*	16S rRNA (guanine(966)-N(2))-methyltransferase	806	1175
1176	MGCS36089_01310			-	HAD family hydrolase	805	1176
1177	MGCS36089_01988			*tdk*	thymidine kinase Tdk	803	1177
1178	MGCS36089_01216			*glnP_1*	glutamine ABC transporter permease GlnP	800	1178
1179	MGCS36089_01076			*msrA*	peptide-methionine (S)-S-oxide reductase MsrA	799	1179
1180	MGCS36089_01728			-	hypothetical protein	798	1180
1181	MGCS36089_02618			-	Nif3-like dinuclear metal center hexameric	797	1181
1182	MGCS36089_00274			-	helix-turn-helix transcriptional regulator	795	1182
1183	MGCS36089_00950			*murJ*	peptidoglycan lipid-II intermediate flippase	794	1183
1184	MGCS36089_02634			-	hypothetical protein	793	1184
1185	MGCS36089_01408			-	HAD family phosphatase	791	1185
1186	MGCS36089_03490			*cotS*	CotS family thiamine kinase	791	1185
1187	MGCS36089_03510			-	SSRC34_2 RNA	781	1187
1188	MGCS36089_03096			-	SmdA family multidrug ABC transporter	775	1188
1189	MGCS36089_03936			-	hypothetical protein	775	1188
1190	MGCS36089_00286			-	1,4-dihydroxy-2-naphthoate octaprenyltransferase	773	1190
1191	MGCS36089_01440			-	GntR family transcriptional regulator	773	1190
1192	MGCS36089_03034			*phrB*	PhrB family deoxyribodipyrimidine photolyase	768	1192
1193	MGCS36089_03692			*dinB*	DNA polymerase IV DinB	766	1193
1194	MGCS36089_03742			-	ABC transporter ATP-binding protein	760	1194
1195	MGCS36089_02664			*malF*	maltose/maltodextrin ABC transport system	759	1195
1196	MGCS36089_01326			-	ECF transporter S component	758	1196
1197	MGCS36089_02968			*metP_2*	methionine uptake ABC transporter permease MetP	755	1197
1198	MGCS36089_01306			-	ammonium transporter	754	1198
1199	MGCS36089_03646			*scrR*	sucrose operon repressor ScrR	749	1199
1200	MGCS36089_03896			-	carbonic anhydrase	748	1200
1201	MGCS36089_00506			-	ECF transporter S component	746	1201
1202	MGCS36089_01300			-	PrtC family collagenase-like protease	744	1202

No.	Locus tag	SignalP6 predicted[(1)]	Virulence[(2)]	Gene	Function	RPKMs in NHP 14[(3)]	RANK[(4)]
1203	MGCS36089_03794			*tsaB*	tRNA (adenosine(37)-N6)-threonylcarbamoyltransferase complex dimerization subunit type 1 TsaB	744	1202
1204	MGCS36089_00900			*murI*	glutamate racemase MurI	739	1204
1205	MGCS36089_01918			*murB*	UDP-N-acetylmuramate dehydrogenase MurB	738	1205
1206	MGCS36089_02536			*fatA*	FatA family acyl-[acyl-carrier-protein]	734	1206
1207	MGCS36089_00818			*yqeK*	bis(5'-nucleosyl)-tetraphosphatase (symmetrical)	733	1207
1208	MGCS36089_00780			*sufC*	Fe-S cluster assembly ATPase SufC	731	1208
1209	MGCS36089_01964			*rluA_1*	RluA family pseudouridine synthase	730	1209
1210	MGCS36089_04270			*trpS*	tryptophanyl-tRNA synthetase	725	1210
1211	MGCS36089_01200			*dhaK*	dihydroxyacetone kinase subunit DhaK	718	1211
1212	MGCS36089_00974			*tmk*	thymidylate kinase Tmk	717	1212
1213	MGCS36089_01724			-	putative peptidoglycan hydrolase	714	1213
1214	MGCS36089_02982			*recR*	recombination mediator RecR	714	1213
1215	MGCS36089_02616			*zupT*	ZIP family metal transporter ZupT	713	1215
1216	MGCS36089_04226			-	DHH family phosphoesterase	713	1215
1217	MGCS36089_02406	Lipo		-	amino-acid ABC transporter substrate-binding protein	712	1217
1218	MGCS36089_03334			*atoA*	acyl CoA:acetate/3-ketoacid CoA transferase beta	711	1218
1219	MGCS36089_00886			*greA*	transcription elongation factor GreA	710	1219
1220	MGCS36089_03410			-	DUF896 family protein	709	1220
1221	MGCS36089_03796			-	DUF1447 family protein	709	1220
1222	MGCS36089_04236			*marC*	MarC family small neutral amino acid	708	1222
1223	MGCS36089_01950			-	DUF4649 domain-containing protein	706	1223
1224	MGCS36089_02656			*malQ*	4-alpha-glucanotransferase (amylomaltase)	700	1224
1225	MGCS36089_04136			*mutS*	DNA mismatch repair protein MutS	700	1224
1226	MGCS36089_00924			-	ECF transporter S component	699	1226
1227	MGCS36089_00600			-	L13_leader RNA.	694	1227
1228	MGCS36089_02458			*ykgJ*	YkgJ family cysteine cluster protein	693	1228
1229	MGCS36089_00904			-	YfcE family metallophosphoesterase	692	1229
1230	MGCS36089_00656			-	type II toxin-antitoxin system Phd/YefM family	690	1230
1231	MGCS36089_00034			-	IS30 family transposase	689	1231
1232	MGCS36089_01826			-	nucleotidyl transferase AbiEii/AbiGii toxin	689	1231
1233	MGCS36089_02504			-	CRISPR-DR22 RNA	689	1231

No.	Locus tag	SignalP6 predicted[1]	Virulence[2]	Gene	Function	RPKMs in NHP 14[3]	RANK[4]
1234	MGCS36089_01422			*dinG*	bifunctional DnaQ family exonuclease -- ATP-dependent helicase DinG	688	1234
1235	MGCS36089_02752			-	resolvase/recombinase family protein	688	1234
1236	MGCS36089_03338			*gntT*	GntT family H+/gluconate symporter or related	688	1234
1237	MGCS36089_00136			-	DUF4041 domain-containing protein	682	1237
1238	MGCS36089_02126			*ylxM*	YlxM superfamily signal recognition particle	679	1238
1239	MGCS36089_02924			-	OFA family MFS transporter	677	1239
1240	MGCS36089_01598			*rgpA*	glycosyltransferase family 1 protein RgpA	674	1240
1241	MGCS36089_02738			-	nucleoside phosphorylase family protein	672	1241
1242	MGCS36089_03222			*trxT*	Trx TCS operon protein TrxT	670	1242
1243	MGCS36089_00008			-	helix-turn-helix domain-containing protein	665	1243
1244	MGCS36089_03246			-	ParB-like nuclease domain-containing protein	664	1244
1245	MGCS36089_00824			-	class I SAM-dependent methyltransferase	658	1245
1246	MGCS36089_03256			*thlA*	thiolase ThlA	657	1246
1247	MGCS36089_00566			-	PTS transporter IIB component UlaB-like protein	652	1247
1248	MGCS36089_01396			*kgdA*	bifunctional 4-hydroxy-2-oxoglutarate (KHG) aldolase/2-dehydro-3-deoxy-phosphogluconate (KDPG) aldolase KdgA	651	1248
1249	MGCS36089_01246			-	MFS transporter	650	1249
1250	MGCS36089_03468			-	YSIRK-targeted surface antigen transcriptional	649	1250
1251	MGCS36089_02140			-	putative TCS histidine kinase sensor	645	1251
1252	MGCS36089_03106			-	SDR family oxidoreductase	645	1251
1253	MGCS36089_03904			-	ABC transporter ATPase/permease	644	1253
1254	MGCS36089_02918			-	LURP-one-related family protein	643	1254
1255	MGCS36089_01474			*pheA*	chorismate mutase PheA	642	1255
1256	MGCS36089_00006			-	DUF951 domain-containing protein	641	1256
1257	MGCS36089_03128			*asnA*	asparagine synthetase AsnA	640	1257
1258	MGCS36089_01078			*yozE*	YozE family protein	639	1258
1259	MGCS36089_01376			*regR*	transcriptional regulator RegR	637	1259
1260	MGCS36089_02556			-	PadR family transcriptional regulator	636	1260
1261	MGCS36089_02024			*lmb_2*	bifunctional metal ABC transporter substrate-binding lipoprotein/laminin-binding lipoprotein Lmb	635	1261
1262	MGCS36089_03520			-	DUF956 family protein	633	1262
1263	MGCS36089_00828			-	MerR family transcriptional regulator	632	1263
1264	MGCS36089_00724			*truA*	tRNA pseudouridine(38-40) synthase TruA	631	1264

No.	Locus tag	SignalP6 predicted(1)	Virulence(2)	Gene	Function	RPKMs in NHP 14(3)	RANK(4)
1265	MGCS36089_00928			*yhcC*	YhcC family Fe-S oxidoreductase	631	1264
1266	MGCS36089_02302			-	inositol monophosphatase family protein	627	1266
1267	MGCS36089_03088			-	DUF910 domain-containing protein	626	1267
1268	MGCS36089_04148			-	YitT family protein putative ABC transporter	626	1267
1269	MGCS36089_02464			-	YesM family TCS sensor histidine kinase	624	1269
1270	MGCS36089_01334			*ppc*	phosphoenolpyruvate carboxylase Ppc	622	1270
1271	MGCS36089_00508			*rihB*	pyrimidine-specific ribonucleoside hydrolase	621	1271
1272	MGCS36089_02854			-	BglX family glycosyl hydrolase	621	1271
1273	MGCS36089_01882			-	integrase catalytic subunit	619	1273
1274	MGCS36089_00930			-	RsmB/NOP family class I SAM-dependent RNA	618	1274
1275	MGCS36089_01222			*glnQ_1*	glutamine ABC transporter ATPase GlnQ	618	1274
1276	MGCS36089_01914			*folQ*	dihydroneopterin aldolase protein FolB	614	1276
1277	MGCS36089_01062			-	DUF3397 domain-containing protein	609	1277
1278	MGCS36089_03922			-	IS30 family transposase	609	1277
1279	MGCS36089_01436			*pepD_1*	C69 family dipeptidase PepD	607	1279
1280	MGCS36089_01314			*yccF*	YccF domain-containing protein	606	1280
1281	MGCS36089_00402			*celA_1*	PTS transporter cellobiose-specific IIB	604	1281
1282	MGCS36089_01928			*maeR*	TCS signal transduction response regulator MaeR	603	1282
1283	MGCS36089_02494			-	CRISPR-DR22 RNA	597	1283
1284	MGCS36089_03662			-	DUF1129 domain-containing protein	591	1284
1285	MGCS36089_02300			*rsmF*	RsmF family rRNA cytosine-C5-methyltransferase	588	1285
1286	MGCS36089_03098			-	VanZ like family glycopeptide antibiotic	588	1285
1287	MGCS36089_00906			-	CBS domain-containing protein	587	1287
1288	MGCS36089_01434			*whiA*	cell division involved DNA-binding protein WhiA	586	1288
1289	MGCS36089_01876			-	DUF5960 family protein	586	1288
1290	MGCS36089_00512			*nrdI_1*	class Ib ribonucleoside-diphosphate reductase	582	1290
1291	MGCS36089_00586	Secreted		-	putative secreted protein	581	1291
1292	MGCS36089_03440			*lacA*	galactose-6-phosphate isomerase subunit LacA	581	1291
1293	MGCS36089_03806			*oppB_2*	oligopeptide ABC transporter permease OppB	581	1291
1294	MGCS36089_01454			-	PTS sugar transport IID subunit	580	1294
1295	MGCS36089_03184			-	GNAT family N-acetyltransferase	578	1295
1296	MGCS36089_03740			-	ABC transporter permease	578	1295

No.	Locus tag	SignalP6 predicted[1]	Virulence[2]	Gene	Function	RPKMs in NHP 14[3]	RANK[4]
1297	MGCS36089_01448			*bga*	beta-galactosidase Bga	573	1297
1298	MGCS36089_01684			*pyrP*	uracil permease protein PyrP	572	1298
1299	MGCS36089_00524	Secreted	Virulence	*ska*	secreted streptokinase Ska	571	1299
1300	MGCS36089_03018			*argR_1*	ArgR family transcriptional regulator	566	1300
1301	MGCS36089_01738			-	MdlB family multidrug ABC transporter ATPase and	562	1301
1302	MGCS36089_01538			*atpE*	ATP synthase C subunit AtpE	560	1302
1303	MGCS36089_03040			*feoA*	ferrous iron transport protein (A) FeoA	552	1303
1304	MGCS36089_03714			-	putative sulfite exporter	552	1303
1305	MGCS36089_00812			*yqeH*	ribosome biogenesis GTPase YqeH	550	1305
1306	MGCS36089_02480			-	sakacin P family class II bacteriocin	545	1306
1307	MGCS36089_00228			-	IS30 family transposase	544	1307
1308	MGCS36089_03810			*glnA*	glutamine synthetase GlnA	543	1308
1309	MGCS36089_03186			-	VOC family protein	541	1309
1310	MGCS36089_01446			-	GntR family transcriptional regulator	539	1310
1311	MGCS36089_00594			-	hypothetical protein	536	1311
1312	MGCS36089_00912			*scp2*	segregation/condensation complex subunit (B)	536	1311
1313	MGCS36089_01442			*agaS*	AgaS superfamily sugar isomerase SIS	536	1311
1314	MGCS36089_03508			*cof*	Cof family hydrolase	536	1311
1315	MGCS36089_03804			*oppC_2*	oligopeptide ABC transporter permease OppC	536	1311
1316	MGCS36089_02642			-	C4-dicarboxylate ABC transporter	535	1316
1317	MGCS36089_02456			*ycjU*	YcjU family beta-phosphoglucomutase or related	533	1317
1318	MGCS36089_02322			*tetR*	TetR family transcriptional regulator	530	1318
1319	MGCS36089_00590			-	NYN domain-containing protein	528	1319
1320	MGCS36089_02946	Lipo		*blaA*	beta-lactamase-related serine hydrolase	527	1320
1321	MGCS36089_02244			-	NAD-dependent deacetylase	523	1321
1322	MGCS36089_03076			*murD*	UDP-N-acetylmuramoyl-L-alanine--D-glutamate	522	1322
1323	MGCS36089_03366			-	DUF4059 family protein	522	1322
1324	MGCS36089_03784			*yhiN*	YhiN family predicted flavoprotein	519	1324
1325	MGCS36089_02604			-	hypothetical protein	517	1325
1326	MGCS36089_03712		Virulence	*cppA*	CppA family putative C3-glycoprotein degrading	515	1326
1327	MGCS36089_02538			*hemN*	HemN family coproporphyrinogen III oxidase or	511	1327
1328	MGCS36089_02760			-	MATE family efflux transporter	511	1327

No.	Locus tag	SignalP6 predicted(1)	Virulence(2)	Gene	Function	RPKMs in NHP 14(3)	RANK(4)
1329	MGCS36089_03588			-	rhodanese-like domain-containing protein	511	1327
1330	MGCS36089_03852			*rpiR*	RpiR family transcriptional regulator	507	1330
1331	MGCS36089_01380			*agaD*	PTS transporter	504	1331
1332	MGCS36089_03320			*priA*	primosomal protein PriA	503	1332
1333	MGCS36089_02372			-	TVP38/TMEM64 family protein	502	1333
1334	MGCS36089_04092			*catE*	catechol-2,3-dioxygenase CatE	501	1334
1335	MGCS36089_00020			*divIC*	septum formation initiator family protein	500	1335
1336	MGCS36089_01358			-	FMN-binding domain containing L-lactate oxidase	496	1336
1337	MGCS36089_02886			*prsW*	PrsW family glutamic-type intramembrane	494	1337
1338	MGCS36089_03898			*radA*	DNA repair protein RadA	493	1338
1339	MGCS36089_03974			*ulaF*	L-ribulose-5-phosphate 4-epimerase UlaF	493	1338
1340	MGCS36089_01828			-	type IV toxin-antitoxin system AbiEi family	490	1340
1341	MGCS36089_00452			-	MdlB family ABC transporter ATP-binding/permease	489	1341
1342	MGCS36089_01962			*nadK*	NAD kinase NadK	484	1342
1343	MGCS36089_02724			*rsuA_1*	ribosomal small subunit pseudouridine synthase	484	1342
1344	MGCS36089_01366			-	HD domain-containing phosphohydrolase	483	1344
1345	MGCS36089_02848			*spxR*	SpxR family CBS-HotDog domain-containing	483	1344
1346	MGCS36089_01660			*capA*	CapA family protein	482	1346
1347	MGCS36089_02652			-	NADPH-dependent FMN reductase	471	1347
1348	MGCS36089_04140			*argR_3*	arginine repressor ArgR	469	1348
1349	MGCS36089_03166			*cas2_2*	CRISPR-associated endonuclease Cas2	468	1349
1350	MGCS36089_01982			*pbuX*	xanthine permease PbuX	467	1350
1351	MGCS36089_03848			*tatD*	Tat protein secretion system quality control	467	1350
1352	MGCS36089_03294			*comFC*	ComFC family predicted	462	1352
1353	MGCS36089_03778			-	MerR/SoxR family transcriptional regulator	462	1352
1354	MGCS36089_02732			*fetB*	iron export ABC transporter permease subunit	461	1354
1355	MGCS36089_02726			-	putative thioesterase	460	1355
1356	MGCS36089_03812			*glnR*	glutamine synthetase transcriptional repressor	460	1355
1357	MGCS36089_03240			-	PurR/LacI family transcriptional regulator	459	1357
1358	MGCS36089_03492			*ecsB*	ABC exoprotein transporter permease EcsB	458	1358
1359	MGCS36089_00782			*sufD*	Fe-S cluster assembly protein SufD	457	1359
1360	MGCS36089_00386			-	toxic anion resistance protein, tellurite	451	1360

No.	Locus tag	SignalP6 predicted(1)	Virulence(2)	Gene	Function	RPKMs in NHP 14(3)	RANK(4)
1361	MGCS36089_01386			*ugl*	unsaturated chondroitin disaccharide hydrolase	451	1360
1362	MGCS36089_02734			*fetA*	iron export ABC transporter ATP-binding subunit	450	1362
1363	MGCS36089_04230			*mnmG*	tRNA uridine-5-carboxymethylaminomethyl(34)	450	1362
1364	MGCS36089_03980			*ulaC*	ascorbate-specific PTS transporter EIIA protein	446	1364
1365	MGCS36089_01772			*mvaS2*	hydroxymethylglutaryl-CoA synthase protein (2)	444	1365
1366	MGCS36089_00418			-	MccC family LD-carboxypeptidase	432	1366
1367	MGCS36089_02762			-	PEP-utilizing enzyme	432	1366
1368	MGCS36089_02596			*yloA*	YloA family predicted ribosome quality control	431	1368
1369	MGCS36089_02404			-	amino acid ABC transporter permease	430	1369
1370	MGCS36089_02242			*lplA_1*	lipoate--protein ligase	428	1370
1371	MGCS36089_04120			-	hypothetical protein	428	1370
1372	MGCS36089_01754			*mvaK1*	mevalonate kinase MvaK1	427	1372
1373	MGCS36089_03638			*manA*	mannose-6-phosphate isomerase ManA	427	1372
1374	MGCS36089_01068			-	hypothetical protein.	426	1374
1375	MGCS36089_02660			-	IS30 family transposase	426	1374
1376	MGCS36089_02316			*hsdS*	type I restriction endonuclease subunit S	425	1376
1377	MGCS36089_01880			*cadA*	cadmium-translocating P-type ATPase CadA	424	1377
1378	MGCS36089_00378		Virulence	*fbp*	secreted fibronectin-binding protein.	422	1378
1379	MGCS36089_00670	Pilin		-	putative lipoprotein	419	1379
1380	MGCS36089_02250			-	NADH-dependent flavin oxidoreductase	418	1380
1381	MGCS36089_00398			-	DeoR-like transcriptional regulator protein	413	1381
1382	MGCS36089_03436			*lacC*	tagatose-6-phosphate kinase LacC	413	1381
1383	MGCS36089_04196			-	Rgg/GadR/MutR family transcriptional regulator	413	1381
1384	MGCS36089_01370			-	SSRC10 RNA	412	1384
1385	MGCS36089_01856			-	hypothetical protein	411	1385
1386	MGCS36089_02936			-	MFS transporter	410	1386
1387	MGCS36089_01220	Secreted		-	glutamine ABC transporter substrate-binding protein	409	1387
1388	MGCS36089_03878		Virulence	*fasX*	FasX RNA	408	1388
1389	MGCS36089_01952			-	DUF1831 domain-containing protein	407	1389
1390	MGCS36089_01984			-	thiamine biosynthesis protein ApbE-like protein	407	1389
1391	MGCS36089_03210			-	Permease	406	1391
1392	MGCS36089_02922			*yrrM*	YrrM family O-methyltransferase	401	1392

No.	Locus tag	SignalP6 predicted[1]	Virulence[2]	Gene	Function	RPKMs in NHP 14[3]	RANK[4]
1393	MGCS36089_02162			-	BaeS family TCS histidine kinase sensor	399	1393
1394	MGCS36089_03892			-	LytTR family transcriptional regulator	399	1393
1395	MGCS36089_01730			-	DUF1149 domain-containing protein	397	1395
1396	MGCS36089_01566			*osmC*	YhfA family OsmC-related salt-stress induced	395	1396
1397	MGCS36089_00138			-	hypothetical protein	393	1397
1398	MGCS36089_00454			-	type II toxin-antitoxin system PemK/MazF family	392	1398
1399	MGCS36089_02960			-	GIY-YIG catalytic domain-containing putative	392	1398
1400	MGCS36089_03252			*caiC*	CaiC family Acyl-CoA synthetase	387	1400
1401	MGCS36089_01028			-	DUF2829 domain-containing protein	386	1401
1402	MGCS36089_02220			*pdxK*	pyridoxamine kinase PdxK	385	1402
1403	MGCS36089_02492			-	CRISPR-DR22 RNA	383	1403
1404	MGCS36089_00826			-	nucleotidyltransferase	381	1404
1405	MGCS36089_00160			*thrC*	threonine synthase ThrC	380	1405
1406	MGCS36089_00902			*rdgB*	RdgB family non-canonical purine NTP	379	1406
1407	MGCS36089_02640			-	DUF3042 family protein	379	1406
1408	MGCS36089_03248			*yncA*	YncA family L-amino acid N-acyltransferase	379	1406
1409	MGCS36089_00686			-	hypothetical protein	375	1409
1410	MGCS36089_02678	Lipo		*malX*	maltose/maltodextrin-binding lipoprotein MalX	374	1410
1411	MGCS36089_01866			*kdpB*	Potassium-transporting ATPase ATP-binding	373	1411
1412	MGCS36089_01906			*thrB*	ThrB family homoserine kinase	372	1412
1413	MGCS36089_02102			-	hypothetical protein	372	1412
1414	MGCS36089_01884			-	DUF4365 family protein	368	1414
1415	MGCS36089_01392			*rpiB*	RpiB/LacA/LacB family sugar-phosphate isomerase	367	1415
1416	MGCS36089_01768			-	HTH domain-containing putative transcriptional	365	1416
1417	MGCS36089_02152	Lipo		*apbE*	ApbE family thiamine biosynthesis lipoprotein	364	1417
1418	MGCS36089_03260			*trmA*	TrmA RNA methyltransferase	364	1417
1419	MGCS36089_03244			*mngB*	alpha-mannosidase MngB	363	1419
1420	MGCS36089_03446			-	PTS sugar transporter subunit IIA	363	1419
1421	MGCS36089_04190			-	radical SAM protein	363	1419
1422	MGCS36089_02570			-	hypothetical protein	361	1422
1423	MGCS36089_00130			*purD*	phosphoribosylamine--glycine ligase PurD	357	1423
1424	MGCS36089_03434			*lacD_2*	tagatose-bisphosphate aldolase LacD	357	1423

No.	Locus tag	SignalP6 predicted[(1)]	Virulence[(2)]	Gene	Function	RPKMs in NHP 14[(3)]	RANK[(4)]
1425	MGCS36089_03234			-	hypothetical protein	356	1425
1426	MGCS36089_01878			*arsR*	cadmium efflux system metalloregulator ArsR/SmtB	355	1426
1427	MGCS36089_03978			*ulaD*	3-keto-L-gulonate 6-phosphate decarboxylase	354	1427
1428	MGCS36089_00564			-	PTS transporter IIA component UlaC-like protein	353	1428
1429	MGCS36089_04030			*mutT*	8-oxo-dGTP diphosphatase, DNA mismatch repair	352	1429
1430	MGCS36089_04040			-	cspA RNA	346	1430
1431	MGCS36089_04232			-	NUDIX domain-containing protein	344	1431
1432	MGCS36089_04060			-	formiminotetrahydrofolate cyclodeaminase	342	1432
1433	MGCS36089_04176			-	MerR family transcriptional regulator	342	1432
1434	MGCS36089_00282			-	Spy392987 RNA	341	1434
1435	MGCS36089_01904			*hom*	homoserine dehydrogenase Hom	341	1434
1436	MGCS36089_01840			-	DUF5966 family protein	340	1436
1437	MGCS36089_01908			*folC*	dihydrofolate synthase FolC	337	1437
1438	MGCS36089_02966	Lipo		*metQ*	methionine uptake ABC transporter	336	1438
1439	MGCS36089_00588			*rmlB*	23S rRNA (guanosine(2251)-2'-O)-methyltransferase RlmB	330	1439
1440	MGCS36089_00916			*yidD*	membrane protein insertion efficiency factor	329	1440
1441	MGCS36089_02246			-	protein-ADP-ribose hydrolase	329	1440
1442	MGCS36089_00842			*dapE*	M20/M25/M40 family metallo-hydrolase,	326	1442
1443	MGCS36089_00836	Lipo		-	amino acid ABC transporter substrate-binding protein	322	1443
1444	MGCS36089_01430			*rapZ*	RNase adapter RapZ	320	1444
1445	MGCS36089_02888			-	DUF1294 domain-containing protein	320	1444
1446	MGCS36089_00396			-	GlpR-like transcriptional regulator protein	319	1446
1447	MGCS36089_03416			*ypbQ*	YpbQ family isoprenylcysteine carboxyl	319	1446
1448	MGCS36089_00784			*sufS*	cysteine desulfurase SufS	317	1448
1449	MGCS36089_03584			-	rhodanese-like domain-containing protein	315	1449
1450	MGCS36089_03864			*ugpA_2*	carbohydrate ABC transporter permease UgpA-like	315	1449
1451	MGCS36089_01444			*lacD_1*	tagatose-bisphosphate aldolase LacD-like	314	1451
1452	MGCS36089_02304			-	UPF0223 family protein	314	1451
1453	MGCS36089_01432			*cofD*	CofD/YvcK superfamily 2-phospho-L-lactate	313	1453
1454	MGCS36089_01910			*folE*	GTP cyclohydrolase I protein FolE	311	1454
1455	MGCS36089_03102			*yutD*	YutD family protein of unknown function	310	1455
1456	MGCS36089_01390			*idnO*	gluconate 5-dehydrogenase IdnO	307	1456

No.	Locus tag	SignalP6 predicted(1)	Virulence(2)	Gene	Function	RPKMs in NHP 14(3)	RANK(4)
1457	MGCS36089_02414			*pyrE*	orotate phosphoribosyltransferase PyrE	307	1456
1458	MGCS36089_03080			-	DUF3165 family protein	306	1458
1459	MGCS36089_02522			*csn2*	CRISPR-associated protein Csn2	305	1459
1460	MGCS36089_00910			*scp1*	segregation/condensation complex subunit (A)	302	1460
1461	MGCS36089_00684			-	toxin-antitoxin system, toxin component, Fic	298	1461
1462	MGCS36089_00956			-	DUF4298 domain-containing protein	298	1461
1463	MGCS36089_03908			-	MarR family transcriptional regulator	292	1463
1464	MGCS36089_01254			*bglA*	6-phospho-beta-glucosidase BglA	291	1464
1465	MGCS36089_03084			*pspE*	PspE family rhodanese-like domain-containing	289	1465
1466	MGCS36089_02902			*nrdH*	glutaredoxin-like protein NrdH	288	1466
1467	MGCS36089_01986			*pptA*	phenylpyruvate tautomerase PptA	287	1467
1468	MGCS36089_02248			*gcvH*	glycine cleavage system protein H GcvH	287	1467
1469	MGCS36089_02416			*pyrF*	orotidine-5'-phosphate decarboxylase PyrF	287	1467
1470	MGCS36089_03586			-	CoA-disulfide reductase	286	1470
1471	MGCS36089_01852			-	DUF5960 family protein	284	1471
1472	MGCS36089_04188			-	arginase family protein	284	1471
1473	MGCS36089_03636			-	IS30 family transposase	282	1473
1474	MGCS36089_03362			*ansP*	AnsP family L-asparagine transporter	280	1474
1475	MGCS36089_03494			*ecsA*	ABC exoprotein transporter ATPase EcsA	279	1475
1476	MGCS36089_03704			-	carbohydrate ABC transporter permease UgpE-like	276	1476
1477	MGCS36089_00456			-	aminoglycoside 6-adenylyltransferase	275	1477
1478	MGCS36089_03866	Lipo		*ugpB_2*	carbohydrate ABC transporter substrate-binding protein	274	1478
1479	MGCS36089_03764			-	hypothetical protein	270	1479
1480	MGCS36089_02314			-	aromatic acid exporter family protein	269	1480
1481	MGCS36089_00560			-	major facilitator transporter family protein	268	1481
1482	MGCS36089_01466			*gstA*	GstA superfamily glutathione-dependent	268	1481
1483	MGCS36089_01112		Virulence	*silA*	TCS DNA-binding response regulator SilA	267	1483
1484	MGCS36089_02478			*spiA*	sakacin P immunity protein SpiA	267	1483
1485	MGCS36089_01218			-	glutamine ABC transporter permease	263	1485
1486	MGCS36089_02252			*lipL*	lipoate--protein ligase	263	1485
1487	MGCS36089_02390			*bcaT*	branched-chain amino acid aminotransferase	263	1485
1488	MGCS36089_03208			-	hypothetical protein	260	1488

No.	Locus tag	SignalP6 predicted(1)	Virulence(2)	Gene	Function	RPKMs in NHP 14(3)	RANK(4)
1489	MGCS36089_02856			-	phosphoglycolate phosphatase	259	1489
1490	MGCS36089_01522			*queT*	Queuosine precursor transporter QueT	256	1490
1491	MGCS36089_02276			*rsmC*	class I SAM-dependent methyltransferase	256	1490
1492	MGCS36089_01252			*bglF*	PTS beta-glucoside transporter IIBCA component	253	1492
1493	MGCS36089_04186			-	MFS transporter	250	1493
1494	MGCS36089_04262			*ybcJ*	ribosome associated protein YbcJ	247	1494
1495	MGCS36089_00908			*xerD_1*	site-specific tyrosine recombinase XerD	246	1495
1496	MGCS36089_01388			*agaF*	PTS transporter	242	1496
1497	MGCS36089_02344			*fieF*	FieF family cation diffusion facilitator family	237	1497
1498	MGCS36089_04168			-	hypothetical protein	236	1498
1499	MGCS36089_03670			-	hypothetical protein	235	1499
1500	MGCS36089_00468			-	hypothetical protein	233	1500
1501	MGCS36089_02930			-	NAD(P)H-dependent oxidoreductase	233	1500
1502	MGCS36089_03862			*ugpE_2*	carbohydrate ABC transporter permease UgpE-like	233	1500
1503	MGCS36089_03660			-	CorA family divalent cation transport protein	231	1503
1504	MGCS36089_00620			-	DNA cytosine methyltransferase	229	1504
1505	MGCS36089_01774			*thyA*	thymidylate synthase ThyA	229	1504
1506	MGCS36089_04044			-	metallo-dependent amidohydrolase	228	1506
1507	MGCS36089_02320			-	ABC transporter ATP-binding protein LolD-like	226	1507
1508	MGCS36089_03860			-	DUF386 family protein	224	1508
1509	MGCS36089_01858			-	XRE family HTH-type transcriptional regulator	223	1509
1510	MGCS36089_03054			-	hypothetical protein	222	1510
1511	MGCS36089_03724			-	Xre family helix-turn-helix transcriptional	221	1511
1512	MGCS36089_02364			*dnaE*	DNA polymerase III subunit alpha DnaE	220	1512
1513	MGCS36089_03224	Lipo		*ugpB_1*	glycerol-3-phosphate ABC transporter	219	1513
1514	MGCS36089_02950			*holA*	DNA polymerase III delta subunit HolA	217	1514
1515	MGCS36089_02986			*focA*	formate transporter FocA	215	1515
1516	MGCS36089_03332			*atoD*	butyrate-acetoacetate CoA-transferase alpha	214	1516
1517	MGCS36089_04260			*pqqL*	pitrilysin family predicted Zn-dependent	213	1517
1518	MGCS36089_03868			*nanE*	N-acetylmannosamine-6-phosphate 2-epimerase	212	1518
1519	MGCS36089_00110			*plsX*	phosphate acyltransferase PlsX	211	1519
1520	MGCS36089_04068			*hutH*	histidine ammonia-lyase HutH	211	1519

No.	Locus tag	SignalP6 predicted[1]	Virulence[2]	Gene	Function	RPKMs in NHP 14[3]	RANK[4]
1521	MGCS36089_02050	Secreted	Virulence	*scpA*	cell surface extracellular C5a peptidase ScpA	210	1521
1522	MGCS36089_02704			*celA_2*	PTS cellobiose transporter IIB subunit CelA	210	1521
1523	MGCS36089_01958			*yjbK*	YbjK superfamily CYTH domain-containing	208	1523
1524	MGCS36089_03842			-	hypothetical protein	208	1523
1525	MGCS36089_02150			-	1,4-dihydroxy-2-naphthoate polyprenyltransferase	206	1525
1526	MGCS36089_02576			*phnK*	PhnK family uncharacterized ABC transporter	206	1525
1527	MGCS36089_00704			-	Cof-type HAD-IIB family hydrolase	205	1527
1528	MGCS36089_02868			-	beta-D-glucuronidase	205	1527
1529	MGCS36089_01710			*trmD*	tRNA (guanosine(37)-N1)-methyltransferase TrmD	201	1529
1530	MGCS36089_00132			*purE*	5-(carboxyamino)imidazole ribonucleotide mutase	200	1530
1531	MGCS36089_01142			-	hypothetical protein	200	1530
1532	MGCS36089_01482			*rodA*	rod shape-determining protein RodA	200	1530
1533	MGCS36089_02722			-	CorA family divalent cation transport protein	197	1533
1534	MGCS36089_01980			*xpt*	xanthine phosphoribosyltransferase Xpt	196	1534
1535	MGCS36089_03926			-	hypothetical protein	196	1534
1536	MGCS36089_03472	Secreted		*aes*	Aes family secreted acetyl esterase/lipase	192	1536
1537	MGCS36089_02926			-	HAD family hydrolase	187	1537
1538	MGCS36089_02970			*metN_2*	methionine uptake ABC transporter ATP-binding	179	1538
1539	MGCS36089_02146			*xerS*	site-specific tyrosine recombinase XerS	174	1539
1540	MGCS36089_03462	Secreted		-	pilus backbone/major protein	174	1539
1541	MGCS36089_00856			*rsmG*	16S rRNA (guanine(527)-N(7))-methyltransferase	172	1541
1542	MGCS36089_04108			-	hypothetical protein	172	1541
1543	MGCS36089_00464			-	hypothetical protein	170	1543
1544	MGCS36089_00400			*celC_1*	PTS transporter cellobiose-specific IIA	168	1544
1545	MGCS36089_00460			-	helix-turn-helix transcriptional regulator	166	1545
1546	MGCS36089_01798			-	hypothetical protein	158	1546
1547	MGCS36089_01248			-	Spy490483c RNA	157	1547
1548	MGCS36089_01130			-	lactobin A/cerein 7B family class IIb	153	1548
1549	MGCS36089_03786			-	hypothetical protein	151	1549
1550	MGCS36089_02834			-	CD1845 family protein	147	1550
1551	MGCS36089_04192			-	IS982 family transposase	147	1550
1552	MGCS36089_03568			-	GlsB/YeaQ/YmgE family stress response membrane	146	1552

No.	Locus tag	SignalP6 predicted[(1)]	Virulence[(2)]	Gene	Function	RPKMs in NHP 14[(3)]	RANK[(4)]
1553	MGCS36089_03262			*recX*	recombination regulator RecX	145	1553
1554	MGCS36089_02870			-	sugar kinase	144	1554
1555	MGCS36089_03976			*ulaE*	L-xylulose 5-phosphate 3-epimerase UlaE	144	1554
1556	MGCS36089_01384			*agaV*	PTS transporter	141	1556
1557	MGCS36089_02840			*aroK*	shikimate kinase AroK	141	1556
1558	MGCS36089_02222			*norG*	PLP-dependent aminotransferase family protein	140	1558
1559	MGCS36089_01322			-	GH25 muramidase superfamily lysozyme	136	1559
1560	MGCS36089_00680			-	lanthionine synthetase C family protein	135	1560
1561	MGCS36089_02378			-	IS1182 family transposase	133	1561
1562	MGCS36089_02744			*dcm*	DNA (cytosine-5-)-methyltransferase Dcm	133	1561
1563	MGCS36089_02990			*niaR*	niacin-responsive transcriptional repressor	133	1561
1564	MGCS36089_02452			-	DUF1524 domain-containing protein	132	1564
1565	MGCS36089_00284			-	NUDIX domain-containing protein	129	1565
1566	MGCS36089_02584	Lipo		-	putative lipoprotein	129	1565
1567	MGCS36089_01272			*pstC*	phosphate ABC transporter, permease protein	128	1567
1568	MGCS36089_01886			-	SAG1252 family conjugative relaxosome accessory	127	1568
1569	MGCS36089_03576			*gatC_2*	aspartyl-tRNA(Asn) or glutamyl-tRNA(Gln)	127	1568
1570	MGCS36089_00998			-	GloA family glyoxalase/bleomycin	124	1570
1571	MGCS36089_00624			-	MvaI/BcnI family restriction endonuclease	123	1571
1572	MGCS36089_00922			-	FMN RNA	123	1571
1573	MGCS36089_02218			-	ECF transporter S component	123	1571
1574	MGCS36089_02798			-	ORF6N domain-containing protein	122	1574
1575	MGCS36089_03190			-	DUF1912 family protein	121	1575
1576	MGCS36089_01250			*bglG_1*	transcriptional antiterminator BglG	119	1576
1577	MGCS36089_03464			*sipA*	signal peptidase I SipA	119	1576
1578	MGCS36089_00434			-	hypothetical protein	116	1578
1579	MGCS36089_00478			-	site-specific integrase	115	1579
1580	MGCS36089_01804			-	hypothetical protein	115	1579
1581	MGCS36089_00666			-	Cro/CI family transcriptional regulator	114	1581
1582	MGCS36089_04082			*treB*	PTS transporter trehalose-specific EIIBC	114	1581
1583	MGCS36089_01368			-	DUF1934 domain-containing protein	113	1583
1584	MGCS36089_01562			*pheS*	phenylalanyl-tRNA synthetase alpha subunit PheS	113	1583

No.	Locus tag	SignalP6 predicted(1)	Virulence(2)	Gene	Function	RPKMs in NHP 14(3)	RANK(4)
1585	MGCS36089_02842			*aroA*	3-phosphoshikimate 1-carboxyvinyltransferase	112	1585
1586	MGCS36089_00476			-	DUF771 domain-containing protein	111	1586
1587	MGCS36089_02020			-	hypothetical protein.	111	1586
1588	MGCS36089_00536			-	thioredoxin family protein	110	1588
1589	MGCS36089_01580			*aspB*	aspartate aminotransferase AspB	110	1588
1590	MGCS36089_01960			-	GTP pyrophosphokinase family protein	110	1588
1591	MGCS36089_02194			*dprA*	DNA-processing protein DprA	110	1588
1592	MGCS36089_01838			-	DUF5962 family protein	109	1592
1593	MGCS36089_01682			*pyrR*	bifunctional pyrimidine regulatory protein/uracil phosphoribosyltransferase PyrR	108	1593
1594	MGCS36089_04066			*potE*	PotE family amino acid transporter	108	1593
1595	MGCS36089_02832			-	DinB family protein	106	1595
1596	MGCS36089_00348			-	DUF4651 domain-containing protein	105	1596
1597	MGCS36089_01410			*fbp2*	fructose-bisphosphatase Fbp2	105	1596
1598	MGCS36089_04170			-	FtsK/SpoIIIE domain-containing protein	105	1596
1599	MGCS36089_02170			-	S41 family peptidase	104	1599
1600	MGCS36089_03204	Lipo		-	putative lipoprotein	103	1600
1601	MGCS36089_01912			*folP*	dihydropteroate synthase protein FolP	102	1601
1602	MGCS36089_02318			-	ABC transporter permease	102	1601
1603	MGCS36089_00162			-	MATE family multidrug efflux transporter	101	1603
1604	MGCS36089_01264			-	cupin domain-containing protein	101	1603
1605	MGCS36089_02800			-	DUF1413 domain-containing protein	101	1603
1606	MGCS36089_00270			-	hypothetical protein	99	1606
1607	MGCS36089_01930			*maeK*	TCS signal transduction sensor histidine kinase	99	1606
1608	MGCS36089_02524			*cas2_1*	CRISPR-associated endoribonuclease Cas2	99	1606
1609	MGCS36089_03448			*glpR*	GlpR family DNA-binding transcriptional	99	1606
1610	MGCS36089_03902			*queH*	epoxyqueuosine reductase QueH	99	1606
1611	MGCS36089_00628			-	ATP-binding protein	98	1611
1612	MGCS36089_00548			-	nucleotidyltransferase family protein	97	1612
1613	MGCS36089_03298			-	YIH1 family putative translation regulator	96	1613
1614	MGCS36089_00614			-	DUF5962 domain-containing protein	95	1614
1615	MGCS36089_01470			-	IS1548 family transposase	95	1614
1616	MGCS36089_01154			-	hypothetical protein	94	1616

No.	Locus tag	SignalP6 predicted[1]	Virulence[2]	Gene	Function	RPKMs in NHP 14[3]	RANK[4]
1617	MGCS36089_02226			*asd*	aspartate-semialdehyde dehydrogenase	93	1617
1618	MGCS36089_01554			-	DUF1146 domain-containing protein	92	1618
1619	MGCS36089_02446			-	HAD hydrolase family protein	92	1618
1620	MGCS36089_04202			-	NUDIX hydrolase	92	1618
1621	MGCS36089_02076			*kefB*	Kef-type K+ transporter membrane component KefB	89	1621
1622	MGCS36089_02928			-	GNAT family N-acetyltransferase	89	1621
1623	MGCS36089_02200			-	MFS transporter	88	1623
1624	MGCS36089_03706			-	carbohydrate ABC transporter permease UgpA-like	88	1623
1625	MGCS36089_02408			-	GatA family amidase	86	1625
1626	MGCS36089_01208			-	acyl dehydratase	85	1626
1627	MGCS36089_01274			*pstA*	phosphate ABC transporter, permease protein	85	1626
1628	MGCS36089_01152			-	ABC transporter ATP-binding protein	79	1628
1629	MGCS36089_04272			*yitT*	putative membrane anchor protein YitT	79	1628
1630	MGCS36089_02746			-	hypothetical protein	72	1630
1631	MGCS36089_00012			*pth*	aminoacyl-tRNA hydrolase Pth	0	1631
1632	MGCS36089_00014			*trcF*	transcription-repair coupling factor TcrF	0	1631
1633	MGCS36089_00016			-	oligosaccharide flippase family protein	0	1631
1634	MGCS36089_00018			-	RNA-binding S4 domain-containing protein	0	1631
1635	MGCS36089_00108			*recO*	DNA repair protein RecO	0	1631
1636	MGCS36089_00112			*acpP_1*	acyl carrier protein AcpP	0	1631
1637	MGCS36089_00114			*purC*	phosphoribosylaminoimidazolesuccinocarboxamide	0	1631
1638	MGCS36089_00142			-	helix-turn-helix domain-containing protein	0	1631
1639	MGCS36089_00144			-	quorum-sensing system DWW-type pheromone	0	1631
1640	MGCS36089_00210			*adk*	adenylate kinase protein Adk	0	1631
1641	MGCS36089_00262			-	FRG domain-containing protein	0	1631
1642	MGCS36089_00264			*xerC*	site-specific integrase	0	1631
1643	MGCS36089_00266			-	DUF3173 domain-containing protein	0	1631
1644	MGCS36089_00268			-	Rep family plasmid replication protein	0	1631
1645	MGCS36089_00272			-	hypothetical protein	0	1631
1646	MGCS36089_00322			*comYB*	competence system type II secretion system	0	1631
1647	MGCS36089_00324	Pilin		*comYC*	competence system prepilin-type N-terminal	0	1631
1648	MGCS36089_00326	Pilin		*comYD*	competence system type II secretion system GspH	0	1631

No.	Locus tag	SignalP6 predicted[1]	Virulence[2]	Gene	Function	RPKMs in NHP 14[3]	RANK[4]
1649	MGCS36089_00328	Pilin		*comYE*	competence system protein ComYE	0	1631
1650	MGCS36089_00330	Pilin		*comYF*	competence system protein ComYF	0	1631
1651	MGCS36089_00332			*comYG*	competence system protein ComYG	0	1631
1652	MGCS36089_00338			-	helix-turn-helix transcriptional regulator	0	1631
1653	MGCS36089_00354			*ssb_1*	single-stranded DNA-binding protein Ssb	0	1631
1654	MGCS36089_00362		Virulence	*rofA*	pilus transcriptional regulator RofA	0	1631
1655	MGCS36089_00364	Secreted		-	secreted pilin backbone/major protein	0	1631
1656	MGCS36089_00366	Secreted		-	secreted pilin minor/ancillary protein	0	1631
1657	MGCS36089_00368			*srtC_1*	class C sortase SrtC	0	1631
1658	MGCS36089_00370			*srtC_2*	class C sortase SrtC	0	1631
1659	MGCS36089_00372	Secreted		-	secreted pilin minor/ancillary protein	0	1631
1660	MGCS36089_00376			*srtC_3*	class C sortase SrtC	0	1631
1661	MGCS36089_00382			*ridA*	RidA family protein	0	1631
1662	MGCS36089_00388			-	hypothetical protein	0	1631
1663	MGCS36089_00416			-	hypothetical protein	0	1631
1664	MGCS36089_00420			-	hypothetical protein	0	1631
1665	MGCS36089_00422			*proX*	prolyl-tRNA synthetase associated	0	1631
1666	MGCS36089_00430			-	ATP-binding cassette domain-containing protein	0	1631
1667	MGCS36089_00436			-	Maf family protein	0	1631
1668	MGCS36089_00438			-	bacteriocin immunity protein	0	1631
1669	MGCS36089_00446			-	GNAT family N-acetyltransferase	0	1631
1670	MGCS36089_00462	Secreted		-	cell surface PgrA surface exclusion. Cell-wall anchoring predicted sortase	0	1631
1671	MGCS36089_00466			-	hypothetical protein	0	1631
1672	MGCS36089_00470			-	hypothetical protein	0	1631
1673	MGCS36089_00472			-	FtsK/SpoIIIE domain-containing protein	0	1631
1674	MGCS36089_00474			-	Rep family protein	0	1631
1675	MGCS36089_00514		Virulence	*mga*	M protein trans-acting positive regulator Mga	0	1631
1676	MGCS36089_00526			*lrp*	PucR family transcriptional regulator/leucine	0	1631
1677	MGCS36089_00534			-	hypothetical protein	0	1631
1678	MGCS36089_00606			-	site-specific integrase	0	1631
1679	MGCS36089_00608			-	helix-turn-helix transcriptional regulator	0	1631
1680	MGCS36089_00610			-	DNA-binding protein	0	1631

No.	Locus tag	SignalP6 predicted(1)	Virulence(2)	Gene	Function	RPKMs in NHP 14(3)	RANK(4)
1681	MGCS36089_00612			-	phage replisome organizer N-terminal	0	1631
1682	MGCS36089_00616			-	bacterial mobilization protein	0	1631
1683	MGCS36089_00618			-	transposase	0	1631
1684	MGCS36089_00622			-	helix-turn-helix transcriptional regulator	0	1631
1685	MGCS36089_00626			-	SIR2 family protein	0	1631
1686	MGCS36089_00632			-	helix-turn-helix domain-containing protein	0	1631
1687	MGCS36089_00634	Secreted		-	CHAP domain-containing protein	0	1631
1688	MGCS36089_00636			-	putative conjugal transfer protein	0	1631
1689	MGCS36089_00638			-	conjugal transfer protein	0	1631
1690	MGCS36089_00640			-	DNA segregation ATPase, conjugal transfer	0	1631
1691	MGCS36089_00642			-	conjugal transfer protein	0	1631
1692	MGCS36089_00644			-	conjugal transfer protein	0	1631
1693	MGCS36089_00646			-	conjugal transfer protein	0	1631
1694	MGCS36089_00648			-	conjugal transfer protein	0	1631
1695	MGCS36089_00650			-	putative conjugal transfer protein	0	1631
1696	MGCS36089_00652			-	replication initiation factor domain-containing	0	1631
1697	MGCS36089_00658			-	FtsK/SpoIIIE domain-containing protein	0	1631
1698	MGCS36089_00660			-	conjugal transfer protein	0	1631
1699	MGCS36089_00662			-	cytoplasmic protein	0	1631
1700	MGCS36089_00664			-	hypothetical protein	0	1631
1701	MGCS36089_00672			-	ImmA/IrrE family metallo-endopeptidase	0	1631
1702	MGCS36089_00674			-	XRE family transcriptional regulator	0	1631
1703	MGCS36089_00676			-	Bacteriocin	0	1631
1704	MGCS36089_00678			-	lantibiotic dehydratase	0	1631
1705	MGCS36089_00682			-	lantibiotic transporter	0	1631
1706	MGCS36089_00688			-	tyrosine-type site-specific	0	1631
1707	MGCS36089_00690			-	PRD domain/PTS transporter IIA domain protein	0	1631
1708	MGCS36089_00694			*gatC_1*	PTS galactitol transporter IIC component GatC	0	1631
1709	MGCS36089_00696			*gatB_1*	PTS galactitol transporter IIB component GatB	0	1631
1710	MGCS36089_00700			-	triose-phosphate isomerase	0	1631
1711	MGCS36089_00702			-	YjbQ family protein	0	1631
1712	MGCS36089_00720			*comX_1*	competence protein ComX	0	1631

No.	Locus tag	SignalP6 predicted(1)	Virulence(2)	Gene	Function	RPKMs in NHP 14(3)	RANK(4)
1713	MGCS36089_00722			-	aspartate kinase	0	1631
1714	MGCS36089_00758			-	IS1548 family transposase	0	1631
1715	MGCS36089_00788			*sufB*	Fe-S cluster assembly protein SufB	0	1631
1716	MGCS36089_00792			*dacA_2*	D-alanyl-D-alanine carboxypeptidase DacA	0	1631
1717	MGCS36089_00808			*comX_2*	competence protein ComX	0	1631
1718	MGCS36089_00838	Lipo		-	MetQ/NlpA family ABC transporter	0	1631
1719	MGCS36089_00840	Lipo		-	MetQ/NlpA family ABC transporter	0	1631
1720	MGCS36089_00914			*rluB*	ribosomal large subunit pseudouridine synthase	0	1631
1721	MGCS36089_00920			*trml*	TrmL family tRNA (cytosine34-2'-O-)-methyl	0	1631
1722	MGCS36089_00962	Lipo		*livK*	branched-chain amino acid ABC transporter	0	1631
1723	MGCS36089_00964			*livH*	branched-chain amino acid ABC transporter	0	1631
1724	MGCS36089_00966			*livM*	branched-chain amino acid ABC transporter	0	1631
1725	MGCS36089_00968			*livG*	branched-chain amino acid ABC transporter	0	1631
1726	MGCS36089_00970			*livF*	branched-chain amino acid ABC transporter	0	1631
1727	MGCS36089_00972			-	cystathionine beta-synthase (CBS)	0	1631
1728	MGCS36089_01002			*sdpI*	SdpI family immunity protein	0	1631
1729	MGCS36089_01012	Secreted		*yoaK*	putative secreted YoaK family protein	0	1631
1730	MGCS36089_01024			-	hypothetical protein	0	1631
1731	MGCS36089_01026	Secreted		-	hypothetical protein	0	1631
1732	MGCS36089_01096			-	hypothetical protein	0	1631
1733	MGCS36089_01098			-	hypothetical protein	0	1631
1734	MGCS36089_01102			-	Blp family class II bacteriocin with	0	1631
1735	MGCS36089_01108			-	IS1182 family transposase	0	1631
1736	MGCS36089_01116			-	IS1548 family transposase	0	1631
1737	MGCS36089_01120		Virulence	*silCR*	streptococcal invasion locus auto-inducing pheromone peptide SilCR	0	1631
1738	MGCS36089_01122			*silC*	streptococcal invasion locus signaling peptide	0	1631
1739	MGCS36089_01124		Virulence	*silD*	streptococcal invasion locus pheromone secretion	0	1631
1740	MGCS36089_01126		Virulence	*silE*	streptococcal invasion locus pheromone	0	1631
1741	MGCS36089_01128			-	BlpM-like bacteriocin with double-glycine leader	0	1631
1742	MGCS36089_01132			-	putative bacteriocin with double-glycine leader	0	1631
1743	MGCS36089_01134			-	putative bacteriocin with double-glycine leader	0	1631
1744	MGCS36089_01136			-	hypothetical protein	0	1631

No.	Locus tag	SignalP6 predicted(1)	Virulence(2)	Gene	Function	RPKMs in NHP 14(3)	RANK(4)
1745	MGCS36089_01138			-	CAAX amino terminal protease family protein	0	1631
1746	MGCS36089_01140			-	hypothetical protein	0	1631
1747	MGCS36089_01144	Secreted		-	secreted transglutaminase	0	1631
1748	MGCS36089_01196			*dhaQ*	DhaKLM operon coactivator DhaQ	0	1631
1749	MGCS36089_01198			*dhaS*	dihydroxyacetone kinase transcriptional	0	1631
1750	MGCS36089_01230			*rnc*	ribonuclease III Rnc	0	1631
1751	MGCS36089_01236			-	hypothetical protein. CW-Pred predicted sortase A cell-wall anchoring motif, position 247..252	0	1631
1752	MGCS36089_01238			-	IS1548 family transposase	0	1631
1753	MGCS36089_01270	Lipo		*pstS*	phosphate ABC transporter substrate-binding	0	1631
1754	MGCS36089_01276			*pstB*	phosphate ABC transporter ATP-binding protein	0	1631
1755	MGCS36089_01278			*phoU_1*	phosphate signaling complex protein PhoU	0	1631
1756	MGCS36089_01280	Secreted		-	hypothetical protein	0	1631
1757	MGCS36089_01298			-	PrtC family collagenase-like protease	0	1631
1758	MGCS36089_01344			-	hypothetical protein	0	1631
1759	MGCS36089_01346			-	hypothetical protein	0	1631
1760	MGCS36089_01348			-	glycosyltransferase	0	1631
1761	MGCS36089_01350			-	glycosyltransferase family 2 protein	0	1631
1762	MGCS36089_01378			-	oligohyaluronate lyase	0	1631
1763	MGCS36089_01404			-	IS3 family transposase	0	1631
1764	MGCS36089_01406			-	Transposase	0	1631
1765	MGCS36089_01428			-	DUF1003 domain-containing protein	0	1631
1766	MGCS36089_01490			*serB*	phosphoserine phosphatase SerB	0	1631
1767	MGCS36089_01520			-	preQ1-II RNA	0	1631
1768	MGCS36089_01568			-	YbgA family DUF1722 domain-containing protein	0	1631
1769	MGCS36089_01584			-	IS1548 family transposase	0	1631
1770	MGCS36089_01680			-	PyrR RNA	0	1631
1771	MGCS36089_01708			*rimM*	ribosome maturation factor RimM	0	1631
1772	MGCS36089_01712			*trxB_1*	NAD(P)/FAD-dependent oxidoreductase	0	1631
1773	MGCS36089_01762			-	HTH domain-containing putative transcriptional	0	1631
1774	MGCS36089_01764	Secreted		-	extracellular cell wall anchored mucin-binding. Cell-wall anchoring predicted sortase	0	1631
1775	MGCS36089_01802			-	DNA (cytosine-5-)-methyltransferase	0	1631

No.	Locus tag	SignalP6 predicted(1)	Virulence(2)	Gene	Function	RPKMs in NHP 14(3)	RANK(4)
1776	MGCS36089_01806			-	arsenate reductase	0	1631
1777	MGCS36089_01808			-	hypothetical protein	0	1631
1778	MGCS36089_01810			-	CPBP family intramembrane metalloprotease	0	1631
1779	MGCS36089_01812			-	hypothetical protein	0	1631
1780	MGCS36089_01814			*traG_1*	type IV secretory system conjugative DNA	0	1631
1781	MGCS36089_01816			-	hypothetical protein	0	1631
1782	MGCS36089_01818			*trbL*	conjugal transfer protein TrbL	0	1631
1783	MGCS36089_01820			-	PrgI family protein	0	1631
1784	MGCS36089_01822			-	AAA family ATPase	0	1631
1785	MGCS36089_01824			-	phage tail tip lysozyme	0	1631
1786	MGCS36089_01830	Secreted		-	cell surface extracellular antigen I/II family. Cell-wall anchoring predicted sortase	0	1631
1787	MGCS36089_01832			-	calcium-binding protein	0	1631
1788	MGCS36089_01834			-	hypothetical protein	0	1631
1789	MGCS36089_01836			-	SNF2-related protein	0	1631
1790	MGCS36089_01842			-	hypothetical protein	0	1631
1791	MGCS36089_01844			-	TOPRIM (topoisomerase-primase) domain-containing protein	0	1631
1792	MGCS36089_01846			-	DUF5965 family protein	0	1631
1793	MGCS36089_01848			-	DUF5945 family protein	0	1631
1794	MGCS36089_01850			-	hypothetical protein	0	1631
1795	MGCS36089_01862			*cadD_1*	CadD family cadmium resistance transporter	0	1631
1796	MGCS36089_01872			-	TIGR01906 family membrane protein	0	1631
1797	MGCS36089_01874			-	hypothetical protein	0	1631
1798	MGCS36089_01888			-	MobC family plasmid mobilization relaxosome	0	1631
1799	MGCS36089_01890			-	SAG1250 family conjugative relaxase	0	1631
1800	MGCS36089_01892			-	Cro/CI family transcriptional regulator	0	1631
1801	MGCS36089_01894			-	pemK RNA	0	1631
1802	MGCS36089_01896			-	DUF3173 domain-containing protein	0	1631
1803	MGCS36089_01916			*folK*	2-amino-4-hydroxy-6- hydroxymethyldihydropteridine pyrophosphokinase protein FolK	0	1631
1804	MGCS36089_01932			*maeP*	Citrate/malate symporter protein MaeP	0	1631
1805	MGCS36089_01934			*maeE*	NAD-dependent malic enzyme MaeE	0	1631
1806	MGCS36089_01938			*araC*	AraC family transcriptional regulator	0	1631

No.	Locus tag	SignalP6 predicted(1)	Virulence(2)	Gene	Function	RPKMs in NHP 14(3)	RANK(4)
1807	MGCS36089_01940			-	NanC-like SGNH/GDSL hydrolase family protein	0	1631
1808	MGCS36089_01976			*guaC*	guanosine 5'-monophosphate oxidoreductase GuaC	0	1631
1809	MGCS36089_01978			-	Purine RNA	0	1631
1810	MGCS36089_02018			-	vicinal oxygen chelate (VOC) family protein	0	1631
1811	MGCS36089_02072			-	IS30 family transposase	0	1631
1812	MGCS36089_02074			*femX*	FemABX-like family peptidoglycan interpeptide	0	1631
1813	MGCS36089_02082			-	DUF3173 family protein	0	1631
1814	MGCS36089_02084			-	hypothetical protein	0	1631
1815	MGCS36089_02086			-	FtsK/SpoIIIE domain-containing protein	0	1631
1816	MGCS36089_02088			-	hypothetical protein	0	1631
1817	MGCS36089_02090			-	hypothetical protein	0	1631
1818	MGCS36089_02092			-	XRE family HTH-type transcriptional regulator	0	1631
1819	MGCS36089_02094			-	hypothetical protein	0	1631
1820	MGCS36089_02096			-	hypothetical protein	0	1631
1821	MGCS36089_02098			-	hypothetical protein	0	1631
1822	MGCS36089_02100			-	hypothetical protein	0	1631
1823	MGCS36089_02104			-	helix-turn-helix domain-containing protein	0	1631
1824	MGCS36089_02106			-	hypothetical protein	0	1631
1825	MGCS36089_02108			-	hypothetical protein	0	1631
1826	MGCS36089_02110			-	Rep family protein	0	1631
1827	MGCS36089_02112			-	hypothetical protein	0	1631
1828	MGCS36089_02114			-	DUF3173 family protein	0	1631
1829	MGCS36089_02116			-	site-specific integrase	0	1631
1830	MGCS36089_02158			-	NAD(P)/FAD-dependent oxidoreductase	0	1631
1831	MGCS36089_02160			-	Gx transporter family protein	0	1631
1832	MGCS36089_02164			-	OmpR family TCS DNA-binding response regulator	0	1631
1833	MGCS36089_02166			-	ABC transporter permease	0	1631
1834	MGCS36089_02168			-	ABC transporter ATPase	0	1631
1835	MGCS36089_02174			*citC*	citrate lyase ligase CitC	0	1631
1836	MGCS36089_02176			*oadA*	oxaloacetate decarboxylase subunit alpha OadA	0	1631
1837	MGCS36089_02188			-	IS982 family transposase	0	1631
1838	MGCS36089_02214			-	Uup family ATPase components of ABC transporters	0	1631

No.	Locus tag	SignalP6 predicted(1)	Virulence(2)	Gene	Function	RPKMs in NHP 14(3)	RANK(4)
1839	MGCS36089_02278			*coaA*	type I pantothenate kinase	0	1631
1840	MGCS36089_02342			*alsT*	sodium:alanine symporter family protein	0	1631
1841	MGCS36089_02346			-	IS1182 family transposase	0	1631
1842	MGCS36089_02354			*phnA*	PnhA family zinc ribbon domain-containing	0	1631
1843	MGCS36089_02410			-	IS3 family transposase	0	1631
1844	MGCS36089_02418			-	PyrR RNA	0	1631
1845	MGCS36089_02490			-	CRISPR-DR22 RNA	0	1631
1846	MGCS36089_02526			*cas1_1*	type II CRISPR-associated endonuclease Cas1	0	1631
1847	MGCS36089_02528			*cas9*	type II CRISPR RNA-guided endonuclease Cas9	0	1631
1848	MGCS36089_02530			-	tracrRNA RNA	0	1631
1849	MGCS36089_02540			-	hypothetical protein	0	1631
1850	MGCS36089_02568			-	ABC transporter ATP-binding protein	0	1631
1851	MGCS36089_02578			-	putative ABC transporter permease component	0	1631
1852	MGCS36089_02580			-	putative ABC transporter substrate binding	0	1631
1853	MGCS36089_02590			-	hypothetical protein	0	1631
1854	MGCS36089_02594			-	hypothetical protein	0	1631
1855	MGCS36089_02620			*trmK*	tRNA (adenine(22)-N(1))-methyltransferase TrmK	0	1631
1856	MGCS36089_02638			*miaA*	tRNA (adenosine(37)-N6)-dimethylallyltransferase	0	1631
1857	MGCS36089_02672			*malD*	maltodextrin transport system permease protein	0	1631
1858	MGCS36089_02674			*malC*	maltodextrin transport system permease protein	0	1631
1859	MGCS36089_02676			*amyB*	cyclomaltodextrinase protein AmyB	0	1631
1860	MGCS36089_02696			-	hypothetical protein	0	1631
1861	MGCS36089_02698			*celB_2*	PTS cellobiose transporter IIC subunit CelB	0	1631
1862	MGCS36089_02700			-	DUF3284 domain-containing protein	0	1631
1863	MGCS36089_02702			*celC_2*	PTS cellobiose transporter IIA subunit CelC	0	1631
1864	MGCS36089_02706			*bglG_2*	transcription antiterminator BglG	0	1631
1865	MGCS36089_02710			*bglB_1*	6-phospho-beta-glucosidase BglB	0	1631
1866	MGCS36089_02712			*pnuC*	nicotinamide riboside transporter PnuC	0	1631
1867	MGCS36089_02718			-	DUF4044 domain-containing protein	0	1631
1868	MGCS36089_02728			-	MFS transporter	0	1631
1869	MGCS36089_02736			*paaI*	PaaI family thioesterase	0	1631
1870	MGCS36089_02748			-	RepA N-terminal domain-containing protein	0	1631

No.	Locus tag	SignalP6 predicted(1)	Virulence(2)	Gene	Function	RPKMs in NHP 14(3)	RANK(4)
1871	MGCS36089_02750			-	hypothetical protein. 1333251..1384384. Region of difference 36089_ROD.7, putative MGE	0	1631
1872	MGCS36089_02754			-	hypothetical protein	0	1631
1873	MGCS36089_02756			-	pemK RNA	0	1631
1874	MGCS36089_02758			-	sigma-70 family RNA polymerase sigma factor like	0	1631
1875	MGCS36089_02764			-	TetR/AcrR family transcriptional regulator	0	1631
1876	MGCS36089_02766			*mobC*	plasmid mobilization relaxosome protein MobC	0	1631
1877	MGCS36089_02768			-	Relaxase	0	1631
1878	MGCS36089_02770			-	conjugative element protein	0	1631
1879	MGCS36089_02772			-	DNA methyltransferase	0	1631
1880	MGCS36089_02776			*topB*	DNA topoisomerase III TopB	0	1631
1881	MGCS36089_02778	Secreted		-	DUF4366 domain-containing predicted secreted	0	1631
1882	MGCS36089_02780			-	conjugal transfer protein	0	1631
1883	MGCS36089_02782			-	CHAP domain-containing protein	0	1631
1884	MGCS36089_02784			*virB4*	type IV secretory pathway component VirB4	0	1631
1885	MGCS36089_02786			-	conjugal transfer protein	0	1631
1886	MGCS36089_02788			*virB6*	VirB6-like conjugal transfer protein	0	1631
1887	MGCS36089_02790			-	Maff2 family protein	0	1631
1888	MGCS36089_02792			-	single-stranded DNA-binding protein	0	1631
1889	MGCS36089_02794			*traG_2*	conjugal transfer protein TraG	0	1631
1890	MGCS36089_02796			*tnpW*	TnpW family transposon-encoded protein	0	1631
1891	MGCS36089_02802			-	phage replisome organizer N-terminal	0	1631
1892	MGCS36089_02804			*mobA*	MobA/MobL family protein	0	1631
1893	MGCS36089_02806			-	DUF3847 domain-containing protein	0	1631
1894	MGCS36089_02808			-	sigma-70 family RNA polymerase sigma factor like	0	1631
1895	MGCS36089_02810			-	MdlB family multidrug ABC transporter ATPase and	0	1631
1896	MGCS36089_02812			-	MdlB family multidrug ABC transporter ATPase and	0	1631
1897	MGCS36089_02814			*ecfA2*	EcfA2 family ECF transporter ATPase	0	1631
1898	MGCS36089_02816			*ecfT*	ECF transporter transmembrane protein EcfT	0	1631
1899	MGCS36089_02818			-	ECF transporter S component	0	1631
1900	MGCS36089_02820			-	TetR/AcrR family transcriptional regulator	0	1631
1901	MGCS36089_02822			-	site-specific serine recombinase family protein	0	1631
1902	MGCS36089_02824			*virD4*	type IV secretory system conjugative DNA	0	1631

No.	Locus tag	SignalP6 predicted(1)	Virulence(2)	Gene	Function	RPKMs in NHP 14(3)	RANK(4)
1903	MGCS36089_02826			-	DUF3801 domain-containing protein	0	1631
1904	MGCS36089_02828			-	NTPase domain-containing protein	0	1631
1905	MGCS36089_02830			-	RepA N-terminal domain-containing protein	0	1631
1906	MGCS36089_02850			-	GNAT family N-acetyltransferase	0	1631
1907	MGCS36089_02872			*melB*	MelB family MFS transporter	0	1631
1908	MGCS36089_02934			*coiA*	competence protein CoiA	0	1631
1909	MGCS36089_02938			*rsuA_2*	ribosomal small subunit pseudouridine synthase	0	1631
1910	MGCS36089_02952			*comEC*	DNA internalization-related competence protein	0	1631
1911	MGCS36089_02954			*comEA*	DNA uptake competence protein ComEA	0	1631
1912	MGCS36089_02958			*trmN6*	tRNA1(Val) A37 N6-methylase TrmN6	0	1631
1913	MGCS36089_02978			*murF*	UDP-N-acetylmuramoyl-tripeptide--D-alanyl-D-	0	1631
1914	MGCS36089_02994			-	IS982 family transposase	0	1631
1915	MGCS36089_03002			-	Rgg/GadR/MutR family transcriptional regulator	0	1631
1916	MGCS36089_03004			-	MFS transporter	0	1631
1917	MGCS36089_03036			-	FeoB associated cysteine-rich protein	0	1631
1918	MGCS36089_03078			-	putative secreted protein.	0	1631
1919	MGCS36089_03092			-	hypothetical protein	0	1631
1920	MGCS36089_03104			-	IS1548 family transposase	0	1631
1921	MGCS36089_03168			*cas1_2*	type I-C CRISPR-associated endonuclease Cas1	0	1631
1922	MGCS36089_03170			*cas4*	CRISPR-associated protein Cas4	0	1631
1923	MGCS36089_03172			*cas7*	type I-C CRISPR-associated protein Cas7/Csd2	0	1631
1924	MGCS36089_03174			*cas8*	type I-C CRISPR-associated protein Cas8c/Csd1	0	1631
1925	MGCS36089_03176			*cas5*	type I-C CRISPR-associated protein Cas5	0	1631
1926	MGCS36089_03178			*cas3*	CRISPR-associated helicase Cas3	0	1631
1927	MGCS36089_03192			-	MarR family winged helix-turn-helix	0	1631
1928	MGCS36089_03194			-	MOP/MATE family multidrug-resistance efflux	0	1631
1929	MGCS36089_03226			*ugpE_1*	glycerol-3-phosphate ABC transporter permease	0	1631
1930	MGCS36089_03228			*ugpA_1*	sugar ABC transporter permease UgpA	0	1631
1931	MGCS36089_03230			*nagC*	NagC family sugar kinase	0	1631
1932	MGCS36089_03232			-	COG3537 superfamily putative	0	1631
1933	MGCS36089_03242			-	metal-independent alpha-mannosidase	0	1631
1934	MGCS36089_03296			*comFA*	ComFA family DNA/RNA helicase	0	1631

No.	Locus tag	SignalP6 predicted(1)	Virulence(2)	Gene	Function	RPKMs in NHP 14(3)	RANK(4)
1935	MGCS36089_03328			-	LysR family transcriptional regulator	0	1631
1936	MGCS36089_03330			*atoB*	3-ketoacyl-CoA thiolase/acetyl-CoA	0	1631
1937	MGCS36089_03350			*drpA*	DNA processing protein (A) DprA	0	1631
1938	MGCS36089_03372	Secreted		*hisJ*	HisJ family amino acid ABC transporter	0	1631
1939	MGCS36089_03456			-	IS982 family transposase	0	1631
1940	MGCS36089_03466	Secreted		-	pilus ancillary/minor protein 1	0	1631
1941	MGCS36089_03512			-	SSRC34_1 RNA	0	1631
1942	MGCS36089_03522			-	acyltransferase family protein	0	1631
1943	MGCS36089_03640			*scrK*	fructokinase protein ScrK	0	1631
1944	MGCS36089_03644			*scrB*	sucrose-6-phosphate hydrolase ScrB	0	1631
1945	MGCS36089_03698			-	alpha-glycosidase	0	1631
1946	MGCS36089_03722			-	Xre family helix-turn-helix transcriptional regulator. Region of difference 36089_ROD.8, putative MGE	0	1631
1947	MGCS36089_03754			-	hypothetical protein	0	1631
1948	MGCS36089_03760			-	hypothetical protein	0	1631
1949	MGCS36089_03762			-	HTH cro/C1-type domain-containing protein	0	1631
1950	MGCS36089_03766			-	type IV secretion system DNA-binding	0	1631
1951	MGCS36089_03768			-	hypothetical protein	0	1631
1952	MGCS36089_03770			-	replication protein	0	1631
1953	MGCS36089_03772			-	DUF3173 family protein	0	1631
1954	MGCS36089_03780			*dnaQ*	DNA polymerase III epsilon subunit DnaQ	0	1631
1955	MGCS36089_03792			*rimI*	ribosomal-protein-alanine N-acetyltransferase	0	1631
1956	MGCS36089_03814			-	YgaE family uncharacterized protein	0	1631
1957	MGCS36089_03850			-	cysteine hydrolase	0	1631
1958	MGCS36089_03856			*nanA*	N-acetylneuraminate lyase	0	1631
1959	MGCS36089_03858			-	DUF624 domain-containing protein	0	1631
1960	MGCS36089_03888			-	MefA-related MFS transporter	0	1631
1961	MGCS36089_03920	Secreted	Virulence	*fbpB*	cell surface fibronectin binding protein (B). Cell-wall anchoring predicted sortase	0	1631
1962	MGCS36089_03928			-	hypothetical protein	0	1631
1963	MGCS36089_03934			*resA*	TlpA family protein disulfide reductase	0	1631
1964	MGCS36089_03972			*bglG_3*	BglG family transcription antiterminator	0	1631
1965	MGCS36089_03984			*ulaA*	ascorbate-specific PTS transporter EIIC protein	0	1631

No.	Locus tag	SignalP6 predicted(1)	Virulence(2)	Gene	Function	RPKMs in NHP 14(3)	RANK(4)
1966	MGCS36089_03988			-	IS1548 family transposase	0	1631
1967	MGCS36089_03996	Secreted		-	putative secreted protein	0	1631
1968	MGCS36089_03998			-	MdlB family ABC transporter ATP-binding/permease	0	1631
1969	MGCS36089_04000			-	hypothetical protein	0	1631
1970	MGCS36089_04002			-	hypothetical protein	0	1631
1971	MGCS36089_04004			-	IS982 family transposase	0	1631
1972	MGCS36089_04006	Secreted		-	TrbC/VirB2-related secreted effector protein	0	1631
1973	MGCS36089_04022			-	hypothetical protein	0	1631
1974	MGCS36089_04056			*hutU*	urocanate hydratase HutU	0	1631
1975	MGCS36089_04058			*fctD*	glutamate formiminotransferase FctD	0	1631
1976	MGCS36089_04062			*fhs_2*	formate--tetrahydrofolate ligase Fhs	0	1631
1977	MGCS36089_04064			*hutD*	histidine uptake and utilization HutD	0	1631
1978	MGCS36089_04080			*treC*	trehalose-6-phosphate hydrolase TreC	0	1631
1979	MGCS36089_04084			*treR*	trehalose operon repressor TreR	0	1631
1980	MGCS36089_04144			-	Uup family of ABC transporter with duplicated	0	1631
1981	MGCS36089_04160			*cadD_2*	CadD family cadmium resistance transporter	0	1631
1982	MGCS36089_04162			-	cadmium efflux system accessory protein	0	1631
1983	MGCS36089_04164			-	DNA-binding HTH domain-containing	0	1631
1984	MGCS36089_04166			-	sigma-70 family RNA polymerase sigma factor	0	1631
1985	MGCS36089_04172			-	hypothetical protein	0	1631
1986	MGCS36089_04174			-	replication protein	0	1631
1987	MGCS36089_04184			-	parvulin-like peptidyl-prolyl isomerase	0	1631
1988	MGCS36089_04204			*padR*	PadR family transcriptional regulator	0	1631
1989	MGCS36089_04206			-	DUF1700 domain-containing protein	0	1631
1990	MGCS36089_04208			-	DUF4097 family protein	0	1631
1991	MGCS36089_04210			-	YhgE/Pip domain-containing protein	0	1631
1992	MGCS36089_04212			-	hypothetical protein	0	1631
1993	MGCS36089_04214			-	IS1182 family transposase	0	1631
1994	MGCS36089_04218			-	helix-turn-helix domain-containing	0	1631
1995	MGCS36089_04228			-	IS1548 family transposase	0	1631
1996	MGCS36089_04234			-	Spd-sr37 RNA	0	1631
1997	MGCS36089_04258			*pqqF*	pitrilysin family predicted Zn-dependent	0	1631

MGCS36089 was recovered from NHP 14 biopsies. 1,630 genes had mapped transcript reads whereas 367 genes did not.

(1) Genes predicted by SignalP 6.0 to have an export signal sequence
(2) Virulence refers to putative virulence genes
(3) RPKMs were assigned by EDGEPro
(4) Rank refers to the corresponding transcript abundance rank based on RPKMs
(5) Lipo, exported lipoprotein attached to the cell envelope
(6) TCS, two-component system

Table S5A. MGCS36044 virulence genes with mapped reads in six NHPs

NHP 15[1]			NHP 13			NHP 14			NHP 11			NHP 16			NHP 9		
No.[2]	Gene[3]	RANK[4]	No.	Gene	RANK	No.	Gene	RANK	No.	Gene	RANK	No.	Gene	RANK	No.	Gene	RANK
1	***sagA_RNA***	4	1	***sagA_RNA***	4	1	***sagA_RNA***	4	1	***sagA_RNA***	4	1	***sagA_RNA***	4	1	***sagA_RNA***	4
2	***sagA***	8	2	***isp***	7	2	***sagA***	9	2	***sagA***	7	2	***isp***	8	2	***sagA***	6
3	***sagF***	10	3	***sagA***	9	3	***isp***	13	3	***sagF***	9	3	***sagF***	10	3	***isp***	11
4	***sagE***	12	4	***sagE***	11	4	***sagF***	15	4	***isp***	11	4	***sagE***	12	4	***sagF***	12
5	***isp***	14	5	***sagF***	13	5	***gapA***	17	5	***sagE***	13	5	***sagA***	13	5	***sagE***	16
6	***sagH***	15	6	***sagH***	17	6	***sagE***	20	6	***sagH***	15	6	***sagH***	15	6	***gapA***	17
7	***sagG***	17	7	***sagG***	20	7	***sagH***	21	7	***sagD***	19	7	***sagG***	20	7	***sagH***	18
8	***sagB***	20	8	***sagD***	22	8	***sagB***	22	8	***sagG***	22	8	***sagB***	21	8	***sagB***	19
9	***sagD***	21	9	***sagB***	23	9	***sagG***	27	9	***sagB***	23	9	***ihk***	22	9	***sagD***	20
10	***sagI***	22	10	***ihk***	25	10	***sagD***	29	10	***gapA***	27	10	***irr***	23	10	***sagG***	23
11	***gapA***	25	11	***irr***	27	11	***irr***	33	11	***sagI***	28	11	***gapA***	25	11	***sagI***	28
12	***irr***	27	12	***gapA***	29	12	***ihk***	41	12	***irr***	30	12	***sagI***	26	12	***srrG***	33
13	***ihk***	31	13	***sagI***	30	13	***srrG***	42	13	***ihk***	38	13	***sagD***	27	13	***sagC***	34
14	***sagC***	36	14	***sagC***	34	14	***sagI***	51	14	***sagC***	40	14	***sagC***	35	14	***ihk***	37
15	***emm***	63	15	***isp2***	65	15	***sagC***	65	15	***emm***	48	15	***emm***	53	15	***irr***	38
16	***srrG***	69	16	***srrG***	77	16	***emm***	84	16	***spg***	72	16	***isp2***	65	16	***emm***	46
17	***ska***	75	17	***perR***	85	17	***perR***	102	17	***srrG***	92	17	***srrG***	71	17	***spg***	90
18	***spg***	100	18	***ska***	96	18	***isp2***	103	18	***Isp2***	119	18	***perR***	78	18	***isp2***	97
19	***perR***	105	19	***ciaH***	117	19	***ska***	114	19	***htrA***	122	19	***spg***	95	19	***perR***	139
20	***isp2***	107	20	***ciaR***	127	20	***spg***	132	20	***ciaR***	125	20	***ciaR***	124	20	***ska***	161
21	***ciaR***	125	21	***emm***	134	21	***htrA***	135	21	***perR***	144	21	***htrA***	142	21	***ciaR***	162
22	***ciaH***	129	22	***htrA***	151	22	***ciaR***	194	22	***ska***	145	22	***ska***	145	22	***htrA***	188
23	***htrA***	131	23	***spg***	163	23	***ciaH***	240	23	***ciaH***	153	23	***ciaH***	170	23	***ciaH***	198
24	***covR***	158	24	***slr***	183	24	***covR***	246	24	***covR***	214	24	***covR***	196	24	***covR***	232
25	***yesN***	278	25	***covR***	230	25	***Slr***	248	25	***yesM***	253	25	***slr***	228	25	***fasX***	243
26	***mtsR***	281	26	***yesM***	314	26	***mtsR***	283	26	***yesN***	324	26	***vicR***	260	26	***yesN***	262
27	***yesM***	295	27	***lmb***	336	27	***lmb***	285	27	***vicR***	378	27	***lmb***	270	27	***yesM***	275
28	***vicK***	394	28	***vicK***	422	28	***yesM***	324	28	***liaR***	440	28	***yesM***	313	28	***ccpA***	532
29	***slr***	409	29	***yesN***	497	29	***yesN***	371	29	***mtsR***	486	29	***vicK***	382	29	***covS***	538
30	***lmb***	448	30	***liaR***	512	30	***vicR***	373	30	***vicK***	509	30	***liaR***	473	**552 total**		
31	***ccpA***	463	31	***liaS***	603	31	***vicK***	389	31	***ccpA***	519	31	***fasX***	488			
32	***liaS***	531	32	***vicR***	618	32	***fasX***	400	32	***lmb***	570	32	***yesN***	606			

**NHP 15**			**NHP 13**			**NHP 14**			**NHP 11**			**NHP 16**
33	***covS***	537	33	***covS***	696	33	***ccpA***	496	33	***covS***	593	**645 total**
34	***vicR***	547	34	***trxR***	709	34	***slo***	574	34	***fasX***	622	
35	***slo***	565	35	***slo***	726	35	***shr***	640	35	***liaS***	741	
36	***liaR***	602	36	***fasX***	733	36	***hlyX***	670	**761 total**			
37	***trxR***	707	37	***hlyX***	741	37	***liaS***	704				
38	***trxS***	828	38	***mtsR***	800	38	***liaR***	709				
39	***fasX***	896	39	***ccpA***	840	39	***covS***	716				
40	***hlyX***	904	40	***fasA***	940	40	***fasC***	793				
41	***hylB***	967	41	***fasC***	948	**994 total**						
42	***nga***	994	42	***silA***	1073							
43	***pulA***	1109	43	***shr***	1092							
44	***shr***	1139	**1,103 total**									
45	***liaF***	1143										
46	***fasC***	1176										
47	***fasA***	1263										
48	***scpA***	1287										
49	***fbp***	1383										
50	***speG***	1425										
51	***cppA***	1508										
52	***silA***	1559										
53	***rofA***	1562										
54	***fasB***	1639										
**1,662 total(5)**												

The gene name *sagA_RNA* was given to the unannotated *sag* operon RNA to differentiate it from *sagA*.

(1) The six NHPs are ordered by number of MGCS36044 vir genes with mapped reads from left (highest) to right (lowest).
(2) No. refers to the number of putative virulence genes with mapped reads ranked by transcript abundance.
(3) MGCS36044 virulence gene name.
(4) Rank refers to the rank number assigned based on transcript abundance from highest (rank=1) to lowest for each NHP.
(5) Total number of MGCS36044 genes with mapped reads per NHP.

Table S5B. MGCS36089 virulence genes with mapped reads in six NHPs

NHP 14[1]			NHP 9			NHP 13			NHP 15			NHP 10			NHP 12		
No. [2]	Gene [3]	RANK [4]	No.	Gene	RANK	No.	Gene	RANK	No.	Gene	RANK	No.	Gene	RANK	No.	Gene	RANK
1	***sagA_RNA***	4	1	***sagA_RNA***	6	1	***sagA_RNA***	4	1	***sagA_RNA***	4	1	***sagA_RNA***	5	1	***sagA_RNA***	5
2	***isp***	7	2	***sagA***	9	2	***sagA***	7	2	***sagF***	9	2	***sagA***	7	2	***sagA***	7
3	***gapA***	14	3	***gapA***	11	3	***isp***	13	3	***sagA***	10	3	***isp***	12	3	***sagF***	12
4	***sagF***	16	4	***irr***	14	4	***gapA***	15	4	***sagE***	12	4	***gapA***	15	4	***isp***	13
5	***sagA***	17	5	***isp***	23	5	***sagB***	18	5	***gapA***	13	5	***sagF***	16	5	***gapA***	16
6	***irr***	18	6	***sagF***	25	6	***sagE***	19	6	***isp***	15	6	***sagE***	20	6	***sagE***	17
7	***ihk***	19	7	***sagE***	26	7	***sagF***	20	7	***sagG***	17	7	***sagH***	21	7	***sagB***	18
8	***sagE***	22	8	***sagB***	30	8	***sagD***	21	8	***sagH***	18	8	***sagB***	23	8	***sagH***	20
9	***sagH***	24	9	***sagH***	32	9	***sagH***	24	9	***sagB***	29	9	***sagI***	29	9	***sagG***	27
10	***sagG***	27	10	***ihk***	33	10	***irr***	27	10	***sagI***	31	10	***sagD***	31	10	***sagD***	31
11	***sagB***	37	11	***sagI***	36	11	***sagC***	28	11	***sagD***	35	11	***sagG***	36	11	***sagC***	33
12	***sagI***	40	12	***sagC***	38	12	***sagI***	31	12	***irr***	38	12	***irr***	37	12	***sagI***	34
13	***sagD***	45	13	***sagD***	42	13	***ihk***	32	13	***sagC***	44	13	***srrG***	41	13	***srrG***	37
14	***srrG***	51	14	***sagG***	46	14	***sagG***	33	14	***ihk***	50	14	***ihk***	42	14	***irr***	39
15	***sagC***	62	15	***srrG***	58	15	***srrG***	40	15	***srrG***	67	15	***sagC***	46	15	***ihk***	44
16	***isp2***	65	16	***emm***	59	16	***emm***	57	16	***spg***	80	16	***emm***	69	16	***perR***	59
17	***perR***	82	17	***spg***	97	17	***perR***	91	17	***htrA***	86	17	***perR***	100	17	***emm***	90
18	***spg***	109	18	***isp2***	113	18	***isp2***	99	18	***isp2***	113	18	***spg***	116	18	***spg***	92
19	***emm***	115	19	***perR***	123	19	***spg***	116	19	***ciaR***	114	19	***yesN***	124	19	***ccpA***	130
20	***slr***	177	20	***htrA***	174	20	***htrA***	171	20	***perR***	133	20	***ciaR***	125	20	***isp2***	155
21	***ciaH***	189	21	***ciaH***	193	21	***covR***	191	21	***ciaH***	136	21	***isp2***	130	21	***htrA***	183
22	***covR***	220	22	***covR***	223	22	***ciaR***	256	22	***mtsR***	168	22	***yesM***	138	22	***ciaH***	193
23	***lmb***	238	23	***ciaR***	228	23	***ccpA***	300	23	***emm***	205	23	***covR***	214	23	***covR***	215
24	***htrA***	247	24	***yesN***	307	24	***yesM***	335	24	***covR***	219	24	***ciaH***	238	24	***ciaR***	257
25	***ciaR***	256	25	***yesM***	320	25	***slr***	413	25	***ccpA***	326	25	***htrA***	249	25	***mtsR***	273
26	***yesM***	353	26	***slr***	358	26	***ciaH***	437	26	***lmb***	336	26	***vicK***	317	26	***vicK***	347
27	***vicK***	429	27	***vicK***	376	27	***vicK***	486	27	***covS***	353	27	***slo***	332	27	***covS***	389
28	***yesN***	430	28	***ccpA***	402	28	***lmb***	504	28	***vicK***	364	28	***slr***	384	28	***yesM***	399
29	***liaS***	545	29	***slo***	424	29	***mtsR***	519	29	***slr***	369	29	***ccpA***	493	29	***slr***	455
30	***vicR***	549	30	***lmb***	430	30	***slo***	603	30	***vicR***	401	30	***covS***	499	**575 total**		
31	***slo***	574	31	***covS***	440	31	***yesN***	615	31	***speG***	451	31	***liaR***	572			

NHP 14			NHP 9			NHP 13			NHP 15			NHP 10		
**No.** (2)	**Gene** (3)	**RANK** (4)	**No.**	**Gene**	**RANK**	**No.**	**Gene**	**RANK**	**No.**	**Gene**	**RANK**	**No.**	**Gene**	**RANK**
32	***mtsR***	605	32	***mtsR***	465	32	***nga***	677	32	***slo***	528	32	***hlyX***	585
33	***covS***	612	33	***vicR***	499	33	***hylB***	725	33	***liaR***	528	33	***vicR***	621
34	***liaR***	614	34	***shr***	691	34	***covS***	754	34	***yesM***	634	34	***lmb***	666
35	***ccpA***	666	35	***liaS***	712	35	***pulA***	787	35	***yesN***	667	35	***nga***	713
36	***shr***	718	36	***hylB***	766	36	***hlyX***	914	36	***shr***	711	36	***liaS***	775
37	***hlyX***	719	37	***hlyX***	774	37	***liaS***	926	37	***hlyX***	746	37	***fasA***	829
38	***fasC***	825	38	***nga***	799	38	***cppA***	958	38	***liaS***	749	**854 total**		
39	***fasA***	841	39	***liaR***	841	39	***vicR***	972	39	***liaF***	812			
40	***nga***	905	40	***trxR***	912	40	***speG***	1016	40	***hylB***	1038			
41	***speG***	973	41	***cppA***	936	41	***fbp***	1040	41	***nga***	1038			
42	***hylB***	1020	42	***fasX***	973	42	***ska***	1063	42	***silA***	1071			
43	***trxR***	1080	43	***ska***	1048	43	***liaR***	1078	43	***cppA***	1115			
44	***trxS***	1127	44	***liaF***	1081	44	***fasA***	1159	**1169 total**					
45	***liaF***	1130	45	***speG***	1091	45	***trxS***	1173						
46	***pulA***	1171	46	***fasA***	1155	46	***trxR***	1190						
47	***ska***	1299	47	***fasC***	1155	47	***shr***	1201						
48	***cppA***	1326	48	***trxS***	1201	**1232 total**								
49	***fbp***	1378	**1255 total**											
50	***fasX***	1388												
51	***silA***	1483												
52	***scpA***	1522												
**1630 total**														

(1) The six NHPs are ordered by number of MGCS36089 vir genes with mapped reads from left (highest) to right (lowest)
(2) No. refers to the number of putative virulence genes with mapped reads ranked by transcript abundance
(3) MGCS36089 virulence gene name
(4) Rank refers to the rank number assigned based on transcript abundance from highest (rank=1) to lowest for each NHP
(5) Total number of MGCS36089 genes with mapped reads per NHP

The gene name *sagA_RNA* was given to the unannotated *sag* operon RNA to differentiate it from *sagA*.

Table S6A. MGCS36044 genes with mapped reads containing an export signal sequence in six NHPs

NHP 15				NHP 13			NHP 14			NHP 11			NHP 16			NHP 9		
Locus tag	Gene	Function	RANK	Locus tag	Gene	RANK	Locus tag	Gene	RANK	Locus tag	Gene	RANK	Locus tag	Gene	RANK	Locus tag	Gene	RANK
2056*	-	TlpA-family protein	9	2056*	-	6	2056*	-	8	2056*	-	10	2056*	-	7	2056*	-	9
2052	***isp***	CHAP domain-containing immunogenic protein	14	2052	***isp***	7	2052	***isp***	13	2052	***isp***	11	2052	***isp***	8	2052	***isp***	11
3146*	***tlpA***	TlpA disulfide reductase	38	3146*	***tlpA***	32	2040*	-	23	1066*	***mtsA***	37	1066*	***mtsA***	41	3146*	***tlpA***	29
2040*	-	ABC transporter protein	46	1066*	***mtsA***	60	1066*	***mtsA***	28	3146*	***tlpA***	42	2040*	-	50	1066*	***mtsA***	45
1066*	***mtsA***	metal ABC transporter	47	2040*	-	63	3146*	***tlpA***	31	516	***emm***	48	516	***emm***	53	516	***emm***	46
3396*	-	PepSY domain-containing protein	51	3616	***isp2***	65	3396*	-	47	2040*	-	51	3146*	***tlpA***	63	3396*	-	69
516	***emm***	cell surface M protein	63	526	***ska***	96	516	***emm***	84	2840	***spg***	72	3616	***isp2***	65	2040*	-	80
526	***ska***	secreted streptokinase	75	1450	***adcA***	113	1450	***adcA***	95	3396*	-	99	2840	***spg***	95	2840	***spg***	90
1450	***adcA***	zinc ABC transporter	89	3396*	-	115	3616	***isp2***	103	3616	***isp2***	119	1450	***adcA***	126	3616	***isp2***	97
2840	***spg***	IgG-binding streptococcal protein	100	516	***emm***	134	526	***ska***	114	1450	***adcA***	139	526	***ska***	145	1450	***adcA***	110
3616	***isp2***	Isp-related CHAP domain-containing protein	107	1094	***lysM***	154	2840	***spg***	132	526	***ska***	145	104	***sibA***	153	526	***ska***	161
3098*	***rbsB***	D-ribose ABC transporter	165	2840	***spg***	163	104	***sibA***	168	392*	-	260	3396*	-	168	3098*	***rbsB***	168
3724	-	putative protein	249	2866*	***slr***	183	3724	-	189	2276*	-	277	1094	***lysM***	172	104	***sibA***	242
2076*	***dppA***	dipeptide-binding ABC transport protein	263	104	***sibA***	199	2866*	***slr***	248	2076*	***dppA***	337	518	-	189	2276*	-	273
1094	***lysM***	peptidoglycan-binding domain-containing protein	264	518	-	209	1094	***lysM***	255	1094	***lysM***	345	2866*	***slr***	228	392*	-	276
2908*	***prsA***	peptidylprolyl isomerase protein	270	2908*	***prsA***	225	3612	***shp***	258	2908*	***prsA***	368	3816	***prgA***	247	2908*	***prsA***	280
3816	***prgA***	surface exclusion domain-containing protein	277	392*	-	269	2076*	***dppA***	265	3610*	***htsA***	375	2276*	-	257	1094	***lysM***	284
3212*	***ugpB_1***	glycerol-3-phosphate ABC transporter protein	311	2276*	-	285	3816	***prgA***	271	3806*	***lppC***	377	2062*	***lmb***	270	3816	***prgA***	317
3806*	***lppC***	e(P4) family 5'-nucleotidase protein	312	3816	***prgA***	297	2062*	***lmb***	285	798*	***oppA_1***	404	2076*	***dppA***	307	518	-	414
104	***sibA***	CHAP domain-containing protein	316	2076*	***dppA***	312	2162*	-	287	3816	***prgA***	415	3724	-	311	2162*	-	428
518	-	2',3'-cyclic-nucleotide 2'-phosphodiesterase/3'-nucleotidase protein	327	2062*	***lmb***	336	2908*	***prsA***	292	2530*	***ybbR***	442	1758	-	323	3806*	***lppC***	449
392*	-	BMP family ABC transporter protein	335	2650*	***malE***	366	798*	***oppA_1***	305	104	***sibA***	474	2036	-	333	588	-	461
2276*	-	nucleoside ABC transporter protein	338	1718	***sdrD***	407	3806*	***lppC***	310	588	-	478	1718	***sdrD***	360	2650*	***malE***	467
2162*	-	FMN-binding major immunogen	353	2036	-	411	2276*	-	349	3724	-	481	392*	-	374	2036	-	477
902*	***yidC_1***	Invertase	362	2162*	-	419	902*	***yidC_1***	354	2034*	-	483	902*	***yidC_1***	378	2076*	***dppA***	497
2036	-	DUF1002 domain-containing protein	389	3806*	***lppC***	430	3610*	***htsA***	361	3098*	***rbsB***	505	2908*	***prsA***	400	798*	***oppA_1***	499

NHP 15			
Locus tag	Gene	Function	RANK
798*	***oppA_1***	oligopeptide ABC transporter protein	403
2866*	***slr***	InlA-like streptococcal leucine rich protein	409
2650*	***malE***	maltose/maltodextrin ABC transport system protein	421
588	**-**	putative protein	430
2062*	***lmb***	metal ABC transporter/laminin-binding protein	448
2530*	***ybbR***	YbbR family protein	451
2574	***aphA***	acid phosphatase	521
3612	***shp***	heme-binding protein	559
3994	***slo***	cytolysin streptolysin O	565
1758	**-**	metallophosphatase/5'-nucleotidase	582
794	***dacA_1***	D-alanyl-D-alanine carboxypeptidase	614
3610*	***htsA***	heme ABC transporter protein	625
1530	**-**	extracellular nuclease	642
4014	***pepD_2***	secreted dipeptidase	654
1718	***sdrD***	SdrD superfamily protein	664
2728*	***nlpA***	MetQ/NlpA family ABC transporter protein	716
2470*	**-**	solute-binding protein	719
2306*	***ptsS***	phosphate ABC transporter protein	741
1738*	**-**	peptidoglycan hydrolase protein	780
1072*	**-**	peptidylprolyl isomerase protein	806
3862*	***yidC_2***	YidC/Oxa1 family insertase	816
2356*	**-**	amino acid ABC transporter protein	827
3714*	**-**	chromosome assembly-related protein	830
1340*	**-**	DUF4430 domain-containing protein	834
24	**-**	class A beta-lactamase-related serine hydrolase	942
1410	***hylB***	hyaluronate lyase	967
3998	***nga***	NAD glycohydrolase	994
3930*	**-**	CYK3 family protein	998
1282*	***pstS***	phosphate ABC transporter protein	1006
2034*	**-**	streptococcal histidine triad (HIT) protein	1012

NHP 13		
Locus tag	Gene	RANK
902*	***yidC_1***	433
3724	**-**	437
1758	**-**	443
3612	***shp***	444
3098*	***rbsB***	454
3610*	***htsA***	456
2470*	**-**	464
588	**-**	482
2530*	***ybbR***	488
798*	***oppA_1***	521
1340*	**-**	615
1530	**-**	639
2356*	**-**	707
4014	***pepD_2***	718
1738*	**-**	724
3994	***slo***	726
794	***dacA_1***	743
2728*	***nlpA***	747
1912	***dacA_3***	779
3714*	**-**	793
1072*	**-**	814
3796*	***oppA_2***	827
2306*	***ptsS***	839
3930*	**-**	903
3862*	***yidC_2***	905
774	**-**	939
2416*	**-**	989
24	**-**	1049
3982	**-**	1067
1234	**-**	1079

NHP 14		
Locus tag	Gene	RANK
2530*	***ybbR***	365
392*	**-**	372
3098*	***rbsB***	382
518	**-**	419
2036	**-**	447
1758	**-**	449
1718	***sdrD***	500
2650*	***malE***	502
2574	***aphA***	558
3994	***slo***	574
1072*	**-**	623
588	**-**	624
3796*	***oppA_2***	626
3614	***shr***	640
2728*	***nlpA***	651
1340*	**-**	724
1912	***dacA_3***	739
2470*	**-**	821
2034*	**-**	826
3930*	**-**	853
24	**-**	865
794	***dacA_1***	914
774	**-**	939
1738*	**-**	953
4014	***pepD_2***	965
3862*	***yidC_2***	977
958*	***fhuD***	979

NHP 11		
Locus tag	Gene	RANK
902*	***yidC_1***	517
2036	**-**	520
2574	***aphA***	550
2062*	***lmb***	570
4014	***pepD_2***	585
1718	***sdrD***	599
2162*	**-**	605
2356*	**-**	680
3212*	***ugpB_1***	724

NHP 16		
Locus tag	Gene	RANK
2356*	**-**	469
798*	***oppA_1***	472
2530*	***ybbR***	474
3806*	***lppC***	515
2162*	**-**	577
1072*	**-**	603
2650*	***malE***	607
3610*	***htsA***	613
3862*	***yidC_2***	614
3898*	***rbsB***	639

NHP 9		
Locus tag	Gene	RANK
3610*	***htsA***	517

NHP 15			
Locus tag	Gene	Function	RANK
3446	**-**	pilus ancillary/minor protein 2	1034
2572	**-**	putative protein	1052
958*	***fhuD***	iron-hydroxamate ABC transporter protein	1083
850*	**-**	amino acid ABC transporter protein	1107
534	***pulA***	pullulanase	1109
1912	***dacA_3***	D,D-carboxypeptidase penicillin-binding protein	1111
774	**-**	ABC amino acid transporter protein	1112
3796*	***oppA_2***	oligopeptide ABC transporter protein	1130
3614	***shr***	heme-binding protein	1139
2416*	**-**	amino-acid ABC transporter protein	1159
1594*	**-**	ABC transporter protein	1171
2680*	***glnP_2***	glutamine permease	1173
776	**-**	DUF2207 domain-containing protein	1229
2944	***plsC***	1-acyl-sn-glycerol-3-phosphate acyltransferase	1273
2058	***scpA***	C5a peptidase	1287
3982	**-**	putative protein	1294
3854*	***ugpB_2***	carbohydrate ABC transporter protein	1311
2160*	***apbE***	ApbE family thiamine biosynthesis protein	1399
672	**-**	putative protein	1401
3926	***speG***	streptococcal pyrogenic exotoxin G	1425
2570*	**-**	putative protein	1474
2954*	***metQ***	methionine uptake ABC transporter protein	1515
3992	**-**	TrbC/VirB2-related effector protein	1517
128	**-**	CHAP domain-containing protein	1539
1234	**-**	glutamine ABC transporter protein	1541
3460	***aes***	Aes family acetyl esterase/lipase	1545
2666*	***malX***	maltose/maltodextrin-binding protein	1599
2934*	***blaA***	beta-lactamase-related serine hydrolase protein	1628
3450	**-**	pilus backbone/major protein	1643
366	**-**	pilin minor/ancillary protein	1656

NHP 13		
Locus tag	Gene	RANK
3614	***shr***	1092

NHPs are organized from left to right by the number of genes with mapped reads, where the highest is on the left.

Locus tags are designated by their number, omitting the strain designation (MGCS36044). Asterisks follow locus tag numbers encoding lipoproteins.

Rank refers to the ranking order of a gene based on the number of normalized counts, where a rank=1 corresponds to the gene with the highest amount.

For each NHP, the total number of MGCS36044 genes identified is followed (/) by the number of genes containing an export signal sequence found within the total number:

NHP 15: 1,662 total/86

NHP 13: 1,103 total/57

NHP 14: 994 total/53

NHP 11: 761 total/35

NHP 16: 645 total/36

NHP 9: 552 total/27

Table S6B. MGCS36089 genes with mapped reads containing an export signal sequence in six NHPs

NHP 14				NHP 13			NHP 9			NHP 15			NHP 10			NHP 12		
Locus tag	Gene	Function	RANK	Locus tag	Gene	RANK	Locus tag	Gene	RANK	Locus tag	Gene	RANK	Locus tag	Gene	RANK	Locus tag	Gene	RANK
2048*	-	TlpA-family protein	6	2044	*isp*	13	2048*	-	10	2048*	-	8	2048*	-	11	2044	*isp*	13
2044	*isp*	CHAP domain-containing immunogenic protein	7	2048*	-	14	2044	*isp*	23	2044	*isp*	15	2044	*isp*	12	2048*	-	23
3736	-	putative protein	33	3736	-	25	3736	-	24	1052*	*mtsA*	27	3736	-	14	3736	-	46
1052*	*mtsA*	metal ABC transporter protein	35	1052*	*mtsA*	43	3158*	*tlpA*	47	2032*	-	45	3158*	*tlpA*	17	1052*	*mtsA*	47
3158*	*tlpA*	TlpA family disulfide reductase	39	3158*	*tlpA*	50	3408*	-	51	1438	*adcA*	68	1052*	*mtsA*	38	2032*	-	78
2032*	-	ABC transporter protein	47	516	*emm*	57	1052*	*mtsA*	55	3736	-	77	516	*emm*	69	3110*	*rbsB*	81
3628	*isp2*	Isp-related CHAP domain-containing immunogenic protein	65	3408*	-	59	516	*emm*	59	2852	*spg*	80	3408*	-	91	3158*	*tlpA*	86
1438	*adcA*	zinc ABC transporter protein	75	3628	*isp2*	99	2032*	-	90	3158*	*tlpA*	88	1438	*adcA*	102	516	*emm*	90
3408*	-	PepSY domain-containing protein	88	2852	*spg*	116	1438	*adcA*	94	3408*	-	89	2032*	-	106	2852	*spg*	92
2852	*spg*	IgG-binding streptococcal protein	109	2662*	*malE*	144	2852	*spg*	97	104	*sibA*	93	2852	*spg*	116	3408*	-	93
516	*emm*	cell surface M protein	115	3110*	*rbsB*	152	3628	*isp2*	113	3628	*isp2*	113	3628	*isp2*	130	1438	*adcA*	117
1080	*lysM*	peptidoglycan-binding domain-containing protein	154	1438	*adcA*	153	3110*	*rbsB*	144	2662*	*malE*	165	3726*	-	165	3628	*isp2*	155
104	*sibA*	CHAP domain-containing protein	160	2032*	-	181	104	*sibA*	167	2920*	*prsA*	190	104	*sibA*	185	104	*sibA*	191
2878*	*slr*	InlA-like streptococcal leucine rich protein	177	2154*	-	222	3828	*prgA*	185	516	*emm*	205	3110*	*rbsB*	226	1080	*lysM*	206
3828	*prgA*	surface exclusion domain-containing protein	209	3828	*prgA*	260	2920*	*prsA*	190	2068*	*dppA*	227	3828	*prgA*	237	2028	-	230
2920*	*prsA*	peptidylprolyl isomerase protein	224	794*	*oppA_1*	263	2662*	*malE*	217	3818*	*lppC*	232	2154*	-	255	2662*	*malE*	233
2054*	*lmb*	metal ABC transporter/laminin-binding protein	238	104	*sibA*	268	2154*	-	218	2588	*aphA*	242	794*	*oppA_1*	265	2068*	*dppA*	242
1706	*sdrD*	SdrD superfamily protein	241	3866*	*ugpB_2*	296	2268*	-	221	2154*	-	248	1080	*lysM*	272	3828	*prgA*	256
2154*	-	FMN-binding major immunogen	242	3818*	*lppC*	302	794*	*oppA_1*	238	3624	*shp*	264	888*	*yidC_1*	282	3224*	*ugpB_1*	259
888*	*yidC_1*	insertase	268	1080	*lysM*	304	888*	*yidC_1*	248	2268*	-	274	2028	-	286	3622*	*htsA*	261
3110*	*rbsB*	D-ribose ABC transporter	271	888*	*yidC_1*	306	2068*	*dppA*	255	888*	*yidC_1*	283	2068*	*dppA*	287	2268*	-	341
794*	*oppA_1*	oligopeptide ABC transporter protein	277	586	-	314	3818*	*lppC*	276	794*	*oppA_1*	315	2920*	*prsA*	291	3818*	*lppC*	342
2068*	*dppA*	dipeptide-binding ABC transport protein	288	2920*	*prsA*	329	2028	-	331	2054*	*lmb*	336	2662*	*malE*	305	888*	*yidC_1*	343
3726*	-	chromosome assembly-related protein	301	2068*	*dppA*	337	3726*	-	344	3622*	*htsA*	354	3818*	*lppC*	319	794*	*oppA_1*	345
2268*	-	nucleoside ABC transporter protein	331	2268*	-	402	2878*	*slr*	358	2878*	*slr*	369	2268*	-	320	2920*	*prsA*	408
1746	-	metallophosphatase/ 5'-nucleotidase	377	2878*	*slr*	413	1080	*lysM*	373	2028	-	380	4008	*slo*	332	1706	*sdrD*	413
3818*	*lppC*	e(P4) family 5'-nucleotidase protein	399	2678*	*malX*	461	4008	*slo*	424	3828	*prgA*	398	3942*	-	342	3874*	*yidC_2*	419
3624	*shp*	heme-binding protein	423	2586	-	493	2054*	*lmb*	430	2544*	*ybbR*	434	2878*	*slr*	384	836*	-	431
2662*	*malE*	maltose/maltodextrin ABC transport system protein	424	2054*	*lmb*	504	3624	*shp*	432	3938	*speG*	451	2026*	-	438	3726*	-	434
3622*	*htsA*	heme ABC transporter protein	428	3622*	*htsA*	522	2544*	*ybbR*	463	3110*	*rbsB*	497	2740*	*nlpA*	460	392*	-	450
392*	-	BMP family ABC transporter protein	436	3726*	-	579	1746	-	473	2026*	-	518	4028	*pepD_2*	509	2878*	*slr*	455

NHP 14				NHP 13			NHP 9			NHP 15			NHP 10			NHP 12		
Locus tag	Gene	Function	RANK	Locus tag	Gene	RANK	Locus tag	Gene	RANK	Locus tag	Gene	RANK	Locus tag	Gene	RANK	Locus tag	Gene	RANK
2028	**-**	DUF1002 domain-containing protein	440	790	***dacA_1***	588	3622*	***htsA***	513	24	**-**	526	2544*	***ybbR***	560	3624	***shp***	465
2544*	***ybbR***	YbbR family protein	502	4008	***slo***	603	392*	**-**	521	4008	***slo***	528	1706	***sdrD***	579	2740*	***nlpA***	519
4008	***slo***	cytolysin streptolysin O	574	3624	***shp***	607	2586	**-**	570	1080	***lysM***	571	3874*	***yidC_2***	592	2348*	**-**	519
3808*	***oppA_2***	oligopeptide ABC transporter protein	603	1518	**-**	608	2348*	**-**	572	4028	***pepD_2***	576	3624	***shp***	624	1726*	**-**	555
3942*	**-**	CYK3 family protein	656	3808*	***oppA_2***	618	3942*	**-**	597	3726*	**-**	588	2298*	***ptsS***	642			
1726*	**-**	peptidoglycan hydrolase protein	674	2028	**-**	632	2740*	***nlpA***	607	3808*	***oppA_2***	628	586	**-**	648			
2740*	***nlpA***	MetQ/NlpA family ABC transporter protein	693	1726*	**-**	637	4028	***pepD_2***	647	1582*	**-**	674	2054*	***lmb***	666			
24	**-**	class A beta-lactamase-related serine hydrolase	710	1746	**-**	667	1706	***sdrD***	649	2298*	***ptsS***	689	3622*	***htsA***	670			
3626	***shr***	heme-binding protein	718	4012	***nga***	677	3808*	***oppA_2***	668	3626	***shr***	711	4012	***nga***	713			
2298*	***ptsS***	phosphate ABC transporter protein	722	392*	**-**	708	3626	***shr***	691	1726*	**-**	713	1726*	**-**	767			
2348*	**-**	amino acid ABC transporter protein	732	1398	***hylB***	725	1058*	**-**	695	1328*	**-**	789	3808*	***oppA_2***	767			
3874*	***yidC_2***	YidC/Oxa1 family insertase	743	2348*	**-**	740	2588	***aphA***	755	392*	**-**	790	518	**-**	790			
1328*	**-**	DUF4430 domain-containing protein	756	532	***pulA***	787	1398	***hylB***	766	670	**-**	798	24	**-**	798			
518	**-**	2',3'-cyclic-nucleotide 2'-phosphodiesterase/3'-nucleotidase protein	806	2588	***aphA***	808	2026*	**-**	776	2348*	**-**	821	1746	**-**	798			
1058*	**-**	peptidylprolyl isomerase protein	827	2460*	**-**	838	1518	**-**	782	1518	**-**	834	2348*	**-**	806			
3458	**-**	pilus ancillary/minor protein 2	835	1706	***sdrD***	861	4012	***nga***	799	1746	**-**	842	1518	**-**	815			
1518	**-**	extracellular nuclease	846	1328*	**-**	884	1582*	**-**	805	2740*	***nlpA***	884	790	***dacA_1***	843			
790	***dacA_1***	D-alanyl-D-alanine carboxypeptidase	851	2544*	***ybbR***	897	1726*	**-**	806	1058*	**-**	884	2586	**-**	846			
1900	***dacA_3***	D,D-carboxypeptidase penicillin-binding protein	853	3874*	***yidC_2***	903	3458	**-**	812	3874*	***yidC_2***	931						
2460*	**-**	solute-binding protein	854	3942*	**-**	948	2692*	***glnP_2***	813	836*	**-**	931						
2586	**-**	putative protein	895	944*	***fhuD***	982	1328*	**-**	863	1706	***sdrD***	936						
4012	***nga***	NAD glycohydrolase	905	4028	***pepD_2***	1015	3874*	***yidC_2***	868	944*	***fhuD***	992						
770	**-**	amino acid ABC transporter protein	908	3938	***speG***	1016	24	**-**	874	790	***dacA_1***	1030						
2588	***aphA***	acid phosphatase	965	3472	***aes***	1021	3224*	***ugpB_1***	916	1398	***hylB***	1038						
1582*	**-**	ABC transporter protein	966	2298*	***ptsS***	1024	790	***dacA_1***	928	4012	***nga***	1038						
3938	***speG***	streptococcal pyrogenic exotoxin G	973	770	**-**	1040	836*	**-**	952	2678*	***malX***	1055						
944*	***fhuD***	iron-hydroxamate ABC transporter protein	984	524	***ska***	1063	770	**-**	1019	586	**-**	1089						
2692*	***glnP_2***	glutamine permease	1014	2406*	**-**	1080	2966*	***metQ***	1025	3996	**-**	1143						
1398	***hylB***	hyaluronate lyase	1020	1582*	**-**	1093	524	***ska***	1048									
2956	***plsC***	1-acyl-sn-glycerol-3-phosphate acyltransferase	1035	1058*	**-**	1094	3938	***speG***	1091									
3014	**-**	putative protein	1067	24	**-**	1106	3462	**-**	1120									
2026*	**-**	streptococcal histidine triad (HIT) protein	1133	2026*	**-**	1112	3866*	***ugpB_2***	1165									
128	**-**	CHAP domain-containing protein	1140	836*	**-**	1139	128	**-**	1176									
4028	***pepD_2***	secreted dipeptidase	1149	518	**-**	1139	518	**-**	1195									

Locus tag	Gene	Function	RANK
772	**-**	DUF2207 domain-containing protein	1158
532	***pulA***	pullulanase	1171
**NHP 14**			
2406*	**-**	amino-acid ABC transporter protein	1217
586	**-**	putative protein	1291
524	***ska***	streptokinase Ska	1299
2946*	***blaA***	beta-lactamase-related serine hydrolase	1320
670	**-**	putative protein	1379
1220	**-**	glutamine ABC transporter protein	1387
2678*	***malX***	maltose/maltodextrin-binding protein	1410
2152*	***apbE***	ApbE family thiamine biosynthesis protein	1417
2966*	***metQ***	methionine uptake ABC transporter protein	1438
836*	**-**	amino acid ABC transporter protein	1443
3866*	***ugpB_2***	carbohydrate ABC transporter protein	1478
3224*	***ugpB_1***	glycerol-3-phosphate ABC transporter protein	1514
2050	***scpA***	C5a peptidase	1522
3472	***aes***	Aes family acetyl esterase/lipase	1537
3462	**-**	pilus backbone/major protein	1540
2584*	**-**	putative protein	1566
3204*	**-**	putative protein	1601

Locus tag	Gene	RANK
2740*	***nlpA***	1145
3626	***shr***	1201
**NHP 13**		
2584*	**-**	1206

Locus tag	Gene	RANK
2460*	**-**	1236
3014	**-**	1236

NHPs are organized from left to right by the number of genes with mapped reads, where the highest is on the left.
Locus tags are designated by their number, omitting the strain designation (MGCS36089). Asterisks follow locus tag numbers encoding lipoproteins.
Rank refers to the ranking order of a gene based on the number of normalized counts, where a rank=1 corresponds to the gene with the highest amount.
For each NHP, the total number of MGCS36089 genes identified is followed (/) by the number of genes containing an export signal sequence found within the total number:

- **NHP 14**: 1,630 total/84
- **NHP 13**: 1,255 total/68
- **NHP 9**: 1,232 total/67
- **NHP 15**: 1,169 total/59
- **NHP 10**: 854 total/49
- **NHP 12**: 575 total/35

Table S7. Differential expression of MGCS36044 genes during *in vivo* versus *in vitro* growth at mid-exponential and early stationary phases

No.	Locus tag	Gene	Virulence[(1)]	SignalP6[(2)]	Function	Fold in vitro ME/ in vivo[(3)]	Fold in vitro ES/ in vivo[(4)]
1	MGCS36044_03776	***rpsN2***			30S ribosomal S14-2 protein RpsN2	252.0	274.0
2	MGCS36044_00484	***rarA***			replication-associated recombination protein A	166.4	79.4
3	MGCS36044_02056	**-**		Lipo[(5)]	putative lipoprotein	117.8	71.3
4	MGCS36044_00482	***ssrS***			6S RNA	99.0	11.0
5	MGCS36044_02052	***isp***	Virulence	Secreted	secreted CHAP domain-containing immunogenic protein	82.9	54.4
6	MGCS36044_02046	**-**			SalY superfamily ABC transporter permease	77.0	77.9
7	MGCS36044_02054	**-**			hypothetical protein	73.7	52.7
8	MGCS36044_00752	***lrgB***			antiholin-like protein LrgB	54.5	3.0
9	MGCS36044_00770	**-**			putative metal homeostasis protein	45.8	44.3
10	MGCS36044_02048	***irr***	Virulence		TCS[(6)] signal transduction response regulator	45.4	40.6
11	MGCS36044_03146	***tlpA***		Lipo	TlpA family protein disulfide reductase protein	42.9	42.8
12	MGCS36044_02050	***ihk***	Virulence		TCS signal transduction histidine kinase	39.6	33.7
13	MGCS36044_02216	**-**			apolipoprotein A1/A4/E family protein	38.0	3.6
14	MGCS36044_02042	***acrA***			AcrA superfamily multidrug efflux pump	37.8	39.2
15	MGCS36044_03144	***msrA/B***			Peptide methionine sulfoxide reductase	36.0	35.8
16	MGCS36044_02044	**-**			LolD superfamily ABC transporter component	34.6	36.0
17	MGCS36044_02040	**-**		Lipo	putative lipoprotein	33.2	35.0
18	MGCS36044_03148	***ccdA***			thiol-disulfide oxidoreductase	26.9	26.9
19	MGCS36044_02384	***ssrA***			transfer-messenger RNA	26.0	2.0
20	MGCS36044_01352	**-**			sigma factor regulator	24.2	9.1
21	MGCS36044_00318	**-**			DUF1033 domain-containing protein	23.5	18.3
22	MGCS36044_00450	**-**			hypothetical protein	21.5	4.3
23	MGCS36044_01020	***lctO***			L-lactate oxidase LctO	21.3	10.5
24	MGCS36044_03128	**-**			GNAT family N-acetyltransferase	21.0	-8.3
25	MGCS36044_03126	***arcB***			ornithine carbamoyltransferase ArcB	20.1	-9.7
26	MGCS36044_02066	**-**			hypothetical protein	17.7	11.1
27	MGCS36044_03122	***arcT***			M20 family metallodipeptidase ArcT	16.8	-5.0
28	MGCS36044_01350	**-**			RpoE superfamily RNA polymerase sigma subunit	16.1	8.9
29	MGCS36044_01450	***adcA***		Secreted	zinc ABC transporter protein	15.5	17.4
30	MGCS36044_02126	**-**			SpF66_sRNA	15.5	5.9
31	MGCS36044_03130	***arcA***			arginine deiminase	15.2	-9.3
32	MGCS36044_01522	***sagF***	Virulence		streptolysin S biosynthesis protein	14.4	3.0
33	MGCS36044_01032	***nrdI_2***			ribonucleotide reductase assembly protein	13.3	12.1
34	MGCS36044_03124	***arcD***			arginine/ornithine antiporter protein	12.6	-5.2
35	MGCS36044_01520	***sagE***	Virulence		streptolysin S self-immunity protein	11.9	2.9
36	MGCS36044_01512	***sagA_RNA***	Virulence		sagA RNA	11.5	-4.0
37	MGCS36044_01034	***nrdE_1***			ribonucleoside-diphosphate reductase alpha subunit	11.2	9.8
38	MGCS36044_03958	***ulaG***			L-ascorbate utilization protein (G)	11.1	10.3
39	MGCS36044_01018	***lctP***			L-lactate permease	11.0	8.3

No.	Locus tag	Gene	Virulence[1]	SignalP6[2]	Function	Fold in vitro ME/ in vivo[3]	Fold in vitro ES/ in vivo[4]
40	MGCS36044_03546	***grpE***			heat shock protein/nucleotide exchange factor	10.9	4.1
41	MGCS36044_03548	***hrcA***			heat-inducible transcriptional repressor	10.6	5.0
42	MGCS36044_03102	***rbsA***			D-ribose transporter ATPase	9.9	4.8
43	MGCS36044_04020	***groES***			co-chaperone	9.8	5.3
44	MGCS36044_01030	***nrdF_1***			ribonucleotide-diphosphate reductase beta subunit	9.3	8.3
45	MGCS36044_01526	***sagH***	Virulence		streptolysin S export permease protein	9.2	2.2
46	MGCS36044_01524	***sagG***	Virulence		streptolysin S export protein	8.9	2.3
47	MGCS36044_03036	***clpA***			ATP-dependent Clp protease ATP-binding subunit	8.8	2.0
48	MGCS36044_03098	***rbsB***		Lipo	D-ribose ABC transporter protein	8.5	2.6
49	MGCS36044_02902	-			transcriptional regulator	8.2	8.7
50	MGCS36044_01332	-			TPP riboswitch	8.1	6.4
51	MGCS36044_01510	***sagA***	Virulence		streptolysin S precursor	7.6	-4.0
52	MGCS36044_01518	***sagD***	Virulence		streptolysin S biosynthesis protein	7.6	2.0
53	MGCS36044_02070	***dppD***			dipeptide ABC transport system protein	7.3	9.4
54	MGCS36044_03396	-		Lipo	PepSY domain-containing protein	7.1	2.5
55	MGCS36044_03878	-			hypothetical protein	7.1	7.3
56	MGCS36044_01330	***thiT***			energy-coupled thiamine transporter	6.7	4.2
57	MGCS36044_03104	***rbsD***			D-ribose pyranase	6.0	3.4
58	MGCS36044_02064	***htpA***			streptococcal histidine triad (HIT)	5.9	6.5
59	MGCS36044_03138	-			MmcQ/YjbR family DNA-binding protein	5.8	5.4
60	MGCS36044_02898	-			CPBP family intramembrane metalloprotease	5.7	5.9
61	MGCS36044_02376	***yhcF***			YhcF family transcriptional regulator	5.6	2.7
62	MGCS36044_03106	***rbsK***			ribokinase	5.5	3.3
63	MGCS36044_00158	-			hypothetical protein	5.4	-4.0
64	MGCS36044_03544	***dnaK***			molecular chaperone	5.4	2.0
65	MGCS36044_02292	***ciaR***	Virulence		TCS response regulator protein	5.2	3.7
66	MGCS36044_03100	***rbsC***			D-ribose ABC transporter permease	4.9	2.2
67	MGCS36044_03118	***arcC***			carbamate kinase	4.8	-12.4
68	MGCS36044_00122	***purN***			phosphoribosylglycinamide formyltransferase	4.6	4.5
69	MGCS36044_00526	***ska***	Virulence	Secreted	secreted streptokinase	4.5	4.2
70	MGCS36044_02458	-			NAD-dependent succinate-semialdehyde dehydrogenase	4.5	-2.5
71	MGCS36044_02670	***dltC***			D-alanine--poly(phosphoribitol) ligase subunit	4.5	3.6
72	MGCS36044_02072	***dppC***			dipeptide ABC transport system permease	4.5	5.7
73	MGCS36044_02340	-			DUF2273 domain-containing protein	4.5	-2.1
74	MGCS36044_03142	***yesN***	Virulence		TCS response regulator	4.5	4.5
75	MGCS36044_03724	-		Secreted	putative protein	4.5	2.6
76	MGCS36044_01690	-			membrane-associated alkaline phosphatase	4.4	3.1
77	MGCS36044_01180	-			cell division protein FtsW-like	4.3	5.1
78	MGCS36044_00120	***purM***			phosphoribosylformylglycinamidine cyclo-ligase	4.3	4.1
79	MGCS36044_01662	***gorA***			glutathione reductase	4.3	3.4
80	MGCS36044_01316	-			NAD(P)-binding domain-containing protein	4.2	4.3
81	MGCS36044_02900	-			DUF3169 family protein	4.1	4.1

No.	Locus tag	Gene	Virulence[1]	SignalP6[2]	Function	Fold in vitro ME/ in vivo[3]	Fold in vitro ES/ in vivo[4]
82	MGCS36044_02866	***slr***	Virulence	Lipo	InlA-like streptococcal leucine rich protein	4.0	4.1
83	MGCS36044_02068	***dppF***			dipeptide ABC transport system protein	3.9	4.8
84	MGCS36044_03020	**-**			phospho-sugar mutase	3.8	3.2
85	MGCS36044_03140	***yesM***	Virulence		TCS sensor kinase	3.8	3.7
86	MGCS36044_02290	***ciaH***	Virulence		TCS sensor histidine kinase	3.7	3.2
87	MGCS36044_00494	***rsmE***			16S rRNA (uracil(1498)-N(3))-methyltransferase	3.6	2.0
88	MGCS36044_02076	***dppA***		Lipo	dipeptide-binding ABC transport system protein	3.6	5.6
89	MGCS36044_03928	**-**			Bacteria_small_SRP	3.6	3.6
90	MGCS36044_02062	***lmb***	Virulence	Lipo	bifunctional metal ABC transporter/laminin-binding protein	3.5	3.7
91	MGCS36044_01688	***rluD***			ribosomal large subunit pseudouridine synthase	3.5	3.7
92	MGCS36044_00768	**-**			SPFH domain-containing protein	3.5	2.2
93	MGCS36044_03378	***ccmA***			CcmA family multidrug ABC transporter component	3.4	3.7
94	MGCS36044_04082	***nrdG***			ribonucleoside-triphosphate reductase activating protein	3.4	3.0
95	MGCS36044_02074	***dppB***			dipeptide ABC transport system permease protein	3.3	4.0
96	MGCS36044_01300	***hprK***			HPr(Ser) kinase/phosphatase	3.3	2.3
97	MGCS36044_04096	**-**			DUF1292 domain-containing protein	3.2	3.8
98	MGCS36044_02334	**-**			Asp23/Gls24 family envelope stress response protein	3.2	-3.5
99	MGCS36044_00572	***talA***			transaldolase	3.1	-20.1
100	MGCS36044_04180	**-**			hypothetical protein	3.1	3.3
101	MGCS36044_00126	***purH***			bifunctional phosphoribosylaminoimidazolecarboxamide formyltransferase/IMP cyclohydrolase	3.0	2.8
102	MGCS36044_04002	***secE***			preprotein translocase subunit protein	3.0	4.2
103	MGCS36044_02010	***glyA***			serine hydroxymethyl transferase	2.9	3.7
104	MGCS36044_02348	**-**			glycine RNA	2.9	4.2
105	MGCS36044_01686	***lspA***			lipoprotein signal peptidase II	2.8	3.2
106	MGCS36044_02668	***dltD***			D-alanyl-lipoteichoic acid biosynthesis protein	2.8	2.2
107	MGCS36044_01712	**-**			glycerophosphodiester phosphodiesterase	2.7	2.0
108	MGCS36044_00510	**-**			PreQ1 RNA	2.7	3.9
109	MGCS36044_00118	***purF***			amidophosphoribosyltransferase	2.6	2.6
110	MGCS36044_04098	***ruvX***			Holliday junction resolvase	2.6	2.7
111	MGCS36044_04004	***pbp2A***			multimodular transpeptidase-transglycosylase penicillin-binding protein	2.6	2.9
112	MGCS36044_03376	**-**			hypothetical protein	2.6	2.8
113	MGCS36044_04276	***parB***			chromosome partitioning protein	2.6	3.5
114	MGCS36044_00148	**-**			low molecular weight phosphotyrosine protein phosphatase	2.6	2.2
115	MGCS36044_02546	**-**			hypothetical protein	2.6	2.6
116	MGCS36044_03596	***cbiO***			cobalt ECF transporter (A) ATPase component	2.6	4.0
117	MGCS36044_00496	**-**			LacI family transcriptional regulator	2.6	2.6
118	MGCS36044_01336	**-**			transposase IS116/IS110/IS902 family protein	2.6	-10.7
119	MGCS36044_03966	***ulaD***			3-keto-L-gulonate 6-phosphate decarboxylase protein	2.5	2.5
120	MGCS36044_03708	**-**			pyridoxamine 5'-phosphate oxidase family protein	2.5	2.2
121	MGCS36044_03120	**-**			IS110 family transposase	2.5	-12.1

No.	Locus tag	Gene	Virulence[(1)]	SignalP6[(2)]	Function	Fold in vitro ME/ in vivo[(3)]	Fold in vitro ES/ in vivo[(4)]
122	MGCS36044_03430	***sgcC***			galactitol-specific PTS transporter IIB subunit	2.5	-2.2
123	MGCS36044_03612	***shp***		Secreted	heme-binding secreted protein	2.4	2.7
124	MGCS36044_03994	***slo***	Virulence	Secreted	streptolysin O	2.4	2.9
125	MGCS36044_00502	-			Udk family kinase	2.4	3.4
126	MGCS36044_01684	***lysR***			LysR family transcriptional regulator	2.4	2.8
127	MGCS36044_00490	-			DUF3013 family protein	2.4	2.2
128	MGCS36044_03016	-			PfkB superfamily kinase	2.3	2.7
129	MGCS36044_00570	-			PTS transporter IIC component ascorbate-specific UlaA-like	2.3	-17.1
130	MGCS36044_01384	***mgtA***			MgtA superfamily cation-translocating P-type ATPase	2.3	3.1
131	MGCS36044_01600	***mscL***			large-conductance mechanosensitive channel protein	2.3	2.0
132	MGCS36044_01674	-			IS30 family transposase	2.3	2.2
133	MGCS36044_02676	***dltX***			teichoic acid D-Ala incorporation-associated protein	2.3	2.4
134	MGCS36044_03592	-			aldo/keto reductase	2.3	2.0
135	MGCS36044_02188	-			DUF3307 domain-containing protein	2.3	2.6
136	MGCS36044_02016	-			MdlB superfamily multidrug ABC transporter permease	2.3	2.6
137	MGCS36044_01718	***sdrD***		Secreted	SdrD superfamily cell surface extracellular protein	2.2	2.4
138	MGCS36044_00670	-			hypothetical protein	2.2	2.6
139	MGCS36044_02162	-		Lipo	FMN-binding major membrane immunogen protein	2.2	3.0
140	MGCS36044_02236	-			asd RNA	2.2	2.3
141	MGCS36044_03278	-			IS30 family transposase	2.2	2.1
142	MGCS36044_00658	-			type II toxin-antitoxin system Phd/YefM family antitoxin	2.2	2.4
143	MGCS36044_04090	***nrdD_2***			anaerobic ribonucleoside-triphosphate reductase	2.2	2.0
144	MGCS36044_02550	***acoB***			pyruvate dehydrogenase E1 component beta subunit	2.2	2.1
145	MGCS36044_02008	-			GNAT family N-acetyltransferase	2.2	2.9
146	MGCS36044_03096	-			IS110 family transposase	2.2	-11.7
147	MGCS36044_02014	-			lysozyme family protein	2.2	3.1
148	MGCS36044_01008	***arsC_1***			arsenate reductase	2.2	2.0
149	MGCS36044_02728	***nlpA***		Lipo	MetQ/NlpA family ABC transporter protein	2.2	2.1
150	MGCS36044_01876	***copY_1***			copper transport repressor	2.1	2.1
151	MGCS36044_04014	***pepD_2***		Secreted	secreted dipeptidase	2.1	-6.1
152	MGCS36044_03488	-			Cps2a family anionic cell wall polymer biosynthesis enzyme	2.1	3.1
153	MGCS36044_03962	***ulaF***			L-ribulose-5-phosphate 4-epimerase	2.1	2.1
154	MGCS36044_03880	-			LytTR family transcriptional regulator	2.1	2.2
155	MGCS36044_03610	***htsA***		Lipo	heme ABC transporter substrate-binding protein	2.1	2.4
156	MGCS36044_02548	***acoC***			dihydrolipoamide acetyltransferase	2.0	2.0
157	MGCS36044_03428	***lacA***			galactose-6-phosphate isomerase subunit	2.0	-2.2
158	MGCS36044_03432	***sgaB***			galactitol-specific PTS transporter IIC subunit	2.0	-2.0
159	MGCS36044_03602	-			CydD-related ABC transporter ATPase/permease	2.0	2.5
160	MGCS36044_04038	***ahpF***			alkyl hydroperoxide reductase F subunit	2.0	-2.8
161	MGCS36044_00656	-			Txe/YoeB family addiction module toxin	2.0	2.1
162	MGCS36044_03720	-			BaeS family TCS sensor histidine kinase	2.0	2.3

No.	Locus tag	Gene	Virulence[1]	SignalP6[2]	Function	Fold in vitro ME/ in vivo[3]	Fold in vitro ES/ in vivo[4]
163	MGCS36044_00124	***vanZ***			VanZ family putative glycopeptide antibiotic resistance protein	2.0	2.0
164	MGCS36044_02636	***-***			rhodanese-related sulfurtransferase	2.0	2.8
165	MGCS36044_01966	***nifS_2***			NifS superfamily cysteine desulfurase	-2.0	-2.3
166	MGCS36044_03000	***-***			DegV family EDD domain-containing protein	-2.0	-2.0
167	MGCS36044_03072	***pspE***			PspE family rhodanese-like domain-containing protein	-2.0	-2.7
168	MGCS36044_02438	***xapA***			XapA family purine-nucleoside phosphorylase	-2.1	-4.0
169	MGCS36044_02278	***cdd***			cytidine deaminase	-2.1	-3.3
170	MGCS36044_02022	***-***			FolA superfamily dihydrofolate reductase	-2.1	-3.8
171	MGCS36044_01968	***ribP***			ribose-phosphate pyrophosphokinase	-2.1	-2.7
172	MGCS36044_02444	***rpiA***			ribose-5-phosphate isomerase	-2.2	-2.3
173	MGCS36044_01964	***-***			DUF1831 domain-containing protein	-2.2	-2.2
174	MGCS36044_01106	***era***			GTPase	-2.2	-2.1
175	MGCS36044_01562	***atpD***			ATP synthase beta subunit	-2.2	-2.2
176	MGCS36044_01554	***atpF***			ATP synthase B subunit	-2.2	-2.8
177	MGCS36044_00920	***-***			CBS domain-containing protein	-2.3	-2.0
178	MGCS36044_03060	***ftsQ***			cell division protein FtsQ/DivIB	-2.4	-2.0
179	MGCS36044_02024	***ldh***			L-lactate dehydrogenase	-2.4	-2.3
180	MGCS36044_03682	***pflB***			formate C-acetyltransferase	-2.4	-7.4
181	MGCS36044_02996	***-***			DUF2140 domain-containing protein	-2.4	-2.1
182	MGCS36044_01928	***folK***			2-amino-4-hydroxy-6-hydroxymethyldihydropteridine pyrophosphokinase	-2.5	-2.1
183	MGCS36044_00592	***-***			NYN domain-containing protein	-2.5	-7.3
184	MGCS36044_01108	***-***			Nudix superfamily phosphohydrolase	-2.6	-2.0
185	MGCS36044_01570	***epuA***			DNA-directed RNA polymerase beta subunit	-2.6	-2.1
186	MGCS36044_02600	***rfbA***			glucose-1-phosphate thymidylyltransferase	-2.6	-2.7
187	MGCS36044_03480	***ecsB***			ABC exoprotein transporter permease	-2.7	-2.2
188	MGCS36044_01560	***atpG***			ATP synthase gamma subunit	-2.8	-2.8
189	MGCS36044_00922	***xerD_1***			site-specific tyrosine recombinase	-2.8	-2.3
190	MGCS36044_01556	***atpH***			ATP synthase delta subunit	-2.9	-3.1
191	MGCS36044_00926	***scp2***			segregation/condensation complex subunit (B)	-2.9	-2.0
192	MGCS36044_00590	***rmlB***			23S rRNA (guanosine(2251)-2'-O)-methyltransferase	-2.9	-11.7
193	MGCS36044_02886	***ptsI***			phosphoenolpyruvate--protein phosphotransferase	-3.0	-2.7
194	MGCS36044_01564	***atpC***			ATP synthase epsilon subunit	-3.0	-3.2
195	MGCS36044_00834	***-***			cysteine hydrolase	-3.0	-2.0
196	MGCS36044_03700	***cppA***	Virulence		CppA family putative C3-glycoprotein degrading proteinase	-3.1	-2.3
197	MGCS36044_01550	***atpE***			ATP synthase C subunit	-3.1	-2.4
198	MGCS36044_03804	***pgk***			phosphoglycerate kinase	-3.1	-2.2
199	MGCS36044_00832	***yqeK***			bis(5'-nucleosyl)-tetraphosphatase (symmetrical)	-3.3	-2.5
200	MGCS36044_03512	***serS***			seryl-tRNA synthetase	-3.3	-3.6
201	MGCS36044_03052	***sepF***			cell division protein	-3.4	-2.8
202	MGCS36044_02372	***pfkA***			6-phosphofructokinase	-3.4	-2.0
203	MGCS36044_03920	***pgi***			Pgi family glucose-6-phosphate isomerase	-3.4	-2.7

No.	Locus tag	Gene	Virulence[(1)]	SignalP6[(2)]	Function	Fold in vitro ME/ in vivo[(3)]	Fold in vitro ES/ in vivo[(4)]
204	MGCS36044_02370	***pyk***			pyruvate kinase	-3.5	-2.1
205	MGCS36044_01558	***atpA***			ATP synthase alpha chain	-3.5	-4.0
206	MGCS36044_02888	***ptsH***			PTS transporter phosphocarrier protein	-3.7	-2.7
207	MGCS36044_03478	***cotS***			CotS family thiamine kinase	-3.7	-2.4
208	MGCS36044_01506	***eno***			phosphopyruvate hydratase -- enolase protein	-3.8	-4.0
209	MGCS36044_03050	***-***			YggT family protein	-3.8	-2.5
210	MGCS36044_02884	***gapN***			NADP-dependent glyceraldehyde-3-phosphate dehydrogenase	-3.9	-4.2
211	MGCS36044_03070	***typA***			translational GTPase	-3.9	-2.0
212	MGCS36044_03506	***manN***			PTS transporter mannose-specific IID component protein	-4.0	-3.4
213	MGCS36044_03650	***-***			DUF1129 domain-containing protein	-4.2	-2.0
214	MGCS36044_01308	***-***			DUF3270 domain-containing protein	-4.5	-2.9
215	MGCS36044_00308	***tyrS***			tyrosyl-tRNA synthetase	-4.6	-2.1
216	MGCS36044_01552	***atpB***			ATP synthase A subunit	-4.7	-4.8
217	MGCS36044_03504	***manM***			PTS transporter mannose-specific IIC component protein	-4.7	-3.2
218	MGCS36044_01508	***srrG***	Virulence		streptolysin S small regulatory RNA	-4.9	-4.6
219	MGCS36044_01354	***tufA***			translation elongation factor Tu protein	-5.5	-2.2
220	MGCS36044_03502	***manL***			PTS transporter mannose-specific IIB & IIA component protein	-6.0	-4.0
221	MGCS36044_00368	***srtC_1***			class C sortase	-6.2	-2.1
222	MGCS36044_01728	***apbA***			2-dehydropantoate 2-reductase	-6.2	-2.7
223	MGCS36044_00176	***rpsS***			30S ribosomal S19 protein	-6.3	-2.2
224	MGCS36044_04222	***marC***			MarC family small neutral amino acid transporter	-6.9	-2.7
225	MGCS36044_00366	***-***		Secreted	secreted pilin minor/ancillary protein	-7.3	-2.3
226	MGCS36044_04224	***mnmA***			tRNA 2-thiouridine(34) synthase	-7.4	-3.2
227	MGCS36044_00178	***rplV***			50S ribosomal L22 protein	-7.4	-2.0
228	MGCS36044_00210	***adk***			adenylate kinase protein	-7.4	-2.2
229	MGCS36044_03538	***phaB***			enoyl-CoA hydratase protein	-7.5	-2.9
230	MGCS36044_03798	***glnA***			glutamine synthetase	-7.6	-2.3
231	MGCS36044_04202	***rpsD***			30S ribosomal S4 protein	-7.9	-2.1
232	MGCS36044_04068	***treB***			PTS transporter trehalose-specific EIIBC component	-8.0	-12.6
233	MGCS36044_00174	***rplB***			50S ribosomal L2 protein	-8.6	-2.0
234	MGCS36044_04144	***rpmGA***			50S ribosomal L33 protein	-8.6	-2.3
235	MGCS36044_01726	***-***			PTS transporter subunit IIC	-8.7	-3.4
236	MGCS36044_01648	***rpmI***			50S ribosomal L35 protein	-9.3	-3.0
237	MGCS36044_02288	***rpsT***			30S ribosomal S20 protein	-9.4	-3.0
238	MGCS36044_01808	***-***			hypothetical protein	-9.5	-2.1
239	MGCS36044_01650	***rplT***			50S ribosomal L20 protein	-10.2	-3.6
240	MGCS36044_01646	***infC***			translation initiation factor	-11.3	-3.3
241	MGCS36044_00758	***fba_2***			fructose-bisphosphate aldolase	-13.9	-11.3
242	MGCS36044_03652	***rpsR***			30S ribosomal S18 protein	-14.4	-3.8
243	MGCS36044_03448	***srtB***			pilus polymerization class B sortase	-14.7	-7.8
244	MGCS36044_03446	***-***		Secreted	pilus ancillary/minor protein 2	-15.1	-7.6

No.	Locus tag	Gene	Virulence(1)	SignalP6(2)	Function	Fold in vitro ME/ in vivo(3)	Fold in vitro ES/ in vivo(4)
245	MGCS36044_03450	**-**		Secreted	pilus backbone/major protein	-20.4	-10.7
246	MGCS36044_00214	***rpmJ***			50S ribosomal L36 protein	-22.4	-2.3
247	MGCS36044_00216	***rpsM***			30S ribosomal S13 protein	-23.0	-4.4
248	MGCS36044_03808	***gapA***	Virulence		glyceraldehyde-3-phosphate dehydrogenase	-25.0	-4.2
249	MGCS36044_00220	***rpoA***			DNA-directed RNA polymerase alpha subunit	-25.6	-2.1
250	MGCS36044_00218	***rpsK***			30S ribosomal S11 protein	-32.0	-3.8
251	MGCS36044_01806	**-**			rli38 RNA	-68.5	-3.4
252	MGCS36044_00224	**-**			L17DE RNA	-100.0	-5.4
253	MGCS36044_01804	***rplL***			50S ribosomal L7/L12 protein	-106.8	-3.7
254	MGCS36044_00222	***rplQ***			50S ribosomal L17 protein	-113.7	-3.9

1,662 MGCS36044 genes with mapped reads from NHP 15 (*in vivo* sample) were ranked by transcript abundance, where the gene with the highest transcript abundance was assigned a rank = 1. Similarly, the same 1,662 MGCS36044 genes were also ranked by the mean transcript abundance calculated from four replicates grown *in vitro*, both at mid-exponential (ME) and early stationary (ES) growth phase. The *in vivo* sample was separately compared to both *in vitro* samples, and a ≥ 2-fold cutoff was used to assign differentially expressed genes. Genes are ordered by fold *in vitro*-ME/*in vivo*, from highest to lowest. Because rank=1 corresponds to the gene with the highest transcript abundance, those genes with positive fold values have higher transcript abundance *in vivo*, compared to *in vitro*.

(1) Virulence refers to putative virulence genes.
(2) Genes predicted by SignalP 6.0 to have an export signal sequence.
(3) Fold *in vitro*-ME/*in vivo* refers to the ratio between the rank number, based on transcript abundance for the mean RPKM value for 4 replicates grown *in vitro* at ME, and the rank number also based on transcript abundance for the replicate grown *in vivo*, collected from NHP 15.
(4) Same as above(3) but *in vitro* at ES instead of ME.
(5) Lipo, exported lipoprotein attached to the cell envelope.
(6) TCS, two-component system.

Table S8. Differentially expressed genes in clinical isolates with mutations in *fasB* or *fasC* compared to isolates lacking mutations in MTR or putative virulence genes

No.	Locus tag[(1)]	Signal6P predicted[(2)]	Virulence[(3)]	Gene	Function	Fold WT/Fas[(4)]
1	MGCS36044_03866		Virulence	*fasX*	FasBCA TCS[(5)] small regulatory RNA	**3.9**
2	MGCS36044_01422			*fbp2*	fructose-bisphosphatase	**3.9**
3	MGCS36044_00526	Secreted	Virulence	*ska*	secreted streptokinase	**3.2**
4	MGCS36044_04194			-	DUF4097 family protein	**3.0**
5	MGCS36044_03128			-	GNAT family N-acetyltransferase	**2.8**
6	MGCS36044_04192			-	DUF1700 domain-containing protein	**2.7**
7	MGCS36044_03126			*arcB*	ornithine carbamoyltransferase	**2.7**
8	MGCS36044_03130			*arcA*	arginine deiminase	**2.7**
9	MGCS36044_04196			-	YhgE/Pip domain-containing protein	**2.7**
10	MGCS36044_04190			*padR*	PadR family transcriptional regulator	**2.6**
11	MGCS36044_00724			-	aspartate kinase	**2.5**
12	MGCS36044_00752			*lrgB*	antiholin-like protein	**2.5**
13	MGCS36044_03512			*serS*	seryl-tRNA synthetase	**2.3**
14	MGCS36044_00154			*adhE*	bifunctional acetaldehyde-CoA/alcohol dehydrogenase	**2.2**
15	MGCS36044_03452			*sipA*	signal peptidase I	**2.2**
16	MGCS36044_00118			*purF*	amidophosphoribosyltransferase	**2.2**
17	MGCS36044_03454	Secreted		-	pilus ancillary/minor protein 1	**2.2**
18	MGCS36044_03124			*arcD*	arginine/ornithine antiporter protein	**2.2**
19	MGCS36044_03788			*oppF_2*	oligopeptide ABC transporter ATPase	**2.1**
20	MGCS36044_03450	Secreted		-	pilus backbone/major protein	**2.1**
21	MGCS36044_03446	Secreted		-	pilus ancillary/minor protein 2	**2.1**
22	MGCS36044_03448			*srtB*	pilus polymerization class B sortase	**2.1**
23	MGCS36044_00116			*purL*	phosphoribosylformylglycinamidine synthase	**2.1**
24	MGCS36044_03790			*oppD_2*	oligopeptide ABC transporter ATPase	**2.1**
25	MGCS36044_00120			*purM*	phosphoribosylformylglycinamidine cyclo-ligase	**2.0**
26	MGCS36044_03792			*oppC_2*	oligopeptide ABC transporter permease	**2.0**
27	MGCS36044_03118			*arcC*	carbamate kinase	**2.0**
28	MGCS36044_03794			*oppB_2*	oligopeptide ABC transporter permease	**1.9**
29	MGCS36044_02476			*manZ*	ManZ family PTS mannose/fructose IID component	**1.9**
30	MGCS36044_03796	Lipo[(6)]		*oppA_2*	oligopeptide ABC transporter substrate-binding lipoprotein	**1.9**
31	MGCS36044_00722			*comX_1*	competence protein	**1.8**
32	MGCS36044_00822			*comX_2*	competence protein	**1.8**
33	MGCS36044_04052			*potE*	PotE family amino acid transporter	**1.8**
34	MGCS36044_03966			*ulaD*	3-keto-L-gulonate 6-phosphate decarboxylase protein	**1.8**
35	MGCS36044_04048			*fhs_2*	formate--tetrahydrofolate ligase	**1.8**
36	MGCS36044_03122			*arcT*	M20 family metallodipeptidase	**1.8**
37	MGCS36044_02928			*nagB*	glucosamine-6-phosphate deaminase	**1.8**
38	MGCS36044_02478			*manY*	ManY family PTS mannose/fructose IIC component	**1.8**

No.	Locus tag[(1)]	Signal6P predicted[(2)]	Virulence[(3)]	Gene	Function	Fold WT/Fas[(4)]
39	MGCS36044_01530	Secreted		-	cell surface extracellular nuclease	**1.8**
40	MGCS36044_01144			-	lactobin A/cerein 7B family class IIb bacteriocin with double-glycine secretion leader peptide	**1.7**
41	MGCS36044_01480			*add*	adenosine deaminase Add	**1.7**
42	MGCS36044_02480			-	AgaB family mannose/fructose/N-acetylgalactosamine-specific component IIB	**1.7**
43	MGCS36044_01118			-	hypothetical protein	**1.7**
44	MGCS36044_04050			*hutD*	histidine uptake and utilization	**1.7**
45	MGCS36044_02482			*manX*	ManX family PTS mannose/fructose IIA component	**1.7**
46	MGCS36044_04054			*hutH*	histidine ammonia-lyase	**1.7**
47	MGCS36044_04046			-	formiminotetrahydrofolate cyclodeaminase	**1.7**
48	MGCS36044_03968			*ulaC*	ascorbate-specific PTS transporter EIIA protein	**1.7**
49	MGCS36044_04044			*fctD*	glutamate formiminotransferase	**1.7**
50	MGCS36044_01152			-	CAAX amino terminal protease family protein	**1.6**
51	MGCS36044_01218			*dhaM*	PTS-dependent dihydroxyacetone kinase phosphotransferase subunit	**1.6**
52	MGCS36044_01150			-	hypothetical protein	**1.6**
53	MGCS36044_01798			*clpL*	ATP-dependent Clp protease ATP-binding subunit	**1.6**
54	MGCS36044_02234			*asd*	aspartate-semialdehyde dehydrogenase	**1.6**
55	MGCS36044_00362		Virulence	*rofA*	pilus transcriptional regulator	**1.6**
56	MGCS36044_01216			*dhaL*	dihydroxyacetone kinase subunit	**1.6**
57	MGCS36044_01154			-	hypothetical protein	**1.6**
58	MGCS36044_03868		Virulence	*fasA*	signal transduction response regulator protein	**1.6**
59	MGCS36044_01220			*gla*	glycerol uptake facilitator protein	**1.6**
60	MGCS36044_01148			-	putative bacteriocin with double-glycine leader peptide	**1.6**
61	MGCS36044_02644			*malQ*	4-alpha-glucanotransferase (amylomaltase) protein	**1.5**
62	MGCS36044_03964			*ulaE*	L-xylulose 5-phosphate 3-epimerase	**1.5**
63	MGCS36044_02642			*glgP*	maltodextrin phosphorylase protein	**1.5**
64	MGCS36044_04198			-	hypothetical protein	**1.5**
65	MGCS36044_03962			*ulaF*	L-ribulose-5-phosphate 4-epimerase	**1.5**
66	MGCS36044_01214			*dhaK*	dihydroxyacetone kinase subunit	**1.5**
67	MGCS36044_03856			*nanE*	N-acetylmannosamine-6-phosphate 2-epimerase	**1.5**
68	MGCS36044_01516		Virulence	*sagC*	streptolysin S biosynthesis protein	**1.5**
69	MGCS36044_01518		Virulence	*sagD*	streptolysin S biosynthesis protein	**1.5**
70	MGCS36044_01754			*gdhA*	NADP-specific glutamate dehydrogenase	**1.5**
71	MGCS36044_01526		Virulence	*sagH*	streptolysin S export permease protein	**1.5**
72	MGCS36044_01528		Virulence	*sagI*	streptolysin S export permease protein	**1.5**
73	MGCS36044_01514		Virulence	*sagB*	streptolysin S biosynthesis protein	**1.5**
74	MGCS36044_03854	Lipo		*ugpB_2*	carbohydrate ABC transporter substrate-binding lipoprotein UgpB-like	**1.5**
75	MGCS36044_03852			*ugpA_2*	carbohydrate ABC transporter permease UgpA-like	**1.5**
76	MGCS36044_01520		Virulence	*sagE*	streptolysin S self-immunity protein	**1.5**

No.	Locus tag(1)	Signal6P predicted(2)	Virulence(3)	Gene	Function	Fold WT/Fas(4)
77	MGCS36044_03970			*ulaB*	ascorbate-specific PTS transporter EIIB protein	**1.5**
78	MGCS36044_00500			*rgfB*	endonuclease/exonuclease/phosphatase family protein	**1.5**
79	MGCS36044_01142			-	BlpM-like bacteriocin with double-glycine leader peptide	**1.5**
80	MGCS36044_00326	Pilin		*comYD*	competence system type II secretion system GspH family protein	**1.5**
81	MGCS36044_01522		Virulence	*sagF*	streptolysin S biosynthesis protein	**1.5**
82	MGCS36044_00518	Secreted		-	cell surface UshA family bifunctional 2',3'-cyclic-nucleotide 2'-phosphodiesterase/3'-nucleotidase	**1.5**
83	MGCS36044_01146			-	putative bacteriocin with double-glycine leader peptide	**1.5**
84	MGCS36044_01524		Virulence	*sagG*	streptolysin S export protein	**1.5**
85	MGCS36044_01134		Virulence	*silCR*	streptococcal invasion locus auto-inducing pheromone peptide	**1.5**
86	MGCS36044_04026			-	cspA RNA	**-1.5**
87	MGCS36044_02956			*metP_2*	methionine uptake ABC transporter permease	**-1.5**
88	MGCS36044_00382			*ridA*	RidA family protein	**-1.5**
89	MGCS36044_02548			*acoC*	dihydrolipoamide acetyltransferase	**-1.5**
90	MGCS36044_02550			*acoB*	pyruvate dehydrogenase E1 component beta subunit	**-1.5**
91	MGCS36044_03360	Secreted		*hisJ*	HisJ family amino acid ABC transporter substrate-binding secreted protein	**-1.5**
92	MGCS36044_03682			*pflB*	formate C-acetyltransferase	**-1.5**
93	MGCS36044_02544			*acoL*	dihydrolipoyl dehydrogenase	**-1.5**
94	MGCS36044_03288			*cysK*	cysteine synthase A	**-1.5**
95	MGCS36044_02954	Lipo		*metQ*	methionine uptake ABC transporter substrate-binding lipoprotein	**-1.5**
96	MGCS36044_01126		Virulence	*silA*	TCS DNA-binding response regulator	**-1.5**
97	MGCS36044_01266			*bglA*	6-phospho-beta-glucosidase	**-1.5**
98	MGCS36044_02552			*acoA*	Pyruvate dehydrogenase E1 component alpha subunit	**-1.5**
99	MGCS36044_02546			-	hypothetical protein	**-1.5**
100	MGCS36044_02866	Lipo	Virulence	*slr*	InlA-like streptococcal leucine rich lipoprotein	**-1.6**
101	MGCS36044_03404			*ypbQ*	YpbQ family isoprenylcysteine carboxyl methyltransferase family protein	**-1.6**
102	MGCS36044_01734			*fruA*	fructose-specific PTS transporter EIIC component	**-1.6**
103	MGCS36044_02162	Lipo		-	FMN-binding protein, major membrane immunogen, membrane-anchored lipoprotein	**-1.6**
104	MGCS36044_01732			*fruK*	1-phosphofructokinase	**-1.6**
105	MGCS36044_01262			*bglG_1*	transcriptional antiterminator	**-1.7**
106	MGCS36044_01730			*fruR*	fructose operon transcriptional repressor protein	**-1.7**
107	MGCS36044_04028			*cspA*	cold-shock protein	**-1.7**
108	MGCS36044_03986			-	hypothetical protein	**-1.7**
109	MGCS36044_01264			*bglF*	PTS beta-glucoside transporter IIBCA component	**-1.8**
110	MGCS36044_01774			-	HTH domain-containing putative transcriptional regulator	**-1.8**
111	MGCS36044_00516	Secreted	Virulence	*emm*	cell surface M protein	**-2.0**

No.	Locus tag[1]	Signal6P predicted[2]	Virulence[3]	Gene	Function	Fold WT/Fas[4]
112	MGCS36044_03908	Secreted	Virulence	*fbpB*	cell surface fibronectin binding protein (B)	**-2.1**
113	MGCS36044_03912			-	Mga-related helix-turn-helix domain-containing transcriptional regulator	**-2.5**
114	MGCS36044_02058	Secreted	Virulence	*scpA*	cell surface extracellular C5a peptidase	**-2.6**
115	MGCS36044_02060			-	SSRC38 RNA	**-2.7**
116	MGCS36044_03994	Secreted	Virulence	*slo*	streptolysin O	**-2.9**
117	MGCS36044_03998	Secreted	Virulence	*nga*	NAD-glycohydrolase Nga	**-2.9**
118	MGCS36044_03996		Virulence	*ifs*	NAD-glycohydrolase inhibitor	**-3.1**
119	MGCS36044_01776	Secreted		-	extracellular cell wall anchored mucin-binding (MucBP) domain-containing protein	**-18.8**

Strains MGCS35798, MGCS35844, MGCS35978, and MGCS36071, lacking mutations in *vir* and major transcriptional regulator (MTR) genes were grown *in vitro*, in rich media, collected at the mid-exponential phase (OD600 = 1), and the transcriptome was compared to that of strains MGCS35873*fasC*, MGCS35893*fasB*, and MGCS35983*fasC*. The mean RPKMs were calculated for four strains lacking mutation in vir or MTR genes and the three strains with mutation in either *fasB* or *fasC*. 119 genes were differentially expressed (DE) between both strain groups. Fold cutoff is ≥1.5-fold.

(1) Locus tags taken from the reference strain, MGCS36044.
(2) Genes predicted by SignalP 6.0 to have an export signal sequence.
(3) Virulence refers to putative virulence genes.
(4) Fold refers to the ratio between the mean of RPKMs for four strains lacking mutation in vir or MTR genes (WT) and the mean of RPKMs for the three *fas* strains.
(5) TCS, two-component system.
(6) Lipo, exported lipoprotein attached to the cell envelope.
