# Supplementary material for: Analysis of Streptococcus dysgalactiae subspecies equisimilis gene transcripts during experimental primate necrotizing myositis
Source: mBio. 2025 Jul 22;16(8):e01349-25. doi: 10.1128/mbio.01349-25 (PMC12345147; doi:10.1128/mbio.01349-25)
Supplement: Supplemental Figures — Figures S1 to S3. [file mbio.01349-25-s0002.pdf]

SUPPLEMENTAL FIGURES LEGENDS

Figure S1. **Sample collection for RNAseq. A.** ***in vivo*** **samples.** Eight NHPs (numbered 9-16) were infected in either leg with MGCS36044 (right leg) and MGCS36089 (left leg), and five biopsies corresponding to concentrical layers from the inoculation point (layer 1-L1) to the outermost layer (layer 5-L5) were collected for each NHP and strain. The total number of biopsies was 40 (8 x 5 layers) per strain. **B.** ***in vitro*** **samples**. Strains MGCS36044 and MGCS36089 were grown *in vitro*, in rich media (THY) and in quadruplicate. Samples for RNA extraction were collected at two time points, at the mid-exponential phase (ME, OD=1), and at the early stationary phase (ES, OD=2).

Figure S2A. **Total number of sequencing reads obtained during the initial round of sequencing**. For each of the eight NHPs (numbered 9-16), cDNA libraries were obtained from five biopsies corresponding to concentrical layers going from the inoculation point (layer 1-L1) to the outermost layer (layer 5-L5) for both strains. The total number of cDNA libraries was 80 (8 NHPs x 5 layers x 2 strains). Raw reads refer to the total number of sequencing reads identified using FastQC. **A**. MGCS36044 reads. **B.** MGCS36089 reads.

Figure S2B. **Total number of sequencing reads corresponding to biopsy layers from 6 NHPs**. cDNA libraries corresponding to biopsy layers, one or two per NHP, were selected for re-sequencing based on the original number of reads

mapping to the pathogen. Only biopsies from six out of the eight original NHPs were selected. In total, cDNA libraries corresponding to nine biopsies (nine layers) per strain were chosen. The X axis represents the NHP number and the biopsy (layer) chosen for re-sequencing. Shown are the total number of sequencing reads identified with FastQC corresponding to MGCS36044 (four additional sequencing runs) and to MGCS36089 (three additional sequencing runs). **A.** The MGCS36044 cDNA libraries re-sequenced are shown as "**NHP**-layer(s)": **9**-L2-L3, **11**-L3-L4, **13**-L1, **14**-L1, **15-**L1-L2, and **16**-L1. **B.** Corresponding MGCS36044 reads mapping to the *Macaca fascicularis* genome assembly. **C.** The MGCS36089 cDNA libraries were: **9**-L1, **10**-L1-L2, **12**-L1, **13**-L1-L2, **14**-L1-L4, and **15**-L1. **D.** Corresponding MGCS36089 reads mapping to the *M. fascicularis* genome assembly.

Figure S3. **Correspondence between gene transcript abundance and number of SDSE mapped reads**. Random progressive downsizing of a fastq file with approximately 28 M reads corresponding to one replicate from MGCS36044 grown *in* vitro generated 22 new fastq files. The reads in each of these fastq files were mapped to MGCS36044 and the number of genes with mapped reads were identified. Also, transcript abundance quartiles based on RPKMs were calculated for the original file. There were 516 genes in the highest transcript abundance (Q1). **A**. Number of genes with mapped reads corresponding to the 22 progressively downsized files (black columns) in decreasing order. For each file the numbers of genes are shown in the X axis. Downsized file numbers are

shown above the columns. The corresponding number of original 516 Q1 genes present in each of the 22 files are shown in grey columns. All 516 Q1 genes were present in the first 10 files, ranging from 2062 to 791 total genes. **B**. R^2 values corresponding to pairwise comparisons of downsized files to the original file containing 28 M reads. Corresponding file numbers (1-22) are shown above each column. **C**. Number of reads corresponding to the downsized fastq files.

Figure S1

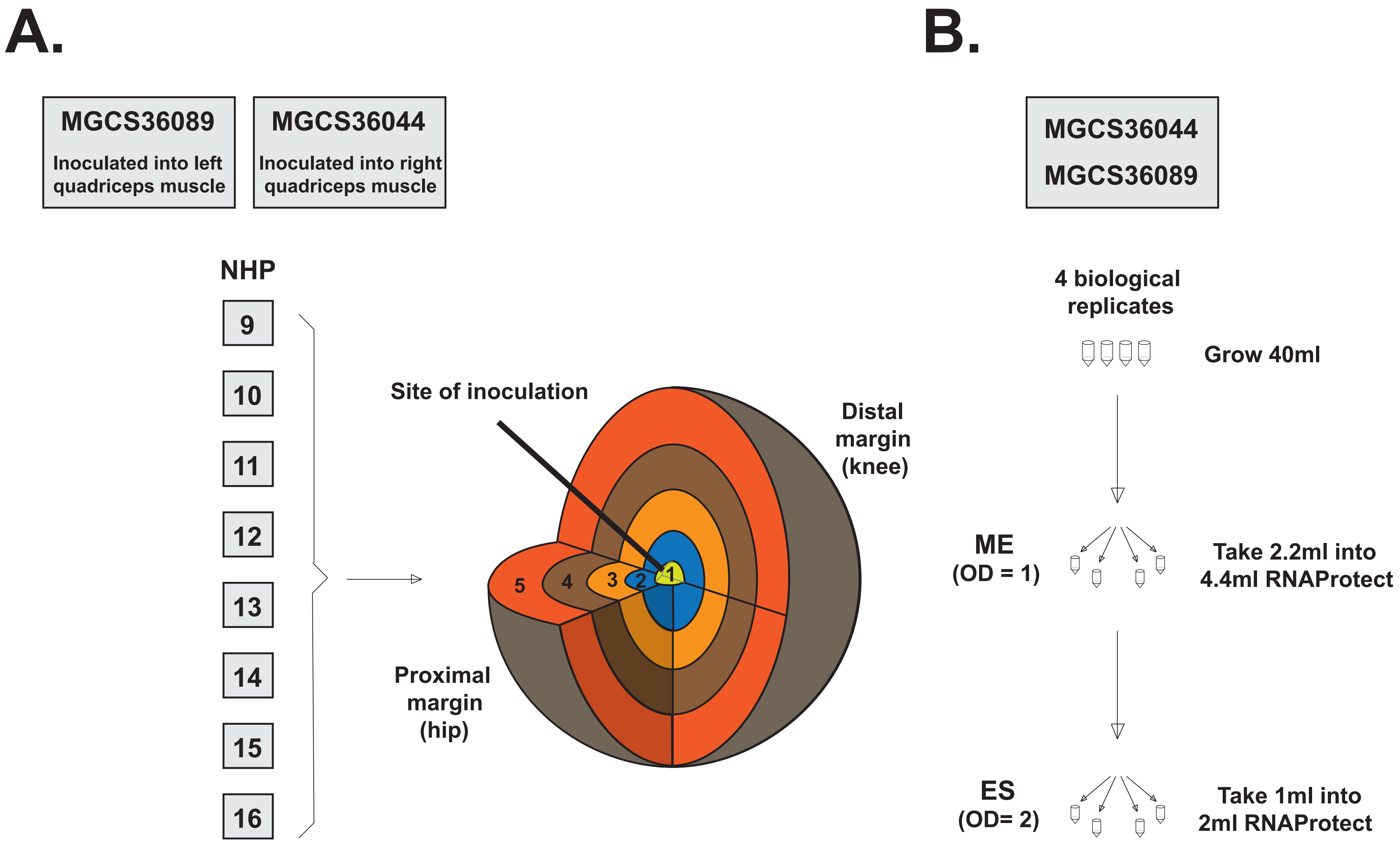
A.
MGCS36089
Inoculated into left quadriceps muscle
MGCS36044
Inoculated into right quadriceps muscle
NHP
9
10
11
12
13
14
15
16
Site of inoculation
Distal margin (knee)
Proximal margin (hip)
5
4
3
2
1
B.
MGCS36044
MGCS36089
4 biological replicates
Grow 40ml
ME (OD = 1)
Take 2.2ml into 4.4ml RNAProtect
ES (OD= 2)
Take 1ml into 2ml RNAProtect

Figure S2A

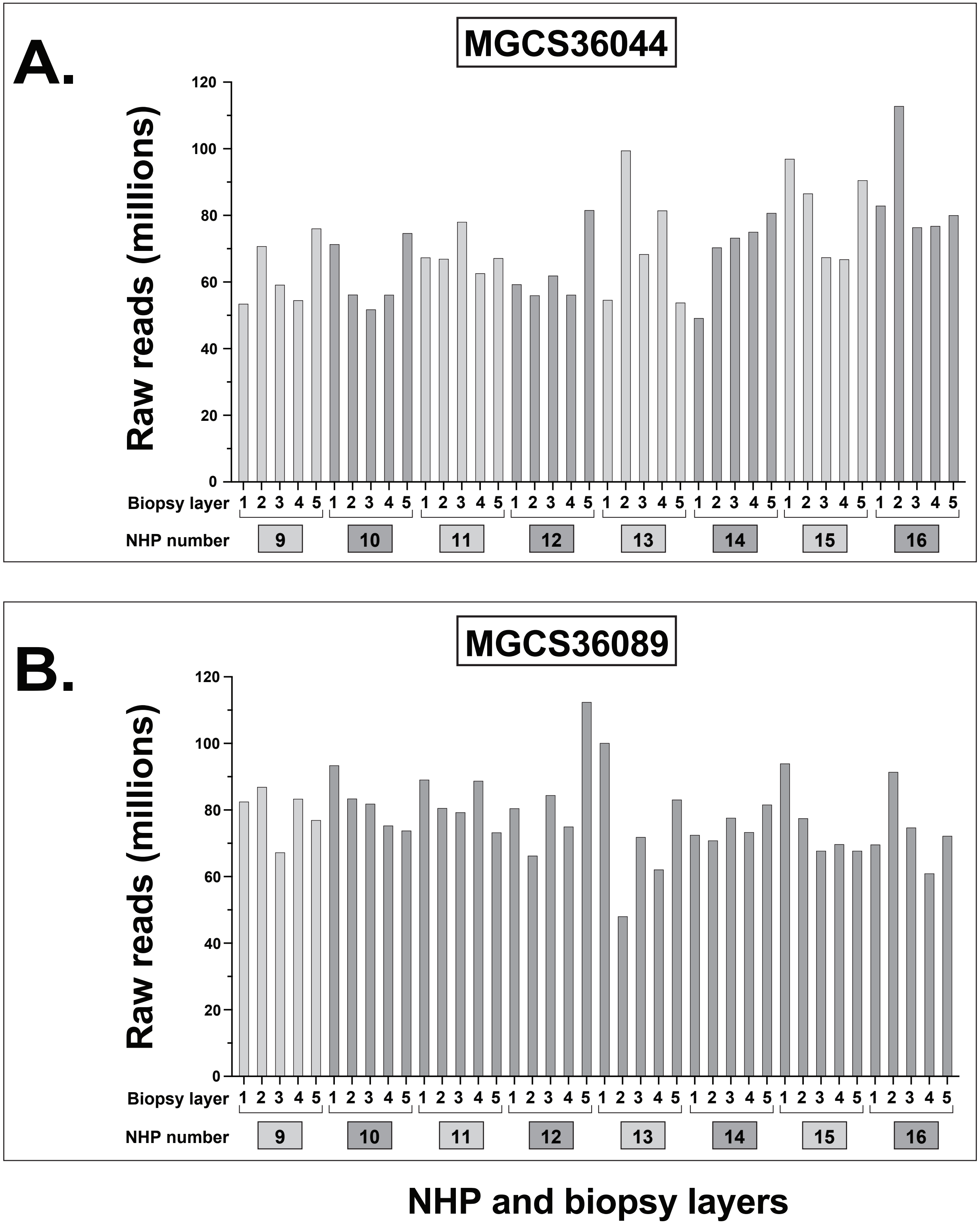
A.
MGCS36044
Raw reads (millions)
120
100
80
60
40
20
0
Biopsy layer
1 2 3 4 5 1 2 3 4 5 1 2 3 4 5 1 2 3 4 5 1 2 3 4 5 1 2 3 4 5 1 2 3 4 5 1 2 3 4 5
NHP number
9
10
11
12
13
14
15
16
B.
MGCS36089
Raw reads (millions)
120
100
80
60
40
20
0
Biopsy layer
1 2 3 4 5 1 2 3 4 5 1 2 3 4 5 1 2 3 4 5 1 2 3 4 5 1 2 3 4 5 1 2 3 4 5 1 2 3 4 5
NHP number
9
10
11
12
13
14
15
16
NHP and biopsy layers

Figure S2B

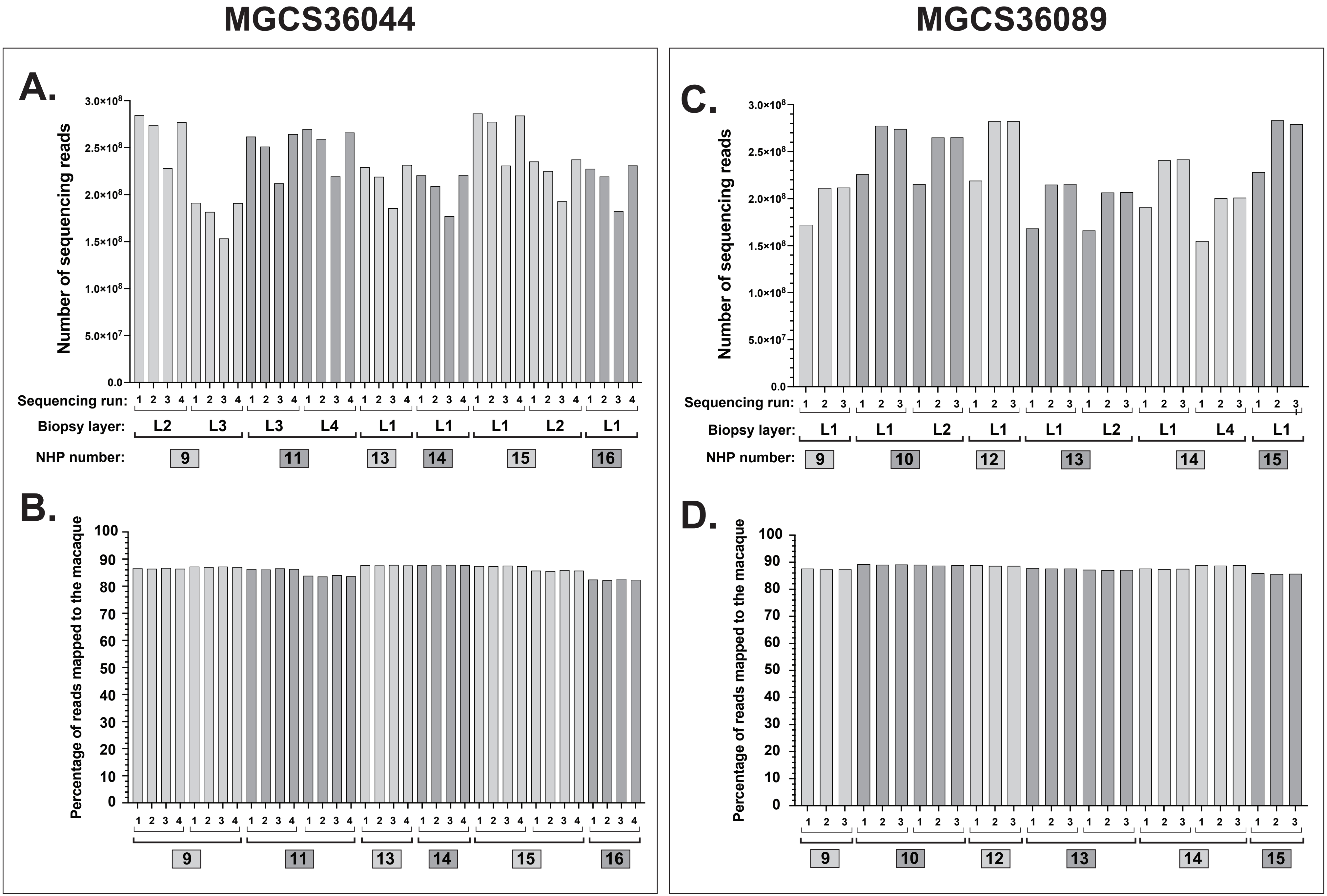
MGCS36044
MGCS36089
A.
Number of sequencing reads
3.0×10^8
2.5×10^8
2.0×10^8
1.5×10^8
1.0×10^8
5.0×10^7
0.0
Sequencing run:
Biopsy layer:
L2 L3 L3 L4 L1 L1 L1 L2 L1
NHP number:
9 11 13 14 15 16
B.
Percentage of reads mapped to the macaque
100 90 80 70 60 50 40 30 20 10 0
9 11 13 14 15 16
C.
Number of sequencing reads
Sequencing run:
Biopsy layer:
L1 L1 L2 L1 L1 L2 L1 L4 L1
NHP number:
9 10 12 13 14 15
D.
Percentage of reads mapped to the macaque
9 10 12 13 14 15

Figure S3

A.

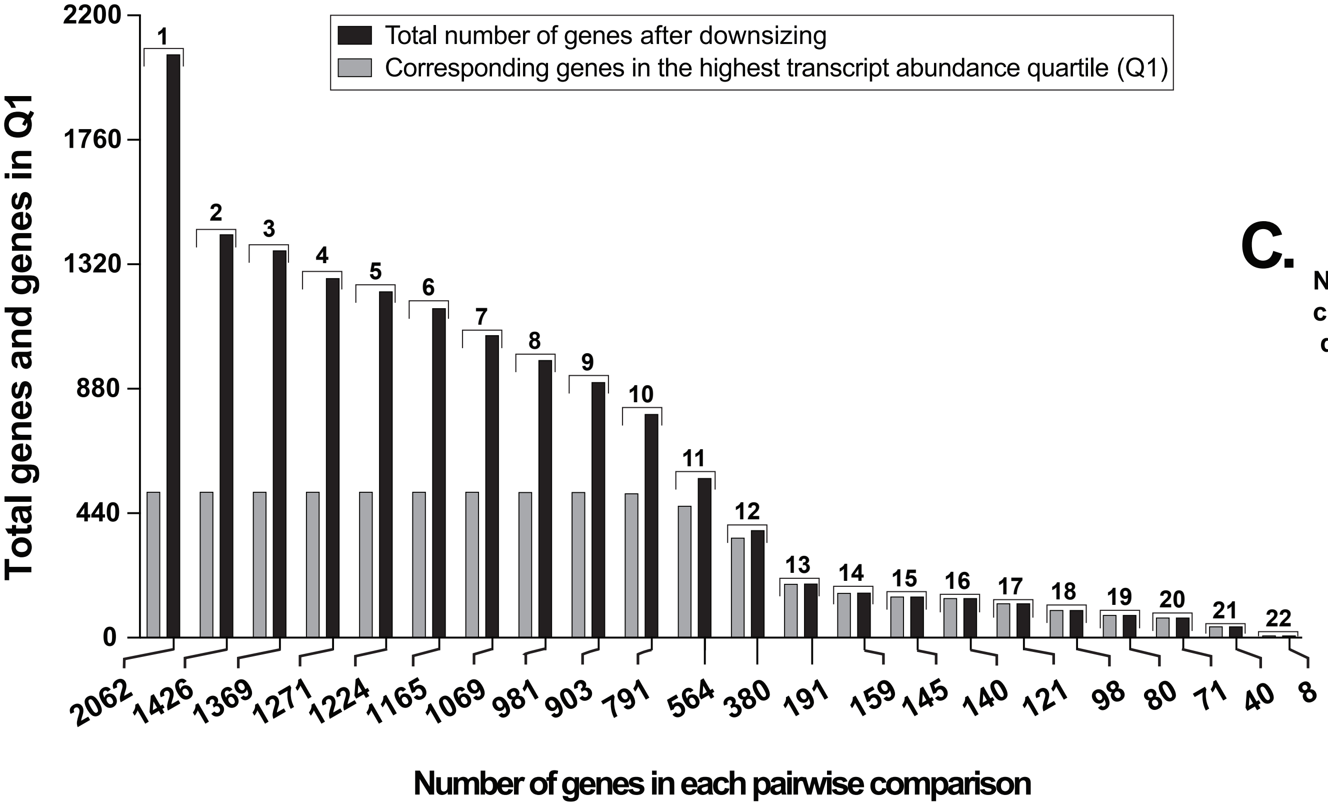

B.

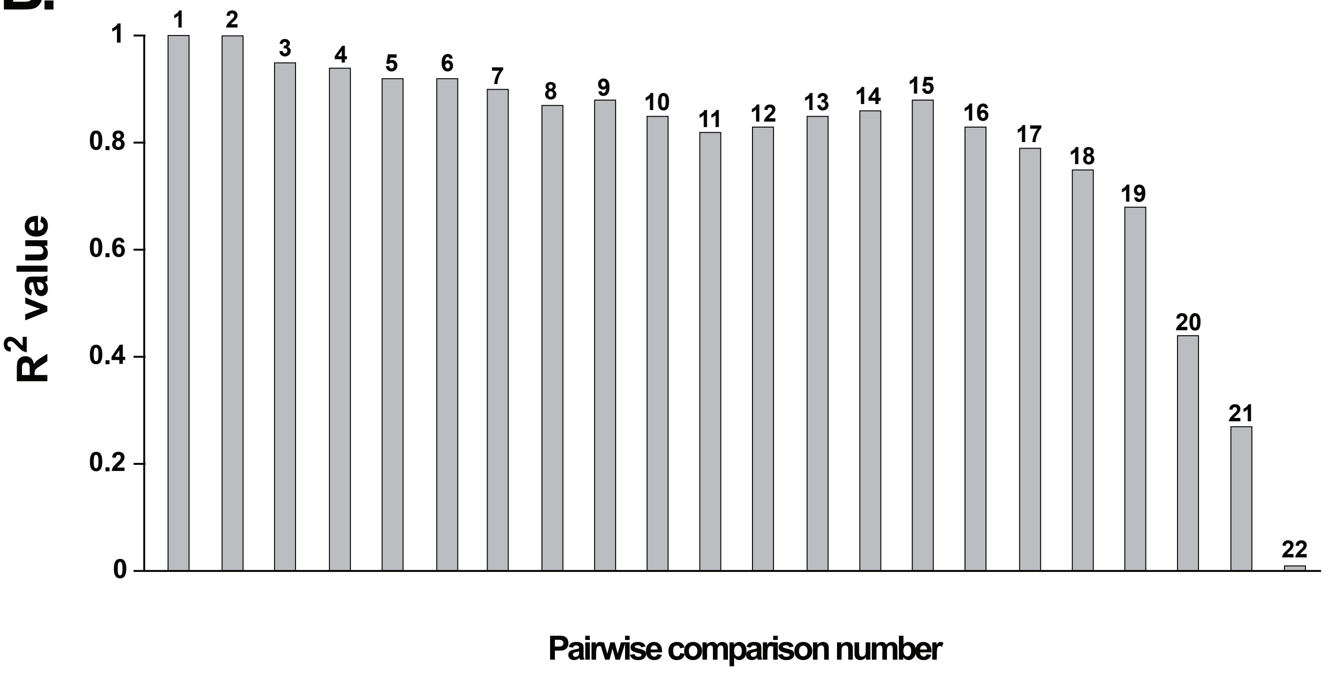

C.

Number of reads corresponding to downsized files

1- 2,000,000
2- 150,000
3- 125,000
4- 100,000
5- 90,000
6- 80,000
7- 70,000
8- 60,000
9- 50,000
10- 40,000
11- 30,000
12- 20,000
13- 10,000
14- 9,000
15- 8,000
16- 7,000
17- 6,000
18- 5,000
19- 4,000
20- 3,000
21- 2,000
22- 1,000
